# A microfluidic optimal experimental design platform for forward design of cell-free genetic networks

**DOI:** 10.1038/s41467-022-31306-3

**Published:** 2022-06-24

**Authors:** Bob van Sluijs, Roel J. M. Maas, Ardjan J. van der Linden, Tom F. A. de Greef, Wilhelm T. S. Huck

**Affiliations:** 1 Institute for Molecules and Materials, Radboud University, Nijmegen, The Netherlands; 2 Laboratory of Chemical Biology, Department of Biomedical Engineering, Eindhoven University of Technology, Eindhoven, The Netherlands; 3 Institute for Complex Molecular Systems, Eindhoven University of Technology, Eindhoven, The Netherlands; 4 Computational Biology Group, Department of Biomedical Engineering, Eindhoven University of Technology, Eindhoven, The Netherlands; 5 Center for Living Technologies, Eindhoven-Wageningen-Utrecht Alliance, Eindhoven, The Netherlands

**Keywords:** Expression systems, Computational platforms and environments

## Abstract

Cell-free protein synthesis has been widely used as a “breadboard” for design of synthetic genetic networks. However, due to a severe lack of modularity, forward engineering of genetic networks remains challenging. Here, we demonstrate how a combination of optimal experimental design and microfluidics allows us to devise dynamic cell-free gene expression experiments providing maximum information content for subsequent non-linear model identification. Importantly, we reveal that applying this methodology to a library of genetic circuits, that share common elements, further increases the information content of the data resulting in higher accuracy of model parameters. To show modularity of model parameters, we design a pulse decoder and bistable switch, and predict their behaviour both qualitatively and quantitatively. Finally, we update the parameter database and indicate that network topology affects parameter estimation accuracy. Utilizing our methodology provides us with more accurate model parameters, a necessity for forward engineering of complex genetic networks.

## Abstract (web summary)

Characterization of cell-free genetic networks is inherently difficult. Here the authors use optimal experimental design and microfluidics to improve characterization, demonstrating modularity and predictability of parts in applied test cases.

## Introduction

Cell-free protein synthesis (CFPS), either using purified components^1^ or based on cell lysates^2^, was originally used as a method to express proteins toxic to the host cell^3^. However, rapid progress in the past decades has shown that CFPS is a promising technology for the creation of biosensors^4,5^, point-of-care expression platforms^6,7^, for educational purposes^8^, and as an essential component for artificial cells^9–13^. In addition, CFPS has been widely used as a molecular “breadboard” for prototyping synthetic genetic circuits and cell-free metabolic engineering^14–17^. To this end, Noireaux and co-workers introduced the *E. coli* transcription translation (TX-TL) Toolbox 1.0 to 3.0 as general CFPS systems built around the endogenous σ-factors of *E. coli*^18–21^. These systems allow for the expression of hundreds of genes simultaneously and are ideal for the creation of synthetic genetic networks^22^. To be able to design larger and more complex cell-free genetic networks, this platform has been extended to include other natural and synthetic transcription factors^4,15,23–26^, riboregulators^27–30^ and dCas9-based repressors which offer the possibility to block any promoter without the need for repressor specific operator sequences^31^.

However, despite the increased availability of a large number of genetic building blocks, construction of complex cell-free genetic circuits has remained slow. Forward design of gene networks has proven difficult due to the lack of precision provided by conventional kinetic characterization methods and insufficient considerations regarding the retroactivity of parts at the system-level, thus rendering the building block not mutually interchangeable or reusable in novel network settings^32–37^.

Several groups have attempted to improve the quality of the kinetic data using batch CFPS systems^14,38,39^, and by studying and modelling transcription and translation reactions separately by measuring both time-resolved mRNA and protein dynamics. Despite these efforts, the predictive power of the resulting models and estimated parameters has remained low, preventing forward design of cell-free circuits. Besides, genetic building blocks are studied either in isolation, or in relatively simple two-gene systems. Recently, Singhal et al. further improved on this work by establishing a computational toolbox for the characterization of genetic building blocks in batch CFPS^40^. They characterized different regulatory interactions of an incoherent feedforward loop (IFFL) in isolation. However, despite a rather involved computational and experimental process, parameters were still mostly covariant and thus unidentifiable.

A fundamental limitation in previous work has been the use of batch systems, where—due to the short expression lifetime and changing transcription and translation rates resulting from the depletion of nutrients—only the first few hours of expression contain information about the reaction kinetics. Batch systems also preclude the study of more complex genetic networks with steady-state behaviour, as reaction products accumulate and nutrients are depleted. Microfluidic systems, in which the reaction media is continuously refreshed, offer long-term protein expression, operate under steady-state kinetics and allow for precise control over DNA concentrations, dilution rates, and the concentration of cellular resources^41–44^.

Microfluidic systems allowed the construction of regulatory networks exhibiting complex behaviour^15,45–48^, Niederholtmeyer et al. studied numerous ring oscillators where the qualitative and quantitative performance of the oscillators in vitro reflect those in in vivo conditions, demonstrating the potential of a cell-free biochemical breadboard^15^. However, similar to conventional batch reactions, the covariance between parameters prevents accurate parameter estimation and thereby also prevents prediction of the phase space of the functional output of complex gene networks in microfluidic systems. To solve these problems and enable forward engineering of cell-free genetic networks, we present a kinetic parameter extraction methodology, where we combine the advantages of CFPS, microfluidics and optimal experimental design (OED)^49,50^ to extract parameters from complete synthetic genetic networks and build a database of characterized building blocks. This database is then used to design two synthetic genetic networks.

**Fig. 1:**
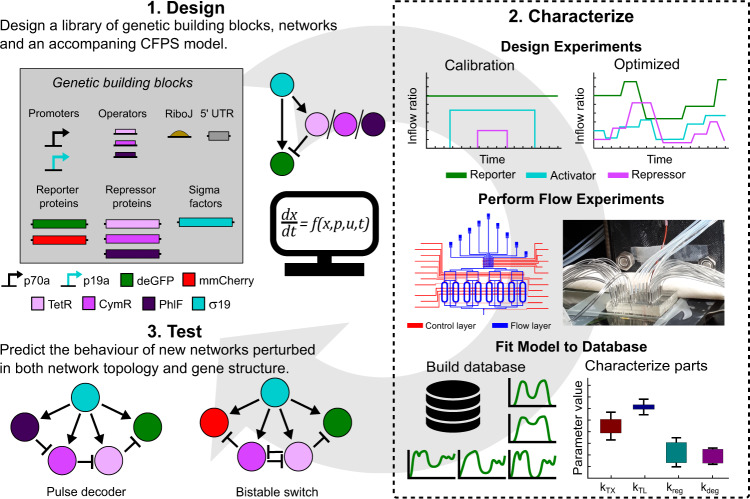
Overview of the design–characterize–test pipeline. (1) IFFLs are assembled from a library of genetic building blocks. All IFFLs share the same activator but differ in the repressors. Detailed gene structures and names are provided in Fig. 2. Besides, a CFPS ODE model is formulated to describe all reactions (Eqs. 3–9 in the Supplementary Information). (2) Calibration and optimized inflow patterns are created (top) and used to perform CFPS experiments in microfluidic chemostats (middle). Schematic representation of the microfluidic device (left) and a picture of a single device plugged in and ready for an experiment (right) are shown. All experiments are combined in a database and we estimate the parameter values and distributions using the associated kinetic models. (3) Using the same library of building blocks, two new networks are assembled and are used as test cases to assess the predictive power of the estimated parameters and model. General overview of the network topology is shown. Detailed gene structures and names are provided in Figs. 4 and 5.

Our methodology is based on a design–characterize–test cycle (Fig. 1), in which we combine microfluidics, optimal experimental design (OED) and optimize a kinetic model of the CFPS process with an agent-based non-linear least-squares optimization routine which utilizes all collected experimental data simultaneously. We first design a library of genetic building blocks containing promoters, operators, a RiboJ sequence, a 5′ UTR (5′ untranslated region), which contains the ribosomal binding site (RBS), and open reading frames (ORFs). Using this library, we designed six incoherent type 1 feed-forward loops (IFFL), that share an activator gene but have different repressors (Fig. 1, step 1). We develop ordinary differential equation (ODE) models of the cell-free IFFL circuits based on Michaelis-Menten and Hill kinetics (Supplementary Information 2).

Next, we move to the characterize phase where we characterize the library of genetic building blocks in three steps (Fig. 1, step 2). First, we perform calibration experiments and use these to optimize a set of control inputs for the IFFL motif (Fig. 1, step 2). The calibration and optimized CFPS experiments are performed in microfluidic chemostats, similar to the ones used by Van der Linden et al.^44^ and Yelleswarapu et al.^47^ (Fig. 1, step 2). All experimental results are then assembled in a database, which we use to obtain parameter sets for the model by simultaneously fitting it to all experimental results in the database (Fig. 1, step 2). Finally, in the test phase, we test the parameter sets and model by predicting the behaviour of two cell-free genetic networks perturbed both in network topology and gene structure (Fig. 1, step 3). The predictions demonstrate that this pipeline opens up the forward design of complex cell-free genetic networks by demonstrating modularity of the genetic building blocks.

## Results

### Designing a library of genetic building blocks and assembly of IFFLs

**Fig. 2:**
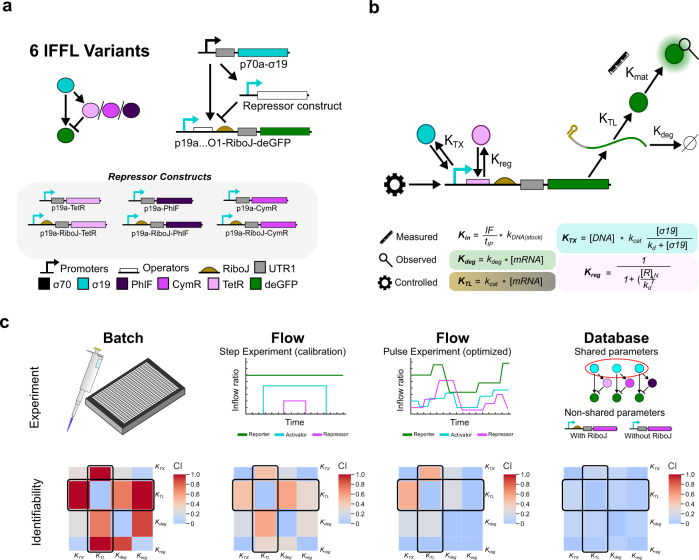
Design of the IFFL networks, model and in silico analysis of parameter identifiability. **a** IFFL variants considered in this study. We used 3 repressor constructs, each of which has a version which includes/excludes a RiboJ sequence. The reporter constructs bear a single operator site for the corresponding repressor downstream of the transcriptional start site. **b** Model describing the CFPS process of a gene. Maturation rates of deGFP and mmCherry are known. Concentrations of the genetic construct over time are controlled, a single protein reporter is observed. All parameters are lumped into four categories (*K*_TX_, *K*_TL_, *K*_reg_ and *K*_deg_), general expressions for all four are provided. **c** An identifiability analysis based on Gábor et al.^33^. The inverse of smallest eigenvalue from the single value decomposition analysis is taken, called the collinearity index, this corresponds to the degree of covariance between parameters for the simulations. Covariance between parameters reduces from batch to flow, step flow to optimized flow and is lowest when all simulations (i.e. whole database) are included in the fitting process. The parameters shared between experiments in the final dataset become a point of information as the difference in expression rates can solely be attributed to non-overlapping parameters such as an identical experiment with and without a RiboJ sequence in the construct. The pairwise collinearities between *K*_TL_ and all other parameters are highlighted by the black boxes.

First, we designed a library of modular genetic building blocks encompassing promoters, operators and open reading frames (ORFs), that allow for the fast assembly of genes in a mix-and-match fashion. Therefore, we created a small library with 2 promoters, 3 operators, a RiboJ sequence^51^, an untranslated region (UTR1) containing a strong ribosomal binding site (RBS)^19^ and 6 ORFs transcribing for 6 different proteins (Fig. 1, step 1). All building blocks have specific overhangs, which were used to assemble them into a vector containing the T500 terminator as described by Sun et al. (see ‘Methods’)^52^. Operators for repressors were inserted downstream of the transcriptional start site^53,54^ and a RiboJ insulator sequence was added in between the operator and the UTR1 to prevent any effect of the operator sequence on translation initiation^51,55^. We subsequently used this library to design six IFFLs (Fig. 2a). An IFFL combines direct activation with delayed inhibition reactions and consists of a single activator activating both a reporter and repressor gene. The repressor in turn inhibits the expression of the reporter resulting in a pulse-like response. In our case, the IFFLs are activated by the p70a-σ19 gene. Besides, each IFFL has a unique repressor construct including a TetR, PhlF or CymR repressor which corresponds to a reporter construct bearing the corresponding operator site. Finally, each individual repressor construct has two configurations, either containing a RiboJ sequence or not.

### Model

To describe the network behaviour, we make use of a coarse-grained model description of the different processes taking place during CFPS. In this model, which is defined by a set of ordinary differential equations (ODEs) (see Supplementary Information 2, Eqs. 3–9), we aim to balance the fidelity of the mechanistic description and the identifiability of the most sensitive parameters. Figure 2b shows the five processes determining gene expression in our microfluidic chemostats. *K*_in_ is a control parameter and models the time-dependent concentration of DNA in each reactor based on the inflow rate where *IF* (the fraction of the replaced reactor volume) divided by *t*_IP_ (min^−1^) the time per input controls how much of the stock concentration, *k*_DNA(stock)_ (nM), flows into the reactor. *K*_TX_ models transcription based on the DNA concentration and a sigma-factor dependent *k*_d_ (nM) and *k*_cat_ (nM min^−1^). We assume that the hybrid promoters, with both σ19 and repressor recognition sites, have the same dissociation constant as the bare promoter (Supplementary Fig. 10). Parameter *K*_TL_ models translation linearly proportional to the mRNA concentration using a RiboJ and gene-specific translation rate *k*_cat_ (nM min^−1^). Parameter *K*_reg_ models the repressor regulation using Hill kinetics with a specific Hill coefficient and *k*_d_ (nM*)* for each repressor. Finally, mRNA degradation is modelled by a linear relationship by *K*_deg_ with a mRNA-dependent degradation rate *k*_deg_ (min^−1^). For the degradation of proteins in the lysate we assume that the half-life >> dilution and is therefore not included. The maturation rates (*K*_mat_) of deGFP and mmCherry were measured directly using a previously established method (Supplementary Fig. 11)^42^.

We make three assumptions about the CFPS process in this model. First, since we determined minimal competition for ribosomes between genes and between sigma-factors for core RNA polymerase (Supplementary Fig. 12), we omitted resource competition in our model. Second, Noireaux et al. demonstrated that σ70 is present at saturating conditions in the lysate^38^, is not limiting and remains constant throughout the experiment. Therefore, we modelled transcription from p70a promoter as a linear term with a single *k*_cat_. Lastly, based on values from Noireaux et al.^38^, who were able to define a lower bound for the concentration of mRNA, we set a soft constraint on the allowed minimum and maximum levels of mRNA in the flow reactor for all activated promoters where the minimum concentration of mRNA at steady state (in flow) is at least double that of its corresponding genetic construct according to min([*mRNA*]_*ss*_) > 2*[*DNA*]_*ss*_ and at most max([*mRNA*]_*ss*_) < 500*[*DNA*]_*ss*_.

### OED and in silico identifiability analysis

In our work, we used optimal experimental design (OED) to increase the information density about the parameters in of the experimental data resulting in optimal identifiability of the model parameters. We do this by optimizing the experimental inflow patterns in such a way that the covariance between parameters in our model is minimized. Covariance between parameters can often be broken by testing specific combinations of control inputs, which can be identified by the OED^56^. In our case, this results in a specific inflow pattern for each DNA construct of the IFFL network, leading to a multitude of different concentrations of the three DNA constructs being tested in a single experiment (Fig. 2c, 3rd panel). A full description of the software toolboxes and computational pipeline, together with a detailed explanation of the implementation of OED and model fitting, are provided in the Supplementary Information.

For optimal experimental design to work we need ‘appropriate’ initial parameter values. This stems from the fact that the parameter sensitivities that make up the Fischer information matrix^57^ are dependent on the parameter values; thus, the covariance changes along with the chosen parameter set^57^. Intuitively, we can imagine a scenario where an initial guess for the repressive strength of the three repressors is too far from its true in vitro counterpart and the repressor template concentration is either too high (there is no transcription) or too low (there is no repression) during an experiment. This renders the system non-responsive to timely changes in the repressor construct concentration and we do not gain any information (In Supplementary Information 3 and 4 we demonstrate the effect of specific input pattern combinations in a tutorial). Therefore, the first experiments we performed were step calibrations (Fig. 2c second panel and Supplementary Fig. 13).

**Fig. 3:**
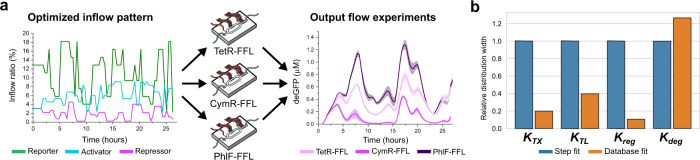
Experimental pipeline and parameter estimation results. **a** Overview of the experimental process with the optimized inflow pattern (left) and output for all three IFFLs with RiboJ (right). Standard deviations from triplicate experiments are indicated in the shaded areas. **b** Normalized and averaged distribution widths per category are plotted for both the calibration step and database fits. The distribution widths are determined for each parameter separately as the ratio between the average of the five highest and lowest values in the distribution, after which they are averaged per category and normalized to the distributions for the individual step experiments. The four parameter categories are transcription (*K*_TX_), translation (*K*_TL_), regulation (*K*_reg_) and degradation (*K*_deg_) as specified in Fig. 2b. For all separate parameter distributions see Supplementary Fig. 13.

Using the step calibration experiments, we obtained a set of initial parameter estimates. With these, we designed an optimized inflow pattern (Fig. 3a) (i.e. a pattern corresponding to the minimized determinant of the Fischer information matrix for which our model suggest that the information density will be highest and covariance in the parameter values lowest) for the IFFLs. Before performing the optimized flow experiments, we first wanted to validate our workflow with an in silico identifiability analysis. For this, we used the initial parameter estimates and simulated different CFPS experiments (batch, step flow and optimized flow) for all six IFFLs. We then calculated the pairwise collinearity index (metric of covariance) between all parameters for the TetR-IFFL network by applying a single value decomposition to the sensitivity vectors, as described by Gábor et al.^33^. For a graphically easier representation, we lumped all parameters in the four categories (*K*_TX_, *K*_TL_, *K*_reg_, *K*_deg_ as indicated in Fig. 2b) and plotted the mean and normalized collinearity index in Fig. 2c. For a series of five in silico batch experiments, sampling the TetR repressor construct DNA concentration from 0 to 1 nM, we identified high levels of covariance between the parameters (Fig. 2c, first panel and Supplementary Fig. 14). The covariance decreased when going from batch to a step flow experiment (Fig. 2c, second panel and Supplementary Fig. 15) and even further for the optimized flow experiment (Fig. 2c, third panel and Supplementary Fig. 15). Finally, collecting all simulated flow experiments, both step and optimized, for all IFFLs into a database and calculating the collinearity indices for the parameters using this database as a whole instead of the individual simulations resulted in the least covariance between all parameters and thus provided maximal identifiability of the parameters (Fig. 2c, last panel and Supplementary Fig. 16).

The reduction in collinearity index obtained when incorporating the full database can be attributed to the inclusion of parts and parameters that are both shared and non-shared between the different networks. For example, for the *K*_TL_ parameters (highlighted collinearities in Fig. 2c), the observed variance between experiments of the same network, with and without RiboJ, can solely be attributed to the difference in *K*_TL_ rates thus resulting in less covariance. This indicates that shared parts between experiments become a point off information as the difference in expression rates can solely be attributed to non-shared parts. Taken together, the in silico experiments indicate that our workflow indeed results in less covariance and thus improved identifiability of the parameters.

### Extracting parameter estimates

We execute the optimized inflow pattern (Fig. 3a), determined using the initial parameter estimates, in microfluidic chemostats, using unique stock concentrations (*K*_DNA(stock)_) for the repressor in each IFFL based on the step calibration experiments. Experiments were performed in triplicate for each IFFL (both with and without RiboJ sequence). Furthermore, an optimized pulse experiment for σ19 activation was performed (Fig. 3a and Supplementary Figs. 17–19). In this work, we applied the same optimized inflow pattern to each IFFL variant. The parameter estimates were subsequently obtained by fitting the model to the collective of all flow data in the database and from fitting the model to the data of the individual step experiments (Supplementary Fig. 20 and Supplementary Data 1) (fitting 7 models to 13 experiments with time-dependent inputs is a non-trivial problem and discussed in Supplementary Information 5).

To showcase narrowing of the parameter distributions we calculated the distribution width, as the ratio between the average of the five highest and lowest values of each parameter distribution, for both the individual step fits and the database fits. We assume that all parameter values in each distribution are reasonable descriptions of the data. We then lumped all parameters in the 4 parameter categories (*K*_TX_, *K*_TL_, *K*_reg_ and *K*_deg_), averaged the distribution widths within each category and normalized them to the individual step fit (Fig. 3b). The improvement (i.e. a decreased distribution width), especially in the regulation parameters *K*_reg_, can be attributed to two factors. First, the responsiveness of the deGFP signal to changes in the DNA concentration is indicative of the repressors’ strength, since build-up of repressor takes time. So, the continuously changing DNA concentrations in the optimized flow experiments improve estimation of the repressors’ *k*_d_. Second, as the parameters between experiments for *K*_TX_ are shared, the strength of the repressor and the *K*_TL_ of its corresponding mRNA are the sole contributors to the observed variance between experiments using different repressors. Moreover, since each network utilizes a repressor construct both including and excluding a RiboJ sequence, the variance observed between these experiments can solely be attributed to *K*_TL_ leaving a smaller range over which *K*_TX_, *K*_reg_, *K*_TL_ and *K*_deg_ can compensate for each other within the set biophysical constraints. Summarized, we see significant narrowing of the parameter distributions for all but one parameter class (*K*_deg_). Therefore, we believe the newly constrained parameter space is internally consistent and small enough to test in a new context.

### Testing the parameter estimates in new networks

**Fig. 4:**
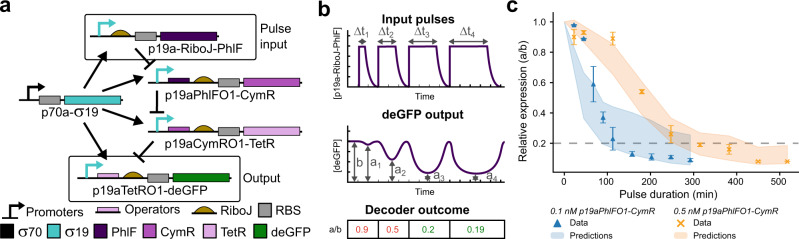
Predicting the behaviour of a pulse decoder. **a** schematic of the pulse decoder network. **b** Schematic overview of the experimental procedure. A longer duration pulse of p19a-RiboJ-PhlF (Δ*t*) results in a lower relative expression (a/b). **c** Data plotted as relative concentration (a/b) against pulse duration for two system configurations (0.1 nM in blue and 0.5 nM p19aPhlFO1-CymR in orange). Error bars indicate the mean and standard deviation over two measurements. The standard deviation of the mean for the first quantile of highest scoring simulations (predictions) is plotted as shaded areas in corresponding colours.

**Fig. 5:**
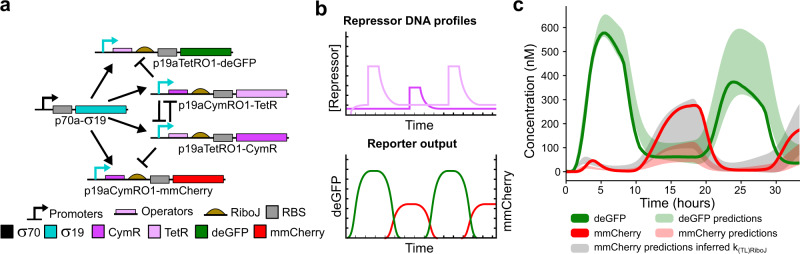
Predicting the behaviour of a bistable switch. **a** Schematic of the bistable switch network. **b** Schematic overview of the experimental procedure for the triple switch experiment. The system starts in the CymR state resulting in deGFP expression. Short increases in either p19aCymRO1-TetR or p19aTetRO1-CymR concentration switch the system to the TetR or CymR state, respectively. **c** Triple switch experiment; experimental data (mean of three measurements) plotted as a solid line. The standard deviation of the mean for the first quantile of highest scoring simulations (predictions) is plotted as shaded areas in corresponding colours. A prediction for the mmCherry signal using the same parameter sets but with an inferred *k*_(TL)RiboJ_ is shown in grey.

In the test phase, we assess if our workflow brings us closer to forward engineering of genetic networks. To do this, we reused building blocks and constructed two circuits, a pulse decoder (Fig. 4) and a bistable switch (Fig. 5), and test if the optimized parameters and model have predictive value. We applied a time-dependent input to both circuits by changing the concentrations of the DNA constructs and predicted the response of both networks.

The pulse decoder is a cascade network driven by σ19 as the activator, three consecutive repressors, and a reporter construct (Fig. 4a). We initiate this network without the PhlF construct. In this state, CymR repressor inhibits the TetR construct and deGFP is produced. We subsequently add the PhlF construct for different lengths of time Δ*t* (defined as the time during which the PhlF construct is actively added), such that when PhlF is present in excess, the resulting decline in CymR production and subsequent activation of TetR production should shut down the production of deGFP. The duration of p19a-RiboJ-PhlF addition determines the drop in relative yield, stabilizing below 20% after a certain threshold duration has been reached (Fig. 4b).

To experimentally validate the pulse decoder, we performed a series of experiments with 9 different pulse durations (Δ*t*) of 5 nM p19a-PhlF for two concentrations of p19aPhlFO1-CymR, 0.1 and 0.5 nM, and created a pulse-response curve (Fig. 4c and Supplementary Fig. 21). For these experiments, we plot the relative drop in expression (a/b in Fig. 4b) versus the pulse duration and as expected, we observed that a longer pulse duration resulted in a stronger reduction of deGFP expression until it stabilizes below 20% (Fig. 4c). The pulse decoder is thus capable of decoding the duration of a pulse, repressing expression to below 20% after a specific duration has been reached. A lower concentration of p19aPhlFO1-CymR (0.1 nM vs. 0.5 nM) resulted in a shift of the entire pulse-response curve to the left as less CymR is present in the system and less PhlF is needed to shut down deGFP expression.

To test if the model with the newly estimated parameters can predict a functional regime, we performed the same set of experiments in silico and scored the simulations against the experiments (all simulated pulse-response curves can be found in Supplementary Data 2 and the used parameter sets in Supplementary Data 1). The shaded area in Fig. 4c represents the standard deviation from the mean of the model simulations. For the 0.1 nM p19aPhlFO1-CymR data series, the model predicts a slightly faster shut down of the CymR production than is observed experimentally, while for 0.5 nM p19aPhlFO1-CymR, the model matches the data series.

First, these results highlight that we are able to obtain both qualitative and quantitatively accurate predictions for a reconfigured network with a perturbed state space. Second, the observed deviation for 0.1 nM p19aPhlFO1-CymR was not unexpected. Microfluidic chemostats do not operate based on continuous flow. A single refresh cycle, which takes 22.5 min, includes a loading step where individual components are added sequentially (lysate, buffer, DNA and MQ). This means that p19a-RiboJ-PhlF is not present in the reactor at the start of the refresh cycle, something that is not accounted for in the model (which assumes immediate PhlF production).

Next, we tested if we would be able to predict the behaviour of a more complex and non-linear system, a bistable switch (Fig. 5). The bistable switch is controlled by the σ19 activator, which drives the expression of two repressors, CymR and TetR and two reporter proteins, deGFP and mmCherry. The reporter constructs are regulated by TetR and CymR, respectively (Fig. 5a). The bistable switch can be in two distinctive states where either one of the two repressors is being expressed. When the system is in the CymR state, CymR is produced continuously and the production of TetR is shut down. When we pulse in p19aCymRO1-TetR, TetR expression is increased and the system switches to the TetR state. The system will subsequently remain in the TetR state until switched back (Fig. 5b).

We chose pulse intensities of 2 nM for p19aTetRO1-CymR and 5 nM for p19aCymRO1-TetR to switch the system (an excess to ensure we can switch the system back and forth, see Supplementary Fig. 22). Figure 5c shows the time-resolved expression traces of both deGFP and mmCherry. We initiated the system in the CymR state by first adding only the p19aTetRO1-CymR construct at 0.2 nM into the reactor. After some time, we added the p19aCymRO1-TetR construct at 0.8 nM, which kept the system in the CymR state. This inhibited the transcription of mmCherry, whereas deGFP was freely expressed. Next, we pulsed an excess of p19aCymRO1-TetR in the reactor, CymR was repressed and the system switched. The system stayed in the TetR state until an excess of p19aTetRO1-CymR was pulsed into the reactor to switch the system back to the CymR state, after which the system was switched once more towards the TetR state.

Similar to the pulse decoder, we simulated the experiment using the estimated parameter sets and plotted the standard deviation from the mean as shaded areas around the actual data. Gratifyingly, predictions matched experimental data rather closely in the case for deGFP. This indicates that our method is able to quantitatively predict properties of more complex networks (all simulated time traces can be found in Supplementary Data 3 and the used parameter sets in Supplementary Data 1). For mmCherry the predicted yields were consistently lower than the experimental yield. This is not surprising, as we did not have any prior data on mmCherry expression with RiboJ and translation rates without RiboJ are generally weaker (Supplementary Fig. 23).

As we have data on translation rates of genes with and without the RiboJ element (Supplementary Fig. 23), we decided to extrapolate these data to parts that did not contain the RiboJ element. We inferred a multiplication factor for the addition of the RiboJ element by taking the median increase for the three repressors upon addition of the RiboJ element. An additional round of predictions, where the mmCherry translation rates are multiplied by this inferred factor, resulted in an improved prediction of the mmCherry signal (Fig. 5c). These results show that information of new combinations of building blocks can be inferred from available data in the database, which will be of use in the formation of a library of genetic elements for forward engineering of cell-free genetic networks.

Note, for a system to be bistable, the *K*_reg_ parameters need to be finely balanced, thus not all parameter sets predicted bistability for our experimental conditions, a small subset of the parameters did not show any, another subset favoured a single state, switching once. Upon closer inspection we found that successful parameter sets shared lowered values for the Hill coefficients of both repressors (between 1 and 2) (Supplementary Fig. 25), indicating that this network topology improves the estimation of *K*_reg_*.*

### Updating the database

In our workflow, we showed that treating all experiments, from different circuit variants, as a collective instead of as separate experiments improves parameter estimates. We explore this in supplementary information 7 and Supplementary Fig. 24, where we compare the predictive power of the model when we fit subsets of the database. Specifically, we fit the step experiments independently, then together, and finally the pulse experiments together. The figure shows a progressive improvement in the predictive power of the model. With the step experiments, we gain initial information about the bounds of the parameter values and the behaviour of independent genes. With the pulse sequence we screen a large part of the space of potential construct concentration combinations and the strength of those interactions at each point, resulting in more refined estimates (this is corroborated by the identifiability analysis in Supplementary Fig. 15 and Fig. 2c, which shows, for example, that the Hill coefficients are less collinear with other parameters in the model for a pulse experiment).

**Fig. 6:**
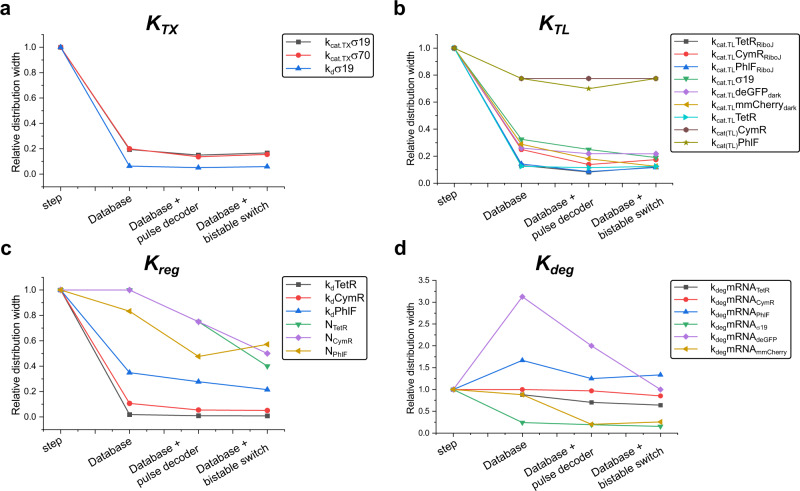
Relative distribution widths. Relative distribution width, relative to the width after step calibration fit, for all parameters grouped per category: **a** transcription parameters (*K*_TX_), **b** translation parameters (*K*_TL_), **c** regulation parameters (*K*_reg_) and **d** degradation parameters (*K*_deg_).

Following this line of reasoning, inclusion of the pulse decoder and bistable switch experiments in the estimation process could result in a further improvement in the parameter estimates. To show this, we took the parameter sets used for the predictions shown in Figs. 4 and 5 and recreated the distributions (Table 1, Supplementary Fig. 25 and Supplementary Data 1). Based on these distributions, we plotted the relative width of these distributions (again as the ratio between the average of the five highest and lowest parameter values and normalized to the calibration step experiments), for all fitting rounds (Fig. 6). Figure 6 highlights that the distribution width of a parameter shrinks in proportion with the complexity of the dataset and that this complexity can be derived from perturbations in both the state space with OED but also the networks space. For example, the parameter space for the Hill coefficients shows to be more constrained when network topologies are used where Hill coefficients play a more prominent role, like the bistable switch. Another striking result is the remarkable lower distribution width for the Hill coefficient of PhlF in the case of the bistable switch experiments. PhlF is not used in this network, but as all parameters are combined and linked in the database, constraining the Hill coefficients of CymR and TetR results in indirectly constraining the Hill coefficient of PhlF. This indicates that combining all experiments for different network configurations and their parameters in a single database can greatly improve characterization of all genetic building blocks in the library. Finally, it demonstrates that a sufficiently complex dataset does map onto genetic building blocks which leads to modularity and predictability.

**Table 1:** Parameter values and distributions.

	Step	Database	Database + pulse decoder	Database + bistable switch
*K*_TX_				
k_cat.TX_σ19 (nM min^−1^)	1.1 (0.1– 4)	1.5 (0.45–3.5)	1.7 (0.5–3)	1.9 (0.48–3.2)
k_cat.TX_σ70 (nM min^−1^)	0.7 (0.1–4)	0.5 (0.2–1.6)	0.7 (0.25–1.37)	0.5 (0.25–1.56)
K_d_σ19 (nM)	636.3 (9–1250)	627.4 (143–1250)	627.4 (168–1188)	794.4 (151–1245)
*K*_TL_				
k_cat.TL_TetR_RiboJ_ (nM min^−1^)	1.6 (0.1–4)	2.8 (0.76–4)	2.5 (1.2–4)	2.0 (0.83–4)
k_cat.TL_CymR_RiboJ_ (nM min^−1^)	1.5 (0.1–4)	1.6 (0.4–4)	1.2 (0.65–3.6)	1.4 (0.5–3.5)
k_cat.TL_PhlF_RiboJ_ (nM min^−1^)	0.6 (0.1–4)	2.3 (0.7–4)	2.2 (1.1–3.8)	2.4 (0.75–3.5)
k_cat.TL_σ19 (nM min^−1^)	0.7 (0.1–4)	0.2 (0.1–1.3)	0.2 (0.1–1)	0.2 (0.1–0.76)
k_cat.TL_deGFP_RiboJ_ (nM min^−1^)	1.8 (0.125–4)	1.0 (0.3–2.5)	0.7 (0.3–2.1)	1.3 (0.33–2.3)
k_cat.TL_mmCherry_RiboJ_ (nM min^−1^)	–	0.5 (0.13–1.5)	0.4 (0.18–1.3)	0.5 (0.17–0.85)
*K*_REG_				
k_d_TetR (nM)	200.1 (1–1250)	27.0 (4–93)	26.5 (7–81)	22.3 (5–53)
k_d_CymR (nM)	207.8 (1–1250)	6.4 (0.6–80)	4.6 (0.8–55)	5.7 (0.57–36)
k_d_PhlF (nM)	3.1 (39–1250)	486.2 (110–1230)	689.5 (133–1183)	545.4 (157–1083)
N_TetR_	2.7 (1–4)	1.5 (1–4)	1.3 (1–3)	1.2 (1–1.6)
N_CymR_	2.7 (1–4)	1.9 (1–4)	2.0 (1–4)	1.4 (1–2)
N_PHLF_	3.1 (1–4)	3.3 (1.2–4)	3.5 (2.1–4)	3.9 (1.75–4)
*K*_DEG_				
k_deg_mRNA-TetR (nM min^−1^)	0.07 (0.008–0.125)	0.04 (0.008–0.11)	0.05 (0.01–0.11)	0.04 (0.01–0.1)
k_deg_mRNA-CymR (nM min^−1^)	0.08 (0.008–0.125)	0.04 (0.008–0.125)	0.06 (0.008–1.21)	0.04 (0.009–0.12)
k_deg_mRNA-PhlF (nM min^−1^)	0.09 (0.025–0.125)	0.06 (0.015–0.125)	0.06 (0.02–0.125)	0.06 (0.018–0.12)
k_deg_mRNA-σ19 (nM min^−1^)	0.11 (0.008–0.125)	0.09 (0.033–0.125)	0.09 (0.04–1.2)	0.09 (0.05–0.12)
k_deg_mRNA-deGFP (nM min^−1^)	0.09 (0.025–0.125)	0.05 (0.008–0.125)	0.04 (0.01–0.1)	0.05 (0.02–0.1)
k_deg_mRNA-mmCherry (nM min^−1^)	–	0.07 (0.008–0.11)	0.08 (0.04–0.125)	0.07 (0.03–1.2)

Mean parameter values, with their upper and lower bounds in brackets, for the estimates from the calibration step experiments, the database and after we updated the database with either the pulse decoder or bistable switch experiments. Graphical representation of the distributions can be found in Supplementary Fig. 25. Units for the parameters are provided in brackets after the parameters name.

## Discussion

Cell-free synthetic biology has been used as a molecular breadboard for the design and testing of new synthetic genetic networks before they are implemented in vivo^15,23,24,58^. However, the genetic building blocks are usually not modular and need to be recharacterized when reused in different network topologies^32^. Here, we demonstrated that our platform, where we combine microfluidics, OED and database fitting, can tackle the modularity problem and predict functional behaviour of two new networks build using the same parts but in different configurations.

Our software is set-up to include any model and any type of experiment (e.g. combining mRNA measurements in batch with OED flow experiments) or microfluidic experimental platform, for example the DNA brush-based system designed by Swank et al.^26^, as long as the complexity of the model scales with the complexity of the dataset^40^. This presents a number of opportunities for further development of the methodology. Comparing a dataset that both adheres to and breaks a known model assumption will be reflected by a shift in the distribution of the parameters involved and pinpoints where mechanistic detail in the model’s description is lacking (model selection)^59^. However, to characterize the genetic building blocks in a more detailed context, more points of control or observables will be needed to increase the information about newly added parameters. These controls can -in principle- be anything that interacts with the CFPS process: antibiotic agents that modify the active ribosome concentration, anti-sigma factors to target transcription, toehold switches which regulate the repression or activation of gene expression at the translational level, or even macroscopic properties such as temperature^60–62^. The addition of more observables, especially mRNA concentrations, could greatly improve parameter estimation, especially for processes like mRNA degradation^63^. A future study could also focus on the utilization of these controls to include the competition for ribosomes and coreRNAP in the mechanistic description of the CFPS process^36,47,64^. Including knowledge about the concentrations of active RNAP and ribosomes would also be a good starting point to address batch-to-batch variation in lysates, as differences in their concentrations likely play a major role^65^. Moreover, Gyorgy et al. have shown that resource competition can be leveraged in combination with OED to characterize genetic building blocks^50,66^.

The continuous expansion of the database opens the door to new design protocols for networks at scale. Reverse engineering larger networks that exhibit complex behaviour has been aptly demonstrated in silico^67,68^, and methods have been developed to a priori reduce the uncertainty about the boundaries of certain kinetic rates prior to fitting a database of experiments^69^. Moreover, a database enables the design of non-intuitive computer-generated network designs, that exhibit significantly more complex behaviour or are designed with a competing evolutionary trade-off in mind, based on actual kinetic data^70,71^.

In conclusion, we demonstrated a microfluidic platform coupled with a computational OED workflow capable of characterizing genetic building blocks for the modular construction of synthetic gene networks in CFPS systems. With this platform in place, future work will include an increase of the library of well-characterised modular building blocks and forward design of larger and more complex (cell-free) genetic networks.

## Methods

### Description of device and microfluidic setup

Devices are created as described by Van der Linden et al.^44^ and Niederholtmeyer et al.^42^ with some minor adjustments. The width of the reactor channels was increased to 250 µm to increase the channel to reactor volume ratio. Besides, control channel 27 was removed and control channels 1–3 were adjusted to fit the increased width of the reactors (see Supplementary Fig. 26).

The microfluidic setup was assembled as described by Van der Linden et al.^44^ with some minor adjustments. Instead of a temperature-controlled box, a temperature-controlled stage (TC-HP75x65, Bioscience Tools) was used. The set temperature was 32.1 °C, resulting in a temperature of 30 °C in the PDMS device (measured using a temperature probe). The pressure on the pneumatic valves was set to 2 bar. Except for the mixing channels which were set to 1.5 bar. Besides, the air outlet of the solenoid valves controlling the mixing channels was connected to a vacuum pump to speed up deflation of the mixing channels. The pressure on the Fluigent system (Fluigent MFCS-EZ^**TM**^) was set to 50 mbar for all fluid lines (Supplementary Fig. 28).

The microfluidic device is controlled by an altered version of the LabView program created by Van der Linden et al.^44^. The LabView program was altered to communicate with the software of the microscope/camera and to work with constantly changing inflow fractions. Predetermined inflow fractions were read from a text file and converted to reactor specific number of load steps (see Supplementary Information 6). The number of steps used was subsequently saved in an output text file. The input text files enabled us to regulate the inflow of DNA for each refresh cycle and per reactor. The output text files were used to calculate the exact concentration of each DNA construct in each reactor over time (see Supplementary Information 6), which were then used for the analysis of the data.

### Construct design

To minimize the effect of the local genetic context on gene expression, all genes were cloned into the pTXTL vector designed by Noireaux et al.^19^. The p70a-S19 plasmid^20^ was bought from Arbor Biosciences. All other genes were cloned using the in vitro GGA technique as described by Sun et al.^52^. Promoter sequences were taken from the toolbox 2.0 plasmids and operator sites were added downstream of the promoter as described by Zong et al.^53^. This design strategy allowed operators and promoters to be exchanged easily between genes, while preserving functionality. To isolate transcription from translation, the RiboJ sequence was added in between the operator and the ribosomal binding site (RBS)^72^. All constructs contain UTR1, bearing a strong RBS^19^. The UTR1 was directly followed by the start codon for the protein sequence, directly followed by the T500 terminator.

Assembly took place like described by Sun et al.^52^. In short, standardized building blocks for the vector, 5′ and 3′ protective regions, promoters, operators, UTR1, RiboJ, coding region and T500 terminator were PCR amplified to introduce Esp3I restriction sites on both ends. Different building blocks were then mixed in a 3:1, vector:insert, ratio and were assembled into a circular construct by 35 cycles of iterative restriction and ligation. GGA products were either directly amplified to retrieve linear templates using PCR amplification or were first transformed into XL1-Blue cells to amplify the plasmid, followed by linearization by PCR. All sequences were sequence verified by BaseClear B.V.

Vector, 5′ and 3′ protective sequences, promoters, UTR1, T500 terminator, deGFP and mmCherry GGA elements were created from the p70a-deGFP, p19a-deGFP and p70a-mmCherry plasmids from Arbor Biosciences^20^. Operator sequences, RiboJ (ultramers, designed after Zong et al.^53^ and Mutalik et al.^72^) and TetR sequence (gBlock) were ordered from IDT and PCR amplified to create GGA elements. PhlF and CymR sequences were PCR amplified from the Addgene plasmids pRF-PhlF (#49367) and pET21a-CymR (#51165), respectively^73^. An overview of the used sequences can be found in Supplementary Table 1.

### CFPS setup

Lysate and feeding buffer (FB) are produced as described by Sun et al.^2^ with some minor alterations. After pelleting and washing the cells, they were stored at −80 °C before being lysed. Instead of bead beating, a French Press was used to lyse the cells. Besides, the composition of the used S30B buffer was 14 mM Mg-glutamate and 150 mM K-glutamate, instead of 14  and 60 mM, respectively. The AA mixture was prepared as described by Caschera and Noireaux^74^.

The final CFPS reaction mixtures contained ~10 mg/mL lysate, 1x FB (1 mM DTT, 1.5 mM each amino acid, 50 mM HEPES, 1.5 mM ATP and GTP, 0.9 mM CTP and UTP, 0.2 mg/ml tRNA, 0.26 mM CoA, 0.33 mM NAD, 0.75 mM cAMP, 0.068 mM folinic acid, 1 mM spermidine and 30 mM 3-PGA), 8 mM Mg-glutamate, 60 mM K-glutamate, 15 mM Maltose, 2% PEG-8000, 2.67 μM GamS (purified following the protocol from Sun et al.^52^) and 1.33 U/mL inorganic pyrophosphatase (IPP). All FB components were purchased from Sigma Aldrich. IPP was purchased from NEB.

For all CFPS reactions, linear DNA templates were used. Linear DNA templates were PCR amplified from plasmids ensuring a minimum of 250 bp of protective DNA upstream and downstream of the promoter and terminator, respectively^52^.

### Performing flow experiments

Flow experiments were performed as described by Van der Linden et al. with some minor adjustments. Besides the device calibration, which determines the refresh ratio (RR) for each reactor separately, a fluorescence calibration was performed using either 5 μM of eGFP and/or mmCherry, by means of serial dilution in the devices. After both calibrations, the devices were cleaned with a 1% Terg-a-Zyme (Sigma Aldrich) solution and washed thoroughly with MQ.

During setup, either the whole CFPS mastermix (everything except DNA and MQ) or separate lysate (also containing GamS and IPP) and energy solutions (ES) (all other CFPS components except DNA and MQ) are loaded into PTFE tubing (ID = 0.56 mm, OD = 1.07 mm, VWR) and cooled to about 0 °C using a homemade lysate cooler (see Supplementary Fig. 27). All other non-cooled components, DNA and MQ, are also preloaded into PTFE tubing and connected to the device using metal connectors (AISI 304 ID/OD × L = 0.35/0.65 × 8 mm, Unimed S.A.).

For all experiments, the refresh fraction (RF), the percentage of the reactor volume that is replaced per dilution cycle, was set to 40%. The inflow ratios for lysate and ES, DNA constructs and MQ were provided by a text file which specifies the inflow ratio for all components per dilution cycle. During each dilution cycle 70% of the refreshed volume is taken up by lysate and ES the remaining 30% is used for DNA and MQ. The concentration of lysate and ES thus stays the same during the experiment. The inflow ratios are then converted to a specific number of load steps using the reactor specific refresh ratio (determined during the device calibration). The amount of load steps used are saved in an output text file. For each experiment one of the reactors is used as a blank, where no DNA is added, and one is used as a positive control, where only the reporter and activator gene are expressed continuously.

For a detailed description of how the reactors work we refer you to the initial papers from Niederholtmeyer et al.^42^ and Van der Linden et al.^44^. In short, a single cycle in the microfluidic program consists of imaging, flushing, loading and mixing. At first images are taken at predetermined positions in all reactors. During the flushing step all channels, except reactors, are filled with fresh material from one of the inlets. Then a specific amount of the just flushed material, determined by the inflow ratio and RR for each reactor, is pushed in each reactor during the loading step. Loading occurs in steps, meaning that the material is added in set increments. After material from all inlets are flushed and loaded into all reactors, order is usually lysate/FB, then all DNAs and MQ last, the contents of the reactors are mixed until a total cycle time of 22.5 min is reached. Images are then again taken at the start of the next cycle.

### Software

LabView (LabView 2019) was used to control the flow through the microfluidic chemostats. ImageJ was used to control the microscope and to extract grey values from the images. Origin (Origin 2020) was used to extract maturation rates for deGFP and mmCherry. An overview for the software that performs the optimizations can be found in Supplementary Information 1. The package is written in Python 3.8 (python software foundation, Delaware US). A simple string-based model object and stepwise translated to an SBML and AMICI object^75^. AMICI is an (excellent) ODE compilation package to C++, its interfaces with python grants the speeds required for fitting models and provides forward sensitivity options^76^. We break the software up in 3 parts, a demo that converts any manually defined model to an AMICI object and allows users to simulate their models with C++ speeds. An Identifiability and experimental design pipeline which takes the AMICI object(s) which allows users to combine different experiments and check which parameters correlate and covary and a model fit module, code can be found at huckgroup github, https://github.com/huckgroup (OED folder). For more information on OED and parameter estimation see Supplementary Information 1–6. For additional literature, we refer the reader to excellent previous work^33,57,77–80^.

### Reporting summary

Further information on research design is available in the Nature Research Reporting Summary linked to this article.

## Supplementary information

Supplementary Information

Reporting Summary

Description of Additional Supplementary Files

Supplementary Dataset 1

Supplementary Dataset 2

Supplementary Dataset 3

## Data Availability

The parameter sets acquired and used for the predictions can be found in Supplementary Data 1. Other data that support the findings of this study are available from the corresponding author upon reasonable request.

## Code availability

Used code is available from the huckgroup github, https://github.com/huckgroup (OED folder). Citeable archived versions can be found 10.5281/zenodo.6610321.
