# Supplementary material for: A microfluidic optimal experimental design platform for forward design of cell-free genetic networks
Source: Nat Commun. 2022 Jun 24;13:3626. doi: 10.1038/s41467-022-31306-3 (PMC9232554; doi:10.1038/s41467-022-31306-3)
Supplement: Supplementary file 1 — Supplementary Information [file 41467_2022_31306_MOESM1_ESM.pdf]

A microfluidic optimal experimental design platform for forward design of cell-free genetic networks

Bob van Sluijs[1], Roel J. M. Maas[1], Ardjan J. van der Linden[2,3,4], Tom F. A. de Greef[1,2,3,4,5], Wilhelm T. S. Huck[1,*]

[1] Institute for Molecules and Materials, Radboud University, Nijmegen, the Netherlands. [2] Laboratory of Chemical Biology, Department of Biomedical Engineering, Eindhoven University of Technology, Eindhoven, the Netherlands. [3] Institute for Complex Molecular Systems, Eindhoven University of Technology, Eindhoven, the Netherlands. [4] Computational Biology Group, Department of Biomedical Engineering, Eindhoven University of Technology, Eindhoven, the Netherlands. [5] Center for Living Technologies, Eindhoven-Wageningen-Utrecht Alliance, Eindhoven, The Netherlands

These authors contributed equally: Bob van Sluijs, Roel J. M. Maas

* email: w.huck@science.ru.nl

Supplementary information

1. Software

Optimal experimental design (OED) and fitting a set of models to 20+ experiments is computationally expensive. For OED the number of equations that need to be solved scales in line with the number of states and parameters, as each state/parameter combination results in an additional ODE that needs to be solved. We chose an excellent toolbox –AMICI- to compile the ODE's in python to C++ files callable from python. AMICI makes use of an SBML file. To facilitated ease of use we mapped a string-based definition of the model onto the SBML format using other standardized formats as intermediates so people can plug and play. We start by defining a model including:

- Model states
- Observables
- Initial guess for parameter value
- Lower and upper bound for each parameter
- Control parameters

Next, we parse the model into a matrix format and obtain a stoichiometric matrix and a flux vector derived from the reaction equations according to (1).

$$\sum_{i=1}^{\mathrm{N}} \mathbf{R}_{\mathrm{ij}}\mathbf{X}_{\mathrm{i}} \overset{\mathrm{k_j}}{\rightarrow} \sum_{i=1}^{\mathrm{M}} \mathbf{P}_{\mathrm{ij}}\mathbf{X}_{\mathrm{i}} \tag{1}$$

Were $j \, \epsilon [1, N]$. Thus $\mathbf{R}$ and $\mathbf{P}$ are $\mathrm{N} \times \mathrm{M}$ matrices representing the number of products in a reaction, respectively, and k is a vector of N reaction rate. To simulate the chemical reaction network, the reaction scheme above is translated into ODE's applying Michealis Menten and Hill type kinetics. We can translate the system into a system of M ODEs (2):

$$\frac{d\mathrm{X}}{dt} = Sv(\mathrm{X}) \tag{2}$$

Where $\boldsymbol{S} \triangleq (\mathbf{P} - \mathbf{R})^T$ is the stoichiometry of the system and $v(X)$ the kinetic equations in the flux vector. The stoichiometry matrix and flux vector are subsequently used to create an Antimony file (human readable version of SBML) using the Tellurium toolbox. Antimony makes use of reaction equations and fluxes, creating a flux vector and stoichiometric matrix facilitates this translation from equations to antimony.

The antimony file is converted to an SBML file using LibSBML which in turn is compiled to C++ using AMICI. There is an additional option to calculate the forward sensitivities in python using the simpy library. For the installation of AMICI we refer the reader to https://pypi.org/project/amici/.[1] With this we create a *ModelObject* that contains the needed information to solve the system in the model solver. The model solver takes the model object and the experimental conditions and or time dependent inputs of the control parameters and simulates the system. If it cannot find an AMICI object, Tellurium will be used, if that is lacking Scipy's integrate (LSODA) will be used. To simulate time dependent control parameters, we use a start-stop approach, and re-initiate the solver with the sensitivities and state data of the last time point. The solver returns a data object that is subsequently used in both the Identifiability analysis, optimal experimental design and model fit (Supplementary Fig. 1).

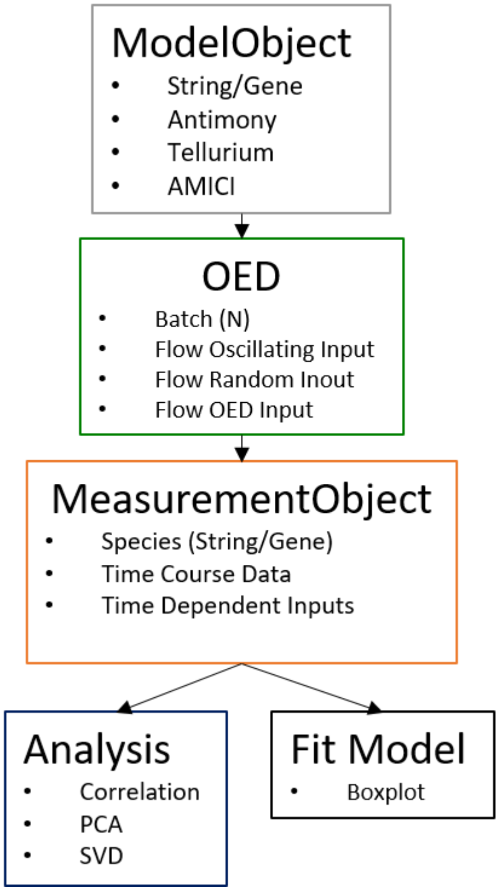

Supplementary Figure 1: Simple overview of python pipeline, we create a model object where we map the different formats (python, antimony etc) to each other. This ODE model can be defined by the user. This model object is passed on to an experimental design module which creates different types of an in silico experiment. This module returns a measurement or set of measurement objects (identical to what would be used for real experimental data, this set of measurements can contain both different models and experimental conditions). This in turn can be passed to an analysis and fitting module, capable of calculating parameter correlations, PCA analysis and SVD analysis to assess the identifiability.

The user can define the model manually in a separate python file (Supplementary Fig. 2). Note that the naming in the models, its states and parameters needs to be consistent, similarly when you define the model make sure that any subtraction or addition inside a fluxterm v in (2) is marked by straight lines '|+|' and |-|, anything between two unmarked signs will be considered a fluxterm.

```
# -*- coding: utf-8 -*-
"""
Created on Wed Mar 23 14:01:40 2022

@author: bob van sluijs
"""
def main():
    models,control = {},{}

    bounds = { '(in)p70_S19':(0.1,3.5),
               '(in)p19_deGFP':(0.1,3.5),
               'KcatP70':(0.1,10),
               'degmRNAS19':(0.01,0.3),
               'KcatP19':(0.1,10),
               'kd_S19':(1,100),
               'degmRNAdeGFP':(0.01,0.3),
               'KcatmRNAS19':(0.1,10),
               'KcatmRNAdeGFP':(0.1,10),
               'kmatdeGFPdark':(0.099,0.1),
               'dil':(0.026,0.043),
               }

    maximum_likelihood = { '(in)p70_S19':1,
                           '(in)p19_deGFP':1,
                           'KcatP70':5,
                           'degmRNAS19':0.025,
                           'KcatP19':5,
                           'kd_S19':100,
                           'degmRNAdeGFP':0.025,
                           'KcatmRNAS19':0.5,
                           'KcatmRNAdeGFP':0.5,
                           'kmatdeGFPdark':0.088,
                           'dil':0.026
                           }

    modelname = 'Toy_Model'
    stringmodel = """
                  + (in)p70_S19 * dil - p70_S19 * dil
                  + (in)p19_deGFP * dil - p19_deGFP * dil
                  + p70_S19 * KcatP70 - dil * mRNAS19 - degmRNAS19 * mRNAS19
                  + p19_deGFP * KcatP19 * ( S19 / kd_S19 ) / ( 1 |+| ( S19 / kd_S19 ) ) - dil * mRNAdeGFP - degmRNAdeGFP * mRNAdeGFP
                  + KcatmRNAS19 * mRNAS19 - dil * S19
                  + KcatmRNAdeGFP * mRNAdeGFP - kmatdeGFPdark * deGFPdark - dil * deGFPdark
                  + kmatdeGFPdark * deGFPdark - dil * deGFP """

    conditions         = []
    states             = ['p70_S19','p19_deGFP','mRNAS19','mRNAdeGFP','S19','deGFPdark','deGFP']
    observables        = ['deGFP']
    control_parameters = ['(in)p70_S19','(in)p19_deGFP','dil']
    models[len(models)]     = ModelObject(stringmodel,states,bounds,ML,observables = observables,name = modelname,control_parameters = control_parameters)
```

Supplementary Figure 2. Toymodel example of 2 genes P70.S19 and P19.deGFP. We define the states, parameters, control parameters and observables, this .py file will need to be called if you want to use this model. Note that addition and subtraction within a flux term is denoted by |+| or |-|. Note that you can simply add another model underneath this toy model to create a set of models.

Next, we import this model and compile it (Supplementary Fig. 3), parsing this file into a Sympy fluxvector and stoichiometric matrix according to equation (1), this facilitates the translation to antimony, which in turn facilitates the translation to SBML and finally AMICI (LibSBML and AMICI have conflicting packages so after the SBML file is created the program is restarted, if there is a mistake e.g. typo, in the model make sure to delete the existing SBML file).[2,3] We subsequently import *GenerateExperiment* capable of creating *in silico* experimental 'data' including the application of different time dependent inputs as per the flow reactor or batch experiments. This module returns a measurement object which can be passed on to different analysis tools, or a parameter fit procedure. Note that 1) these measurements can be combined in a dictionary and still be passed on to the fitting 2) the measurement object does not have to have the same model, they can be complementary with a shared parameter space (e.g. if when we want to fit the 3 feed forward loop experiments simultaneously). The code can be found on the huckgroup GitHub https://github.com/huckgroup/ (folder OED).

```python
"""import the model"""
import ToyModel as model
models,control = model.main()

"""create th SBML model and Amici models, needed because packacges conflict"""
for i in range(len(models)):
    model = models[i]
    model.SBMLconversion()
    model.PytoCompile()

"""import modules for experiment generation and identifiability analysis"""
from Measurements import GenerateExperiments
from IdentifiabilityAnalysisModelSets import IdentifiabilityAnalysis

"""name of folder where identifiability of experiment will be stored"""
name = model.name + 'First Test'

"""create a 'fake' measurement objects in the GenerateExperiment class
(you can do batch, random pulse etc. just check measurement folder)"""
m = GenerateExperiments(model)
measurement_1 = m.return_random_pulse_measurement(store = True,name = name,time = (0,100))
measurement_2 = m.return_random_pulse_measurement(store = True,name = name,time = (0,100))
#...
#...
#...

"""its a dict {0:Measurement object}, lets combine 2  experiments together and
give them to the identifiabilitiy analysis (i.e. it combines them and does the idenfiability analysis as if its 1)"""
dataset = {}

"""build a dataset, Note we only have 1 model but
the dataset can consists of measurements with multiple models"""
dataset[len(database)] = measurement_1
dataset[len(database)] = measurement_2

"""ANALYSE: Collinearity index, correlation and PCA"""
EXP = IdentifiabilityAnalysis(dataset)
EXP.Correlation(name = name)
EXP.PCA(name = name)
EXP.SVD(name = name)

"""FIT: fit the measurements and retrieve parameter estimates"""
 BoxplotOptimization(database,
                     optimization_number = 100,
                     ,generations = 10,
                     ,agents = 10,
                     ,storedata = '[LOCATION]',
                     startsamples = 1000)
```

Supplementary Figure 3. Import the model and compile it to C++, this AMICI compilation is stored on your desktop. If the file already exists it will skip this step. We can use the model to create in silico measurements which can be passed onto the analysis and/or fit procedure.

2. Model

The IVTT process is modeled by the ODE's defined in (2). The individual processes can be broken down into the following equations. First, we model the inflow of the genetic material into the microfluidic reactors according to

$$\frac{d[DNA]}{dt} = (in)\mathrm{DNA} * \mathrm{dil} - [DNA] * \mathrm{dil} \tag{3}$$

Where the concentration of the DNA constructs [DNA] is proportional to its inflow rate and the concentration of genetic material in the stock and the outflow. The concentration flowing into the reactor itself can be calculated according to

$$(\mathrm{in})\mathrm{DNA} = \frac{\mathrm{IF}}{\mathrm{t_{ip}}} * \mathrm{K_{DNA}}(\mathrm{stock}) \tag{4}$$

where IF (the fraction of the replaced reactor volume) divided by t_{IP} (min^{-1}) the time per input, controls how much of the $k_{DNA(stock)}$ (*nM*) stock concentration flows into the reactor. We choose the stock concentration at the start of the experiment and control the inflow fraction k_{IF} and the time per input $t_{IP.}$ during the experiment. Next, we model mRNA in the reactor, we use hill type kinetics as shown in equation (5) with a production term (including regulation) a linear degradation term and an outflow term. The production is proportional to the concentration of DNA in the reactor, the sigma factor that recruits the polymerase and any regulation by –in our case- repressors.

$$\frac{\mathrm{d}[mRNA]}{\mathrm{dt}} = [DNA] * \mathrm{k_{cat.TX}\sigma 19} \frac{\frac{[\sigma 19]}{\mathrm{k_d \sigma 19}}}{1 + \frac{[\sigma 19]}{\mathrm{k_d\sigma 19}} + (\frac{[R]}{\mathrm{k_d R}})^{\mathrm{N_R}}} - \mathrm{k_{deg} mRNA} - \mathrm{dil} * [mRNA] \tag{5}$$

Where [DNA] is the concentration of the genetic material in the reactor according to (3), $[\sigma 19]$ the concentration of $\sigma 19$ activator, $[R]$ the concentration of the repressor present. $k_{\mathrm{cat.TX}}\sigma 19$ is the catalysis rate for mRNA transcription in *nM/min*, $k_d\sigma 19$ the dissociation constant in *nM* for the $\sigma 19$ activator, $k_d R$ the dissociation constant for this repressor, N_R the hill coefficient of this repressor, $k_{\mathrm{deg}} mRNA$ the degredation rate of mRNA in min^{-1} and dil the refresh/flow rate of the lysate and genetic material into and out of the reactor in min^{-1}.

For genes transcribed by the $\sigma 70$ activator the fluxterm is independent of the $\sigma 70$ concentration. We assume that $\sigma 70$ is always present in the lysate and its concentration remains unchanged as it is not expressed by our constructs, the production term V_{TX} for mRNA becomes

$$\mathrm{V_{TX}} = [DNA] * \mathrm{k_{cat.TX}\sigma 70} \frac{1}{1 + (\frac{[R]}{\mathrm{k_d R}})^{\mathrm{N_R}}} \tag{6}$$

Next, we model translation of the mRNA into protein, assuming the translation rate is proportional to the mRNA concentration and resources like ribosomes are not limiting (for our chosen conditions). Nor did we include protein degradation under the assumption that this will occur at a significantly slower rate than its dilution due to flowrate. Thus, we end up with a simple term

$$\frac{d[P]}{dt} = k_{cat.TL}P * [mRNA_p] - dil * [P] \quad (7)$$

Where $k_{cat.TL}P$ is the translation rate in *nM/min* of a specific protein. In our system we have mRNA constructs that both include and exclude a RiboJ sequence for all repressors. The model accounts for this by substituting the $k_{cat.TL}P$ parameter for a different $k_{cat.TL}P_{RiboJ}$ parameter. Finally, for reporter proteins we need to account for the maturation of the fluorophores from their dark state, (P_{dark}), to the matured state (P_{mat}), we do this by including a maturation term in (7).

$$\frac{d[P_{dark}]}{dt} = k_{cat.TL}P_{dark} * [mRNA_p] - dil * [P_{dark}] - k_{mat} * [P_{dark}] \quad (8)$$

Where k_{mat} is a measured maturation rate in *min*$^{-1}$. The concentration of matured fluorophore is then modelled by

$$\frac{d[P_{mat}]}{dt} = k_{mat} * [P_{dark}] - dil * [P_{mat}] \quad (9)$$

Equation 2-9 summarized in a practical example; the IFFL under the control of the *TetR* Repressor (DNA constructs are annotated with the sigma-factor controlling the promoter before the full stop and the protein that is expressed after. An operator site embedded in the promoter is indicated by brackets):

$$\frac{d[\sigma_{70}.\sigma_{19}]}{dt} = (in)\sigma_{70}.\sigma_{19} * dil - [\sigma_{70}.\sigma_{19}] * dil$$

$$\frac{d[\sigma_{19}.TetR]}{dt} = (in)\sigma_{19}.TetR * dil - [\sigma_{19}.TetR] * dil$$

$$\frac{d[\sigma_{19}(TetR).deGFP]}{dt} = (in)\sigma_{19}.deGFP * dil - [\sigma_{19}(TetR).deGFP] * dil$$

$$\frac{d[mRNA_{\sigma_{19}}]}{dt} = [\sigma_{70}.\sigma_{19}] * k_{cat.TX}\sigma 70 - k_{deg}mRNA_{\sigma 19} * [mRNA_{\sigma_{19}}] - dil * [mRNA_{\sigma_{19}}]$$

$$\frac{d[mRNA_{TetR}]}{dt} = [\sigma_{19}.TetR] * k_{cat.TX}\sigma 19 \frac{\frac{[\sigma 19]}{k_d\sigma 19}}{1 + \frac{[\sigma 19]}{k_d\sigma 19}} - k_{deg}mRNA_{TetR} * [mRNA_{TetR}] - dil * [mRNA_{TetR}]$$

$$\frac{d[mRNA_{deGFP}]}{dt} = [\sigma_{19}(TetR).deGFP] * k_{cat.TX}\sigma 19 \frac{\frac{[\sigma 19]}{k_d\sigma 19}}{1 + \frac{[\sigma 19]}{k_d\sigma 19} + (\frac{[TetR]}{k_d TetR})^{N_{TetR}}} - k_{deg}mRNA_{deGFP} * [mRNA_{deGFP}] - dil * [mRNA_{deGFP}]$$

$$\frac{d[\sigma_{19}]}{dt} = k_{cat.TL}\sigma 19 * [mRNA_{\sigma_{19}}] - dil * [\sigma_{19}]$$

$$\frac{d[TetR]}{dt} = k_{cat.TL}TetR * [mRNA_{TetR}] - dil * [TetR]$$

$$\frac{d[deGFP(dark)]}{dt} = k_{cat.TL}deGFP_{dark} * [mRNA_{deGFP(dark)}] - dil * [deGFP(dark)] - k_{mat}deGFP * [deGFP(dark)]$$

$$\frac{d[deGFP]}{dt} = k_{mat}deGFP * [deGFP(dark)] - dil * [deGFP]$$

States

- **DNA**
  - $\sigma_{70}.\sigma_{19},$
  - $\sigma_{19}.TetR$
  - $\sigma_{19}(TetR).deGFP$
- **mRNA**
  - $mRNA_{\sigma_{19}}$
  - $mRNA_{TetR}$
  - $mRNA_{deGFP}$
- **Protein**
  - σ_{19}
  - $TetR$
  - $deGFP(dark)$
  - $deGFP$

Control parameters

- $\mathbf{(in)\sigma_{70}.\sigma_{19}}$
- $\mathbf{(in)\sigma_{19}.TetR}$
- $\mathbf{(in)\sigma_{19}.deGFP}$
- $\mathbf{dil}$

Parameters

- **Transcription ($\mathbf{K_{TX}}$)**
  - $\mathbf{k_{cat.TX}\sigma 70}$
  - $\mathbf{k_{cat.TX}\sigma 19}$
  - $\mathbf{k_{d}\sigma 19}$
- **Translation ($\mathbf{K_{TL}}$)**
  - $\mathbf{k_{cat.TL}\sigma 19}$
  - $\mathbf{k_{cat.TL}TetR}$
  - $\mathbf{k_{cat.TL}deGFP_{dark}}$
- **Regulation ($\mathbf{K_{reg}}$)**
  - $\mathbf{k_{d}TetR}$
  - $\mathbf{N_{TetR}}$
- **Degredation ($\mathbf{K_{deg}}$)**
  - $\mathbf{k_{deg}mRNA_{\sigma 19}}$
  - $\mathbf{k_{deg}mRNA_{TetR}}$
  - $\mathbf{k_{deg}mRNA_{deGFP(dark)}}$

Measured parameters

- **Maturation**
  - $\mathbf{k_{mat}deGFP}$

3. Fisher Information and Optimal Experimental Design

Most optimal design problems revolve the fisher information matrix (FIM). The fisher information of a parameter -geometrically- is the curvature around its maximum likelihood estimate for a given dataset. Noisy data, parameter insensitivities or covariance with other parameters equate to a flat curvature representing less information.[4] Since noisy data and parameter insensitivity are intrinsic to the experimental set-up and the observables, reducing the covariance between parameters by optimizing a sequence of control inputs is crucial. To score the amount of information present in the dataset we make use of the D-optimal criterion of the Fisher information. By taking the determinant of the covariance matrix of parameters we calculate the volume of the parameters space wherein an optimal convergence solution for a given dataset can be found. For ODE's the FIM itself can be computed from the parameter sensitivities using the following steps: first, differentiating the model states with respect to the parameters according to:

$$\frac{d}{dt}\frac{\partial x}{\partial p_i} = \frac{\partial f(x,u,p)}{\partial x}\frac{\partial x}{\partial p_i} + \frac{\partial f(x,u,p)}{\partial p_i} \quad for\ i = 1, \dots, N_p \tag{10}$$

Where $\frac{\partial f(x,u,p)}{\partial x}$ is the Jacobian, $\frac{\partial x}{\partial p_i}$ the sensitivity coefficients and $\frac{\partial f(x,u,p)}{\partial p_i}$ the parameter sensitivities. Note, we end up with an additional N_p * N_x number of sensitivity ODE's. We subsequently construct a sensitivity matrix from the solution according to:

$$\boldsymbol{S} = \begin{bmatrix} \frac{\partial y_{j1}}{\partial p_1}(t_1) & \cdots & \frac{\partial y_{j1}}{\partial p_{N_p}}(t_1) \\ \vdots & & \vdots \\ \frac{\partial y_{j1}}{\partial p_1}(t_{N_t}) & \cdots & \frac{\partial y_{j1}}{\partial p_{N_p}}(t_{N_t}) \\ \frac{\partial y_{j2}}{\partial p_1}(t_1) & \cdots & \frac{\partial y_{j2}}{\partial p_{N_p}}(t_1) \\ \vdots & & \vdots \\ \frac{\partial y_{j2}}{\partial p_1}(t_{N_t}) & \cdots & \frac{\partial y_{j2}}{\partial p_{N_p}}(t_{N_t}) \end{bmatrix}$$

Where y is are the subset of observable states x, p the parameter and t the time point of the observation up to t_{N_t} and j the index corresponding to individual experiments. This matrix can be stacked with additional observables and a number of separate experiments. With this, The FIM is subsequently computed according to

$$\mathbf{F} = \mathbf{S}^{\mathrm{T}}\mathbf{S} \tag{11}$$

We only include the unknown parameters to compute the Fisher information matrix i.e. we exclude the measured constants (fluorophore maturation rates) and control parameters (dilution rate and refresh fraction of genetic material). To subsequently select the most informative input pattern during the optimization we use the d-optimality criterion taking the $\max_D(\mathrm{F})$. A good example of this is given in Sinkoe et al..[5] Note, that the structure of the fisher information matrix highlights the need for reasonable initial estimates of the parameters (i.e. a calibration step experiment). The Fisher information matrix is built using the derivatives of the parameters with respect to the observables, the value of those parameter is therefore important. The forward sensitivity maps onto the impact a parameter has at a specific time on the rate of change of the observable. Thus, if your forward sensitivities do not map onto the experimentally observed rates of change, we no longer approximate the potential parameter cloud that can describe the dataset.

4. Identifiability Analysis

For the identifiability performed of specific parameters, we perform a different operation on the Fisher information matrix **F** by making use of the methods described by Gabor *et al.*.[6] We perform a single value decomposition to the FIM in (11) according to $\frac{\mathbf{F}}{||\mathbf{F}||}$, the collinearity index CI is subsequently calculated according to $\frac{1}{\sqrt{\min(\lambda)}}$ where $\min(\lambda)$ is the smallest. With this we can compare and contrast the information in different sets of experiments about specific parameters, in rough terms, which experiment could be considered a valuable addition to the database and which experiment is best suited to identify a specific parameter (instead of the whole, as is done with the D criterion). We make use of this analysis to get an indication about the degree to which parameters covary (and target parameters that can be chosen to decouple the covariances). In addition to the collinearity index, we also calculate the correlations between parameter sensitivities, in contrast to the CI which maps onto the length of the diagonal of the parameter cloud that can describe the data, the correlations give the direction of this diagonal. If a correlation between forward sensitivities is positive it means that an increase or a decrease of the parameter values affects the observed output in the same manner. If the correlation is negative, they have opposite effects on the observation.

We demonstrate this identifiability analysis using a toy model of the following genes:

Constructs in Model: $\sigma_{70}.\sigma_{19} + \sigma_{19}.\mathrm{deGFP(dark)}$

In this example we only make pairwise comparisons between parameters. We start with a simple batch experiment and calculate the correlations between the forward sensitivities of the parameters to the *deGFP* observation. In supplementary figure 4 we see a positive correlation between the transcription and translation of the activator σ_{19} and the translation of *deGFP* but a negative correlation (i.e. opposite effects) between that same *deGFP* translation rate and the dissociation constant of σ_{19} or the degradation rates of both *deGFP* or σ_{19} mRNA. These correlations do not change as we move from batch to flow.

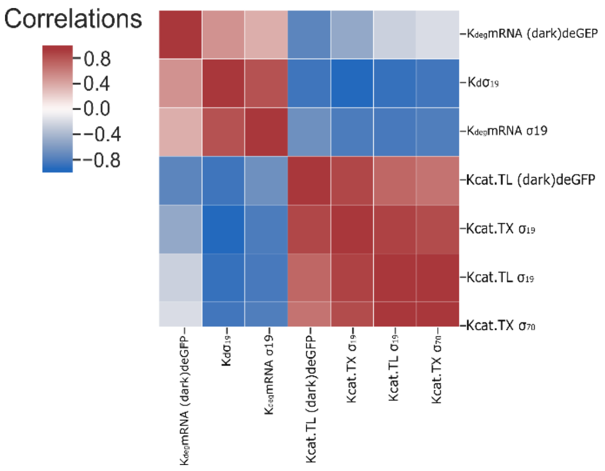

Supplementary Figure 4. The figure shows the correlations between the parameter sensitivities for this network.

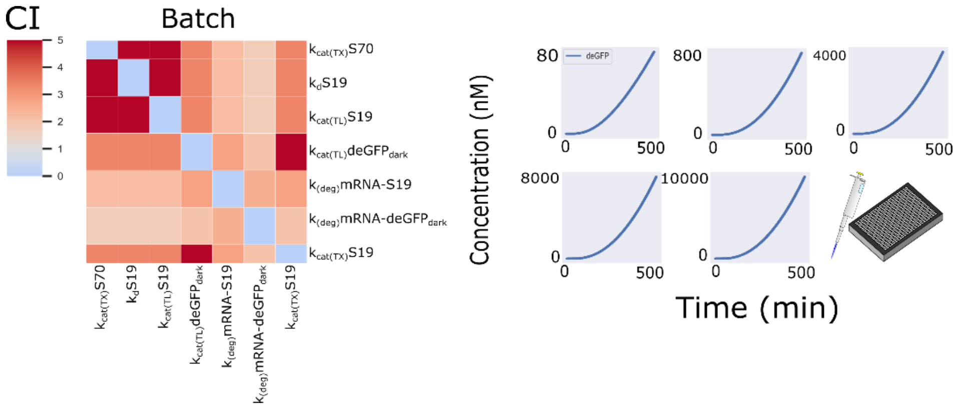

Supplementary Figure 5: The figure shows the smallest eigenvalue following an SVD on the Fisher information matrix created for each parameter pair derived from a simulated batch experiments (with $\sigma_{70}.\sigma_{19}$ concentration range of 0.01, 0.1, 0.2, 0.5, 1 nM and 10 nM of $\sigma_{19}.deGFP$(dark)).

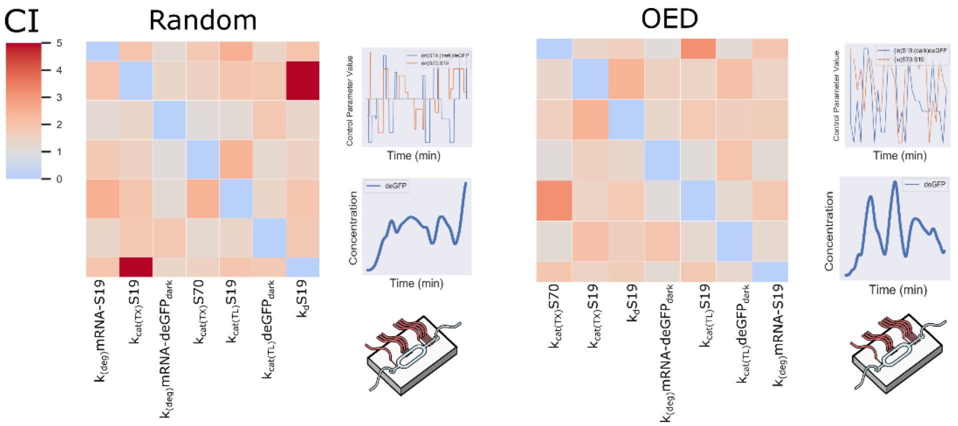

Supplementary Figure 6: The figure shows the smallest eigenvalue or collinearity index following an SVD on the Fisher information matrix created for each parameter pair. In the heatmap on the left the values are based on an experiment where the genetic constructs were flowed in at random (shown is the best set out of 100 random sets of control inputs). The heatmap on the right utilized the OED algorithm to find the best set of control inputs, OED algorithm makes use of the same structure as the algorithm for the model fit.

However, the collinearity index does change as we increase the complexity of our experiment. The difference between batch and flow in Supplementary Figure 5 and 6 highlights the utility of microfluidic flow devices. Besides, the difference between a random pulse set versus an optimized pulse set in Supplementary Figure 6 highlights the utility of OED as the experiment is tuned to maximize the information about the parameters. The overall idea is that we can create an *in silico* database of experiments, test the identifiability and if we find a promising set of experiments we can fit these to quantify the effect this set of experiments has on individual parameters distributions (all before any actual experiment is performed) (Supplementary Fig. 7).

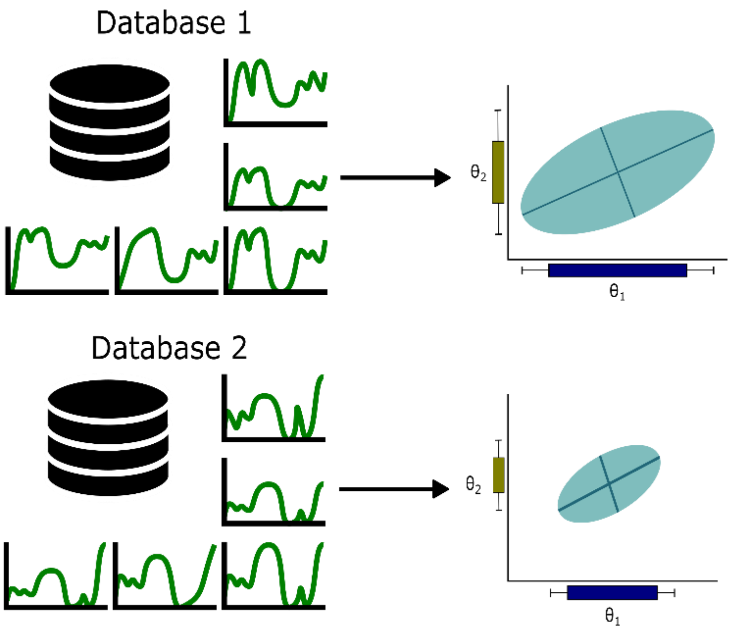

Supplementary Figure 7: The figure is an abstraction of the utility of the identifiability analysis, where the user can use this pipeline to create different databases and quantify the knowledge about the parameters within each database without doing an experiment. By doing this we can screen which combination of parts, addition of control inputs and/or observables best reduces the covariance between unknown parameters. Assemble parts > create in silico measurement > Fit simulated data.

5. Model Fit and Prediction of New Functional Behaviour in Test Networks

The experimental data we obtain is complex, not including the calibration step experiments, the model needs to fit *7* experiments for 3 IFFLs with wavelike time dependent inputs, this is a non-trivial optimization problem. To fit the data, we use our own flexible agent-based optimization algorithm and minimize the difference between the fast fourier transform of the experimental data and the simulation according to

$$S = \sum_{k=1}^{i}\sum_{l=1}^{o} \frac{\sum(\mathrm{FFT}(\mathrm{N}_{\mathrm{k,l}}) - \mathrm{FFT}(\mathrm{M}_{\mathrm{k,l}}))^2}{\left(\frac{\sum_{\mathrm{j=1}}^{\mathrm{n}} \mathrm{M}_{\mathrm{k,l}}}{\mathrm{n}}\right)} \tag{12}$$

Where N is the experiment and M the corresponding simulation time course data vector, *l* the index of the experiment, *o* the observable and *n* the timepoint. The fitting procedure consists of 3 steps:

- Monte Carlo Samling (Latin hypercube), discard unfit ~80%
- Initial fit of OED experiments with MC samples as starting point
- Refinement of initial fits using forward sensitivities (optimization)

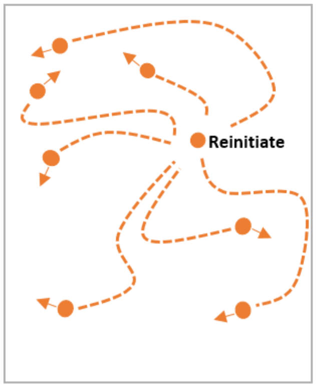

Algorithm

```
Require: Parameter optimization
1.   Define model, parameter boundaries, observables and control parameters
2.   Initiate Fitness landscape and agents.
3.   Iteration = 0
     While convergence < threshold:
          For i = 0 to N_agents Do
               mutate agent[i]
                    if agent[i].score > agent[i-1].score:
                         accept movement of agent
                         if all agent.accepted[-3] == False and agent.current > gradient.current :
                              update gradient
                         if agent.score / fittest agent.score > n and iteration > m:
                              re-initialize next to fittest agent
          Iteration += 1
```

Supplementary Figure 8: The figure is an abstraction and overview of the fit algorithm.

The parameter space for a single parameter is created by building a (uniform)logarithmically spaced vector V with n the size of the vector (100). The individual parameters $V_i = 10^{\theta_i}$ where θ_i is sampled from a uniformly distributed vector $\boldsymbol{\theta}$ defined by $\boldsymbol{\theta} = \{\mathrm{r} : \mathrm{r}\ \exists \mathrm{i} \in \mathbb{N} \text{ such that } \mathrm{r} = \log_{10}(\alpha) + \frac{\log_{10}(\beta)}{\mathrm{n}} i, \text{and } \mathrm{r} \in [\log_{10}(\alpha), \log_{10}(\beta))\}\}$ where α and β, the respective upper and lower bound of a parameter. This means that agents move across the fitness landscape by mutating the index (up or down) within the parameter vector (agents communicate/recombine with each other by passing on these indices). The agents coalesce every 20 turns around the –at this point- fittest agent in the set. An agent can be given a random mutation (any value between the boundaries of the parameters set) or a local mutation deviating only a single index. To apply a directed mutation, we calculate the forward sensitivity with respect to the likelihood and where we end up with the following equation.

$$\frac{dL(Y:p)}{dp} = \sum_{i=1}^{n}\big(y(t_i) - x(t_i, p)\big) \; * \; x_p(t_i, p) \tag{13}$$

Where y is the experimental data, x the simulated equivalent, and x_p the vector with parameter sensitivities from equation (10), thus if we subtract the model simulation from the original experimental data and multiply this with the forward sensitivities, we obtain the direction of mutation. Because calculating the forward sensitivities is computationally expensive, we only update them if 3 successive mutations are not successful (Supplementary Fig. 8).

To obtain the parameter sets shown in the paper, we sampled 500.000 parameter sets using Monte Carlo sampling and simulated the flow experiments to hone in on the most promising regions of the parameter space. We subsequently used the fittest 5000 to initiate the optimization algorithm. We optimized and refined 10.000 parameter sets. These sets were subsequently checked to see if they fufilled the alloted mRNA constraints (the concentration of mRNA at steady state, without repressor, needs to be at minimum, twice the concentration of the added DNA and at maximum 500 times). We subsequently selected all parameter sets that had fitscores that deveate 42.5% from the best fit score assuming that all these paramater sets are equally likely (in line with an outlier for the largest observed experimental variation). Finally, we tested these parameters sets on the step calibration experiments. This left us with ~2500 sets (Supplementary Fig. 9) and again filter scores deveate 42.5% from the best fittest score. This left us with ~600 parameter sets. These sets we subsequently used to predict the networks behavior of the test models. Similarly these sets were used to contrast the ratiometric difference between the upper and lower bound of the parameters. We added a csv file with the parameter values in the datafolder (Supplementary Data 1).

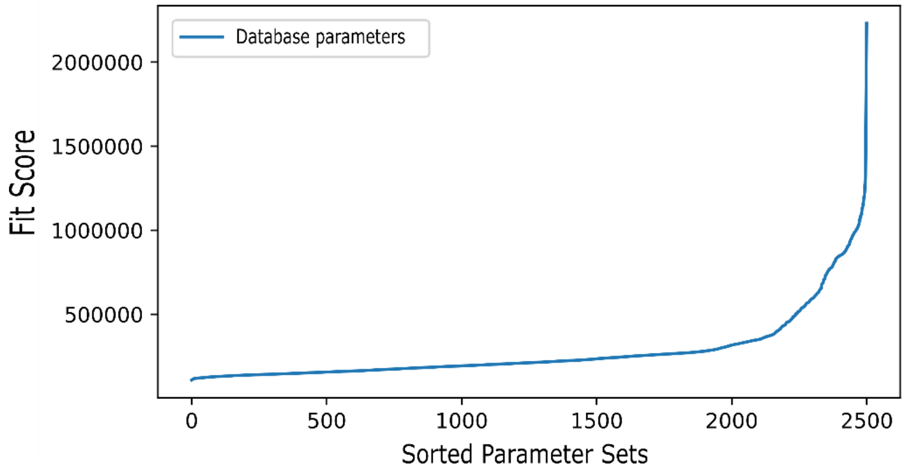

Supplementary Figure 9: The figure shows the convergence of the fitting procedure. The curve is obtained after scoring the fitness of the parameter fitted on the pulse experiment data and simulating all the calibration step experiments. Note, the tail end shows sets, that are deemed fit for the pulse experiments but clearly cannot predict the step experiments.

6. Optimization of Control Inputs and Command File Generation

For the optimization of the control inputs, we use the same framework as shown in supplementary figure 8. We define the observables (whose forward sensitivities are used to build the Fisher information matrix), the time between each input and the control parameters (genes). Each control parameter has a corresponding vector of input times and parameter values, both of these have a corresponding index. The agents randomly sample a sequence of input times of a random size for mutation i.e. if the flow device pumps in new lysate every 22.5 minutes then the agents in the algorithm can mutate a control parameter

in that specific 22.5 minute window or it can sample multiple adjacent time windows to effectively apply the mutation over a longer time window e.g. *n* * 22.5, where *n* is the window size. We noted that scores improved if we allowed the algorithm to set the inflow of compounds to 0 for a time as opposed to modifying the current inflow rate. As such the agents have a random change (20%) each iteration to set the inflow rate of a compound to 0.

Once the optimization is complete, we obtain a vector with times and inflow fractions for the control parameters K_{in}. These need to be translated to pump steps, which is done by the LabView software. Each step represents a replacement of approximately ~0.5% of the reactor volume (varies slightly per reactor and is compensated for with a calibration performed for each reactor in each device (see Methods)), thus translating the inflow fractions to the number of pump steps is done according to

$$N = \frac{IF}{RR} * RF * DNA(stock) \tag{11}$$

Where N is the number of steps, IF the inflow fraction, RR the reactor specific refresh ratio (the amount replaced by a single pump step) and RF the refresh fraction (the total amount of material that is freshed per refresh cycle, usually 40%). The number of steps used for the inflow of all CFPS components is saved in a textfile. The actual amount of DNA flown into the reactors is then calculated from the number of steps according to

$$[DNA] = N * RR * DNA(stock) \tag{12}$$

7. Testing different parameter subsets

In Supplementary Figure 24, we compare the predictive power of the model when we fit subsets of the database. Specifically, we fit the step experiments independently, then together, and finally the pulse experiments together. Fitted individually, the fluxes from each step in the IVTT process and between the different repressors are not balanced and we cannot predict the behaviour of new networks with any accuracy. Fitting all step experiments simultaneously and using the resulting parameters to predict new behaviour improves the prediction but is still not refined enough as the regulation parameters are not balanced. This balance improves as we fit the pulse experiments together. With the pulse sequence we screen a large part of the space of potential construct concentration combinations and the strength of those interactions at each point, providing us with more refined estimates. This is corroborated by the identifiability analysis in Supplementary Figure 15 and Figure 2c in the main text. To illustrate: the collinearity for the Hill coefficients with other parameters decreases in the model for a pulse experiment, which results in better Hill coefficient estimates and in turn better predictive power when we fit the model to a set of pulse experiments. From the step experiments alone, we can neither predict the behaviour of the bistable switch or the pulse decoder, because the regulation parameters are not sufficiently accurate.

Supplementary figures and tables

Supplementary Figure 10: deGFP expression from different promoters

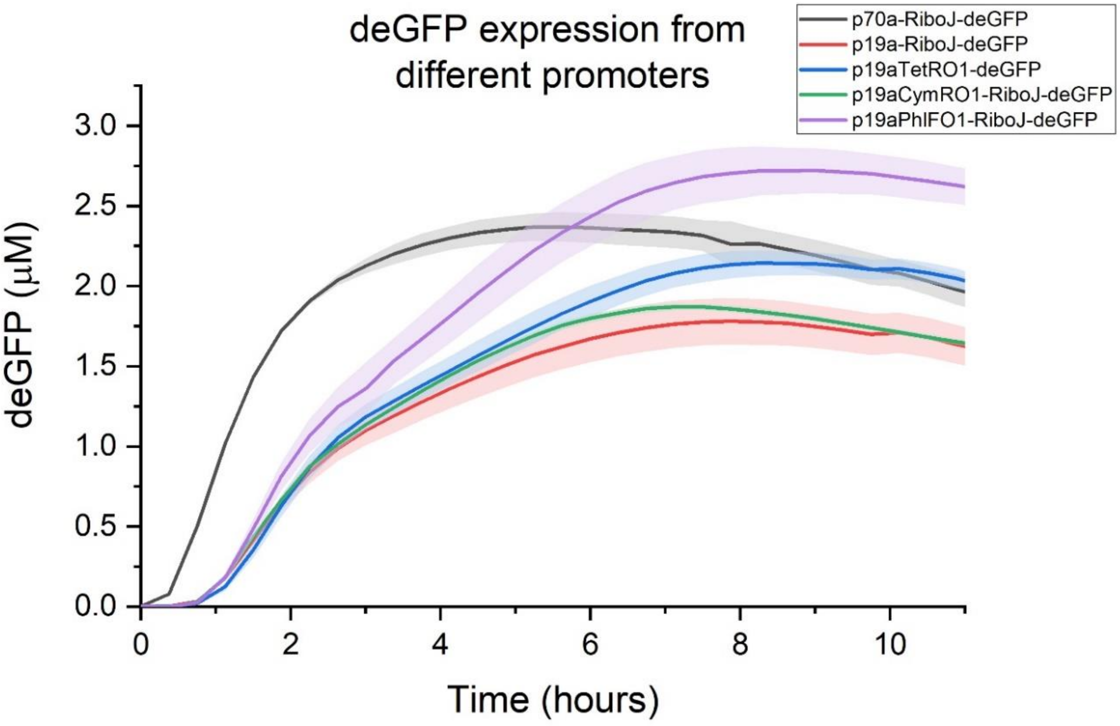

Supplementary Figure 10: Transcriptional efficiency is similar for all four p19a variants. Flow experiments are performed with 5 nM of either p70a-RiboJ-deGFP or any of the four p19a promoters with 5 nM of p70a-S19. Since all constructs contain the RiboJ insulator and the same deGFP sequence, translation rates can be assumed to be the same for all constructs. Differences in expression yield can thus be attributed solely to differences in transcription yield. Solid lines indicate the mean of duplicate measurements. The standard deviation around the mean is plotted as shaded areas in matching colors.

Supplementary Figure 11: Maturation rate determination

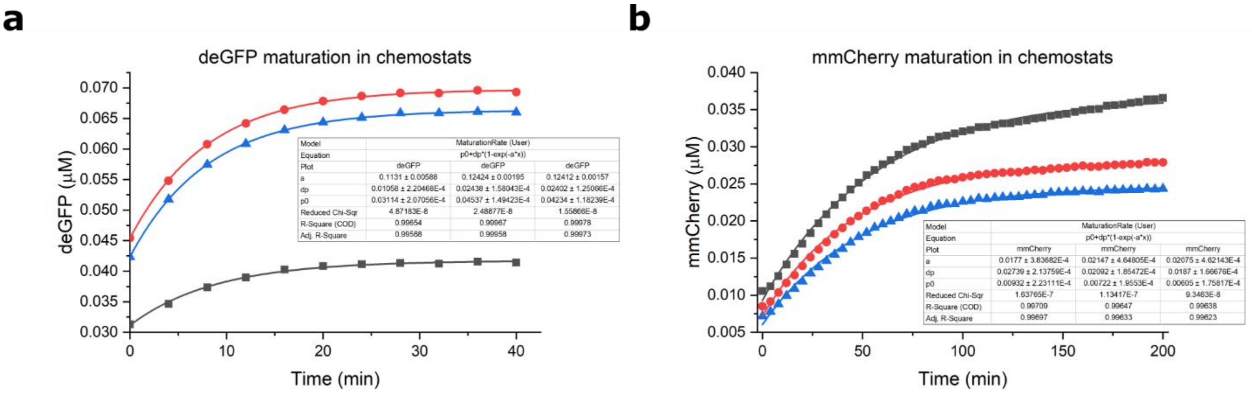

*Supplementary Figure 11: Maturation rates are measured directly for both deGFP and mmCherry as described in Niederholtmeyer et al.[7] In short, fluorescent increase after RNase A addition is followed over time (symbols). The line represents the least square fit to the data using the formula F(x) = P0 + dP(1-exp(-a*x)), with P0 the protein concentration at t=0, dP the total gain in protein concentration and a the maturation rate in min^{-1}. The inset shows the fitted values for all three parameters. a) deGFP maturation in chemostats. After 20 minutes of batch IVTT, RNase A (0.6 uM) is added. b) mmCherry maturation in chemostats. After 63 minutes of batch IVTT, RNase A (0.6 uM) is added.*

Supplementary Figure 12: Limited competition for ribosomes and coreRNAP

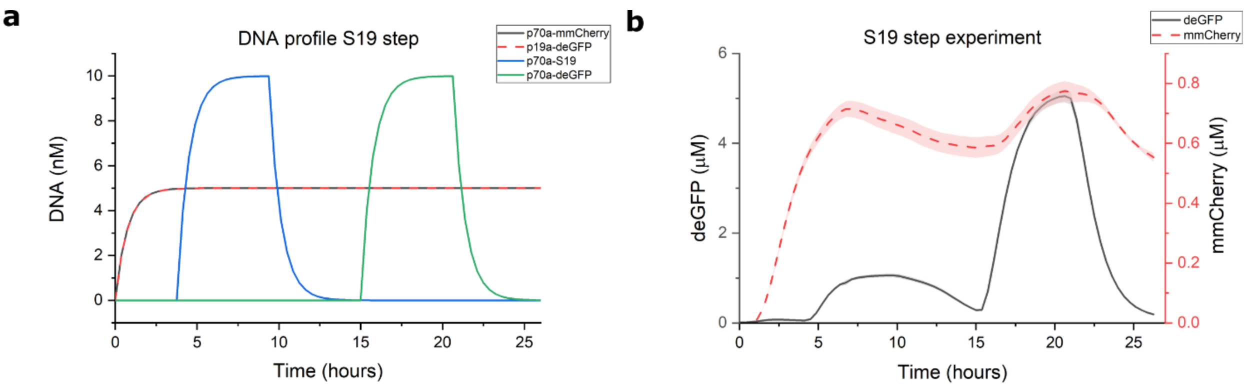

Supplementary Figure 12: Competition for ribosomes and coreRNAP can be neglected in our system. a) DNA profile for S19 step experiment. b) Output of the S19 step experiment. Solid and dashed lines indicate the mean of triplicate measurements. The standard deviation around the mean is plotted as shaded areas in matching colors.

Supplementary Figure 13: Initial calibration step experiments

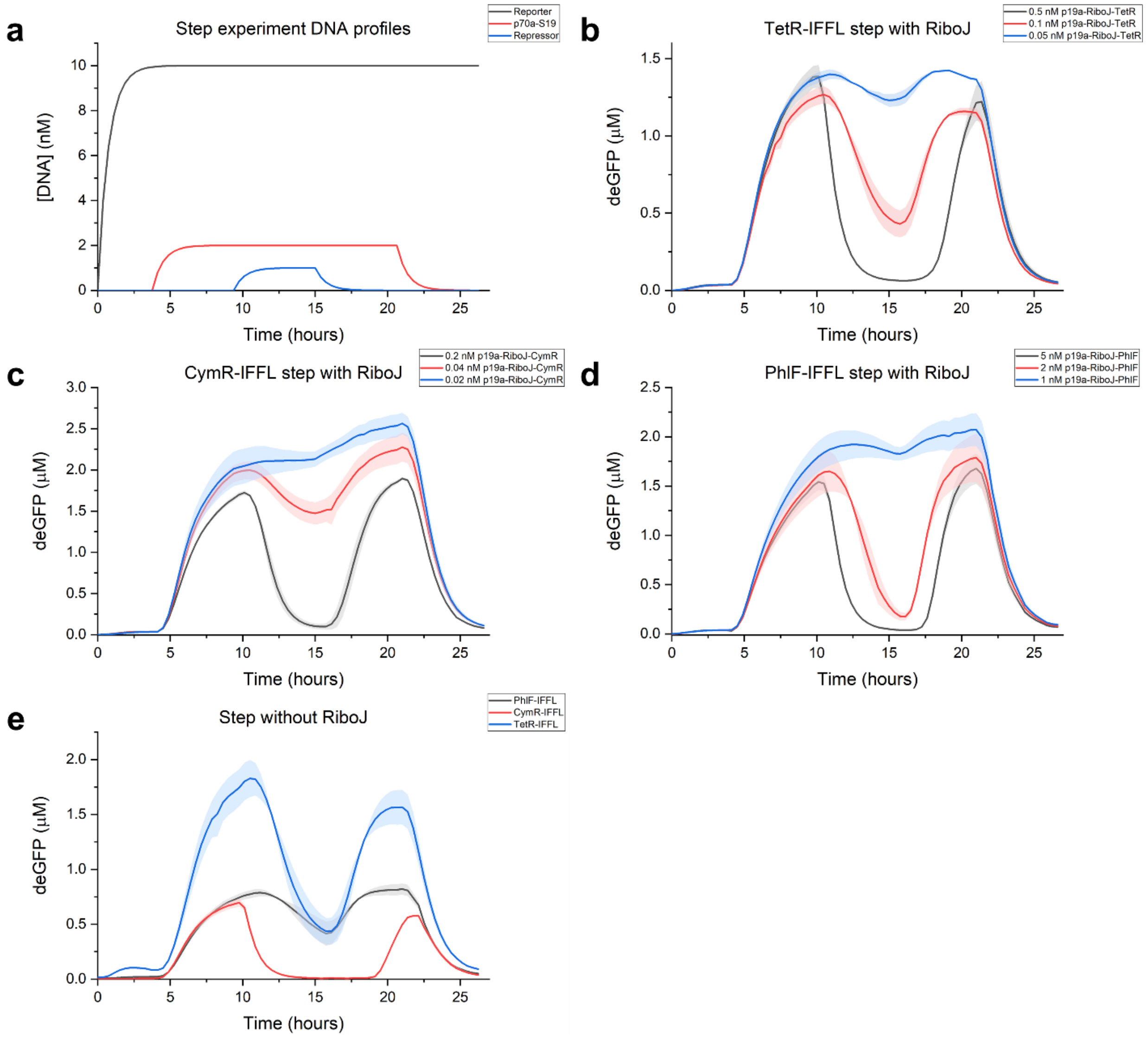

Supplementary Figure 13: Step experiment data for all IFFLs. a) DNA profile used for each experiment, the final DNA concentration reached for the repressor constructs is varied for each IFFL (see legends in b-d). For the experiments without RiboJ the repressor concentration used was kept constant at 1 nM. Concentrations of the reporter and activator constructs were 10 nM and 2 nM, respectively. Step experiment outputs are plotted against time for the TetR-FFL with RiboJ (b), CymR-FFL with RiboJ (c), PhlF-FFL with RiboJ(d) and All variants without RiboJ (e). Solid lines indicate the mean of duplicate (with RiboJ) or triplicate (without RiboJ) measurements. The standard deviation around the mean is plotted as shaded areas in matching colors.

Supplementary Figure 14: Pairwise collinearity indices for an in silico batch experiment.

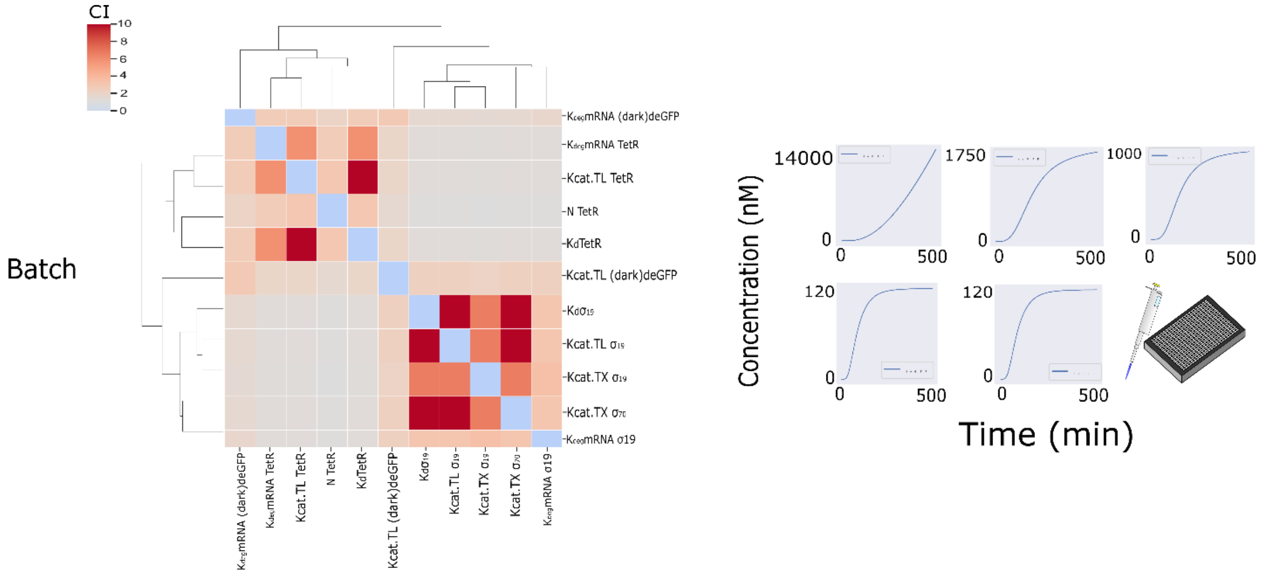

Supplementary Figure 14. The pairwise collinearity indices shown in figure 2, for the batch experiments with the data of the simulations (with 2nM p70a-σ19 *nM and 10 nM of p19aTetRO1-deGFP and a concentration range of 0.01, 0.1, 0.2, 0.5, 1 nM for the p19a-TetR gene). We show the time-course profiles of simulated deGFP signal.*

Supplementary Figure 15: Pairwise collinearity indices for in silico flow experiments.

Calibration Step

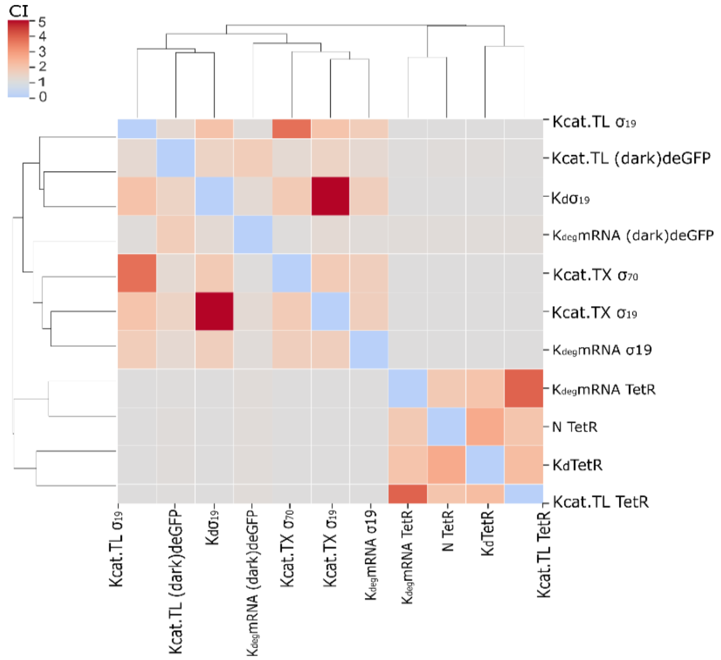

OED

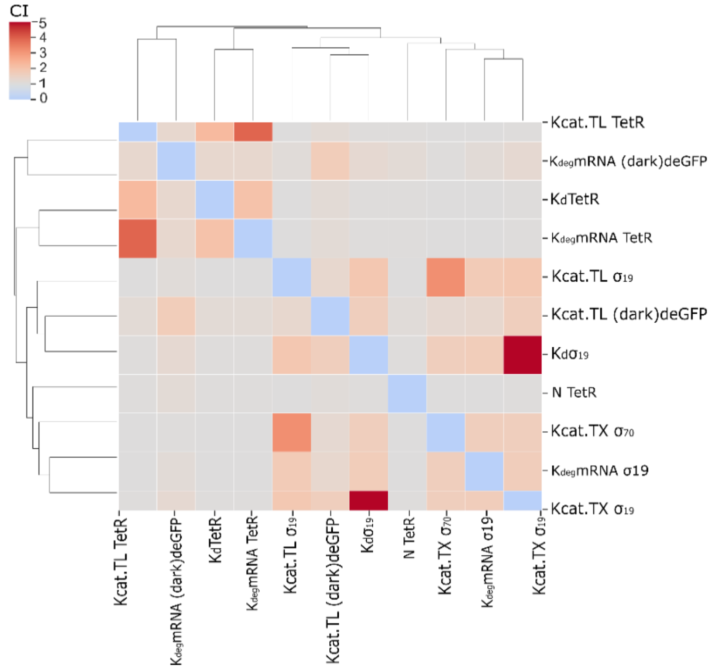

Supplementary Figure 15. The pairwise collinearity indices shown in figure 2. Especially the regulation parameters (hill coefficients and dissociation constant for TetR) are decoupled.

Supplementary Figure 16: Pairwise collinearity indices for all in silico flow experiments combined in the database.

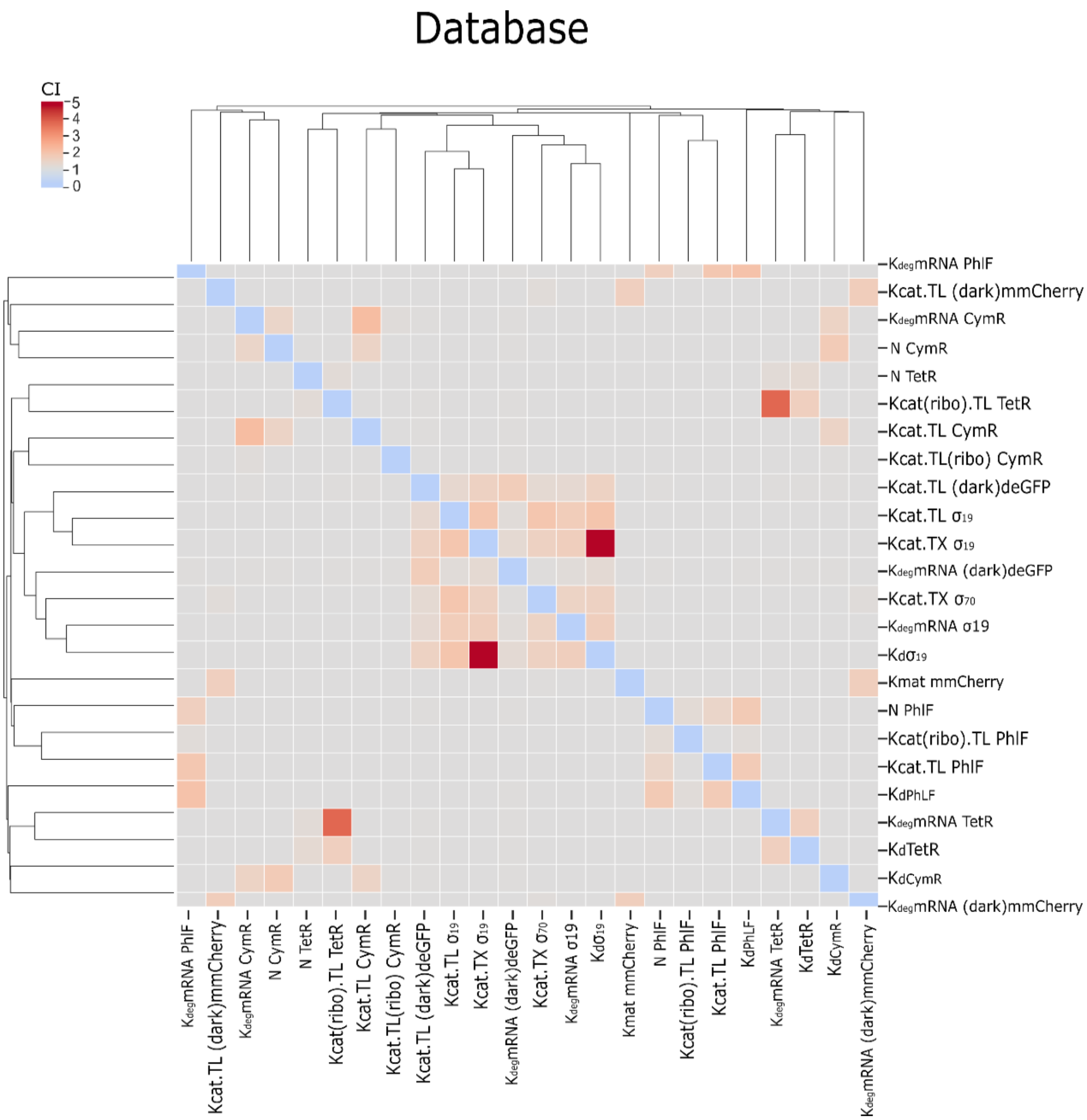

Supplementary Figure 16. The pairwise collinearities between parameters in the entire database.

Supplementary Figure 17: DNA profiles for optimized experiments with RiboJ

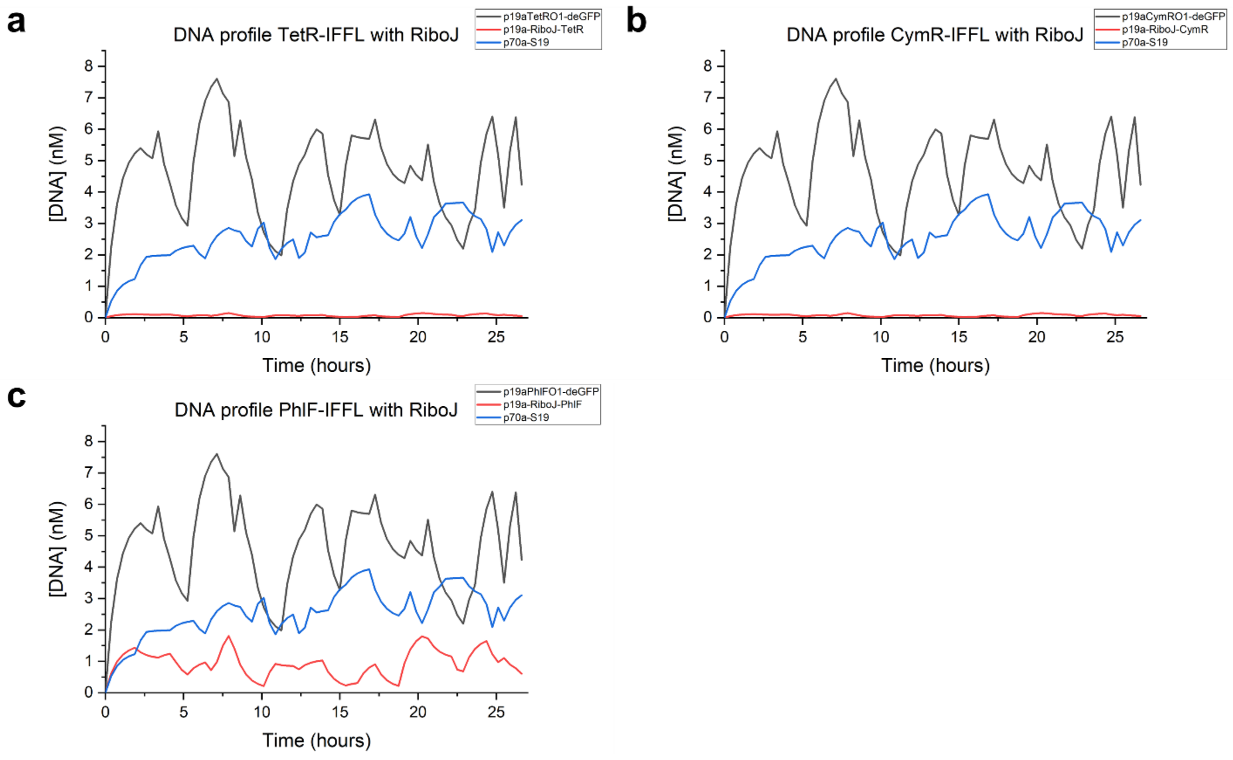

Supplementary Figure 17: DNA profiles for the optimized pulse experiments with RiboJ for a) TetR-IFFL, b) CymR-IFFL and c) PhlF-IFFL. The pattern is the same for all three FFLs, only the concentration ranges in which the repressor changes vary.

Supplementary Figure 18: DNA profiles and deGFP output for optimized experiments without RiboJ

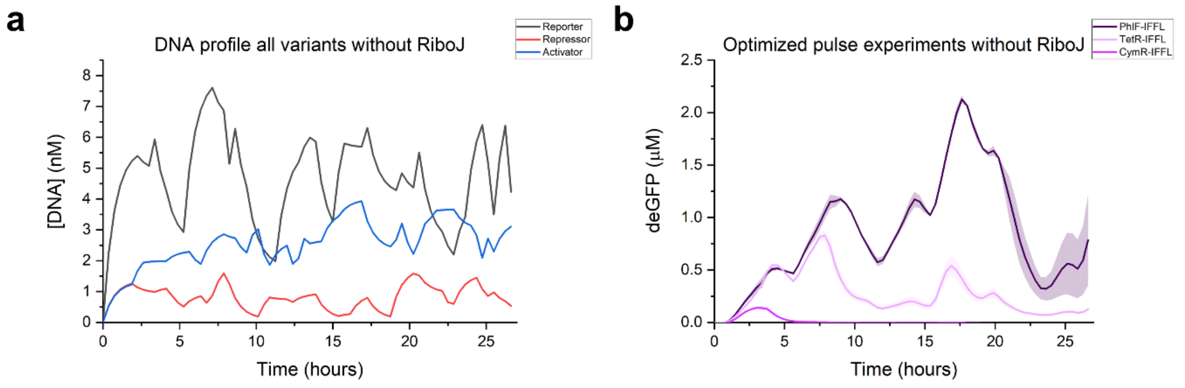

Supplementary Figure 18: DNA profile (a) and output (b) for the optimized pulse experiments without RiboJ. The same DNA profile was used for all three variants. Solid lines indicate the mean of triplicate measurements. The standard deviation around the mean is plotted as shaded areas in matching colors.

Supplementary Figure 19: Optimized experiment for S19 activation and mmCherry expression

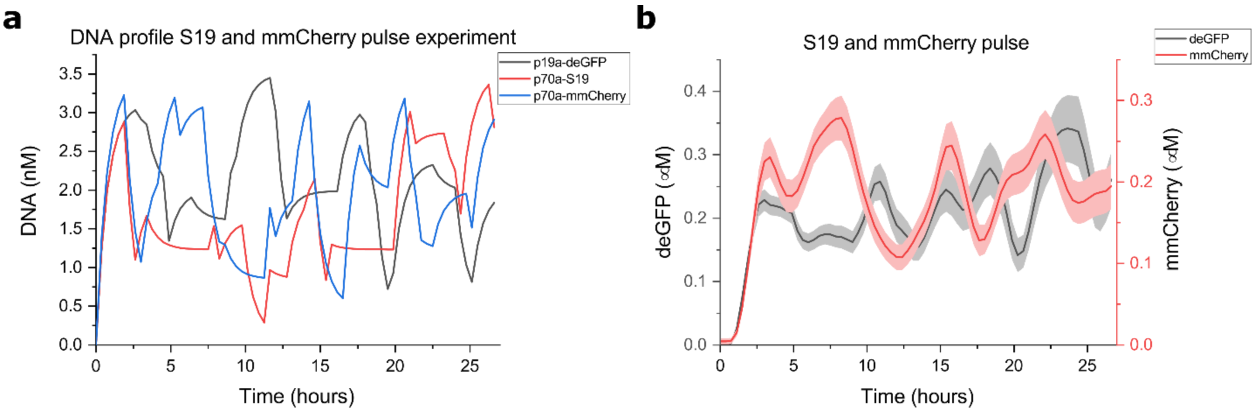

Figure 19: DNA profile (a) and output (b) for the optimized pulse experiments for σ19 activation and mmCherry expression. Solid lines indicate the mean of triplicate measurements. The standard deviation around the mean is plotted as shaded areas in matching colors..

Supplementary Figure 20: Parameter distributions from calibration step and database

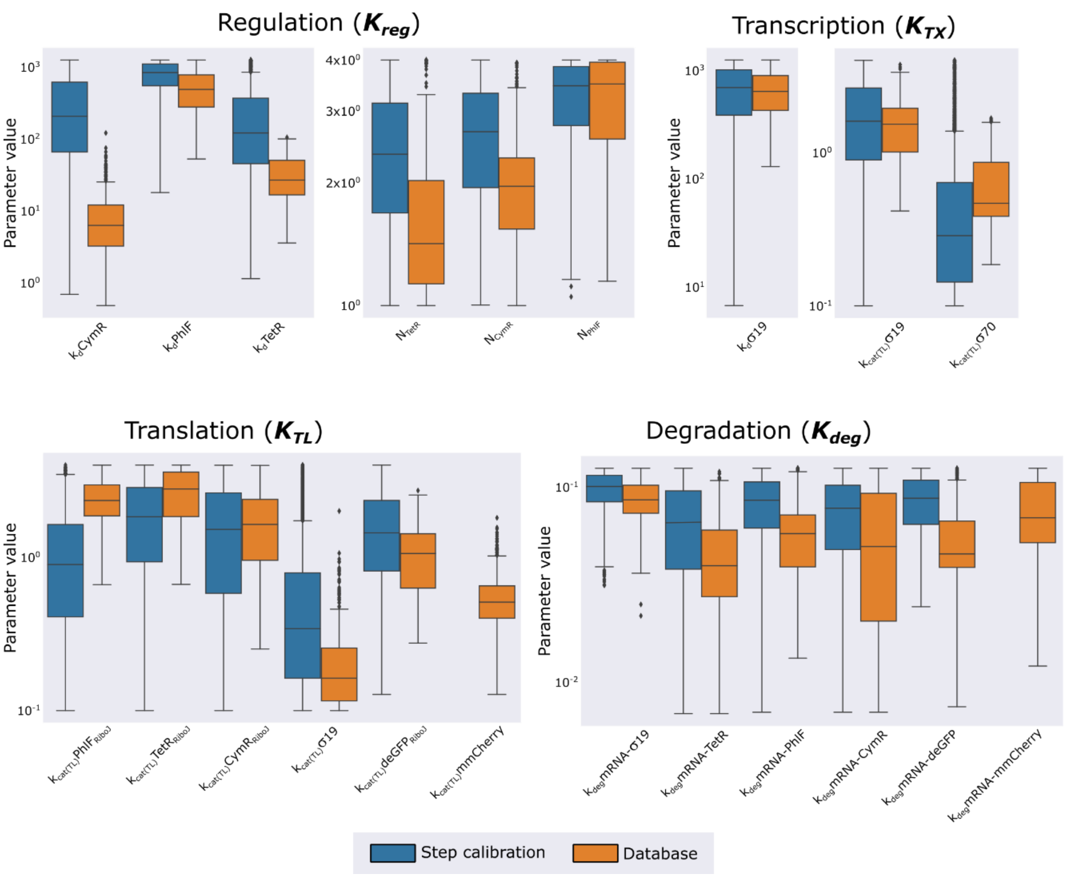

Supplementary Figure 20: Parameter values and distributions plotted using the parameter sets which score within 42.5% of the best scoring set (about the maximum variation seen in experiments). Parameter are characterized by the four categories. For each parameter the distributions are indicated for both the step calibration (blue) and database (orange). The box itself displays the quartiles of the middle 50%, with a line showing the median value. The whiskers of the box show the highest and lowest values, outliers shown as individual datapoints. 1580 datasets were used for the Step calibration and 600 for the database distributions.

Supplementary Figure 21: DNA profile and deGFP output for a pulse decoder experiment

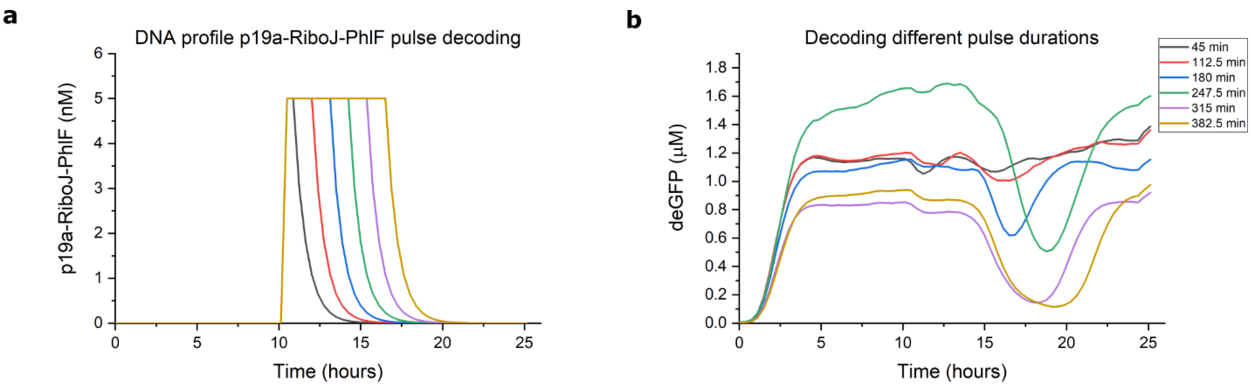

Supplementary Figure 21: Example of an experiment to determine the response curve for the pulse decoder network. a) DNA profile for the p19a-RiboJ-PhlF during an experiment. b) Experimental output belonging to the DNA profiles in a). Concentrations of the other genes were 2 nM p70a-S19, 5 nM p19aTetRO1-deGFP, 0.5 nM p19aCymRO1-TetR and 0.5 nM p19aPhlFO1-CymR and were present at these concentrations during the entire experiment. To convert the experimental output to the datapoints shown in figure 4.c, we divided the minimal deGFP yield after 12.5 hours by the average yield between 5 and 12.5 hours.

Supplementary Figure 22: Determining the switching regime for the bistable switch

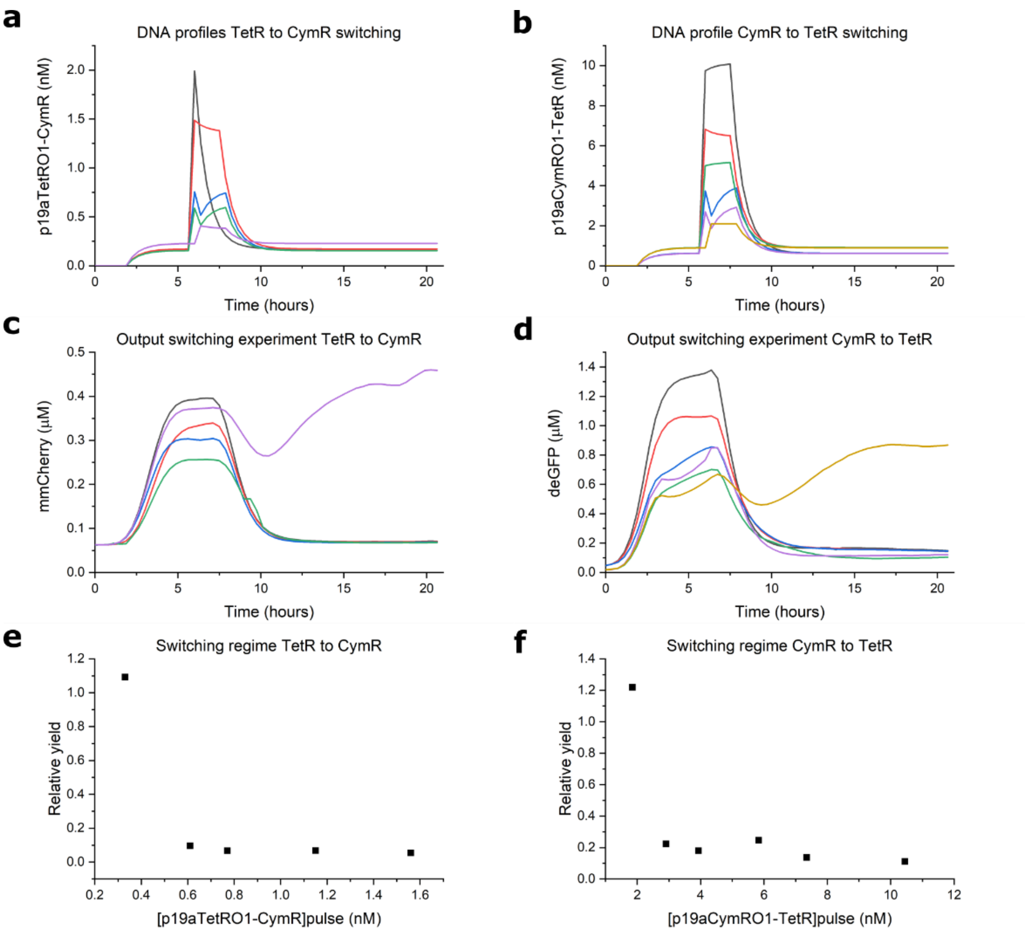

Supplementary Figure 22: Determining concentrations where the system would switch. DNA profiles of the different switching pulses used for determining the switching regime towards the CymR state (a) and the TetR state (b). The rest of the DNA concentrations were 2 nM p70a-S19, 5 nM of both p19aTetRO1-deGFP and p19aCymRO1-mmCherry and 0.2 nM p19aTetRO1-CymR or either 0.8 nM p19aCymRO1-TetR. The experimental output for switching towards the CymR state (c) and the TetR state (d) corresponding to the input patterns shown in (a) and (b), respectively. The purple plot in c) and the yellow plot in d) show no switching, the system goes back to the original state after a short drop in expression. In these instances, the bifurcation point is not reached and so the system doesn't switch. The switching regimes are determined in both directions by plotting the ratio between the expression of mmCherry (e) and deGFP (f) in the timeframes 5-7.5 hours and 15-20 hours.

Supplementary Figure 23: Difference in translation rate with and without the RiboJ element for the three repressors

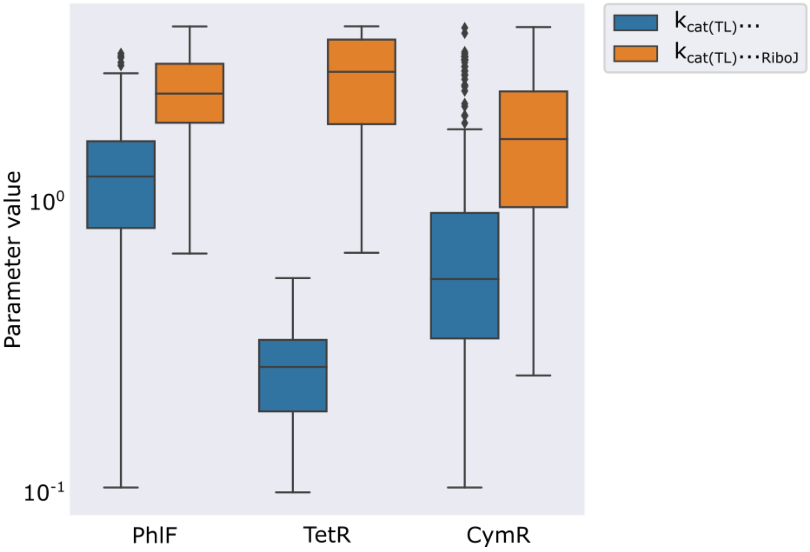

Supplementary Figure 23: Parameter values and distributions of the translation rates with and without RiboJ element plotted using the parameter sets which score within 42.5% of the best scoring set (about the maximum variation seen in experiments). Parameter sets from the database fitting have been used. The box itself displays the quartiles of the middle 50%, with a line showing the median value. The whiskers of the box show the highest and lowest values, outliers shown as individual datapoints. 600 datasets were used to create the boxplots.

Supplementary Figure 24: Additional predictions indicating the predictive power of the parameter sets derived using our approach.

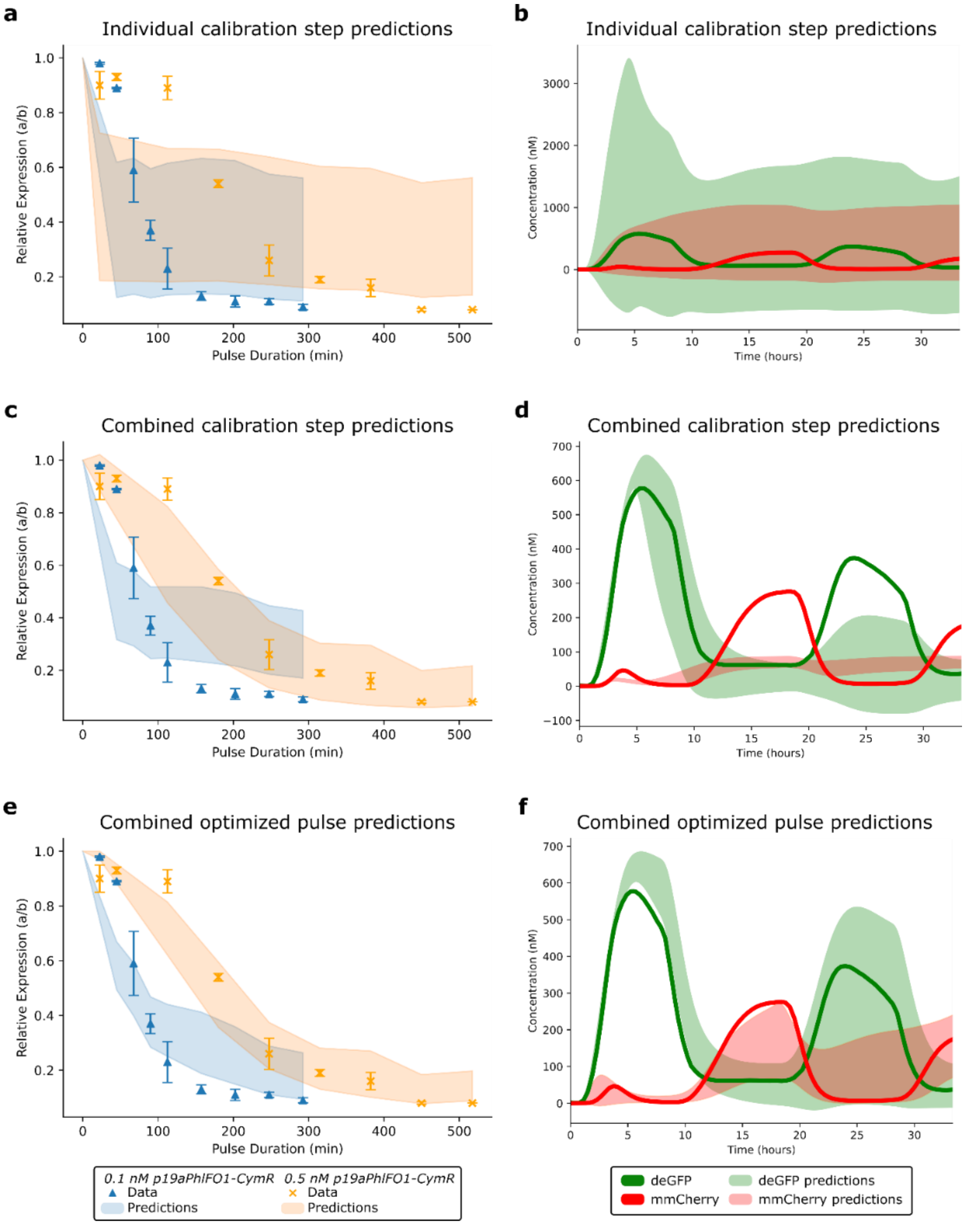

Supplementary Figure 24: We compare the predictive power of the model for both the pulse decoder and bistable switch when we fit subsets of the database, specifically we fit the step experiments independently (a and b), then together (c and d), and finally the pulse experiments together (e and f). Note that for the fit of all step experiments for the bistable switch (d), the concentrations of deGFP match for the initial pulse but the regulation parameters are not sufficient to predict bistability. The pulse experiments do predict bistability but have more uncertainty compared to figure 5 in the main text. The step experiments resemble the pulse decoder experiments yet these steps did not probe very low concentration ranges thus the predictions are worse for the 0.1 nM concentration of p19aPhlFO1-CymR. Error bars in in a, c and e indicate mean and standard deviation over duplicate measurements. Solid lines in b, d and f indicate the mean over triplicate measurements. The standard deviation of the mean for the first quantile of the highest scoring simulated experiments (predictions) is plotted as shaded areas in corresponding colors. Number of parameter sets used are a,b 75, c,d, 162 e,f 55.

Supplementary Figure 25: Parameter distributions from calibration step, initial database and updated databases

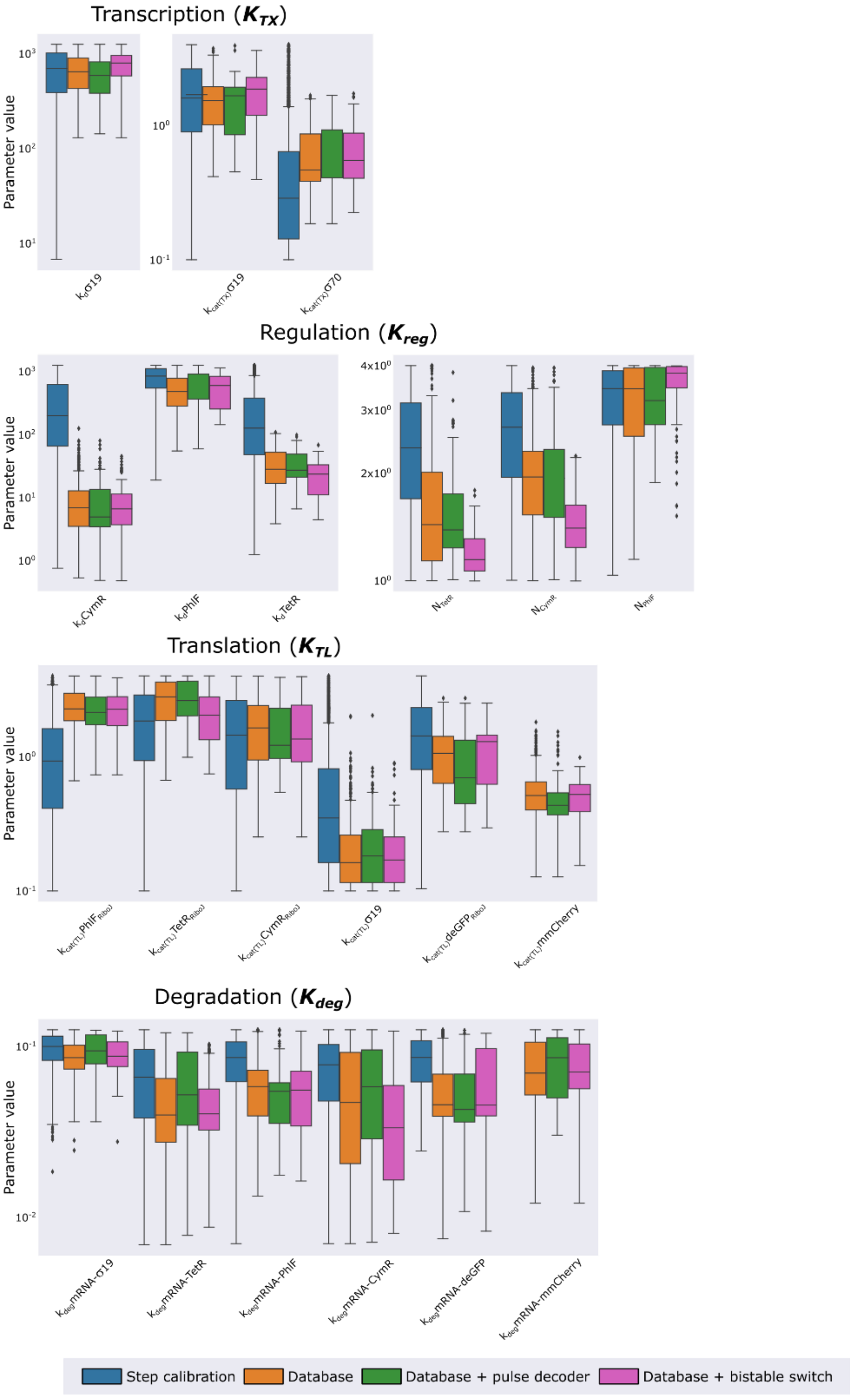

Supplementary Figure 25: Parameter values and distributions plotted using the parameter sets which score within 42.5% of the best scoring set (about the maximum variation seen in experiments). Parameter are characterized by the four categories. For each parameter the distributions are indicated for the step calibration (blue), database (orange) and updated databases with the pulse decoder (green) and bistable switch (purple) experiments. The box itself displays the quartiles of the middle 50%, with a line showing the median value. The whiskers of the box show the highest and lowest values, outliers shown as individual datapoints. 1580 datasets were used for the Step calibration and 600 for the database distributions and 150 each for the updated databases.

Supplementary Figure 26: Layout of microfluidic chemostats

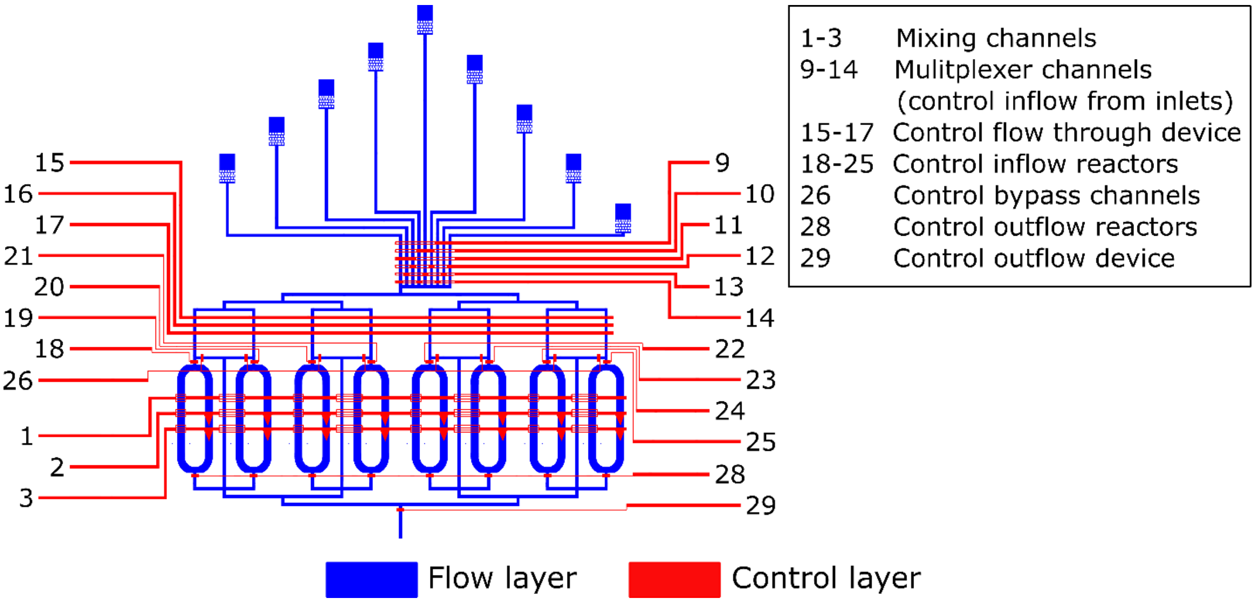

Supplementary Figure 26: Layout of the microfluidic device based on the devices from Niederholtmeyer et al.[7] and Van der Linden et al..[8] The devices consist of two distinct layers, a flow layer (blue) and a control layer (red). At intersections of the two, pressurizing the control layer result in blocking the flow in the flow layer. The control layers are numbered, the purpose of each channel is provided in the legend.

Supplementary Figure 27: Lysate cooler

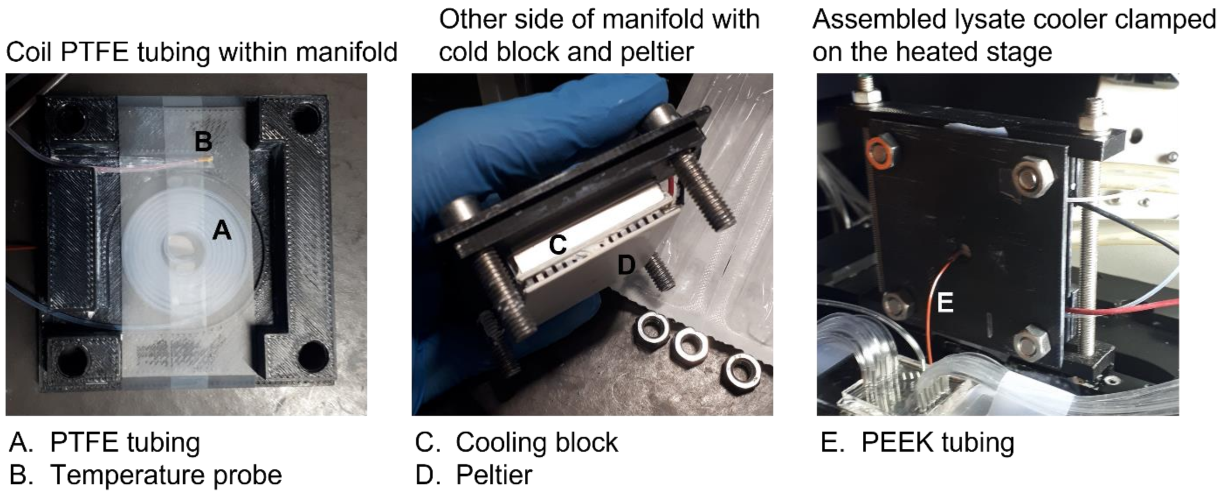

Supplementary Figure 27: Lysate cooler consists of a 3D printed manifold, which clamps the coiled-up PTFE tubing (A) together with the temperature probe (B) against the cold side of a 4x4 cm peltier (D). The hot-side of the peltier is pressed against a cooling block (C), connected to a cooled water bath at 16C, functioning as a heat sink. The lysate cooler is held in place by another 3D printed clamp, clamping the lysate cooler to the microscope stage. Lysate is transported from the lysate cooler to the device using PEEK tubing with an inner diameter of 125 um to decrease the death volume (the volume between the cooler and the device) as much as possible.

Supplementary Figure 28: Microfluidic setup overview

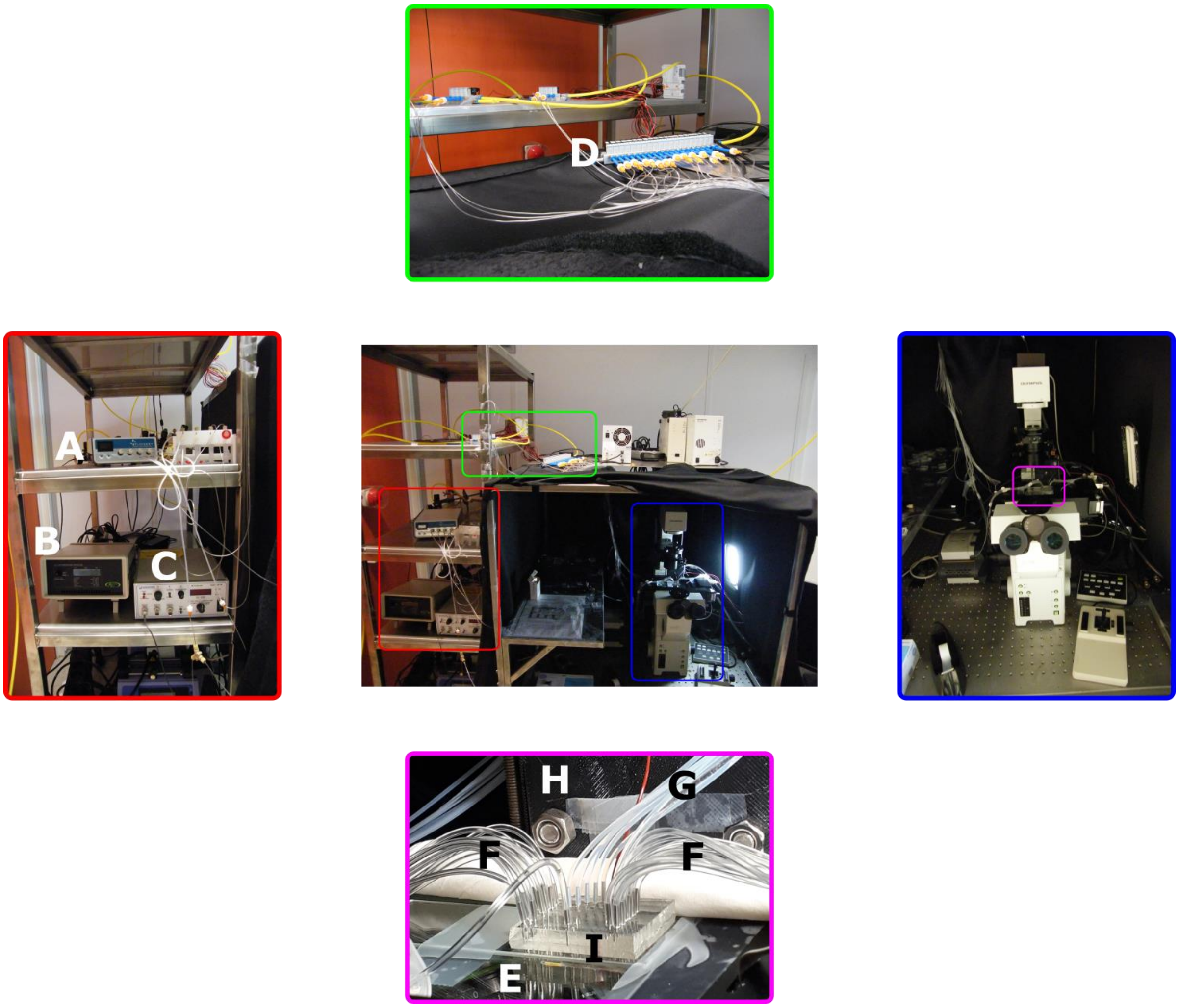

Supplementary Figure 28: Overview of the microfluidic setup (middle), with zoomed in pictures of the controllers (red box), actuators controlling the control channels (green box), inverted microscope (blue box) and device mounted on temperature stage (purple box). A) Fluigent controller applies a constant pressure of 50 mbar to all fluid lines. B) Temperature controller for the temperature-controlled stage C) Temperature controller for the lysate cooler D) Array of two-way valves, each valve controls a single control channel which can be either pressurized or not. E) Temperature controlled stage F) Tubing filled with MQ or air plugged into the control channels of the device. G) PTFE tubing filled with DNA/MQ plugged into the inlets of the flow layer. H) lysate cooler with in red the PEEK tubing. I) PDMS chip bonded to a glass microscopy slide.

Supplementary table 1: Sequence overview

Supplementary table 1: Sequences used in this work. Constructs are created by simply pasting the different parts as presented here together. Colours indicate RiboJ (yellow), operators (different shades of purple/pink) and ORFs (green, red and different shades of purple/pink matching the operators). References used to design the sequences are provided behind the names.

Name	Sequence with 5' GGA overhang
5' + p19a[9]	From pTXTL-p19a-deGFP plasmid from Daicel Arbor Biosciences
3' + T500[9]	From pTXTL-p70a-deGFP plasmid from Daicel Arbor Biosciences
UTR1_RiboJ[9]	From pTXTL-p70a-deGFP plasmid from Daicel Arbor Biosciences
UTR1_prom[9]	From pTXTL-p70a-deGFP plasmid from Daicel Arbor Biosciences
RiboJ_prom[10]	GCAGTGTCACCGGATGTGCTTTCCGGTCTGATGAGTCCGTGAGGACGAAACAGCCTCTACAA<br>ATAATTTTGTTTAA
RiboJ_oper[10]	AAGCTGTCACCGGATGTGCTTTCCGGTCTGATGAGTCCGTGAGGACGAAACAGCCTCTACAA<br>ATAATTTTGTTTAA
TetRO1[11]	GCAGTCCCTATCAGTGATAGAGATCACACTCCTTC
CymRO1[11]	GCAGACAAACAGACAATCTGGTCTGTTTGTATTAC
PhlFO1[11]	GCAGATGATACGAAACGTACCGTATCGTTAAGGTC
deGFP[9]	AATGGAGCTTTTCACTGGCGTTGTTCCCATCCTGGTCGAGCTGGACGGCGACGTAAACGGCC<br>ACAAGTTCAGCGTGTCCGGCGAGGGCGAGGGCGATGCCACCTACGGCAAGCTGACCCTGAA<br>GTTCATCTGCACCACCGGCAAGCTGCCCGTGCCCTGGCCCACCCTCGTGACCACCCTGACCTA<br>CGGCGTGCAGTGCTTCAGCCGCTACCCCGACCACATGAAGCAGCACGACTTCTTCAAGTCCG<br>CCATGCCCGAAGGCTACGTCCAGGAGCGCACCATCTTCTTCAAGGACGACGGCAACTACAAG<br>ACCCGCGCCGAGGTGAAGTTCGAGGGCGACACCCTGGTGAACCGCATCGAGCTGAAGGGCA<br>TCGACTTCAAGGAGGACGGCAACATCCTGGGGCACAAGCTGGAGTACAACTACAACAGCCA<br>CAACGTCTATATCATGGCCGACAAGCAGAAGAACGGCATCAAGGTGAACTTCAAGATCCGCC<br>ACAACATCGAGGACGGCAGCGTGCAGCTCGCCGACCACTACCAGCAGAACACCCCCATCGGC<br>GACGGCCCCGTGCTGCTGCCCGACAACCACTACCTGAGCACCCAGTCCGCCCTGAGCAAAGA<br>CCCCAACGAGAAGCGCGATCACATGGTCCTGCTGGAGTTCGTGACCGCCGCCGGGATCTAAC<br>T
mmCherry[9]	AATGGTGAGCAAGGGCGAAGAAGATAACATGGCCATCATCAAGGAGTTCATGCGCTTCAAG<br>GTGCACATGGAGGGCTCCGTGAACGGCCACGAGTTCGAGATCGAGGGCGAGGGCGAGGGC<br>CGCCCCTACGAGGGCACCCAGACCGCCAAGCTGAAGGTGACCAAGGGTGGCCCCCTGCCCTT<br>CGCCTGGGACATCCTGTCCCCTCAGTTCATGTACGGCTCCAAGGCCTACGTGAAGCACCCCGC<br>CGACATCCCCGACTACTTGAAGCTGTCCTTCCCCGAGGGCTTCAAGTGGGAGCGCGTGATGA<br>ACTTCGAGGACGGCGGCGTGGTGACCGTGACCCAGGACTCCTCCCTGCAGGACGGCGAGTT<br>CATCTACAAGGTGAAGCTGCGCGGCACCAACTTCCCCTCCGACGGCCCCGTAATGCAGAAGA<br>AGACCATGGGCTGGGAGGCCTCCTCCGAGCGGATGTACCCCGAGGACGGCGCCCTGAAGGG<br>CGAGATCAAGCAGAGGCTGAAGCTGAAGGACGGCGGCCACTACGACGCTGAGGTCAAGAC<br>CACCTACAAGGCCAAGAAGCCCGTGCAGCTGCCCGGCGCCTACAACGTCAACATCAAGTTGG<br>ACATCACCTCCCACAACGAGGACTACACCATCGTGGAACAGTACGAACGCGCCGAGGGCCGC<br>CACTCCACCGGCGGCATGGACGAGCTGTACAAGTAACT
TetR[12]	AATGTCTAGATTAGATAAAAGTAAAGTGATTAACAGCGCATTAGAGCTGCTTAATGAGGTCG<br>GAATCGAAGGTTTAACAACCCGTAAACTCGCCCAGAAGCTAGGTGTAGAGCAGCCTACATTG<br>TATTGGCATGTAAAAAATAAGCGGGCTTTGCTCGACGCCTTAGCCATTGAGATGTTAGATAG<br>GCACCATACTCACTTTTGCCCTTTAGAAGGGGAAAGCTGGCAAGATTTTTTACGTAATAACGC<br>TAAAAGTTTTAGATGTGCTTTACTAAGTCATCGCGATGGAGCAAAAGTACATTTAGGTACACG<br>GCCTACAGAAAAACAGTATGAAACTCTCGAAAATCAATTAGCCTTTTTATGCCAACAAGGTTT

	TTCACTAGAGAATGCATTATATGCACTCAGCGCTGTGGGGCATTTTACTTTAGGTTGCGTATT<br>GGAAGATCAAGAGCATCAAGTCGCTAAAGAAGAAAGGGAAACACCTACTACTGATAGTATG<br>CCGCCATTATTACGACAAGCTATCGAATTATTTGATCACCAAGGTGCAGAGCCAGCCTTCTTA<br>TTCGGCCTTGAATTGATCATATGCGGATTAGAAAAACAACTTAAATGTGAAAGTGGGTCTTAA<br>CT
CymR[12]	AATGAGCCCGAAACGTCGTACCCAGGCAGAACGTGCAATGGAAACCCAGGGTAAACTGATT<br>GCAGCAGCACTGGGTGTTCTGCGTGAAAAAGGTTATGCAGGTTTTCGTATTGCAGATGTTCC<br>GGGTGCAGCCGGTGTTAGCCGTGGTGCACAGAGCCATCATTTTCCGACCAAACTGGAACTGC<br>TGCTGGCAACCTTTGAATGGCTGTATGAGCAGATTACCGAACGTAGCCGTGCACGTCTGGCA<br>AAACTGAAACCGGAAGATGATGTTATTCAGCAGATGCTGGATGATGCAGCAGAATTTTTTCT<br>GGATGATGATTTTAGCATCAGCCTGGATCTGATTGTTGCAGCAGATCGTGATCCGGCACTGC<br>GTGAAGGTATTCAGCGTACCGTTGAACGTAATCGTTTTGTTGTTGAAGATATGTGGCTGGGT<br>GTGCTGGTGAGCCGTGGTCTGAGCCGTGATGATGCCGAAGATATTCTGTGGCTGATTTTTAA<br>CAGCGTTCGTGGTCTGGCAGTTCGTAGCCTGTGGCAGAAAGATAAAGAACGTTTTGAACGTG<br>TGCGTAATAGCACCCTGGAAATTGCACGTGAACGTTATGCAAAATTCAAACGTTAACT
PhlF[12]	AATGGCACGTACCCCGAGCCGTAGCAGCATTGGTAGCCTGCGTAGTCCGCATACCCATAAAG<br>CAATTCTGACCAGCACCATTGAAATCCTGAAAGAATGTGGTTATAGCGGTCTGAGCATTGAA<br>AGCGTTGCACGTCGTGCCGGTGCAAGCAAACCGACCATTTATCGTTGGTGGACCAATAAAGC<br>AGCACTGATTGCCGAAGTGTATGAAAATGAAAGCGAACAGGTGCGTAAATTTCCGGATCTGG<br>GTAGCTTTAAAGCCGATCTGGATTTTCTGCTGCGTAATCTGTGGAAAGTTTGGCGTGAAACCA<br>TTTGTGGTGAAGCATTTCGTTGTGTTATTGCAGAAGCACAGCTGGACCCTGCAACCCTGACCC<br>AGCTGAAAGATCAGTTTATGGAACGTCGTCGTGAGATGCCGAAAAAACTGGTTGAAAATGCC<br>ATTAGCAATGGTGAACTGCCGAAAGATACCAATCGTGAACTGCTGCTGGATATGATTTTTGG<br>TTTTTGTTGGTATCGCCTGCTGACCGAACAGCTGACCGTTGAACAGGATATTGAAGAATTTAC<br>CTTCCTGCTGATTAATGGTGTTTGTCCGGGTACACAGCGTTAACT

References

1. Fröhlich, F. *et al.* AMICI: high-performance sensitivity analysis for large ordinary differential equation models. doi:10.1093/bioinformatics/btab227.
2. Choi, K. *et al.* Tellurium: An Extensible Python-based Modeling Environment for Systems and Synthetic Biology. *Bio Systems* **171**, 74 (2018).
3. Smith, L. P., Bergmann, F. T., Chandran, D. & Sauro, H. M. Antimony: a modular model definition language. *Bioinformatics* **25**, 2452–2454 (2009).
4. Kreutz, C., Raue, A., Kaschek, D. & Timmer, J. Profile likelihood in systems biology. *The FEBS Journal* **280**, 2564–2571 (2013).
5. Sinkoe, A. & Hahn, J. Optimal Experimental Design for Parameter Estimation of an IL-6 Signaling Model. *Processes 2017, Vol. 5, Page 49* **5**, 49 (2017).
6. Gábor, A., Villaverde, A. F. & Banga, J. R. Parameter identifiability analysis and visualization in large-scale kinetic models of biosystems. *BMC Systems Biology* **11**, 1–16 (2017).
7. Niederholtmeyer, H., Stepanova, V. & Maerkl, S. J. Implementation of cell-free biological networks at steady state. *Proceedings of the National Academy of Sciences* **110**, 15985–15990 (2013).
8. van der Linden, A. J. *et al.* A Multilayer Microfluidic Platform for the Conduction of Prolonged Cell-Free Gene Expression. *JoVE (Journal of Visualized Experiments)* **2019**, e59655 (2019).
9. Garamella, J., Marshall, R., Rustad, M. & Noireaux, V. The All E. coli TX-TL Toolbox 2.0: A Platform for Cell-Free Synthetic Biology. *ACS Synthetic Biology* **5**, 344–355 (2016).
10. Mutalik, V. K. *et al.* Precise and reliable gene expression via standard transcription and translation initiation elements. *Nature Methods* **10**, 354–360 (2013).
11. Zong, Y. *et al.* Insulated transcriptional elements enable precise design of genetic circuits. *Nature Communications* **8**, 52 (2017).
12. Stanton, B. C. *et al.* Genomic mining of prokaryotic repressors for orthogonal logic gates. *Nature Chemical Biology* **10**, 99–105 (2014).
