# Supplementary material for: A microfluidic optimal experimental design platform for forward design of cell-free genetic networks
Source: Nat Commun. 2022 Jun 24;13:3626. doi: 10.1038/s41467-022-31306-3 (PMC9232554; doi:10.1038/s41467-022-31306-3)
Supplement: Supplementary file 2 — Reporting Summary [file 41467_2022_31306_MOESM2_ESM.pdf]

nature portfolio

Corresponding author(s): *Double-blind peer review submissions: write DBPR and your manuscript number here instead of author names.*

Last updated by author(s): *YYYY-MM-DD*

Reporting Summary

Nature Portfolio wishes to improve the reproducibility of the work that we publish. This form provides structure for consistency and transparency in reporting. For further information on Nature Portfolio policies, see our Editorial Policies and the Editorial Policy Checklist.

Statistics

For all statistical analyses, confirm that the following items are present in the figure legend, table legend, main text, or Methods section.

n/a	Confirmed	
☐	☒	The exact sample size (*n*) for each experimental group/condition, given as a discrete number and unit of measurement
☐	☒	A statement on whether measurements were taken from distinct samples or whether the same sample was measured repeatedly
☒	☐	The statistical test(s) used AND whether they are one- or two-sided<br>*Only common tests should be described solely by name; describe more complex techniques in the Methods section.*
☐	☒	A description of all covariates tested
☐	☒	A description of any assumptions or corrections, such as tests of normality and adjustment for multiple comparisons
☐	☒	A full description of the statistical parameters including central tendency (e.g. means) or other basic estimates (e.g. regression coefficient) AND variation (e.g. standard deviation) or associated estimates of uncertainty (e.g. confidence intervals)
☒	☐	For null hypothesis testing, the test statistic (e.g. *F*, *t*, *r*) with confidence intervals, effect sizes, degrees of freedom and *P* value noted<br>*Give P values as exact values whenever suitable.*
☒	☐	For Bayesian analysis, information on the choice of priors and Markov chain Monte Carlo settings
☐	☒	For hierarchical and complex designs, identification of the appropriate level for tests and full reporting of outcomes
☒	☐	Estimates of effect sizes (e.g. Cohen's *d*, Pearson's *r*), indicating how they were calculated

Our web collection on statistics for biologists contains articles on many of the points above.

Software and code

Policy information about availability of computer code

Data collection	Microfluidic chemostats were controlled using LabView (we used the code created by Van der Linden et al. JOVE 2019).<br>Data acquisition was done using ImageJ.
Data analysis	Maturation rates were determined using Origin.<br>All other data was analyzed using Python Software>3.7 (anaconda) from the python software foundation (Delaware USA).<br>Packages include AMICI, libSBML, Tellurium, pyDOE, Libroadrunner, Antimony and Openblas. For more information see the github (https://github.com/huckgroup, (OED folder, 10.5281/zenodo.6610321)).

For manuscripts utilizing custom algorithms or software that are central to the research but not yet described in published literature, software must be made available to editors and reviewers. We strongly encourage code deposition in a community repository (e.g. GitHub). See the Nature Portfolio guidelines for submitting code & software for further information.

Data

Policy information about availability of data

All manuscripts must include a data availability statement. This statement should provide the following information, where applicable:

- Accession codes, unique identifiers, or web links for publicly available datasets
- A description of any restrictions on data availability
- For clinical datasets or third party data, please ensure that the statement adheres to our policy

All used parameter sets can be found in Supplementary Data 1. The raw datafiles generated during and/or analysed during the current study are available from the corresponding author on reasonable request.
The raw images/grey values are only available on request as these are not transferable between expression systems. Grey values depend heavily on microfluidic

device/microscope/light source used.

Field-specific reporting

Please select the one below that is the best fit for your research. If you are not sure, read the appropriate sections before making your selection.

☒ Life sciences ☐ Behavioural & social sciences ☐ Ecological, evolutionary & environmental sciences

For a reference copy of the document with all sections, see nature.com/documents/nr-reporting-summary-flat.pdf

Life sciences study design

All studies must disclose on these points even when the disclosure is negative.

Sample size	Experiments were performed in either duplicate or triplicate. When only a trend was to be identified, different conditions were tested once. All experiments were performed using the same batch of lysate.
Data exclusions	Data from flow experiments were only excluded when the reactor itself was malfunctioning. For example, air bubbles or a broken valve in the reactor would lead to improper functioning of a specific reactor. The data from these reactors were excluded from the analysis.
Replication	All flow experiments contained a single reactor where the conditions between experiments were kept the same. Reproducibility was verified by comparing the outcome of these reactors between devices and experiments.
Randomization	Randomization was not necessary for our study. Stochasticity did not play a predominant role in our experiments.
Blinding	Blinding was not relevant in our work as the data analysis was performed by software and not by human interpretation.

Reporting for specific materials, systems and methods

We require information from authors about some types of materials, experimental systems and methods used in many studies. Here, indicate whether each material, system or method listed is relevant to your study. If you are not sure if a list item applies to your research, read the appropriate section before selecting a response.

Materials & experimental systems

n/a	Involved in the study	
☒	☐	Antibodies
☒	☐	Eukaryotic cell lines
☒	☐	Palaeontology and archaeology
☒	☐	Animals and other organisms
☒	☐	Human research participants
☒	☐	Clinical data
☒	☐	Dual use research of concern

Methods

n/a	Involved in the study	
☒	☐	ChIP-seq
☒	☐	Flow cytometry
☒	☐	MRI-based neuroimaging
