# Supplementary material for: A microfluidic optimal experimental design platform for forward design of cell-free genetic networks
Source: Nat Commun. 2022 Jun 24;13:3626. doi: 10.1038/s41467-022-31306-3 (PMC9232554; doi:10.1038/s41467-022-31306-3)
Supplement: Supplementary file 3 — Description of Additional Supplementary Files [file 41467_2022_31306_MOESM3_ESM.pdf]

Title: Supplementary Data 1:
Description: Derived parameter sets. Parameter sets used for the distributions and/or predictions grouped per fitting round. Which experiments are used to retrieve the parameter sets are specified in the sheet title.

Title: Supplementary Data 2:
Description: Pulse decoder predictions. Plots showing all 150 individual fits used to create the prediction cloud shown in figure 4c of the main text.

Title: Supplementary Data 3:
Description: Bistable switch predictions. Plots showing all 150 individual fits used to create the prediction cloud shown in figure 5c of the main text.
