# Supplementary figures and images for: A microfluidic optimal experimental design platform for forward design of cell-free genetic networks (part 2 of 4)
Source: Nat Commun. 2022 Jun 24;13:3626. doi: 10.1038/s41467-022-31306-3 (PMC9232554; doi:10.1038/s41467-022-31306-3)

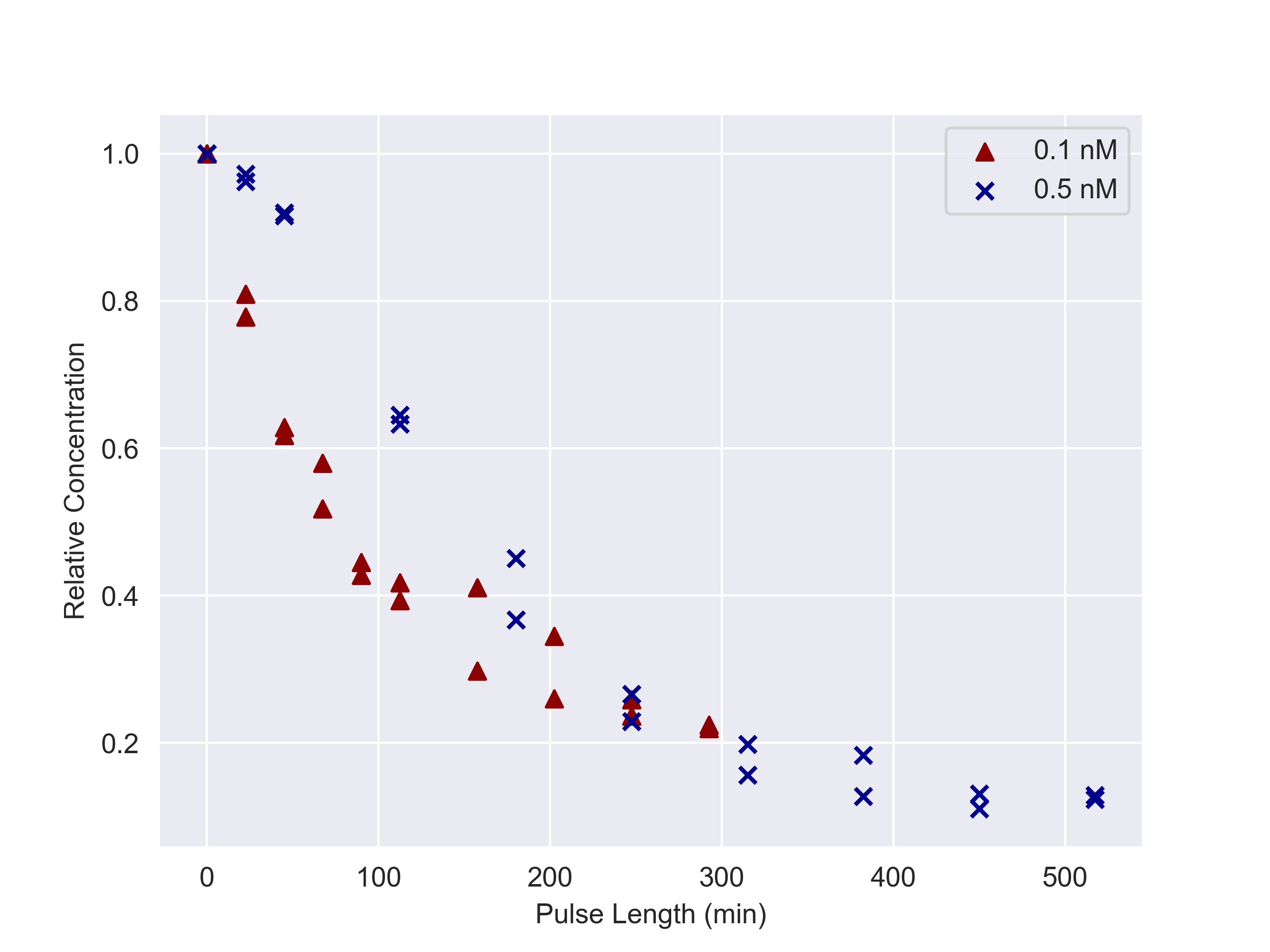

Supplement: Supplementary file 5 — Supplementary Dataset 2 [file 41467_2022_31306_MOESM5_ESM.zip › Individual Simulations Pulse Decoder/53.png]

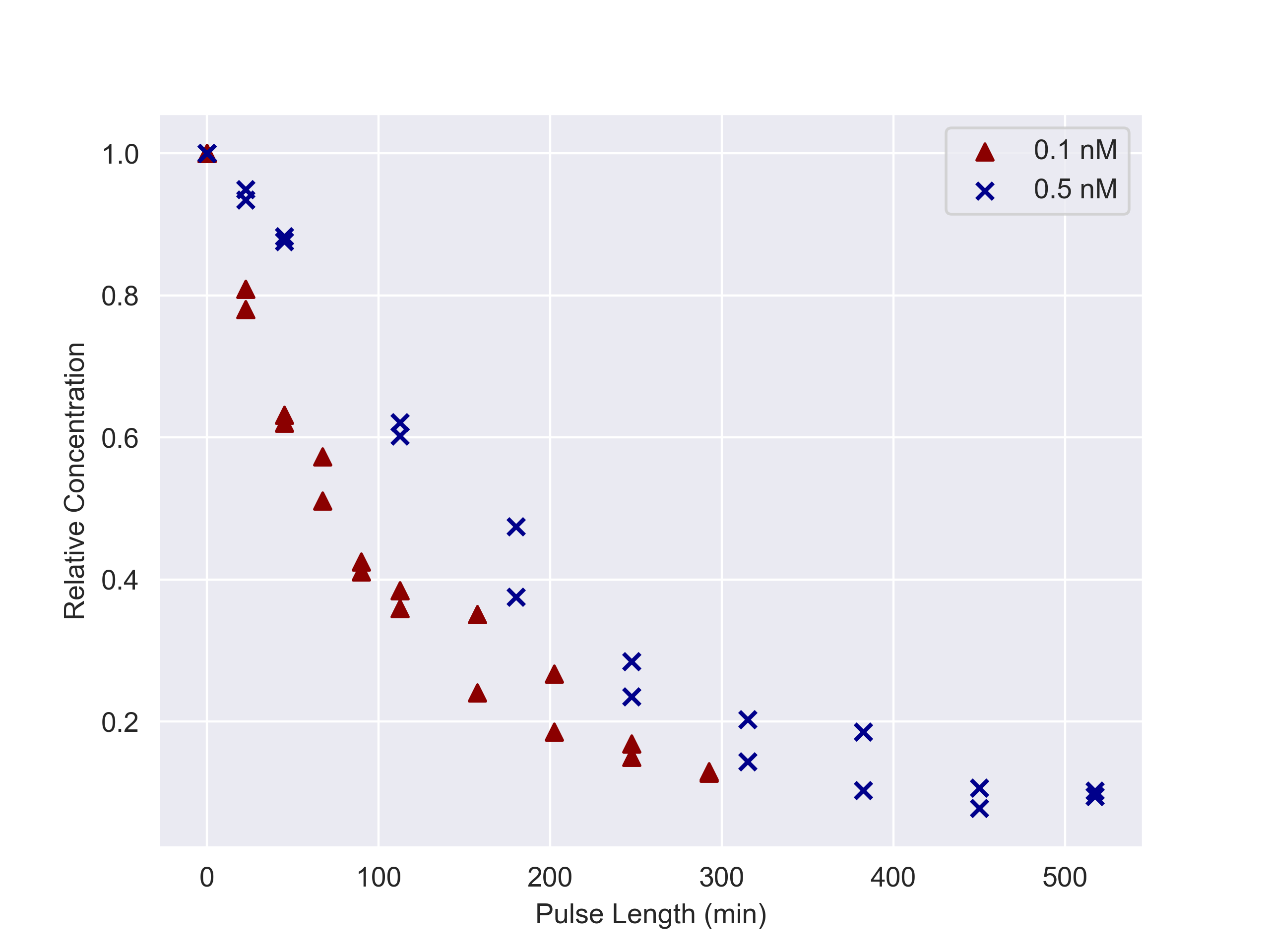

Supplement: Supplementary file 5 — Supplementary Dataset 2 [file 41467_2022_31306_MOESM5_ESM.zip › Individual Simulations Pulse Decoder/54.png]

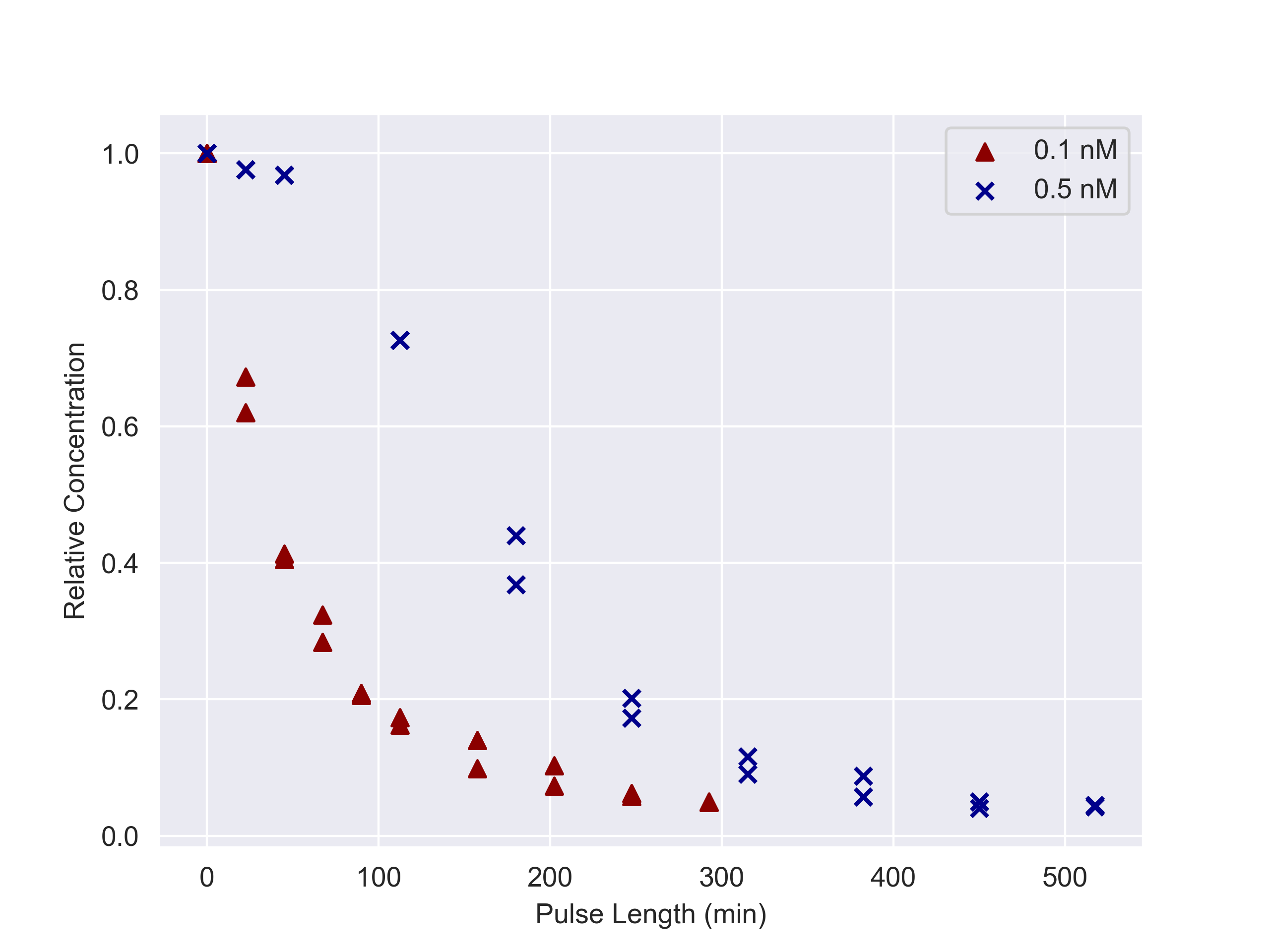

Supplement: Supplementary file 5 — Supplementary Dataset 2 [file 41467_2022_31306_MOESM5_ESM.zip › Individual Simulations Pulse Decoder/55.png]

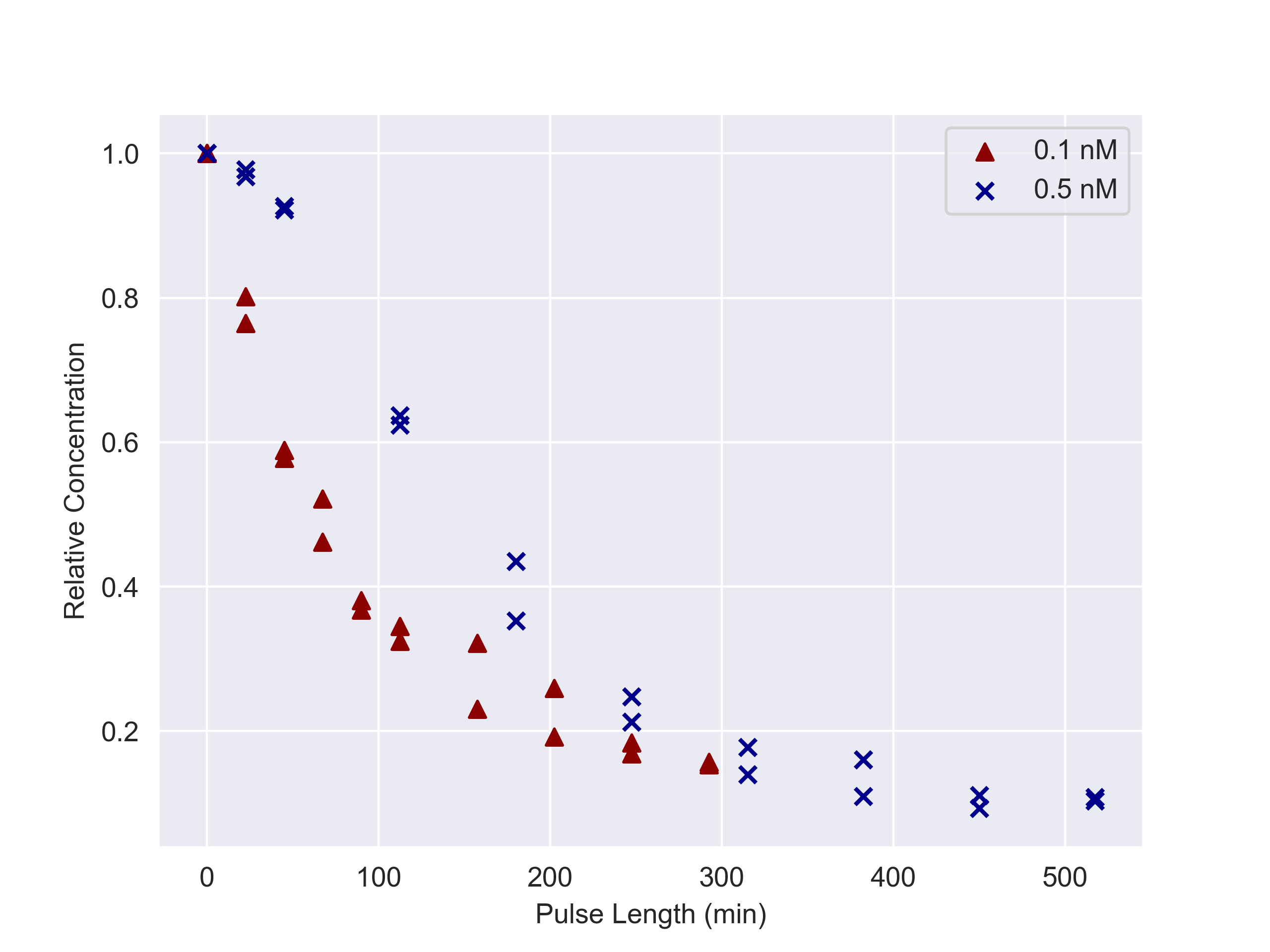

Supplement: Supplementary file 5 — Supplementary Dataset 2 [file 41467_2022_31306_MOESM5_ESM.zip › Individual Simulations Pulse Decoder/56.png]

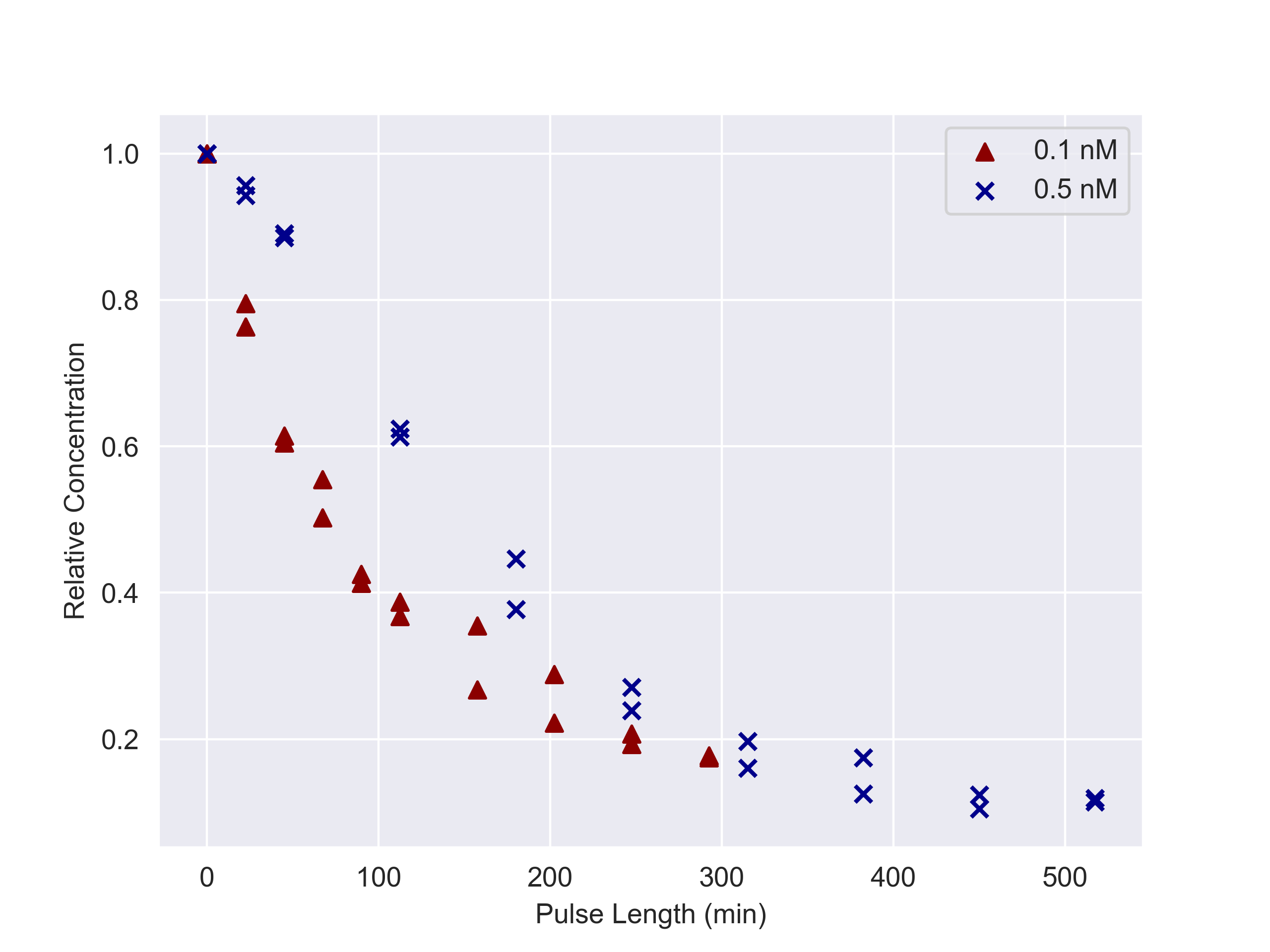

Supplement: Supplementary file 5 — Supplementary Dataset 2 [file 41467_2022_31306_MOESM5_ESM.zip › Individual Simulations Pulse Decoder/57.png]

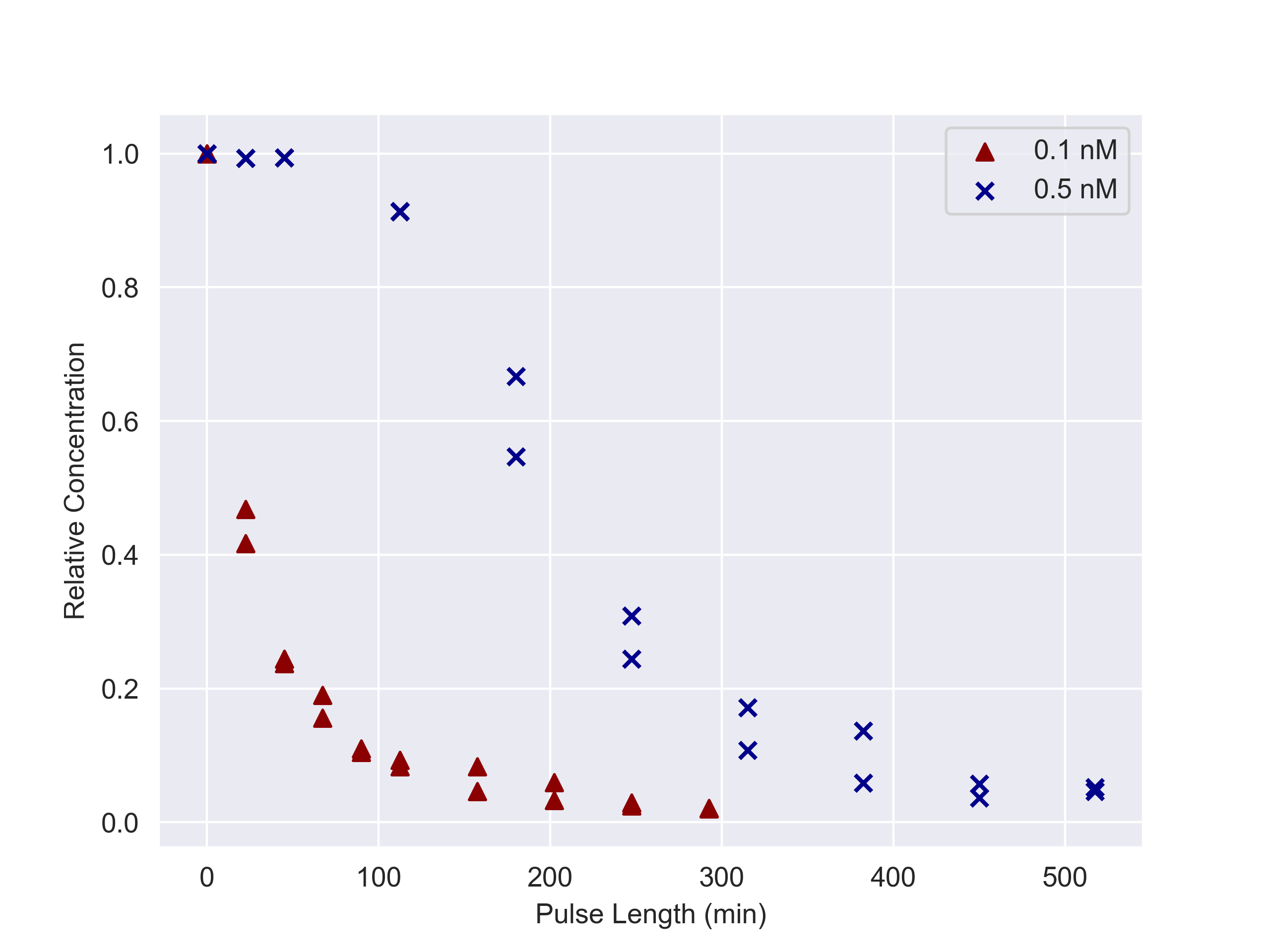

Supplement: Supplementary file 5 — Supplementary Dataset 2 [file 41467_2022_31306_MOESM5_ESM.zip › Individual Simulations Pulse Decoder/58.png]

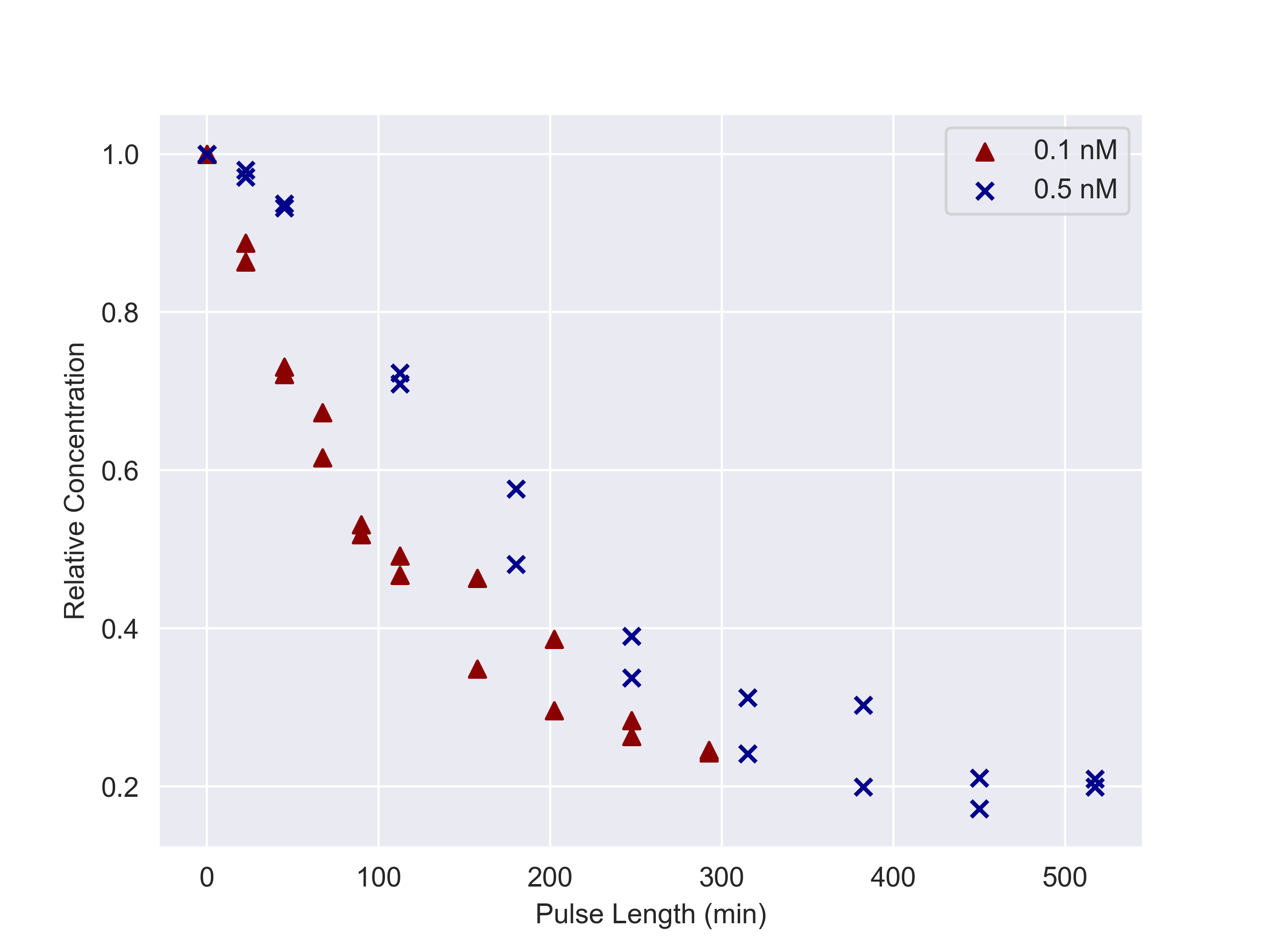

Supplement: Supplementary file 5 — Supplementary Dataset 2 [file 41467_2022_31306_MOESM5_ESM.zip › Individual Simulations Pulse Decoder/59.png]

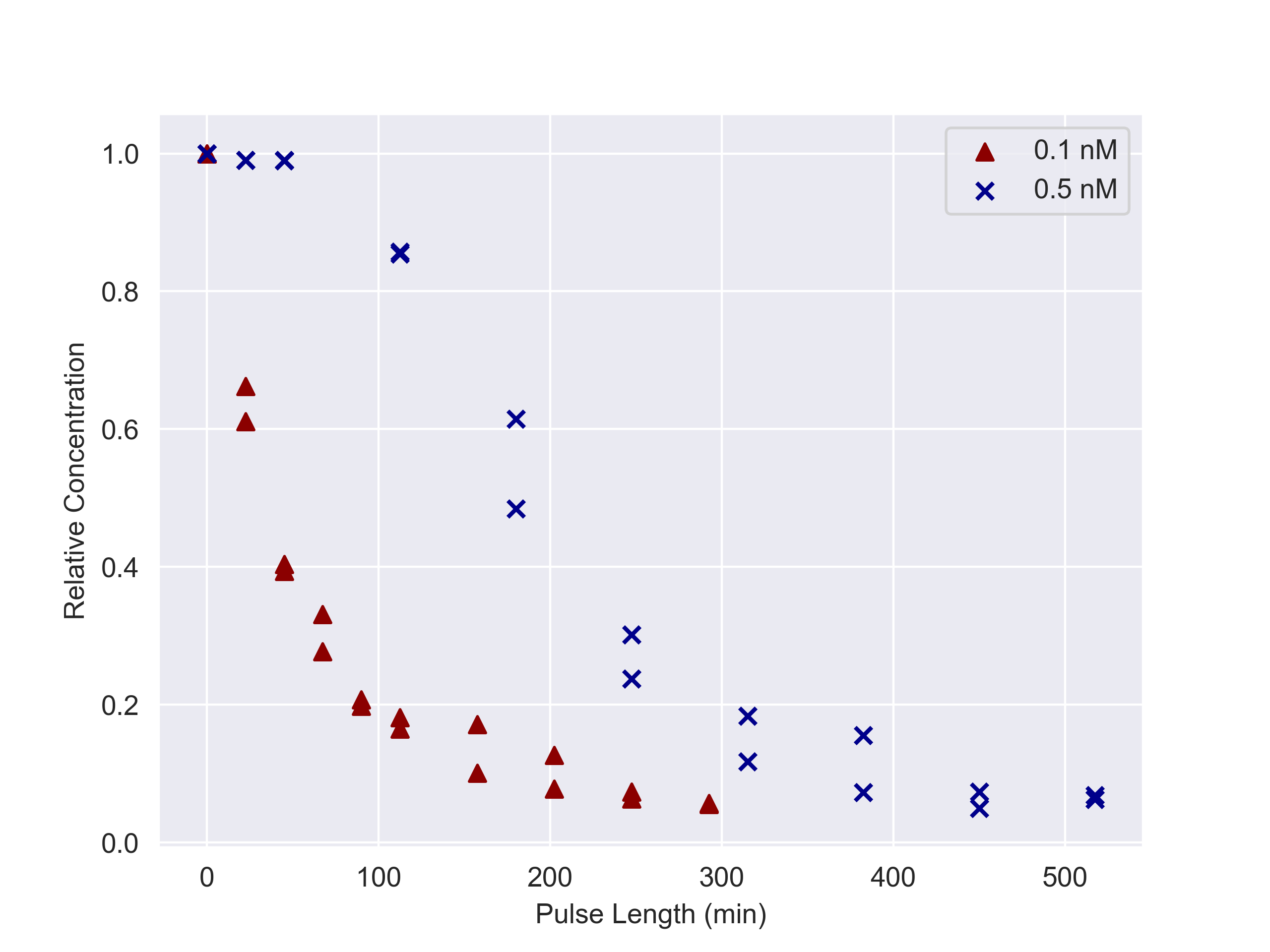

Supplement: Supplementary file 5 — Supplementary Dataset 2 [file 41467_2022_31306_MOESM5_ESM.zip › Individual Simulations Pulse Decoder/6.png]

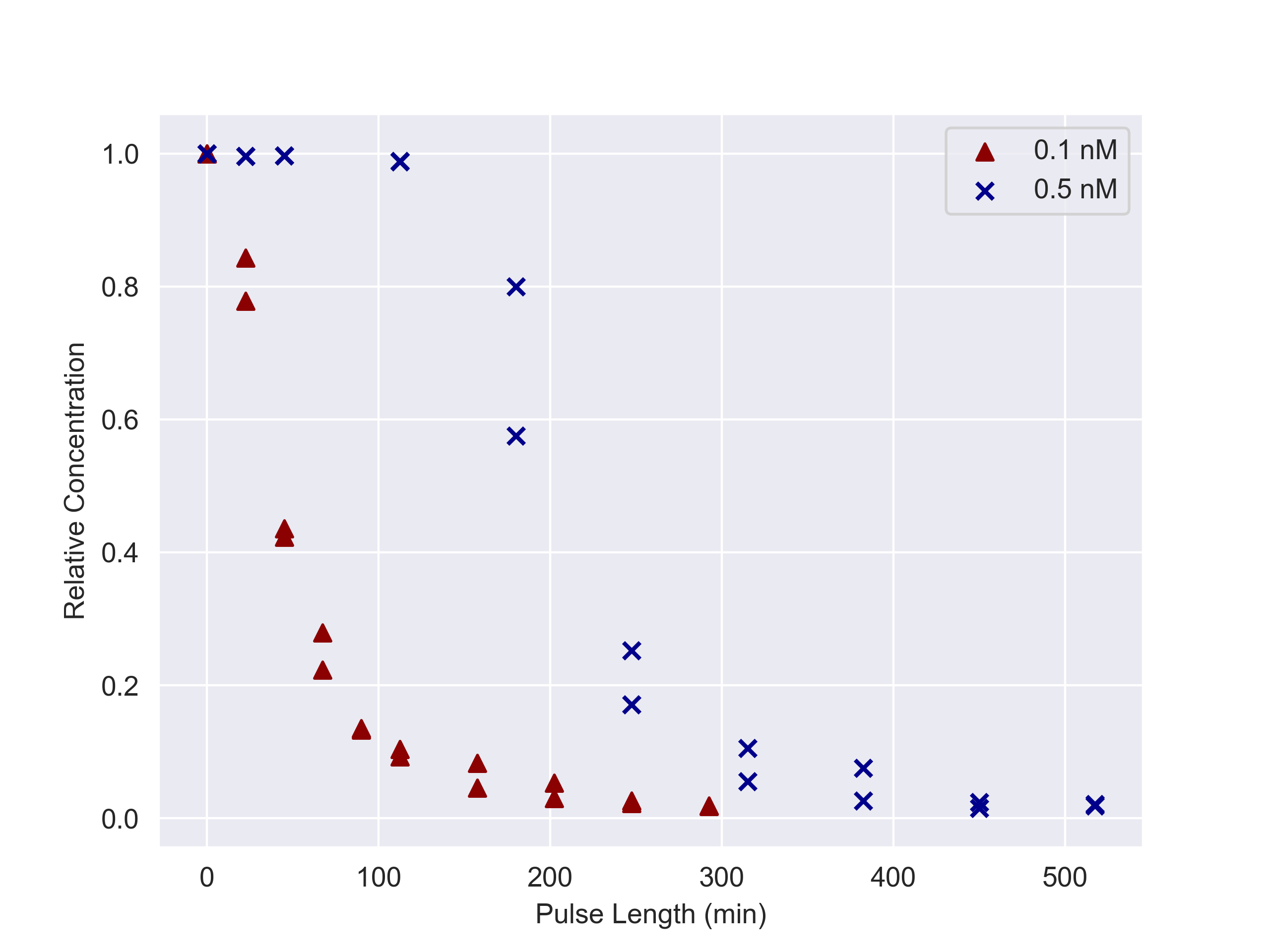

Supplement: Supplementary file 5 — Supplementary Dataset 2 [file 41467_2022_31306_MOESM5_ESM.zip › Individual Simulations Pulse Decoder/60.png]

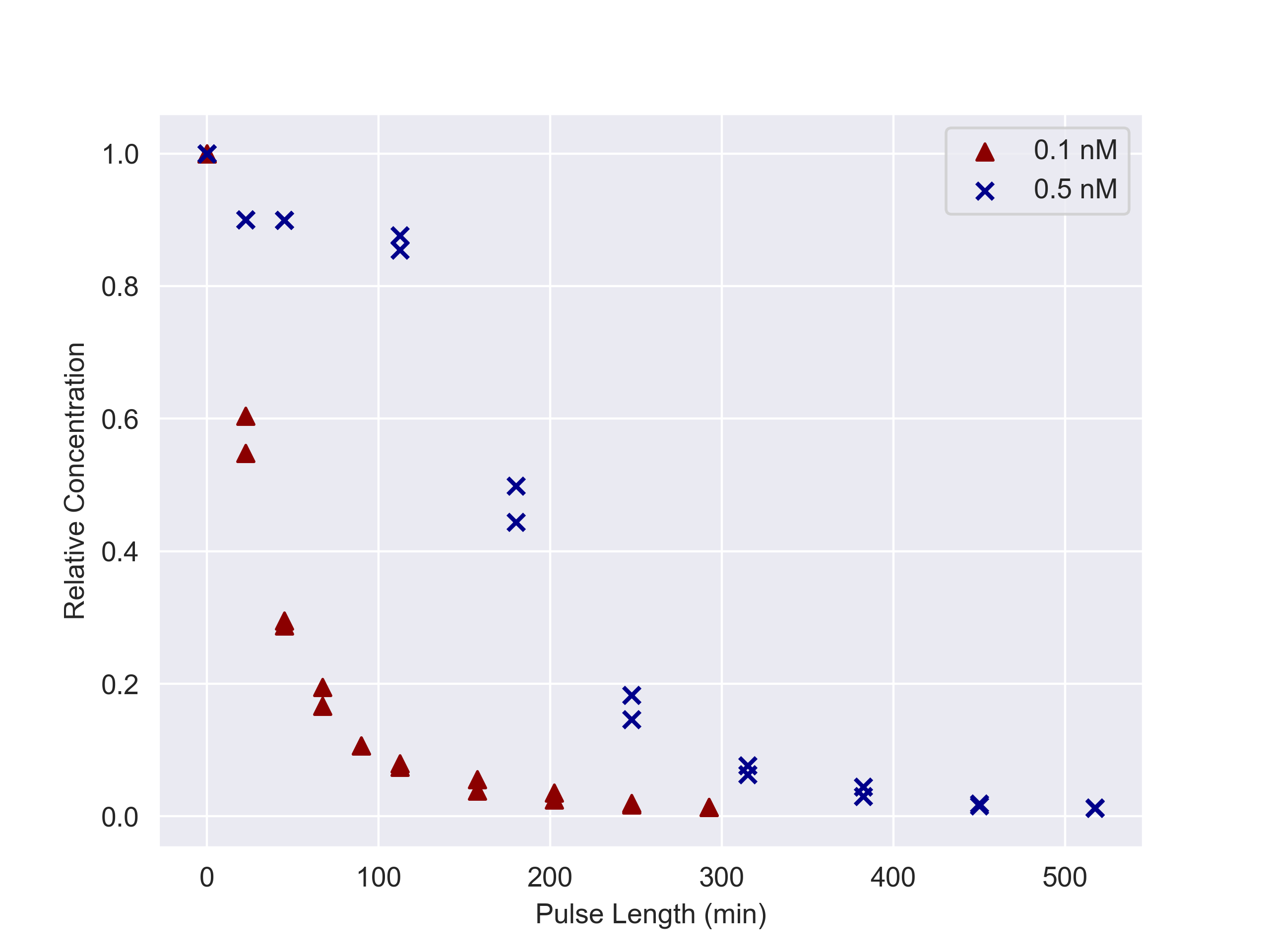

Supplement: Supplementary file 5 — Supplementary Dataset 2 [file 41467_2022_31306_MOESM5_ESM.zip › Individual Simulations Pulse Decoder/61.png]

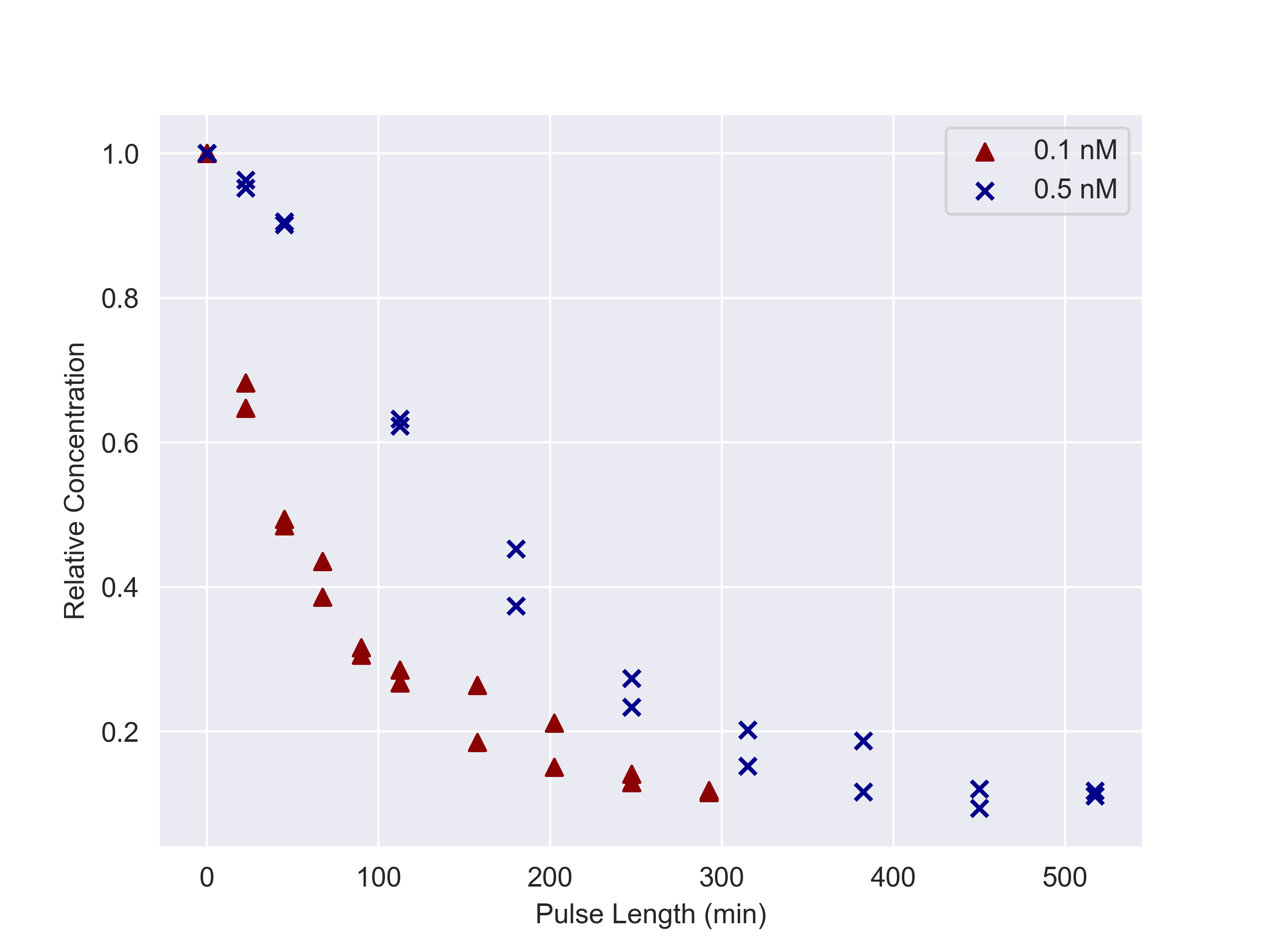

Supplement: Supplementary file 5 — Supplementary Dataset 2 [file 41467_2022_31306_MOESM5_ESM.zip › Individual Simulations Pulse Decoder/62.png]

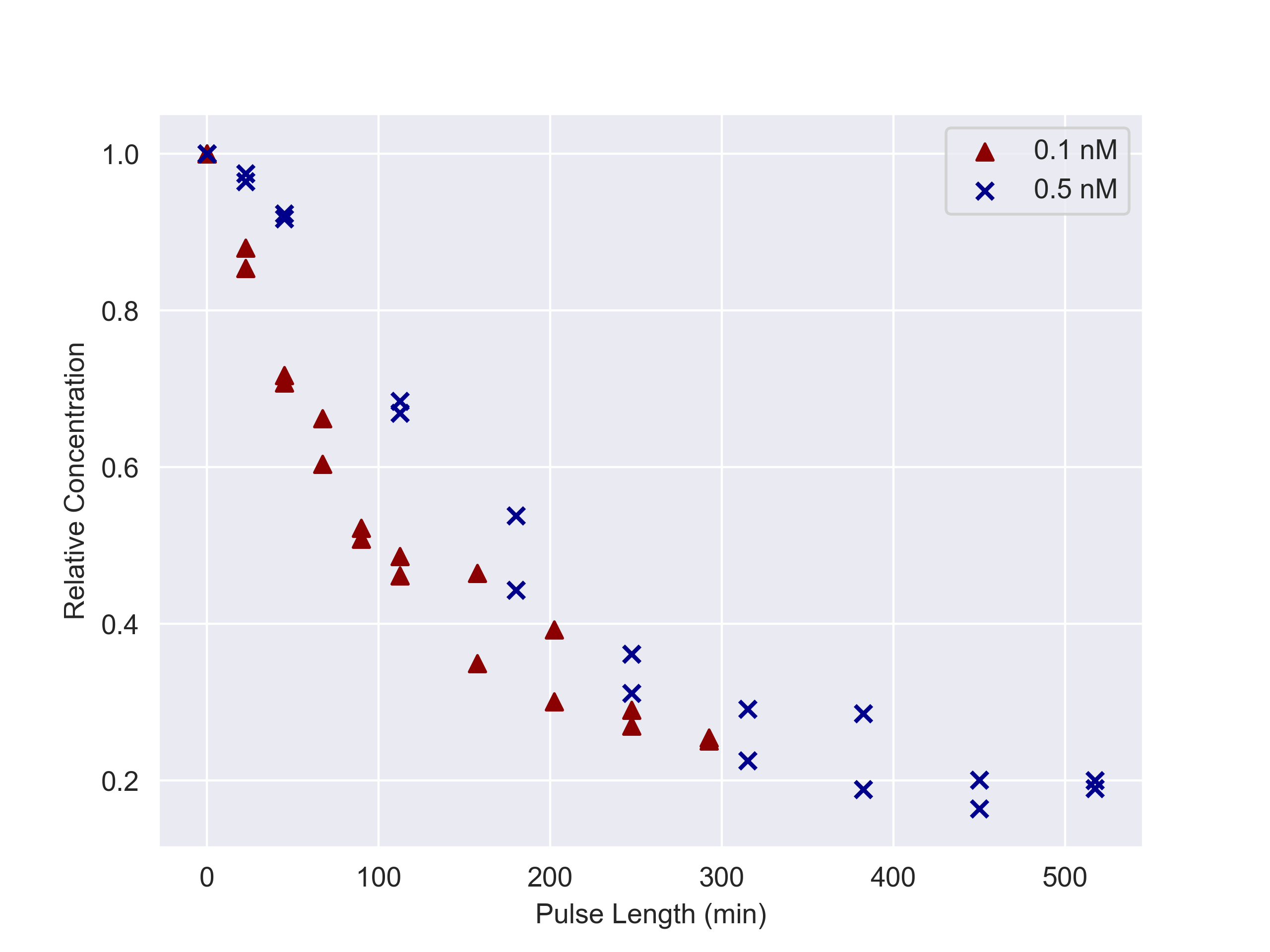

Supplement: Supplementary file 5 — Supplementary Dataset 2 [file 41467_2022_31306_MOESM5_ESM.zip › Individual Simulations Pulse Decoder/63.png]

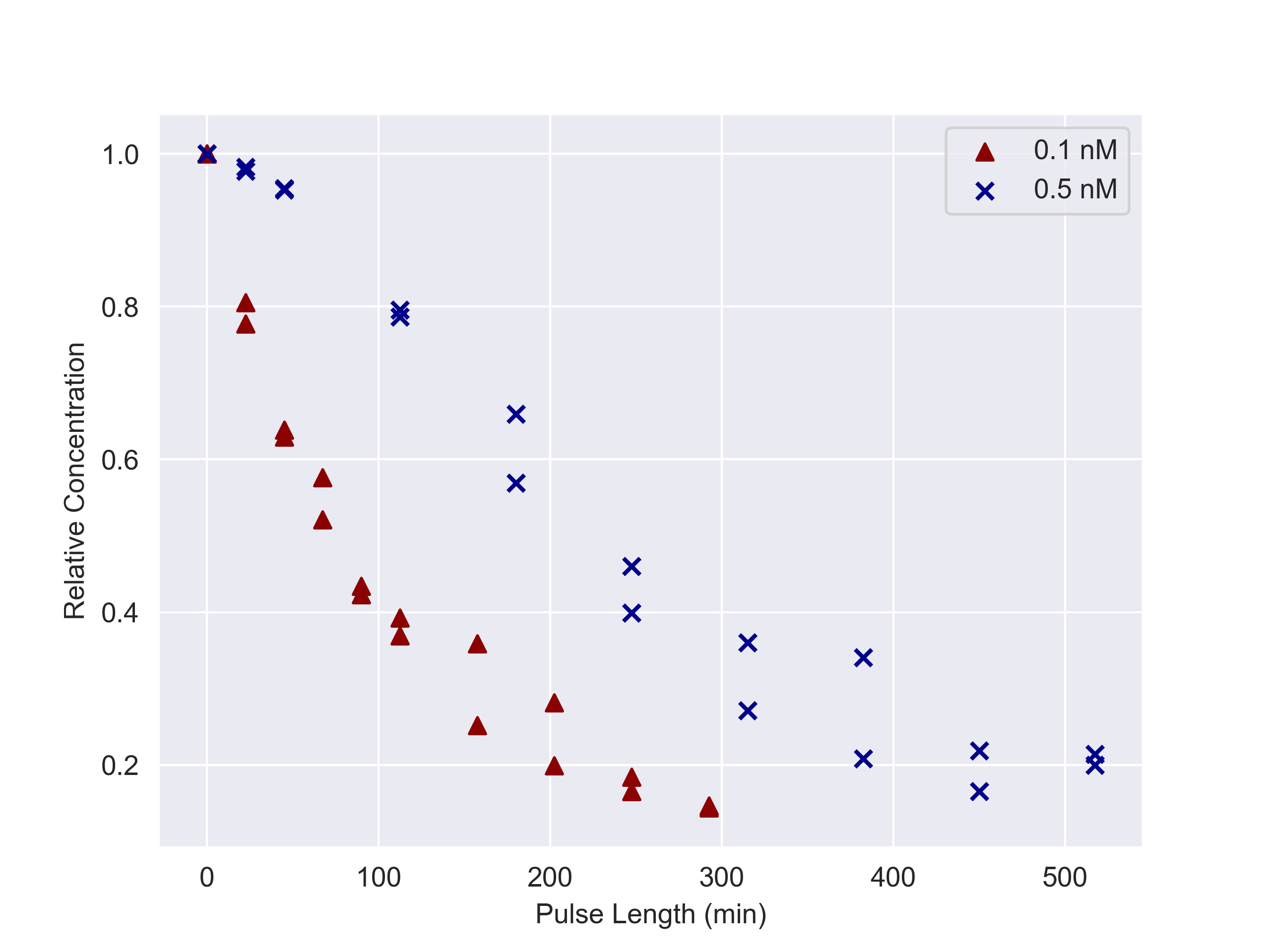

Supplement: Supplementary file 5 — Supplementary Dataset 2 [file 41467_2022_31306_MOESM5_ESM.zip › Individual Simulations Pulse Decoder/64.png]

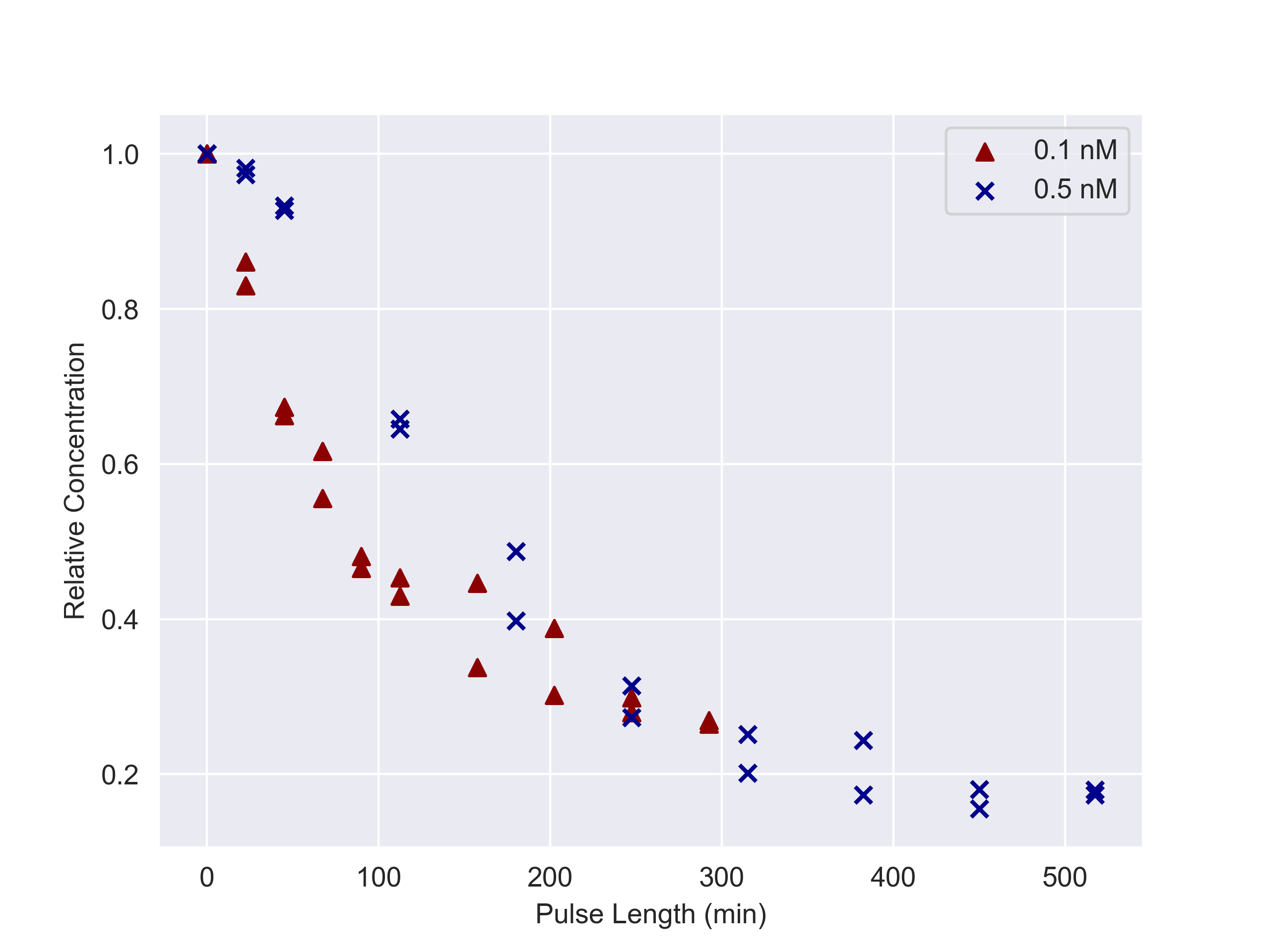

Supplement: Supplementary file 5 — Supplementary Dataset 2 [file 41467_2022_31306_MOESM5_ESM.zip › Individual Simulations Pulse Decoder/65.png]

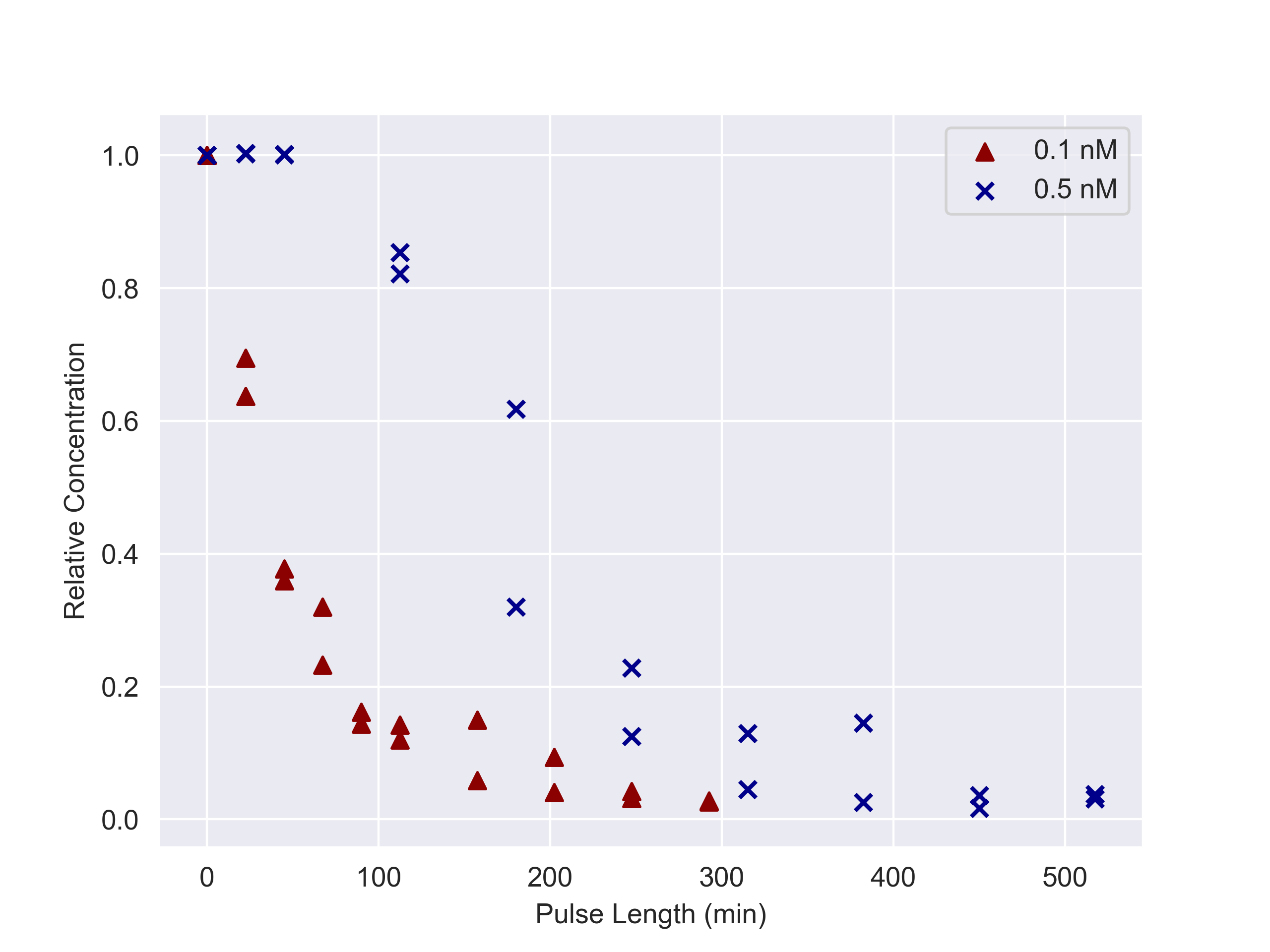

Supplement: Supplementary file 5 — Supplementary Dataset 2 [file 41467_2022_31306_MOESM5_ESM.zip › Individual Simulations Pulse Decoder/66.png]

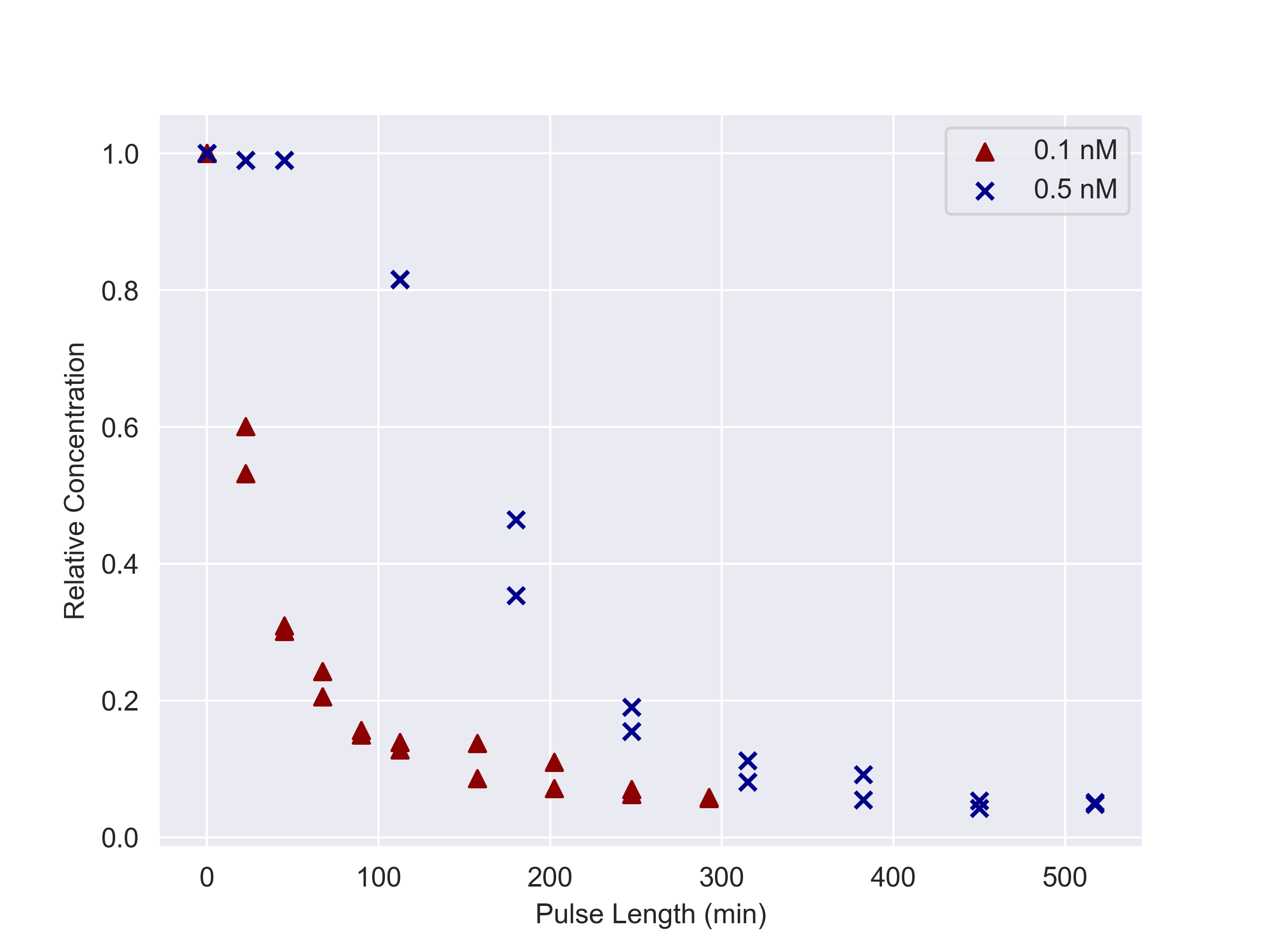

Supplement: Supplementary file 5 — Supplementary Dataset 2 [file 41467_2022_31306_MOESM5_ESM.zip › Individual Simulations Pulse Decoder/67.png]

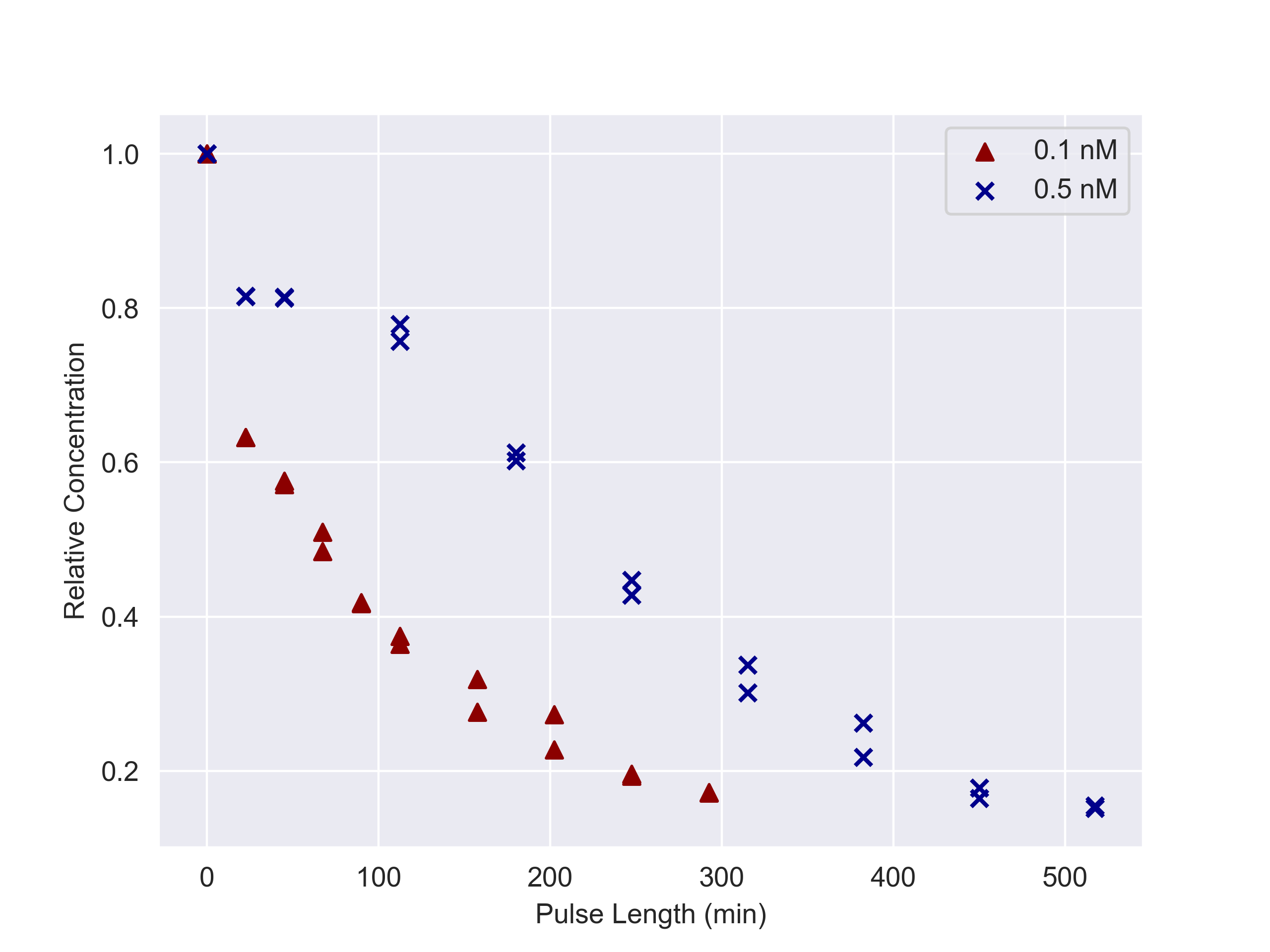

Supplement: Supplementary file 5 — Supplementary Dataset 2 [file 41467_2022_31306_MOESM5_ESM.zip › Individual Simulations Pulse Decoder/68.png]

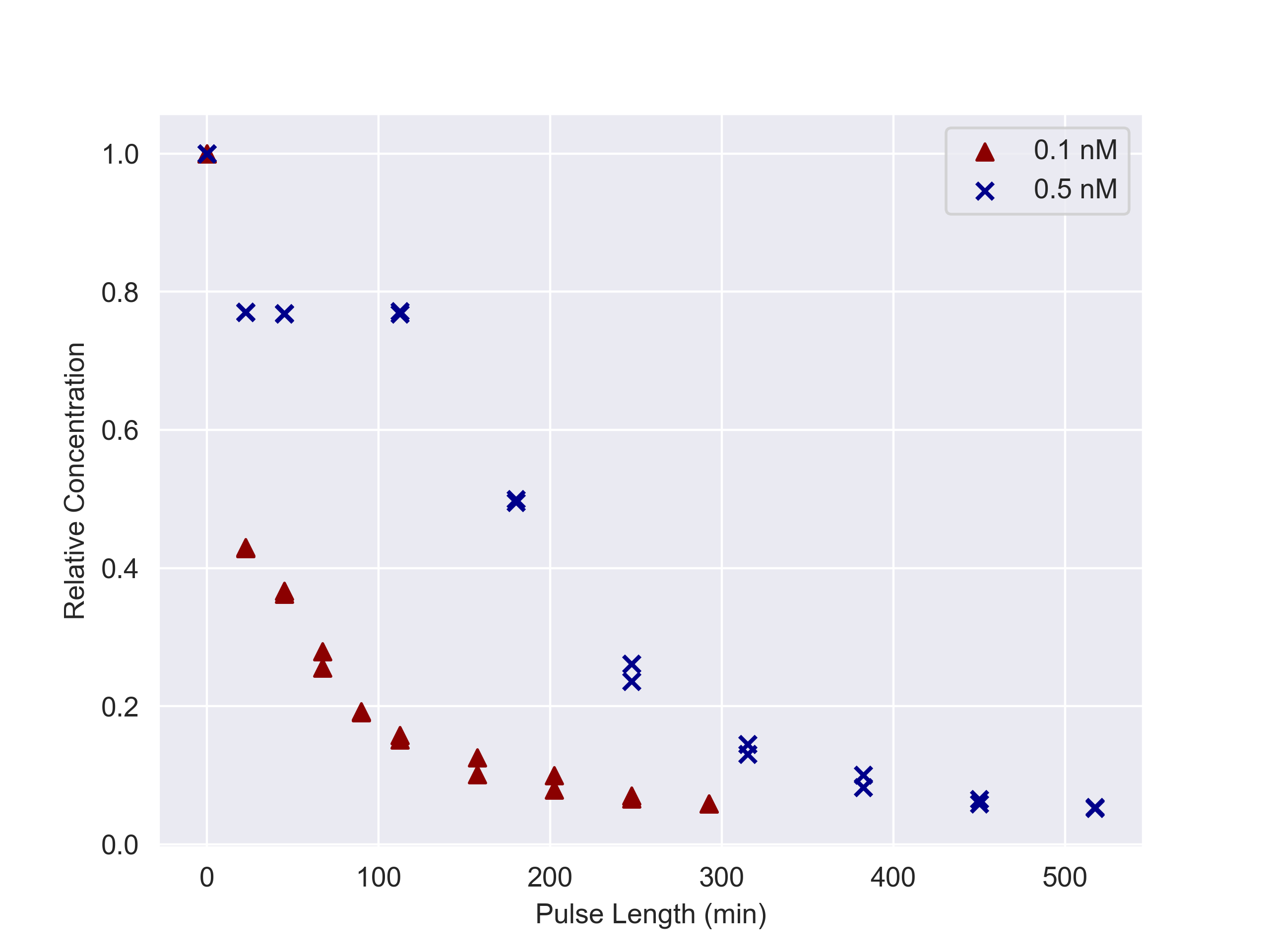

Supplement: Supplementary file 5 — Supplementary Dataset 2 [file 41467_2022_31306_MOESM5_ESM.zip › Individual Simulations Pulse Decoder/69.png]

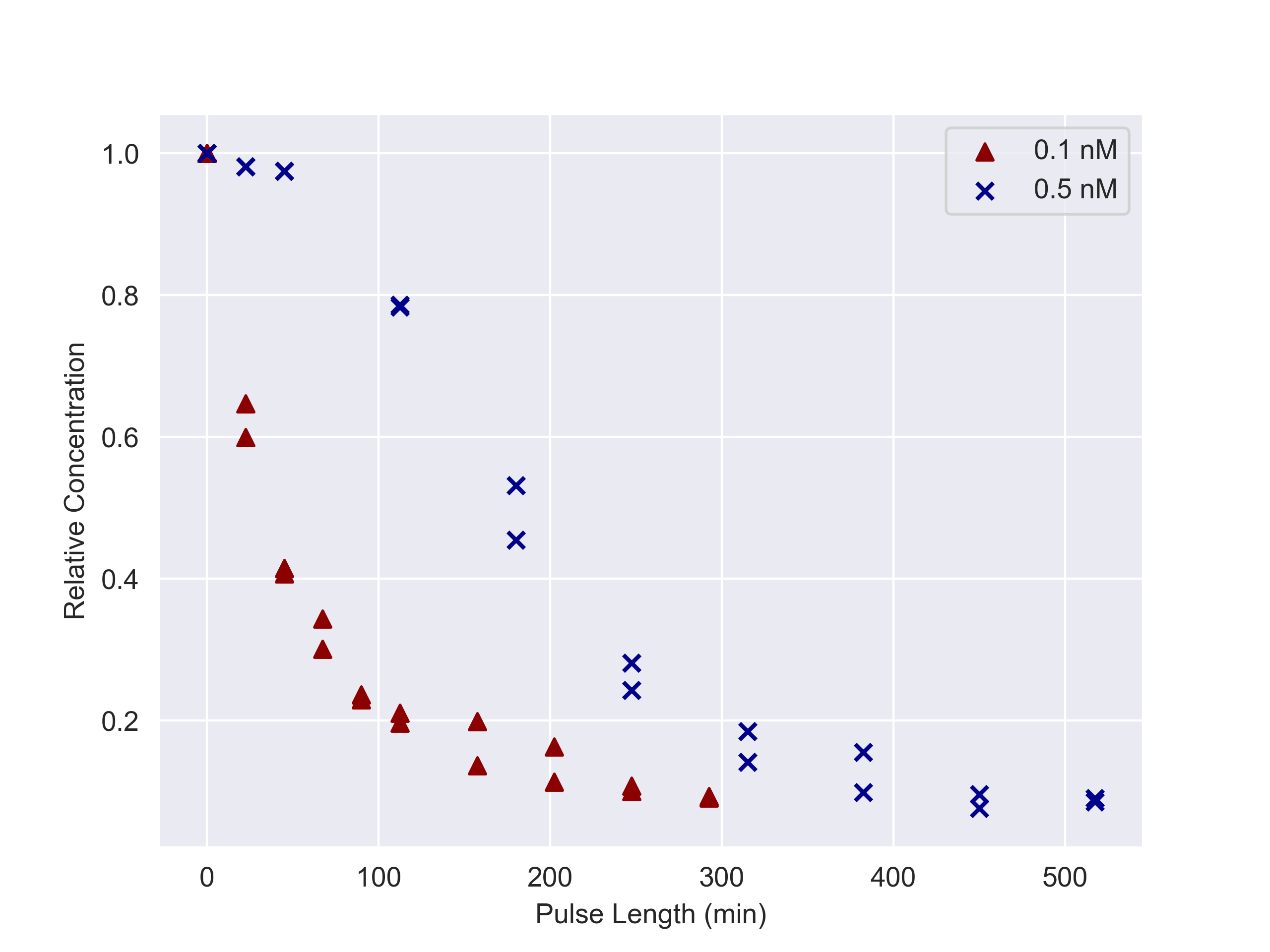

Supplement: Supplementary file 5 — Supplementary Dataset 2 [file 41467_2022_31306_MOESM5_ESM.zip › Individual Simulations Pulse Decoder/7.png]

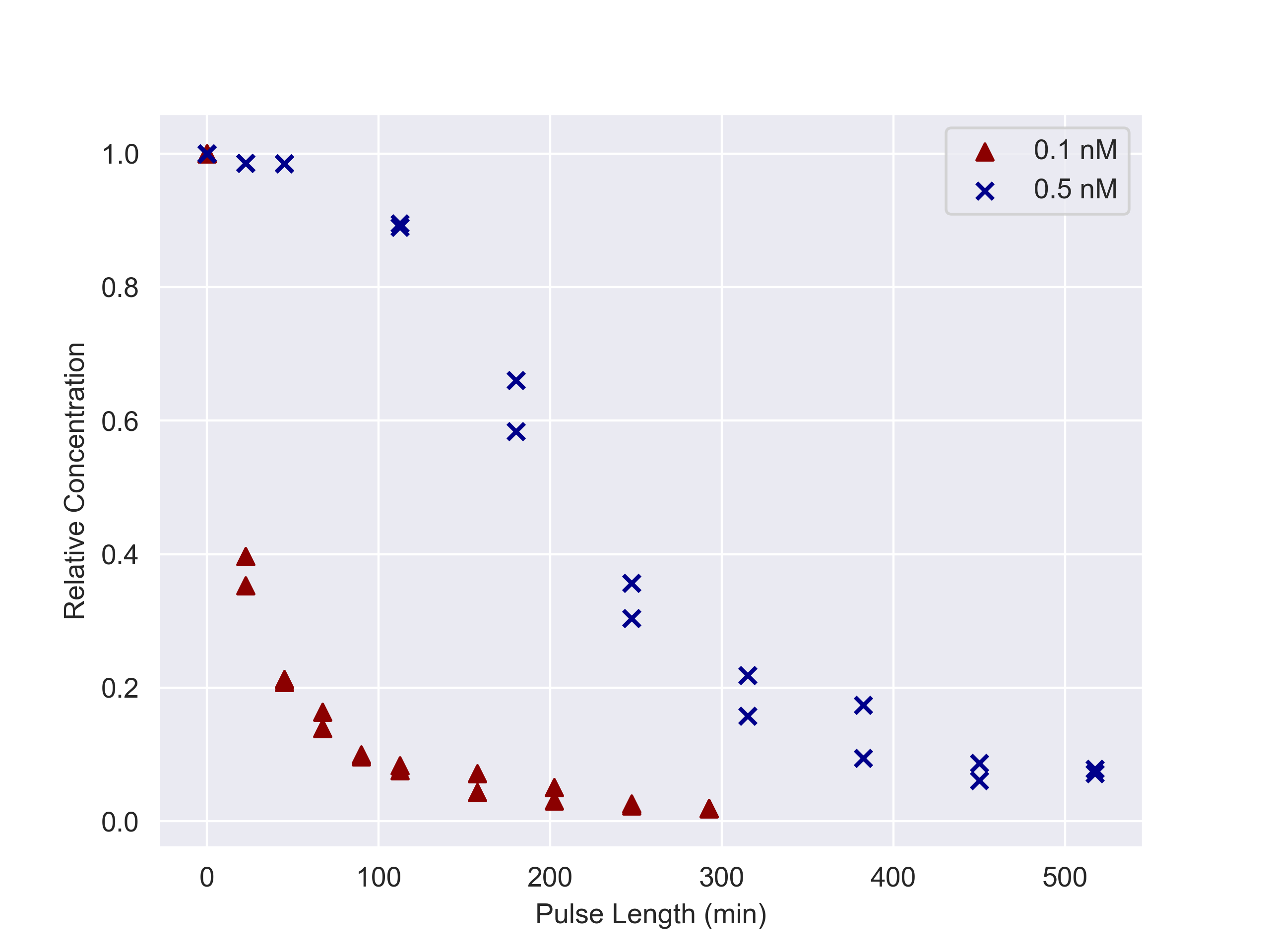

Supplement: Supplementary file 5 — Supplementary Dataset 2 [file 41467_2022_31306_MOESM5_ESM.zip › Individual Simulations Pulse Decoder/70.png]

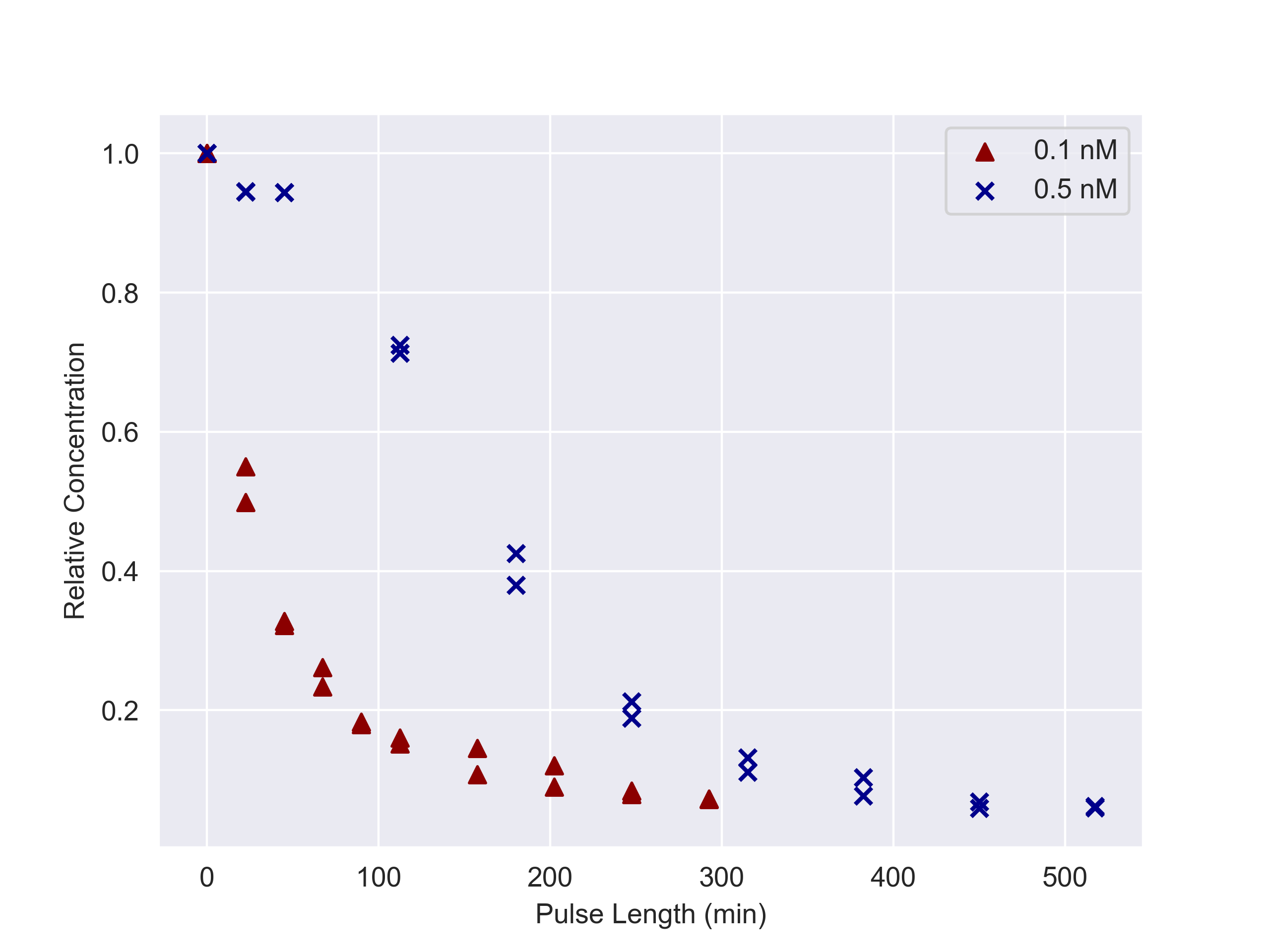

Supplement: Supplementary file 5 — Supplementary Dataset 2 [file 41467_2022_31306_MOESM5_ESM.zip › Individual Simulations Pulse Decoder/71.png]

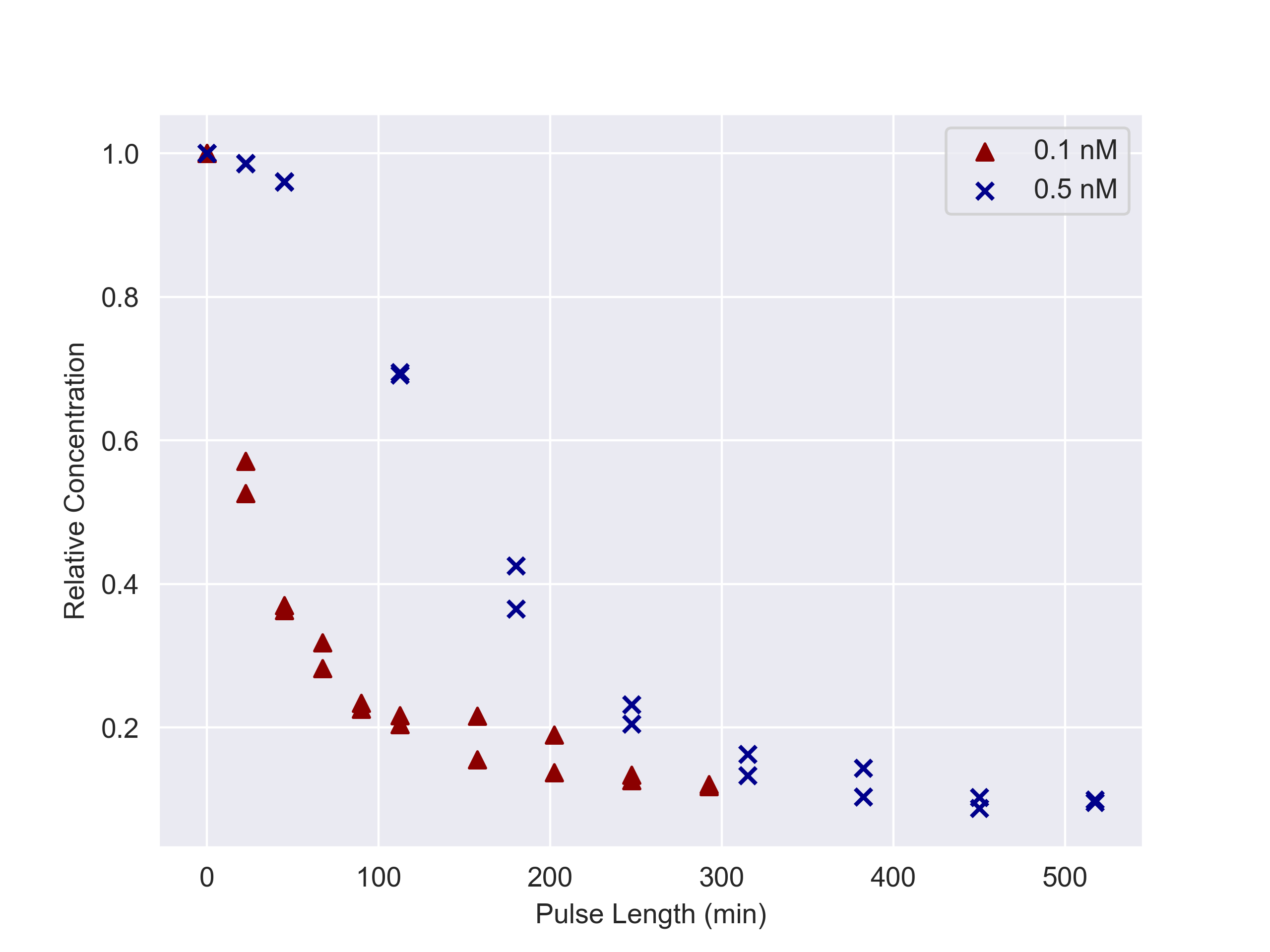

Supplement: Supplementary file 5 — Supplementary Dataset 2 [file 41467_2022_31306_MOESM5_ESM.zip › Individual Simulations Pulse Decoder/72.png]

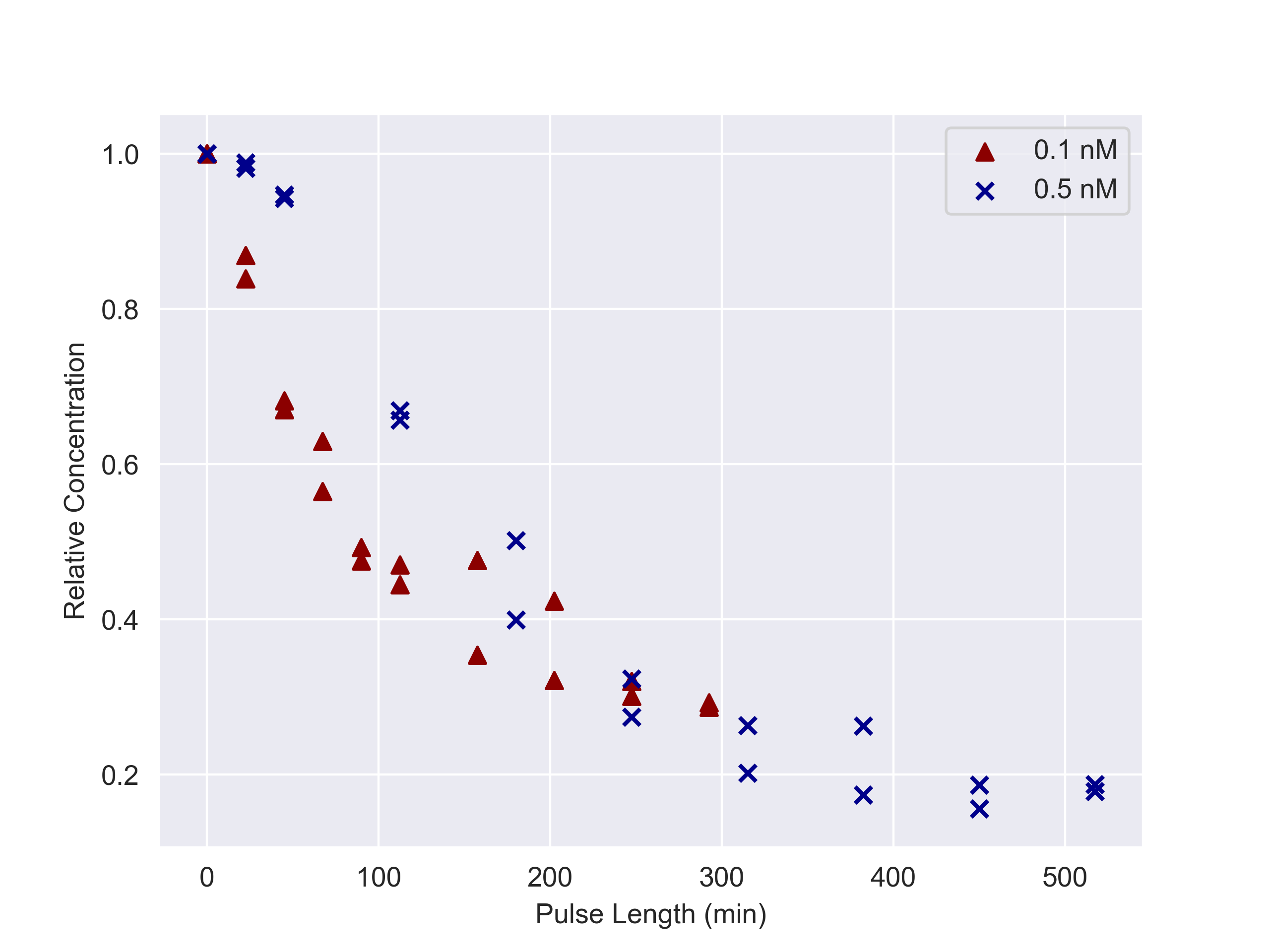

Supplement: Supplementary file 5 — Supplementary Dataset 2 [file 41467_2022_31306_MOESM5_ESM.zip › Individual Simulations Pulse Decoder/73.png]

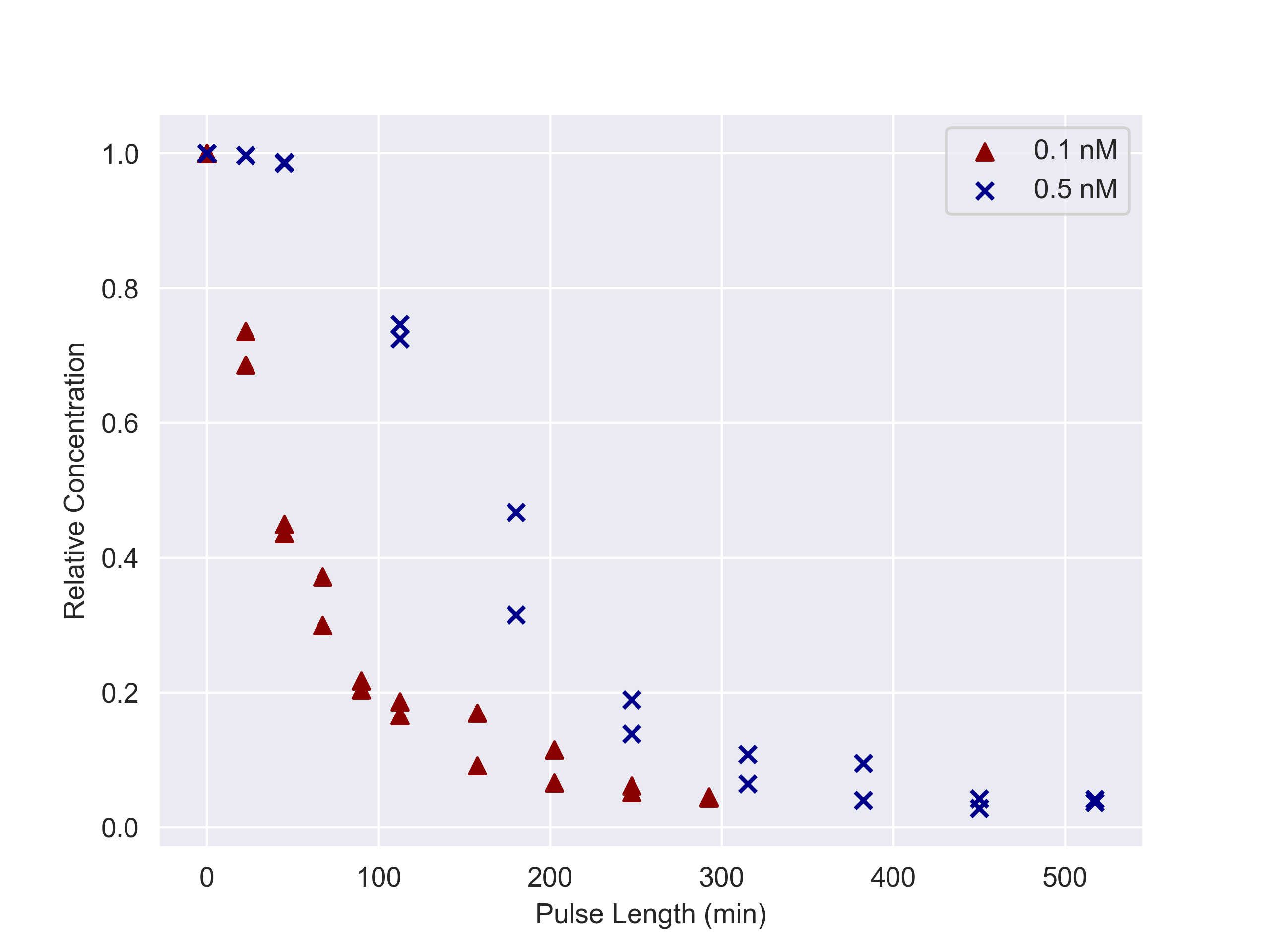

Supplement: Supplementary file 5 — Supplementary Dataset 2 [file 41467_2022_31306_MOESM5_ESM.zip › Individual Simulations Pulse Decoder/74.png]

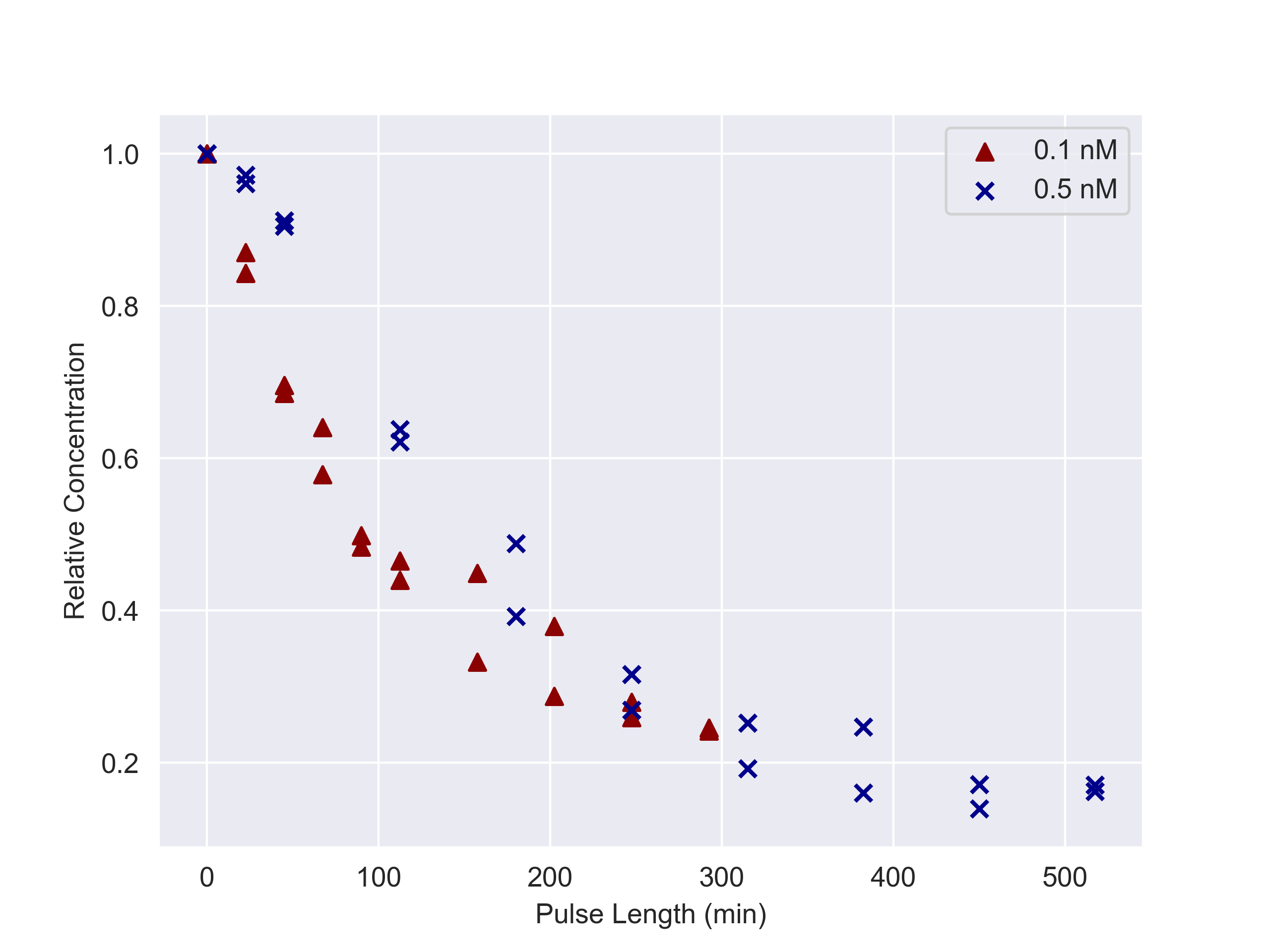

Supplement: Supplementary file 5 — Supplementary Dataset 2 [file 41467_2022_31306_MOESM5_ESM.zip › Individual Simulations Pulse Decoder/75.png]

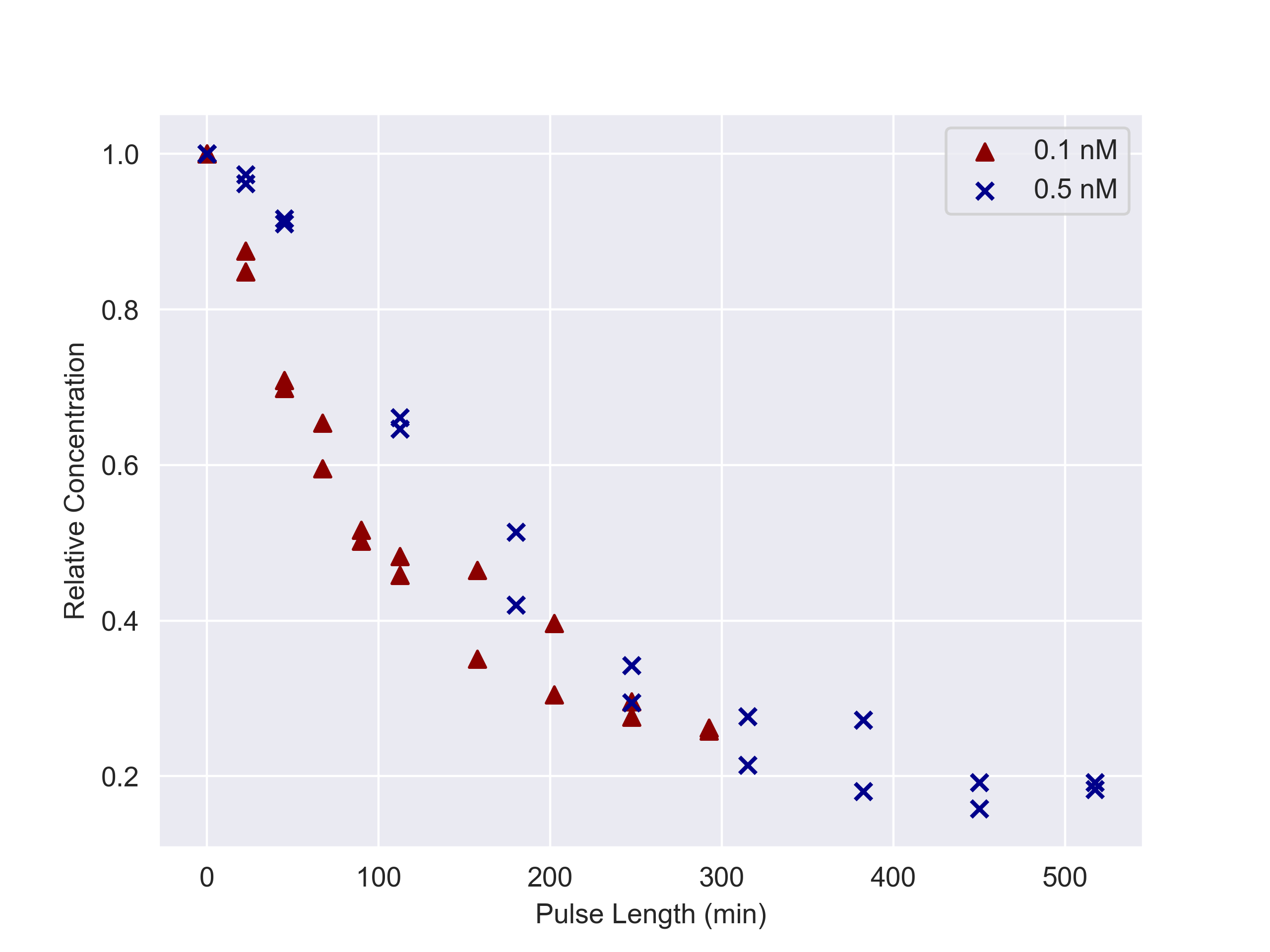

Supplement: Supplementary file 5 — Supplementary Dataset 2 [file 41467_2022_31306_MOESM5_ESM.zip › Individual Simulations Pulse Decoder/76.png]

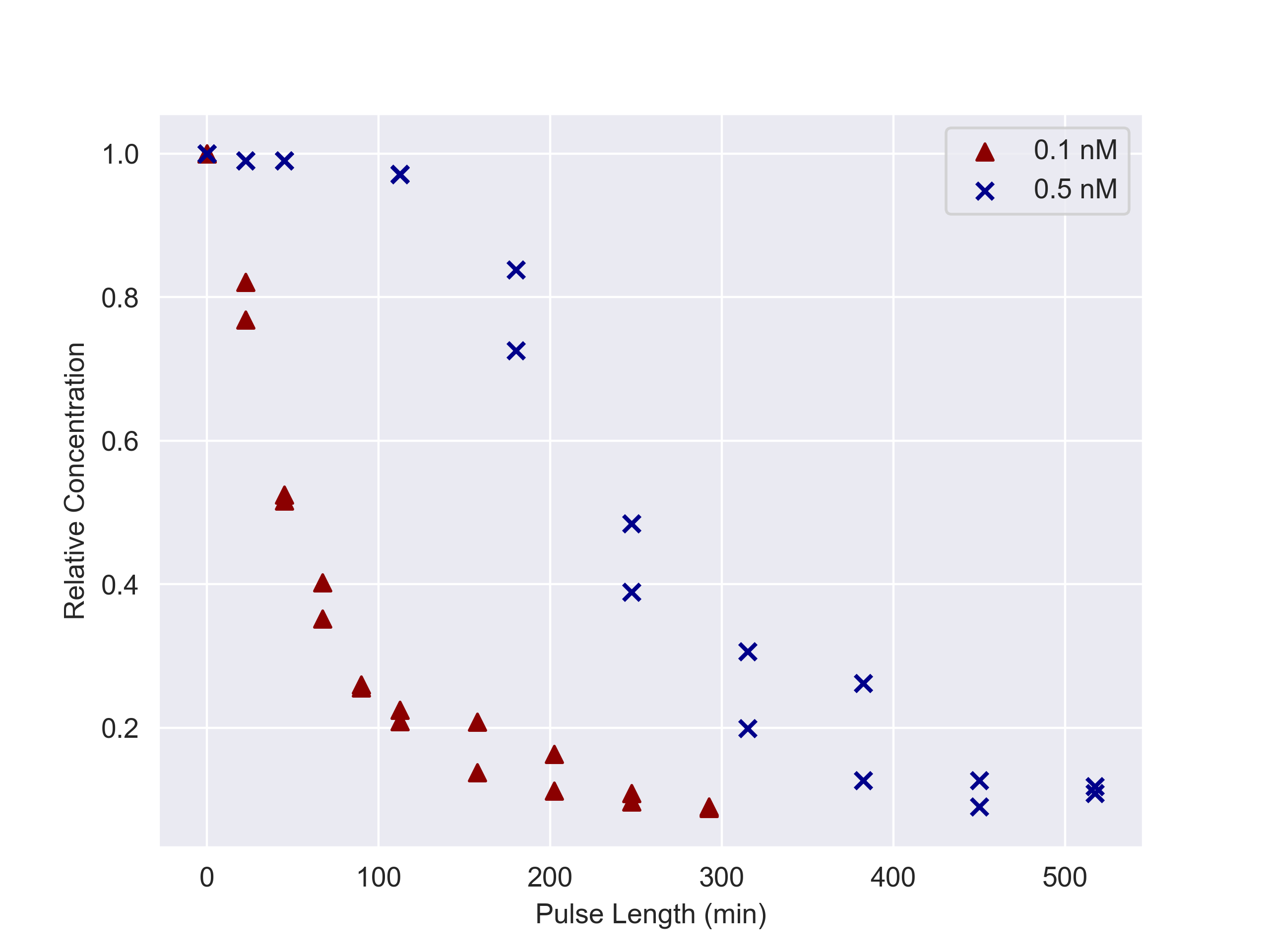

Supplement: Supplementary file 5 — Supplementary Dataset 2 [file 41467_2022_31306_MOESM5_ESM.zip › Individual Simulations Pulse Decoder/77.png]

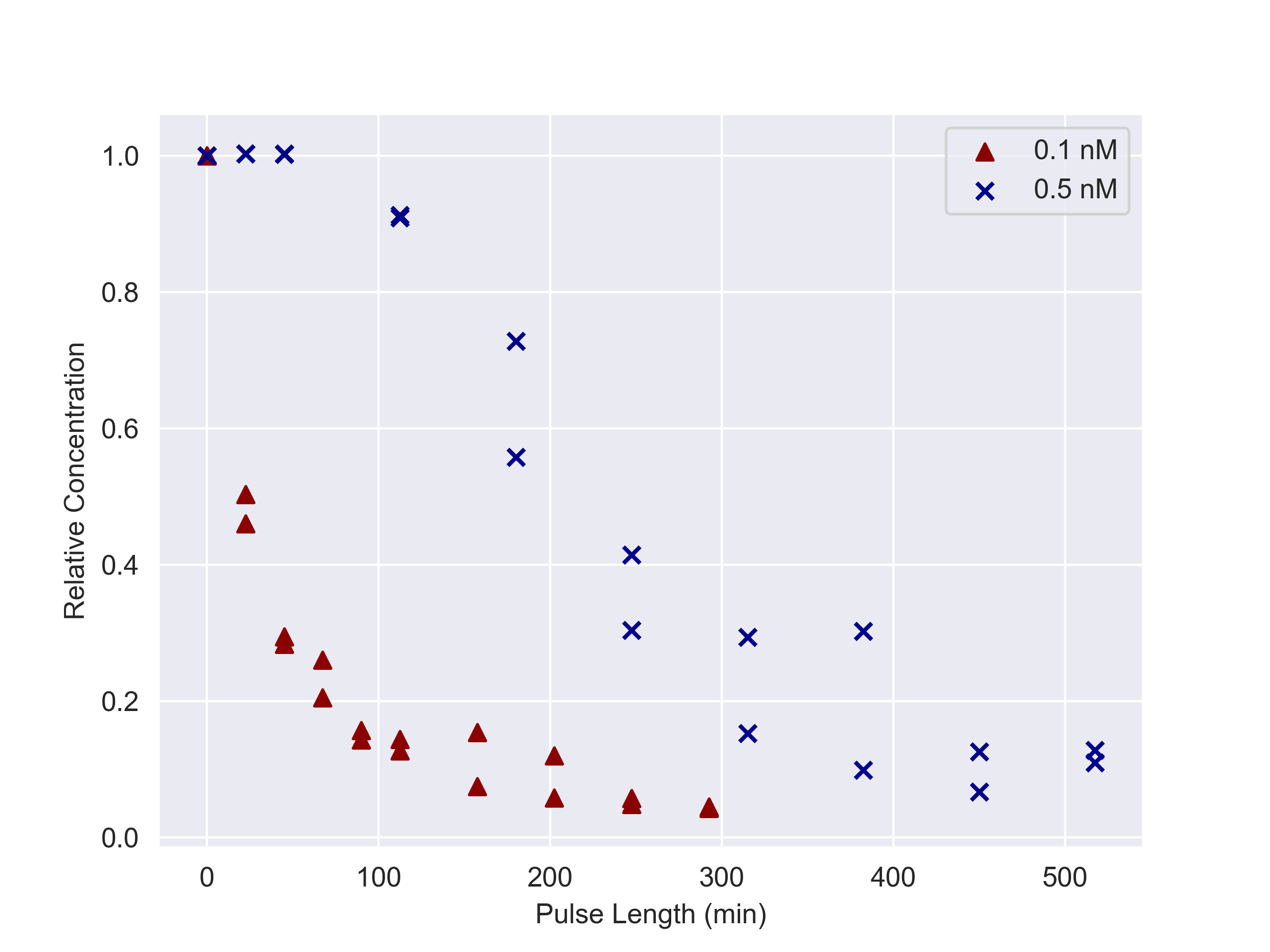

Supplement: Supplementary file 5 — Supplementary Dataset 2 [file 41467_2022_31306_MOESM5_ESM.zip › Individual Simulations Pulse Decoder/78.png]

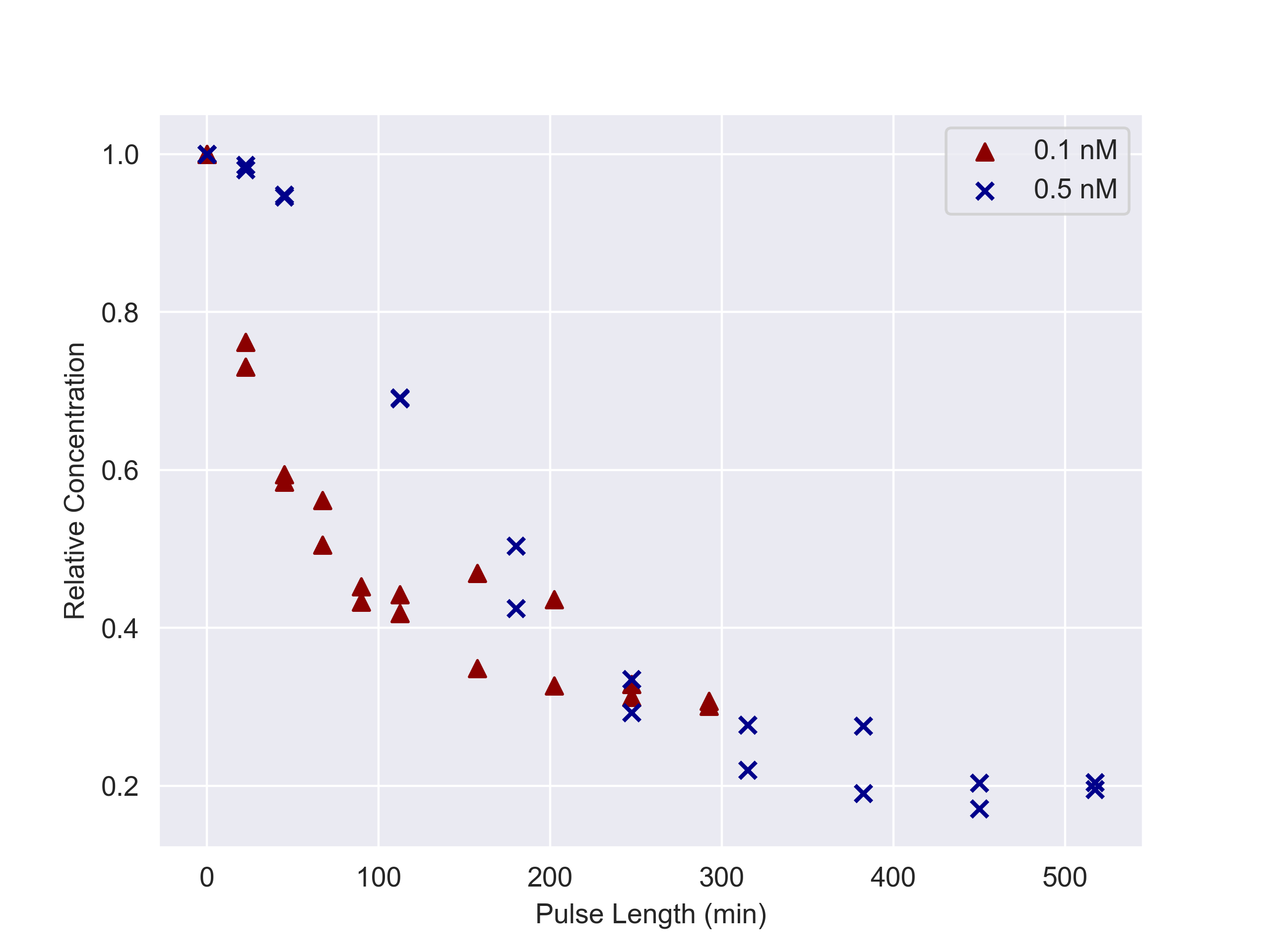

Supplement: Supplementary file 5 — Supplementary Dataset 2 [file 41467_2022_31306_MOESM5_ESM.zip › Individual Simulations Pulse Decoder/79.png]

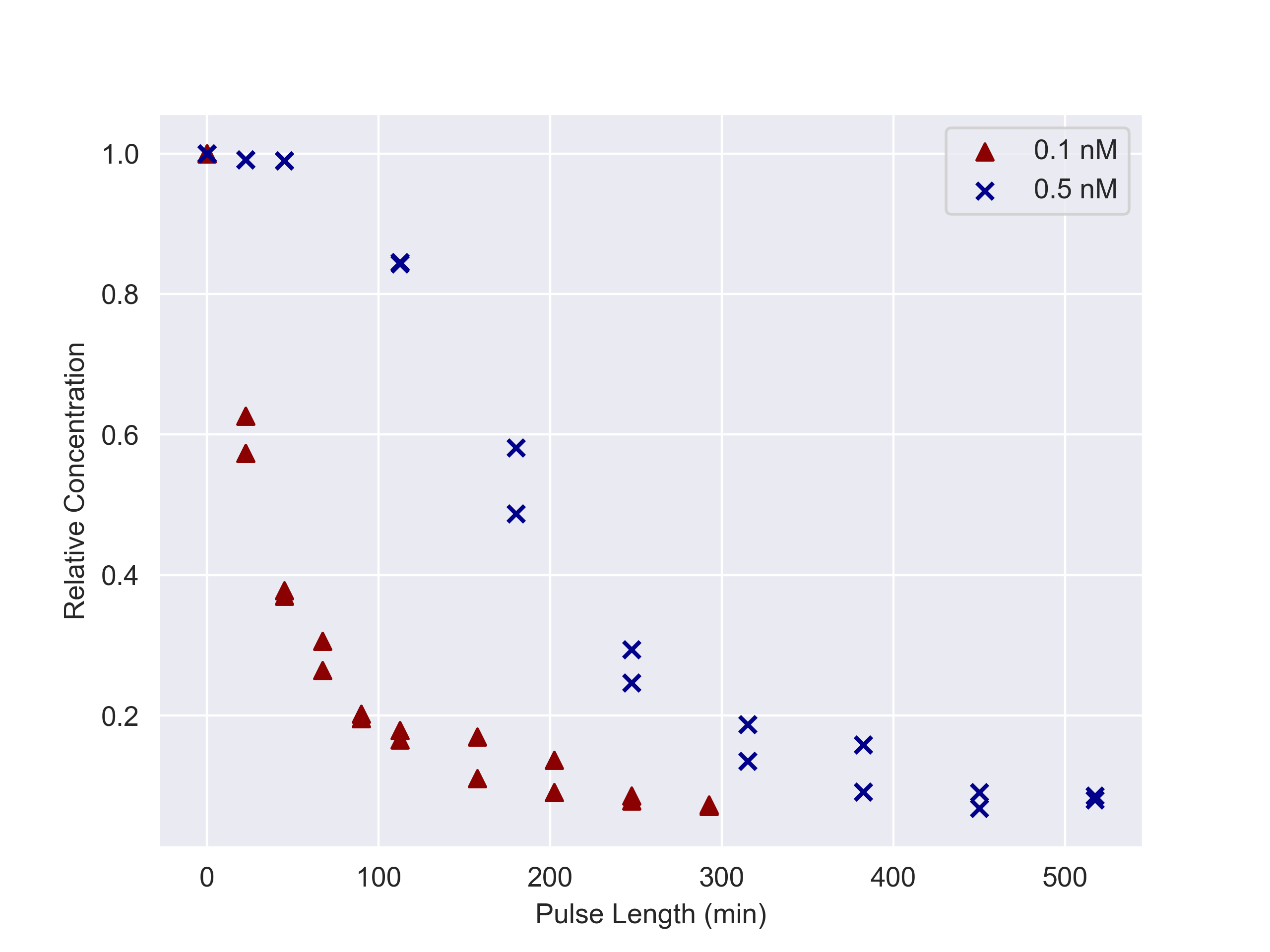

Supplement: Supplementary file 5 — Supplementary Dataset 2 [file 41467_2022_31306_MOESM5_ESM.zip › Individual Simulations Pulse Decoder/8.png]

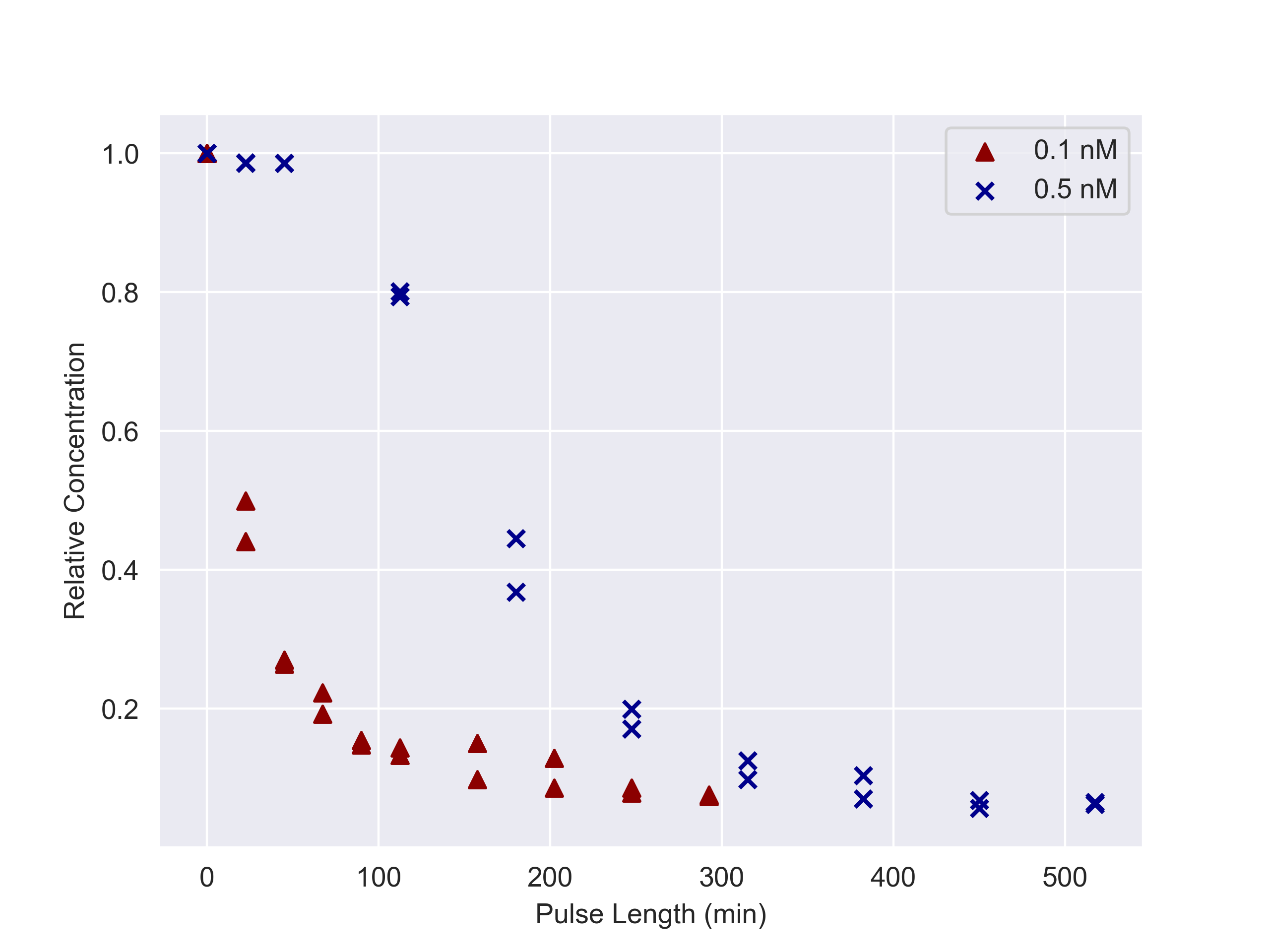

Supplement: Supplementary file 5 — Supplementary Dataset 2 [file 41467_2022_31306_MOESM5_ESM.zip › Individual Simulations Pulse Decoder/80.png]

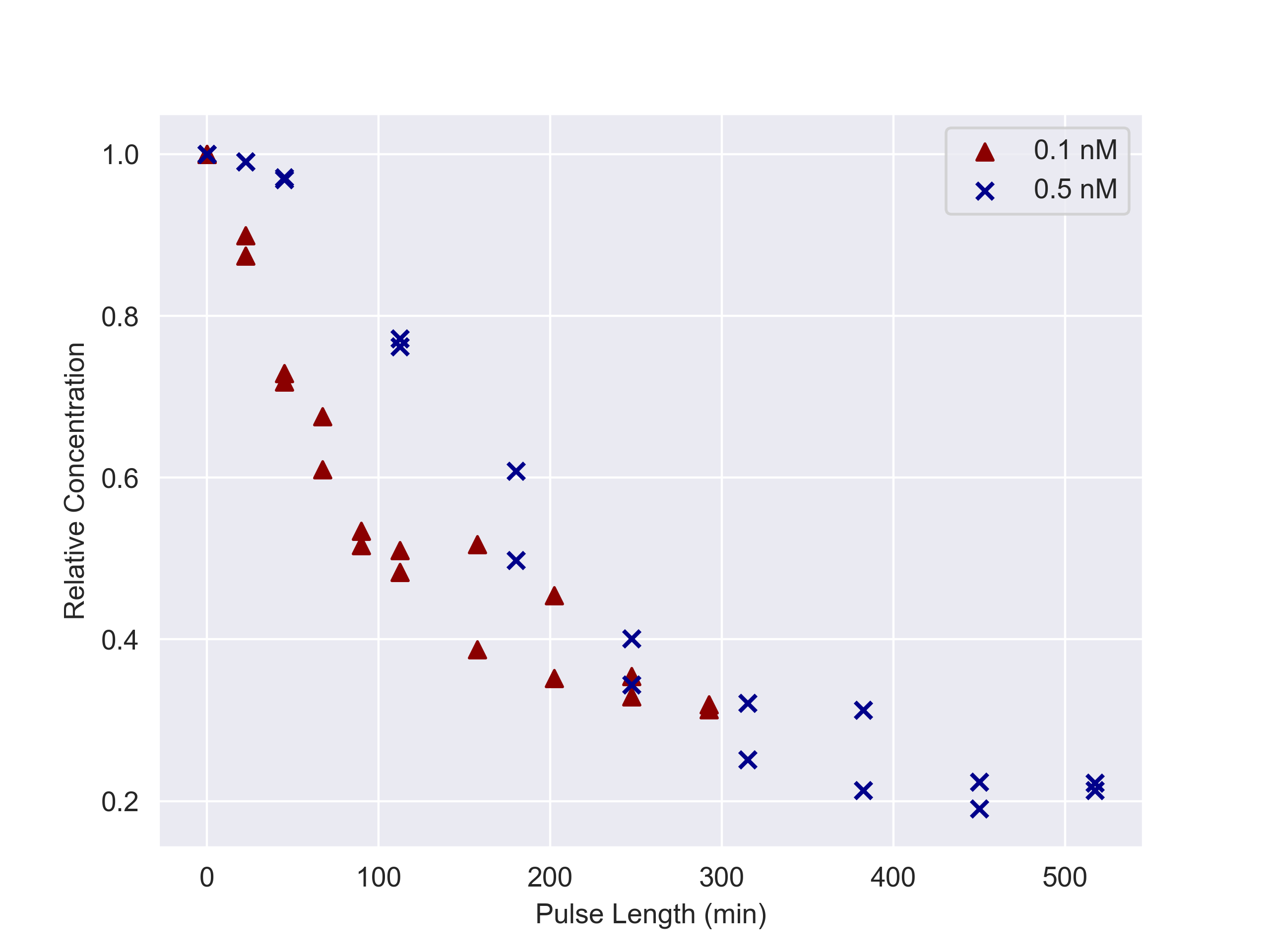

Supplement: Supplementary file 5 — Supplementary Dataset 2 [file 41467_2022_31306_MOESM5_ESM.zip › Individual Simulations Pulse Decoder/81.png]

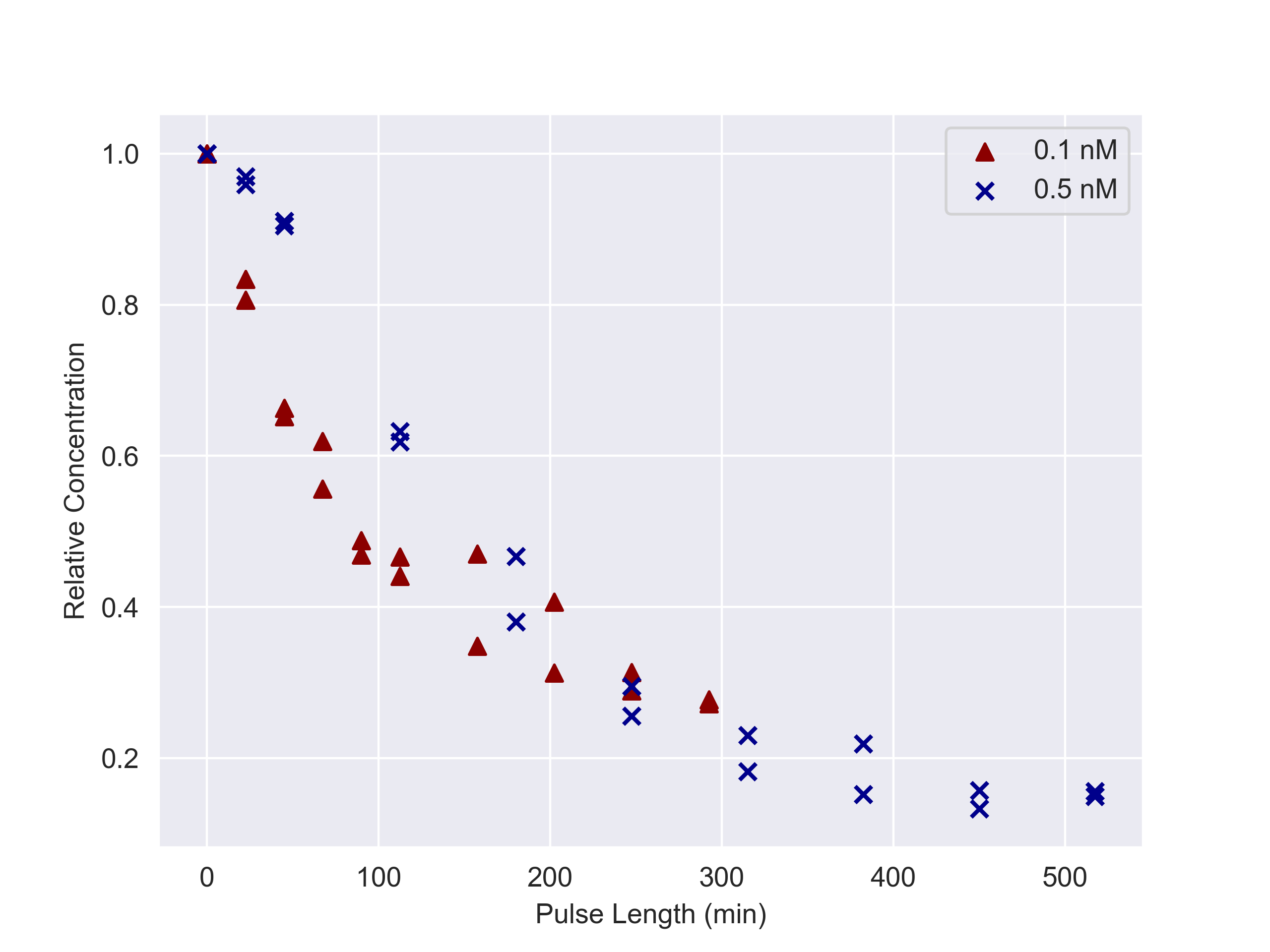

Supplement: Supplementary file 5 — Supplementary Dataset 2 [file 41467_2022_31306_MOESM5_ESM.zip › Individual Simulations Pulse Decoder/82.png]

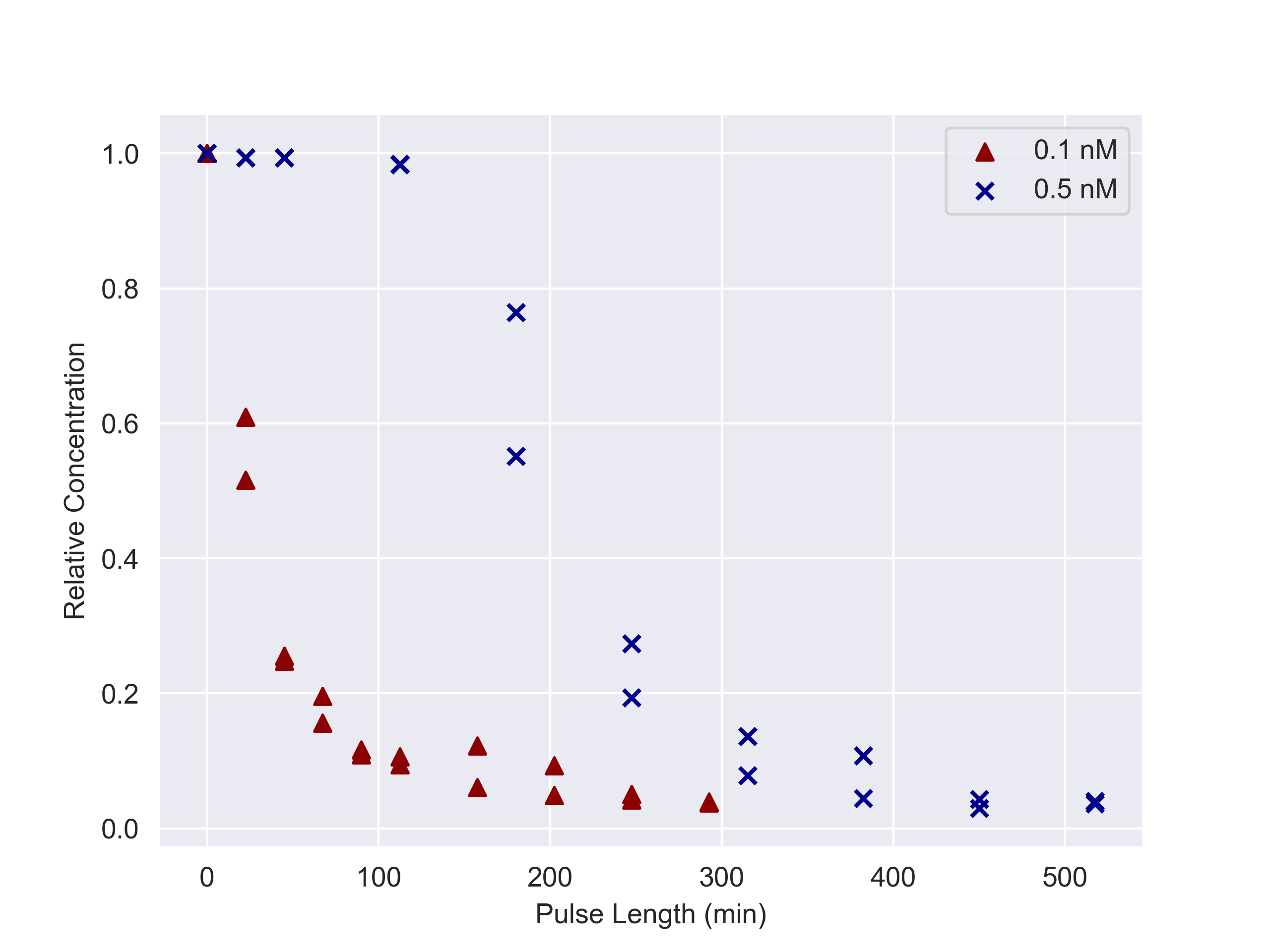

Supplement: Supplementary file 5 — Supplementary Dataset 2 [file 41467_2022_31306_MOESM5_ESM.zip › Individual Simulations Pulse Decoder/83.png]

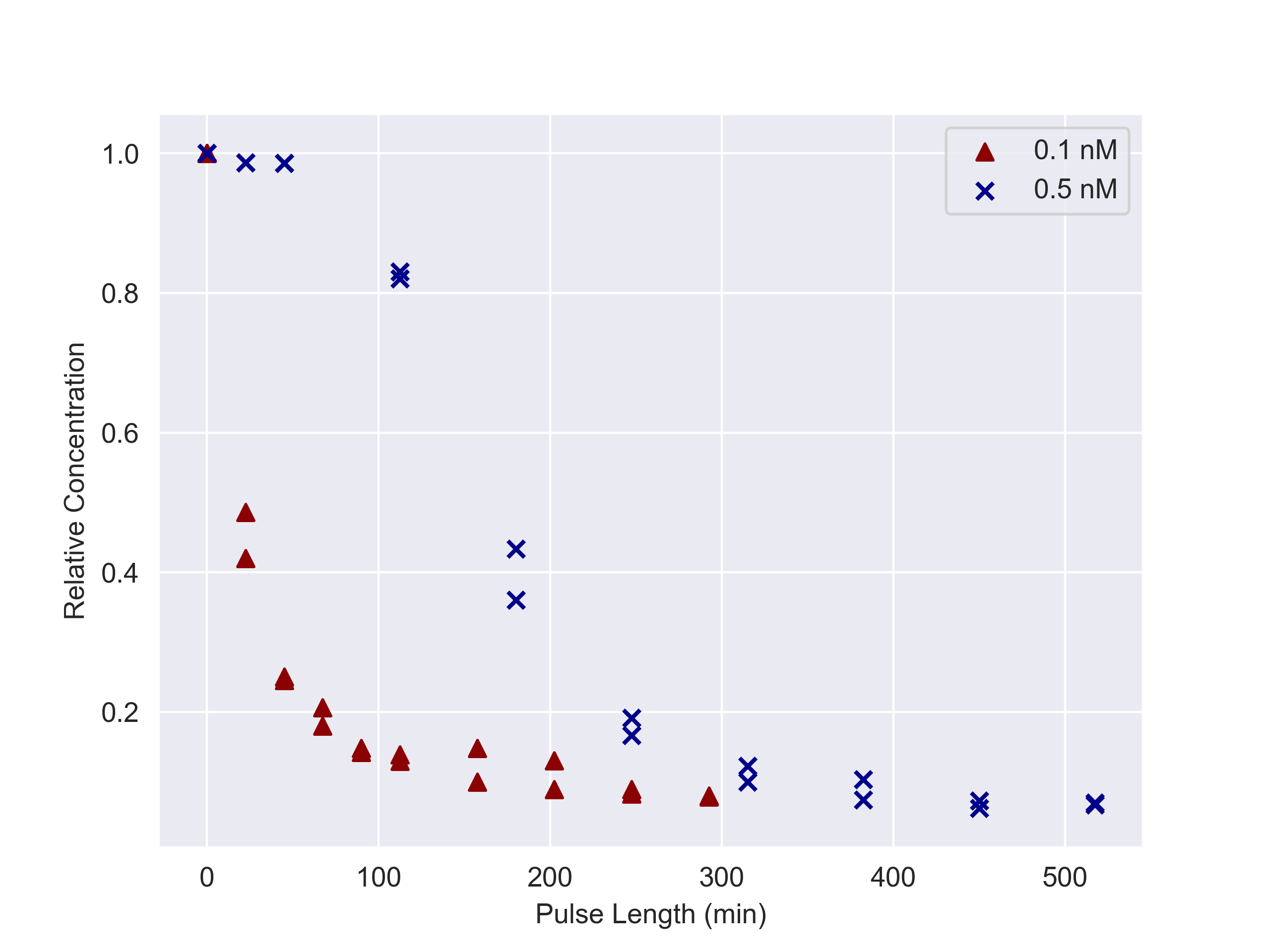

Supplement: Supplementary file 5 — Supplementary Dataset 2 [file 41467_2022_31306_MOESM5_ESM.zip › Individual Simulations Pulse Decoder/84.png]

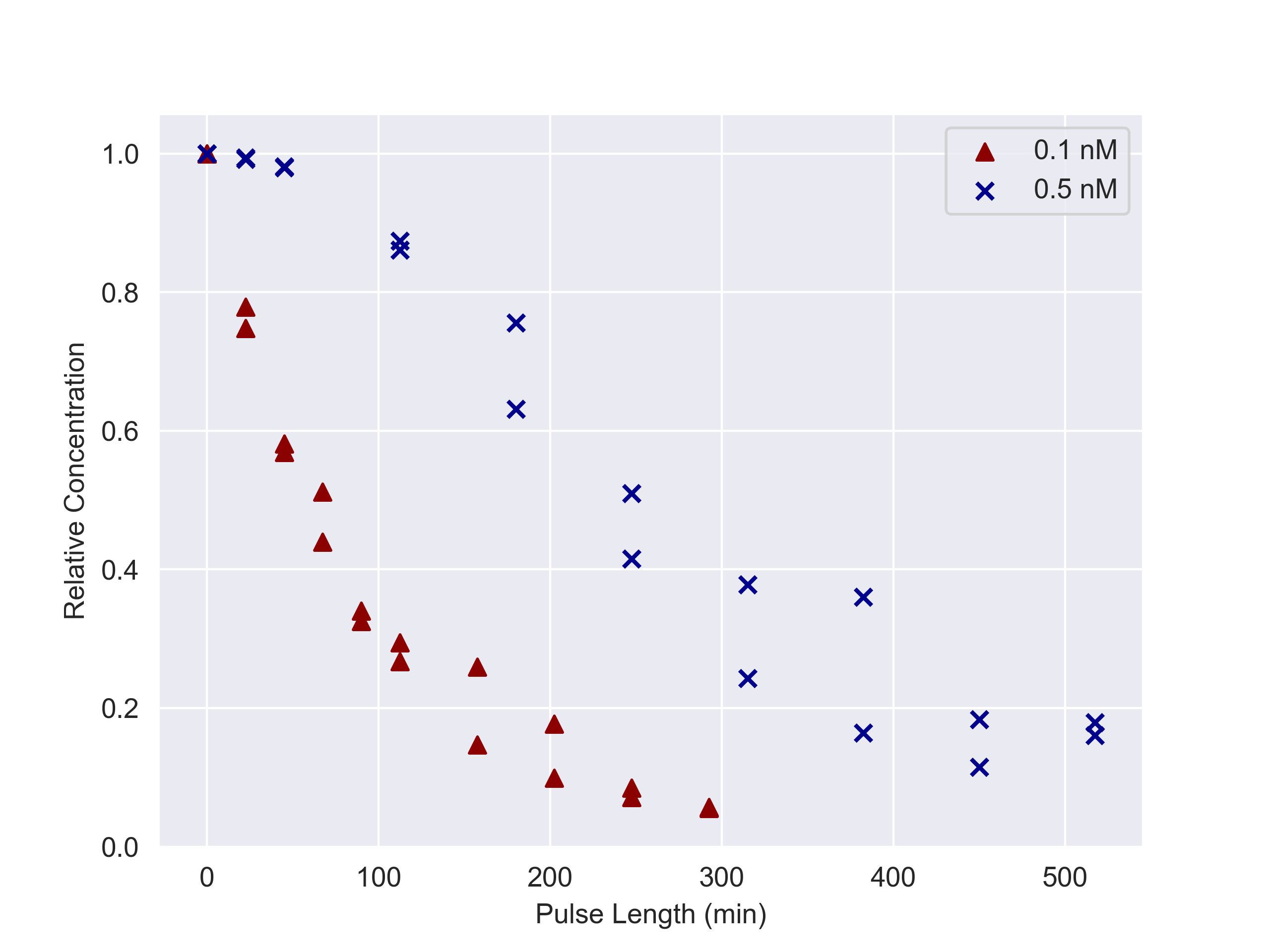

Supplement: Supplementary file 5 — Supplementary Dataset 2 [file 41467_2022_31306_MOESM5_ESM.zip › Individual Simulations Pulse Decoder/85.png]

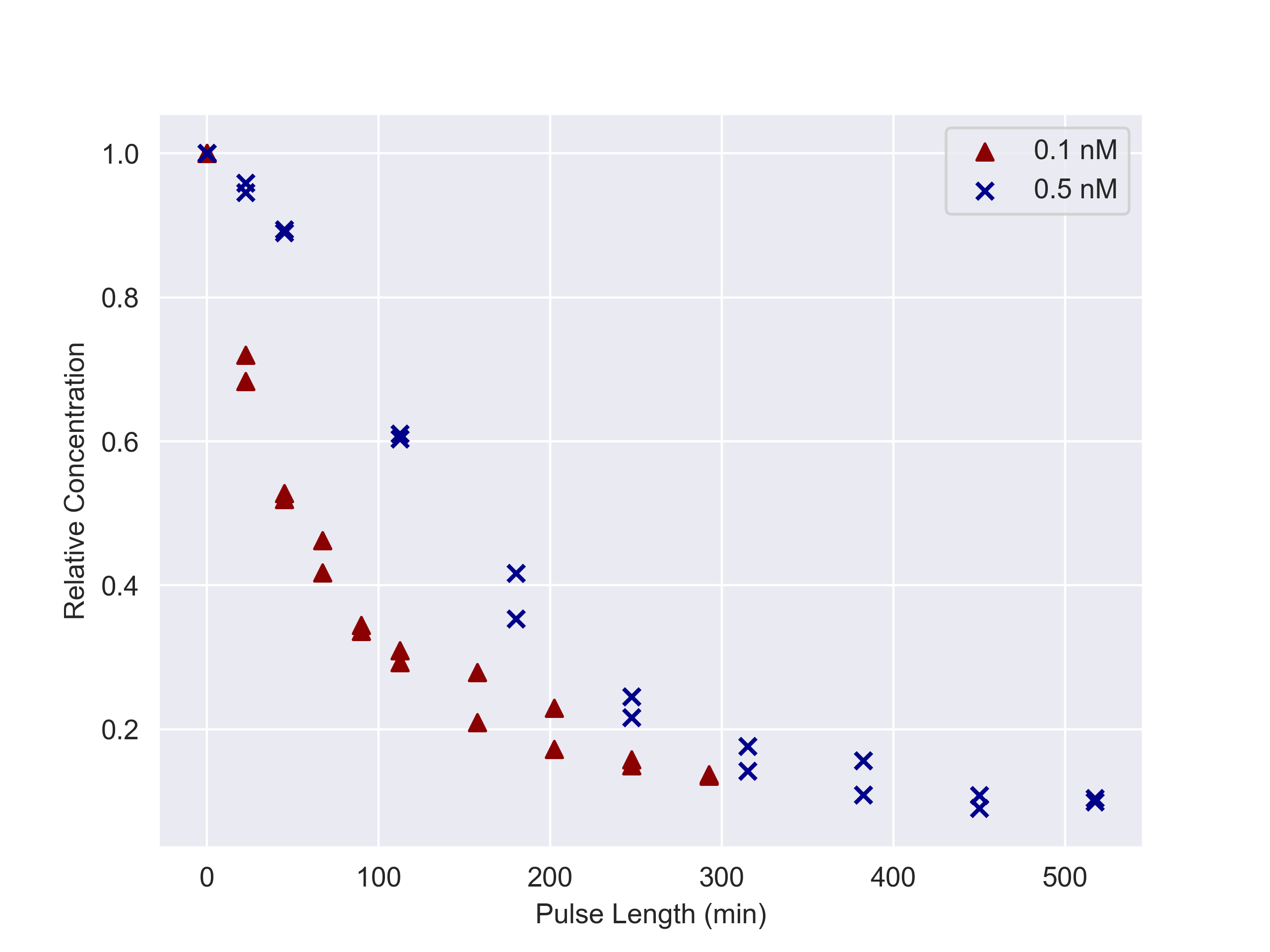

Supplement: Supplementary file 5 — Supplementary Dataset 2 [file 41467_2022_31306_MOESM5_ESM.zip › Individual Simulations Pulse Decoder/86.png]

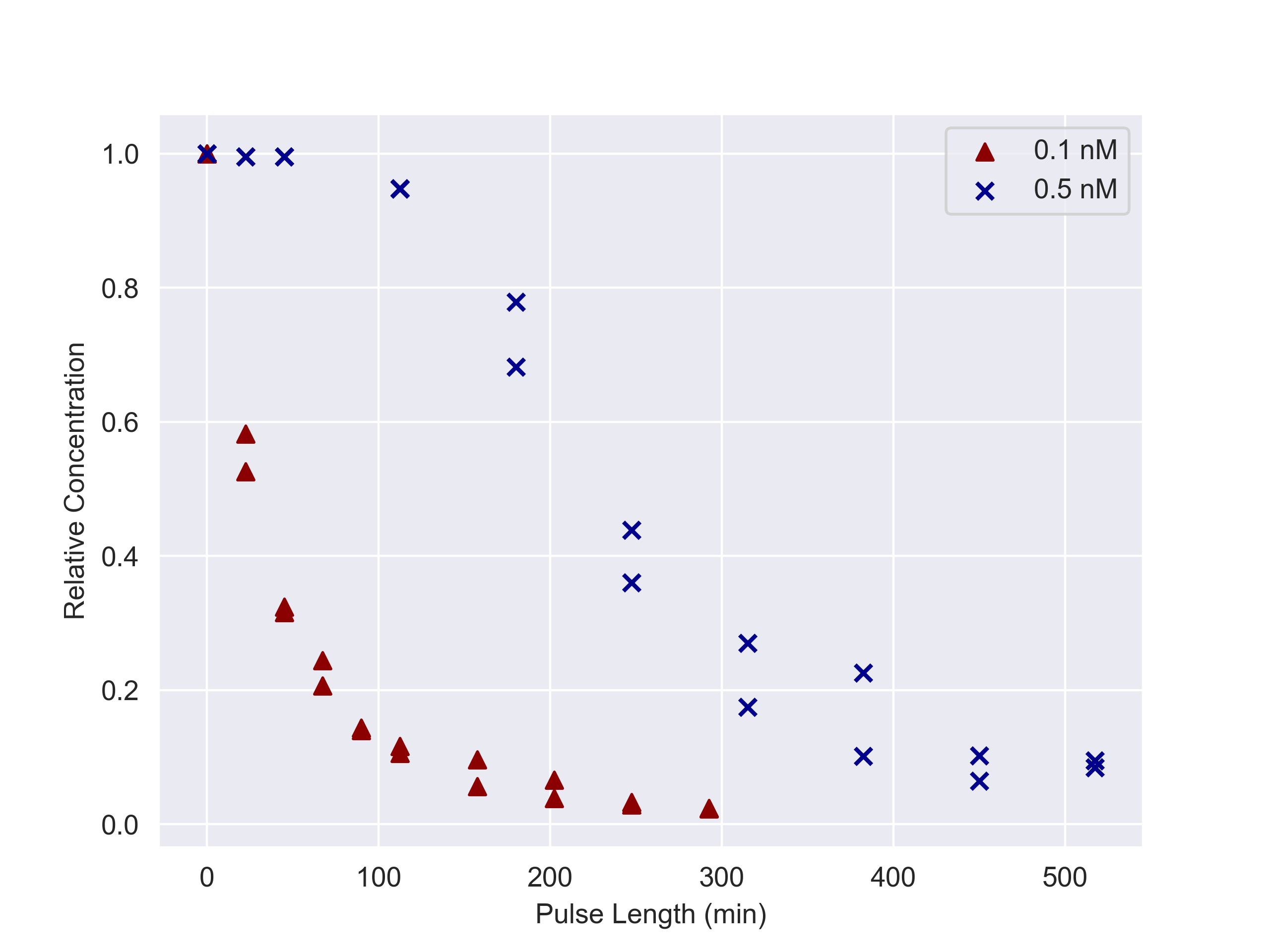

Supplement: Supplementary file 5 — Supplementary Dataset 2 [file 41467_2022_31306_MOESM5_ESM.zip › Individual Simulations Pulse Decoder/87.png]

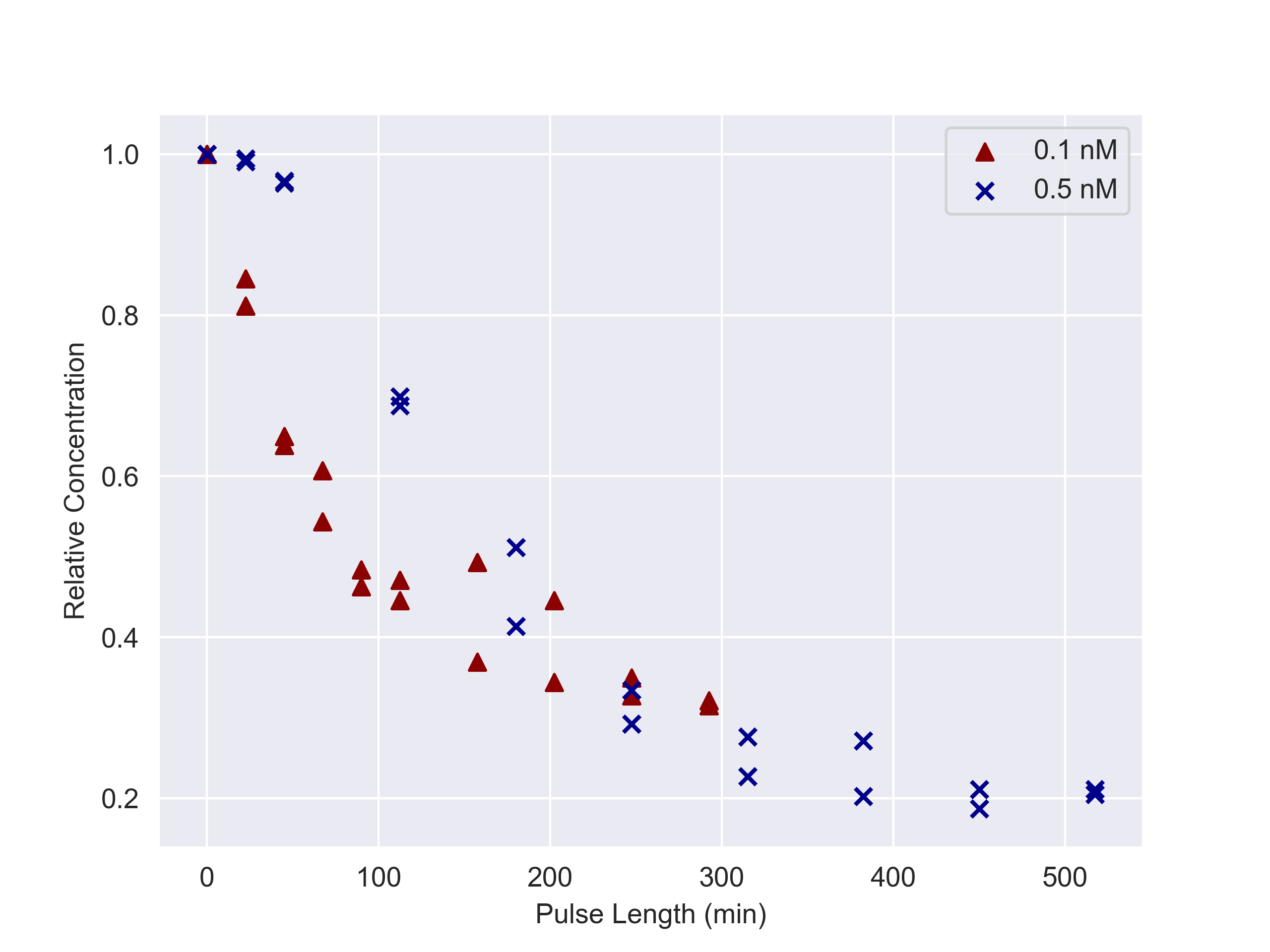

Supplement: Supplementary file 5 — Supplementary Dataset 2 [file 41467_2022_31306_MOESM5_ESM.zip › Individual Simulations Pulse Decoder/88.png]

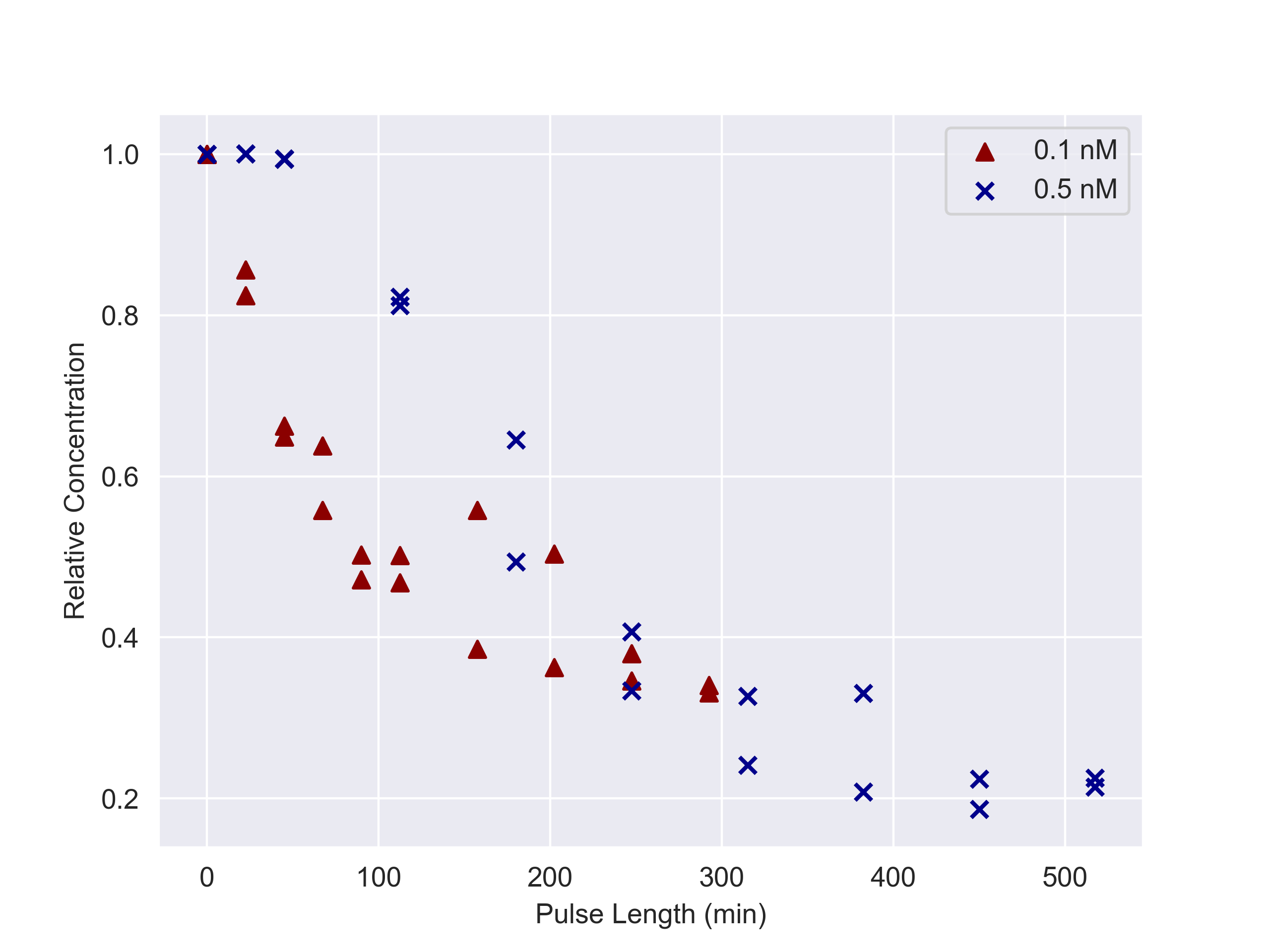

Supplement: Supplementary file 5 — Supplementary Dataset 2 [file 41467_2022_31306_MOESM5_ESM.zip › Individual Simulations Pulse Decoder/89.png]

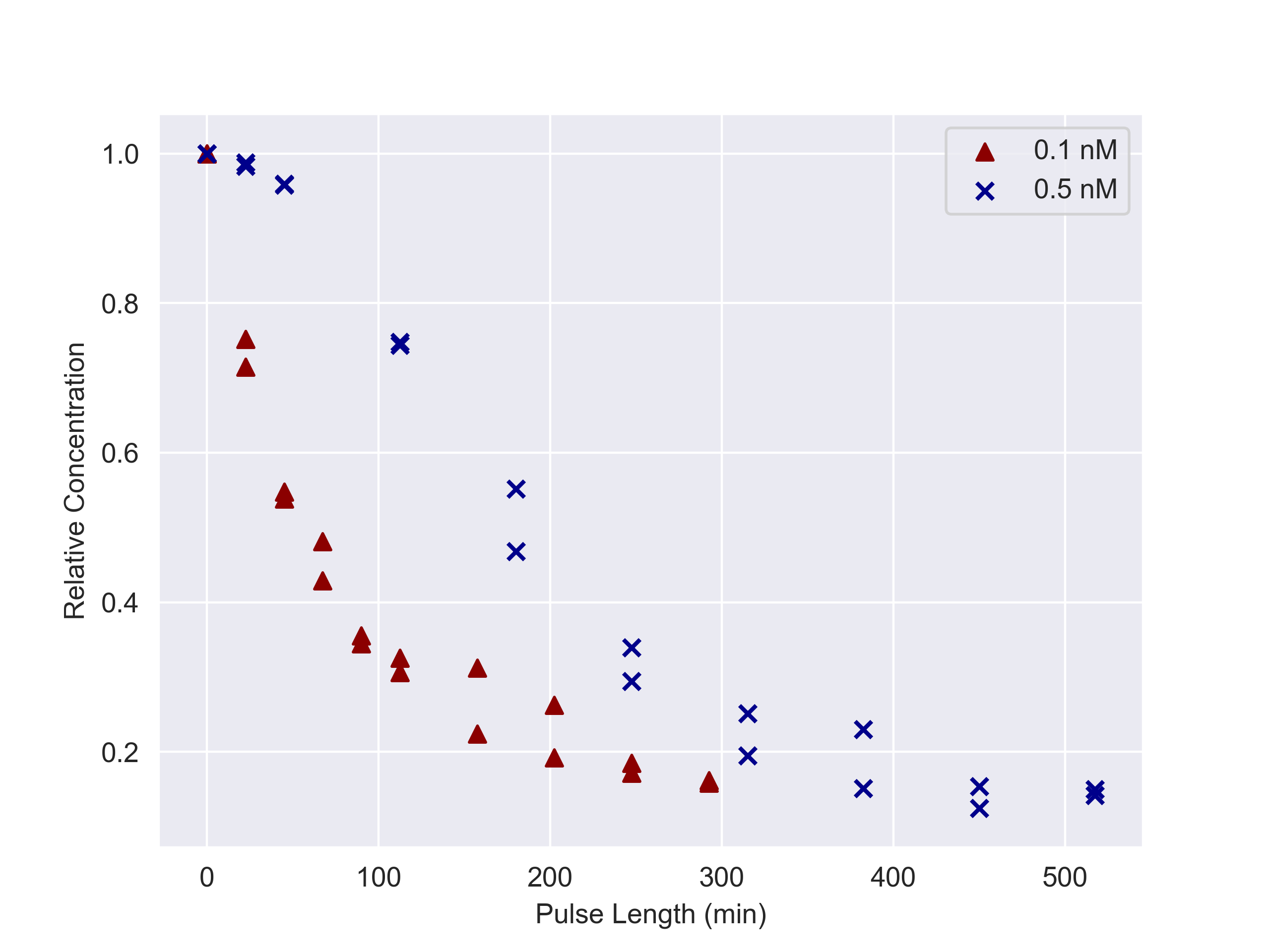

Supplement: Supplementary file 5 — Supplementary Dataset 2 [file 41467_2022_31306_MOESM5_ESM.zip › Individual Simulations Pulse Decoder/9.png]

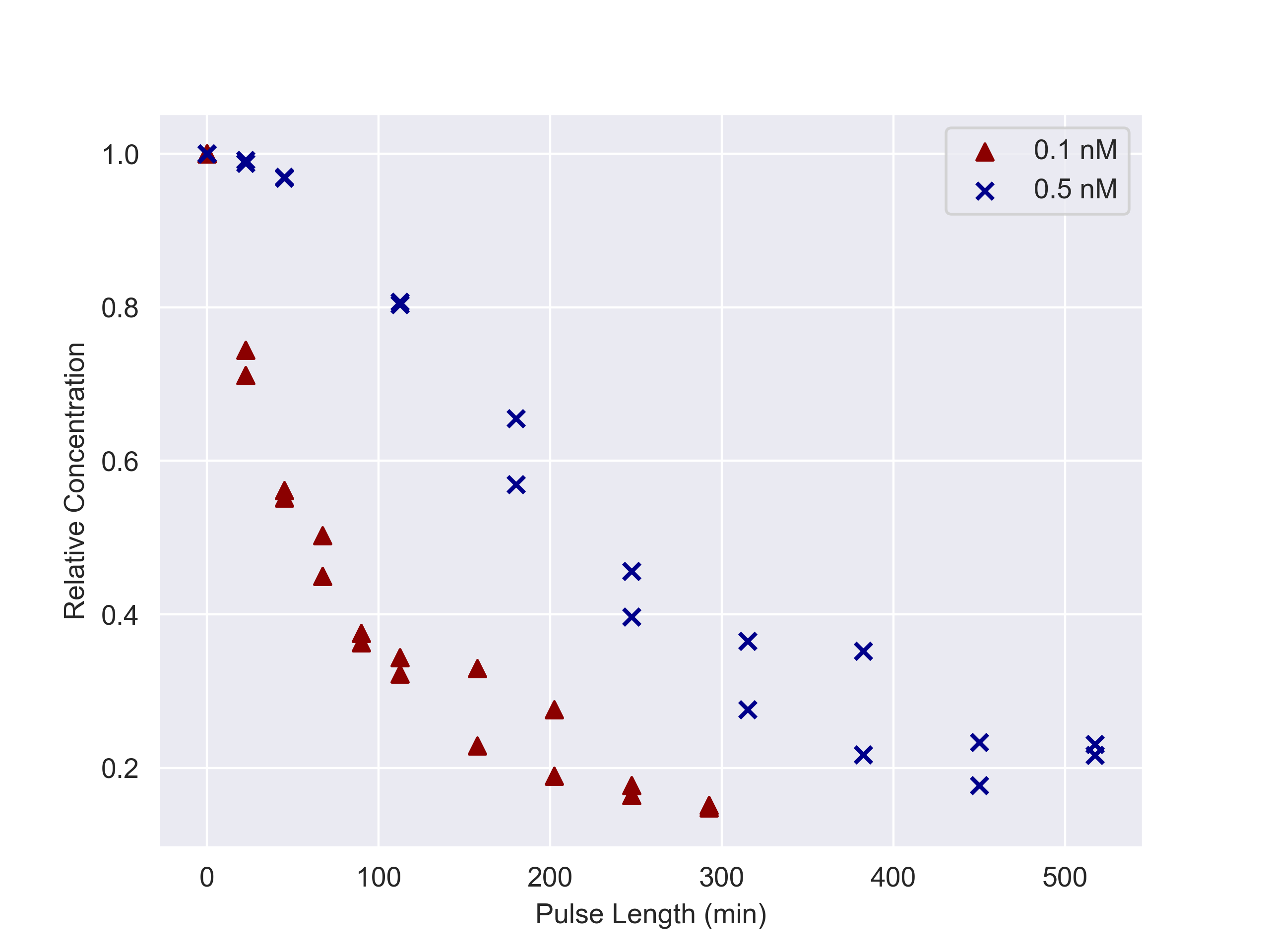

Supplement: Supplementary file 5 — Supplementary Dataset 2 [file 41467_2022_31306_MOESM5_ESM.zip › Individual Simulations Pulse Decoder/90.png]

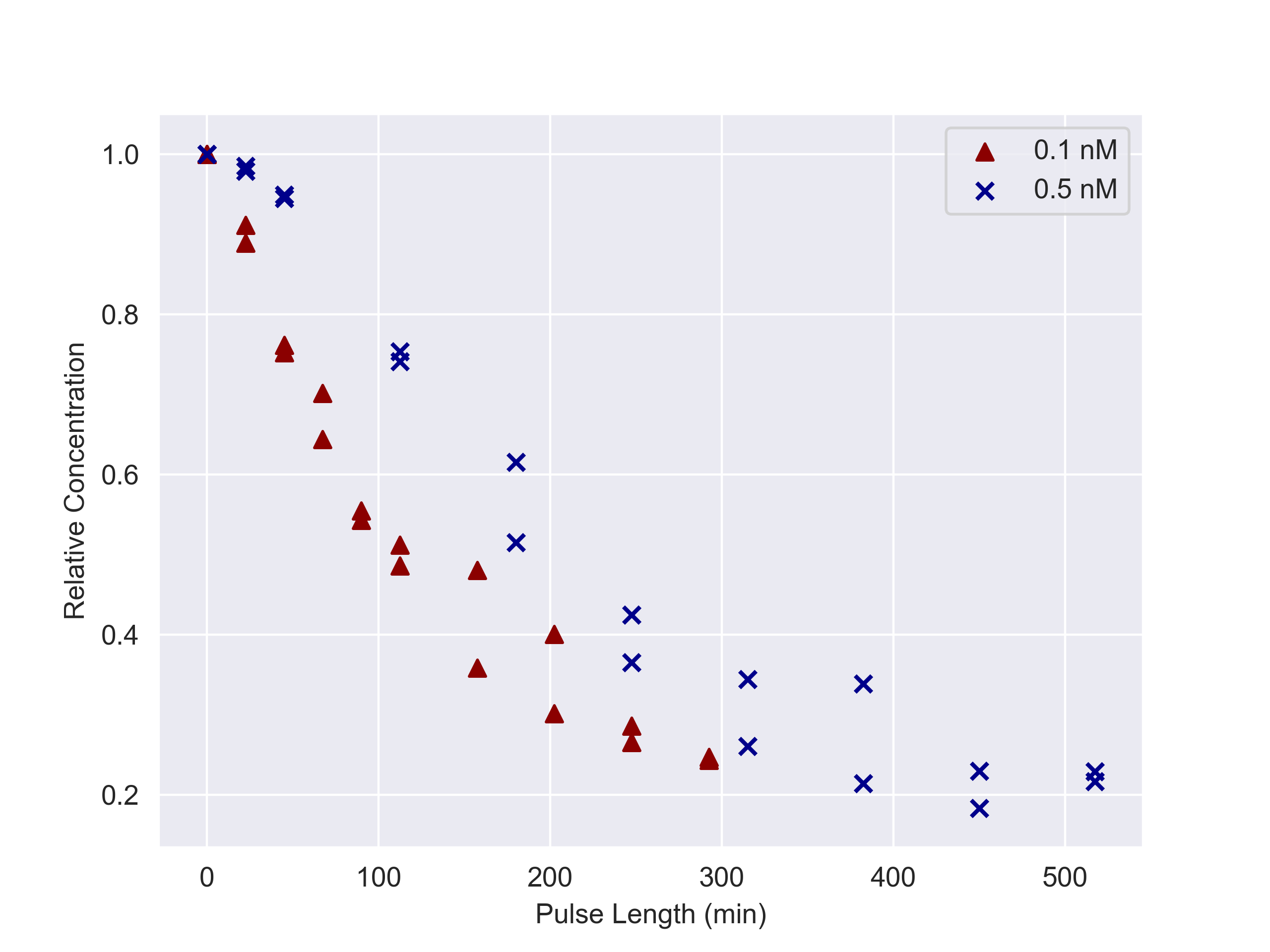

Supplement: Supplementary file 5 — Supplementary Dataset 2 [file 41467_2022_31306_MOESM5_ESM.zip › Individual Simulations Pulse Decoder/91.png]

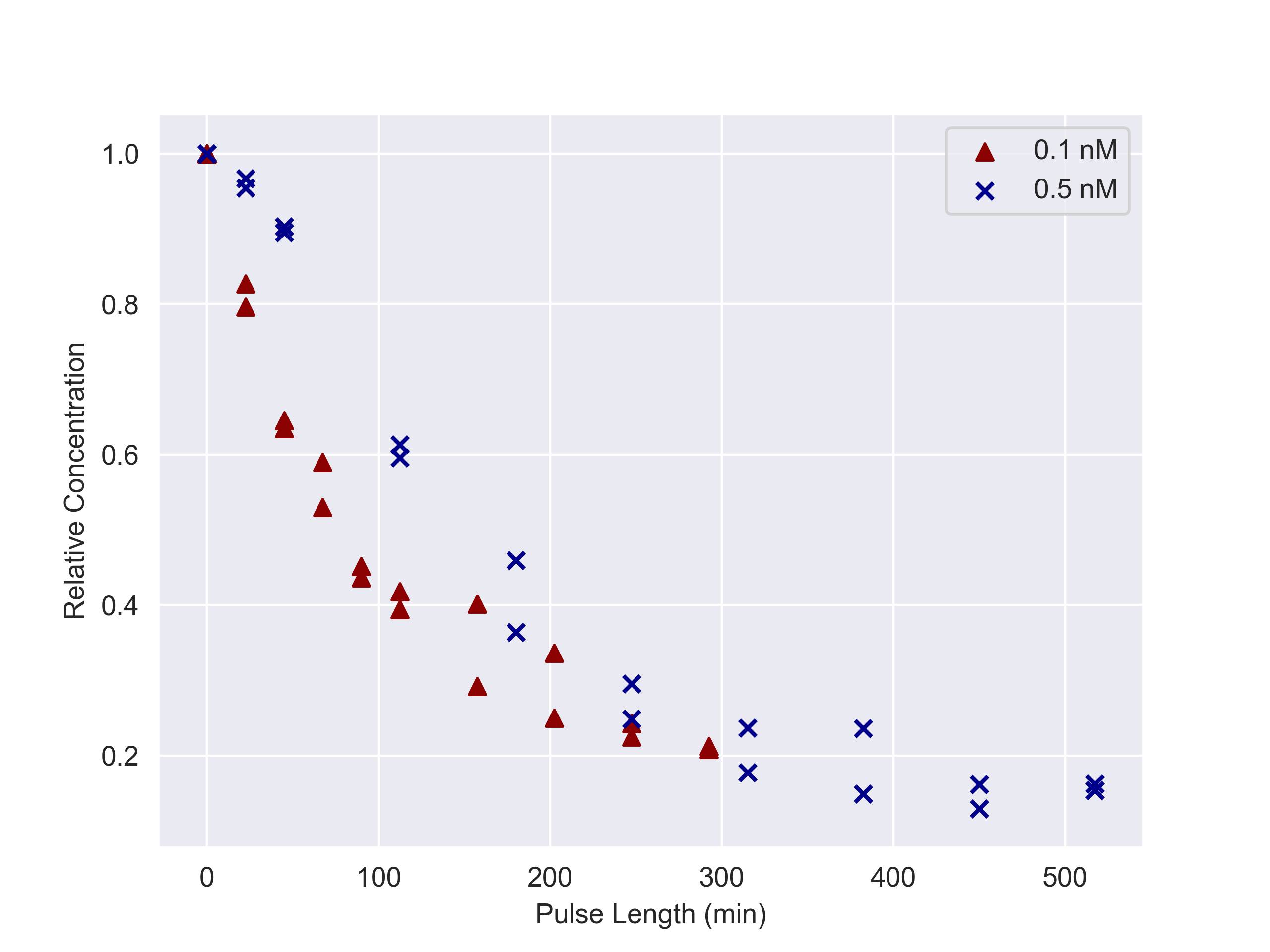

Supplement: Supplementary file 5 — Supplementary Dataset 2 [file 41467_2022_31306_MOESM5_ESM.zip › Individual Simulations Pulse Decoder/92.png]

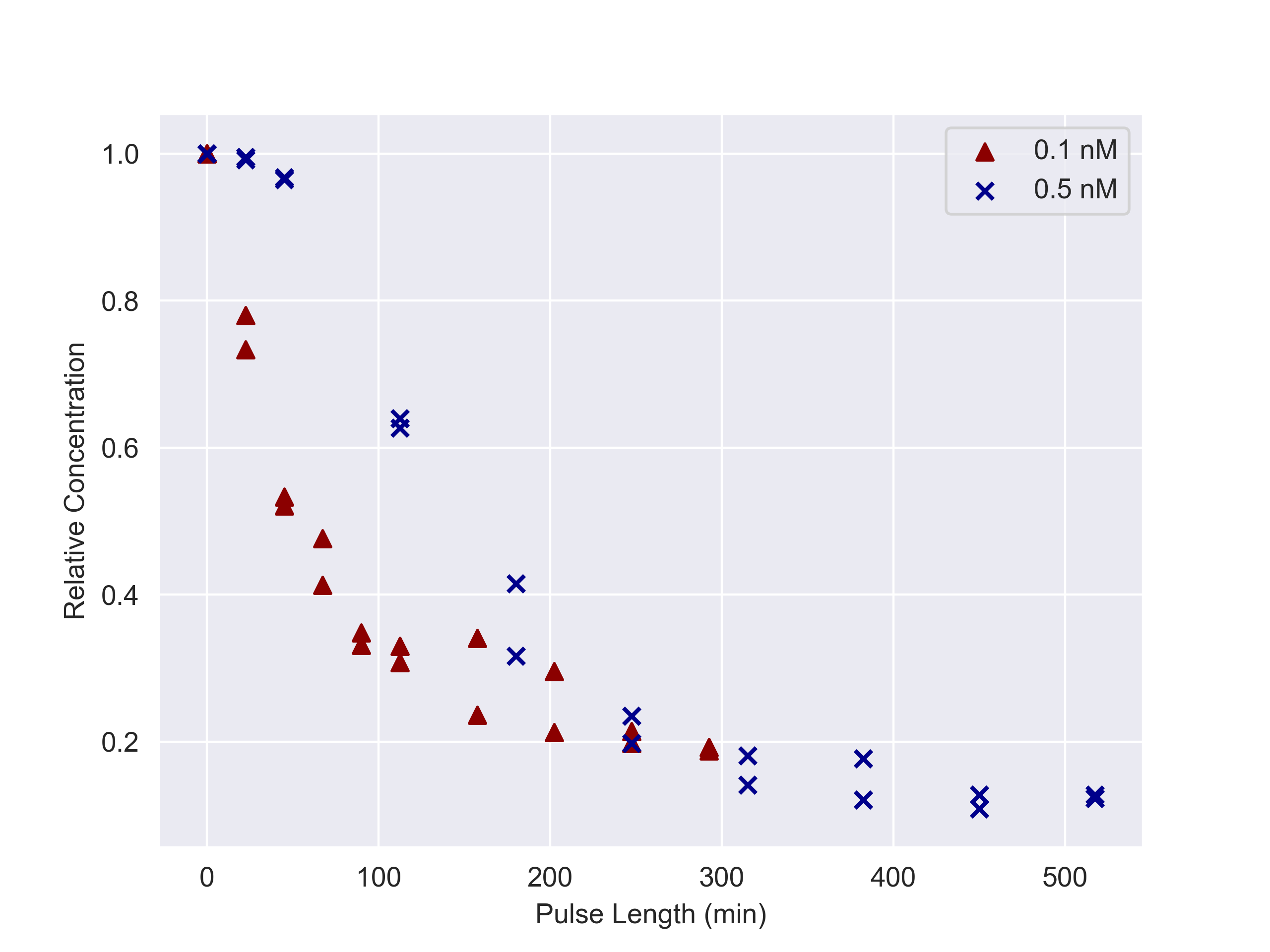

Supplement: Supplementary file 5 — Supplementary Dataset 2 [file 41467_2022_31306_MOESM5_ESM.zip › Individual Simulations Pulse Decoder/93.png]

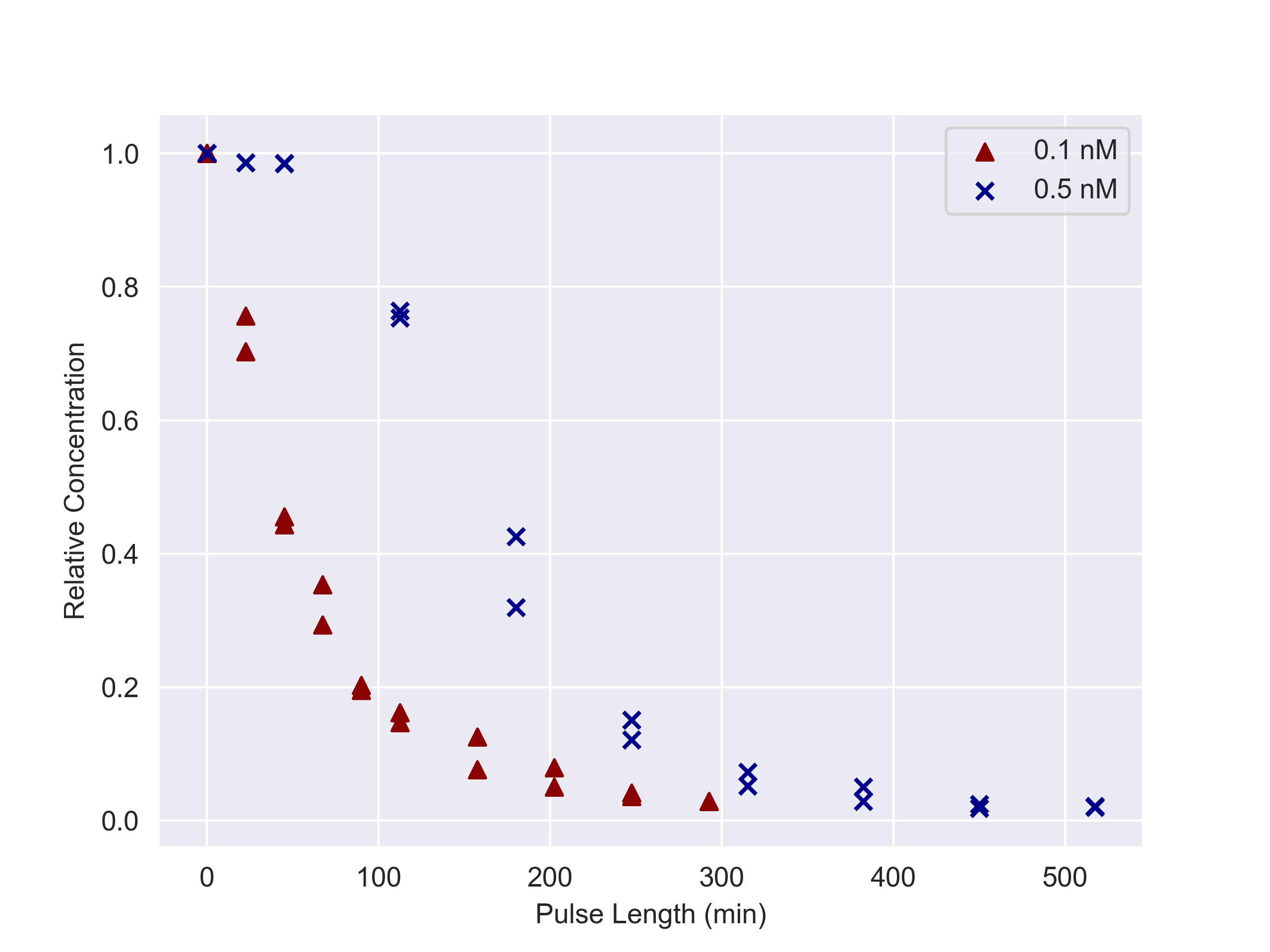

Supplement: Supplementary file 5 — Supplementary Dataset 2 [file 41467_2022_31306_MOESM5_ESM.zip › Individual Simulations Pulse Decoder/94.png]

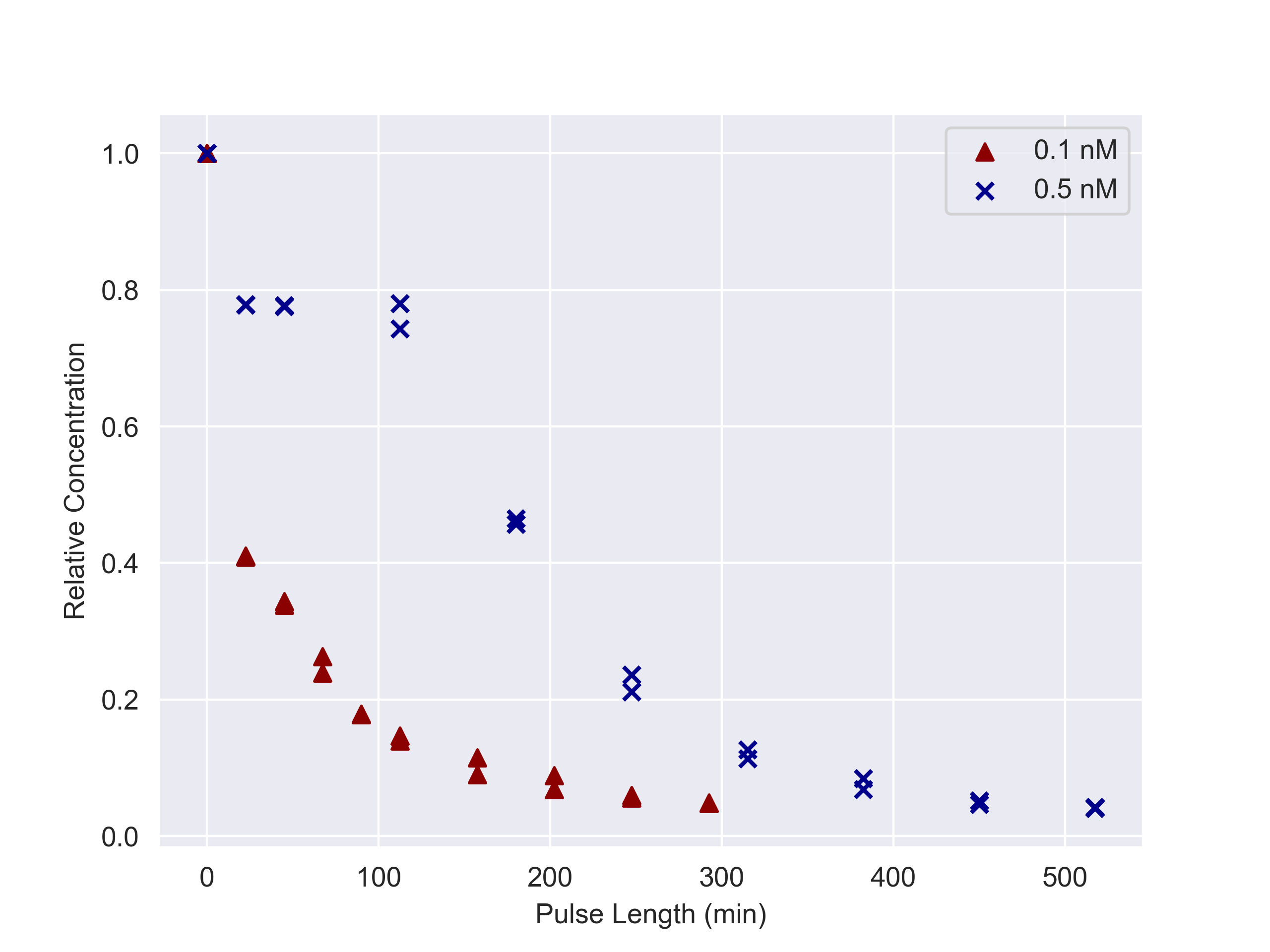

Supplement: Supplementary file 5 — Supplementary Dataset 2 [file 41467_2022_31306_MOESM5_ESM.zip › Individual Simulations Pulse Decoder/95.png]

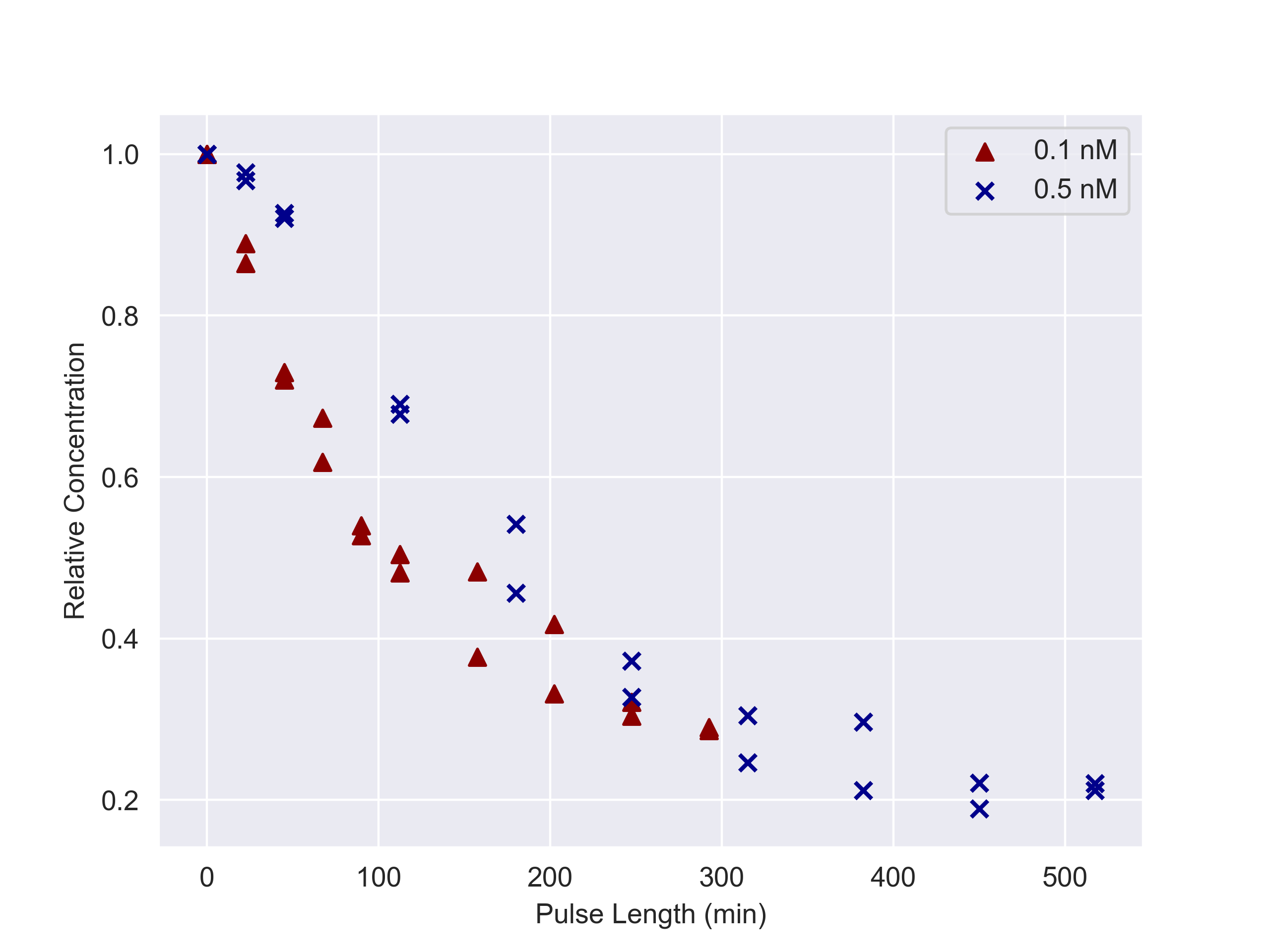

Supplement: Supplementary file 5 — Supplementary Dataset 2 [file 41467_2022_31306_MOESM5_ESM.zip › Individual Simulations Pulse Decoder/96.png]

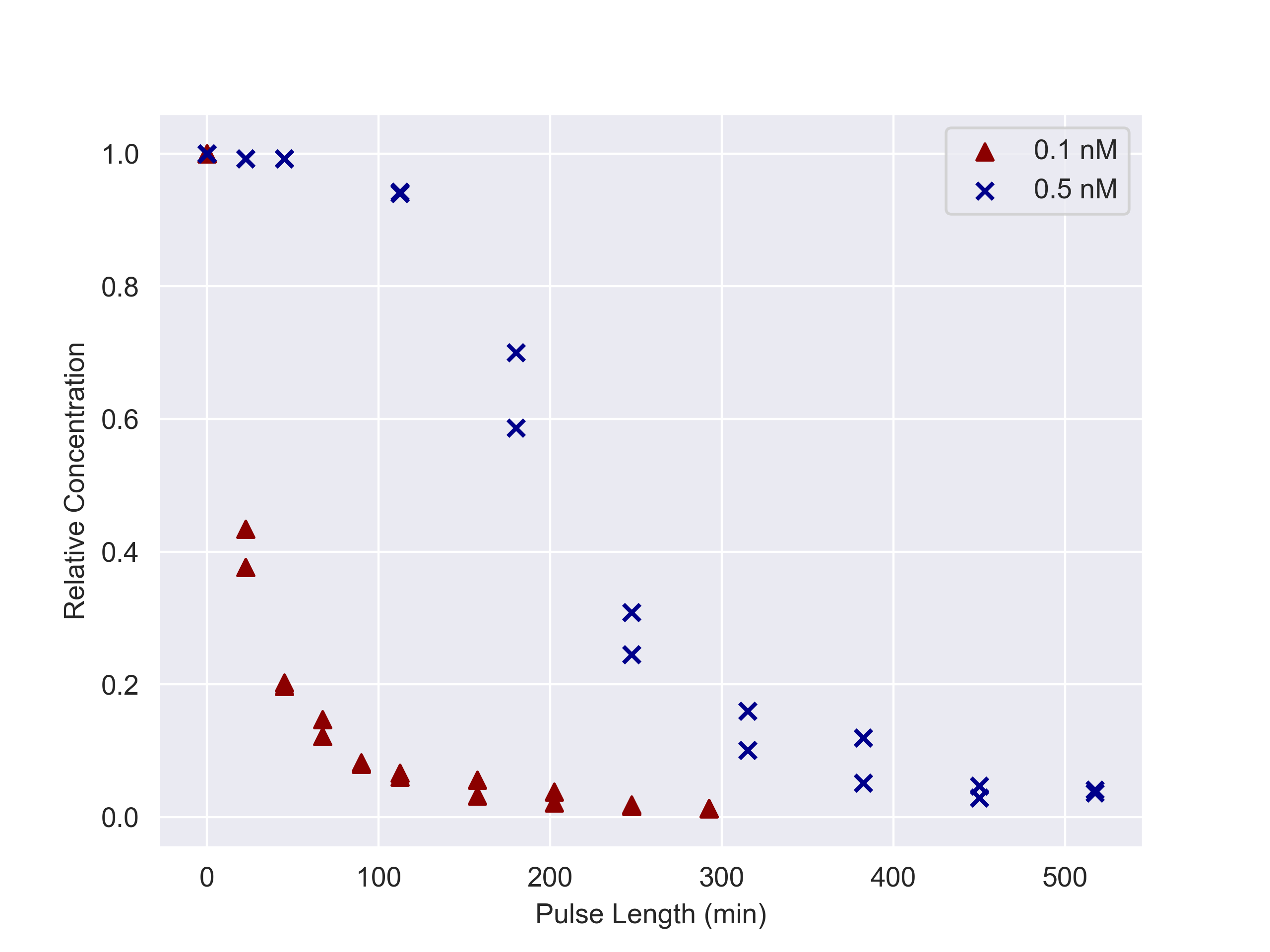

Supplement: Supplementary file 5 — Supplementary Dataset 2 [file 41467_2022_31306_MOESM5_ESM.zip › Individual Simulations Pulse Decoder/97.png]

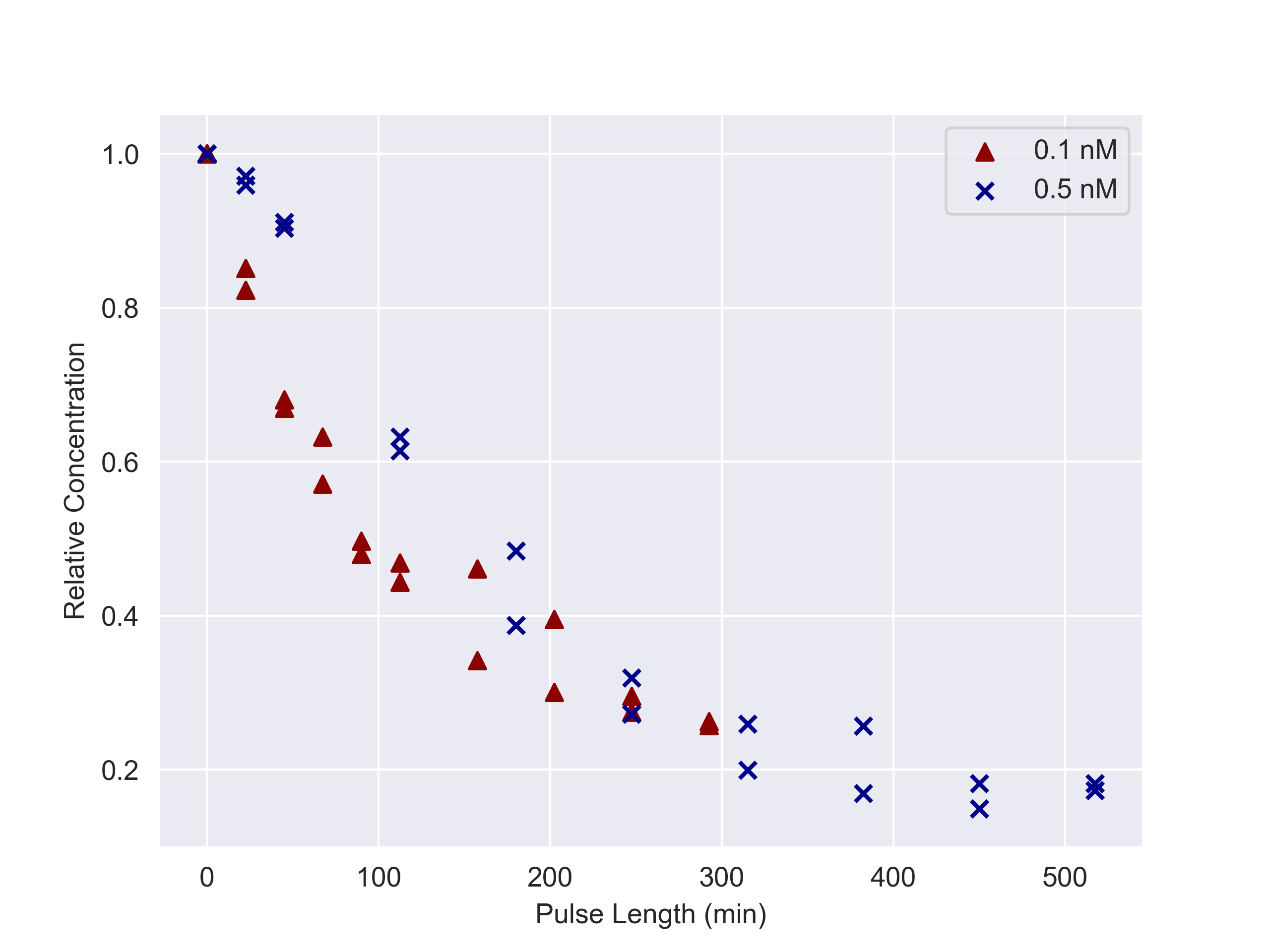

Supplement: Supplementary file 5 — Supplementary Dataset 2 [file 41467_2022_31306_MOESM5_ESM.zip › Individual Simulations Pulse Decoder/98.png]

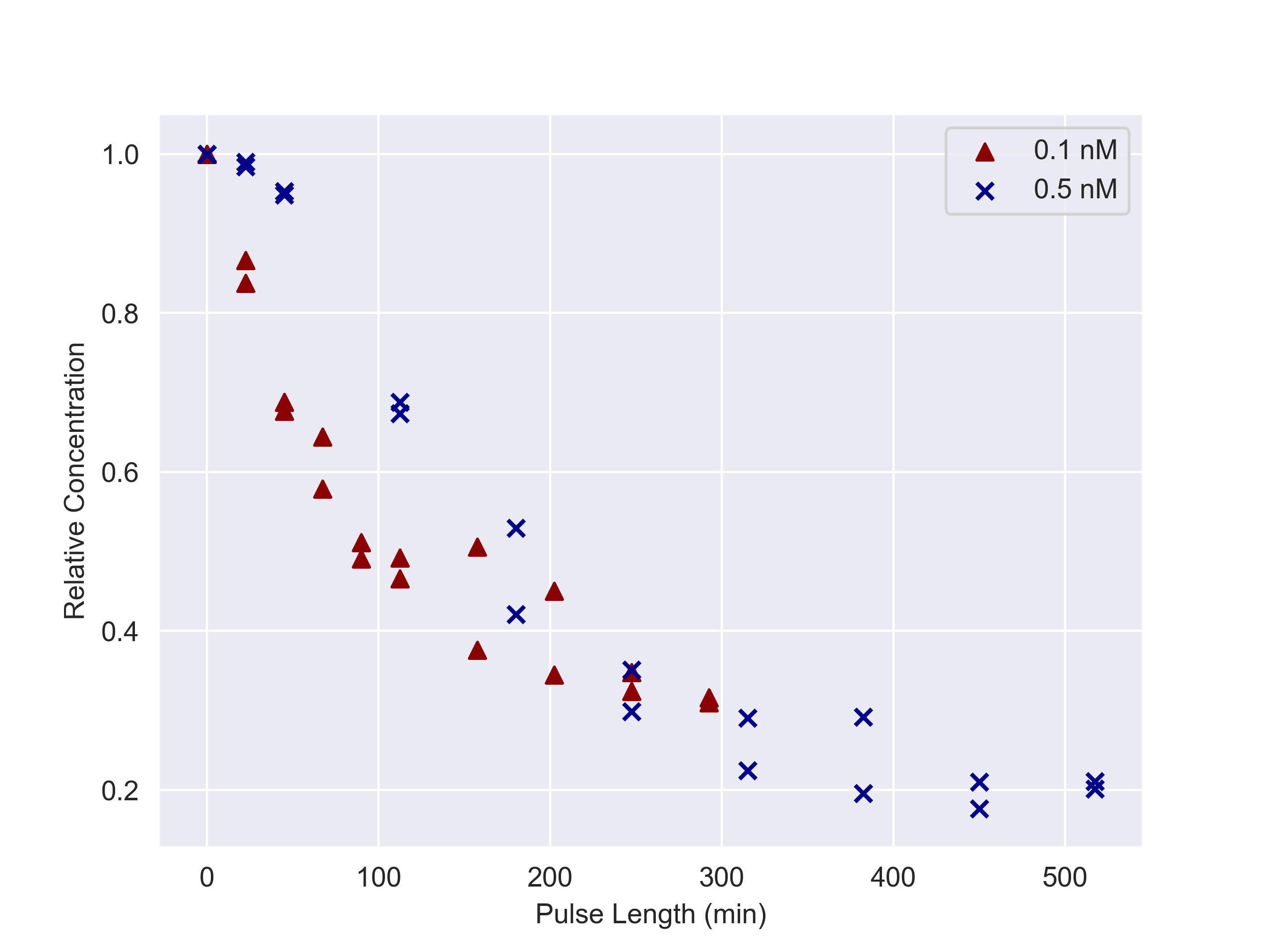

Supplement: Supplementary file 5 — Supplementary Dataset 2 [file 41467_2022_31306_MOESM5_ESM.zip › Individual Simulations Pulse Decoder/99.png]

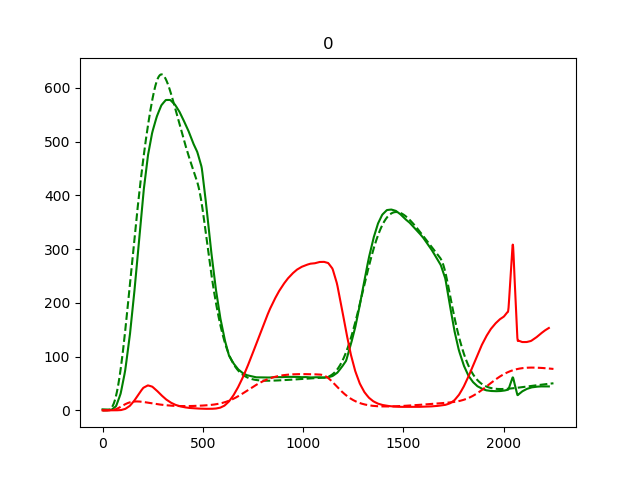

Supplement: Supplementary file 6 — Supplementary Dataset 3 [file 41467_2022_31306_MOESM6_ESM.zip › Individual Simulations Bistable Switch/0.png]

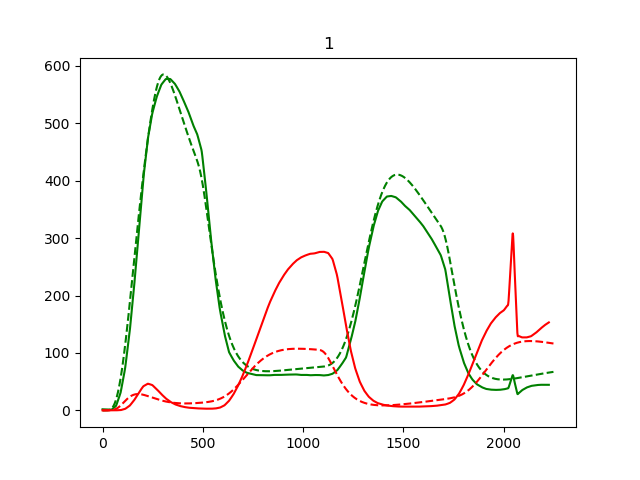

Supplement: Supplementary file 6 — Supplementary Dataset 3 [file 41467_2022_31306_MOESM6_ESM.zip › Individual Simulations Bistable Switch/1.png]

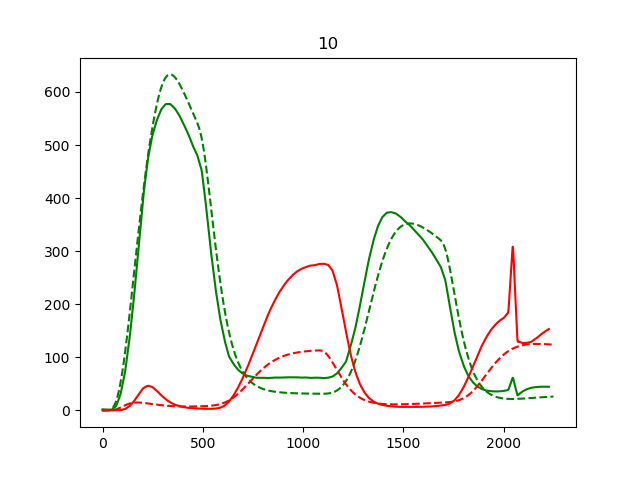

Supplement: Supplementary file 6 — Supplementary Dataset 3 [file 41467_2022_31306_MOESM6_ESM.zip › Individual Simulations Bistable Switch/10.png]

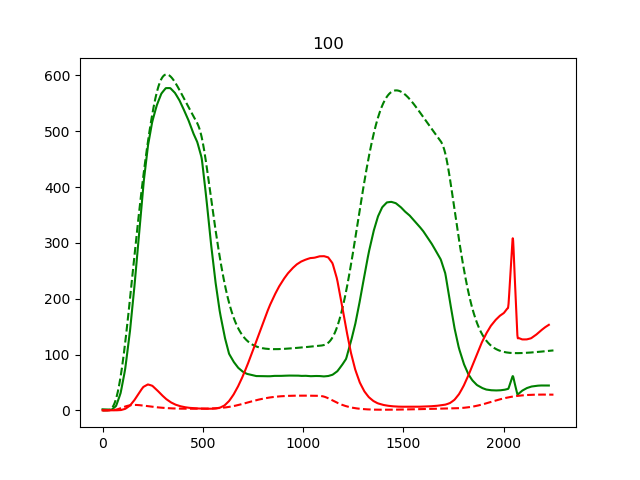

Supplement: Supplementary file 6 — Supplementary Dataset 3 [file 41467_2022_31306_MOESM6_ESM.zip › Individual Simulations Bistable Switch/100.png]

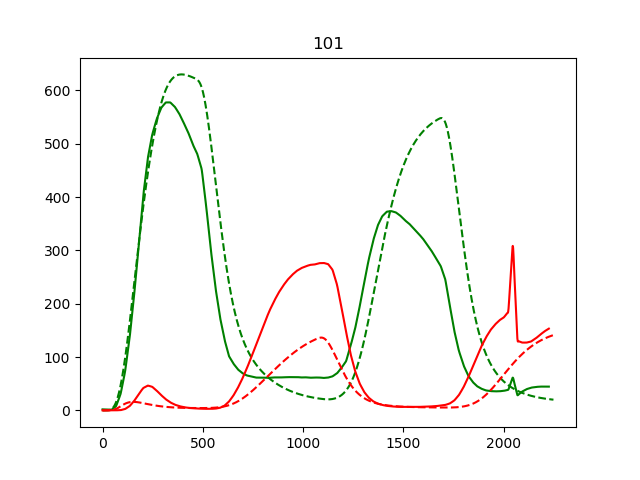

Supplement: Supplementary file 6 — Supplementary Dataset 3 [file 41467_2022_31306_MOESM6_ESM.zip › Individual Simulations Bistable Switch/101.png]

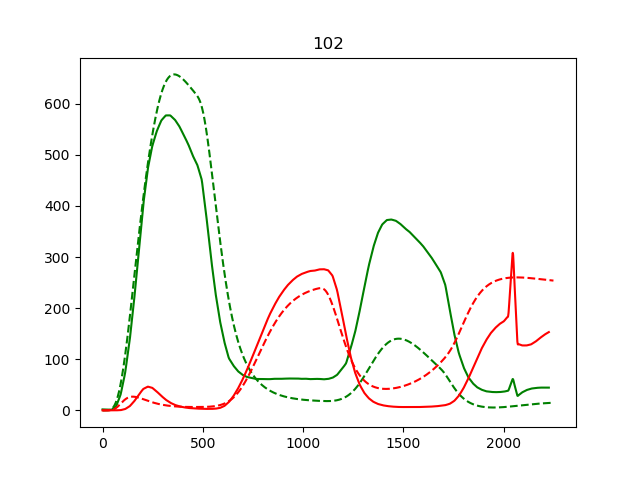

Supplement: Supplementary file 6 — Supplementary Dataset 3 [file 41467_2022_31306_MOESM6_ESM.zip › Individual Simulations Bistable Switch/102.png]

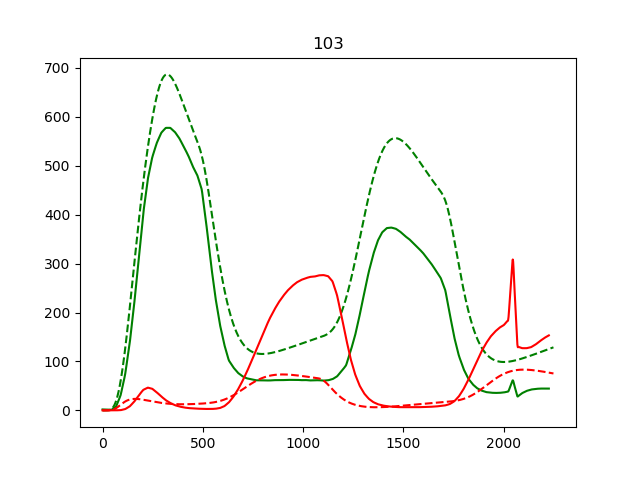

Supplement: Supplementary file 6 — Supplementary Dataset 3 [file 41467_2022_31306_MOESM6_ESM.zip › Individual Simulations Bistable Switch/103.png]

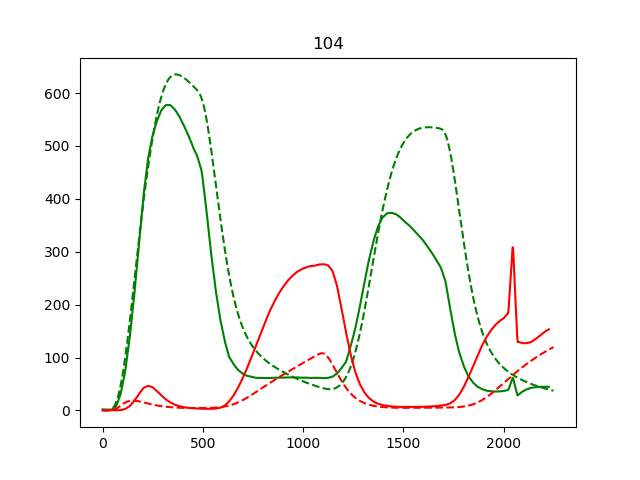

Supplement: Supplementary file 6 — Supplementary Dataset 3 [file 41467_2022_31306_MOESM6_ESM.zip › Individual Simulations Bistable Switch/104.png]

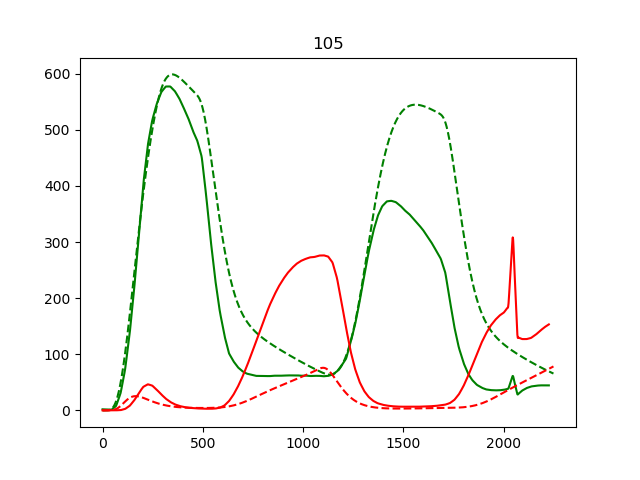

Supplement: Supplementary file 6 — Supplementary Dataset 3 [file 41467_2022_31306_MOESM6_ESM.zip › Individual Simulations Bistable Switch/105.png]

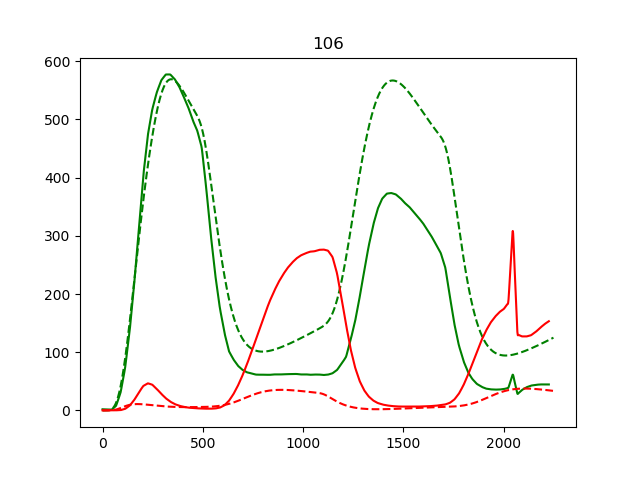

Supplement: Supplementary file 6 — Supplementary Dataset 3 [file 41467_2022_31306_MOESM6_ESM.zip › Individual Simulations Bistable Switch/106.png]

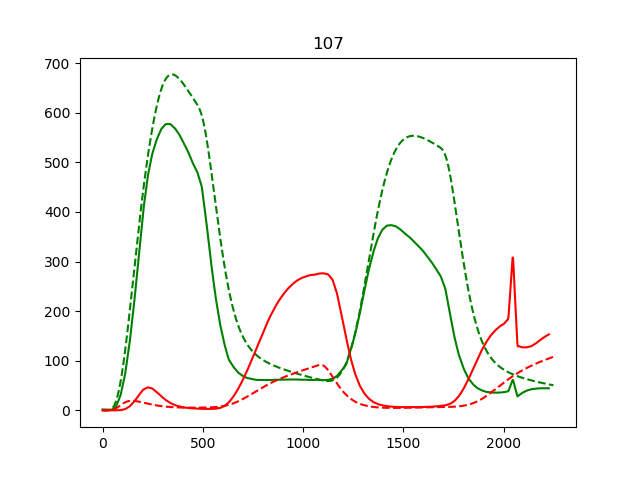

Supplement: Supplementary file 6 — Supplementary Dataset 3 [file 41467_2022_31306_MOESM6_ESM.zip › Individual Simulations Bistable Switch/107.png]

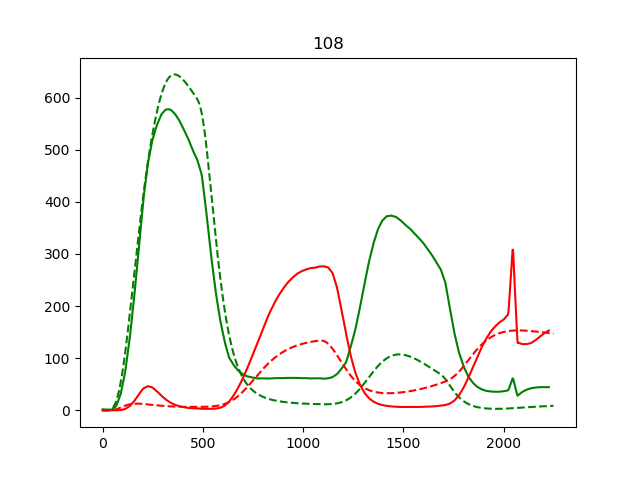

Supplement: Supplementary file 6 — Supplementary Dataset 3 [file 41467_2022_31306_MOESM6_ESM.zip › Individual Simulations Bistable Switch/108.png]

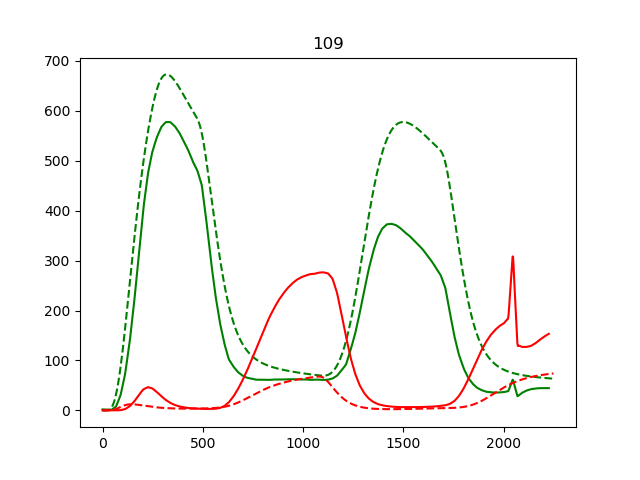

Supplement: Supplementary file 6 — Supplementary Dataset 3 [file 41467_2022_31306_MOESM6_ESM.zip › Individual Simulations Bistable Switch/109.png]

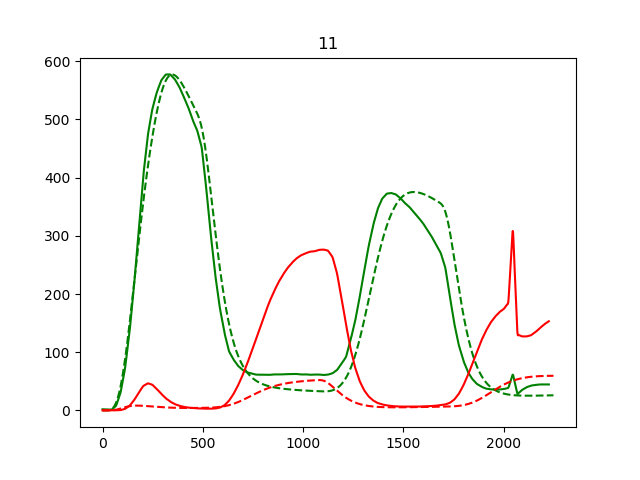

Supplement: Supplementary file 6 — Supplementary Dataset 3 [file 41467_2022_31306_MOESM6_ESM.zip › Individual Simulations Bistable Switch/11.png]

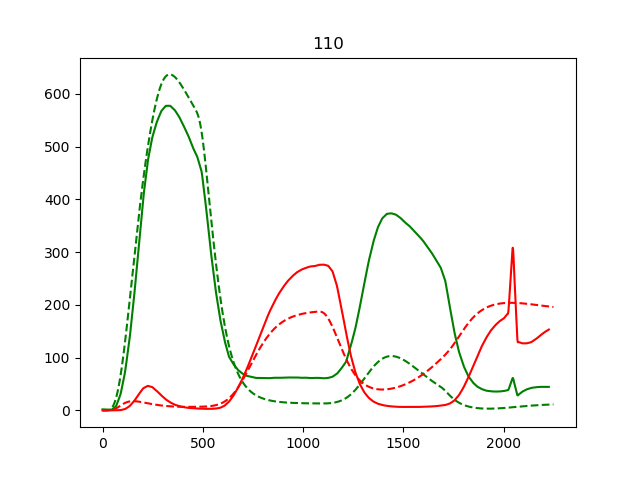

Supplement: Supplementary file 6 — Supplementary Dataset 3 [file 41467_2022_31306_MOESM6_ESM.zip › Individual Simulations Bistable Switch/110.png]

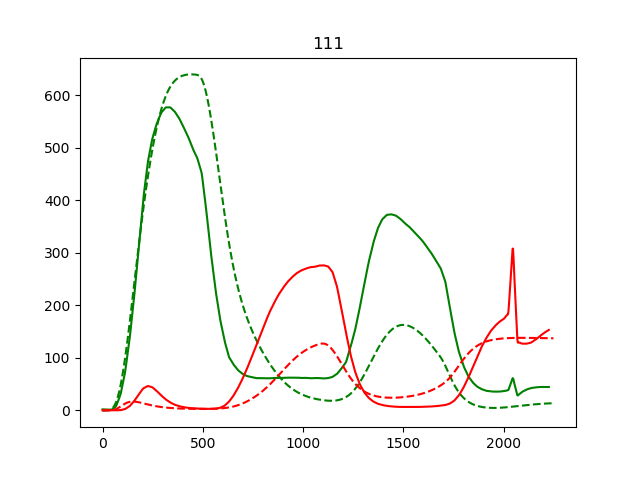

Supplement: Supplementary file 6 — Supplementary Dataset 3 [file 41467_2022_31306_MOESM6_ESM.zip › Individual Simulations Bistable Switch/111.png]

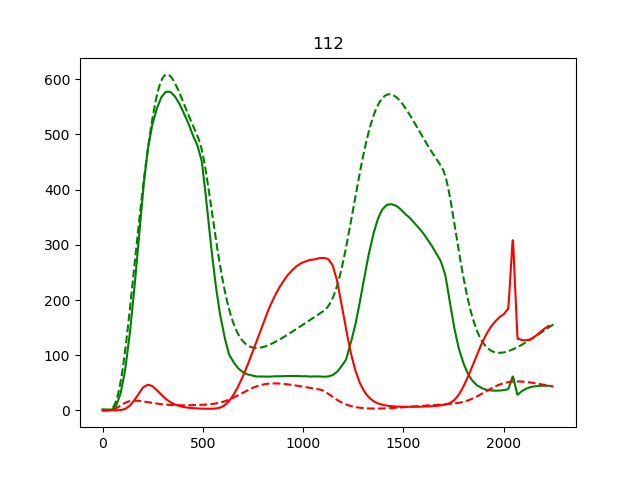

Supplement: Supplementary file 6 — Supplementary Dataset 3 [file 41467_2022_31306_MOESM6_ESM.zip › Individual Simulations Bistable Switch/112.png]

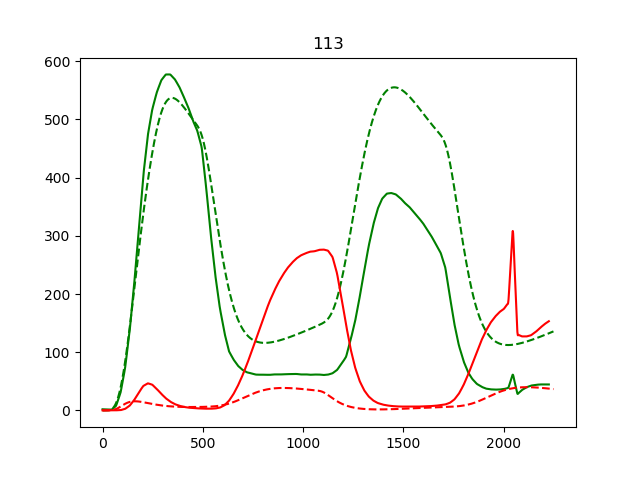

Supplement: Supplementary file 6 — Supplementary Dataset 3 [file 41467_2022_31306_MOESM6_ESM.zip › Individual Simulations Bistable Switch/113.png]

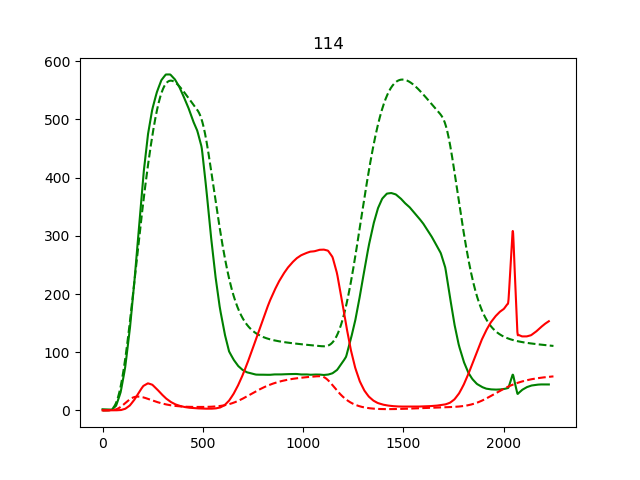

Supplement: Supplementary file 6 — Supplementary Dataset 3 [file 41467_2022_31306_MOESM6_ESM.zip › Individual Simulations Bistable Switch/114.png]

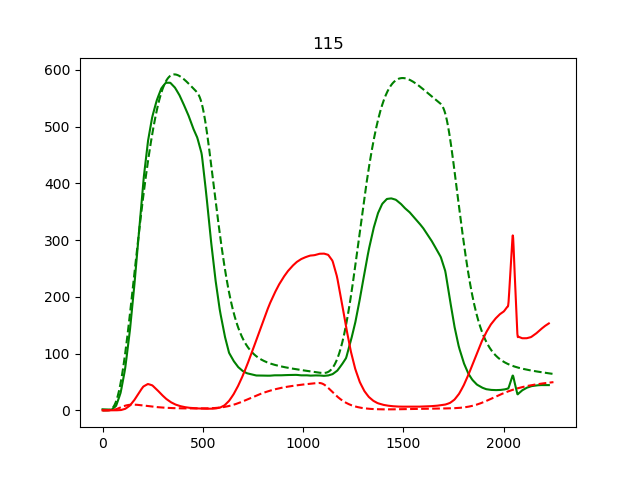

Supplement: Supplementary file 6 — Supplementary Dataset 3 [file 41467_2022_31306_MOESM6_ESM.zip › Individual Simulations Bistable Switch/115.png]

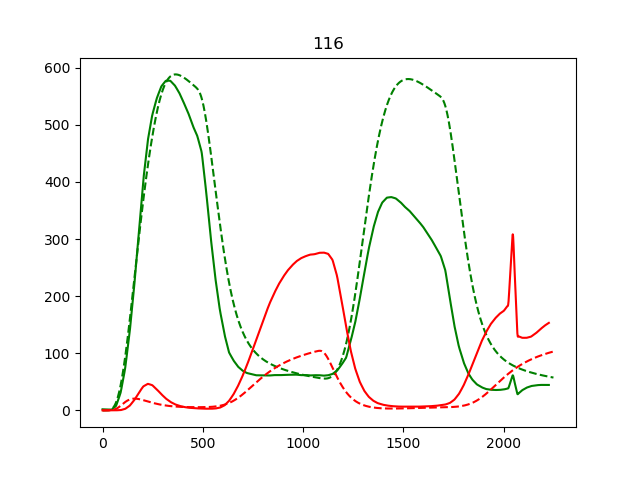

Supplement: Supplementary file 6 — Supplementary Dataset 3 [file 41467_2022_31306_MOESM6_ESM.zip › Individual Simulations Bistable Switch/116.png]

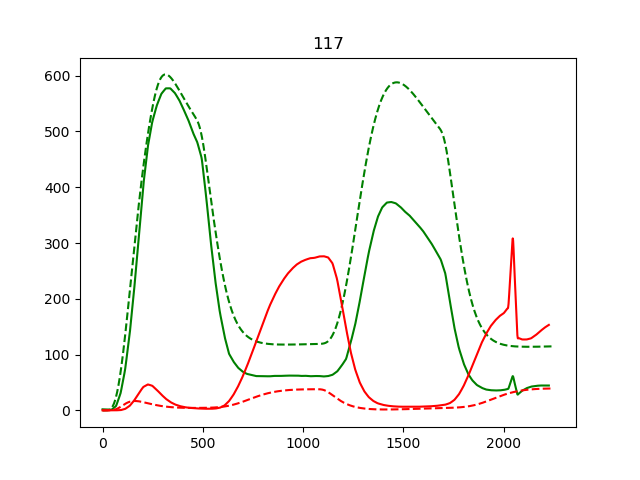

Supplement: Supplementary file 6 — Supplementary Dataset 3 [file 41467_2022_31306_MOESM6_ESM.zip › Individual Simulations Bistable Switch/117.png]

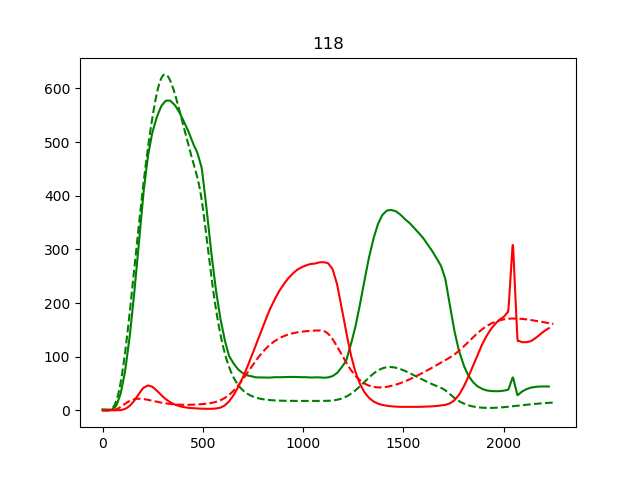

Supplement: Supplementary file 6 — Supplementary Dataset 3 [file 41467_2022_31306_MOESM6_ESM.zip › Individual Simulations Bistable Switch/118.png]

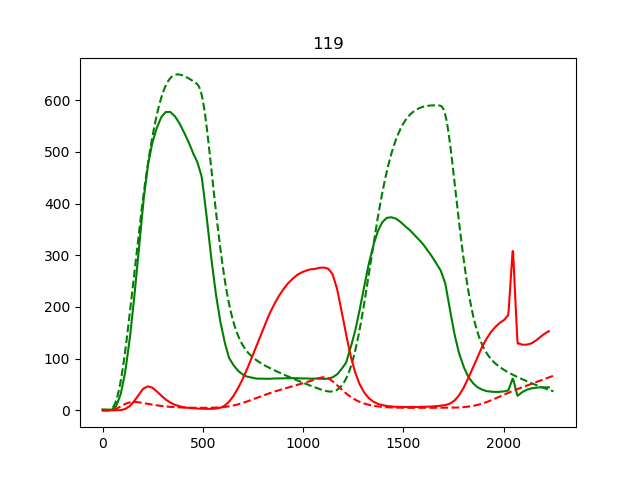

Supplement: Supplementary file 6 — Supplementary Dataset 3 [file 41467_2022_31306_MOESM6_ESM.zip › Individual Simulations Bistable Switch/119.png]

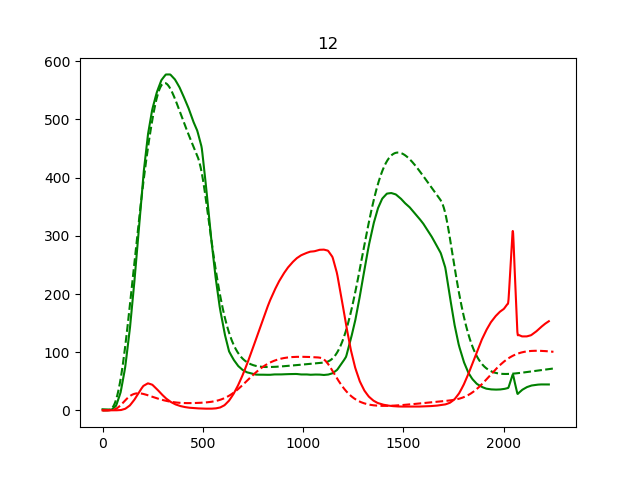

Supplement: Supplementary file 6 — Supplementary Dataset 3 [file 41467_2022_31306_MOESM6_ESM.zip › Individual Simulations Bistable Switch/12.png]

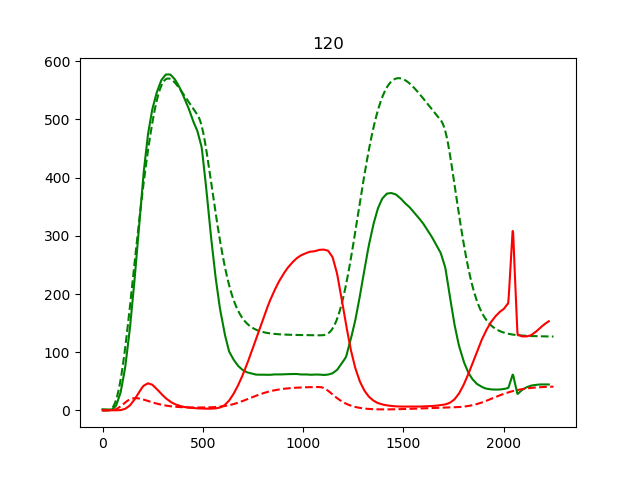

Supplement: Supplementary file 6 — Supplementary Dataset 3 [file 41467_2022_31306_MOESM6_ESM.zip › Individual Simulations Bistable Switch/120.png]

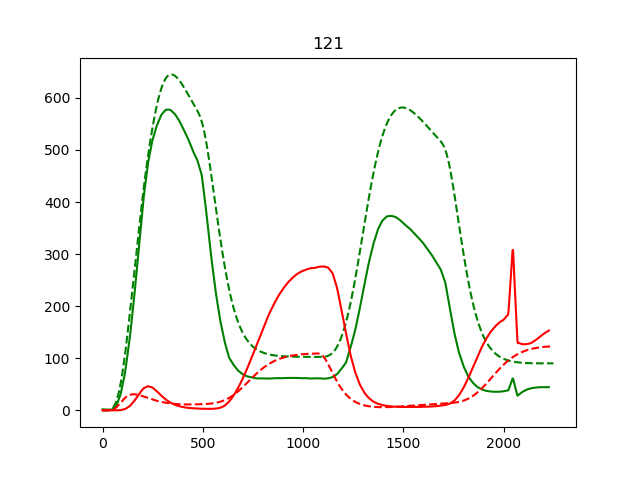

Supplement: Supplementary file 6 — Supplementary Dataset 3 [file 41467_2022_31306_MOESM6_ESM.zip › Individual Simulations Bistable Switch/121.png]

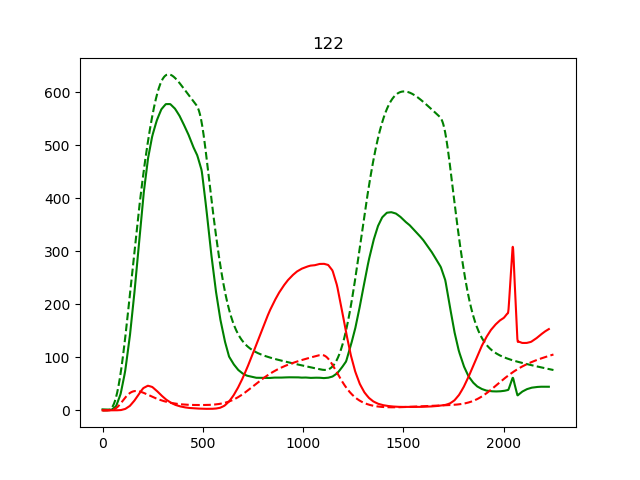

Supplement: Supplementary file 6 — Supplementary Dataset 3 [file 41467_2022_31306_MOESM6_ESM.zip › Individual Simulations Bistable Switch/122.png]

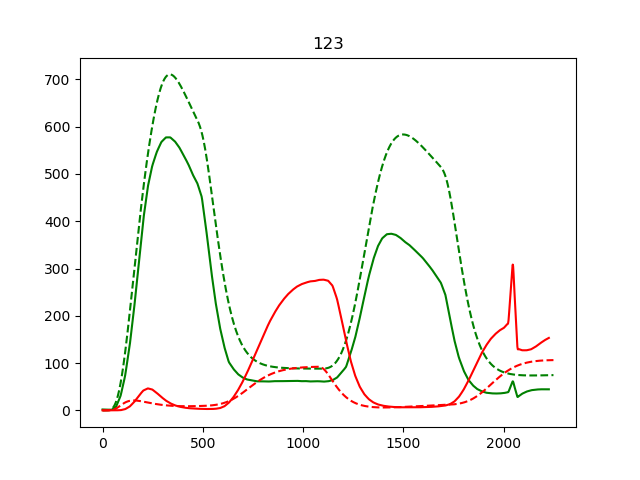

Supplement: Supplementary file 6 — Supplementary Dataset 3 [file 41467_2022_31306_MOESM6_ESM.zip › Individual Simulations Bistable Switch/123.png]

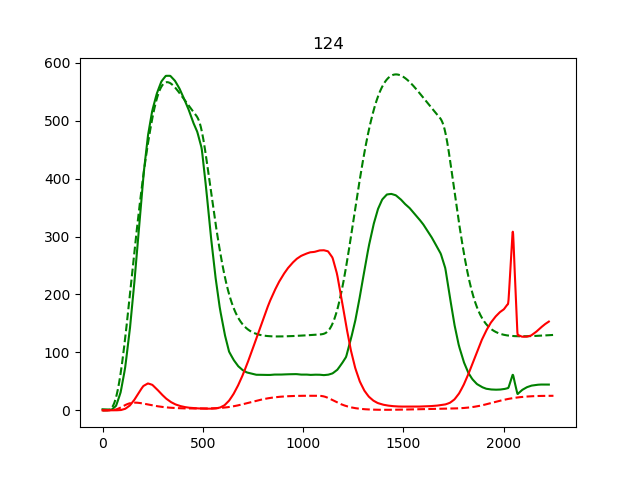

Supplement: Supplementary file 6 — Supplementary Dataset 3 [file 41467_2022_31306_MOESM6_ESM.zip › Individual Simulations Bistable Switch/124.png]

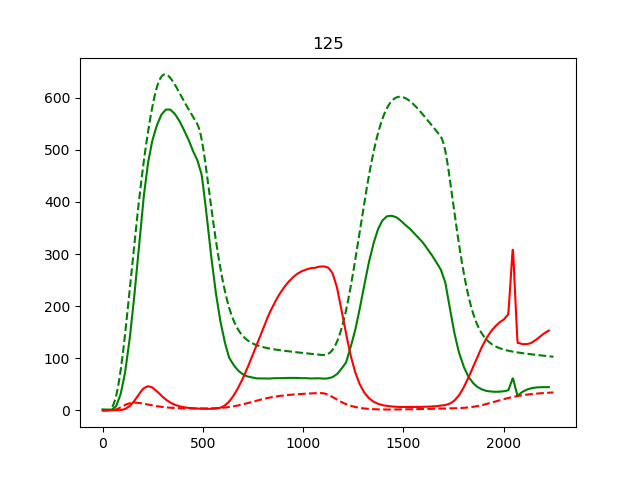

Supplement: Supplementary file 6 — Supplementary Dataset 3 [file 41467_2022_31306_MOESM6_ESM.zip › Individual Simulations Bistable Switch/125.png]

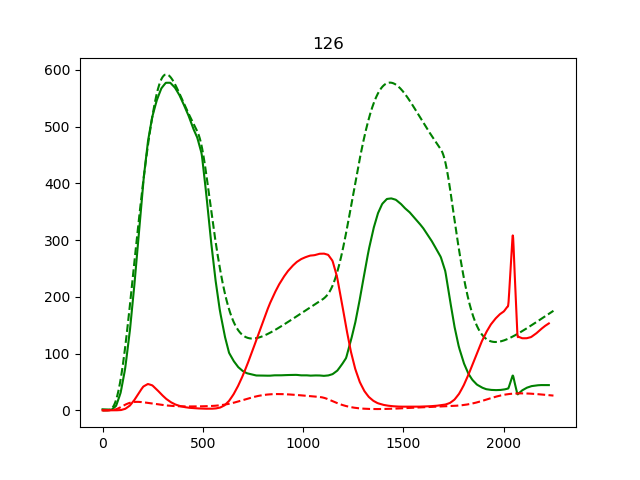

Supplement: Supplementary file 6 — Supplementary Dataset 3 [file 41467_2022_31306_MOESM6_ESM.zip › Individual Simulations Bistable Switch/126.png]

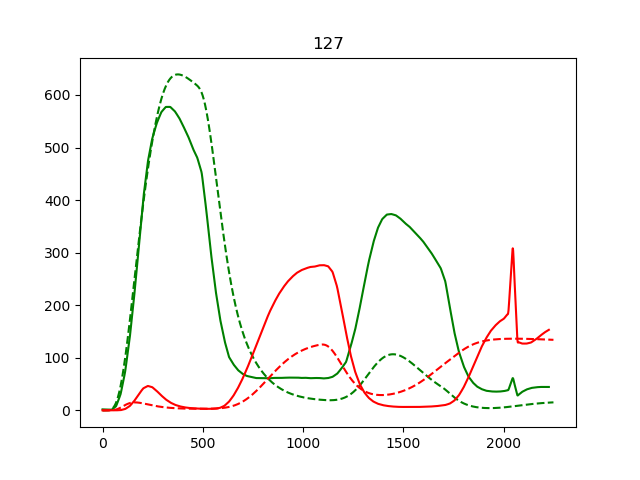

Supplement: Supplementary file 6 — Supplementary Dataset 3 [file 41467_2022_31306_MOESM6_ESM.zip › Individual Simulations Bistable Switch/127.png]

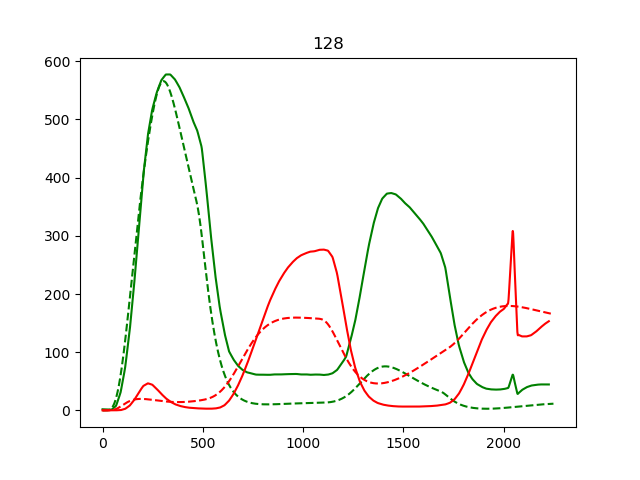

Supplement: Supplementary file 6 — Supplementary Dataset 3 [file 41467_2022_31306_MOESM6_ESM.zip › Individual Simulations Bistable Switch/128.png]

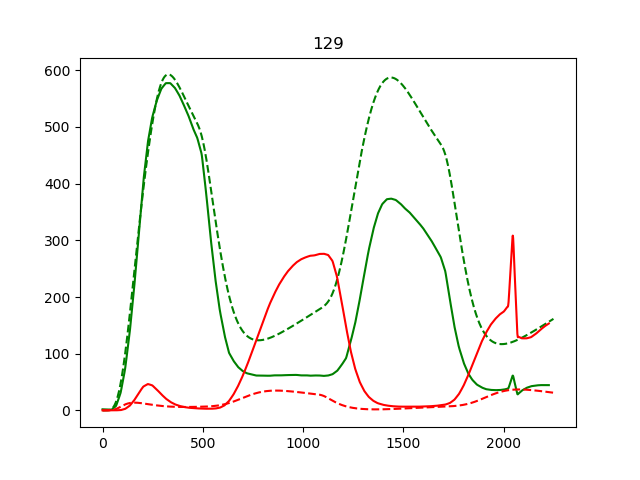

Supplement: Supplementary file 6 — Supplementary Dataset 3 [file 41467_2022_31306_MOESM6_ESM.zip › Individual Simulations Bistable Switch/129.png]

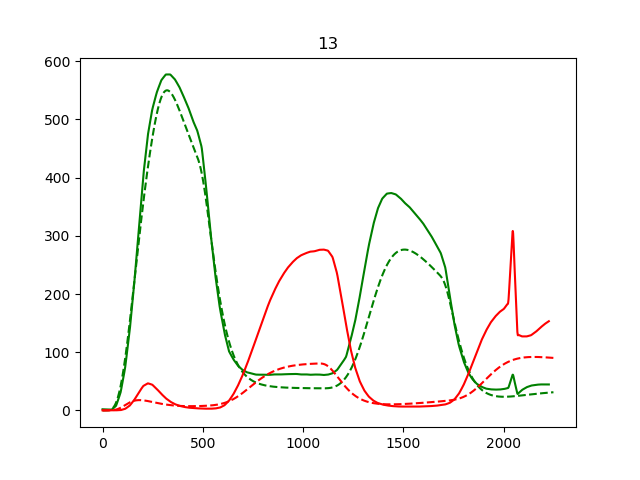

Supplement: Supplementary file 6 — Supplementary Dataset 3 [file 41467_2022_31306_MOESM6_ESM.zip › Individual Simulations Bistable Switch/13.png]

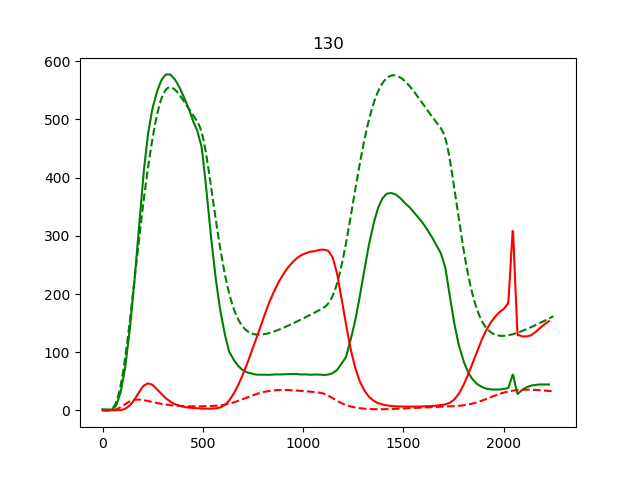

Supplement: Supplementary file 6 — Supplementary Dataset 3 [file 41467_2022_31306_MOESM6_ESM.zip › Individual Simulations Bistable Switch/130.png]

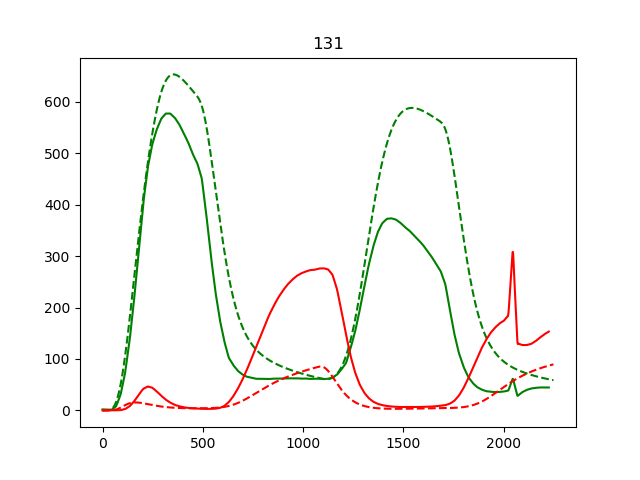

Supplement: Supplementary file 6 — Supplementary Dataset 3 [file 41467_2022_31306_MOESM6_ESM.zip › Individual Simulations Bistable Switch/131.png]

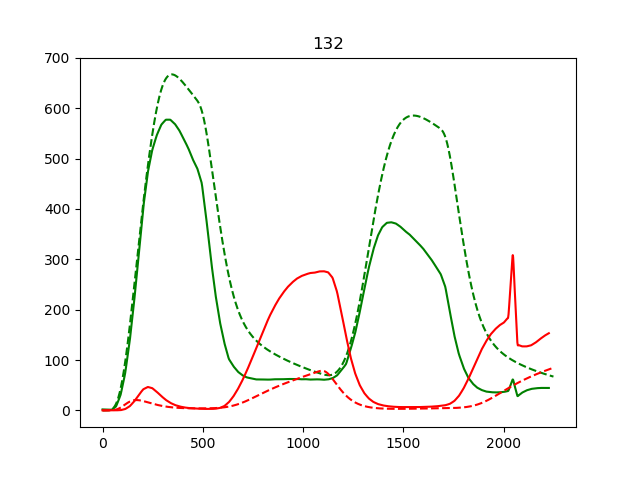

Supplement: Supplementary file 6 — Supplementary Dataset 3 [file 41467_2022_31306_MOESM6_ESM.zip › Individual Simulations Bistable Switch/132.png]

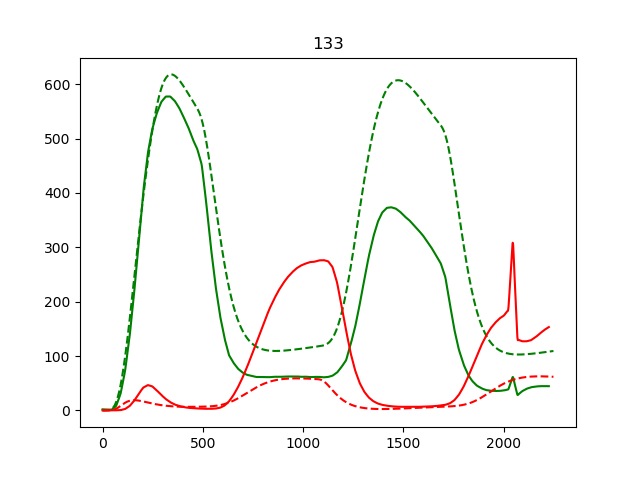

Supplement: Supplementary file 6 — Supplementary Dataset 3 [file 41467_2022_31306_MOESM6_ESM.zip › Individual Simulations Bistable Switch/133.png]

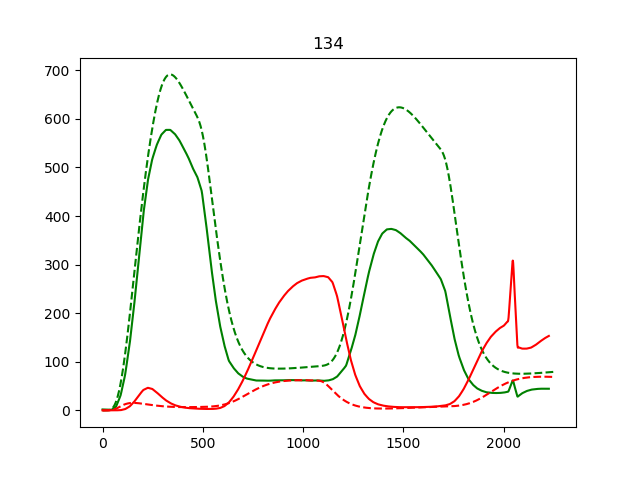

Supplement: Supplementary file 6 — Supplementary Dataset 3 [file 41467_2022_31306_MOESM6_ESM.zip › Individual Simulations Bistable Switch/134.png]

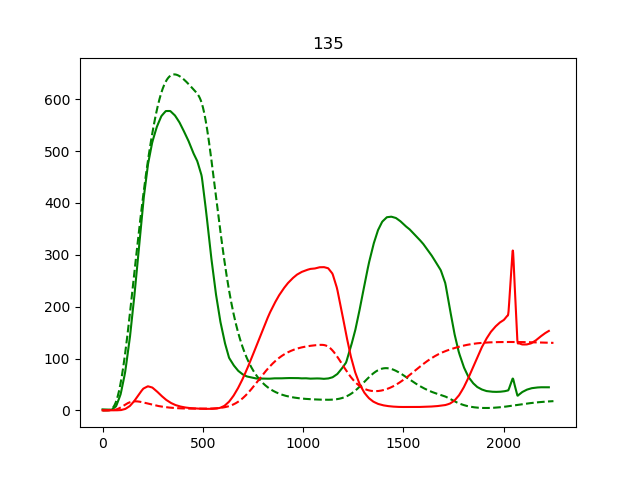

Supplement: Supplementary file 6 — Supplementary Dataset 3 [file 41467_2022_31306_MOESM6_ESM.zip › Individual Simulations Bistable Switch/135.png]

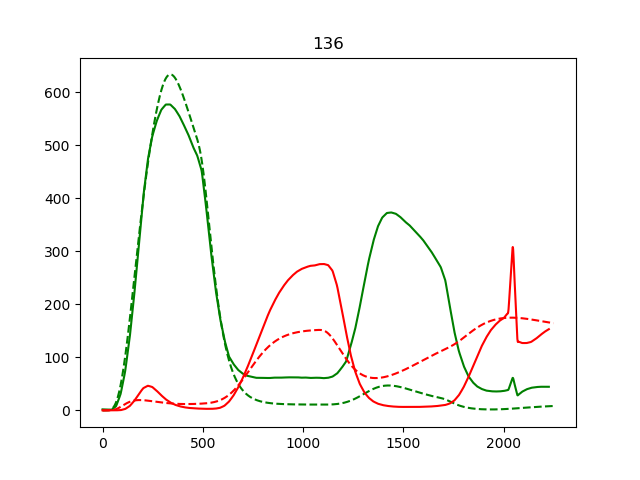

Supplement: Supplementary file 6 — Supplementary Dataset 3 [file 41467_2022_31306_MOESM6_ESM.zip › Individual Simulations Bistable Switch/136.png]

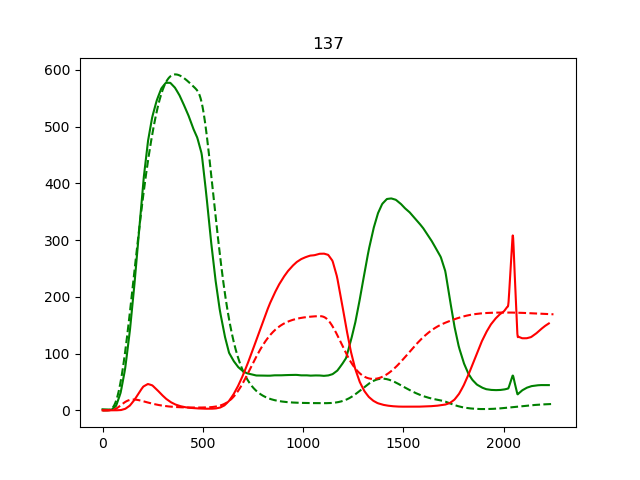

Supplement: Supplementary file 6 — Supplementary Dataset 3 [file 41467_2022_31306_MOESM6_ESM.zip › Individual Simulations Bistable Switch/137.png]

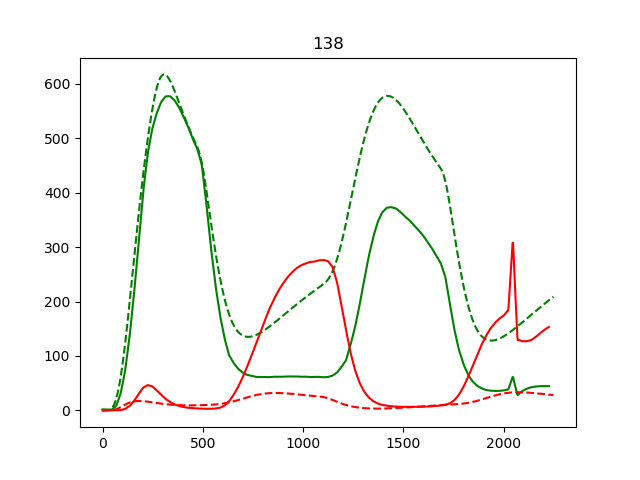

Supplement: Supplementary file 6 — Supplementary Dataset 3 [file 41467_2022_31306_MOESM6_ESM.zip › Individual Simulations Bistable Switch/138.png]

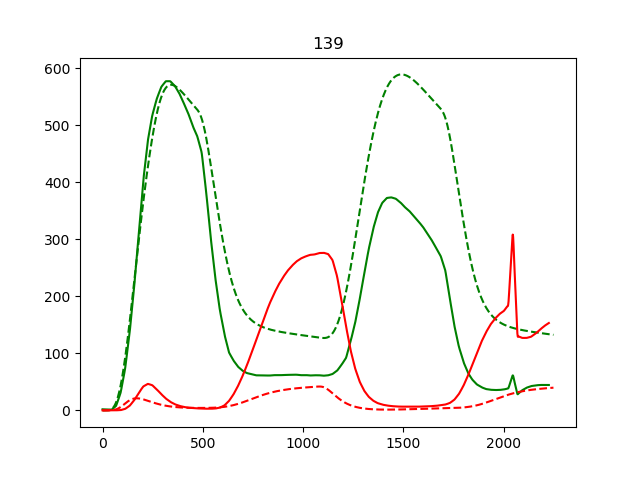

Supplement: Supplementary file 6 — Supplementary Dataset 3 [file 41467_2022_31306_MOESM6_ESM.zip › Individual Simulations Bistable Switch/139.png]

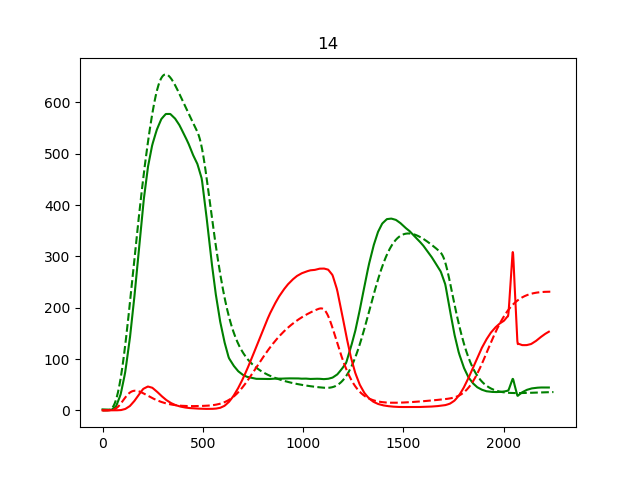

Supplement: Supplementary file 6 — Supplementary Dataset 3 [file 41467_2022_31306_MOESM6_ESM.zip › Individual Simulations Bistable Switch/14.png]

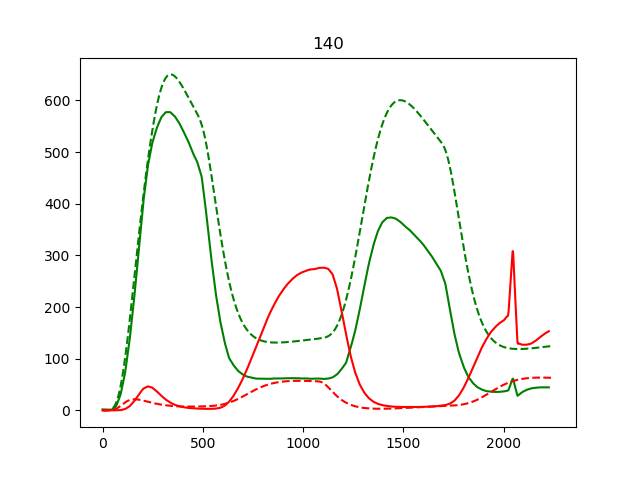

Supplement: Supplementary file 6 — Supplementary Dataset 3 [file 41467_2022_31306_MOESM6_ESM.zip › Individual Simulations Bistable Switch/140.png]

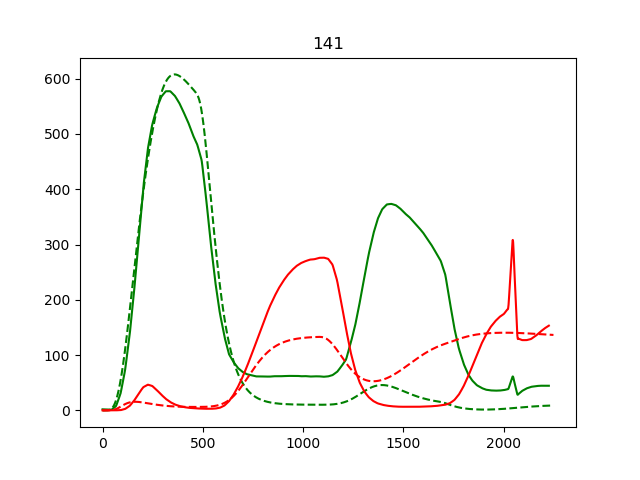

Supplement: Supplementary file 6 — Supplementary Dataset 3 [file 41467_2022_31306_MOESM6_ESM.zip › Individual Simulations Bistable Switch/141.png]
