# Supplementary figures and images for: A microfluidic optimal experimental design platform for forward design of cell-free genetic networks (part 1 of 4)
Source: Nat Commun. 2022 Jun 24;13:3626. doi: 10.1038/s41467-022-31306-3 (PMC9232554; doi:10.1038/s41467-022-31306-3)

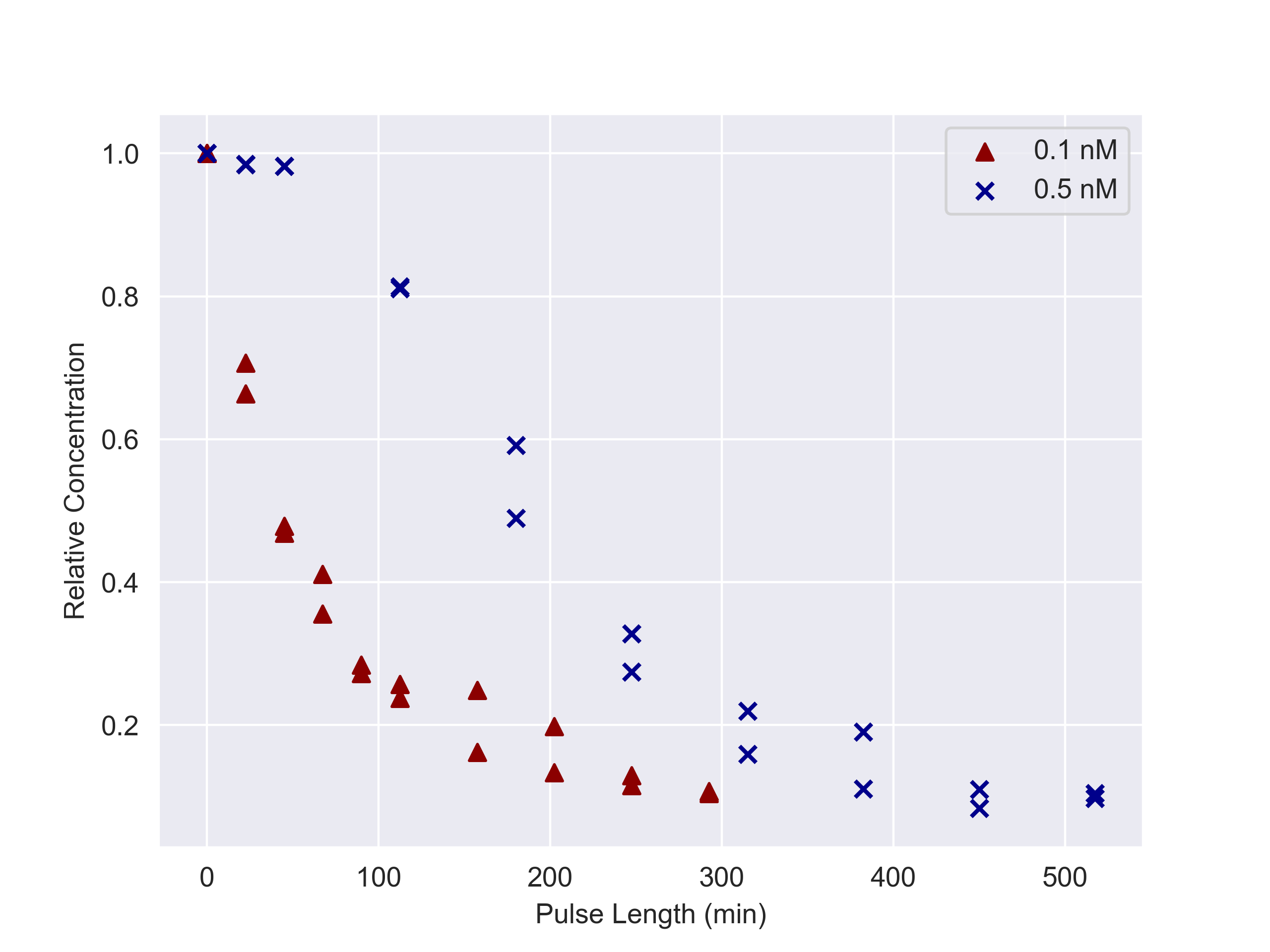

Supplement: Supplementary file 5 — Supplementary Dataset 2 [file 41467_2022_31306_MOESM5_ESM.zip › Individual Simulations Pulse Decoder/0.png]

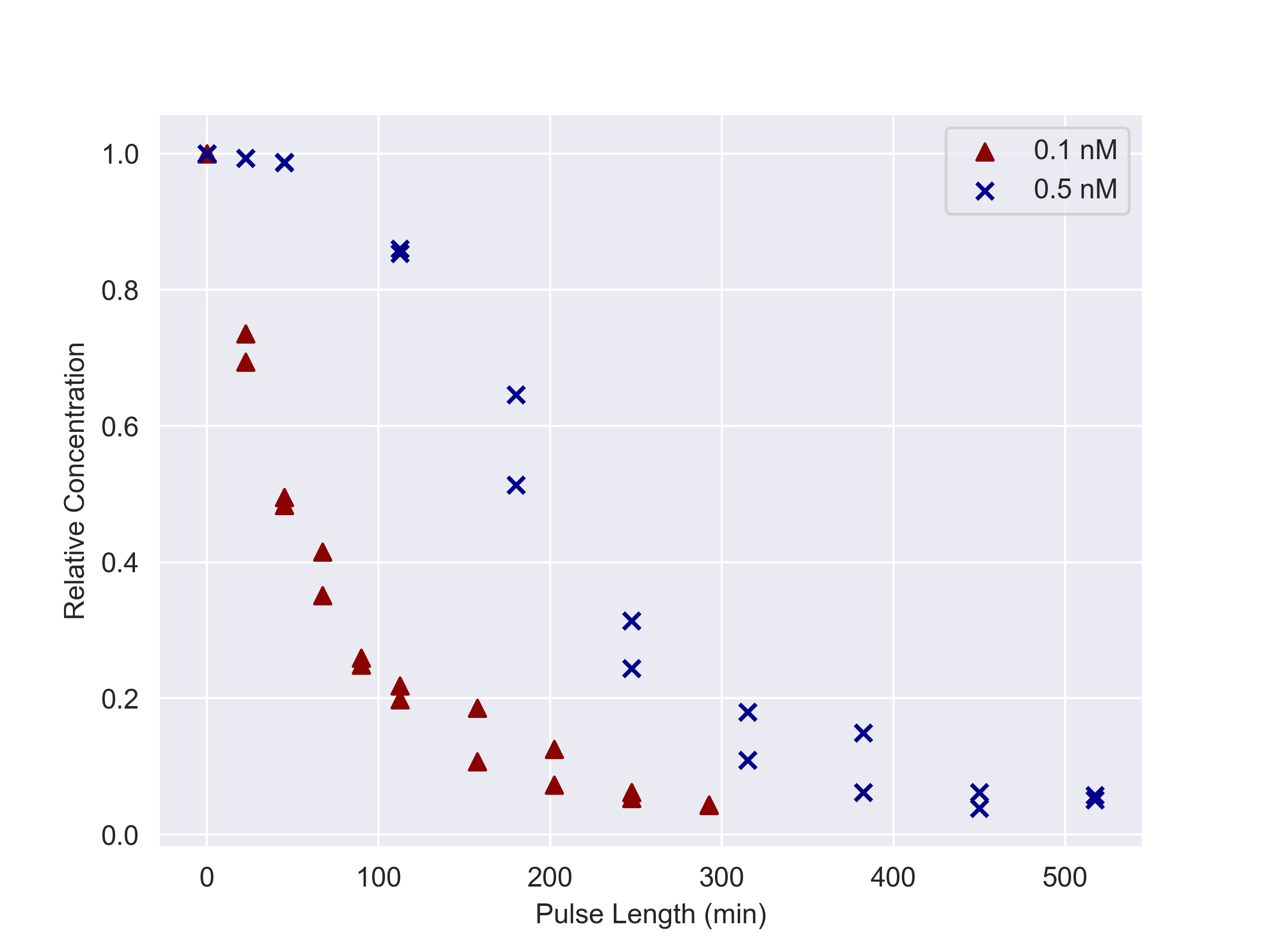

Supplement: Supplementary file 5 — Supplementary Dataset 2 [file 41467_2022_31306_MOESM5_ESM.zip › Individual Simulations Pulse Decoder/1.png]

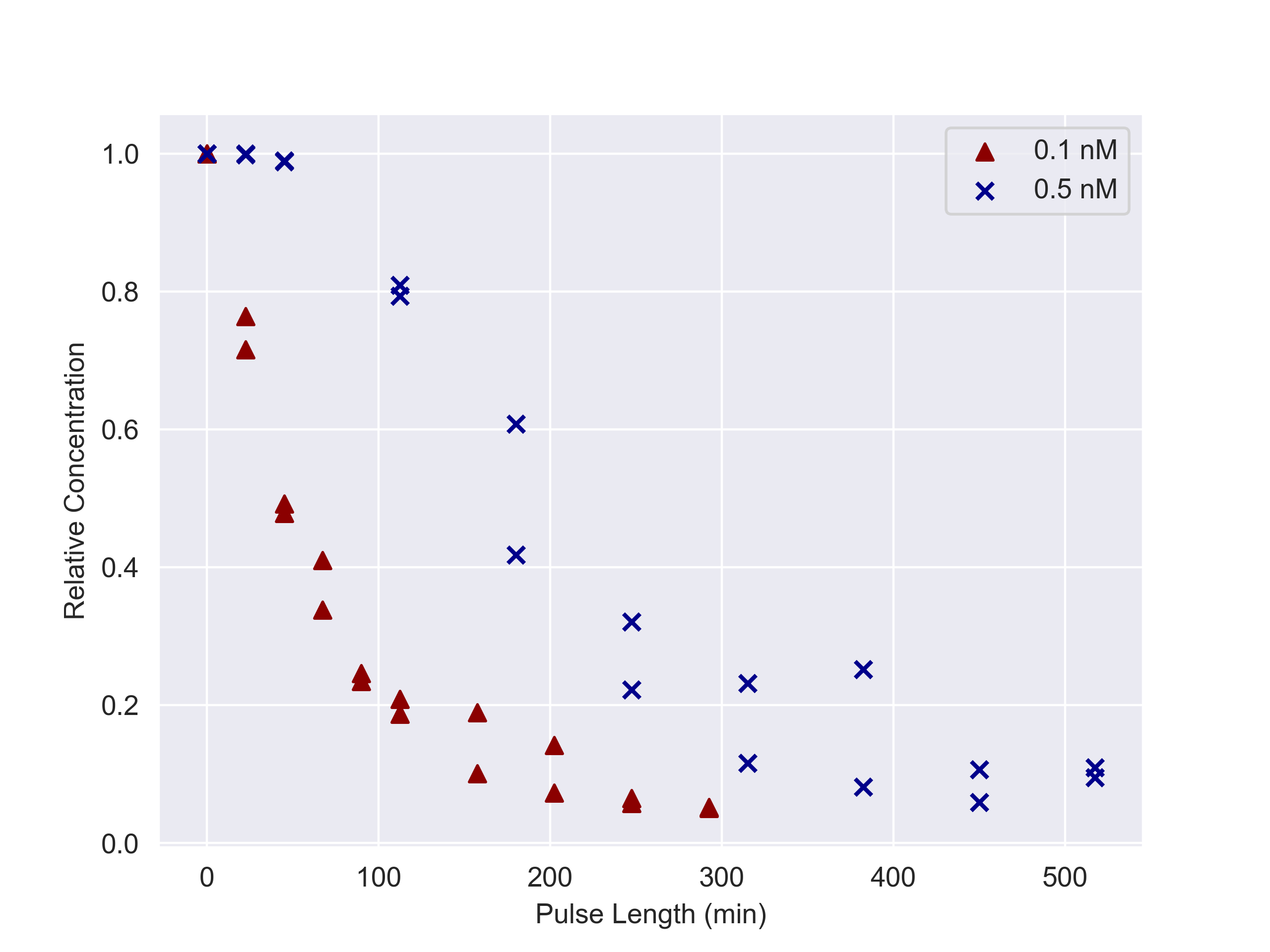

Supplement: Supplementary file 5 — Supplementary Dataset 2 [file 41467_2022_31306_MOESM5_ESM.zip › Individual Simulations Pulse Decoder/10.png]

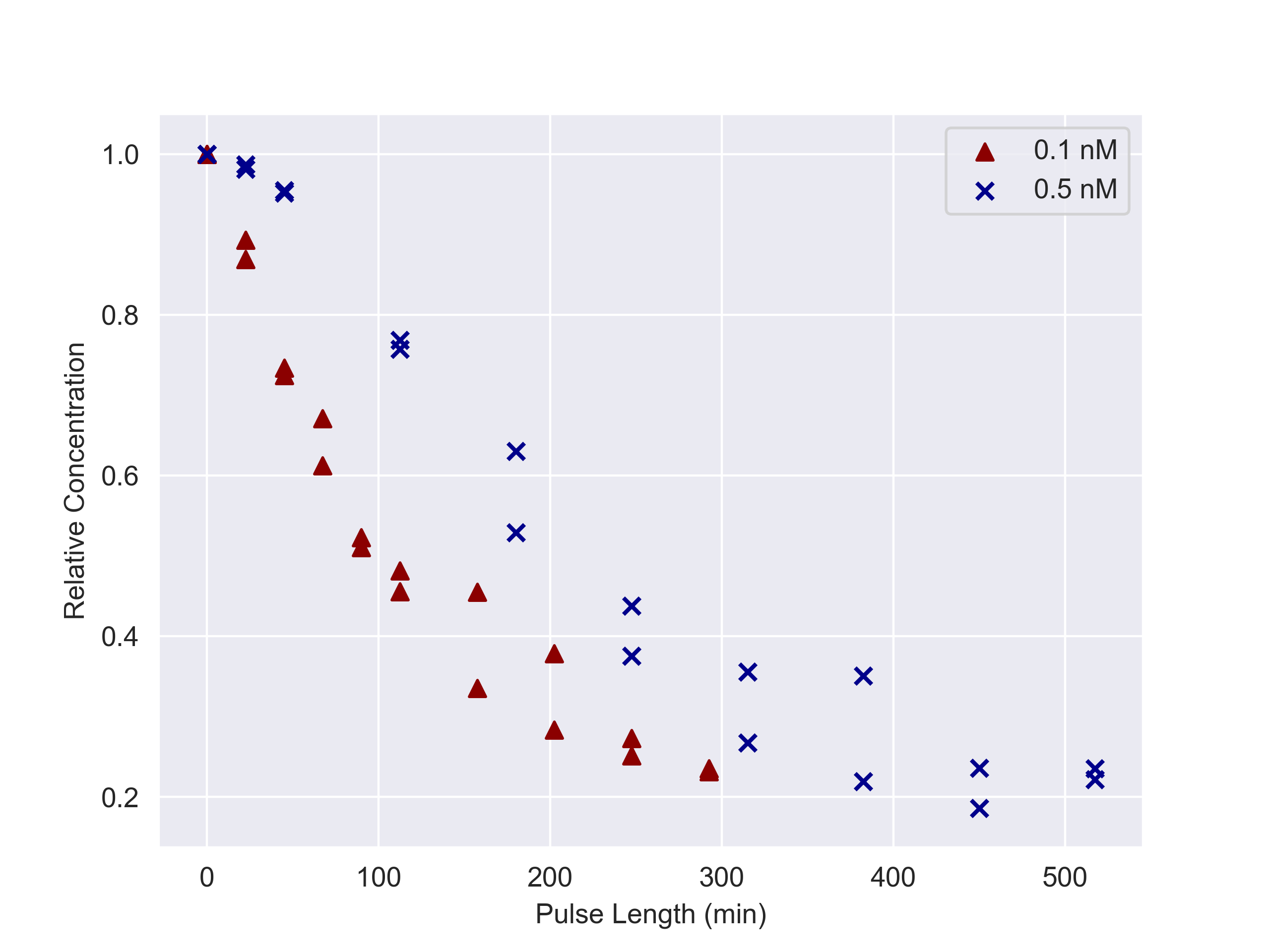

Supplement: Supplementary file 5 — Supplementary Dataset 2 [file 41467_2022_31306_MOESM5_ESM.zip › Individual Simulations Pulse Decoder/100.png]

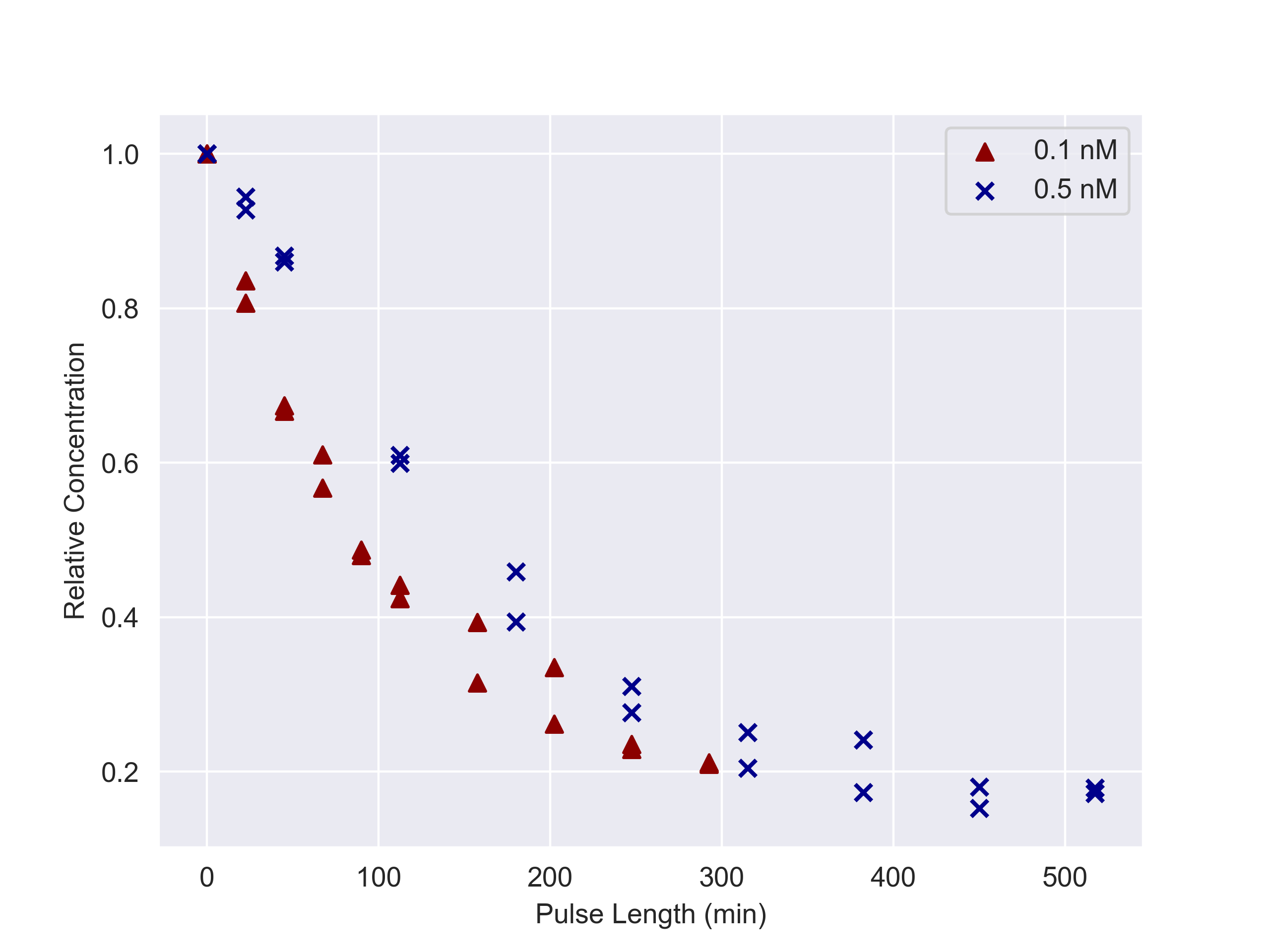

Supplement: Supplementary file 5 — Supplementary Dataset 2 [file 41467_2022_31306_MOESM5_ESM.zip › Individual Simulations Pulse Decoder/101.png]

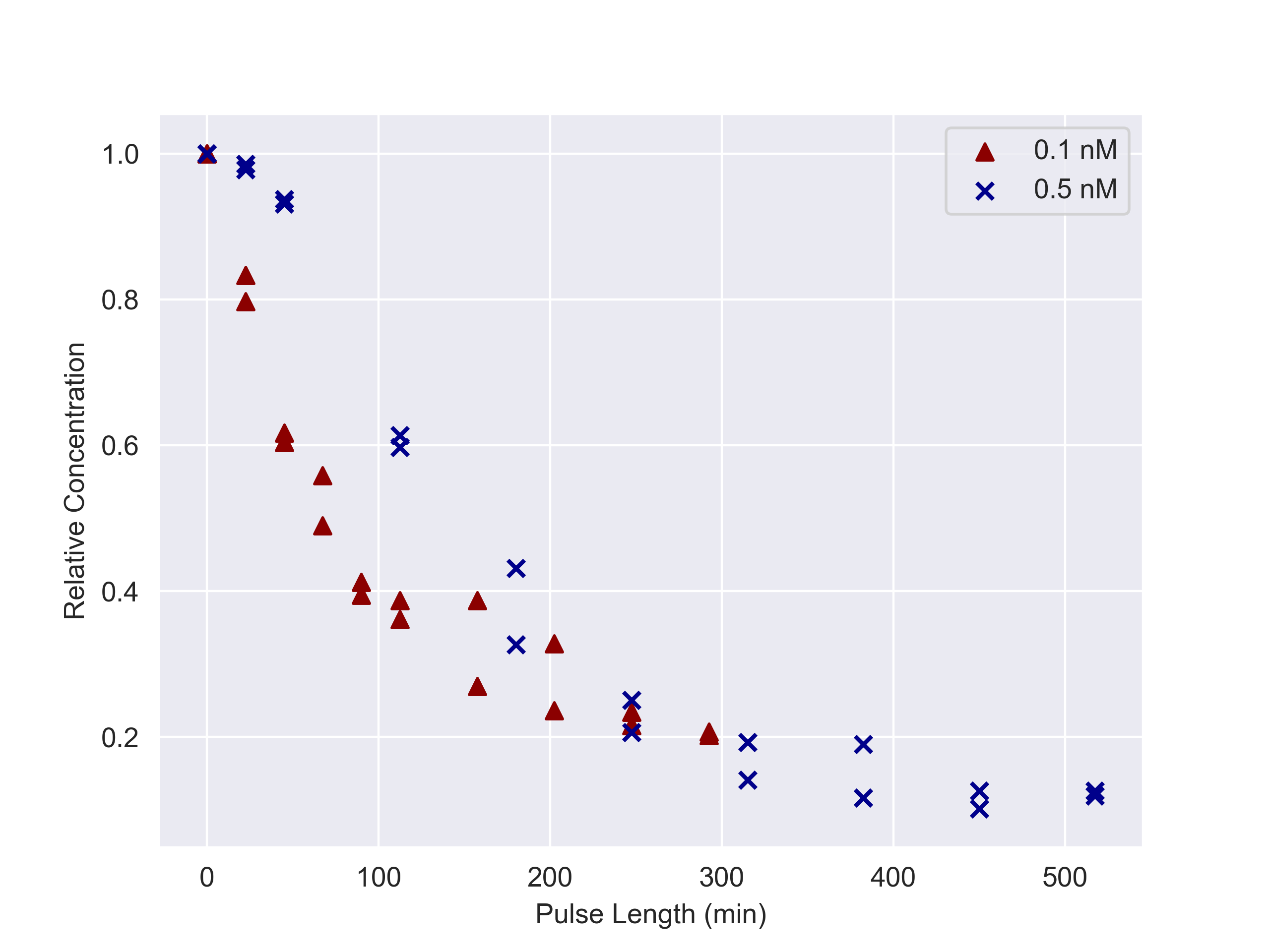

Supplement: Supplementary file 5 — Supplementary Dataset 2 [file 41467_2022_31306_MOESM5_ESM.zip › Individual Simulations Pulse Decoder/102.png]

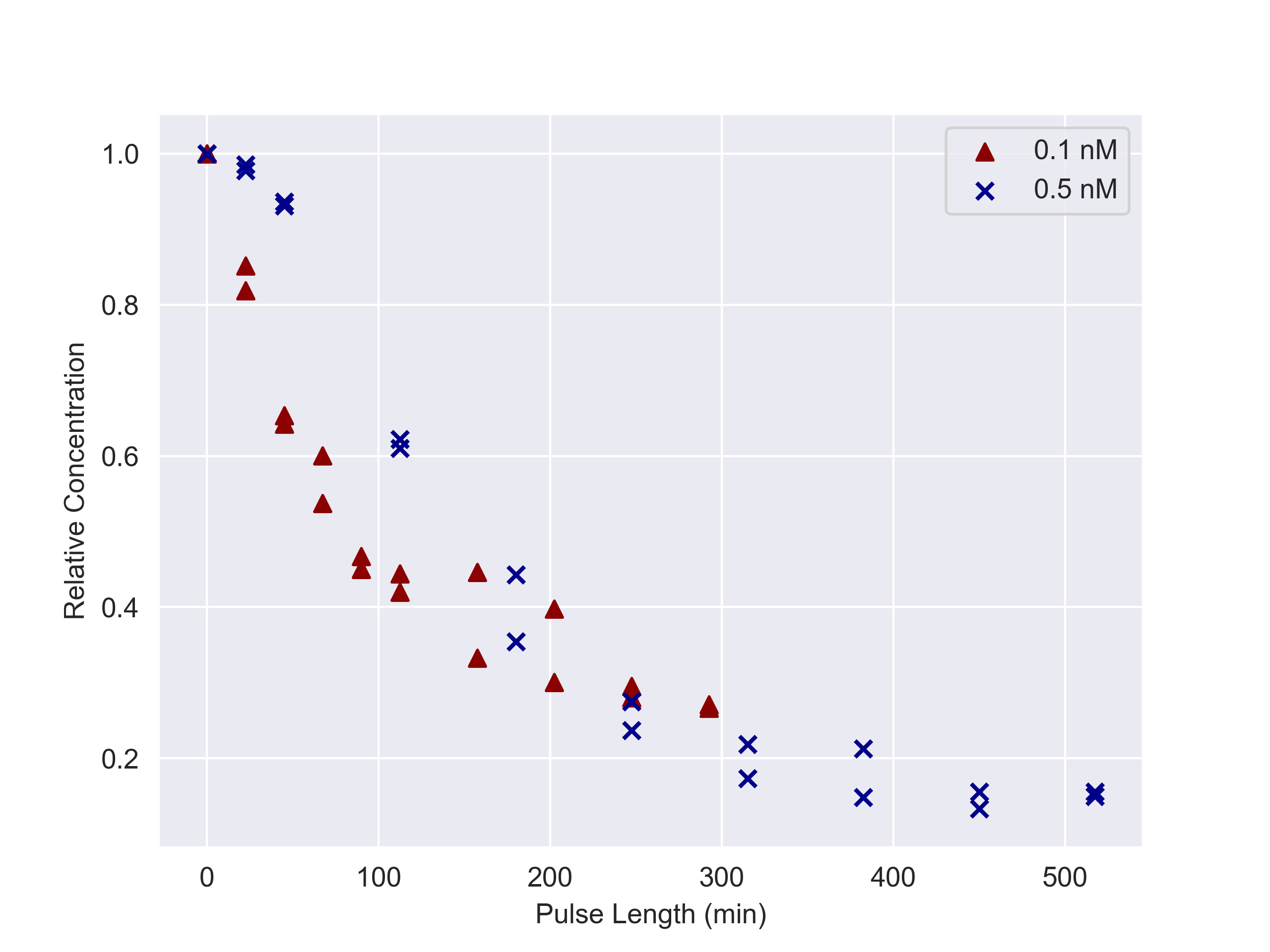

Supplement: Supplementary file 5 — Supplementary Dataset 2 [file 41467_2022_31306_MOESM5_ESM.zip › Individual Simulations Pulse Decoder/103.png]

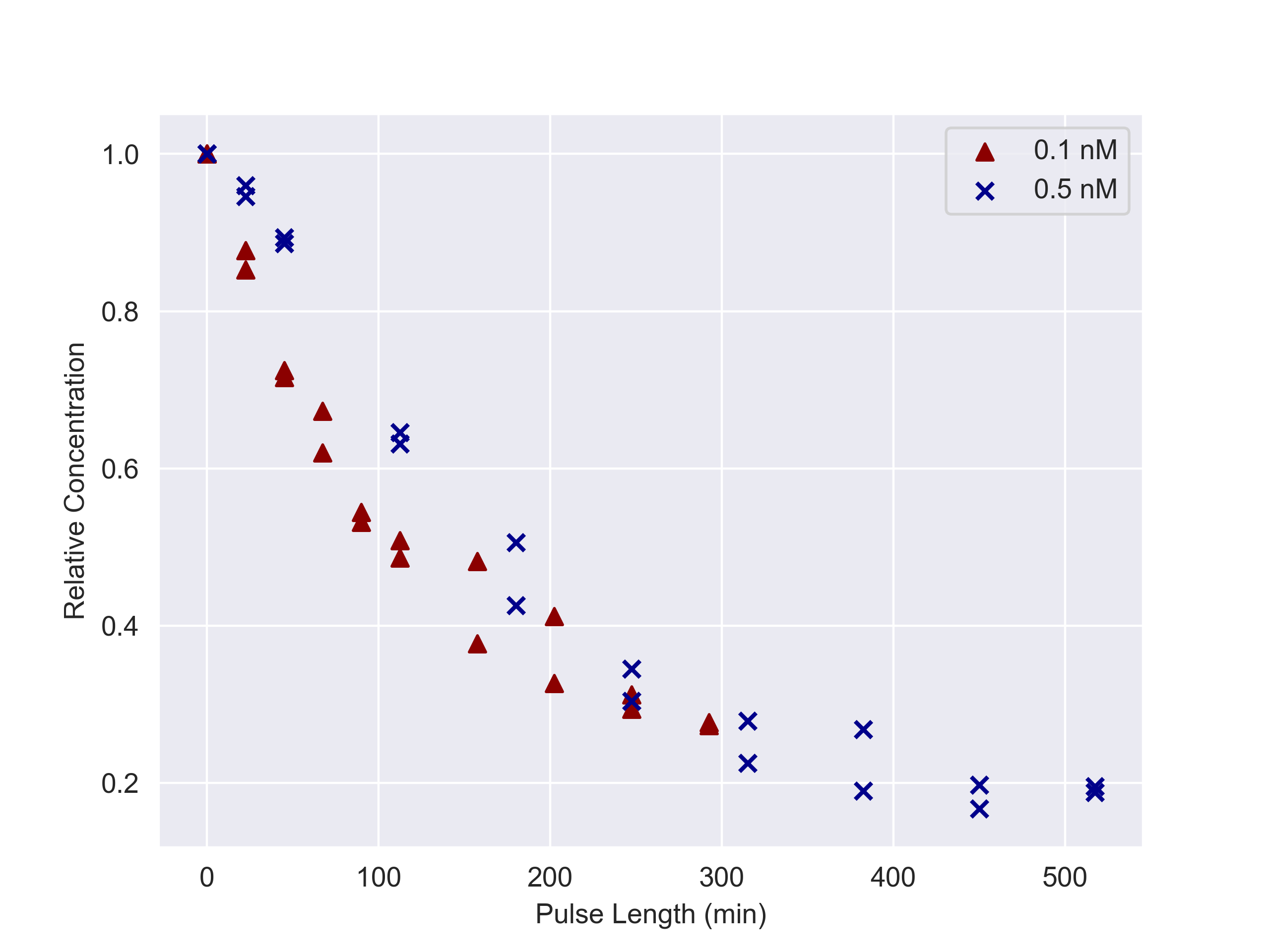

Supplement: Supplementary file 5 — Supplementary Dataset 2 [file 41467_2022_31306_MOESM5_ESM.zip › Individual Simulations Pulse Decoder/104.png]

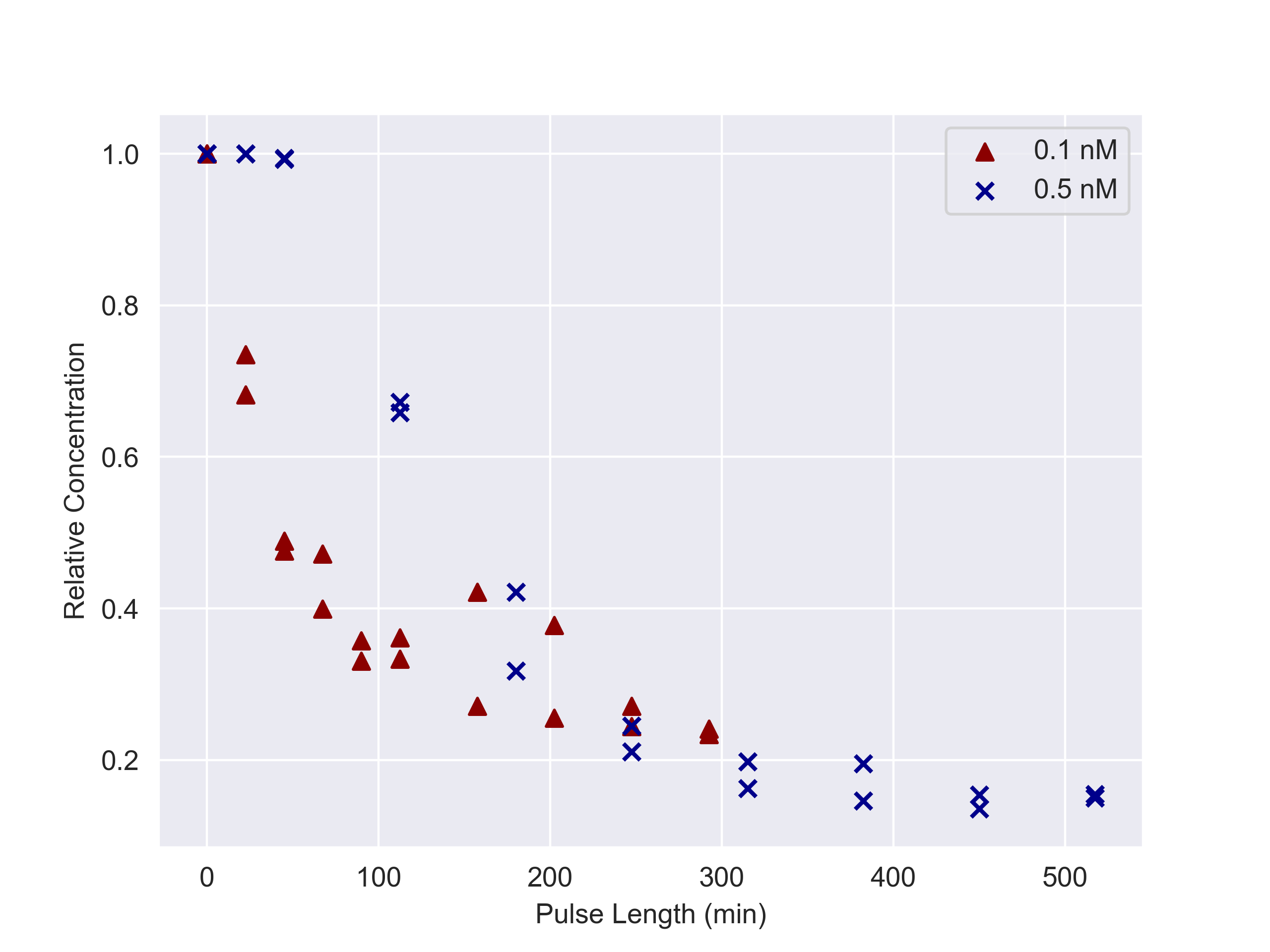

Supplement: Supplementary file 5 — Supplementary Dataset 2 [file 41467_2022_31306_MOESM5_ESM.zip › Individual Simulations Pulse Decoder/105.png]

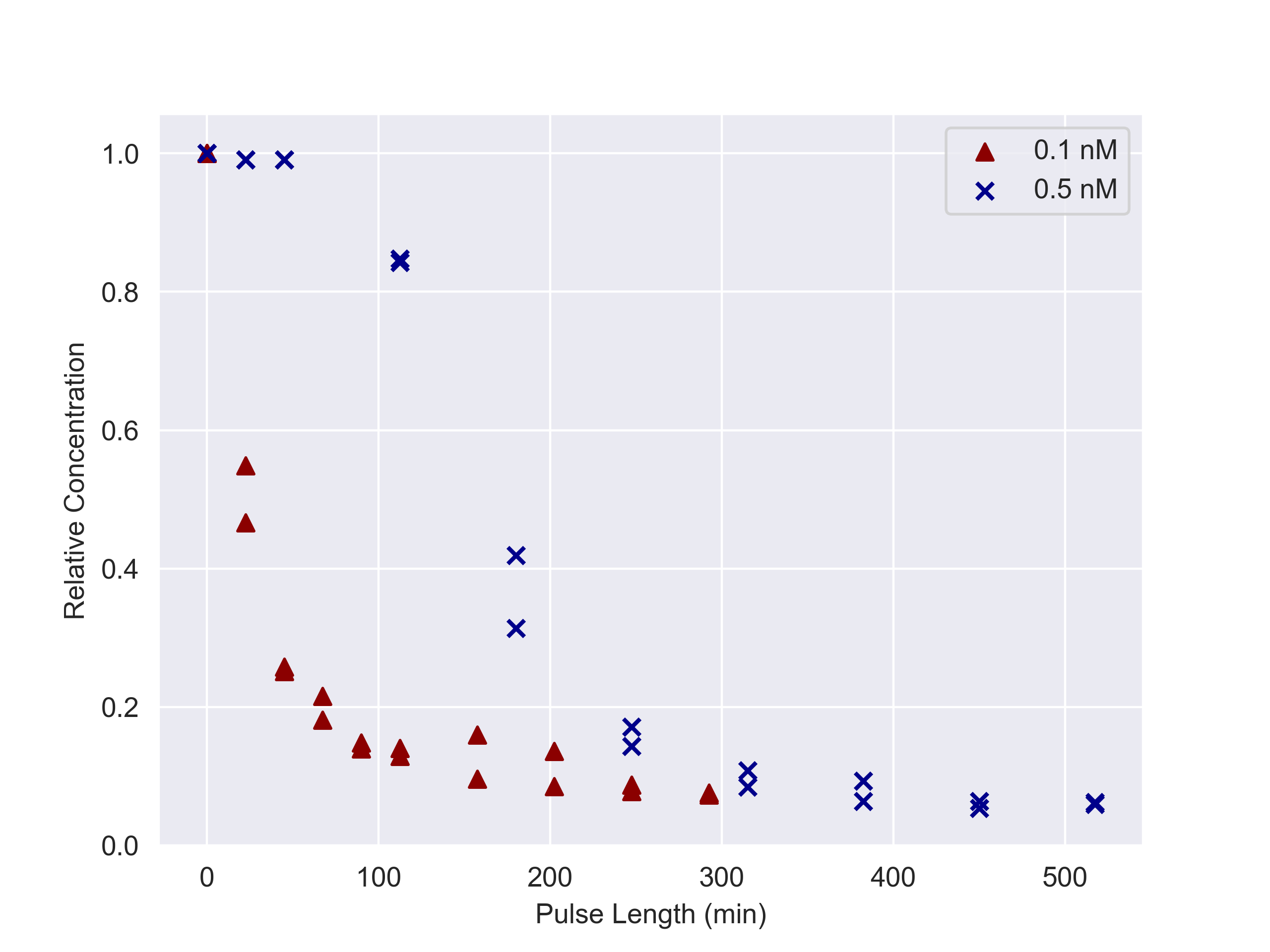

Supplement: Supplementary file 5 — Supplementary Dataset 2 [file 41467_2022_31306_MOESM5_ESM.zip › Individual Simulations Pulse Decoder/106.png]

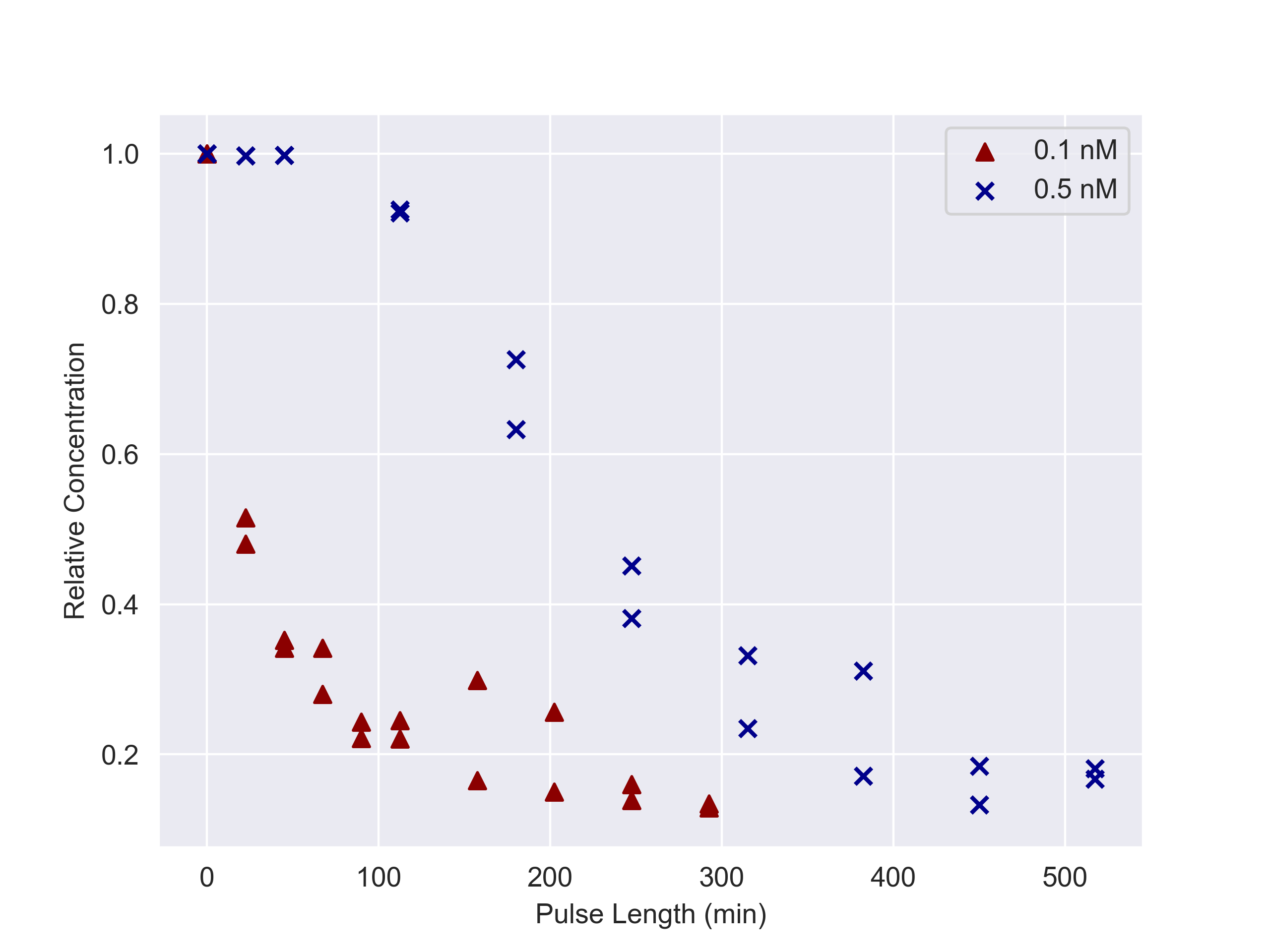

Supplement: Supplementary file 5 — Supplementary Dataset 2 [file 41467_2022_31306_MOESM5_ESM.zip › Individual Simulations Pulse Decoder/107.png]

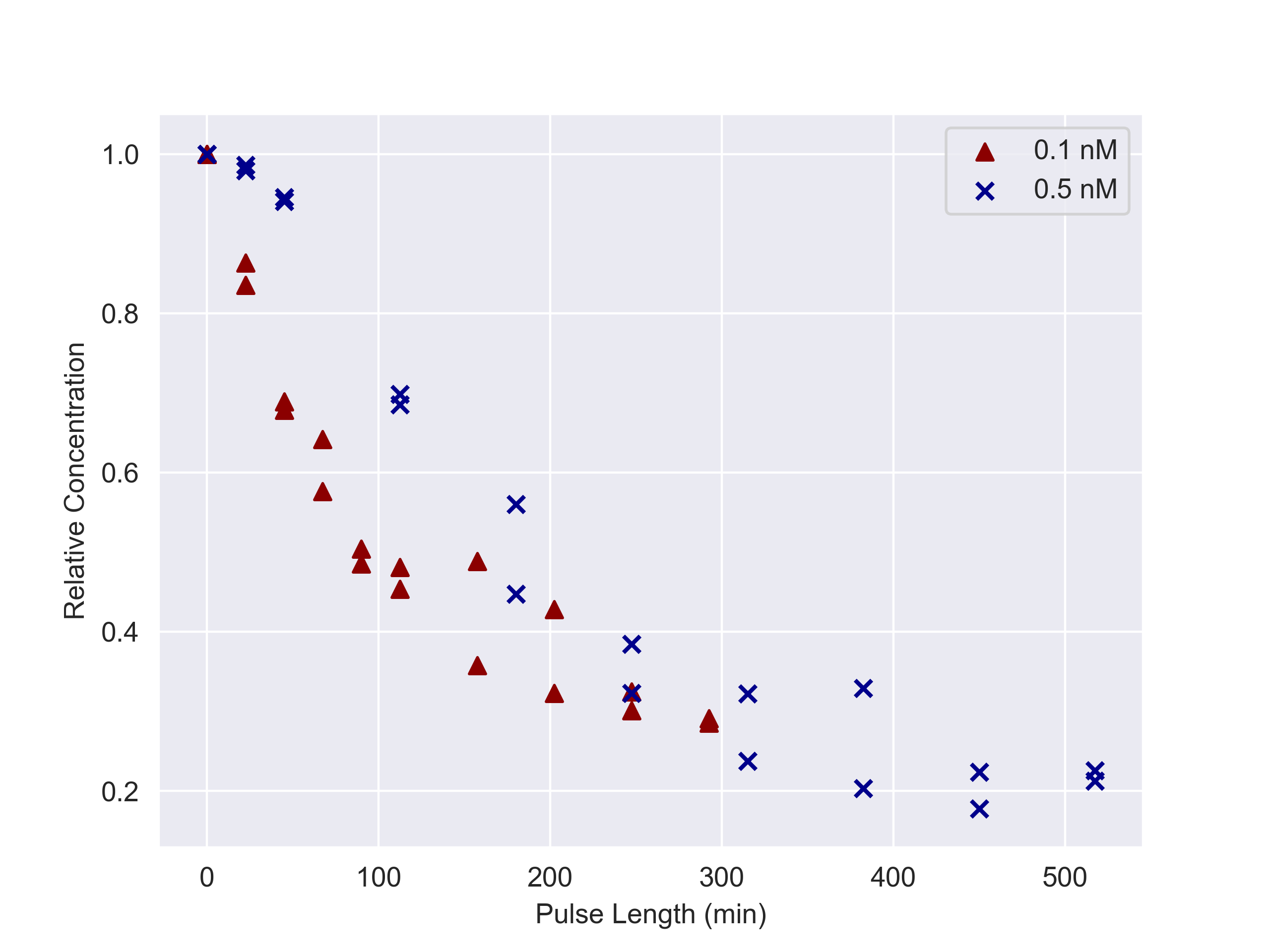

Supplement: Supplementary file 5 — Supplementary Dataset 2 [file 41467_2022_31306_MOESM5_ESM.zip › Individual Simulations Pulse Decoder/108.png]

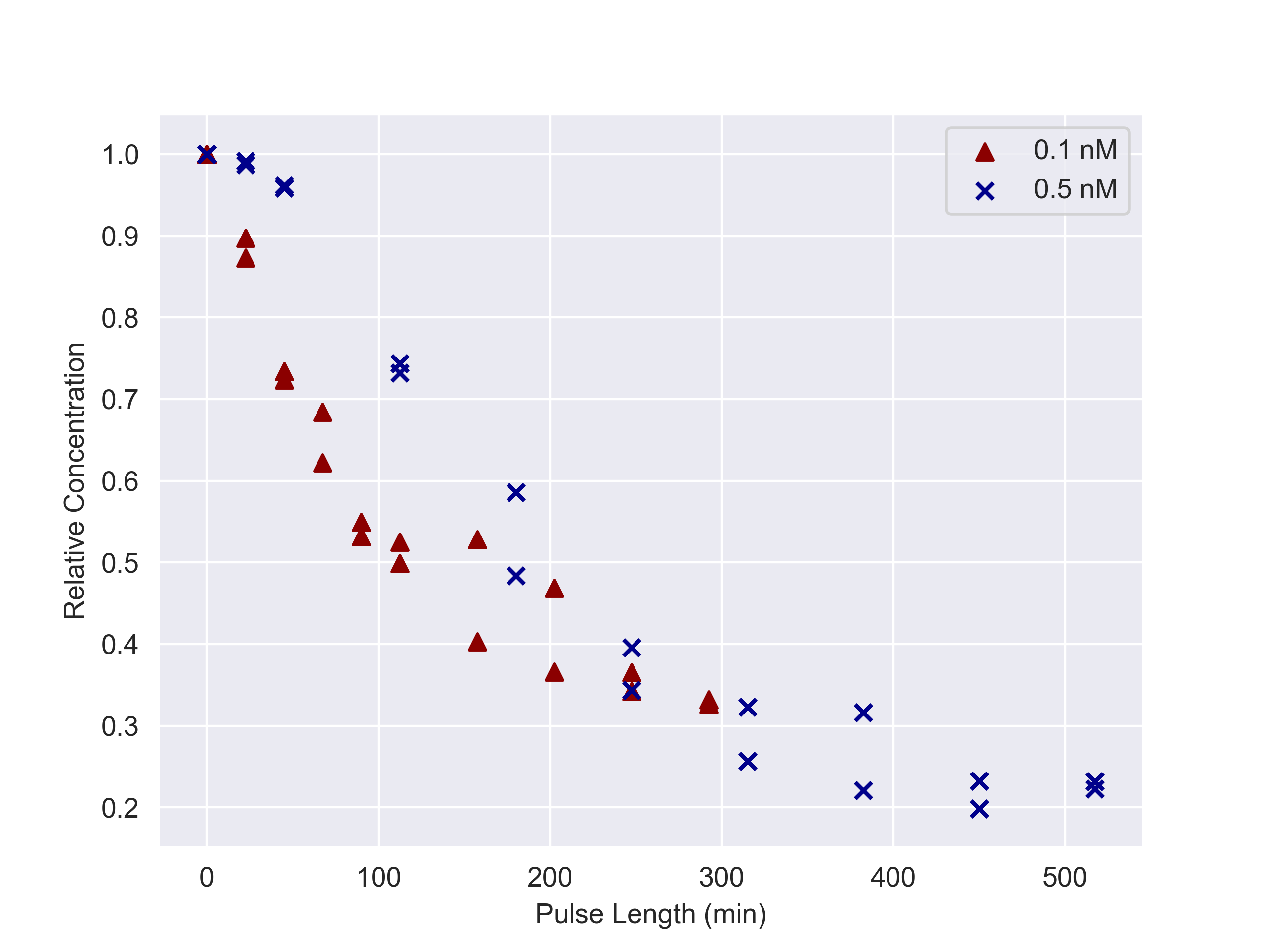

Supplement: Supplementary file 5 — Supplementary Dataset 2 [file 41467_2022_31306_MOESM5_ESM.zip › Individual Simulations Pulse Decoder/109.png]

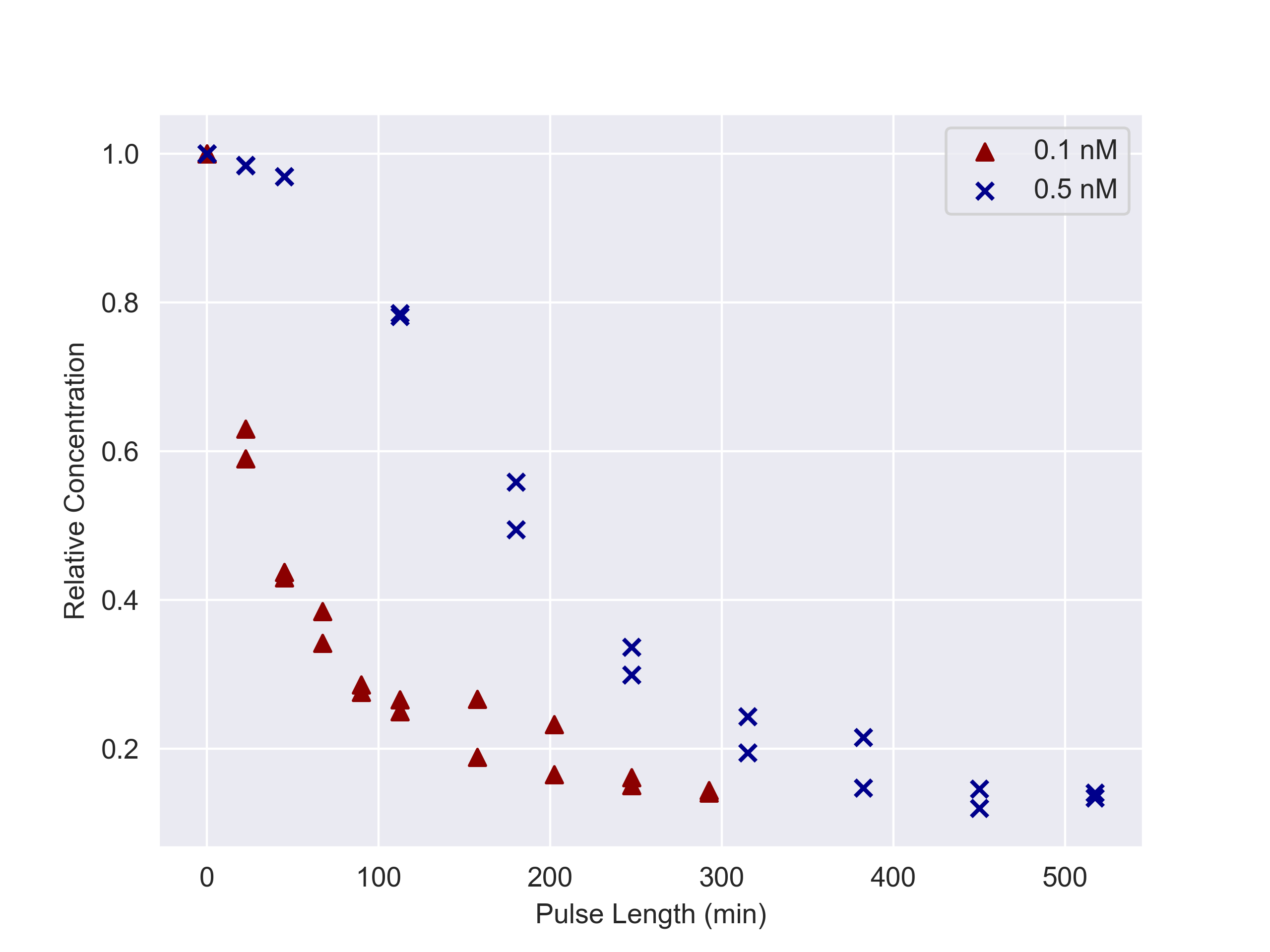

Supplement: Supplementary file 5 — Supplementary Dataset 2 [file 41467_2022_31306_MOESM5_ESM.zip › Individual Simulations Pulse Decoder/11.png]

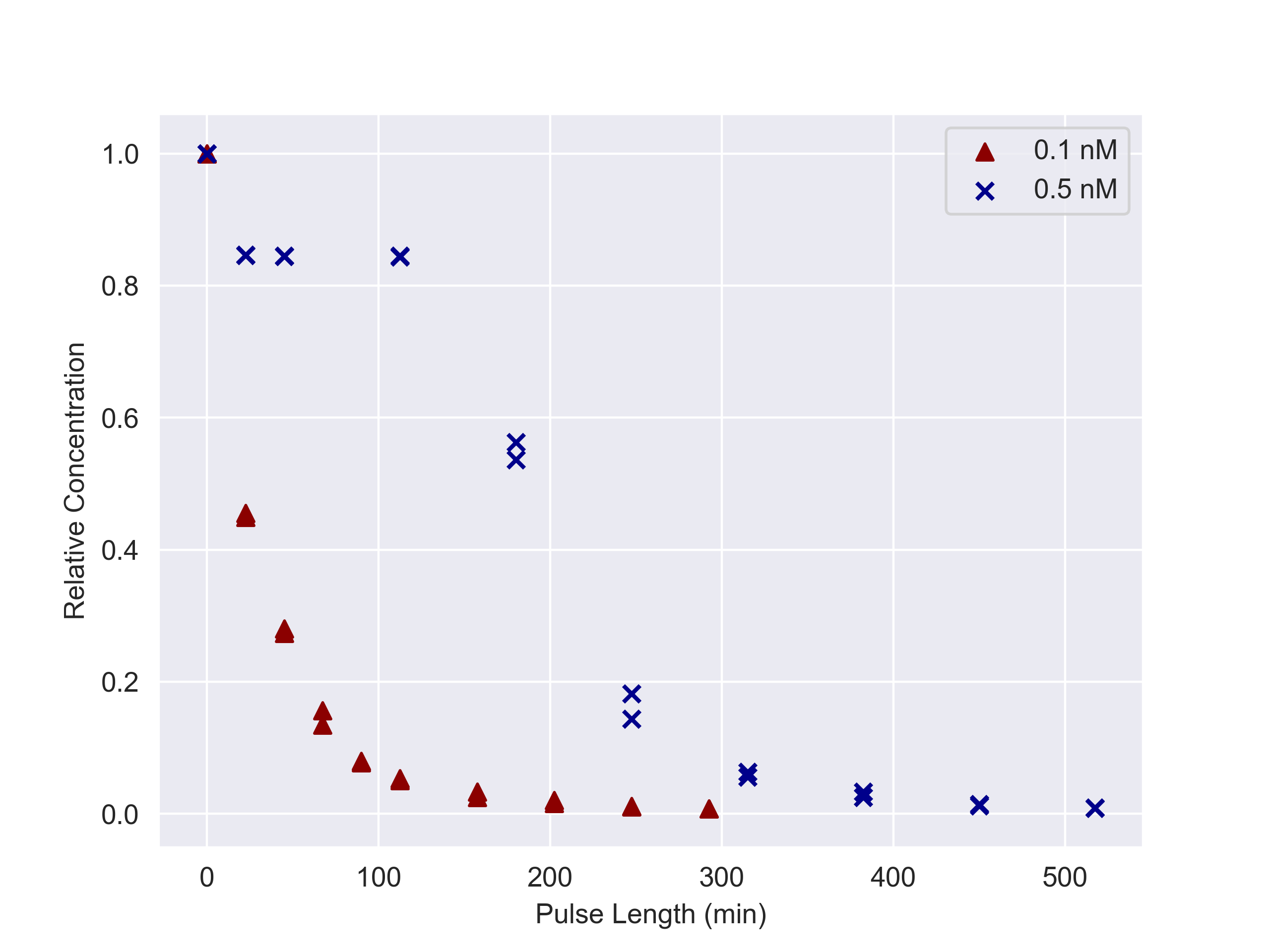

Supplement: Supplementary file 5 — Supplementary Dataset 2 [file 41467_2022_31306_MOESM5_ESM.zip › Individual Simulations Pulse Decoder/110.png]

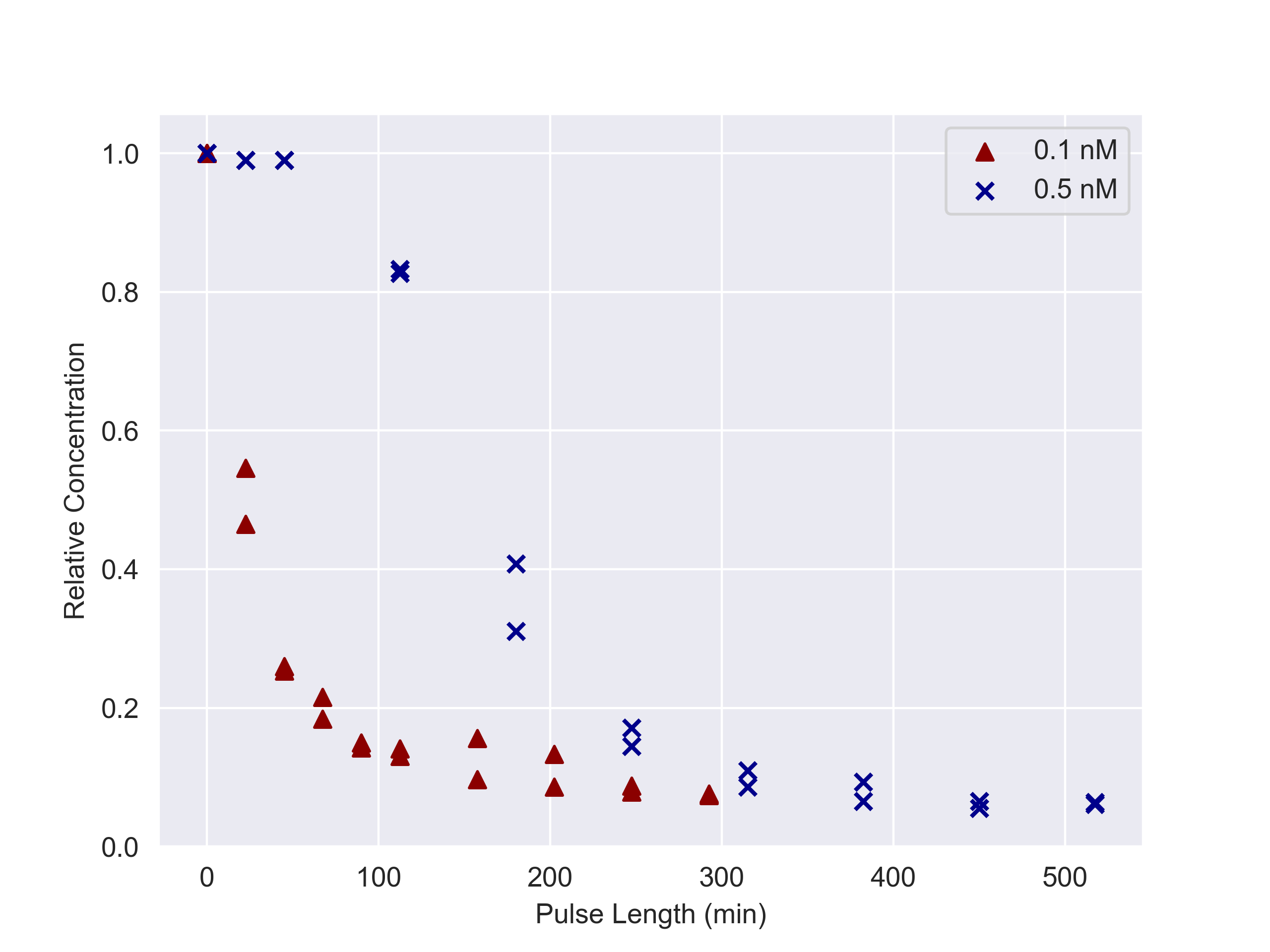

Supplement: Supplementary file 5 — Supplementary Dataset 2 [file 41467_2022_31306_MOESM5_ESM.zip › Individual Simulations Pulse Decoder/111.png]

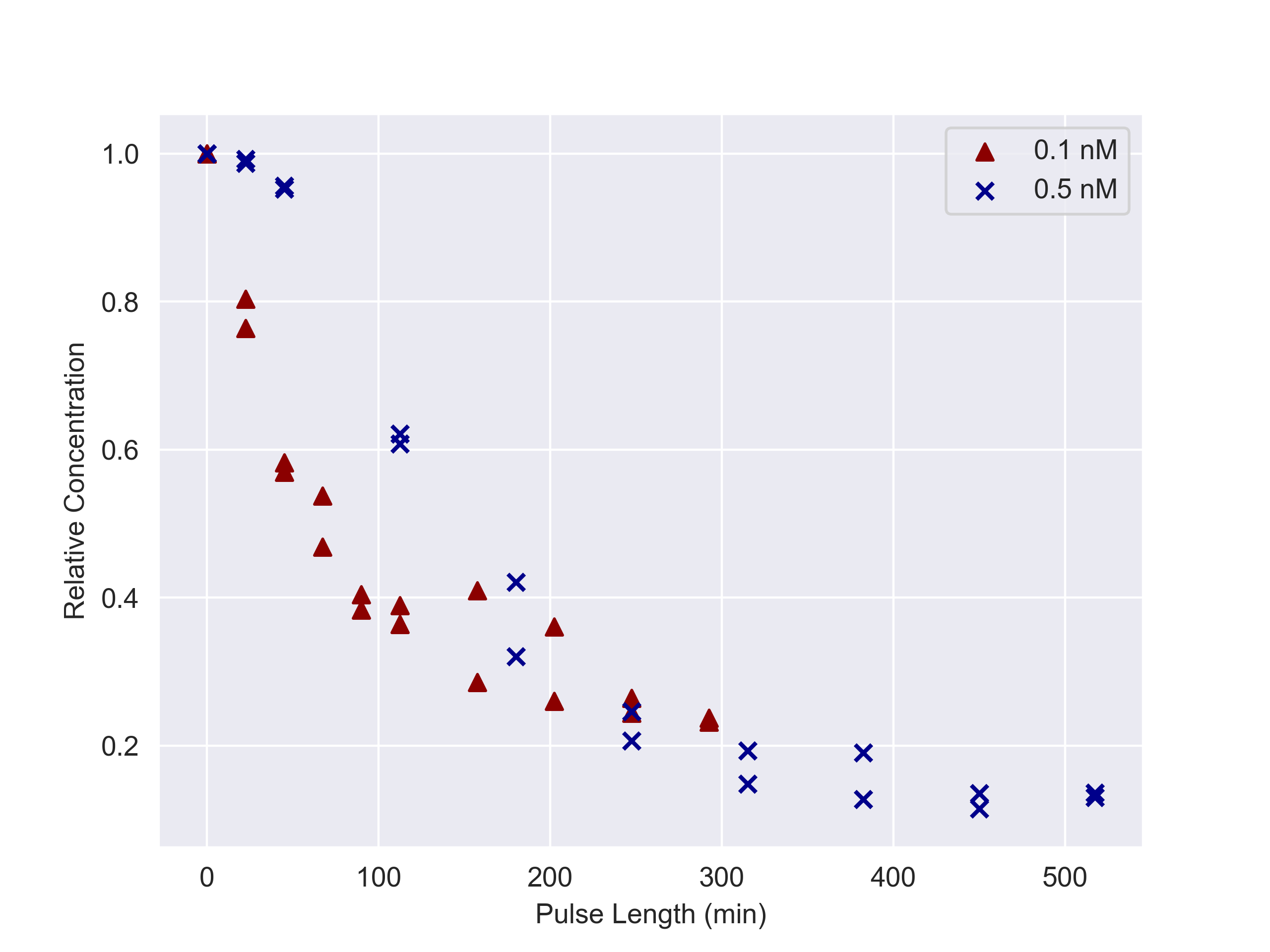

Supplement: Supplementary file 5 — Supplementary Dataset 2 [file 41467_2022_31306_MOESM5_ESM.zip › Individual Simulations Pulse Decoder/112.png]

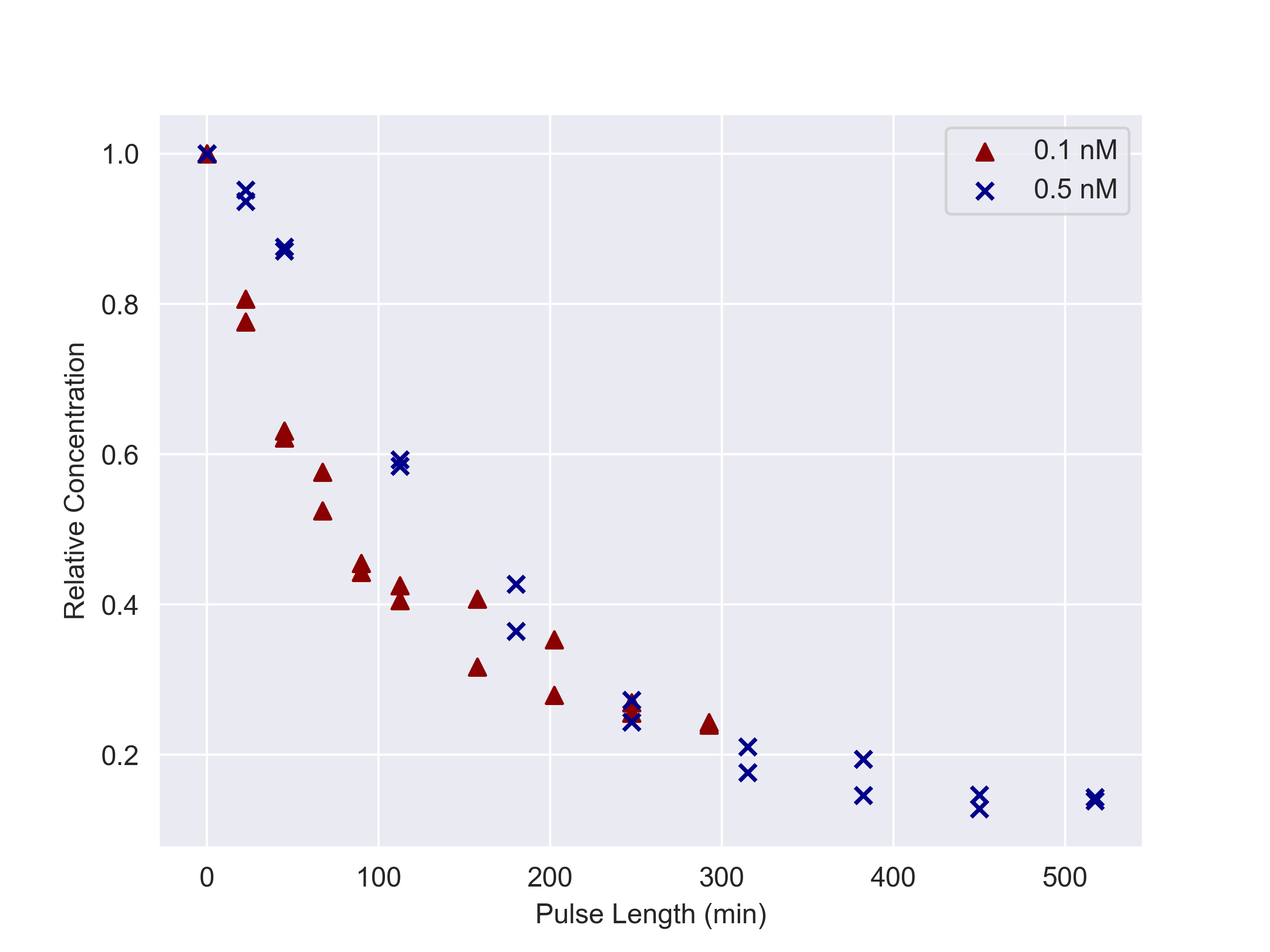

Supplement: Supplementary file 5 — Supplementary Dataset 2 [file 41467_2022_31306_MOESM5_ESM.zip › Individual Simulations Pulse Decoder/113.png]

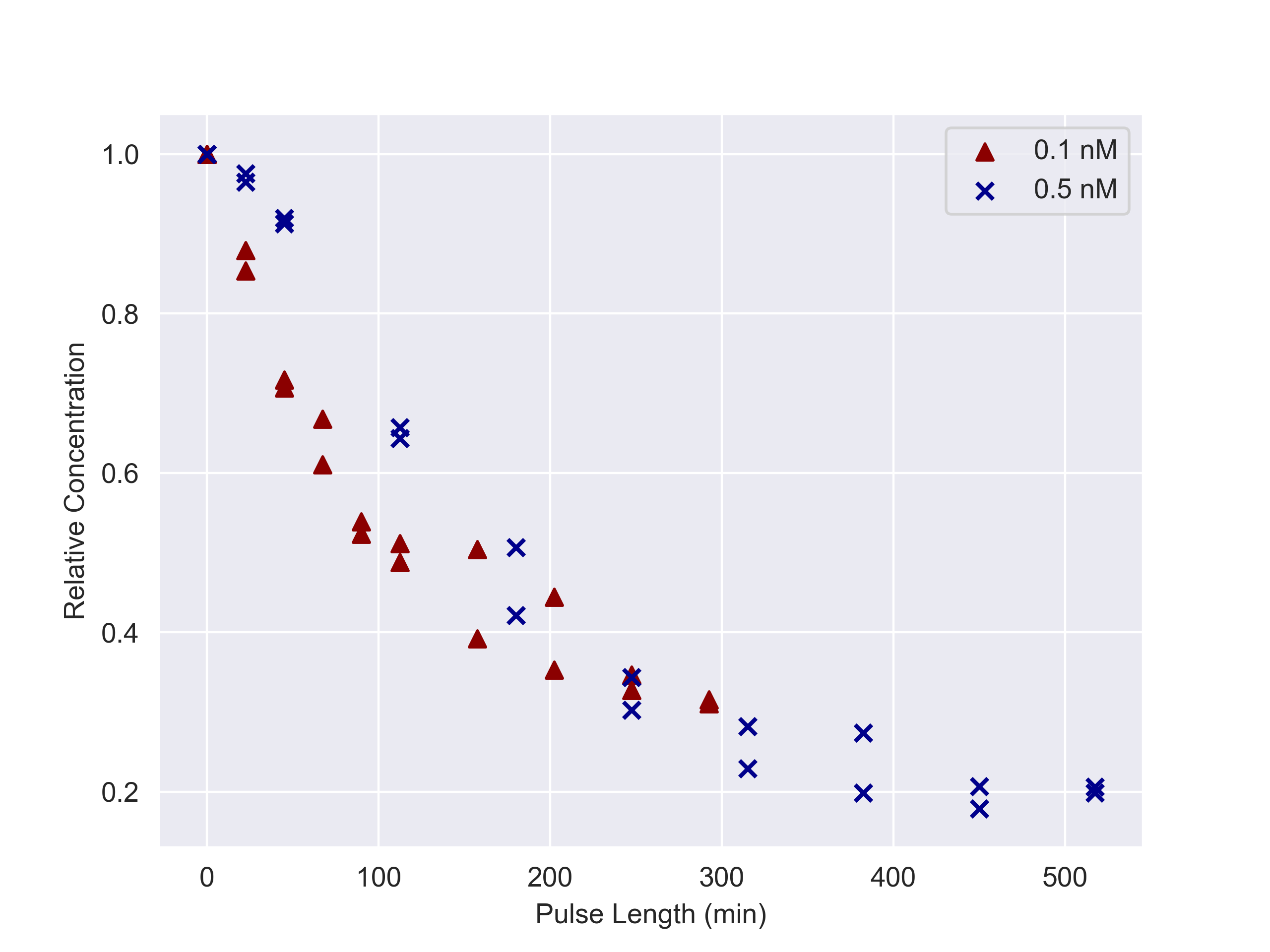

Supplement: Supplementary file 5 — Supplementary Dataset 2 [file 41467_2022_31306_MOESM5_ESM.zip › Individual Simulations Pulse Decoder/114.png]

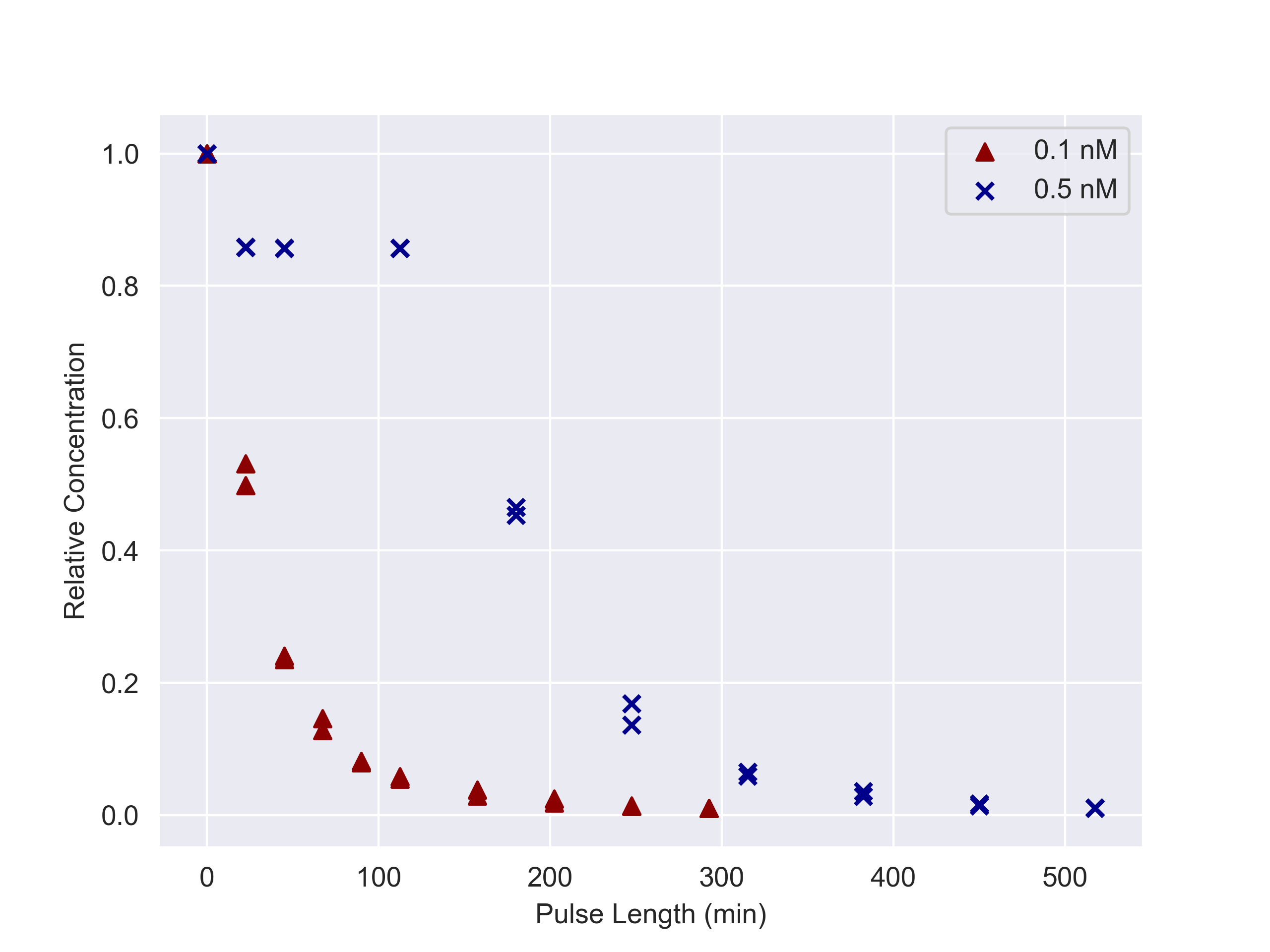

Supplement: Supplementary file 5 — Supplementary Dataset 2 [file 41467_2022_31306_MOESM5_ESM.zip › Individual Simulations Pulse Decoder/115.png]

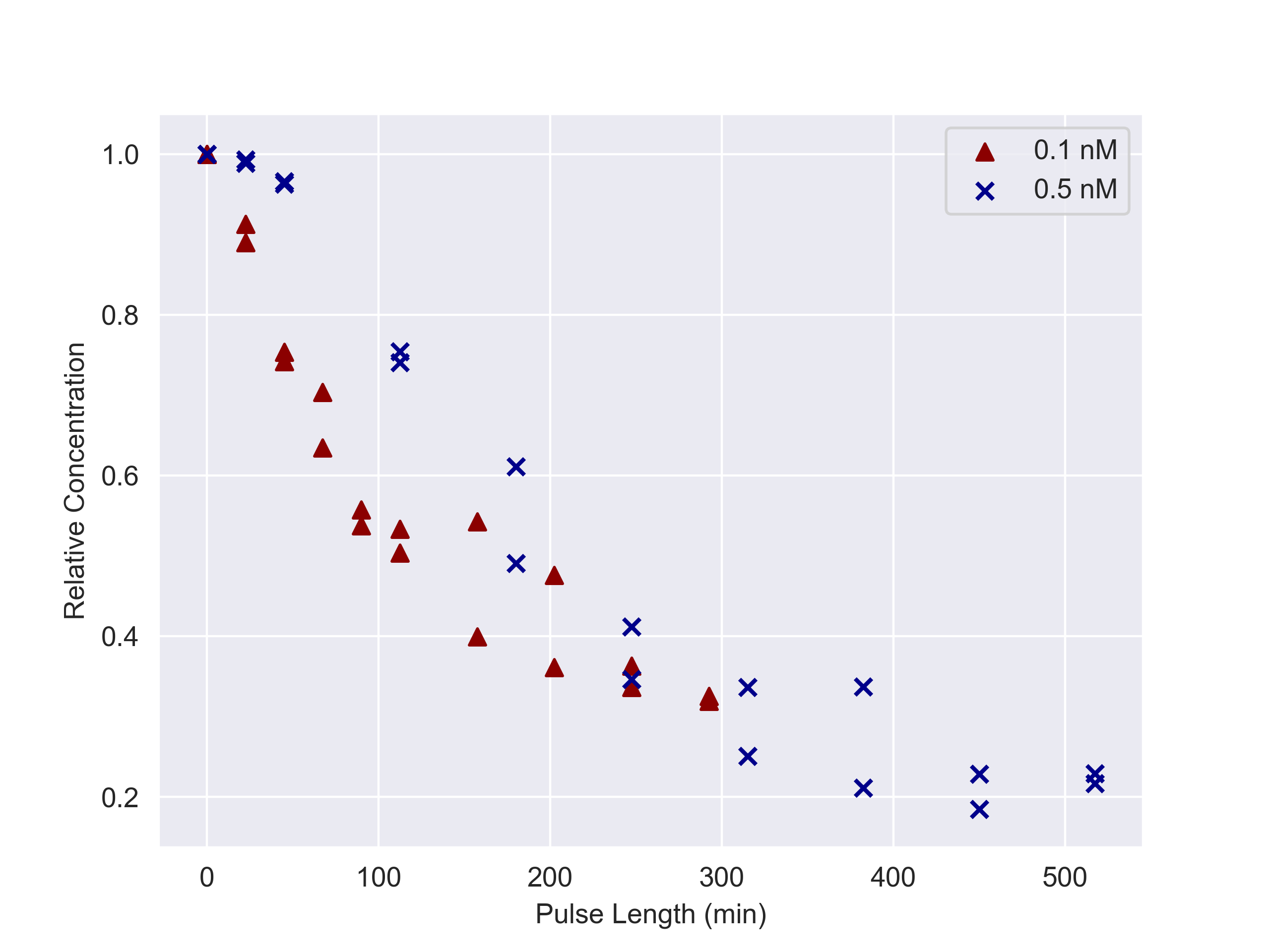

Supplement: Supplementary file 5 — Supplementary Dataset 2 [file 41467_2022_31306_MOESM5_ESM.zip › Individual Simulations Pulse Decoder/116.png]

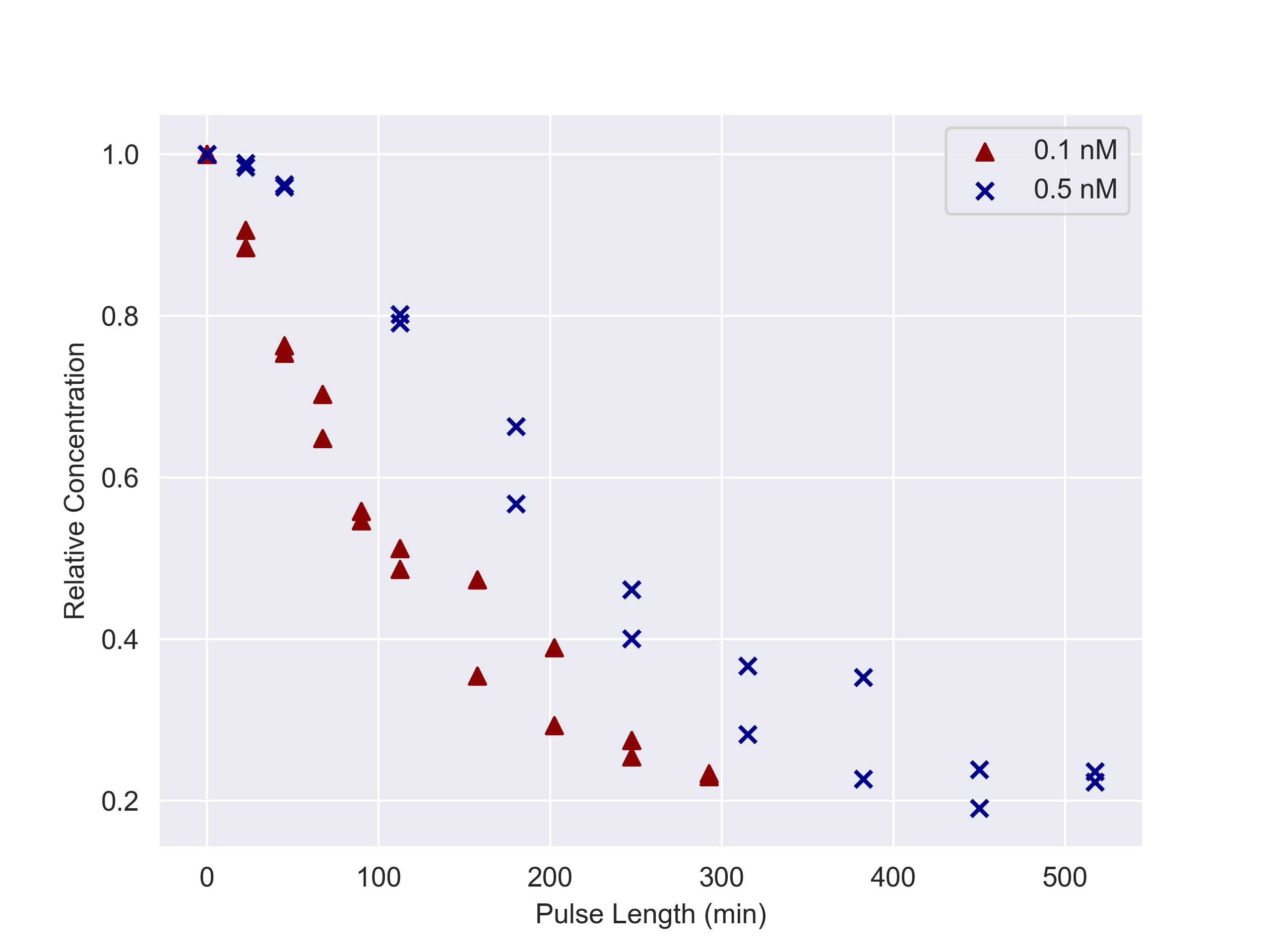

Supplement: Supplementary file 5 — Supplementary Dataset 2 [file 41467_2022_31306_MOESM5_ESM.zip › Individual Simulations Pulse Decoder/117.png]

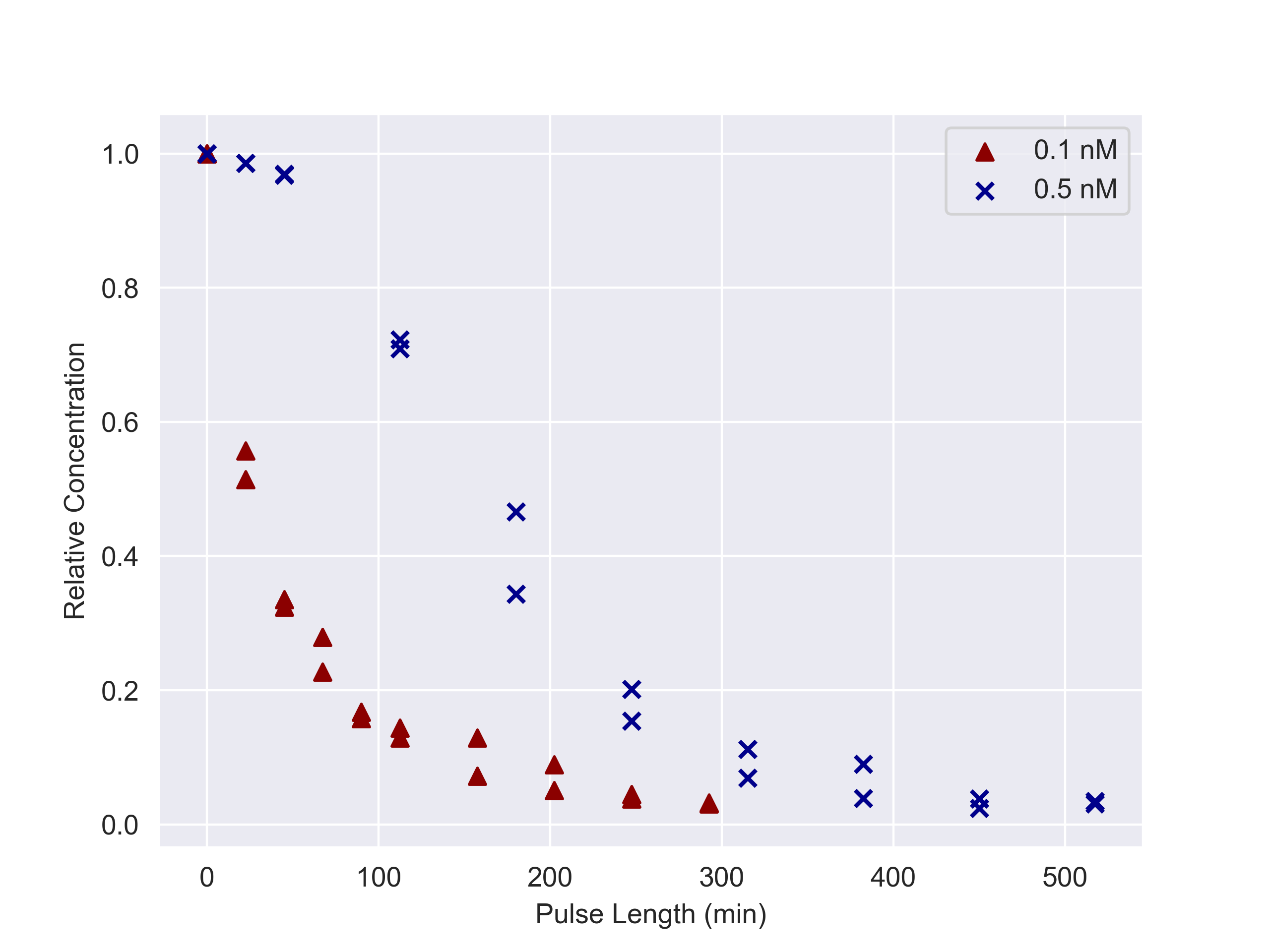

Supplement: Supplementary file 5 — Supplementary Dataset 2 [file 41467_2022_31306_MOESM5_ESM.zip › Individual Simulations Pulse Decoder/118.png]

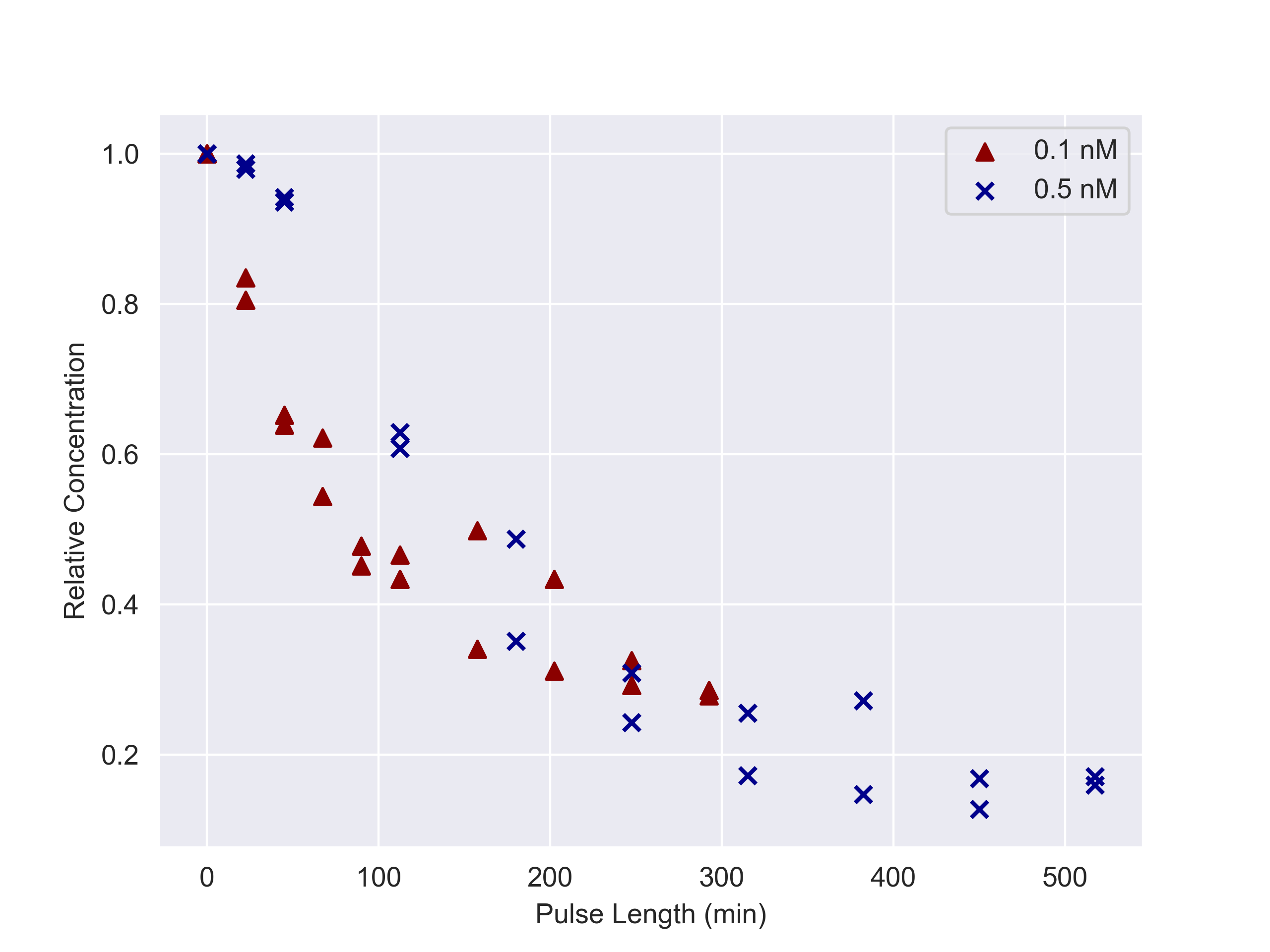

Supplement: Supplementary file 5 — Supplementary Dataset 2 [file 41467_2022_31306_MOESM5_ESM.zip › Individual Simulations Pulse Decoder/119.png]

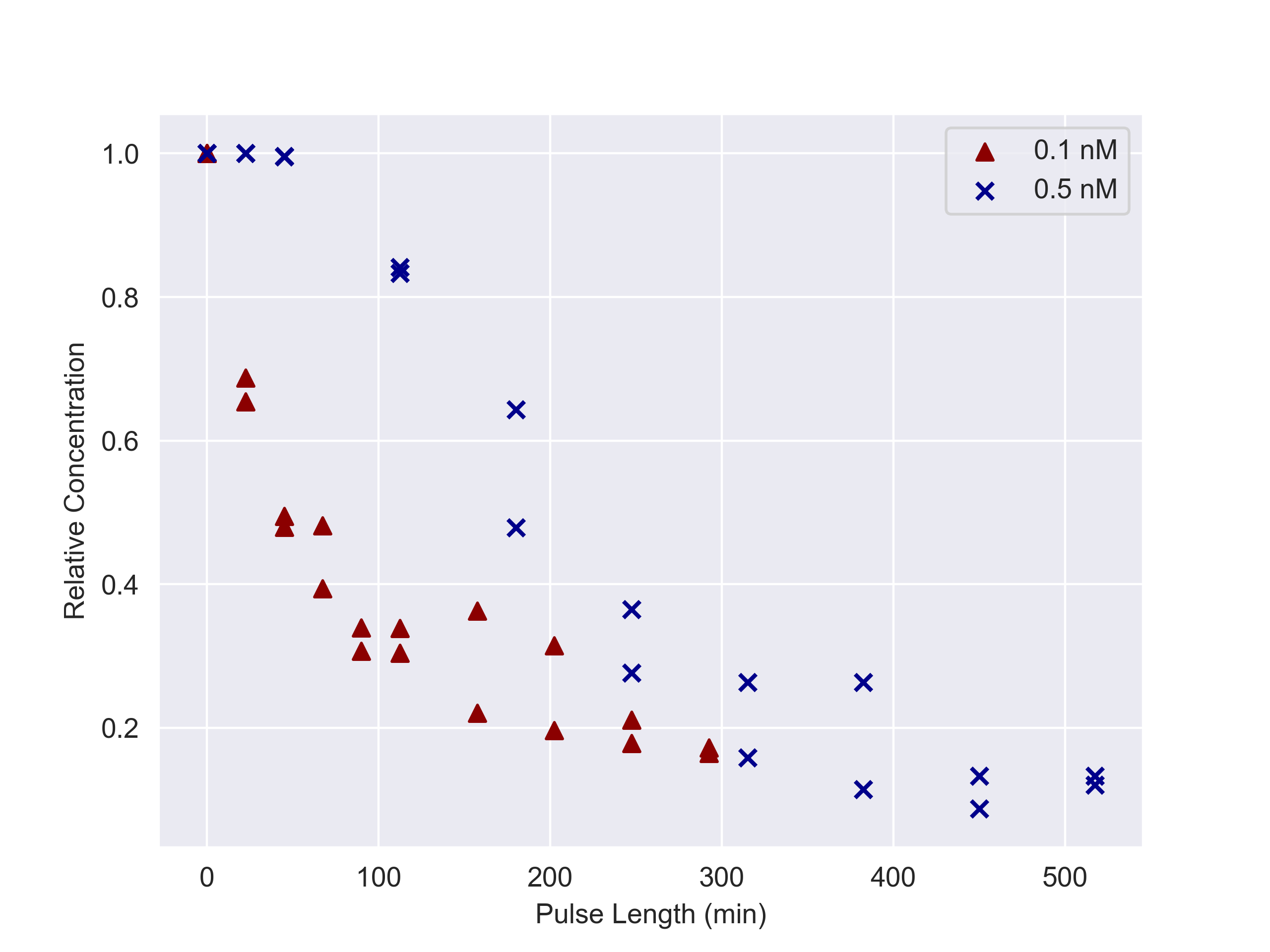

Supplement: Supplementary file 5 — Supplementary Dataset 2 [file 41467_2022_31306_MOESM5_ESM.zip › Individual Simulations Pulse Decoder/12.png]

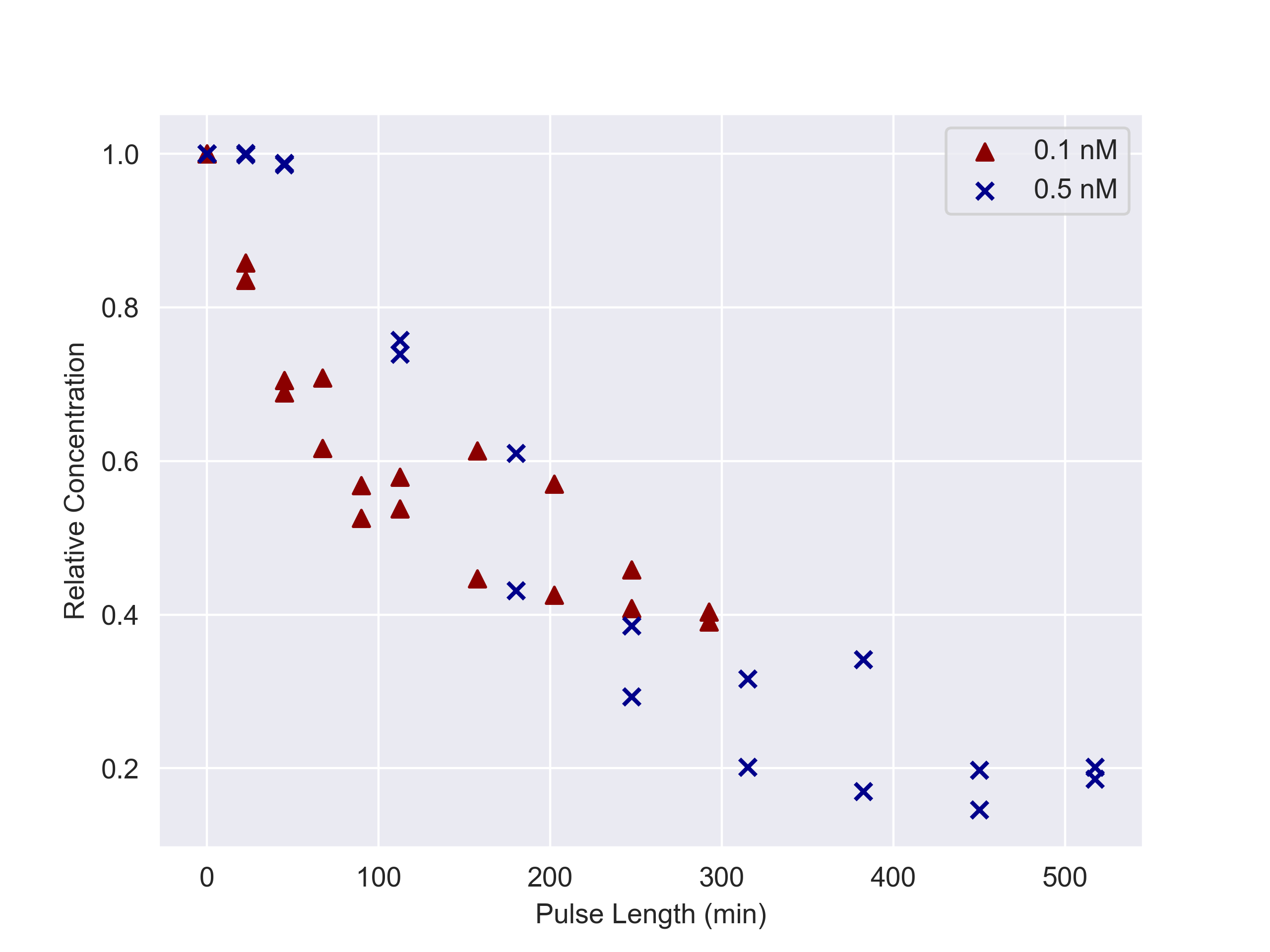

Supplement: Supplementary file 5 — Supplementary Dataset 2 [file 41467_2022_31306_MOESM5_ESM.zip › Individual Simulations Pulse Decoder/120.png]

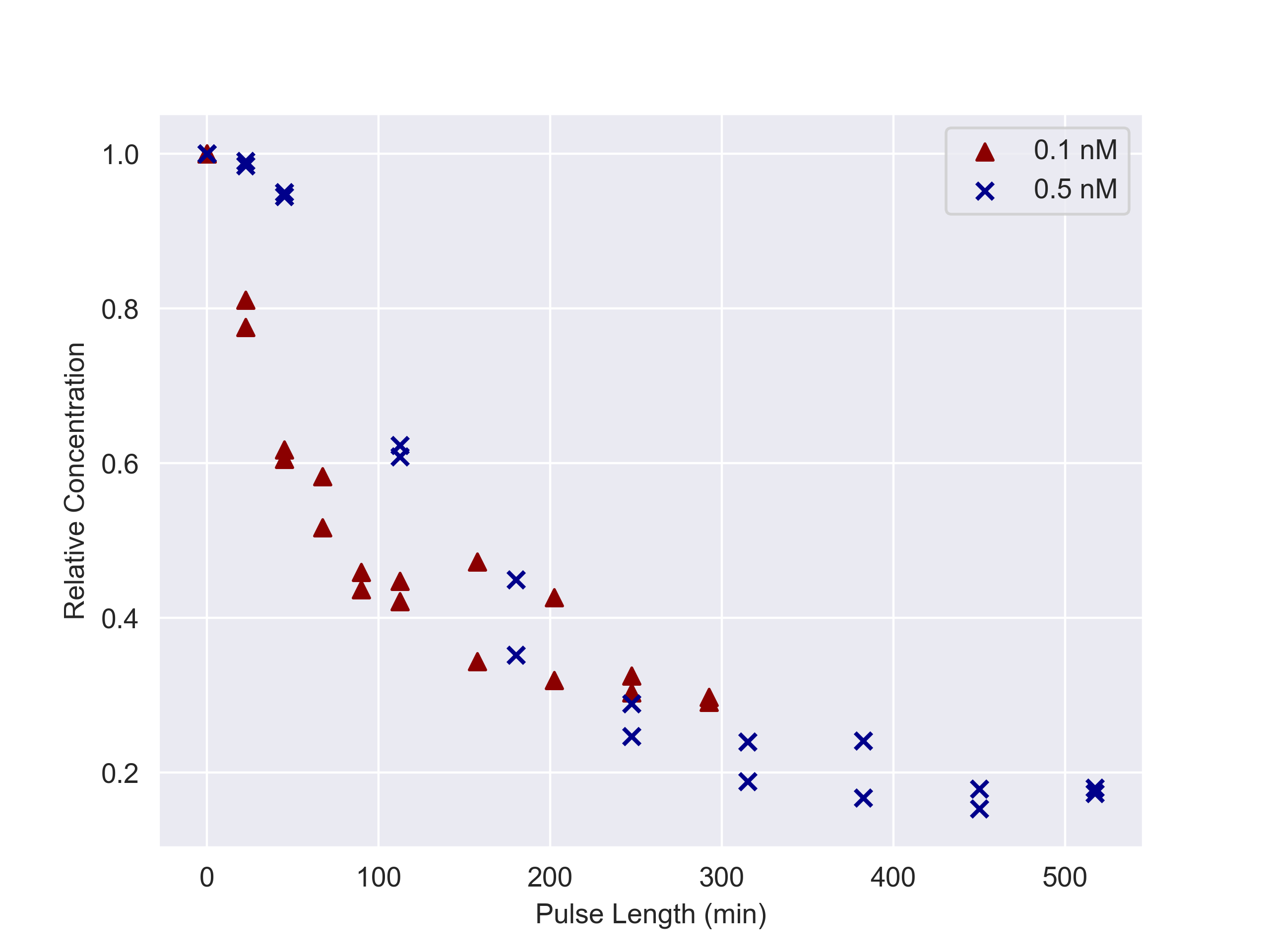

Supplement: Supplementary file 5 — Supplementary Dataset 2 [file 41467_2022_31306_MOESM5_ESM.zip › Individual Simulations Pulse Decoder/121.png]

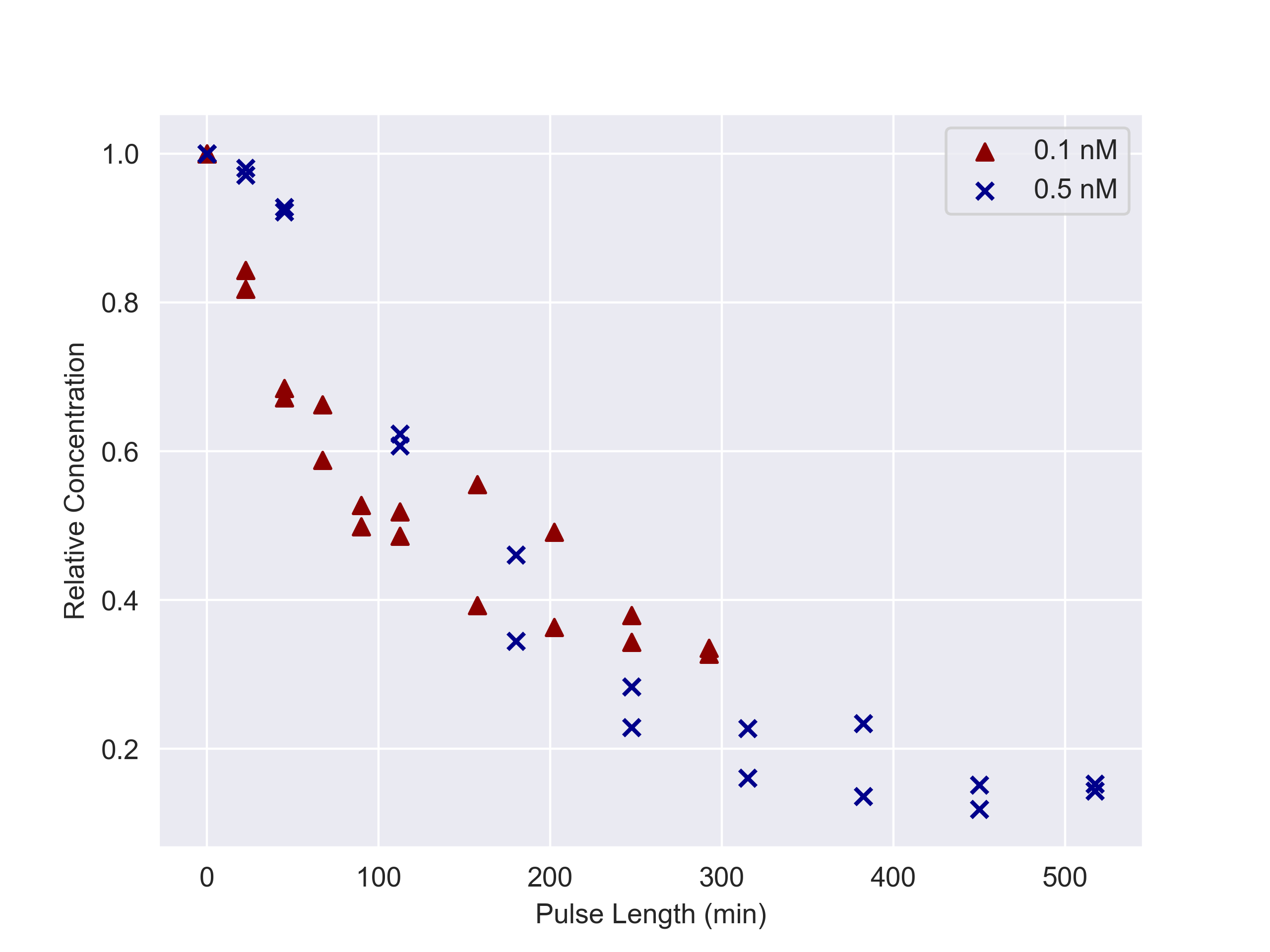

Supplement: Supplementary file 5 — Supplementary Dataset 2 [file 41467_2022_31306_MOESM5_ESM.zip › Individual Simulations Pulse Decoder/122.png]

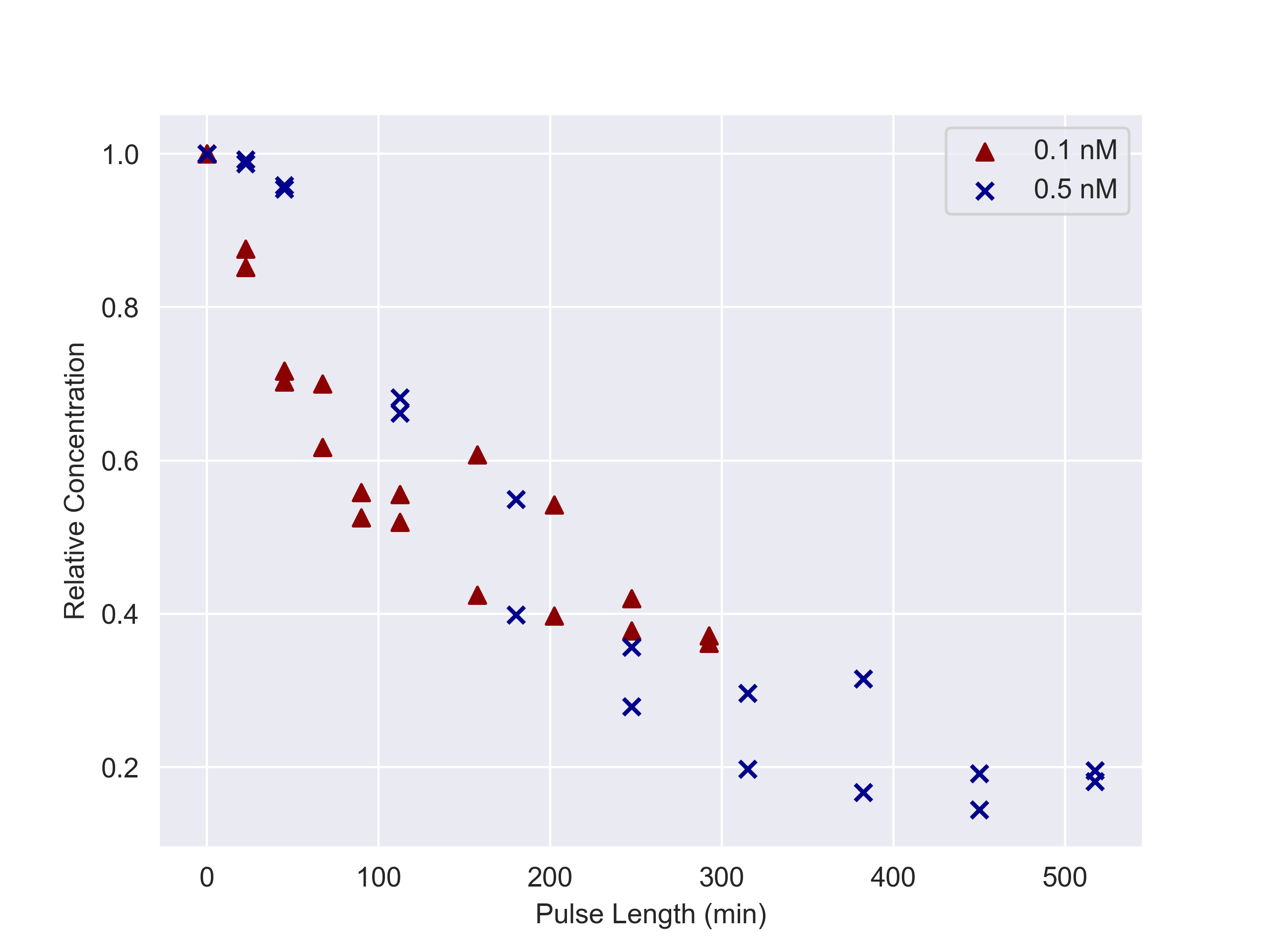

Supplement: Supplementary file 5 — Supplementary Dataset 2 [file 41467_2022_31306_MOESM5_ESM.zip › Individual Simulations Pulse Decoder/123.png]

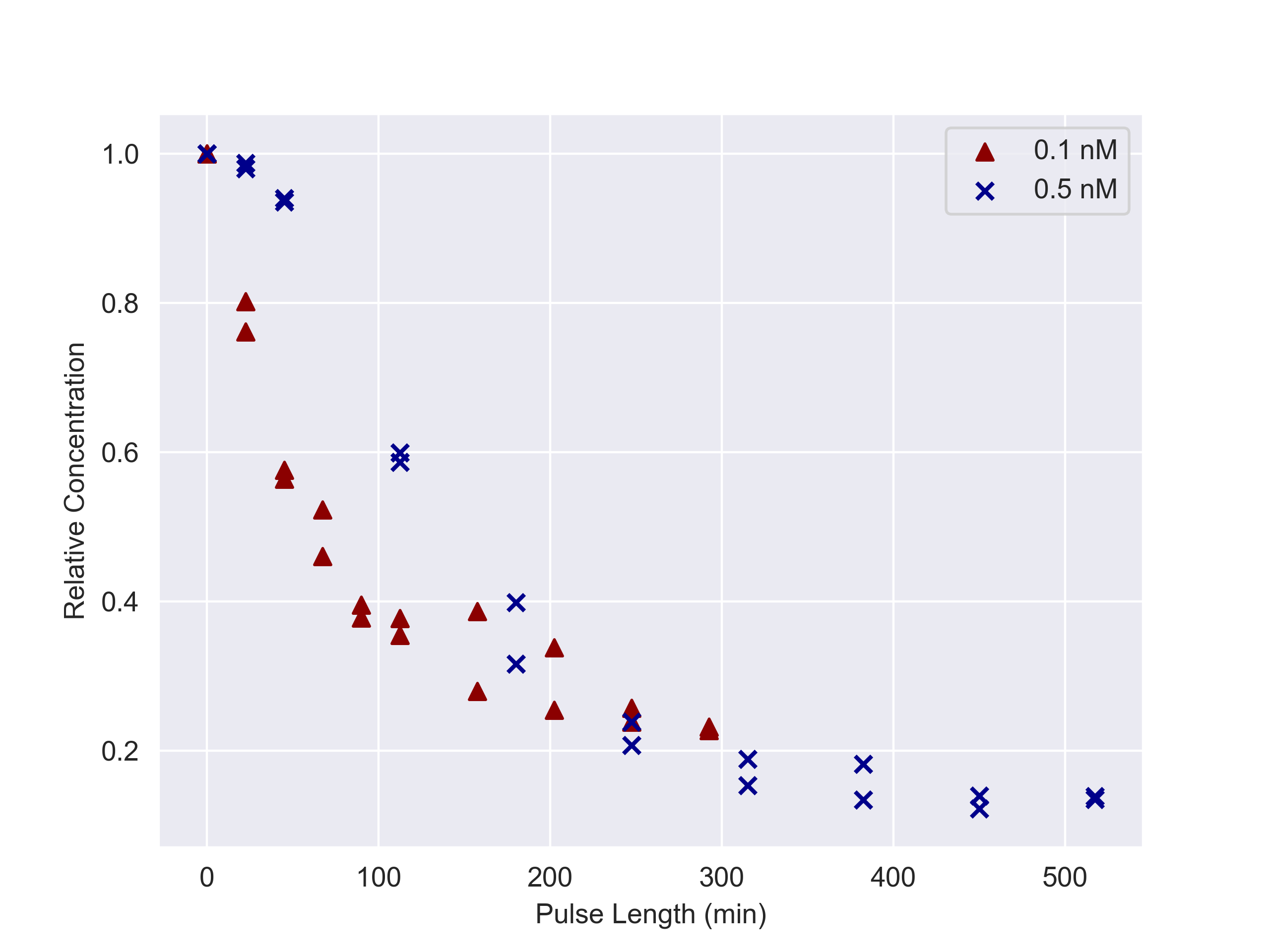

Supplement: Supplementary file 5 — Supplementary Dataset 2 [file 41467_2022_31306_MOESM5_ESM.zip › Individual Simulations Pulse Decoder/124.png]

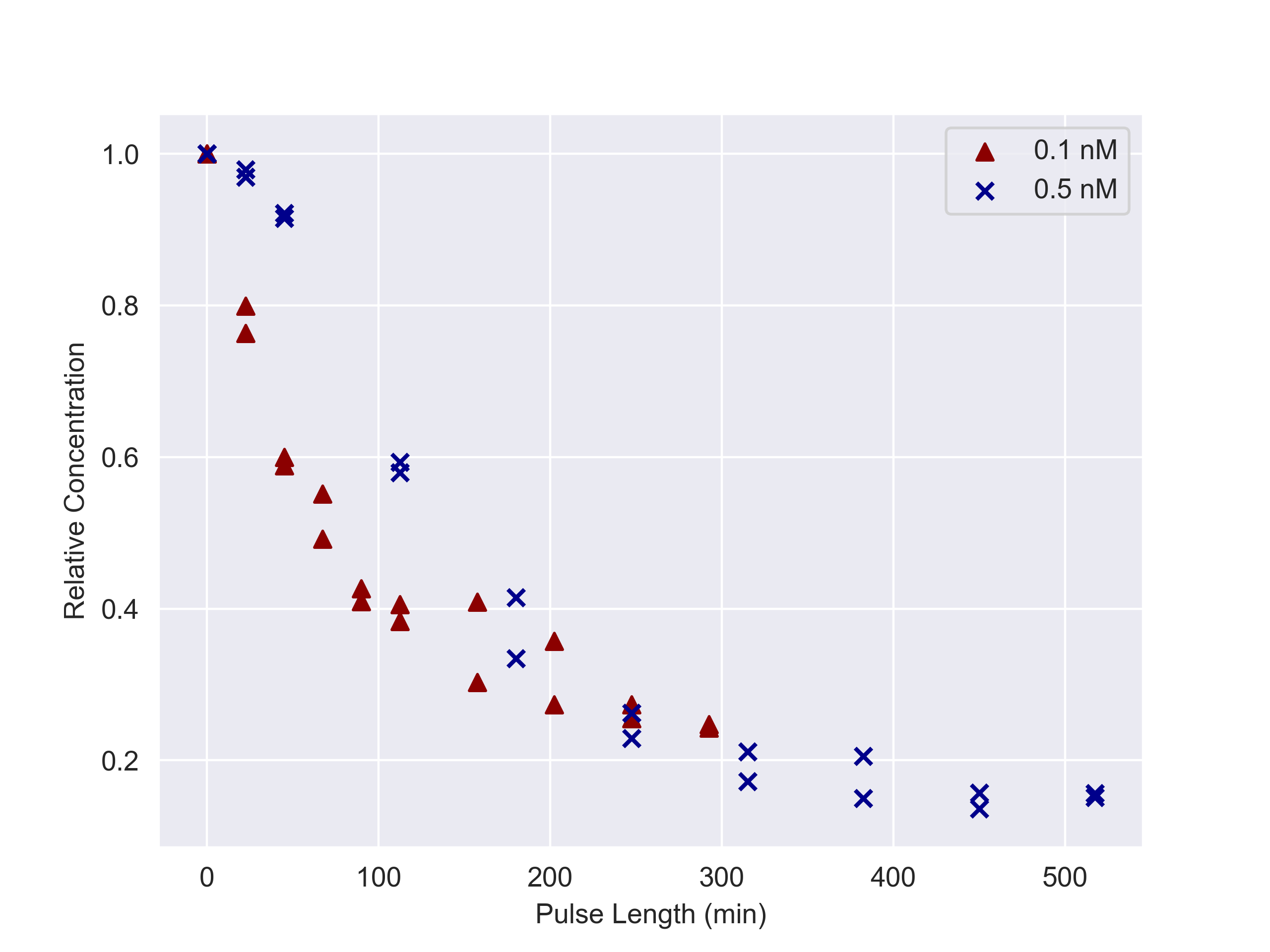

Supplement: Supplementary file 5 — Supplementary Dataset 2 [file 41467_2022_31306_MOESM5_ESM.zip › Individual Simulations Pulse Decoder/125.png]

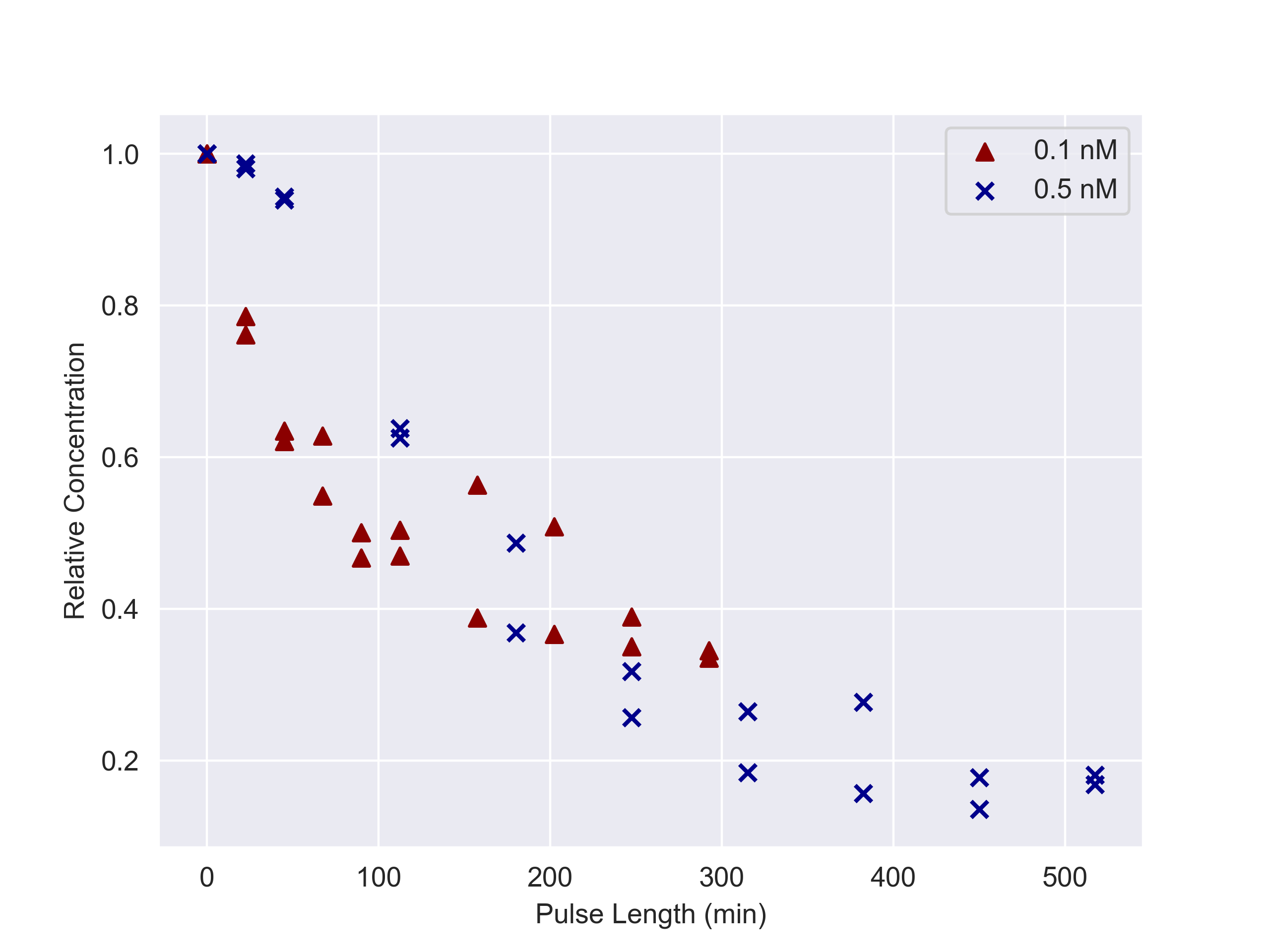

Supplement: Supplementary file 5 — Supplementary Dataset 2 [file 41467_2022_31306_MOESM5_ESM.zip › Individual Simulations Pulse Decoder/126.png]

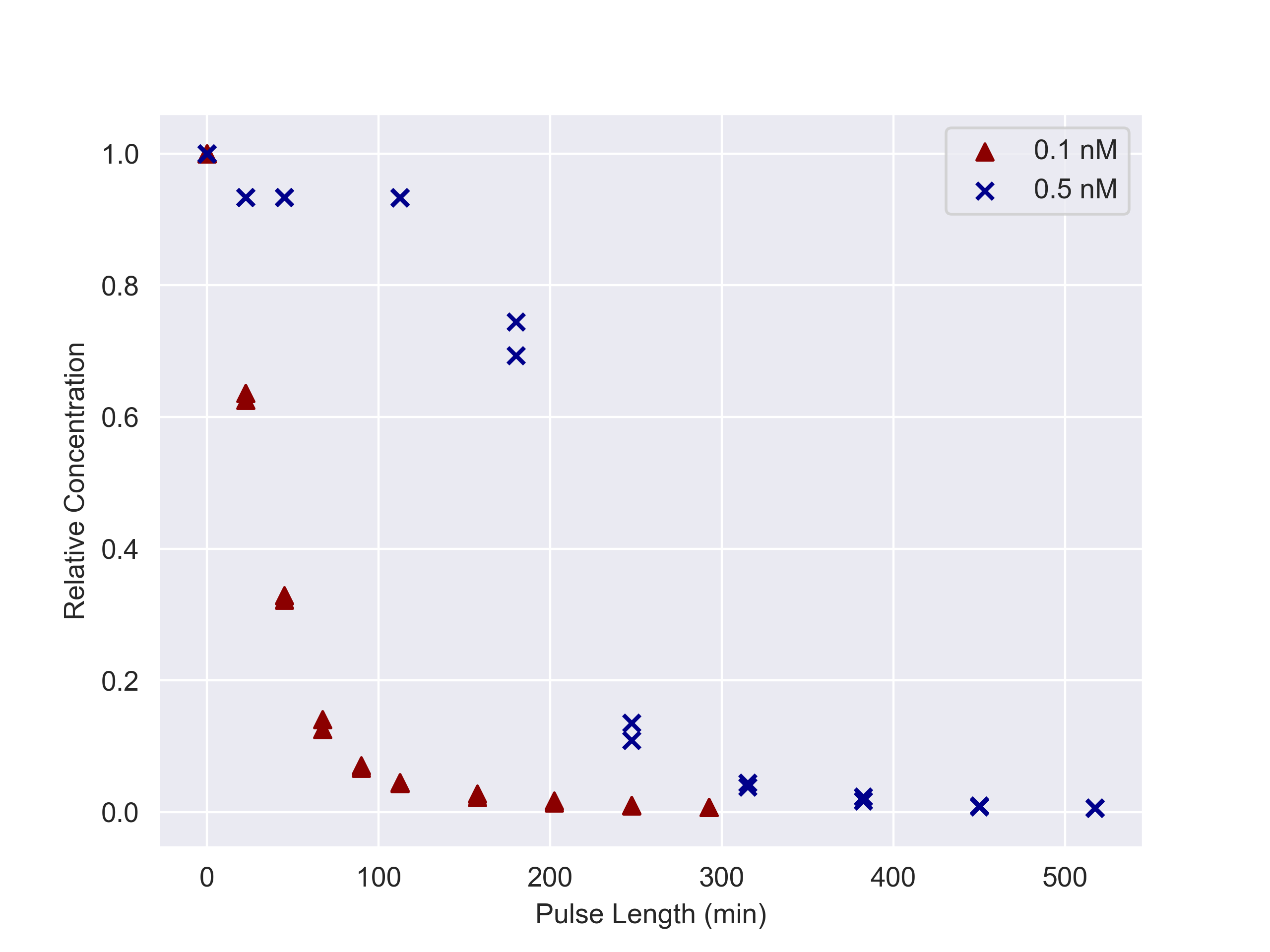

Supplement: Supplementary file 5 — Supplementary Dataset 2 [file 41467_2022_31306_MOESM5_ESM.zip › Individual Simulations Pulse Decoder/127.png]

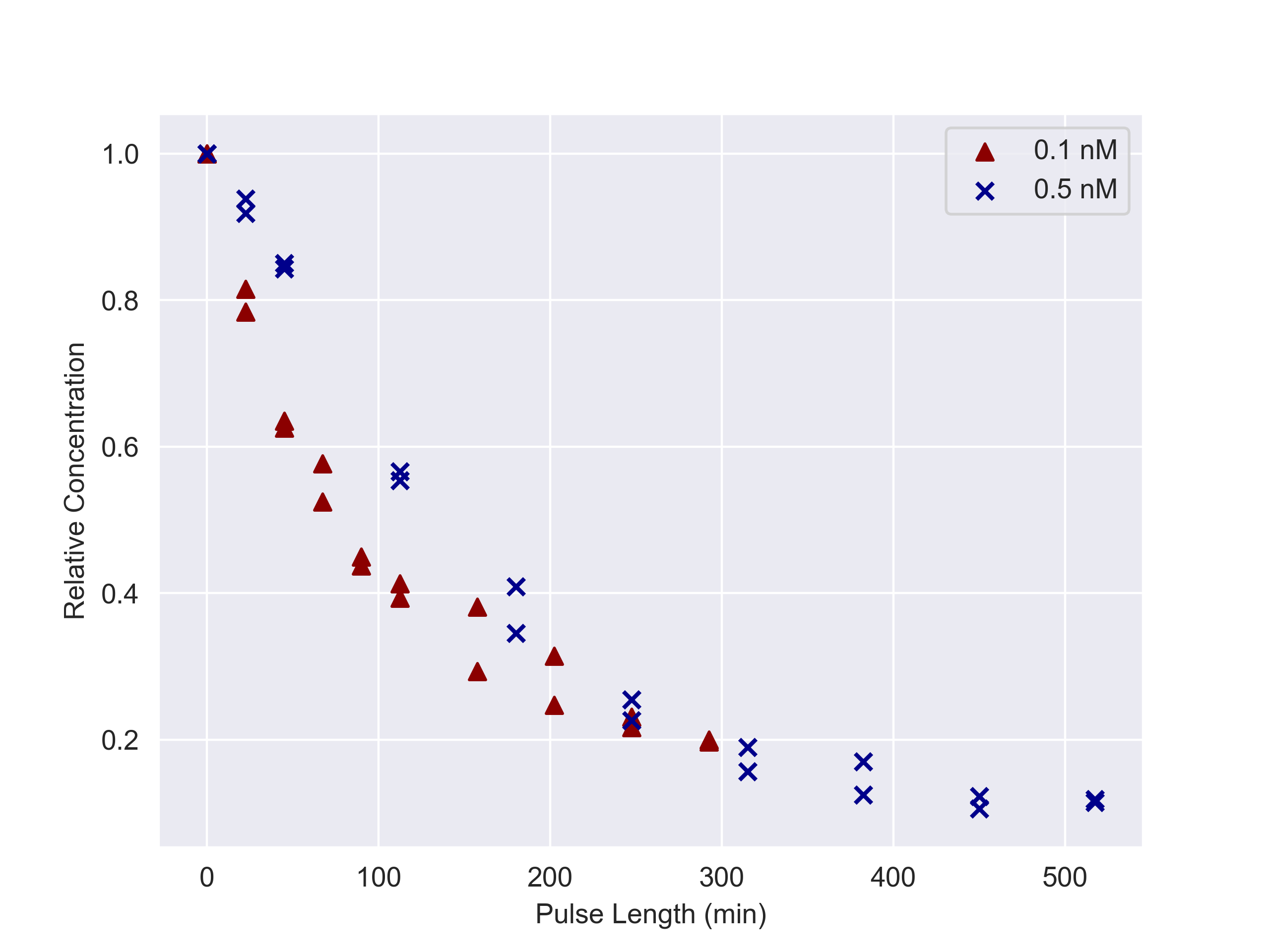

Supplement: Supplementary file 5 — Supplementary Dataset 2 [file 41467_2022_31306_MOESM5_ESM.zip › Individual Simulations Pulse Decoder/128.png]

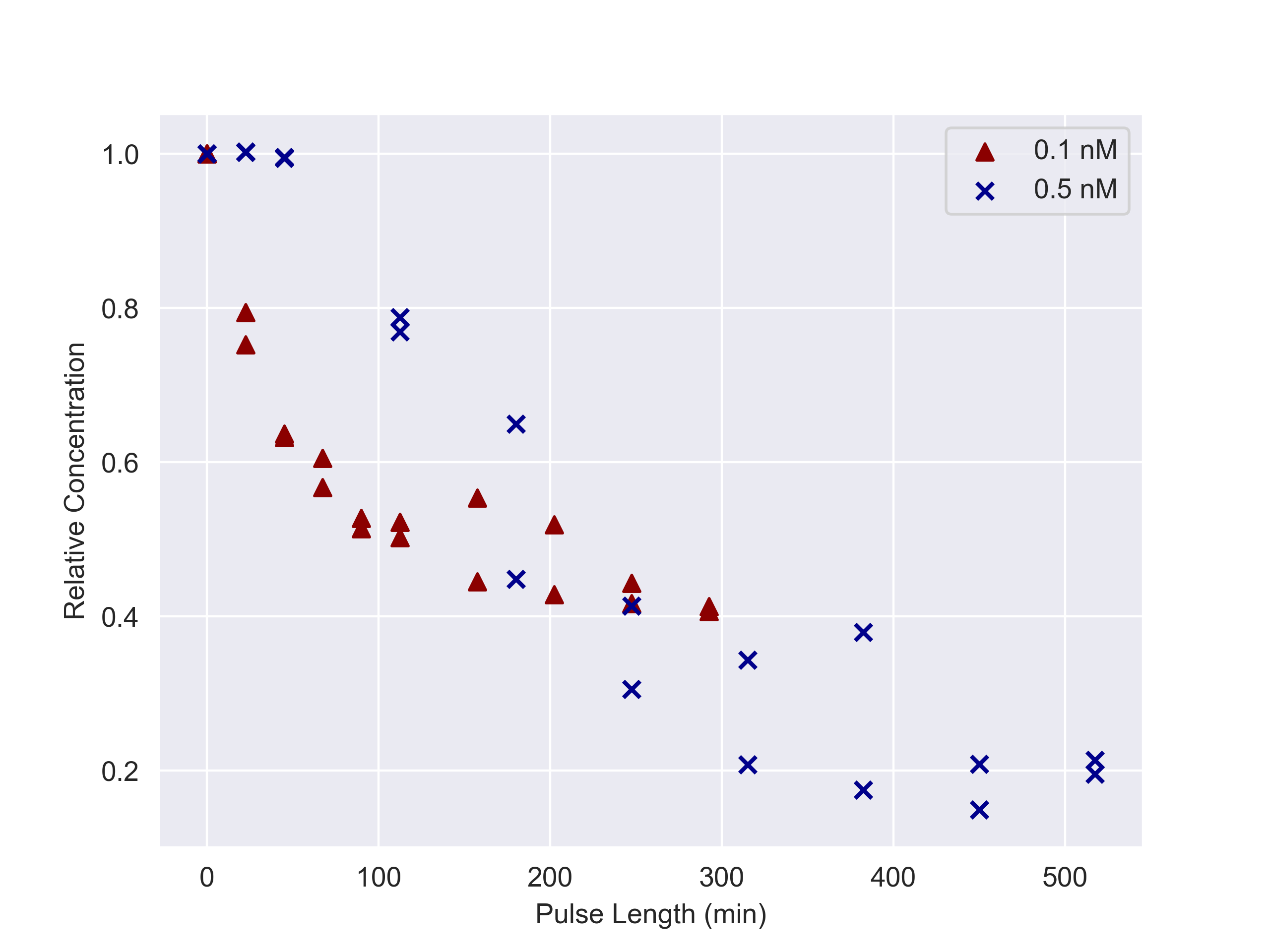

Supplement: Supplementary file 5 — Supplementary Dataset 2 [file 41467_2022_31306_MOESM5_ESM.zip › Individual Simulations Pulse Decoder/129.png]

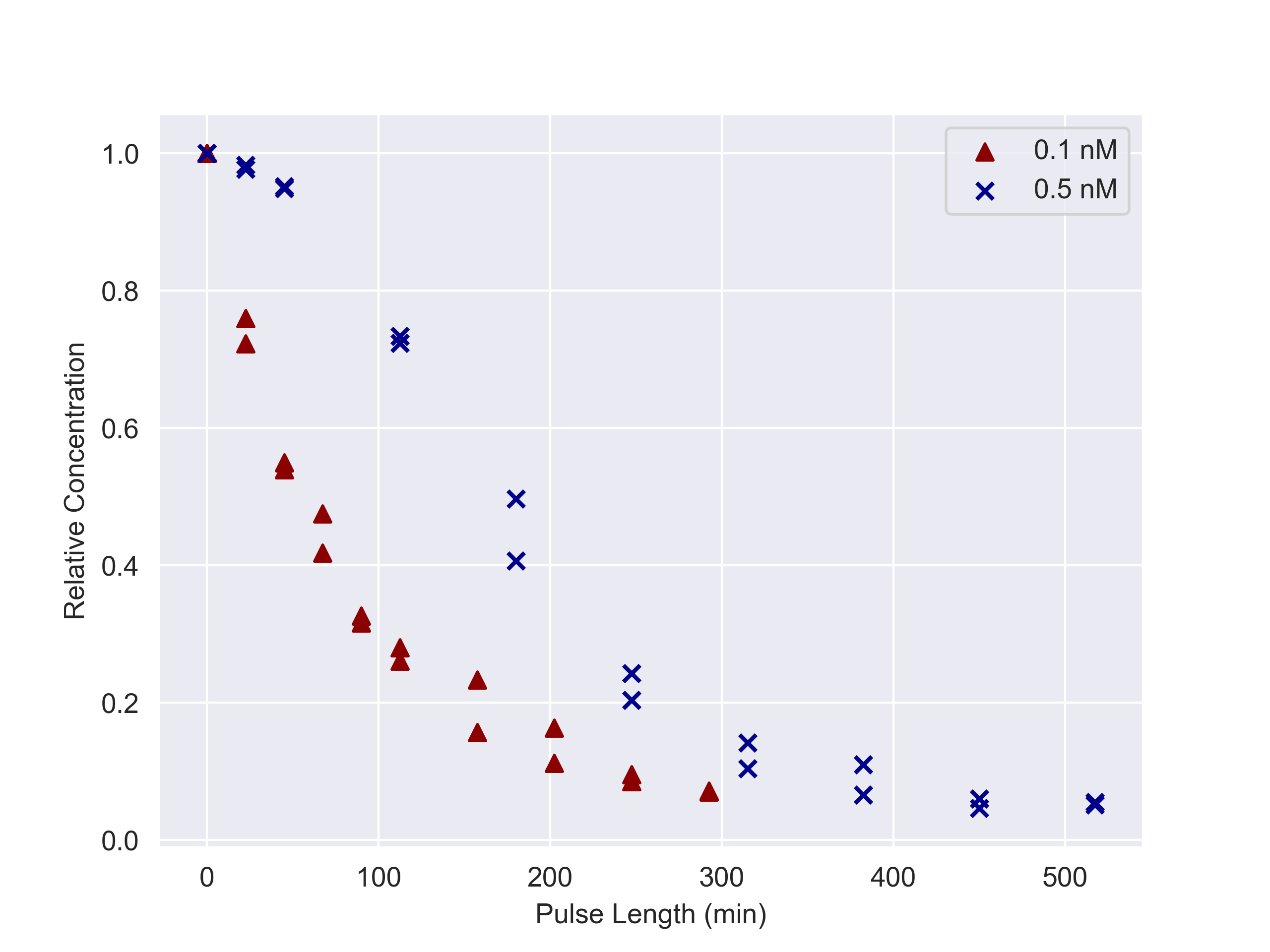

Supplement: Supplementary file 5 — Supplementary Dataset 2 [file 41467_2022_31306_MOESM5_ESM.zip › Individual Simulations Pulse Decoder/13.png]

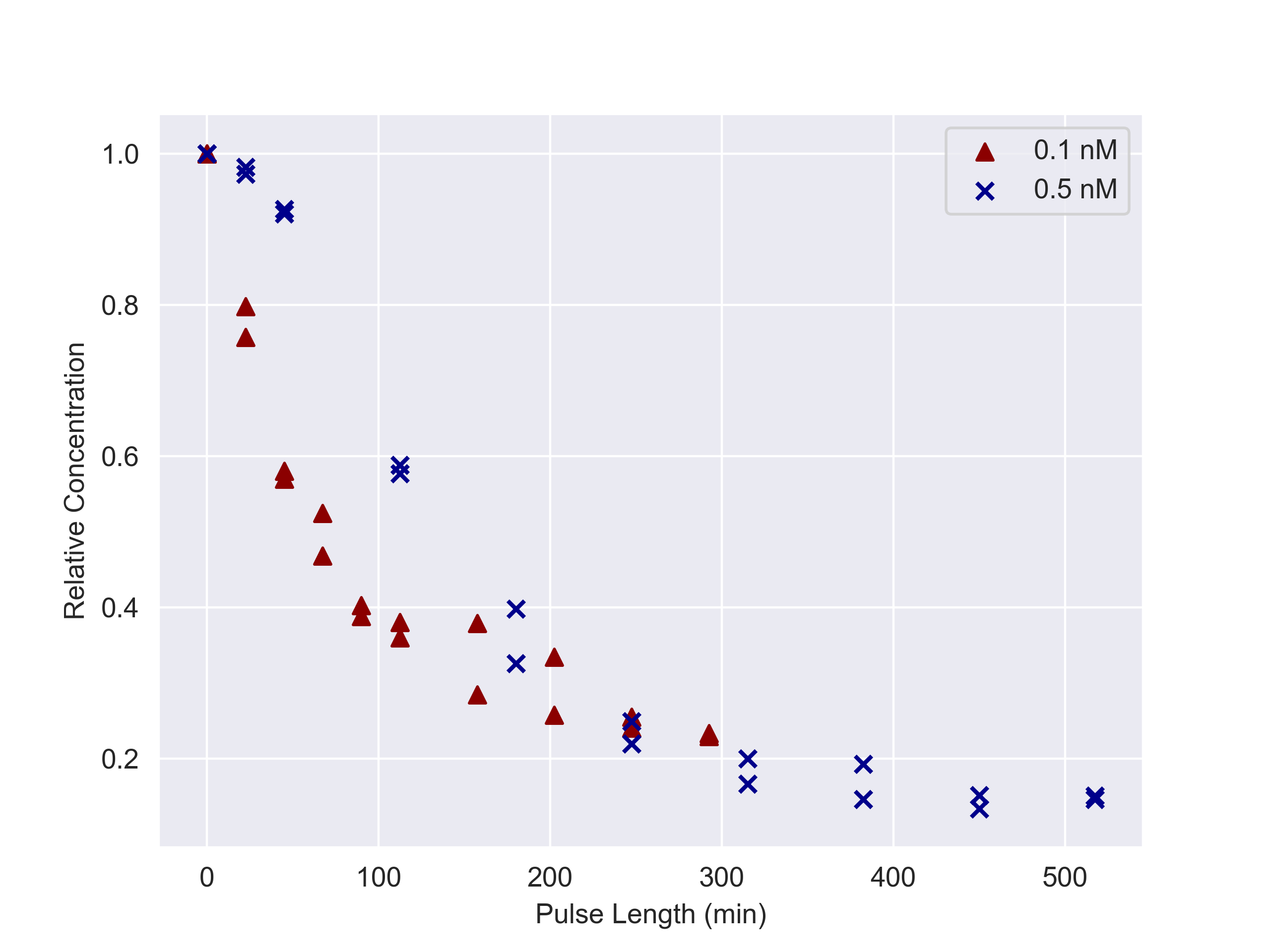

Supplement: Supplementary file 5 — Supplementary Dataset 2 [file 41467_2022_31306_MOESM5_ESM.zip › Individual Simulations Pulse Decoder/130.png]

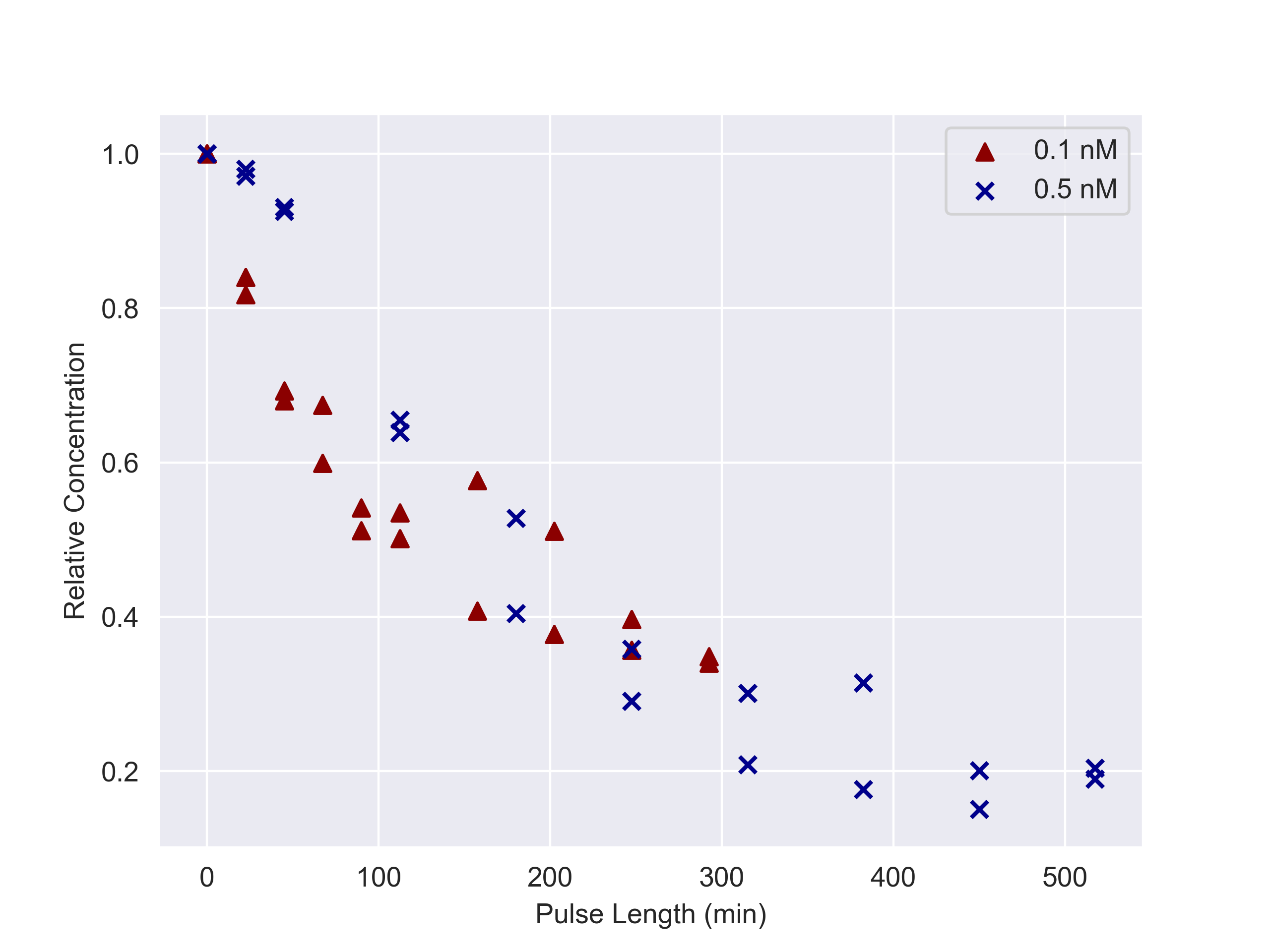

Supplement: Supplementary file 5 — Supplementary Dataset 2 [file 41467_2022_31306_MOESM5_ESM.zip › Individual Simulations Pulse Decoder/131.png]

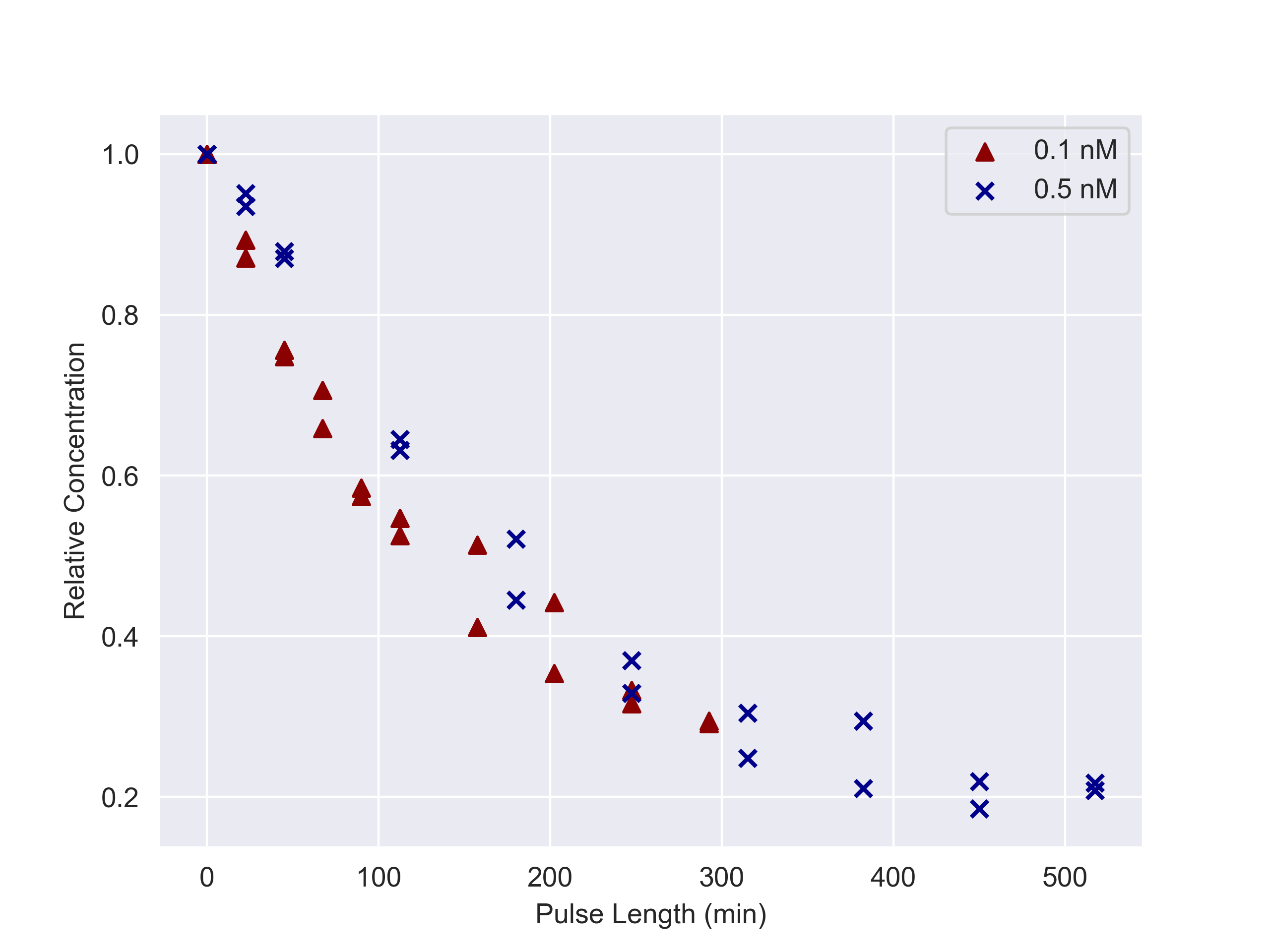

Supplement: Supplementary file 5 — Supplementary Dataset 2 [file 41467_2022_31306_MOESM5_ESM.zip › Individual Simulations Pulse Decoder/132.png]

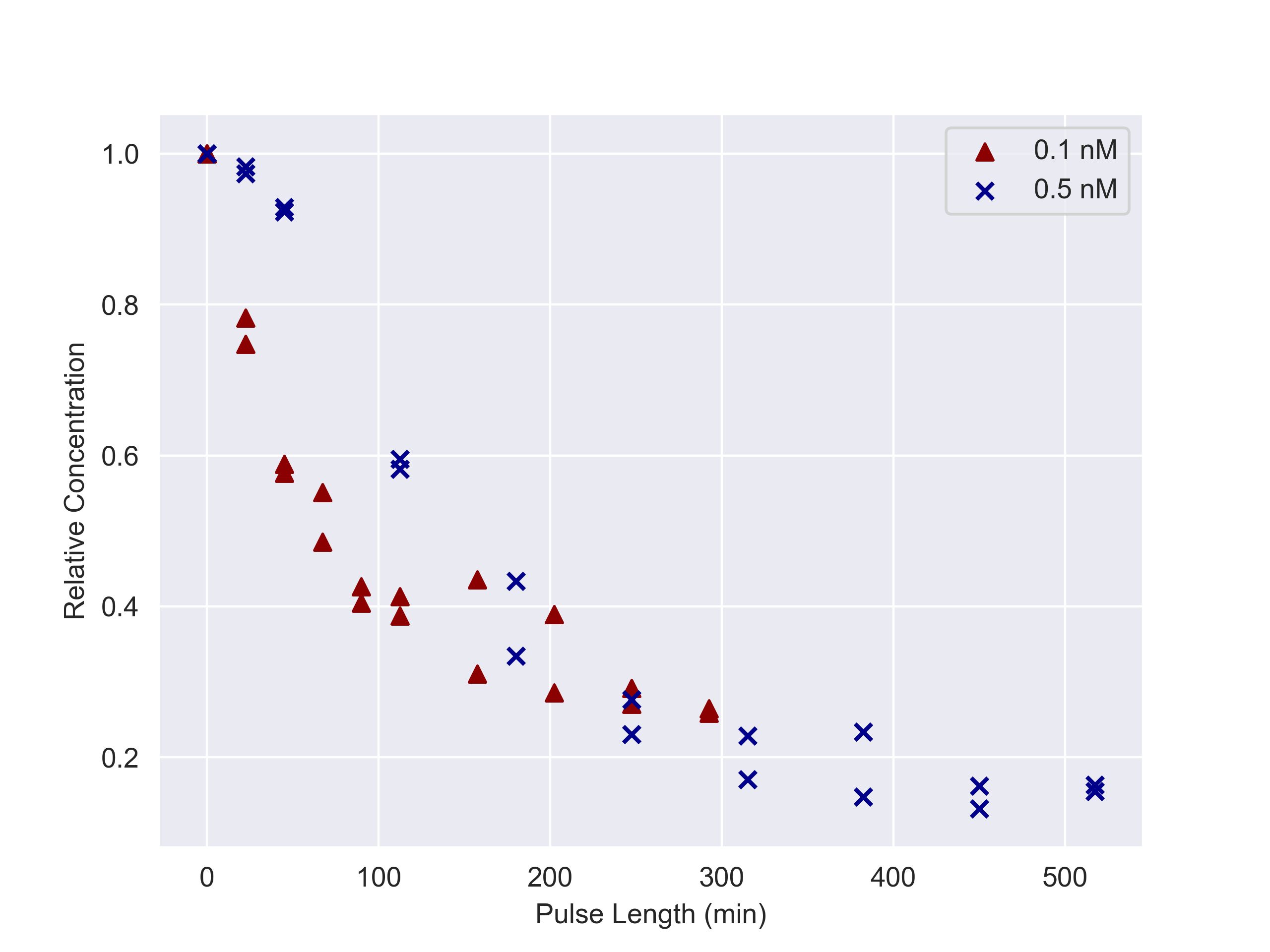

Supplement: Supplementary file 5 — Supplementary Dataset 2 [file 41467_2022_31306_MOESM5_ESM.zip › Individual Simulations Pulse Decoder/133.png]

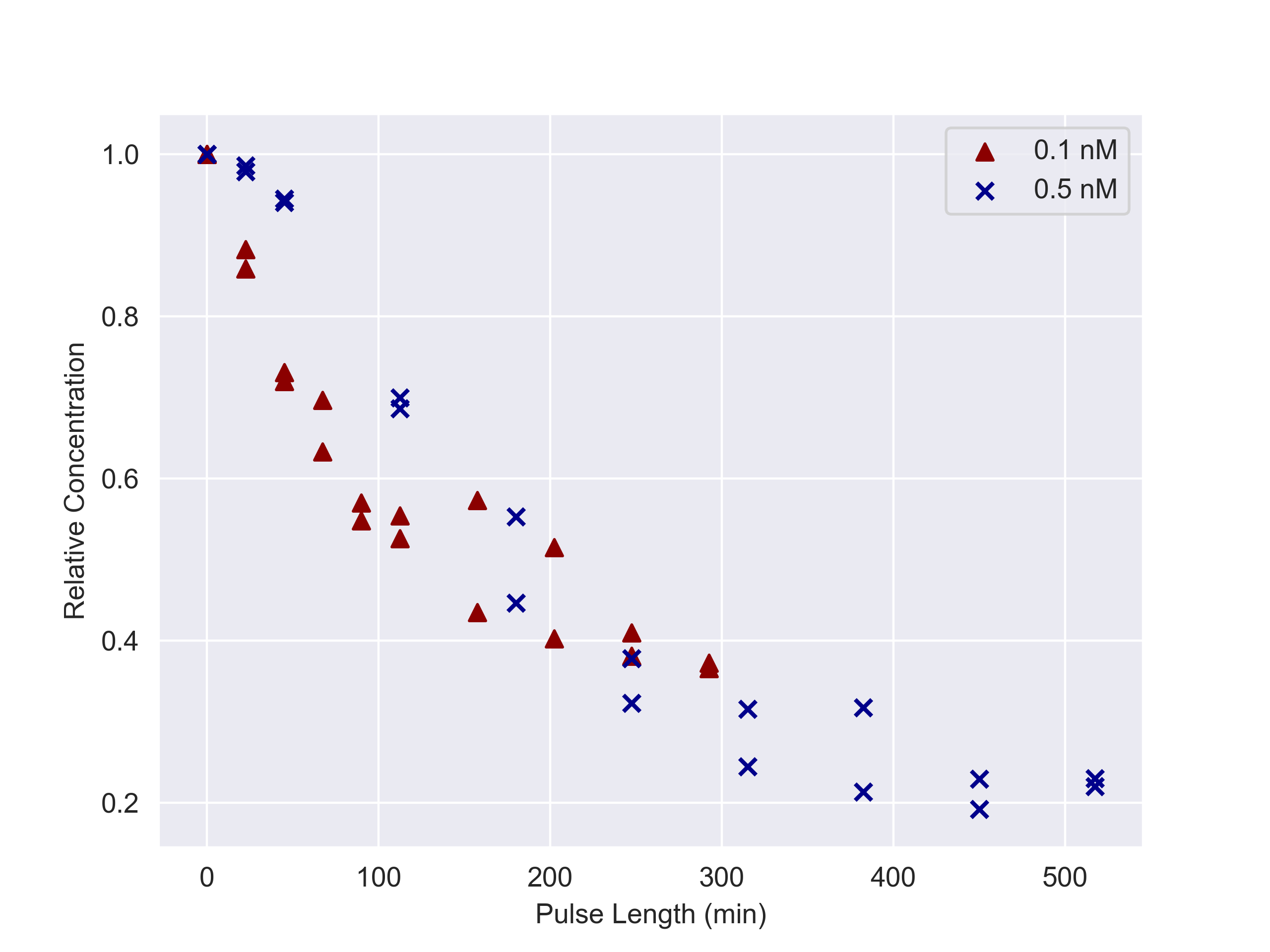

Supplement: Supplementary file 5 — Supplementary Dataset 2 [file 41467_2022_31306_MOESM5_ESM.zip › Individual Simulations Pulse Decoder/134.png]

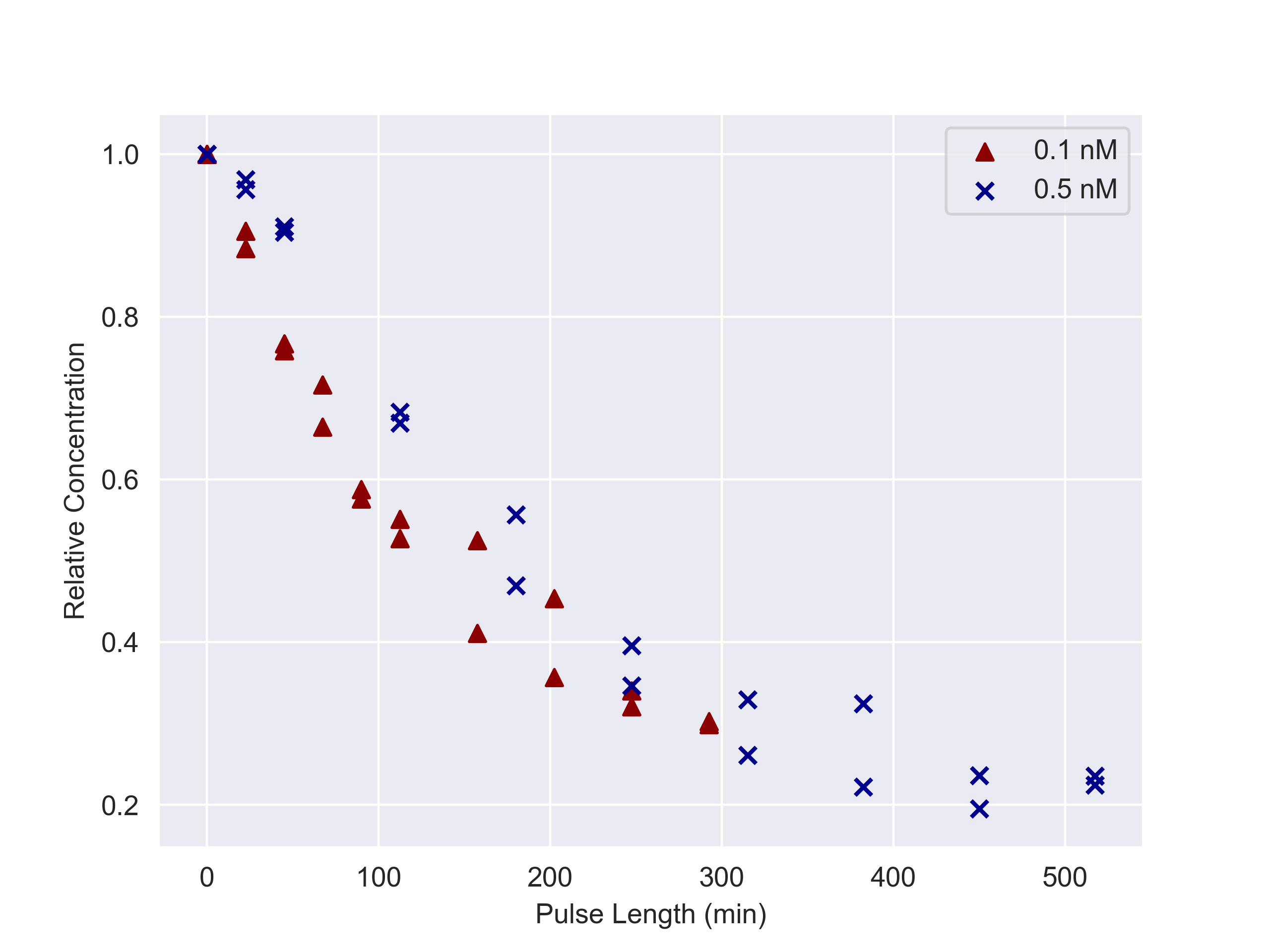

Supplement: Supplementary file 5 — Supplementary Dataset 2 [file 41467_2022_31306_MOESM5_ESM.zip › Individual Simulations Pulse Decoder/135.png]

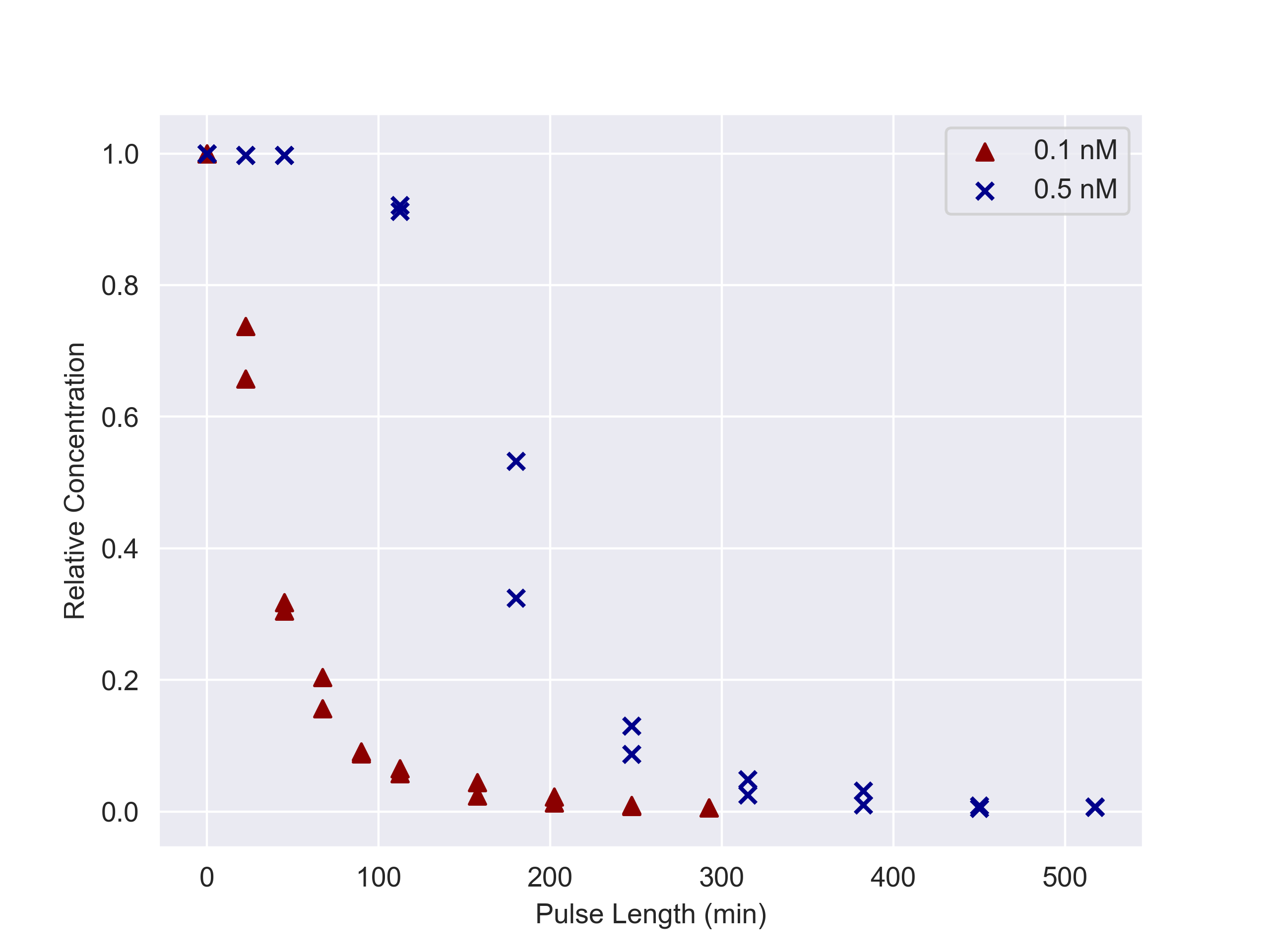

Supplement: Supplementary file 5 — Supplementary Dataset 2 [file 41467_2022_31306_MOESM5_ESM.zip › Individual Simulations Pulse Decoder/136.png]

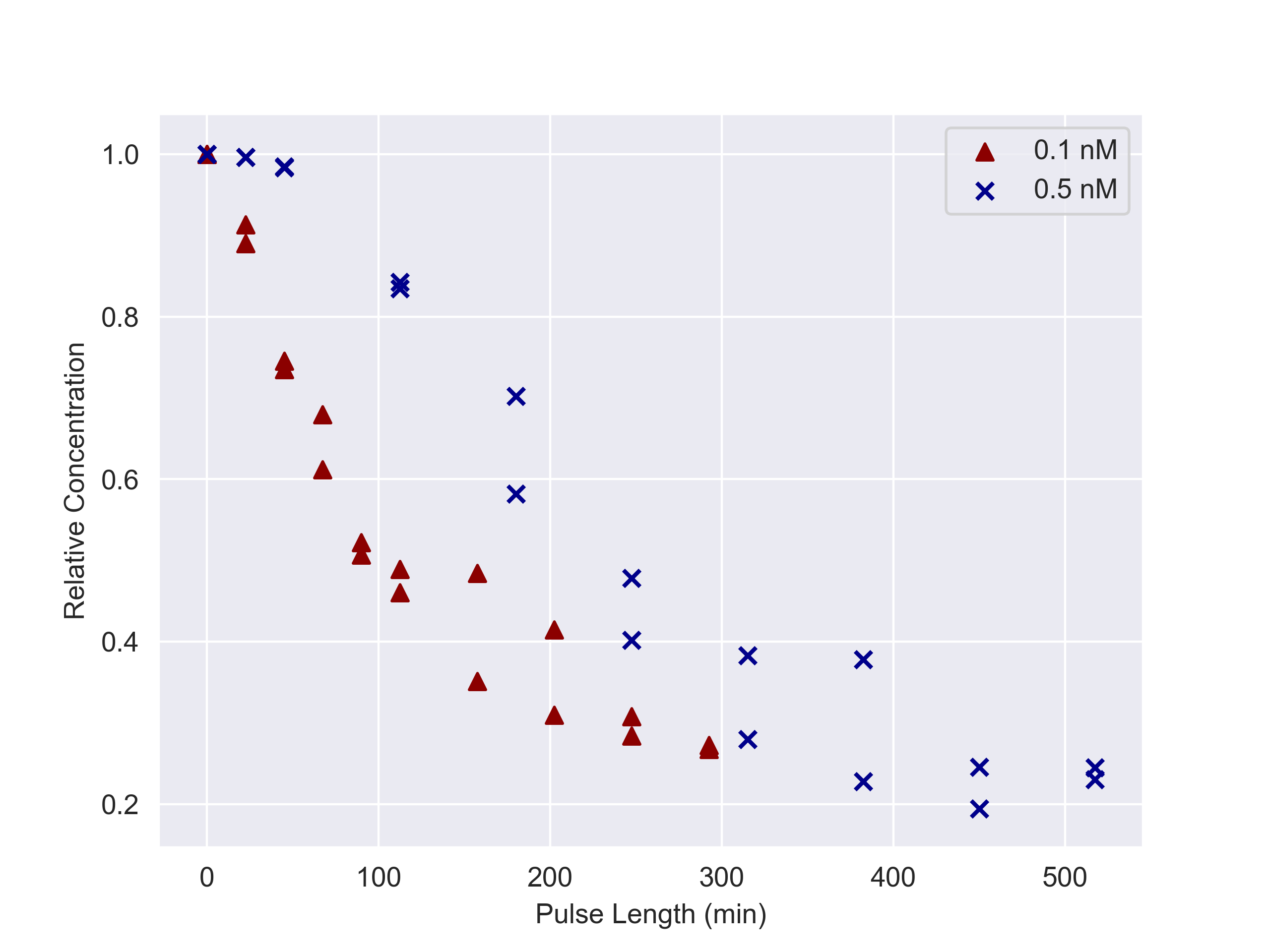

Supplement: Supplementary file 5 — Supplementary Dataset 2 [file 41467_2022_31306_MOESM5_ESM.zip › Individual Simulations Pulse Decoder/137.png]

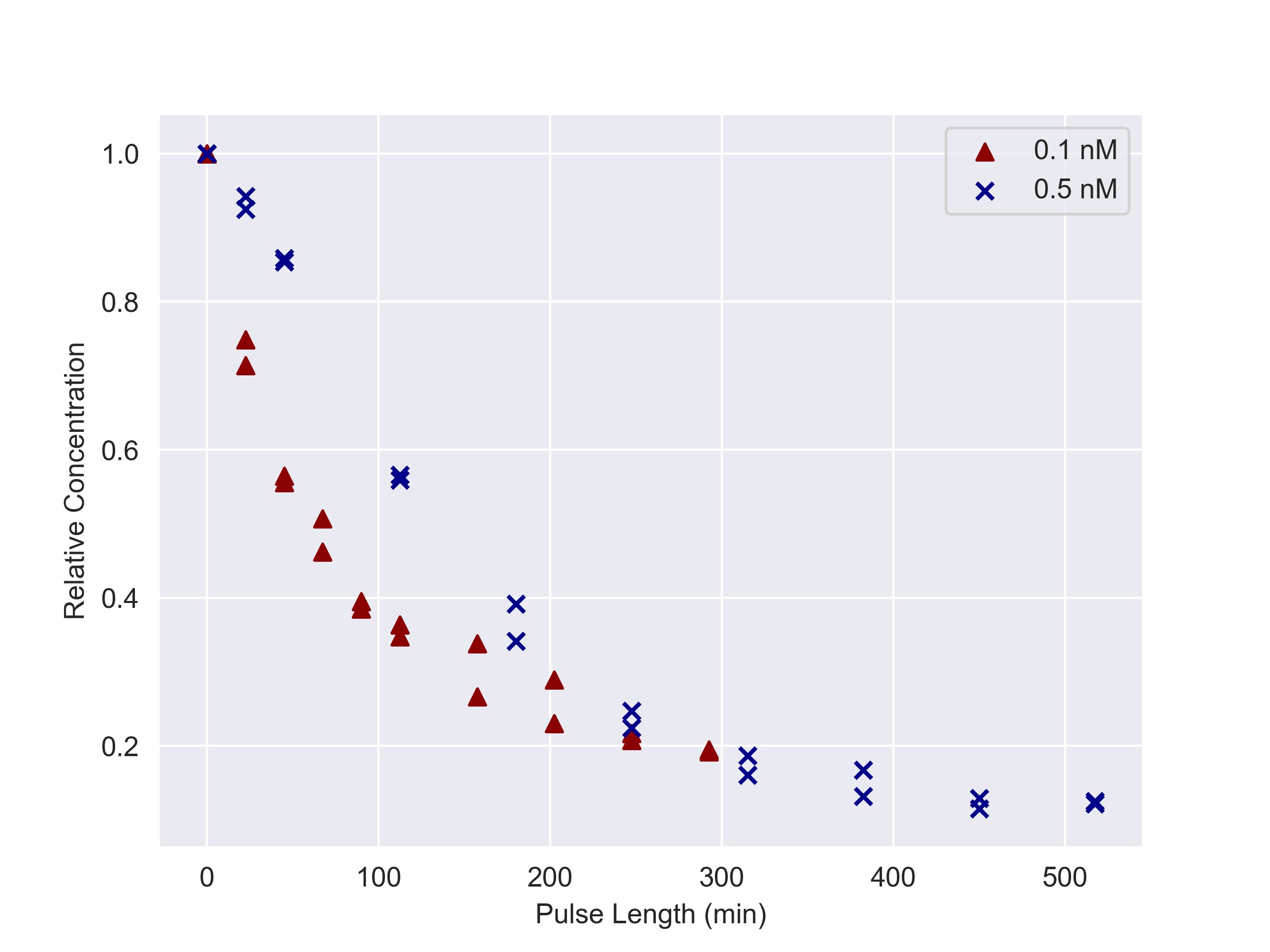

Supplement: Supplementary file 5 — Supplementary Dataset 2 [file 41467_2022_31306_MOESM5_ESM.zip › Individual Simulations Pulse Decoder/138.png]

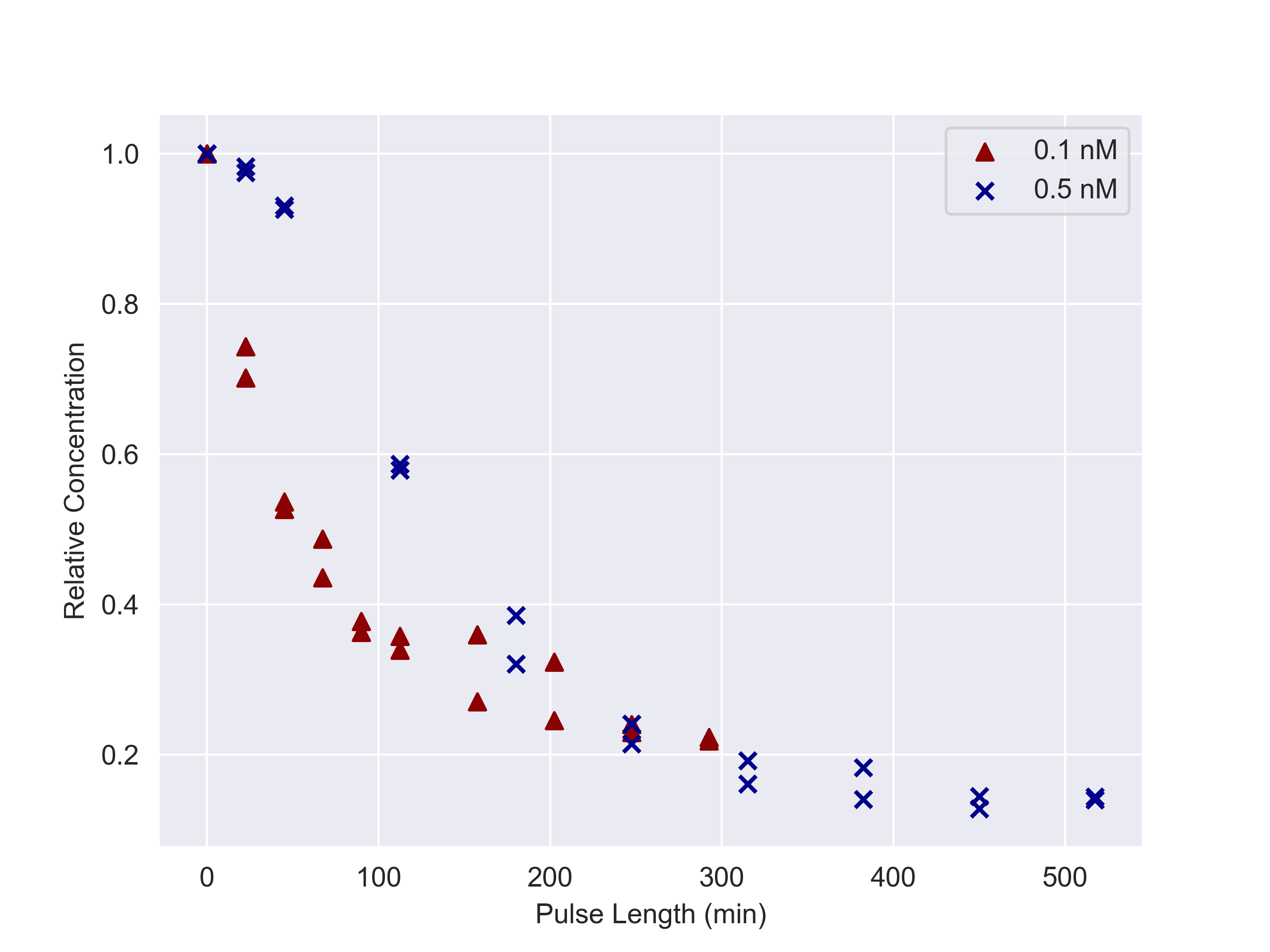

Supplement: Supplementary file 5 — Supplementary Dataset 2 [file 41467_2022_31306_MOESM5_ESM.zip › Individual Simulations Pulse Decoder/139.png]

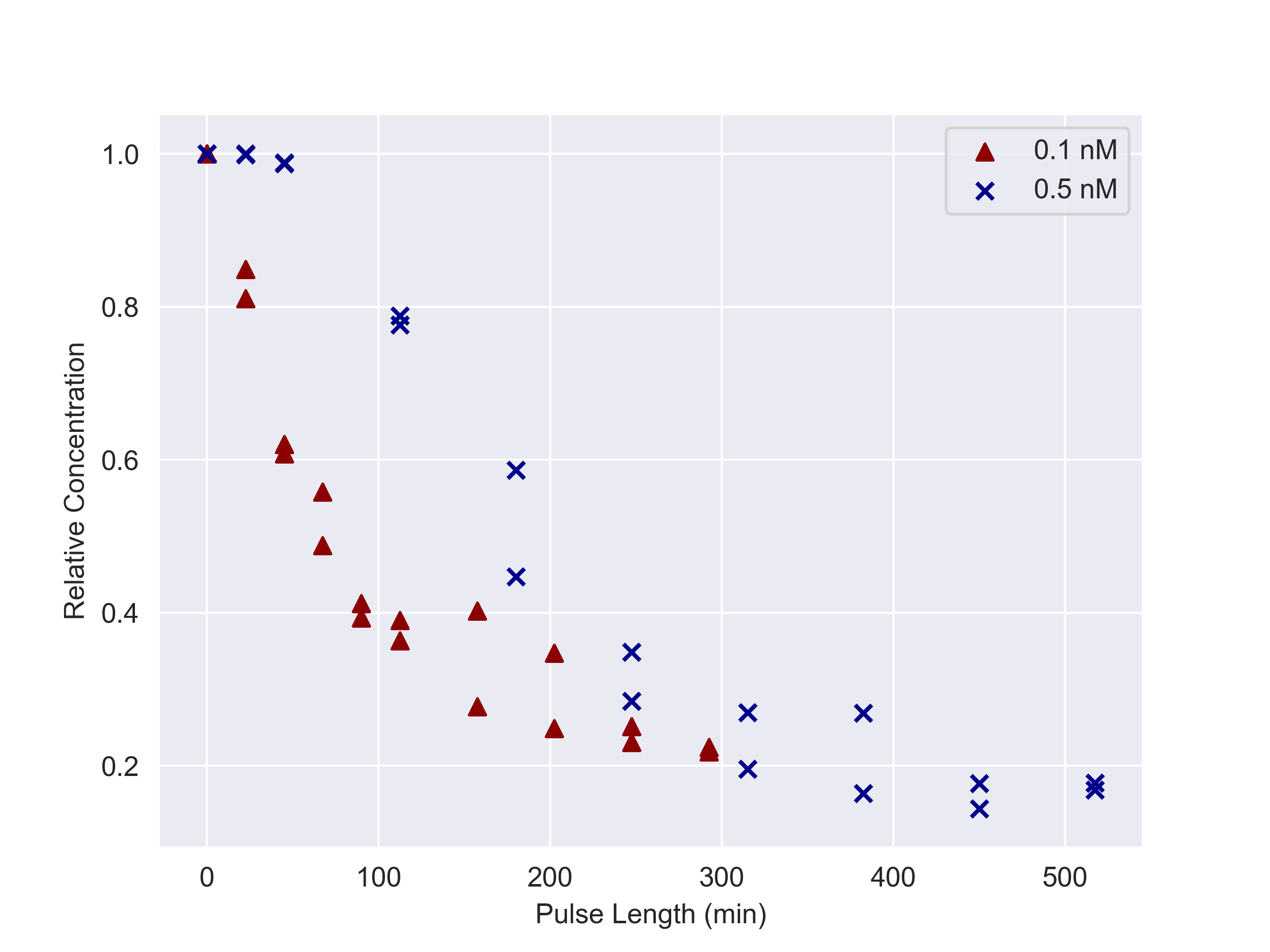

Supplement: Supplementary file 5 — Supplementary Dataset 2 [file 41467_2022_31306_MOESM5_ESM.zip › Individual Simulations Pulse Decoder/14.png]

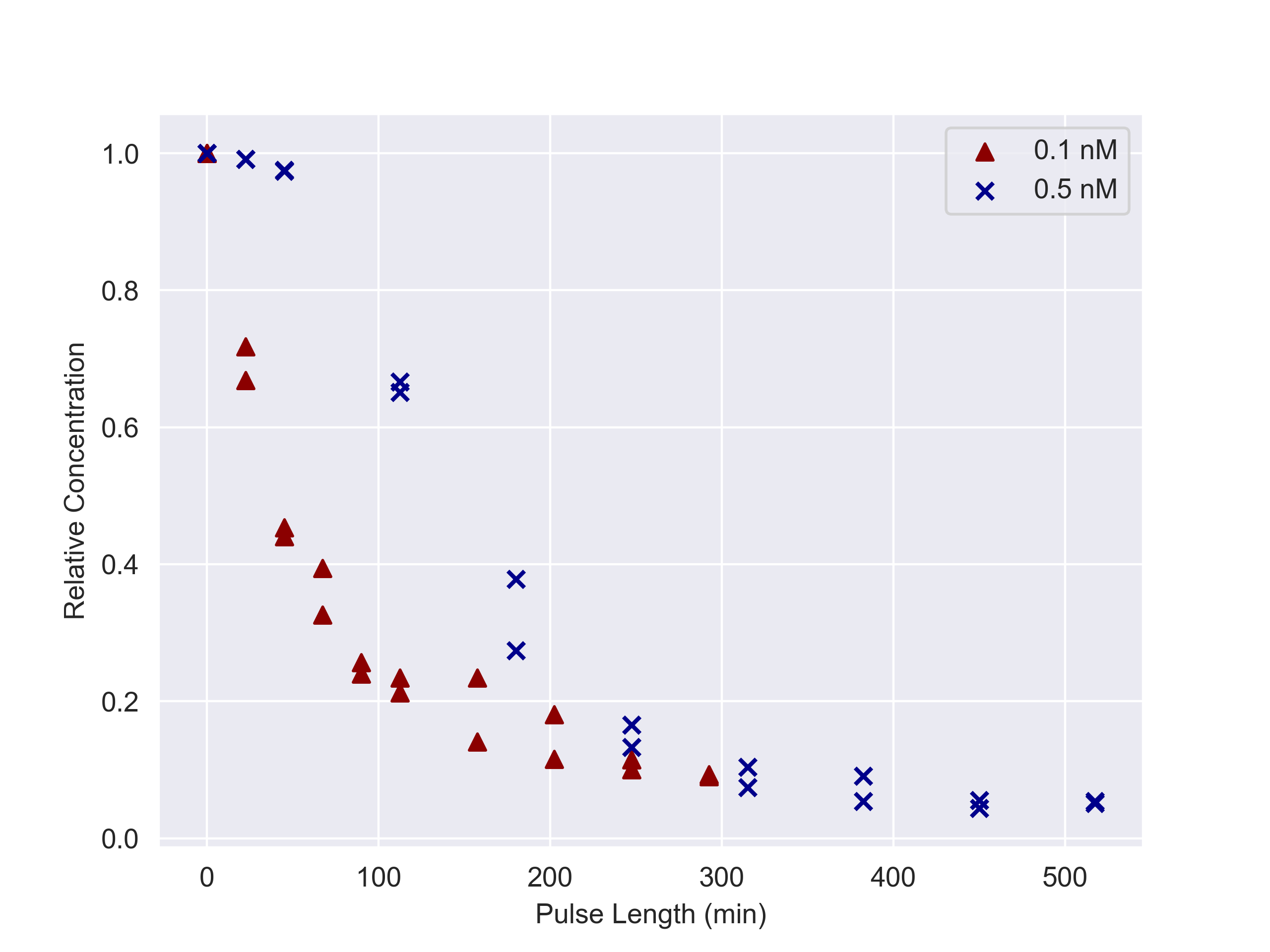

Supplement: Supplementary file 5 — Supplementary Dataset 2 [file 41467_2022_31306_MOESM5_ESM.zip › Individual Simulations Pulse Decoder/140.png]

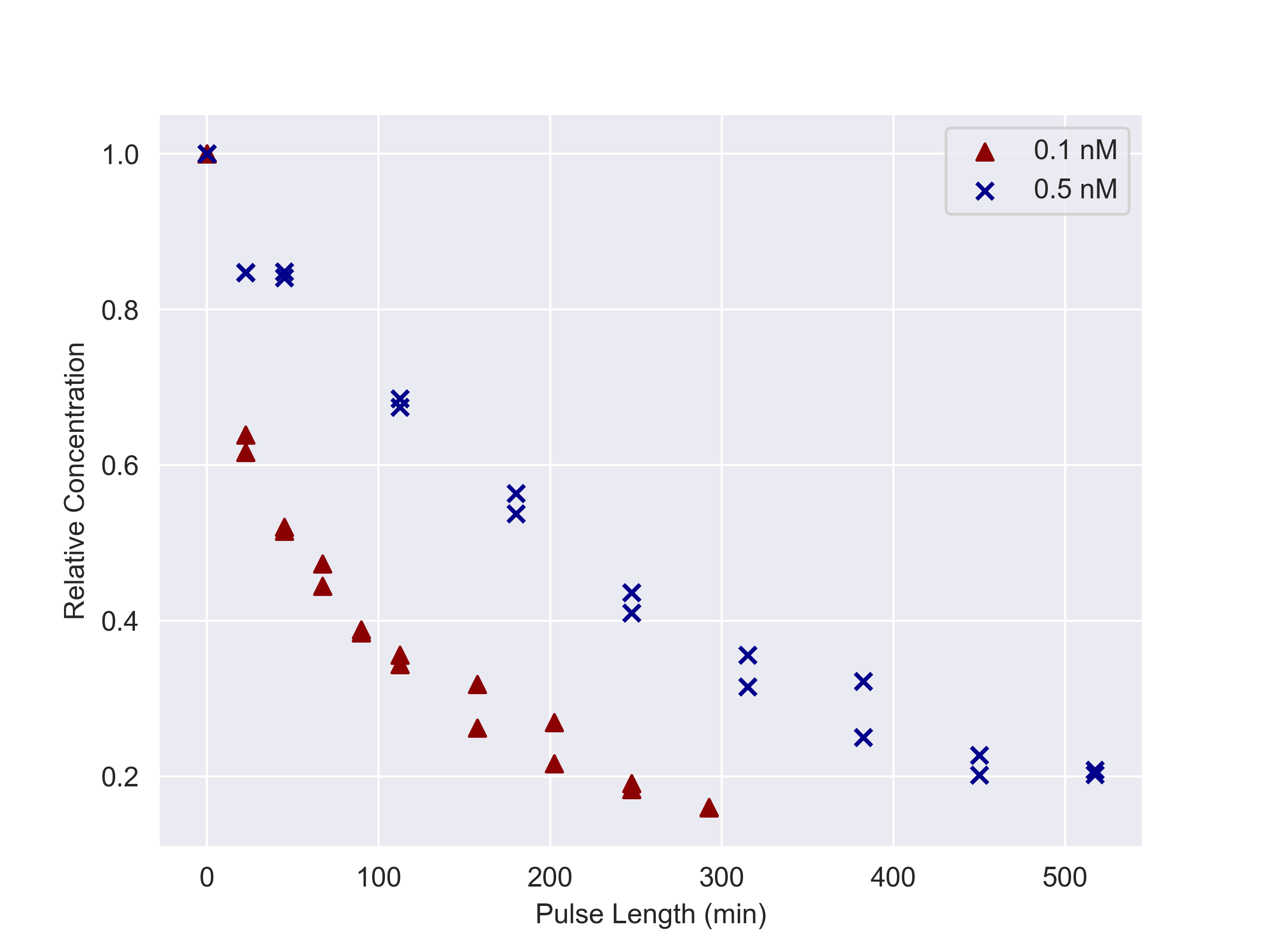

Supplement: Supplementary file 5 — Supplementary Dataset 2 [file 41467_2022_31306_MOESM5_ESM.zip › Individual Simulations Pulse Decoder/141.png]

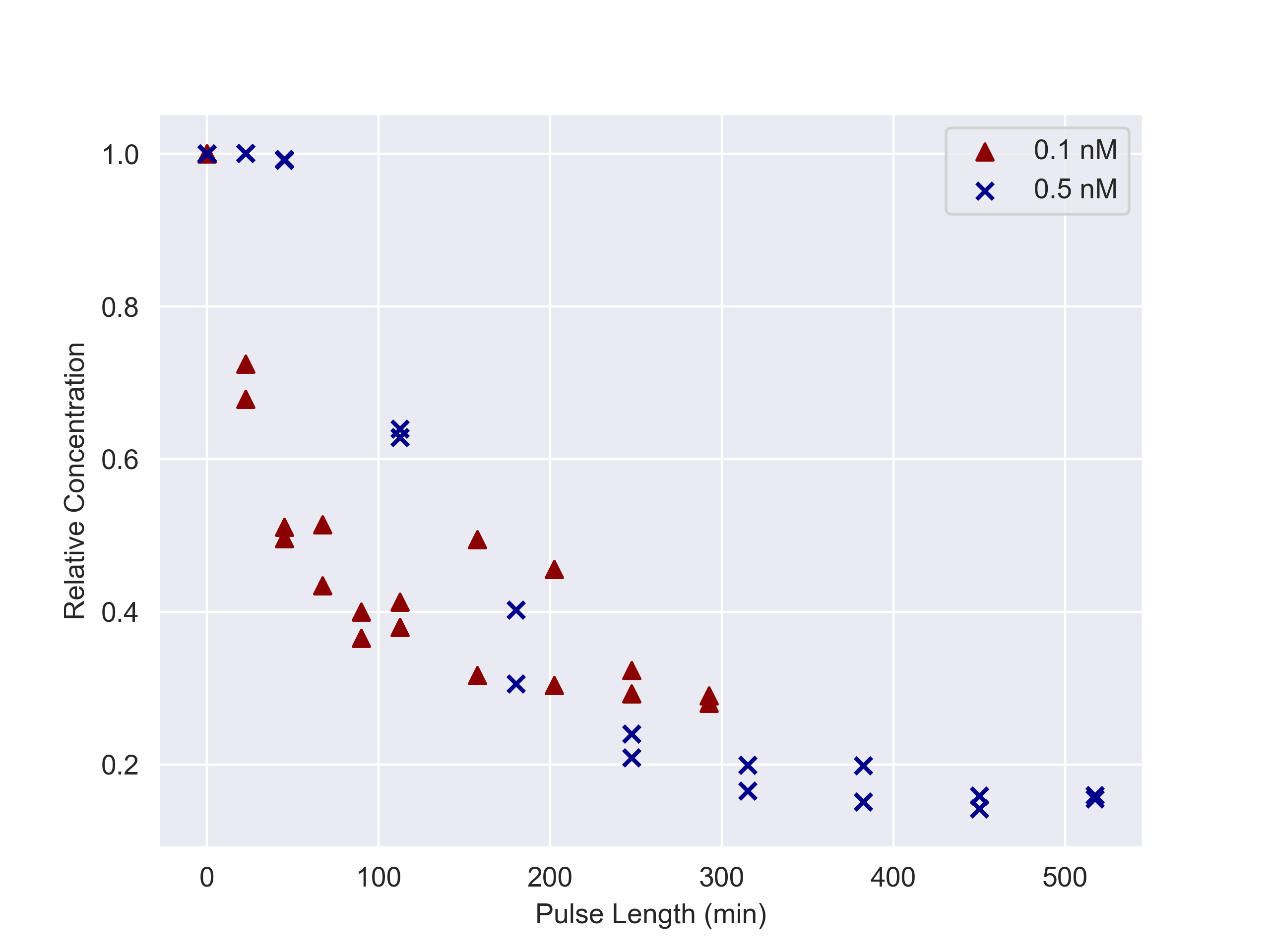

Supplement: Supplementary file 5 — Supplementary Dataset 2 [file 41467_2022_31306_MOESM5_ESM.zip › Individual Simulations Pulse Decoder/142.png]

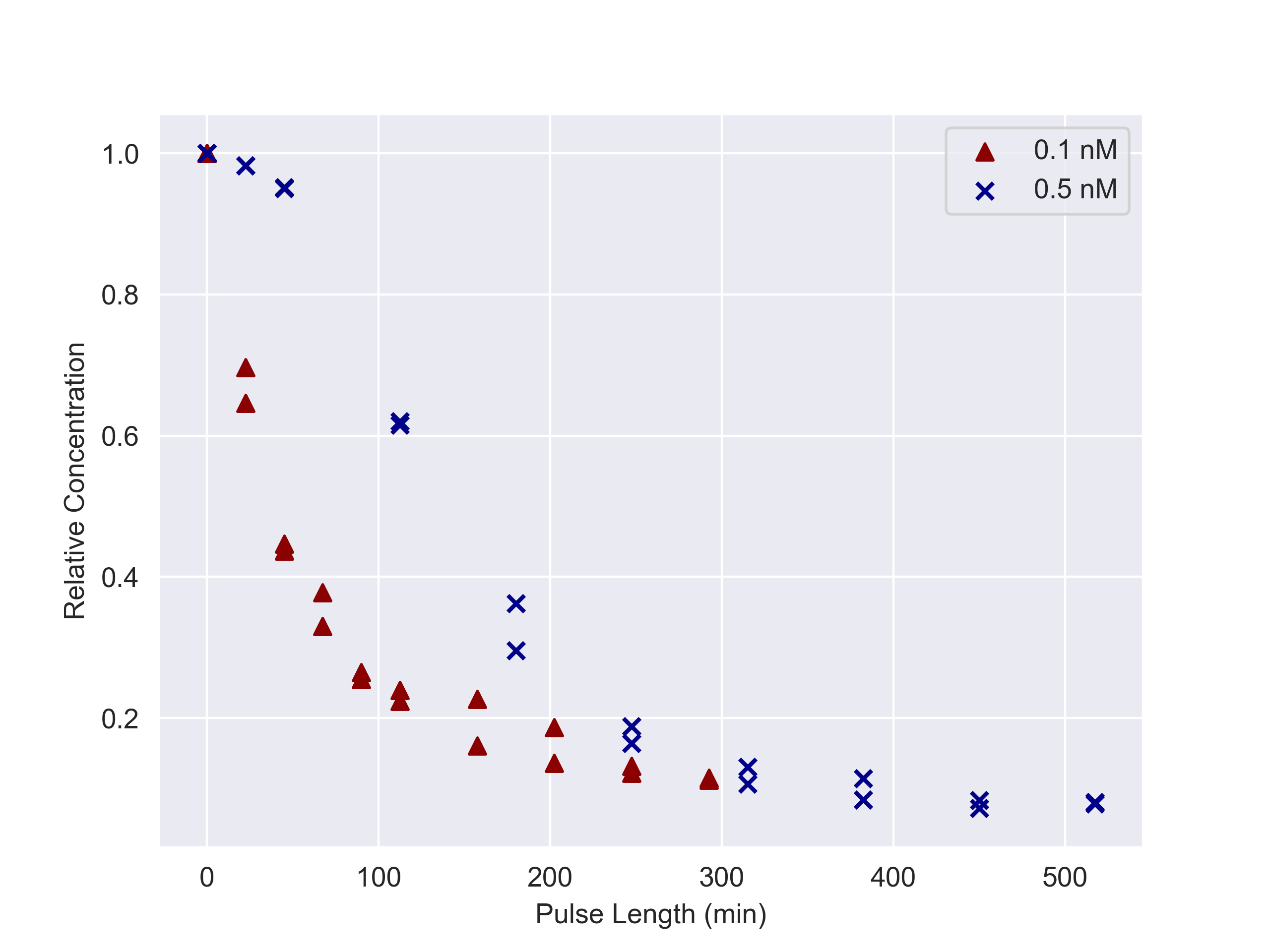

Supplement: Supplementary file 5 — Supplementary Dataset 2 [file 41467_2022_31306_MOESM5_ESM.zip › Individual Simulations Pulse Decoder/143.png]

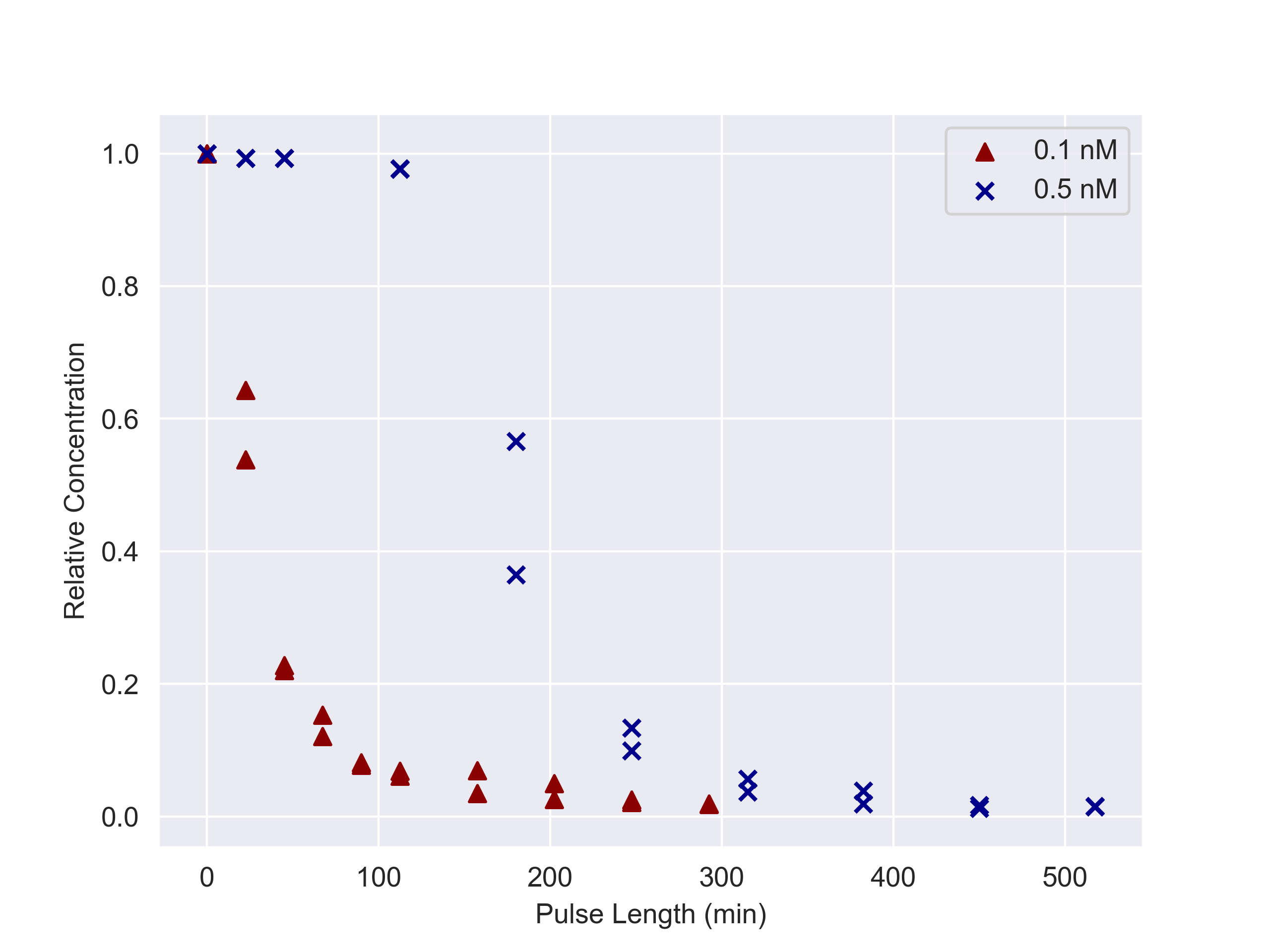

Supplement: Supplementary file 5 — Supplementary Dataset 2 [file 41467_2022_31306_MOESM5_ESM.zip › Individual Simulations Pulse Decoder/144.png]

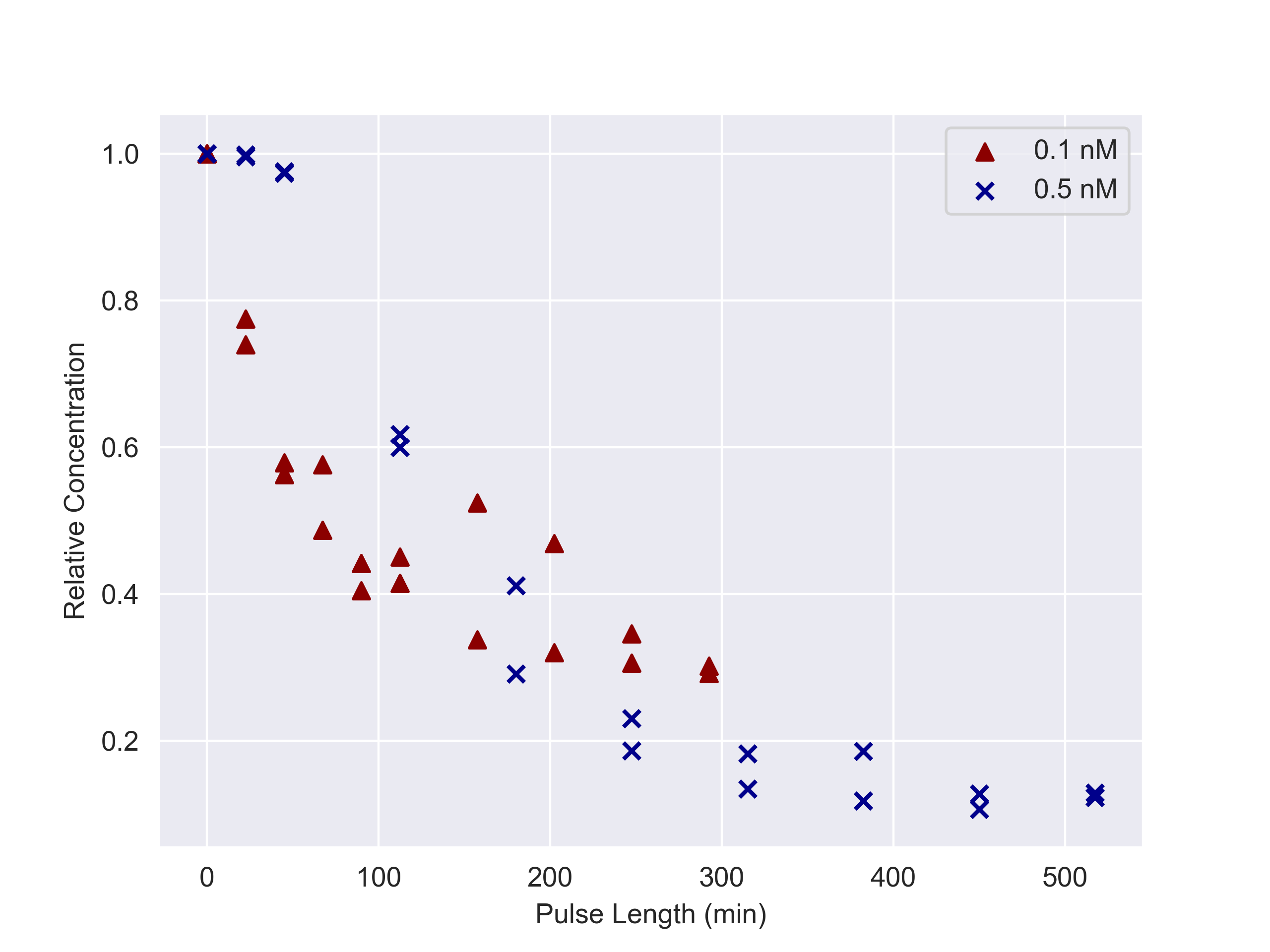

Supplement: Supplementary file 5 — Supplementary Dataset 2 [file 41467_2022_31306_MOESM5_ESM.zip › Individual Simulations Pulse Decoder/145.png]

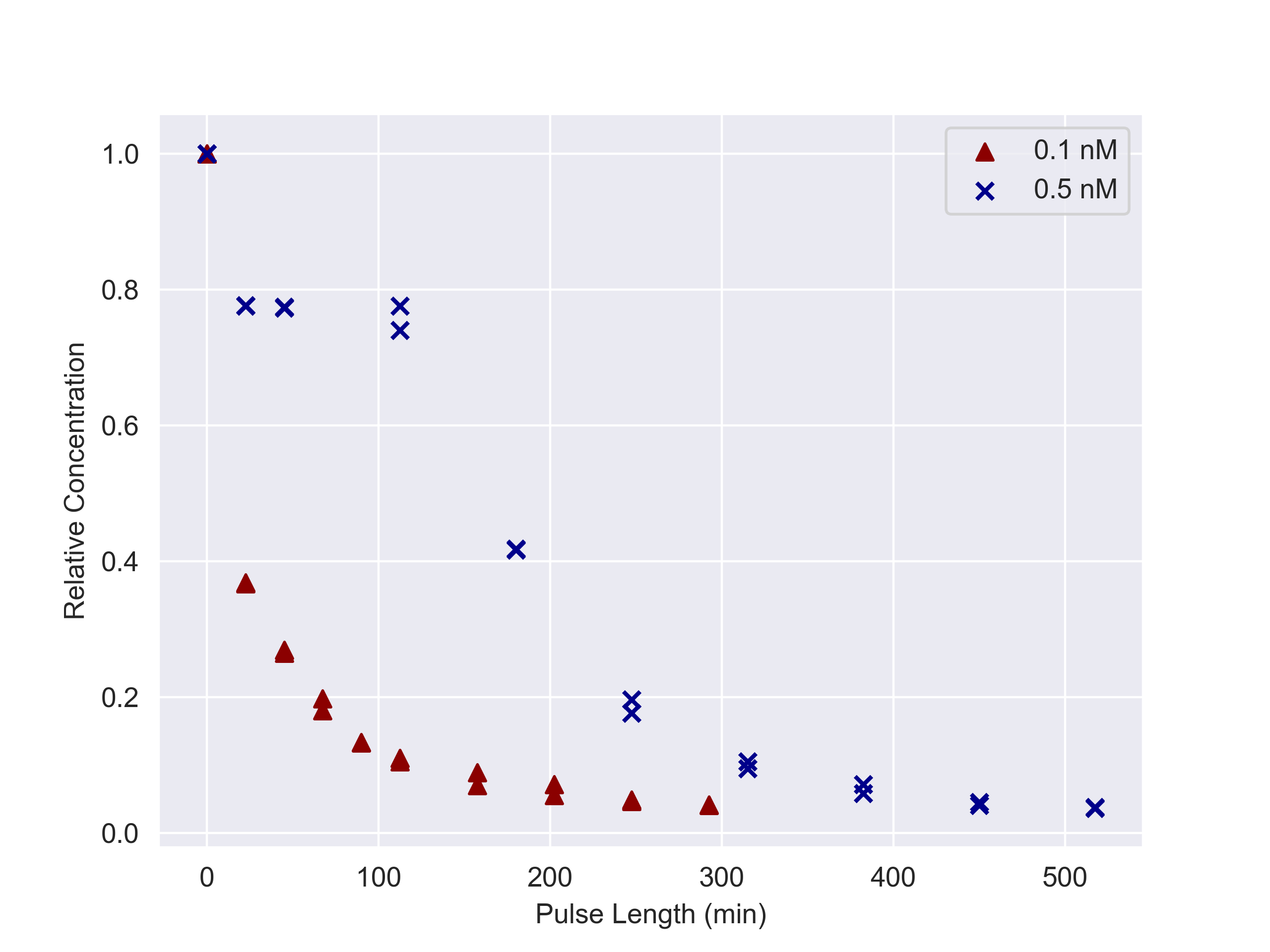

Supplement: Supplementary file 5 — Supplementary Dataset 2 [file 41467_2022_31306_MOESM5_ESM.zip › Individual Simulations Pulse Decoder/146.png]

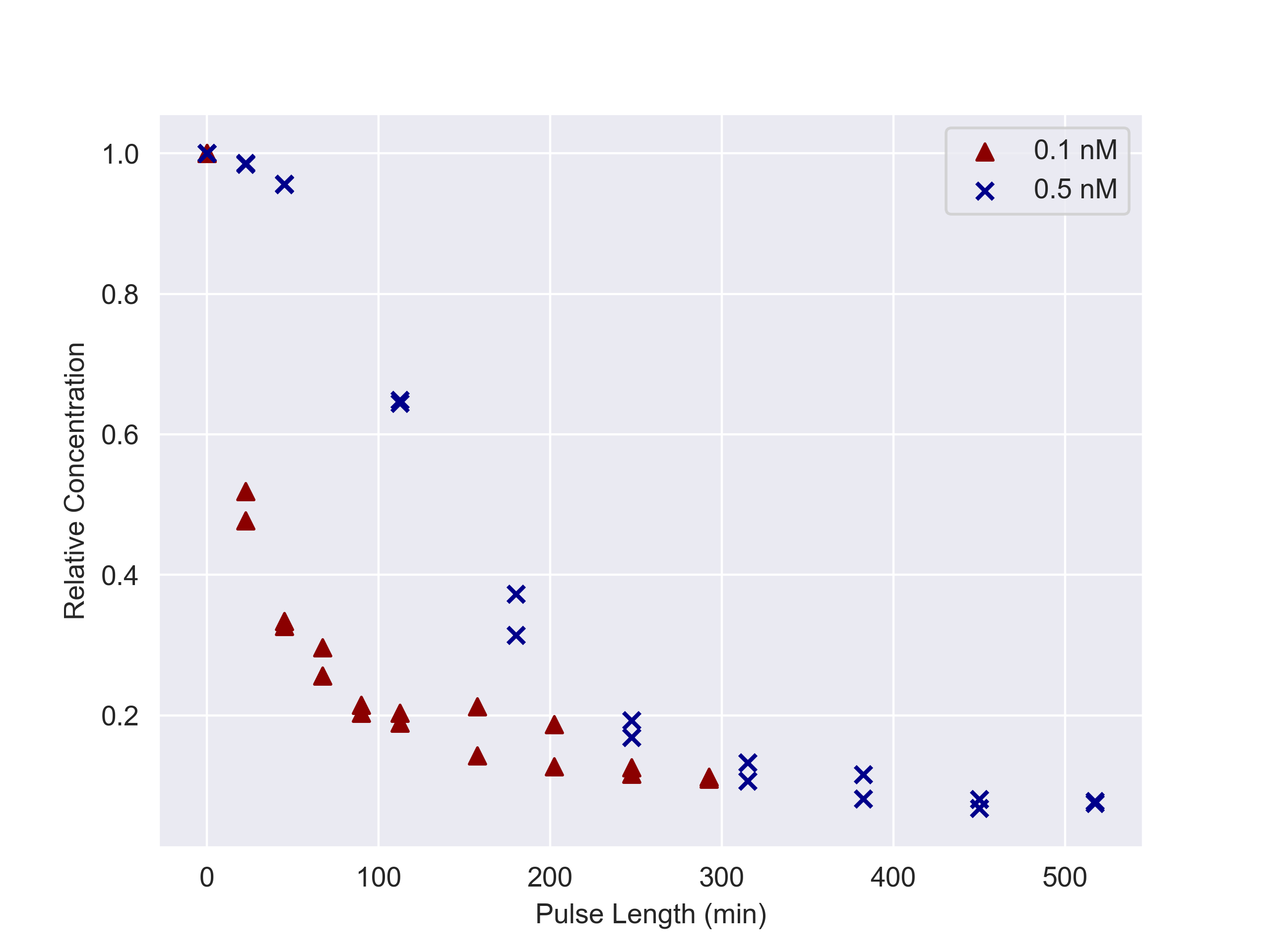

Supplement: Supplementary file 5 — Supplementary Dataset 2 [file 41467_2022_31306_MOESM5_ESM.zip › Individual Simulations Pulse Decoder/147.png]

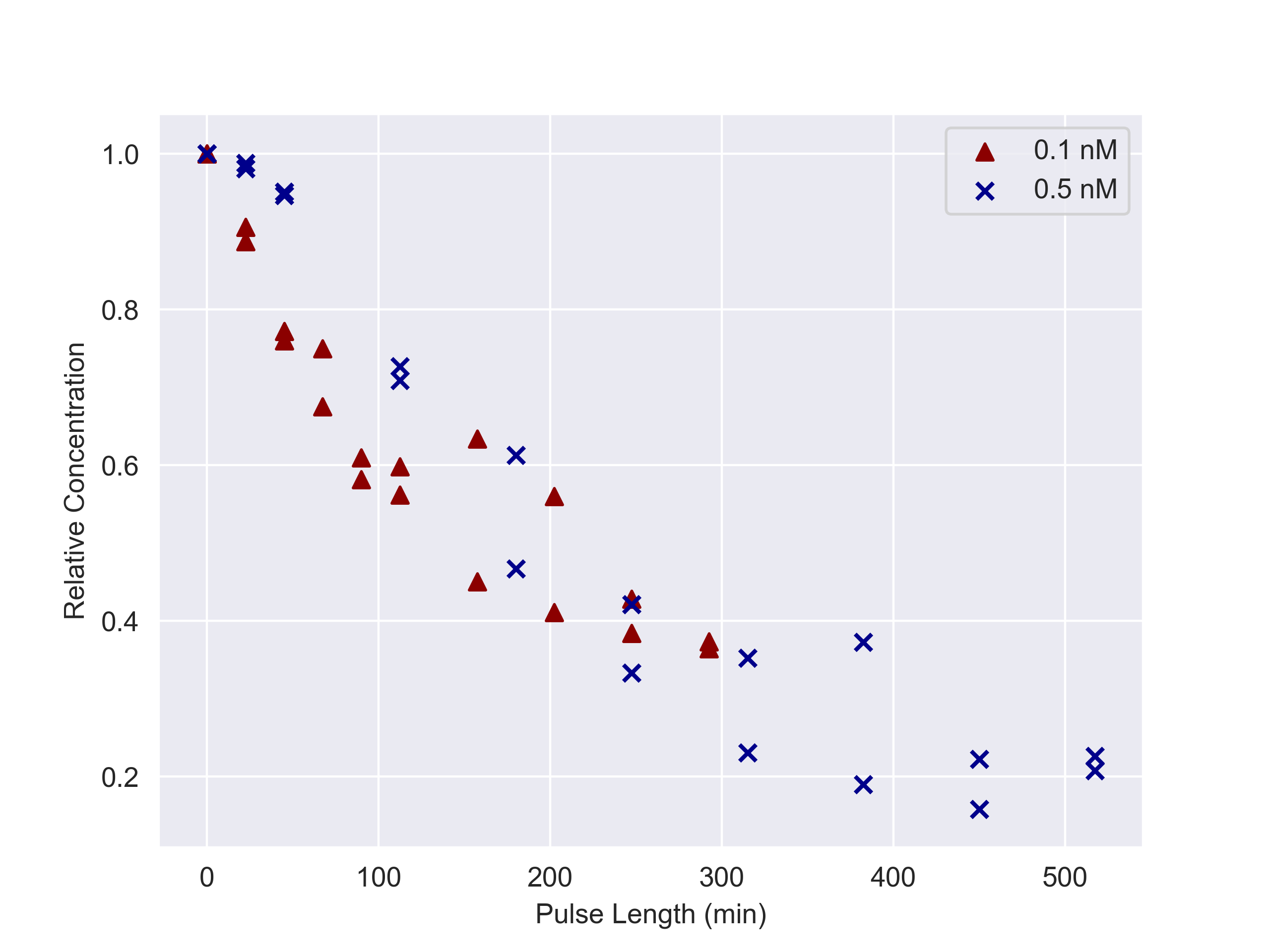

Supplement: Supplementary file 5 — Supplementary Dataset 2 [file 41467_2022_31306_MOESM5_ESM.zip › Individual Simulations Pulse Decoder/148.png]

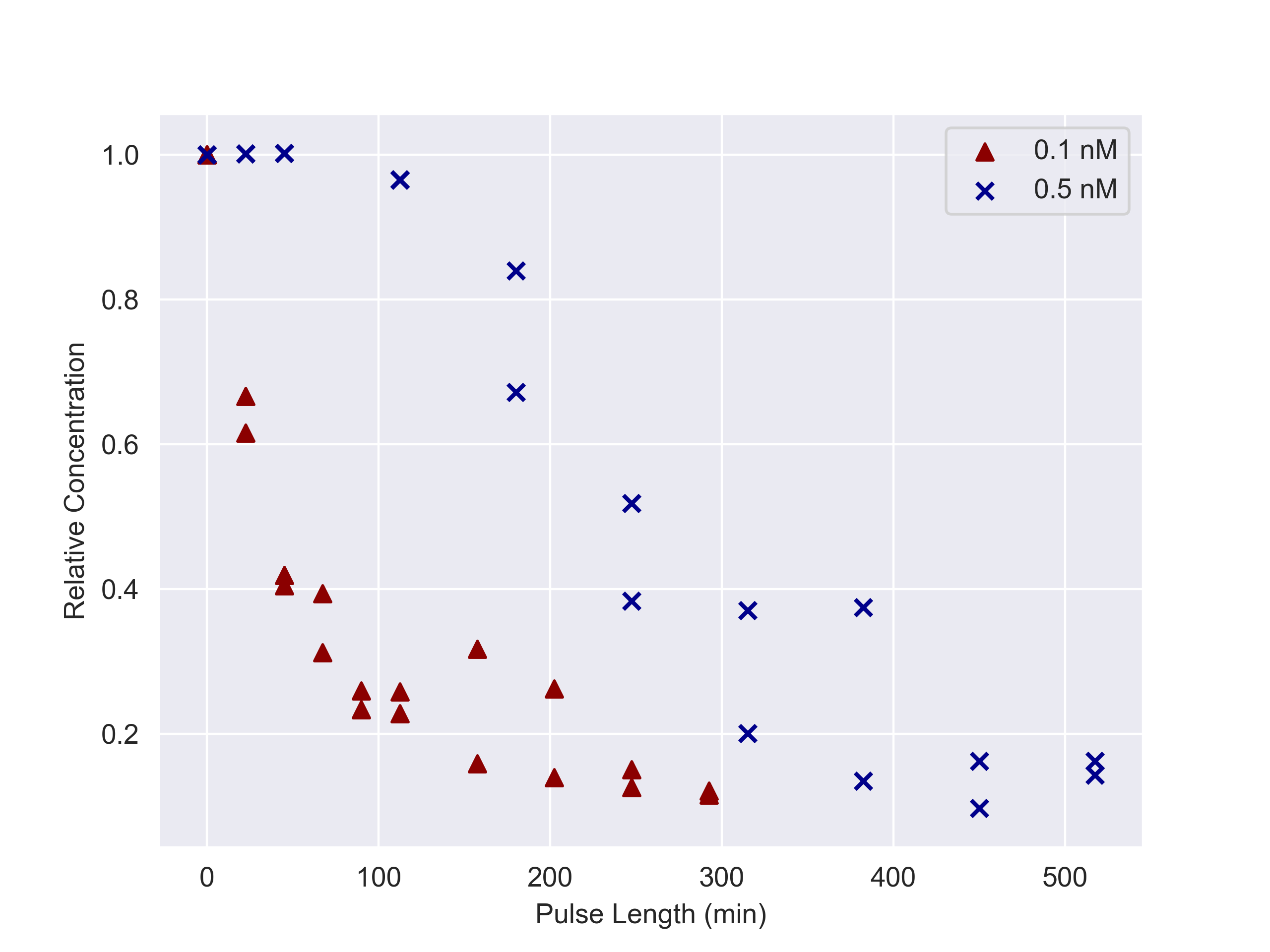

Supplement: Supplementary file 5 — Supplementary Dataset 2 [file 41467_2022_31306_MOESM5_ESM.zip › Individual Simulations Pulse Decoder/149.png]

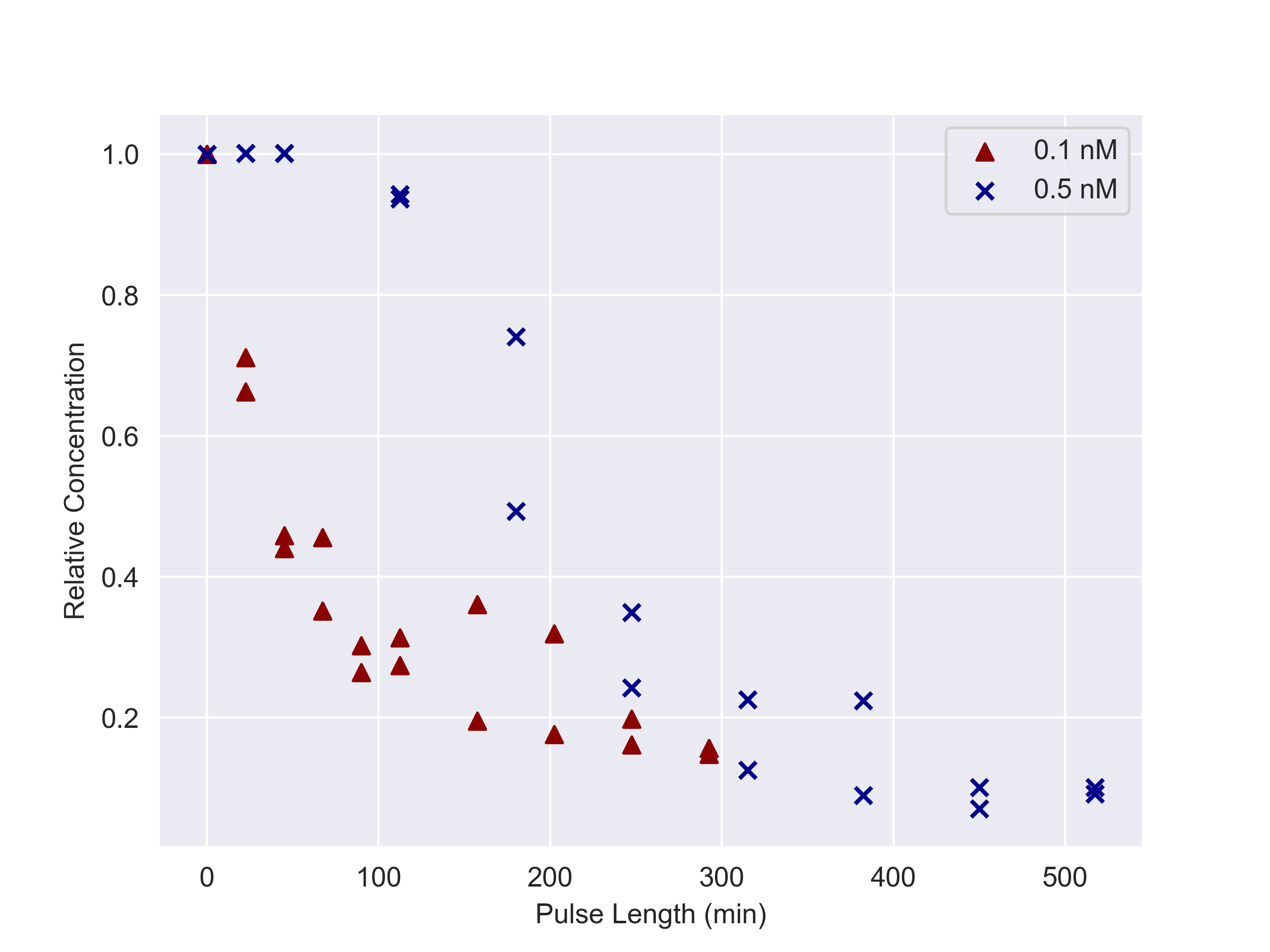

Supplement: Supplementary file 5 — Supplementary Dataset 2 [file 41467_2022_31306_MOESM5_ESM.zip › Individual Simulations Pulse Decoder/15.png]

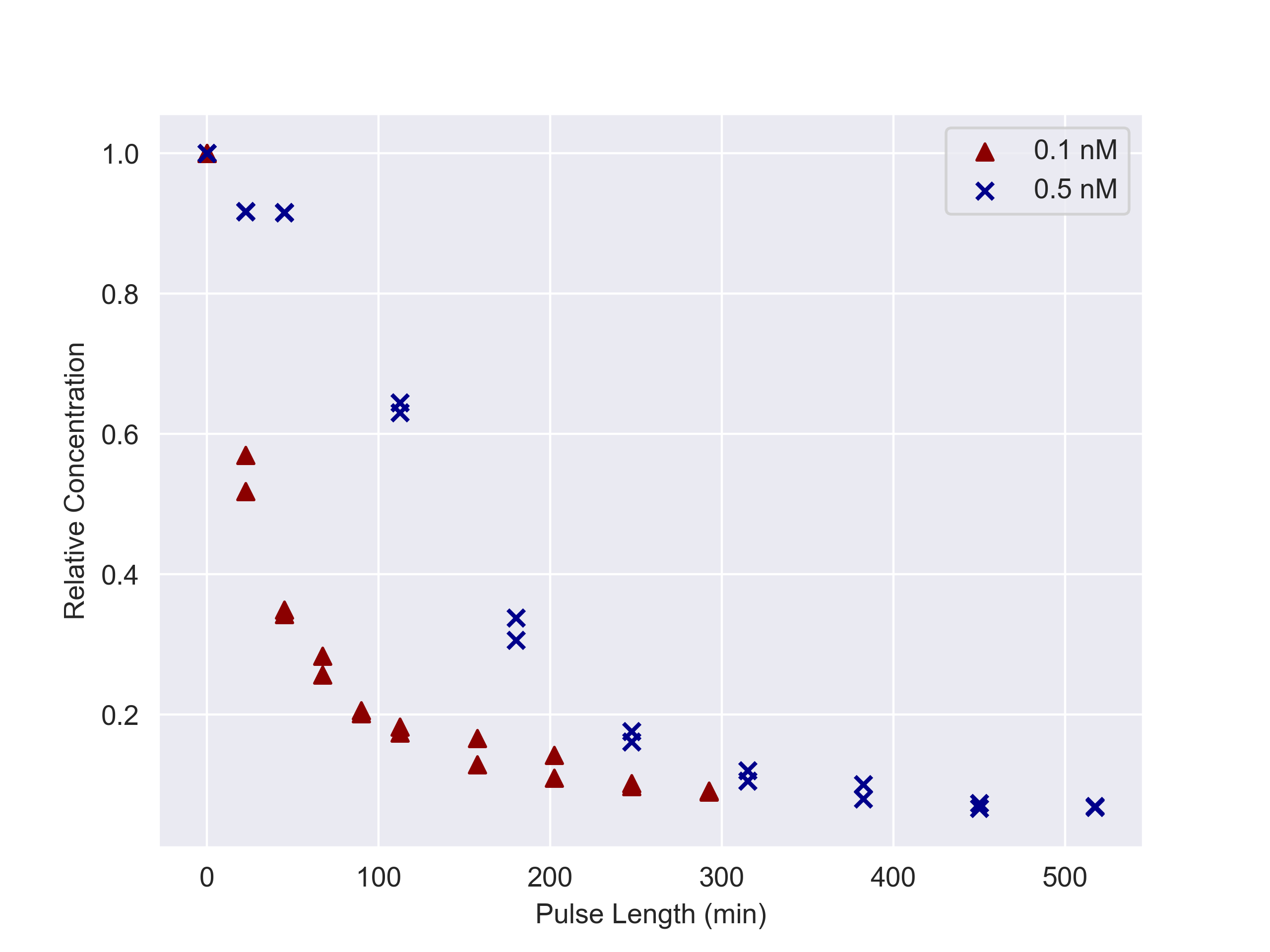

Supplement: Supplementary file 5 — Supplementary Dataset 2 [file 41467_2022_31306_MOESM5_ESM.zip › Individual Simulations Pulse Decoder/150.png]

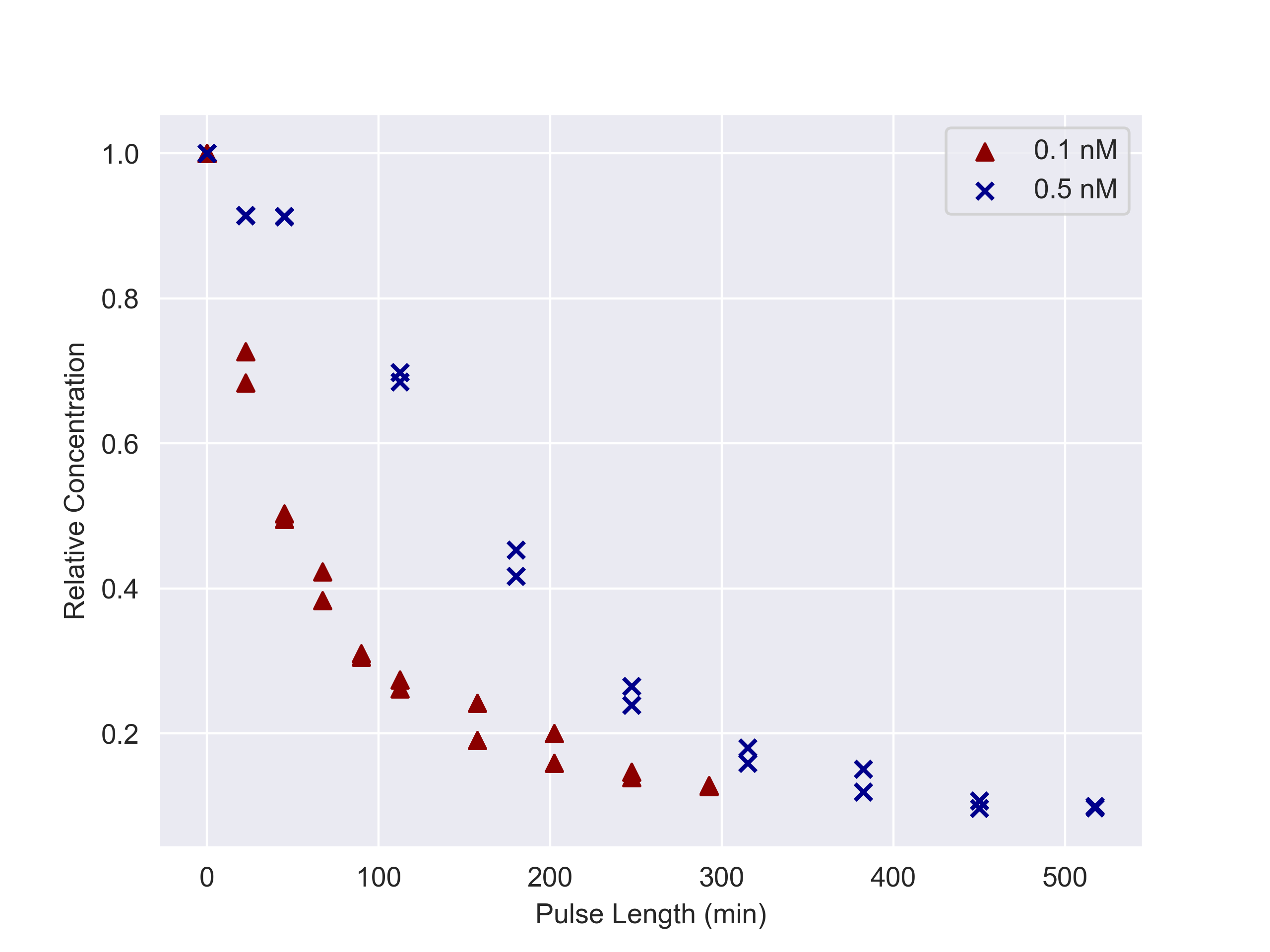

Supplement: Supplementary file 5 — Supplementary Dataset 2 [file 41467_2022_31306_MOESM5_ESM.zip › Individual Simulations Pulse Decoder/16.png]

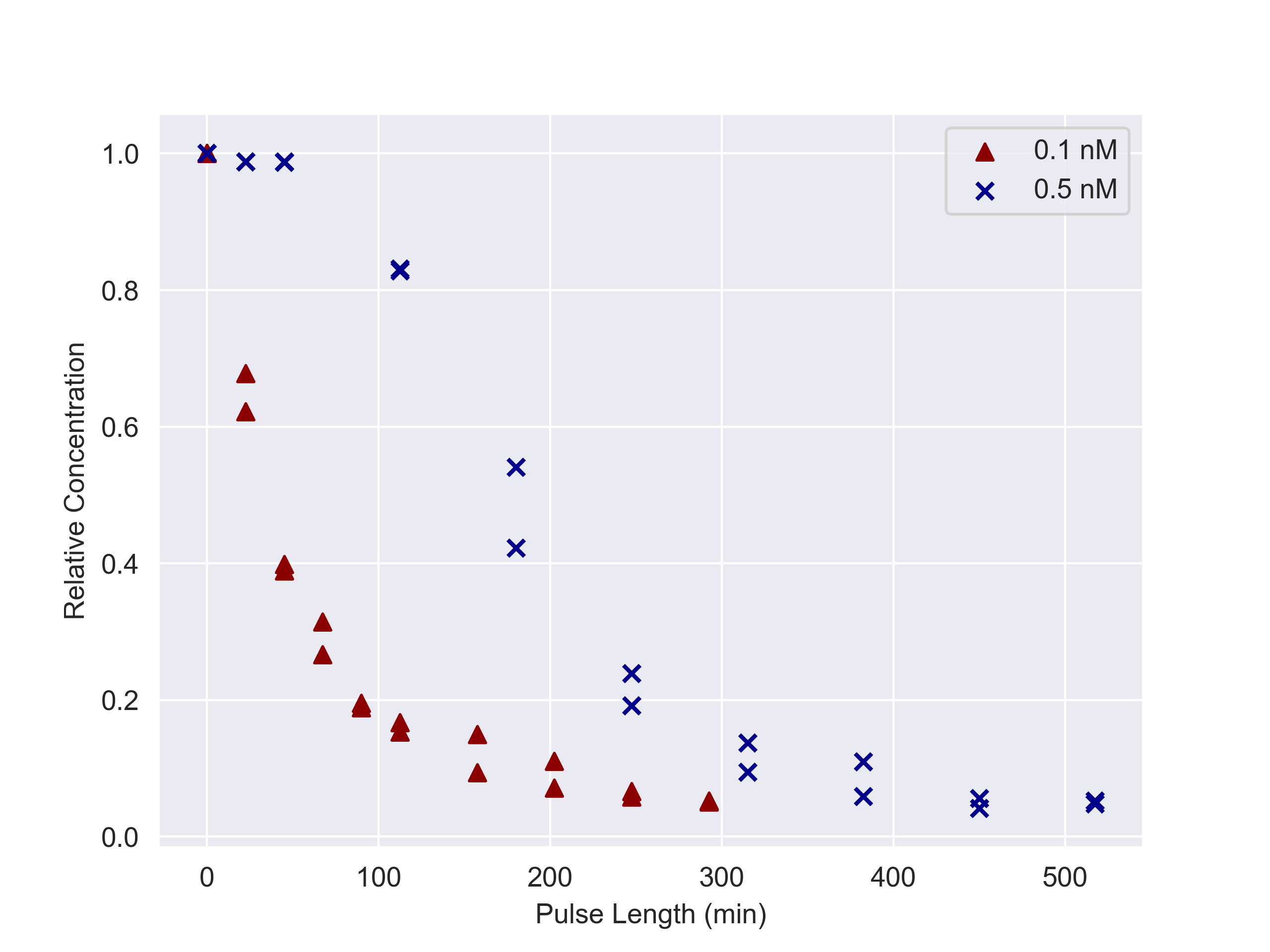

Supplement: Supplementary file 5 — Supplementary Dataset 2 [file 41467_2022_31306_MOESM5_ESM.zip › Individual Simulations Pulse Decoder/17.png]

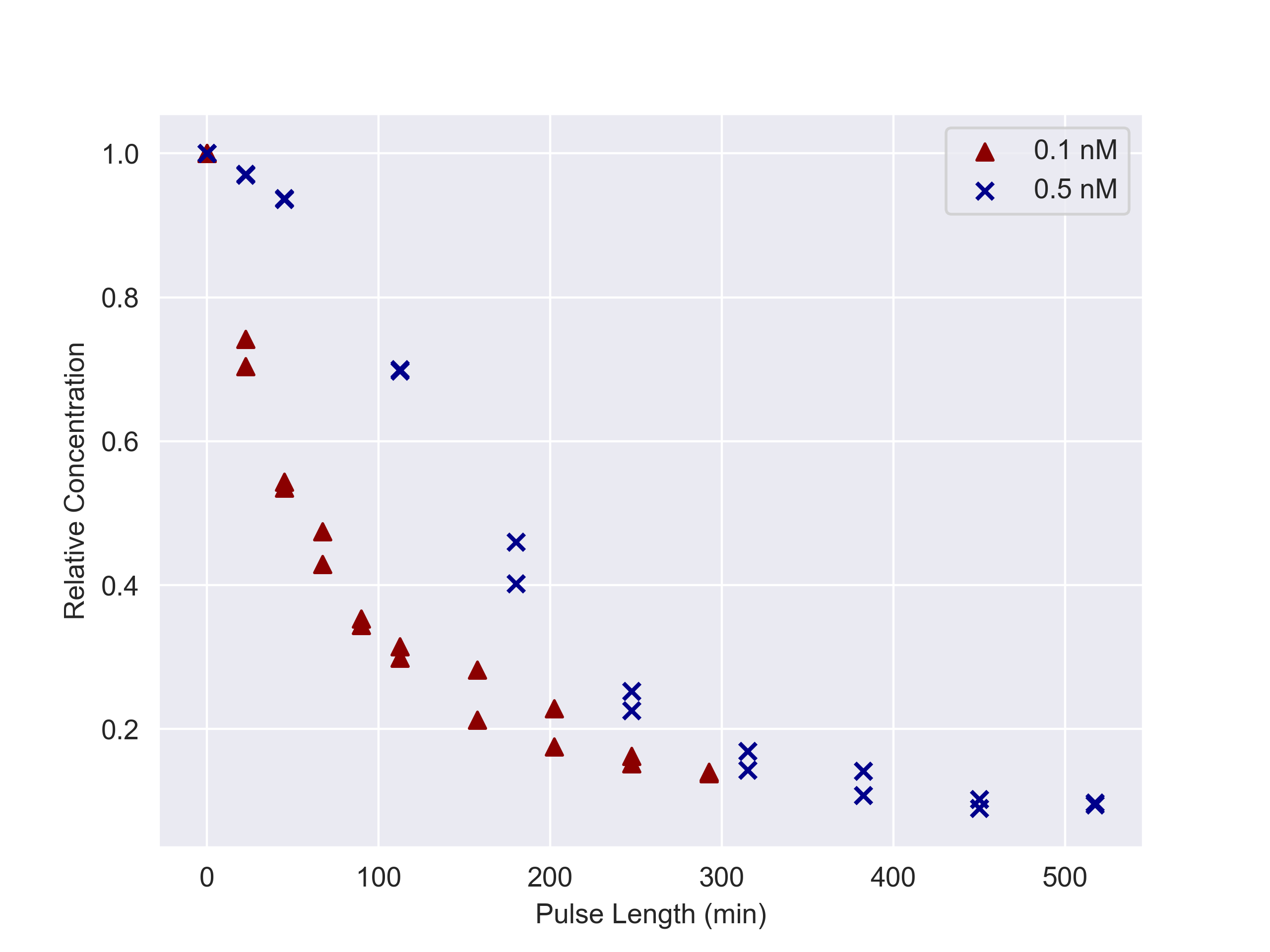

Supplement: Supplementary file 5 — Supplementary Dataset 2 [file 41467_2022_31306_MOESM5_ESM.zip › Individual Simulations Pulse Decoder/18.png]

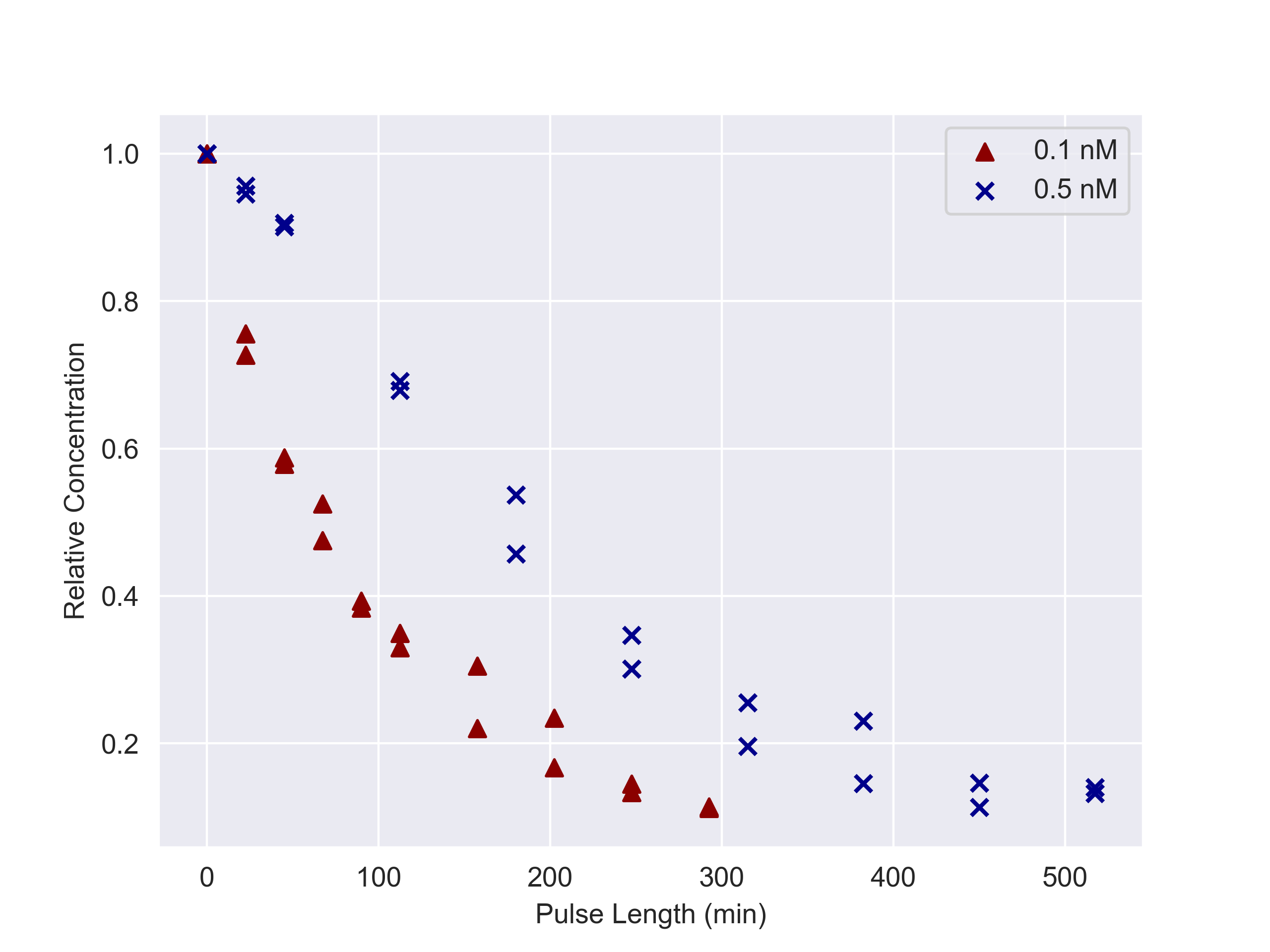

Supplement: Supplementary file 5 — Supplementary Dataset 2 [file 41467_2022_31306_MOESM5_ESM.zip › Individual Simulations Pulse Decoder/19.png]

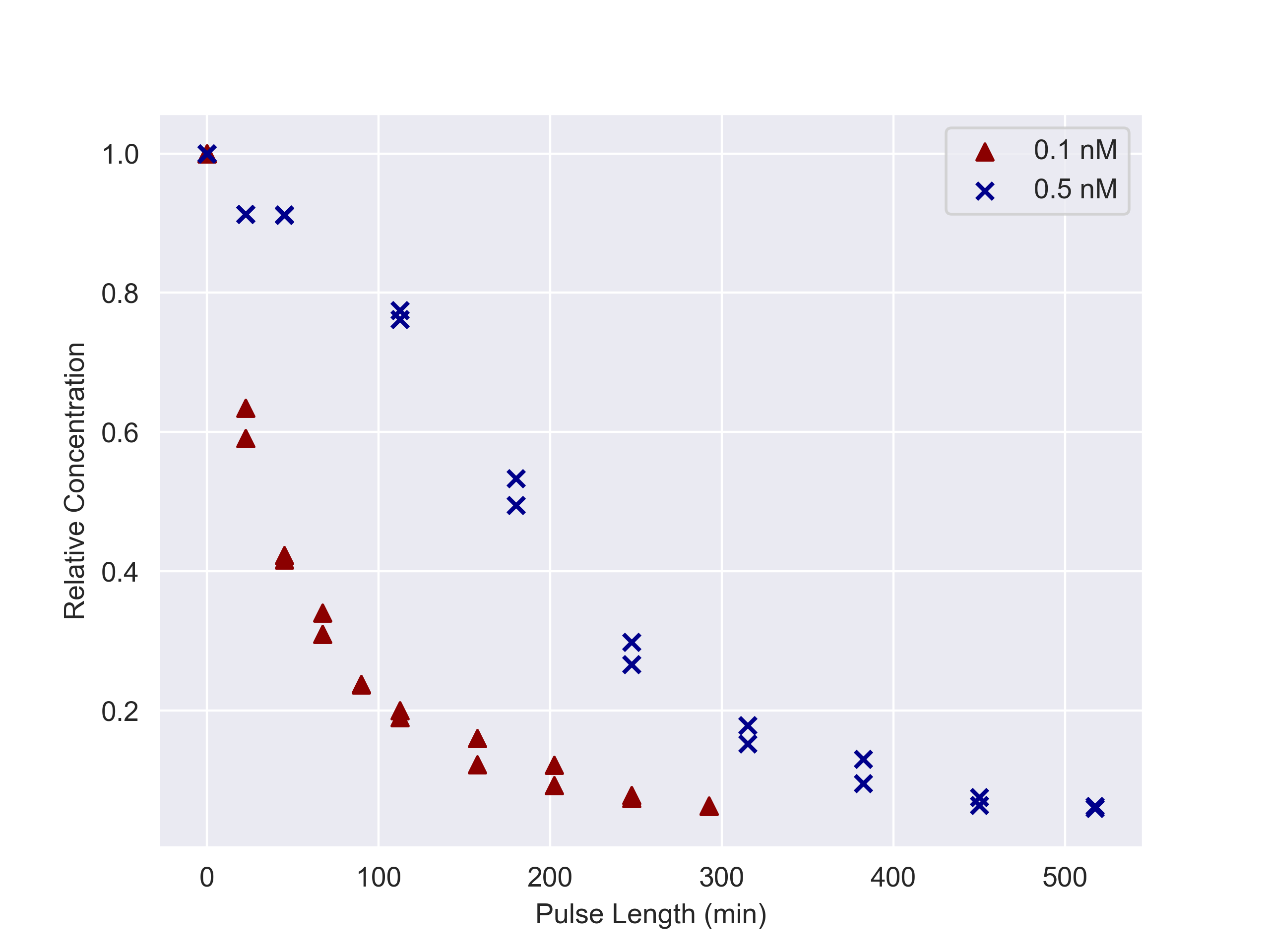

Supplement: Supplementary file 5 — Supplementary Dataset 2 [file 41467_2022_31306_MOESM5_ESM.zip › Individual Simulations Pulse Decoder/2.png]

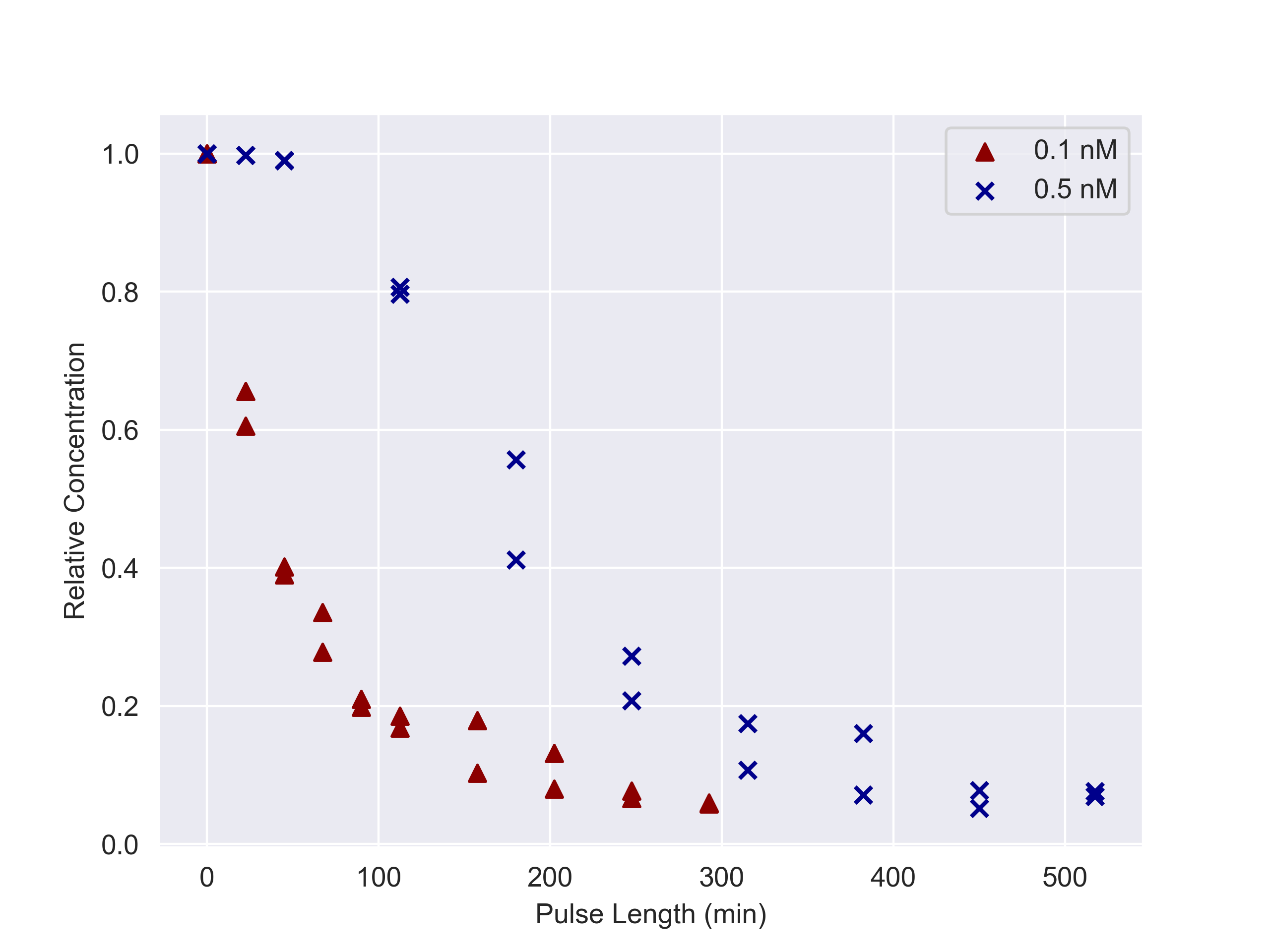

Supplement: Supplementary file 5 — Supplementary Dataset 2 [file 41467_2022_31306_MOESM5_ESM.zip › Individual Simulations Pulse Decoder/20.png]

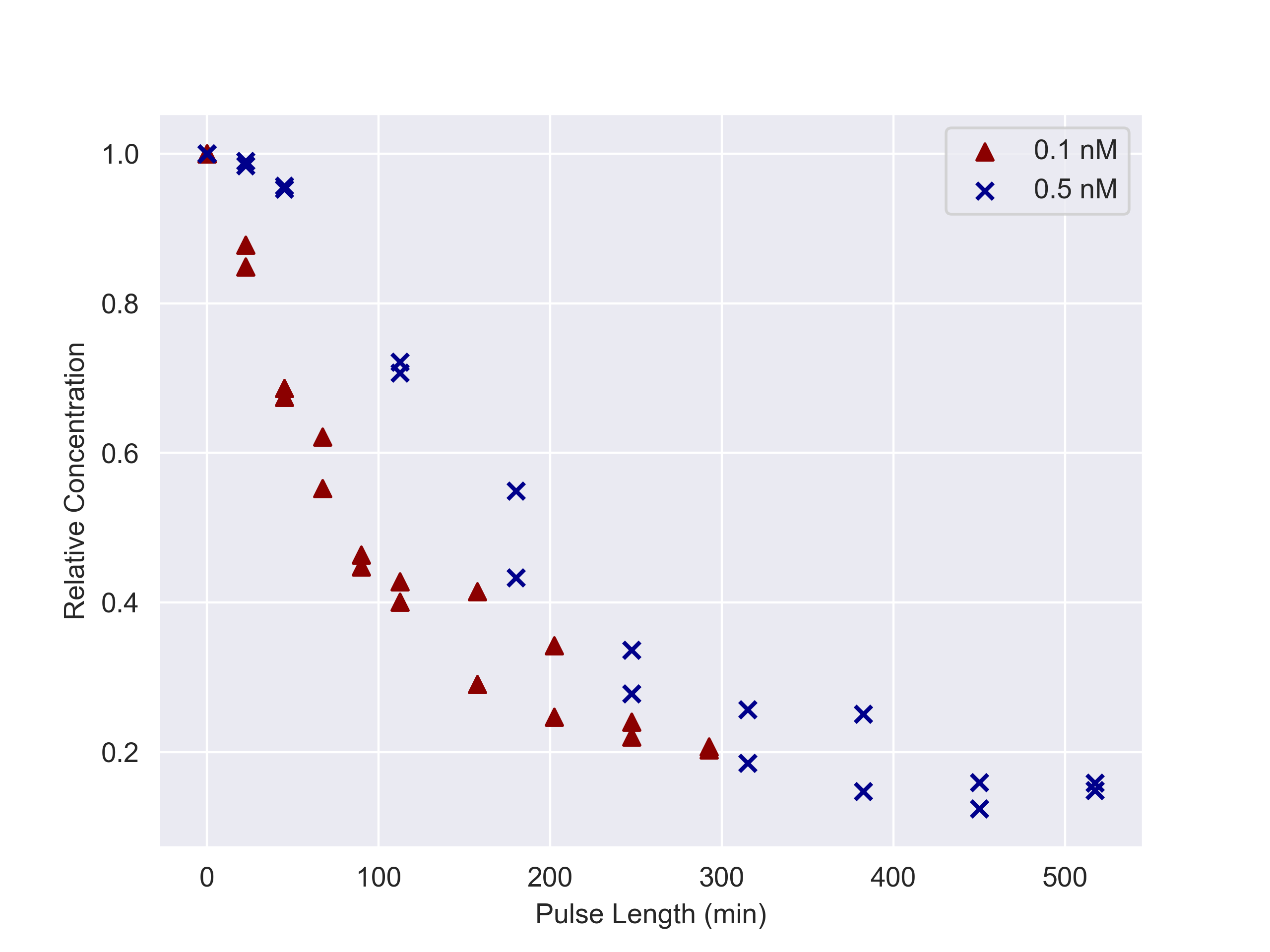

Supplement: Supplementary file 5 — Supplementary Dataset 2 [file 41467_2022_31306_MOESM5_ESM.zip › Individual Simulations Pulse Decoder/21.png]

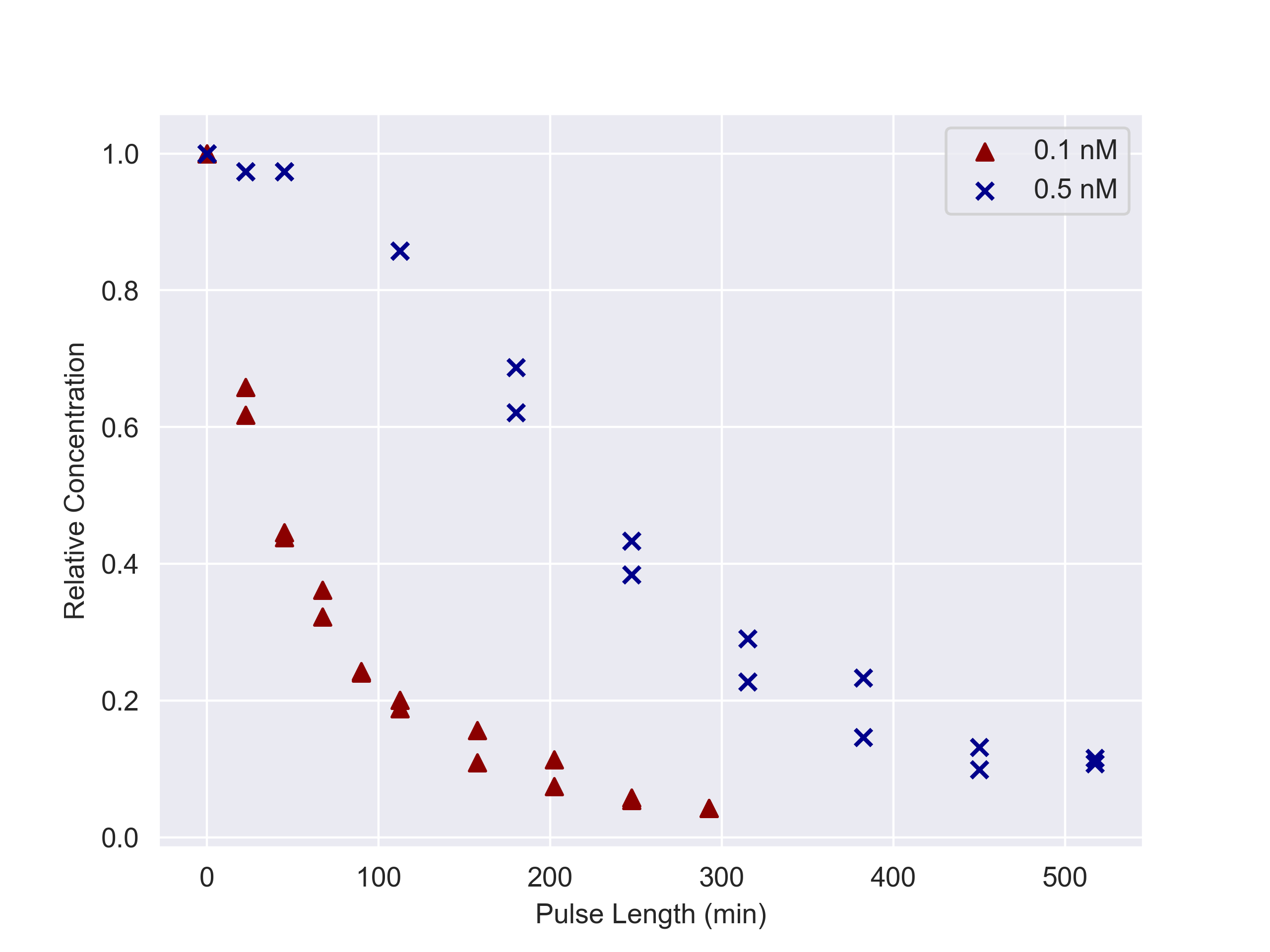

Supplement: Supplementary file 5 — Supplementary Dataset 2 [file 41467_2022_31306_MOESM5_ESM.zip › Individual Simulations Pulse Decoder/22.png]

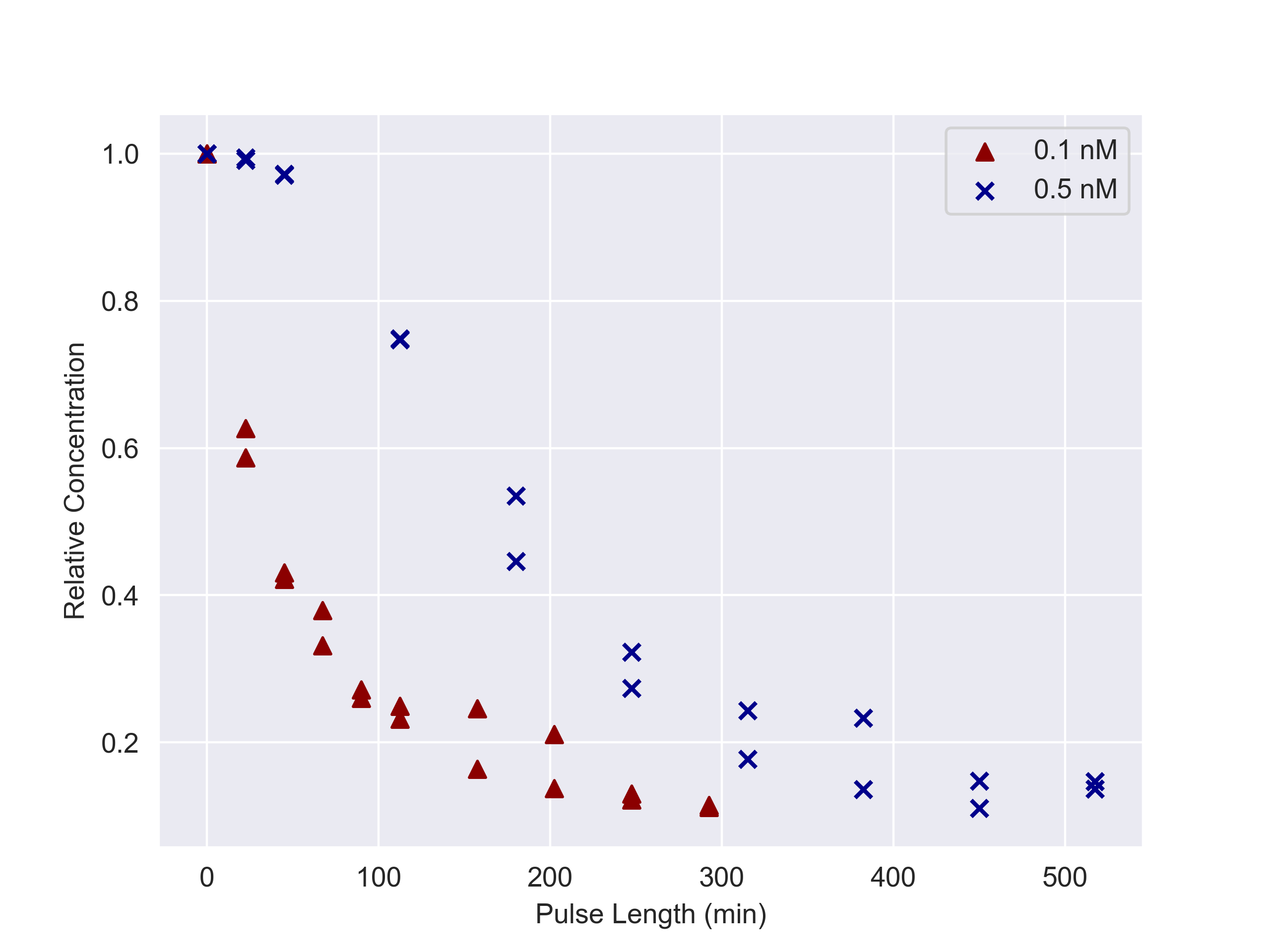

Supplement: Supplementary file 5 — Supplementary Dataset 2 [file 41467_2022_31306_MOESM5_ESM.zip › Individual Simulations Pulse Decoder/23.png]

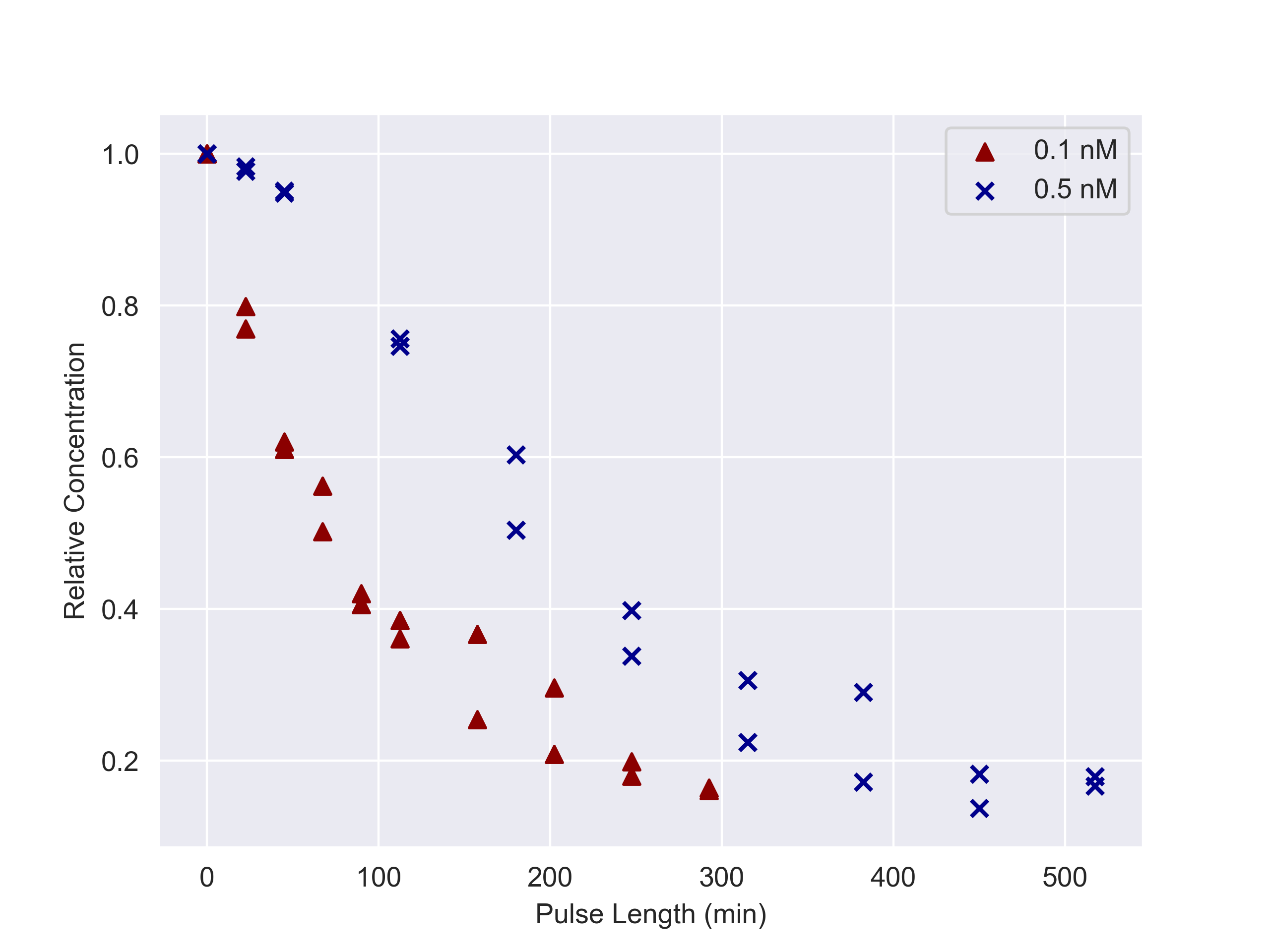

Supplement: Supplementary file 5 — Supplementary Dataset 2 [file 41467_2022_31306_MOESM5_ESM.zip › Individual Simulations Pulse Decoder/24.png]

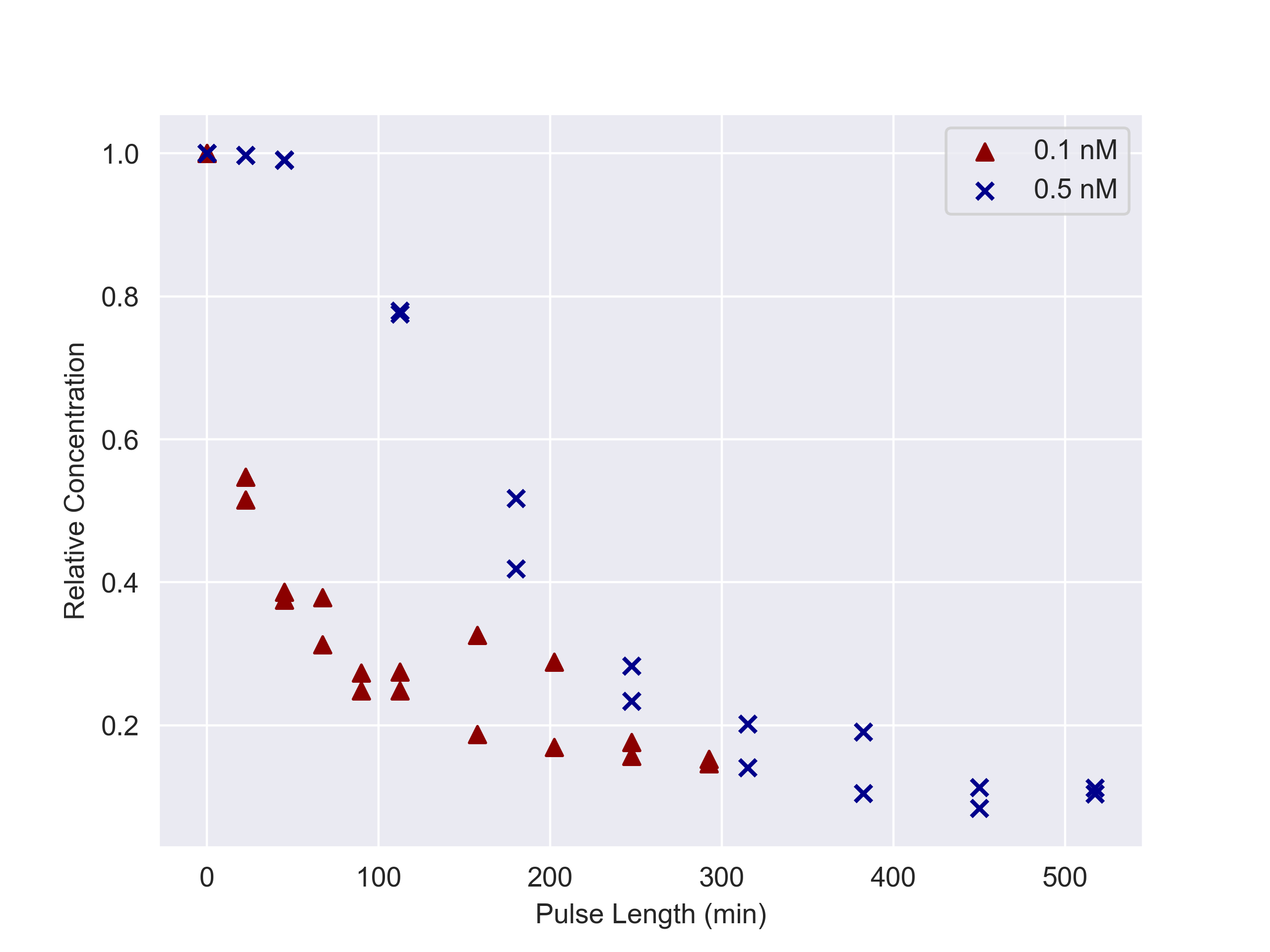

Supplement: Supplementary file 5 — Supplementary Dataset 2 [file 41467_2022_31306_MOESM5_ESM.zip › Individual Simulations Pulse Decoder/25.png]

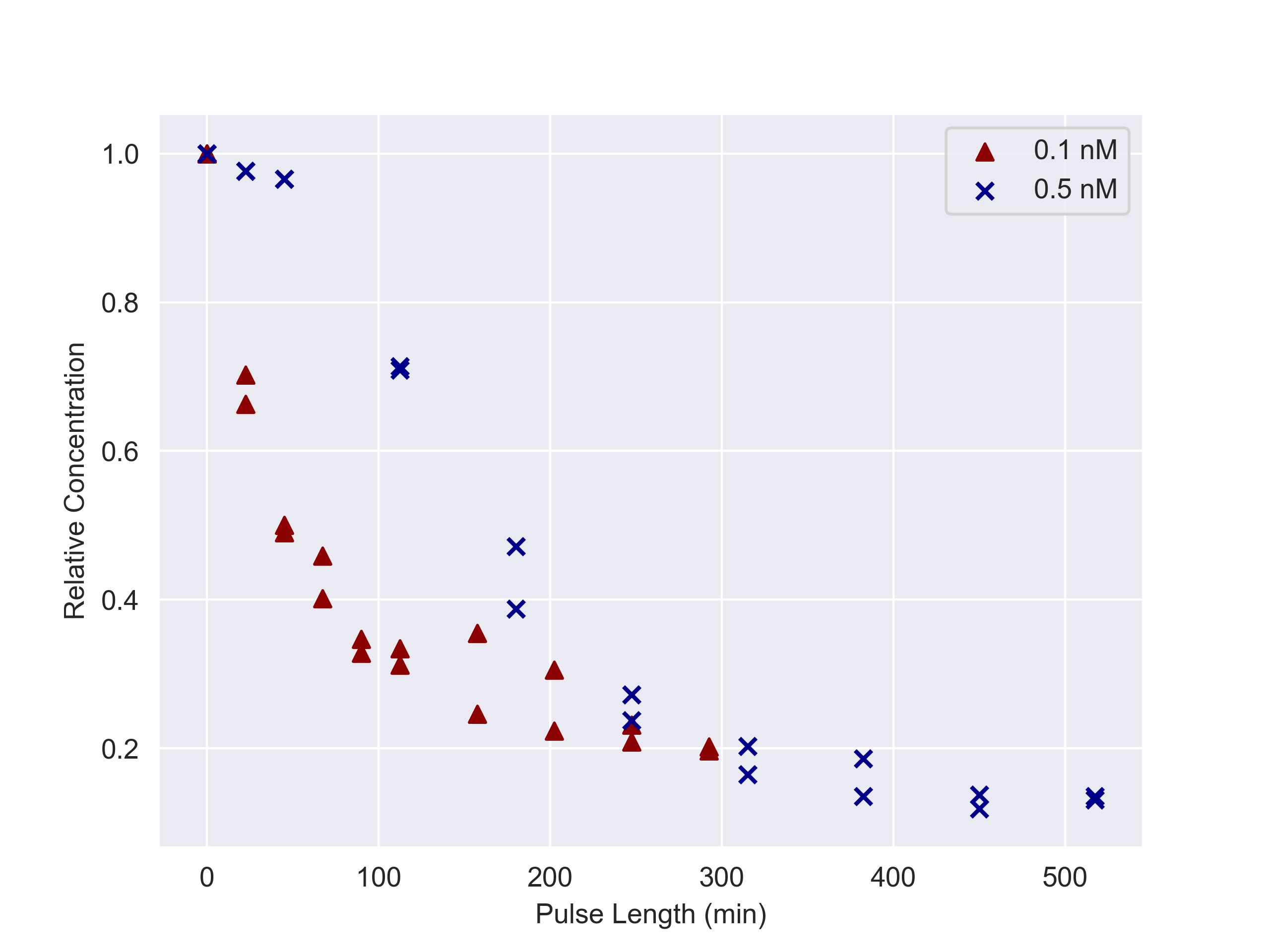

Supplement: Supplementary file 5 — Supplementary Dataset 2 [file 41467_2022_31306_MOESM5_ESM.zip › Individual Simulations Pulse Decoder/26.png]

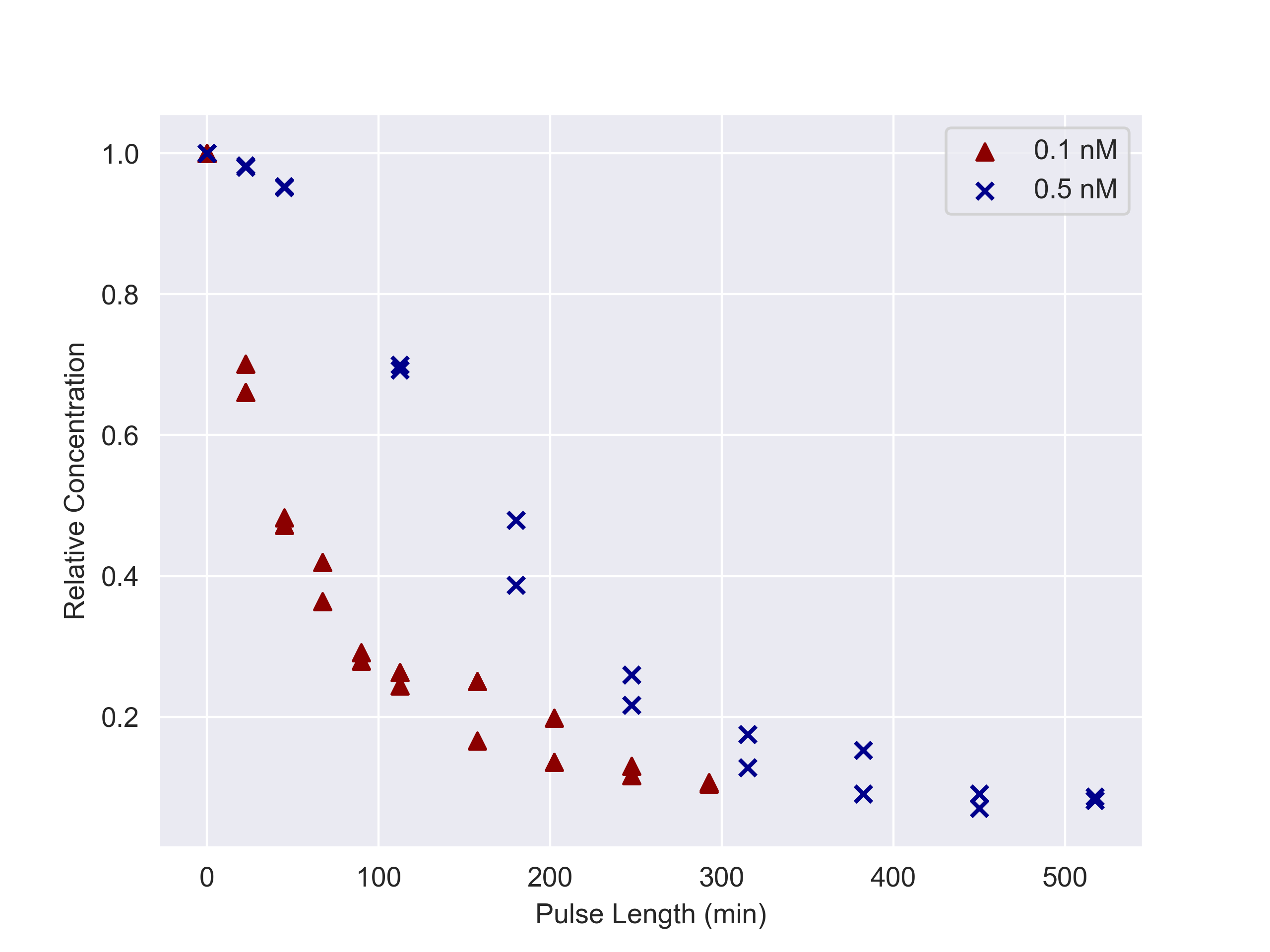

Supplement: Supplementary file 5 — Supplementary Dataset 2 [file 41467_2022_31306_MOESM5_ESM.zip › Individual Simulations Pulse Decoder/27.png]

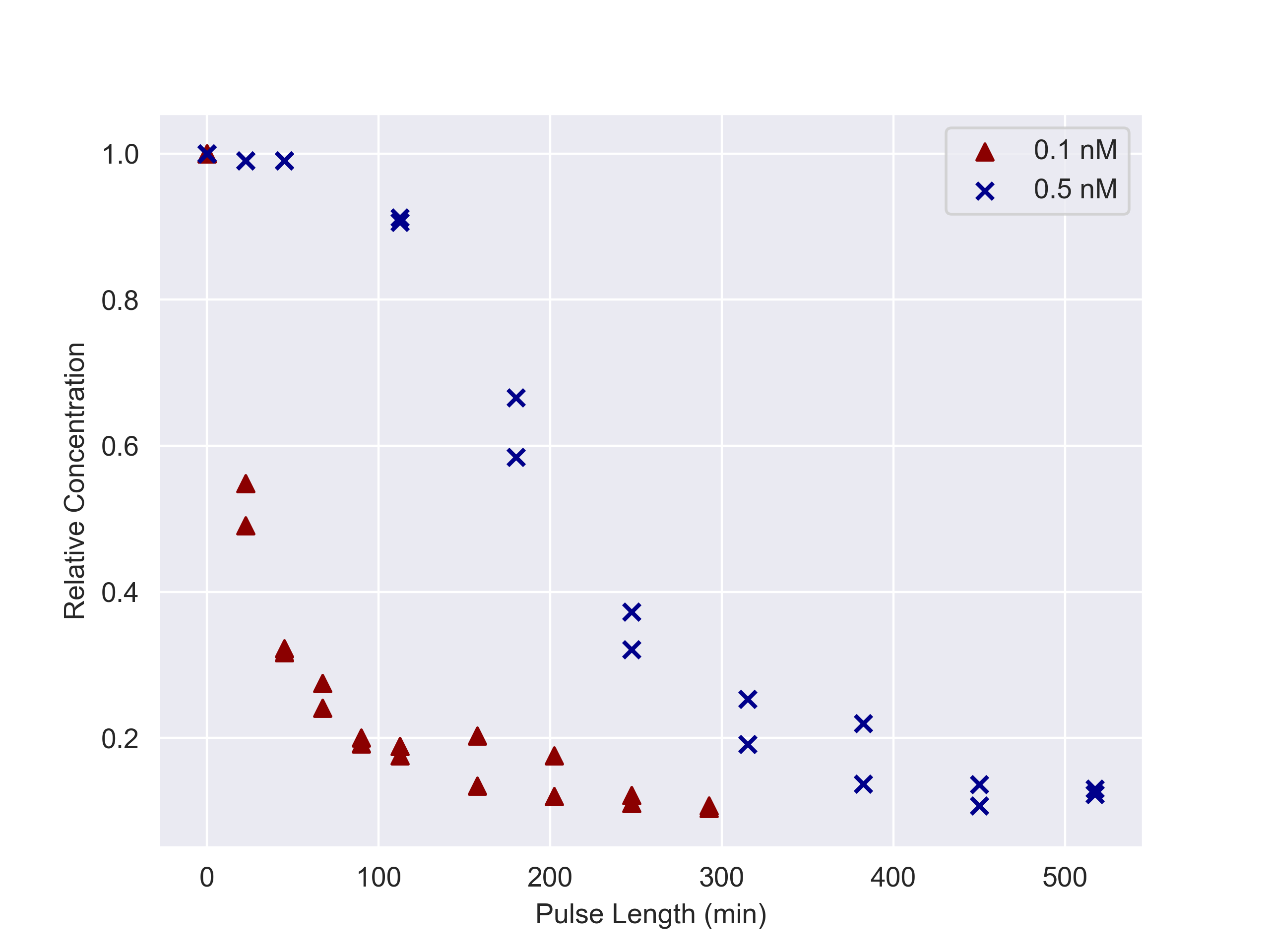

Supplement: Supplementary file 5 — Supplementary Dataset 2 [file 41467_2022_31306_MOESM5_ESM.zip › Individual Simulations Pulse Decoder/28.png]

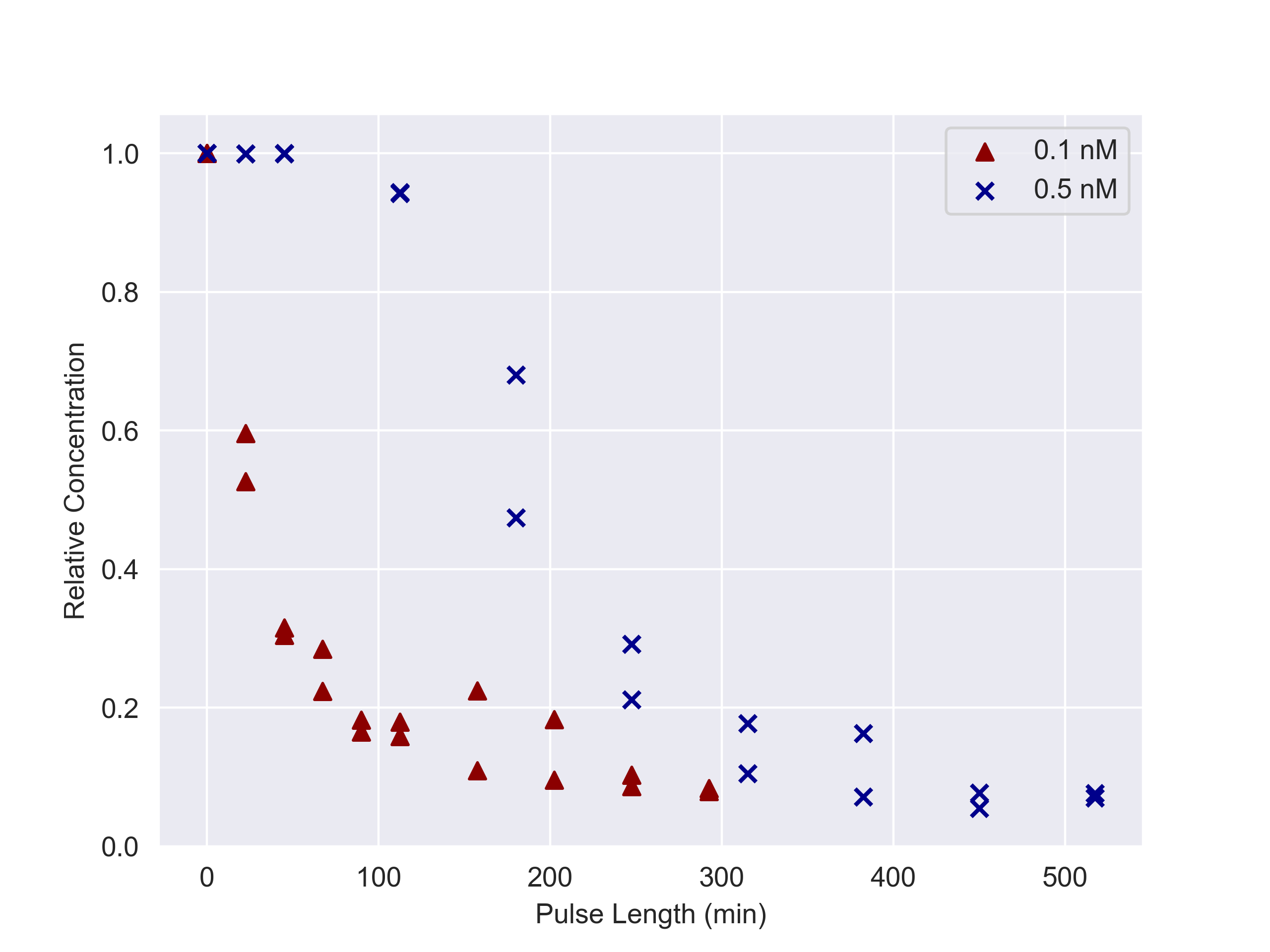

Supplement: Supplementary file 5 — Supplementary Dataset 2 [file 41467_2022_31306_MOESM5_ESM.zip › Individual Simulations Pulse Decoder/29.png]

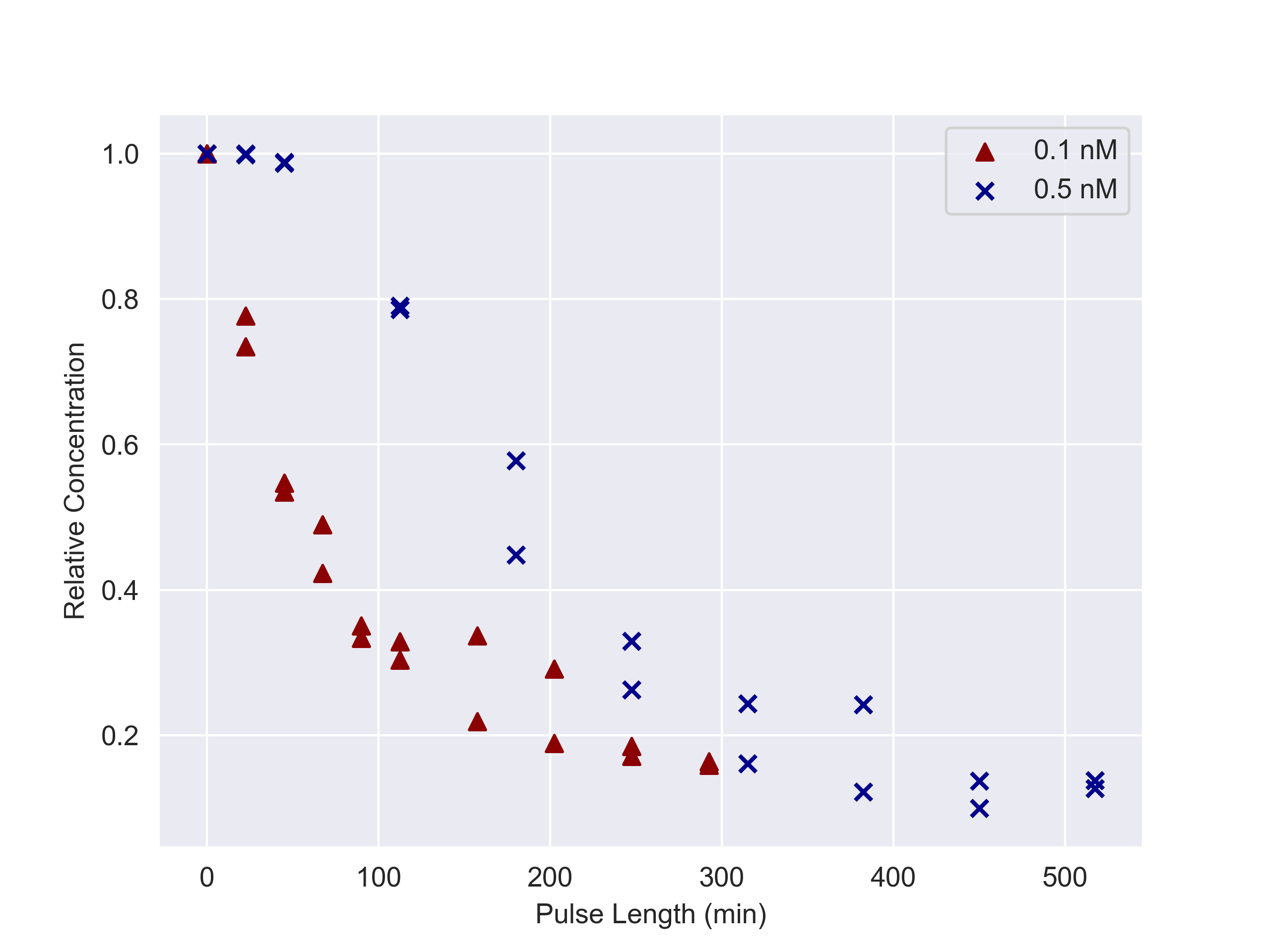

Supplement: Supplementary file 5 — Supplementary Dataset 2 [file 41467_2022_31306_MOESM5_ESM.zip › Individual Simulations Pulse Decoder/3.png]

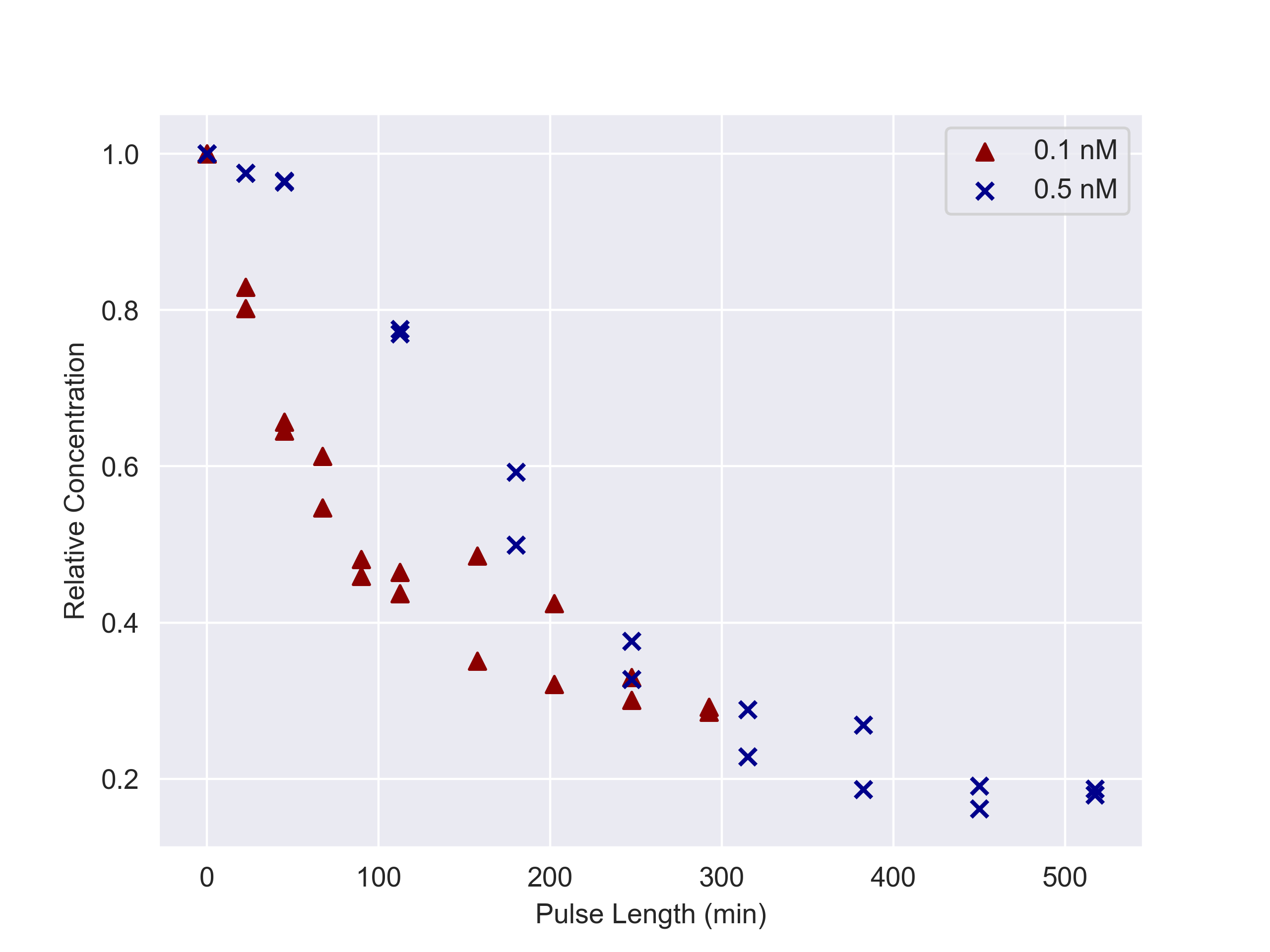

Supplement: Supplementary file 5 — Supplementary Dataset 2 [file 41467_2022_31306_MOESM5_ESM.zip › Individual Simulations Pulse Decoder/30.png]

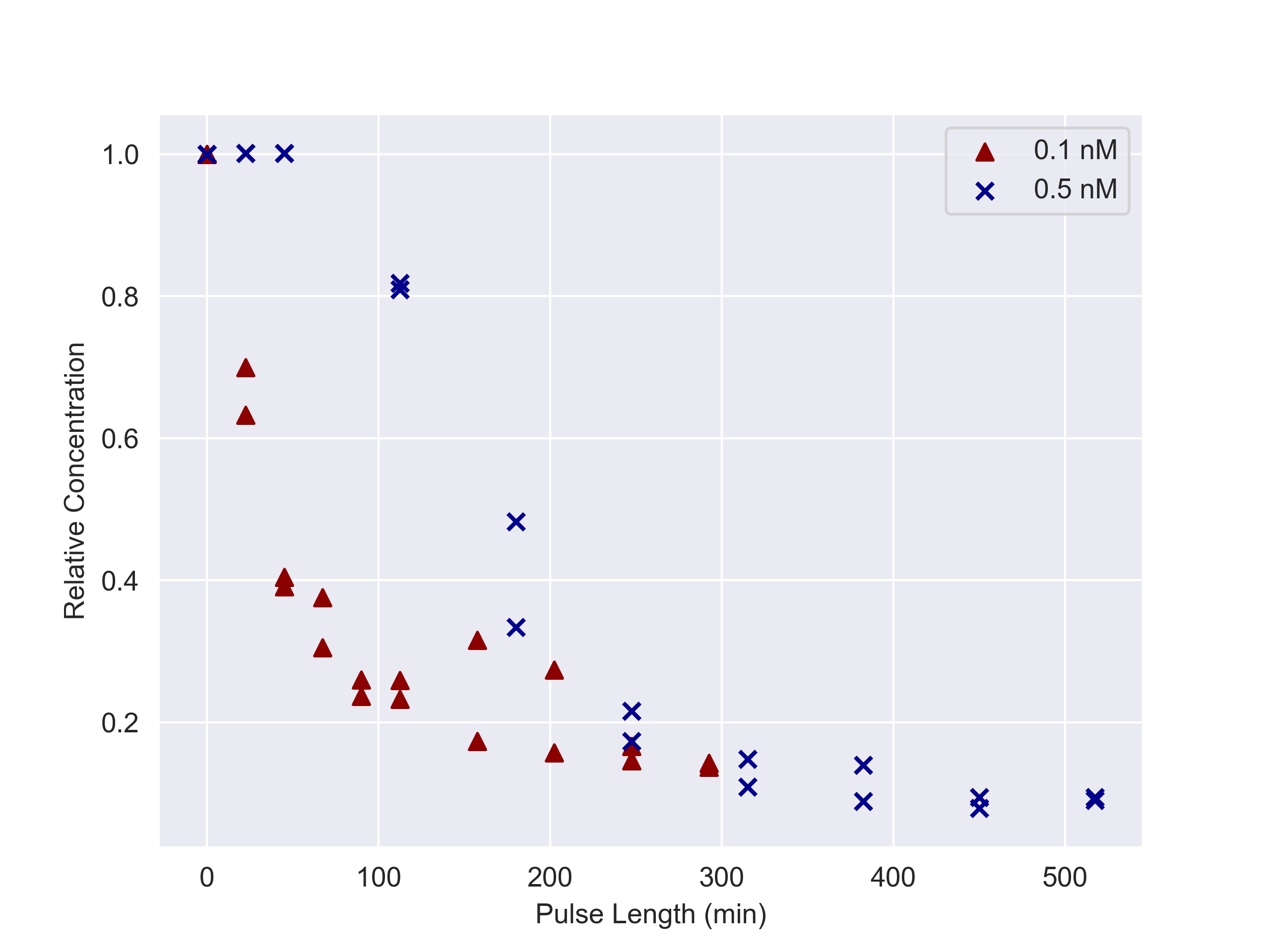

Supplement: Supplementary file 5 — Supplementary Dataset 2 [file 41467_2022_31306_MOESM5_ESM.zip › Individual Simulations Pulse Decoder/31.png]

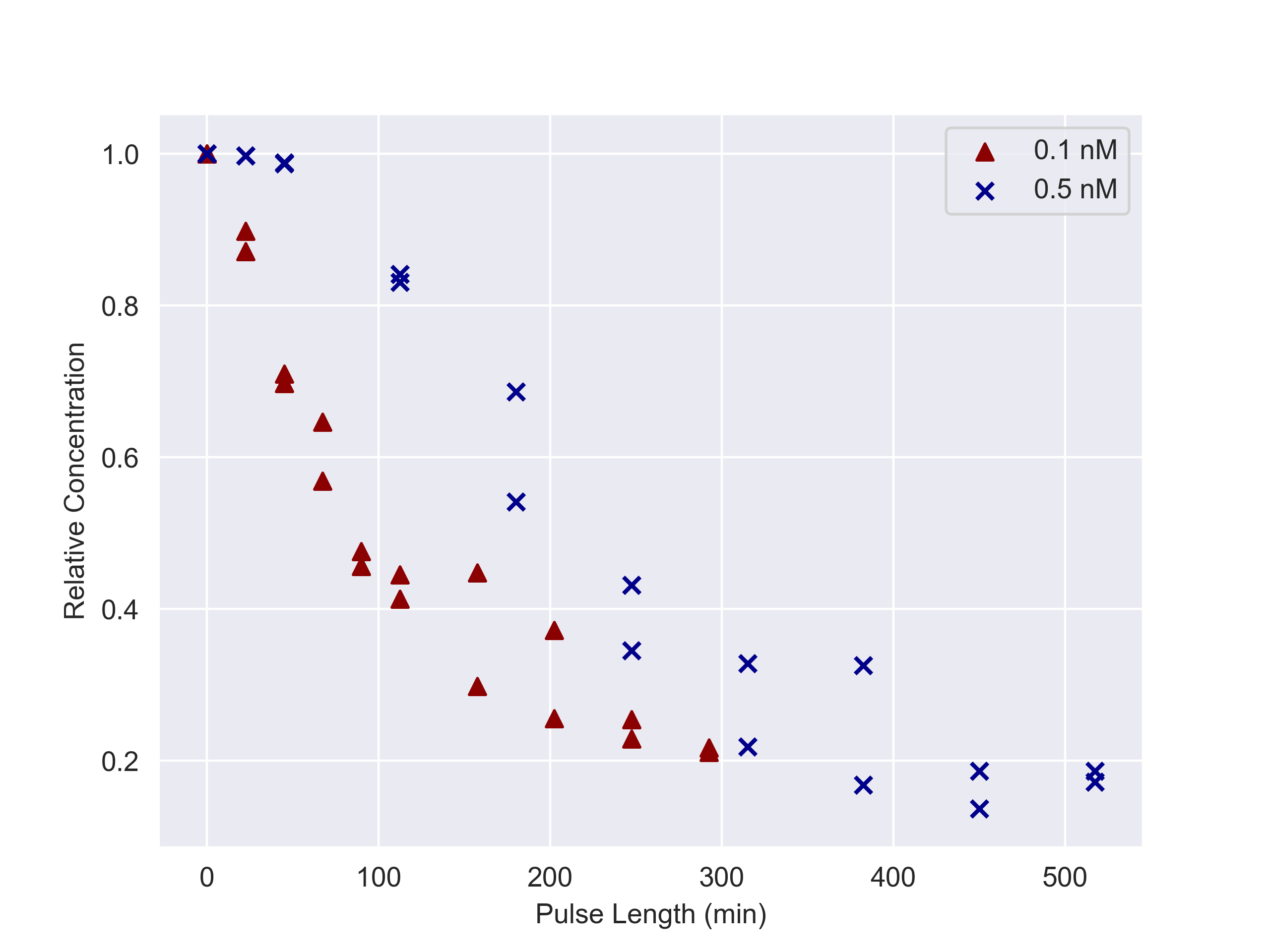

Supplement: Supplementary file 5 — Supplementary Dataset 2 [file 41467_2022_31306_MOESM5_ESM.zip › Individual Simulations Pulse Decoder/32.png]

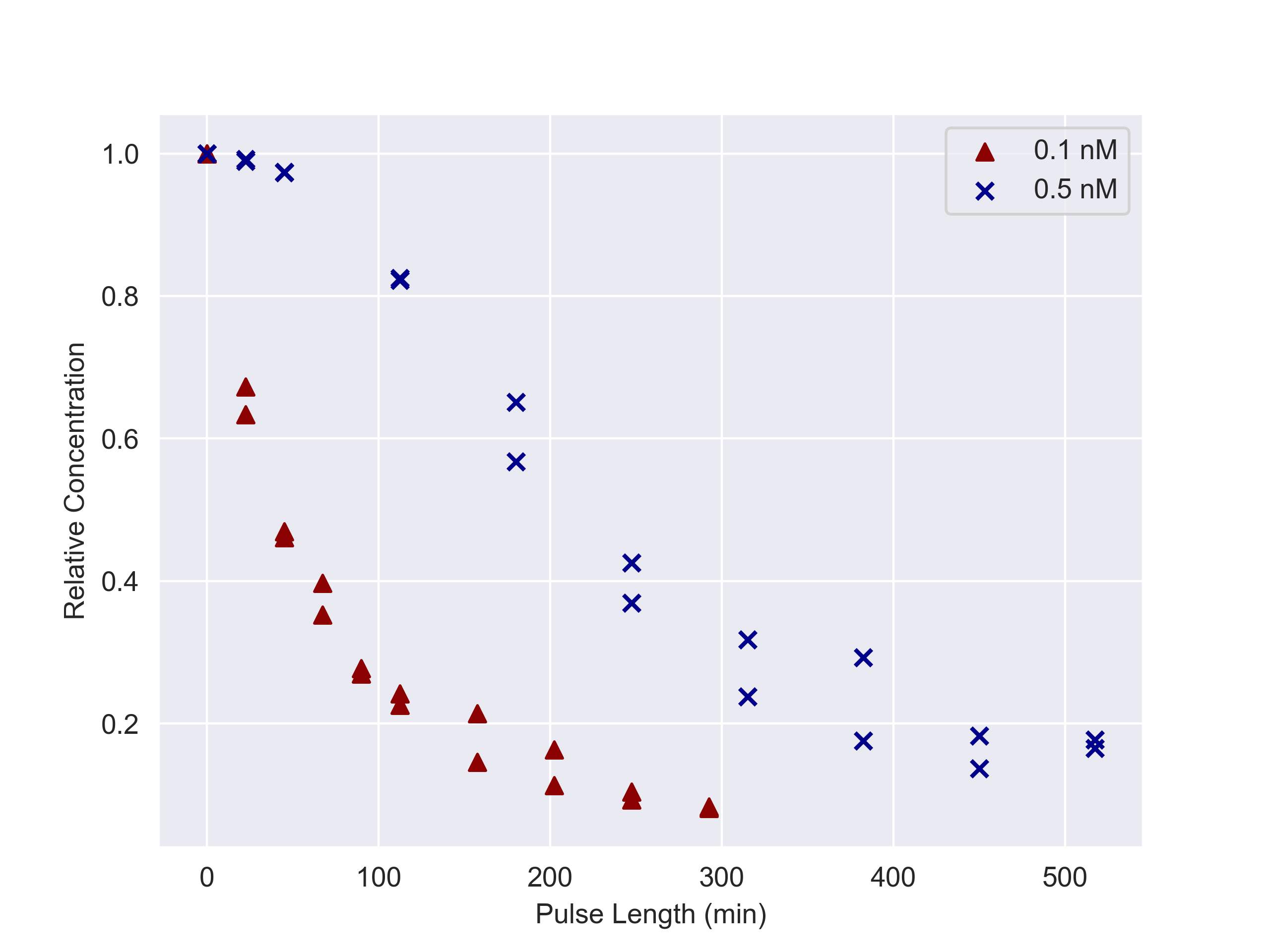

Supplement: Supplementary file 5 — Supplementary Dataset 2 [file 41467_2022_31306_MOESM5_ESM.zip › Individual Simulations Pulse Decoder/33.png]

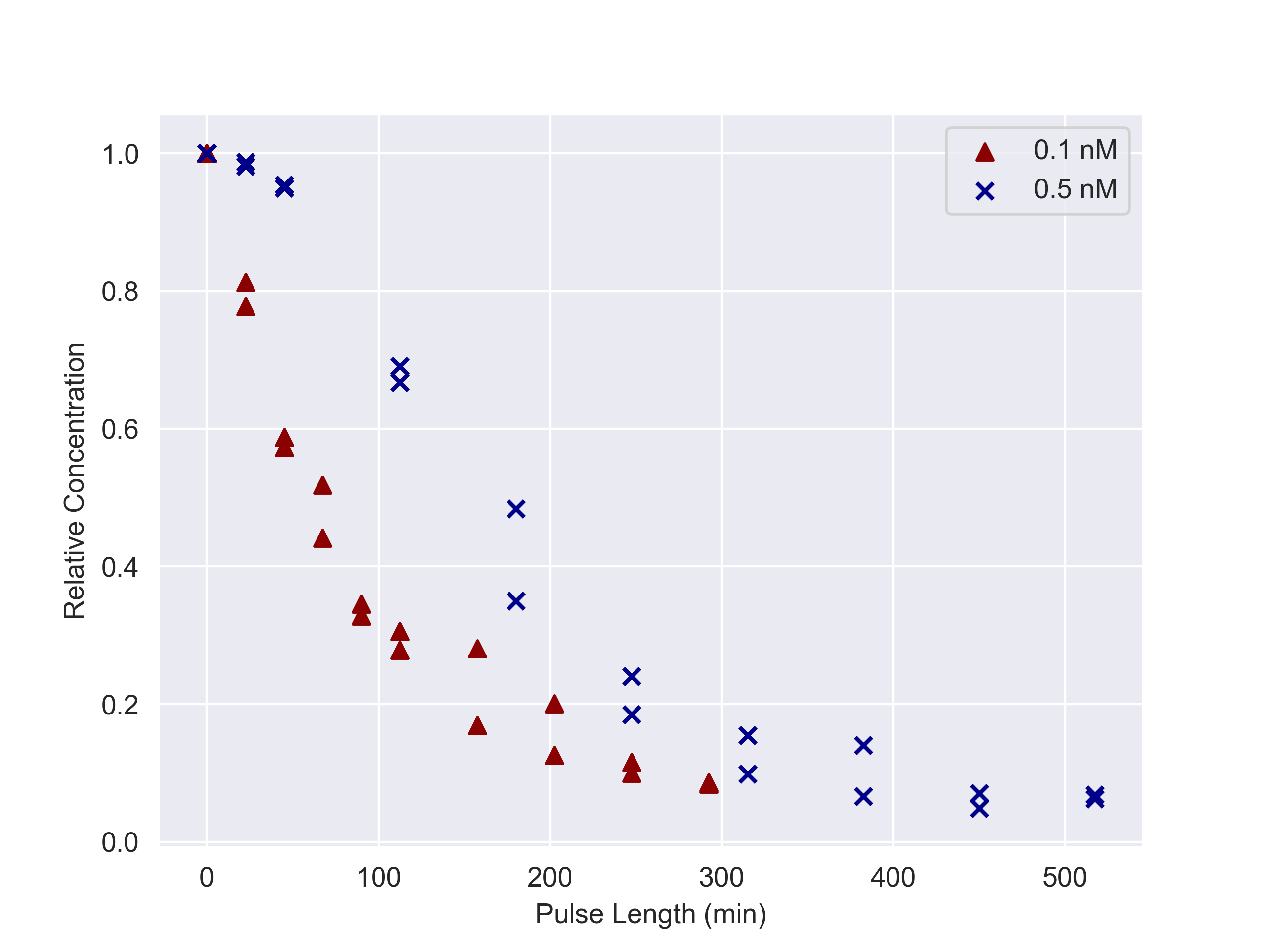

Supplement: Supplementary file 5 — Supplementary Dataset 2 [file 41467_2022_31306_MOESM5_ESM.zip › Individual Simulations Pulse Decoder/34.png]

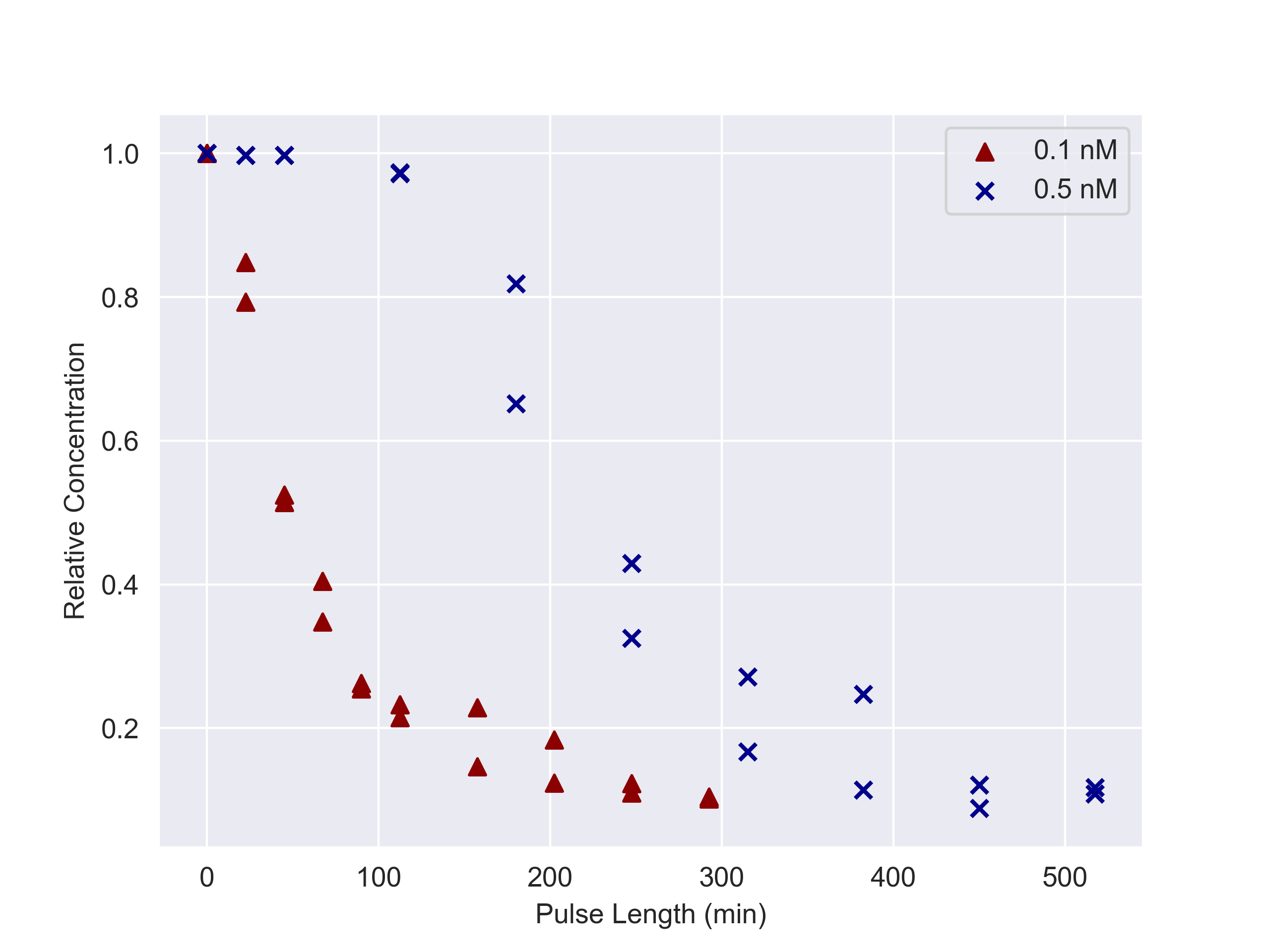

Supplement: Supplementary file 5 — Supplementary Dataset 2 [file 41467_2022_31306_MOESM5_ESM.zip › Individual Simulations Pulse Decoder/35.png]

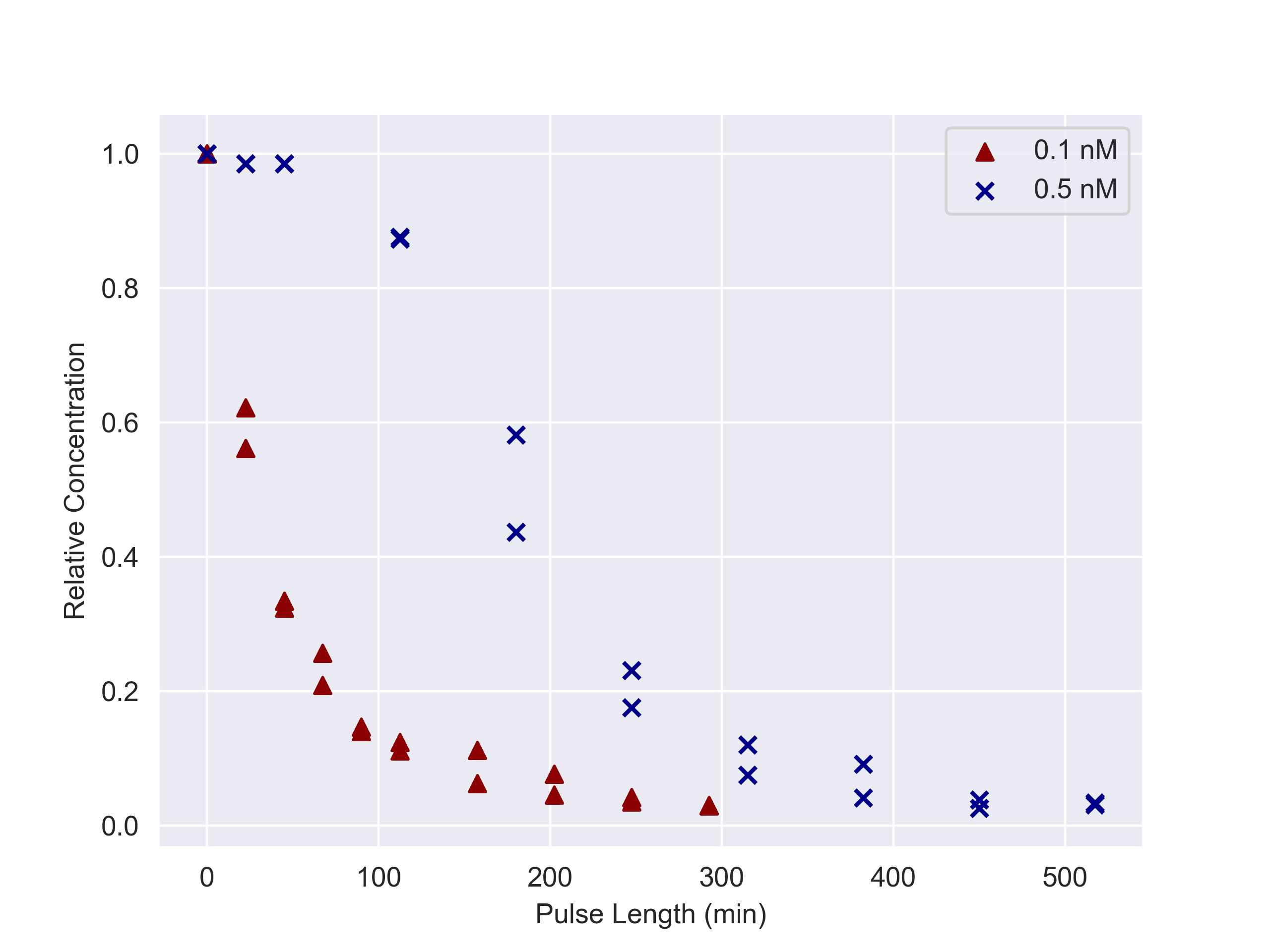

Supplement: Supplementary file 5 — Supplementary Dataset 2 [file 41467_2022_31306_MOESM5_ESM.zip › Individual Simulations Pulse Decoder/36.png]

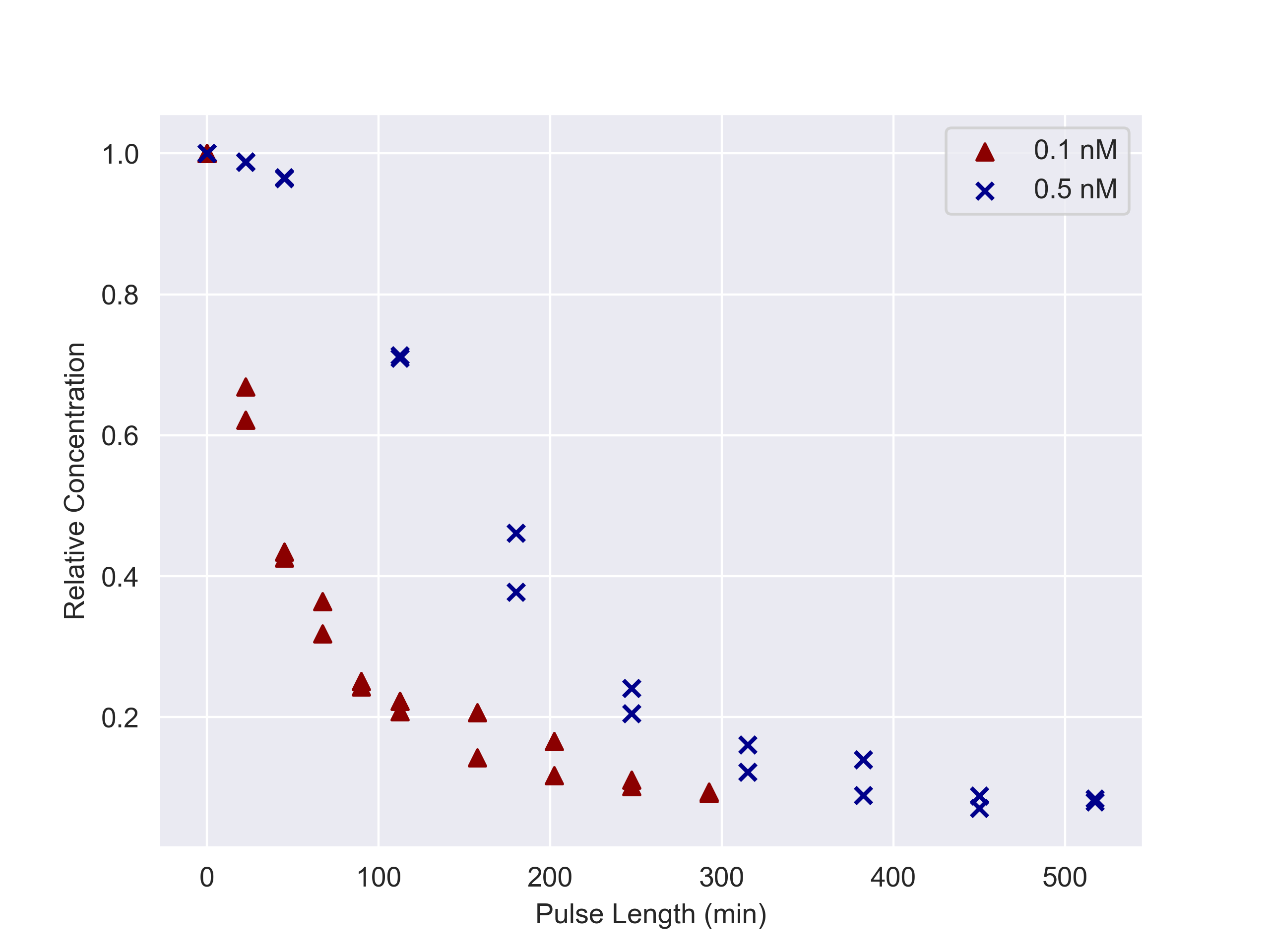

Supplement: Supplementary file 5 — Supplementary Dataset 2 [file 41467_2022_31306_MOESM5_ESM.zip › Individual Simulations Pulse Decoder/37.png]

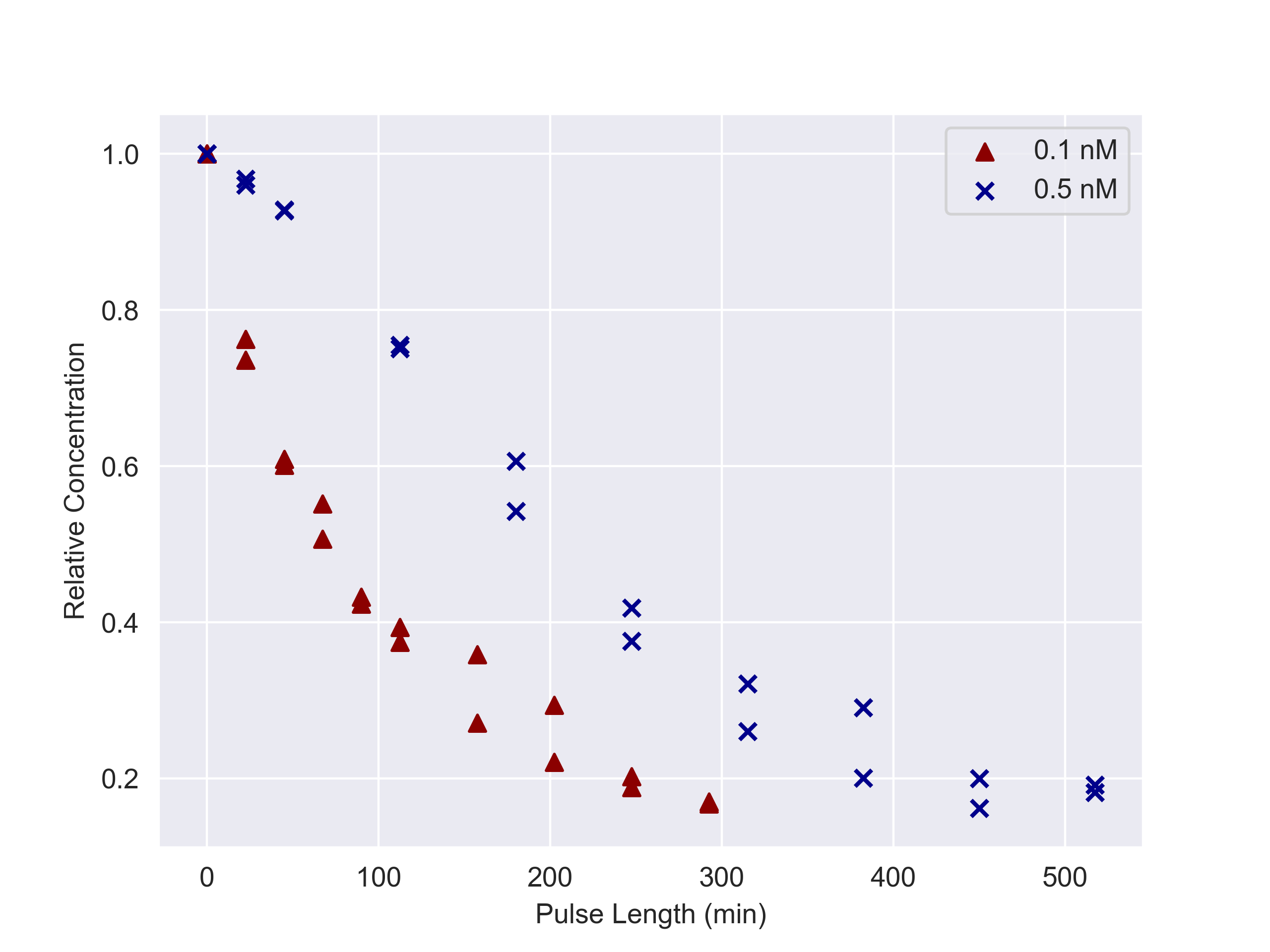

Supplement: Supplementary file 5 — Supplementary Dataset 2 [file 41467_2022_31306_MOESM5_ESM.zip › Individual Simulations Pulse Decoder/38.png]

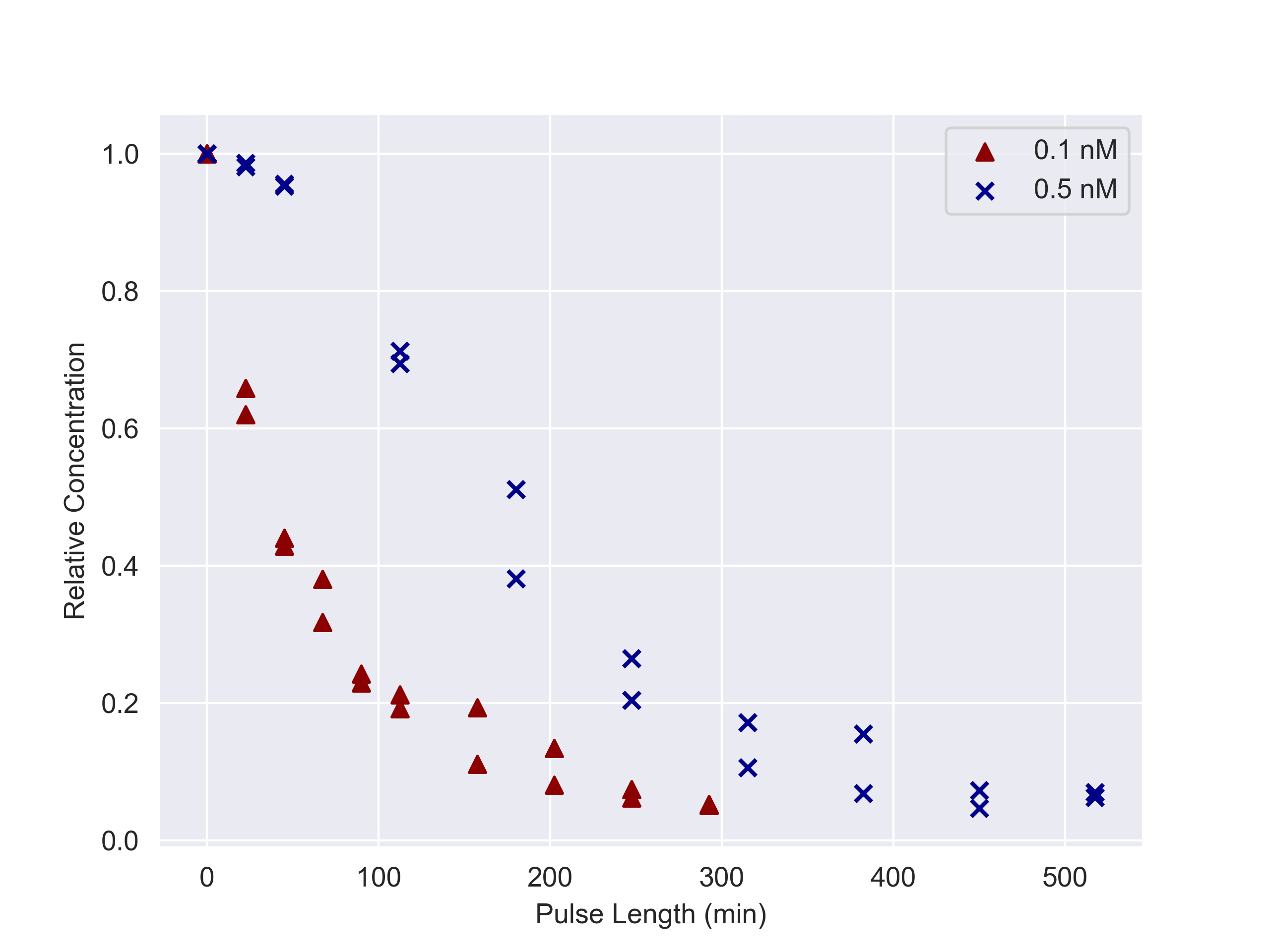

Supplement: Supplementary file 5 — Supplementary Dataset 2 [file 41467_2022_31306_MOESM5_ESM.zip › Individual Simulations Pulse Decoder/39.png]

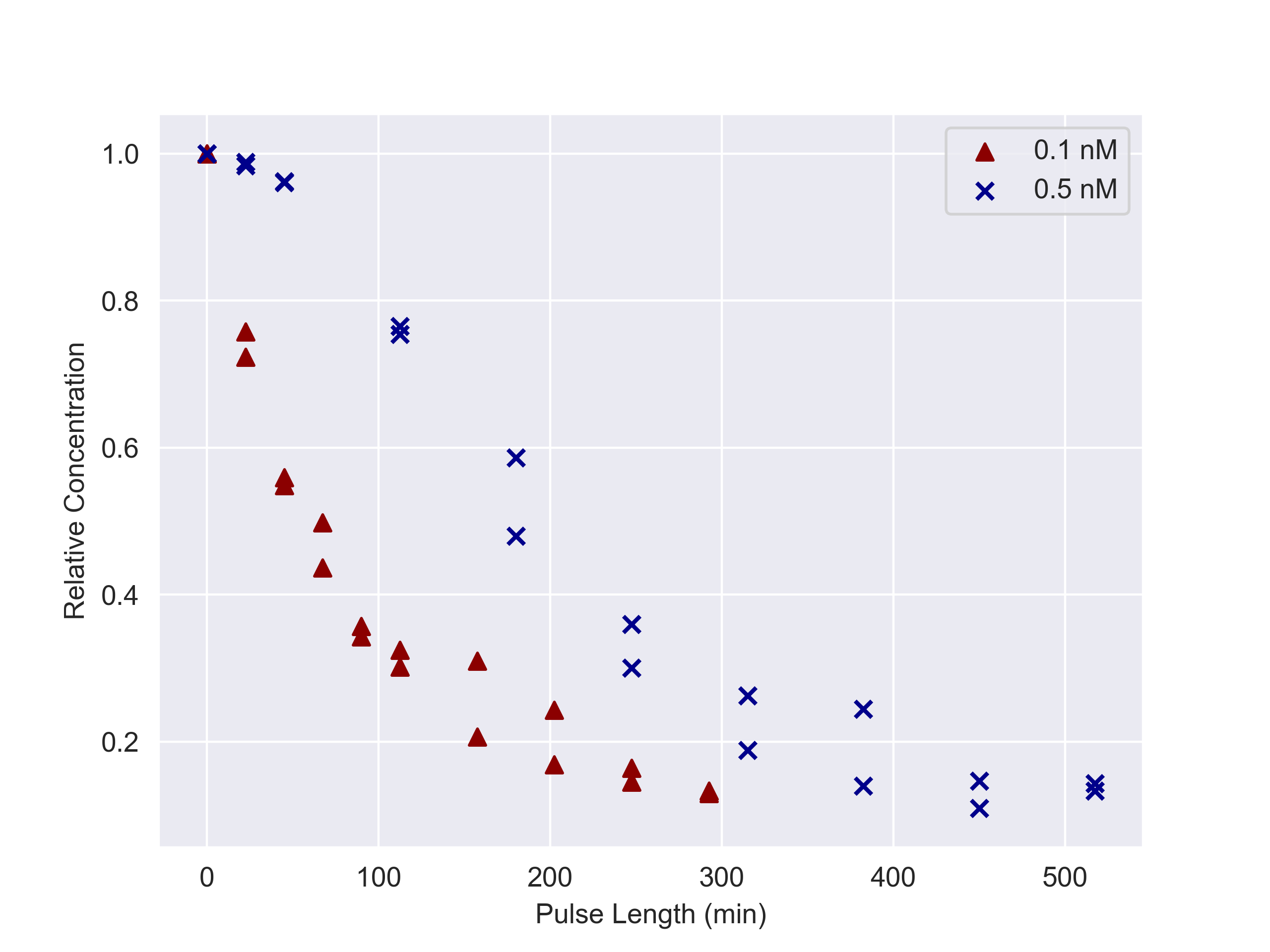

Supplement: Supplementary file 5 — Supplementary Dataset 2 [file 41467_2022_31306_MOESM5_ESM.zip › Individual Simulations Pulse Decoder/4.png]

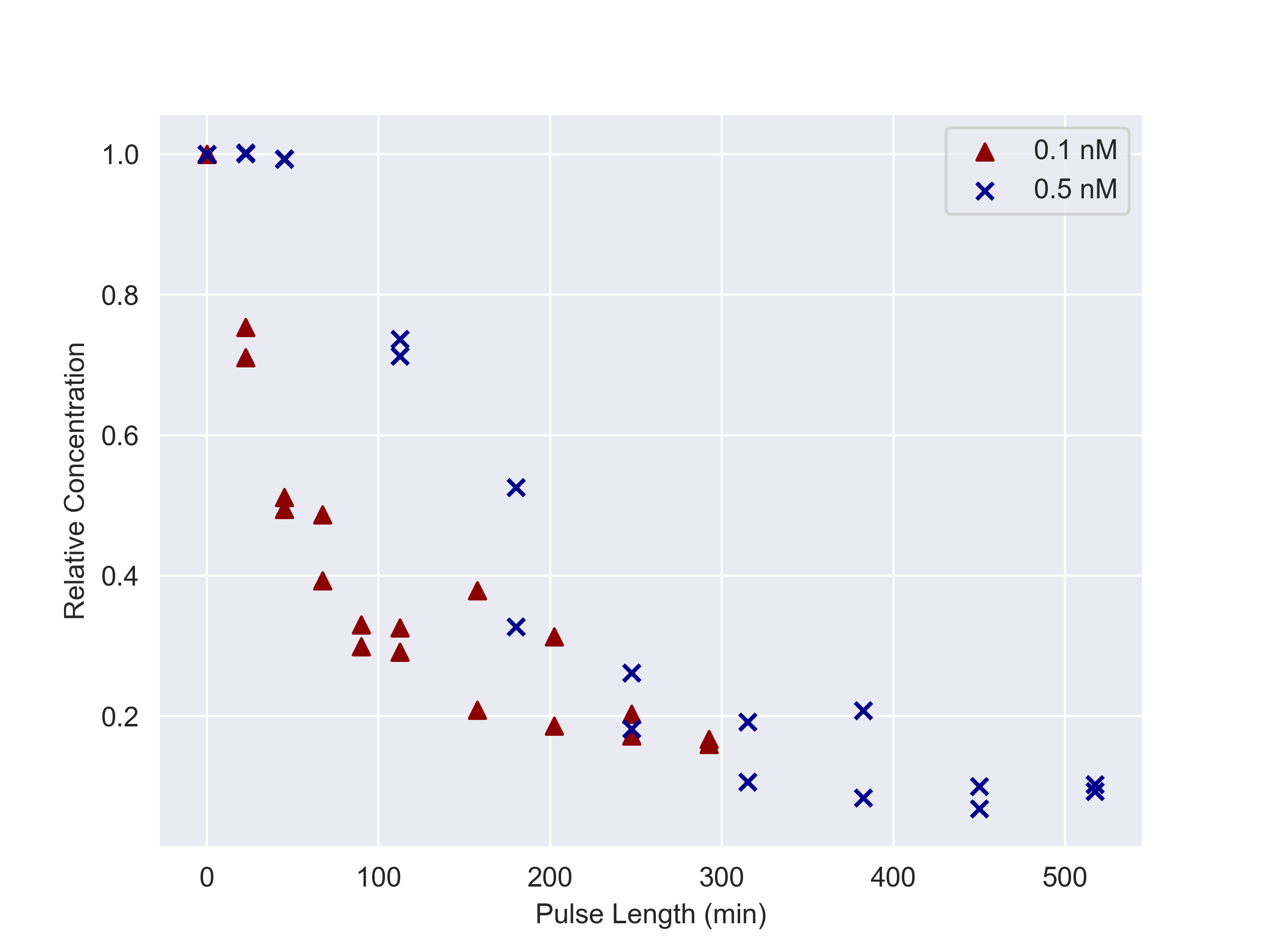

Supplement: Supplementary file 5 — Supplementary Dataset 2 [file 41467_2022_31306_MOESM5_ESM.zip › Individual Simulations Pulse Decoder/40.png]

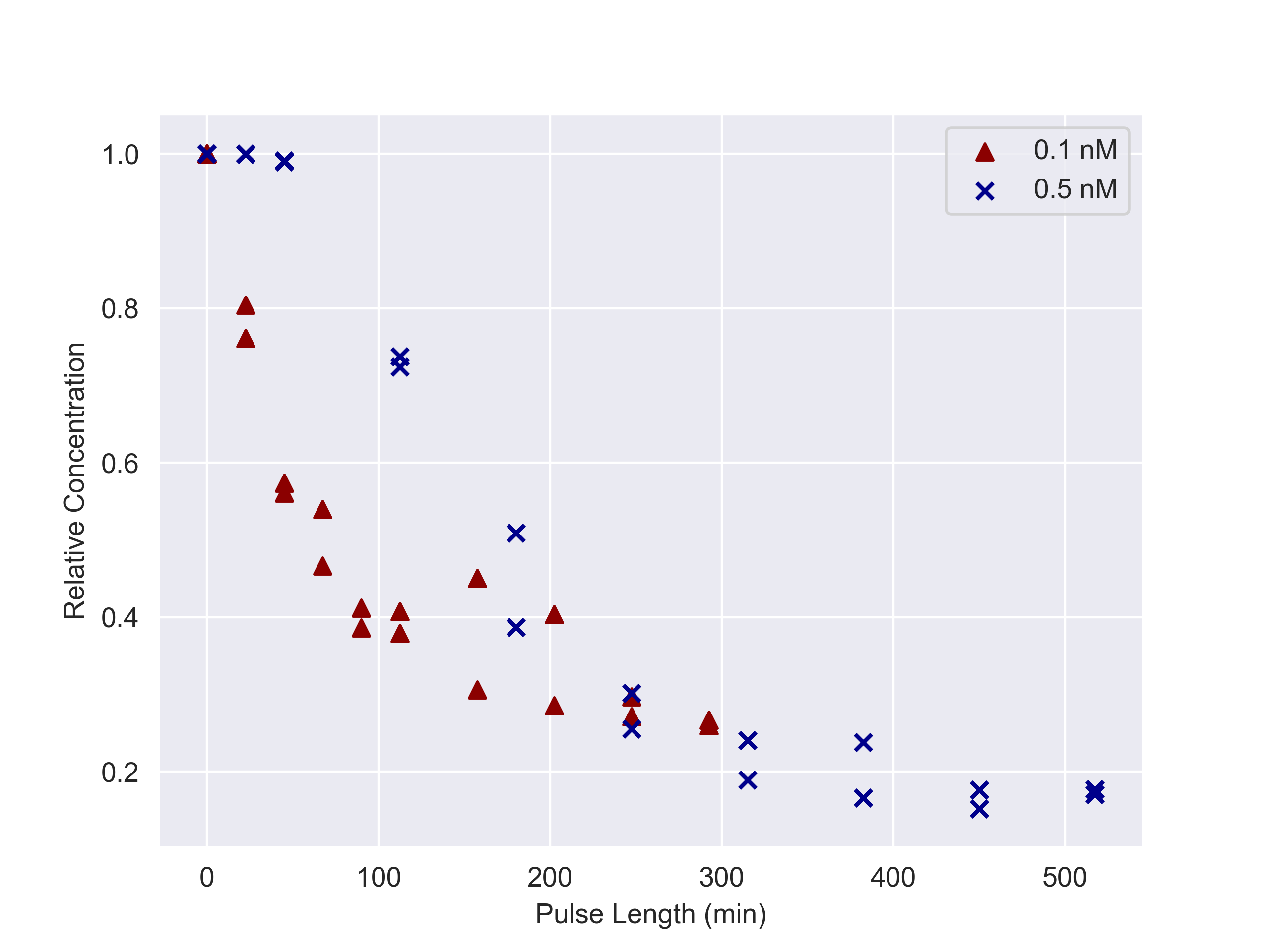

Supplement: Supplementary file 5 — Supplementary Dataset 2 [file 41467_2022_31306_MOESM5_ESM.zip › Individual Simulations Pulse Decoder/41.png]

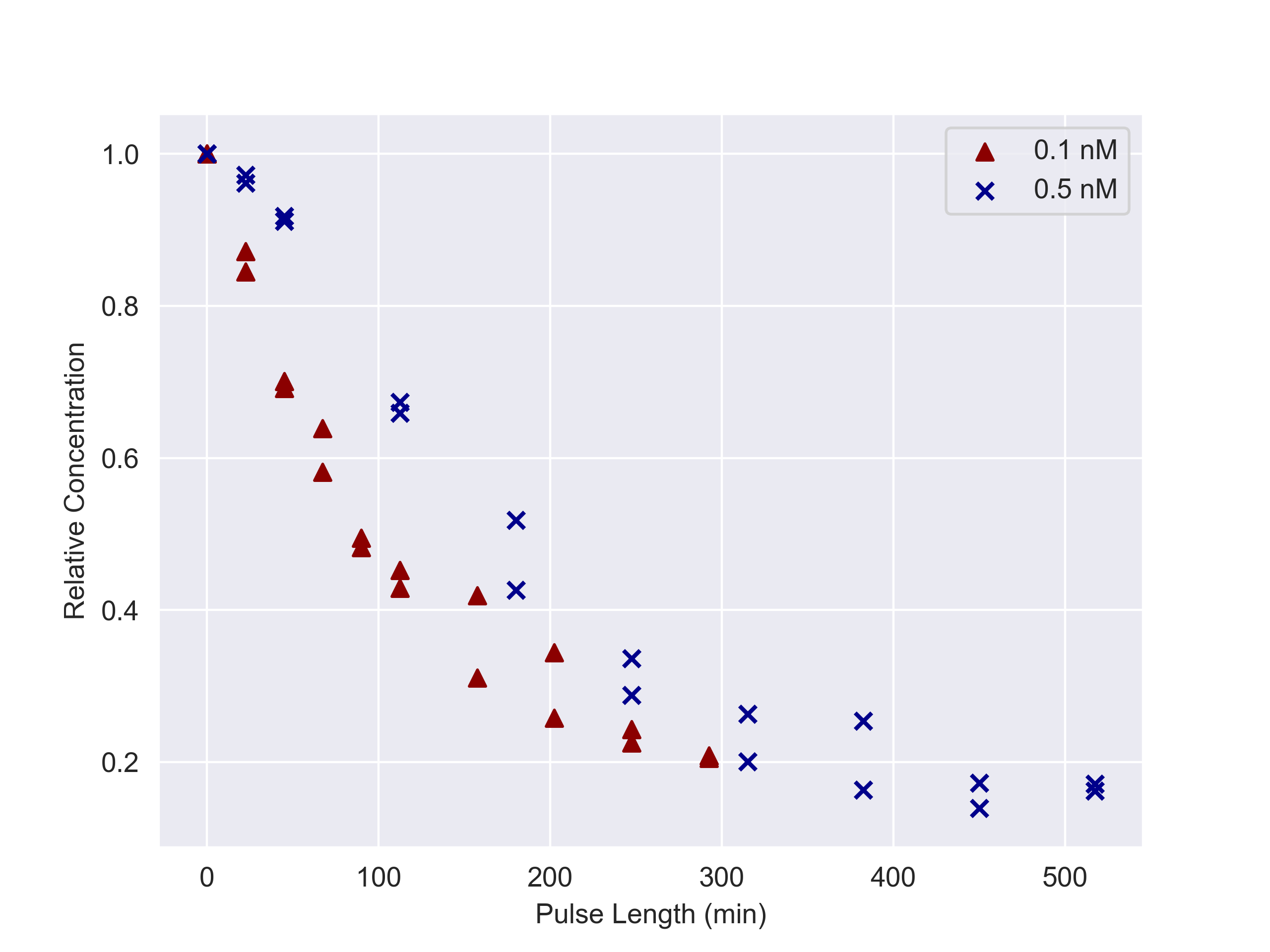

Supplement: Supplementary file 5 — Supplementary Dataset 2 [file 41467_2022_31306_MOESM5_ESM.zip › Individual Simulations Pulse Decoder/42.png]

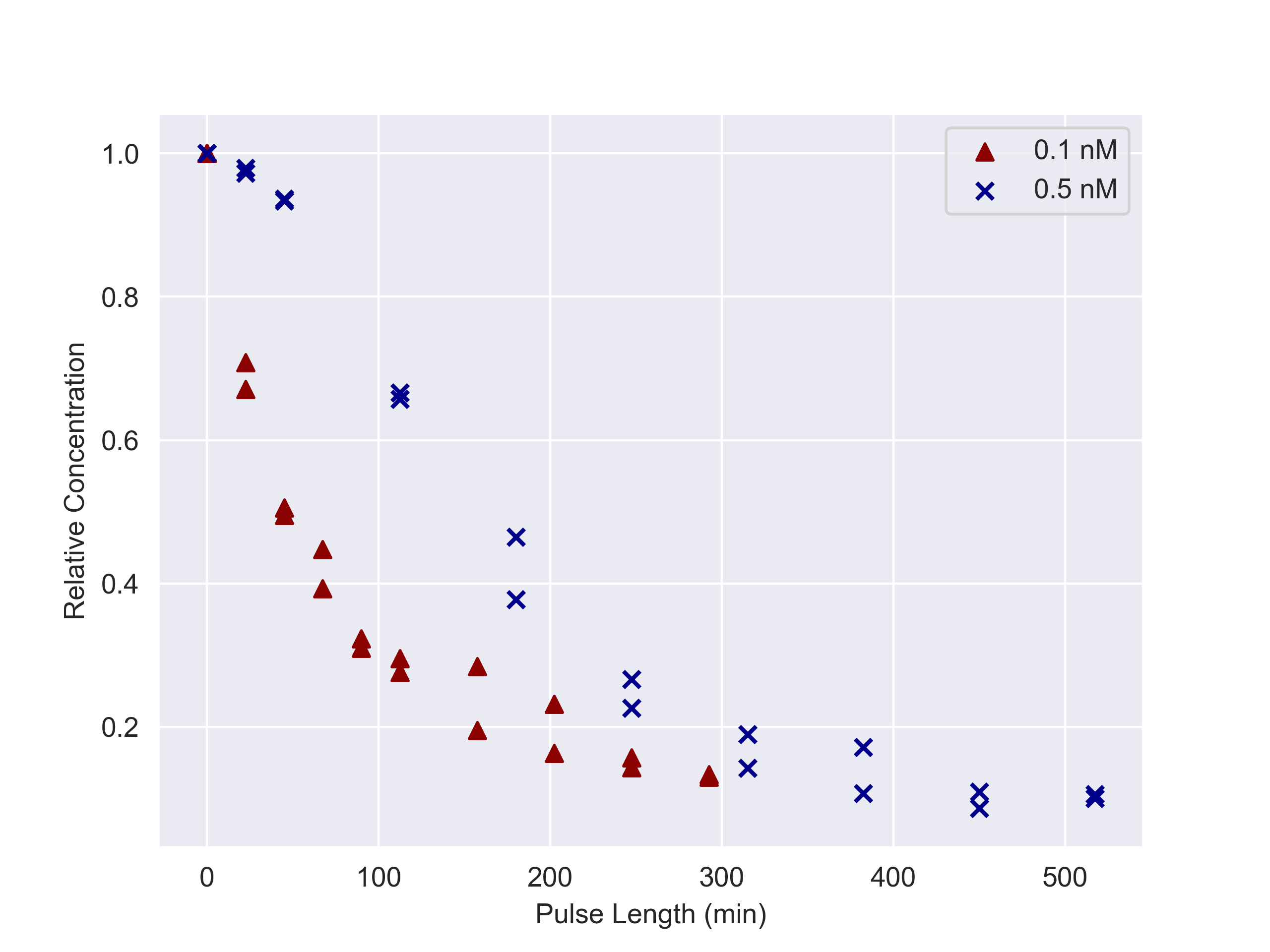

Supplement: Supplementary file 5 — Supplementary Dataset 2 [file 41467_2022_31306_MOESM5_ESM.zip › Individual Simulations Pulse Decoder/43.png]

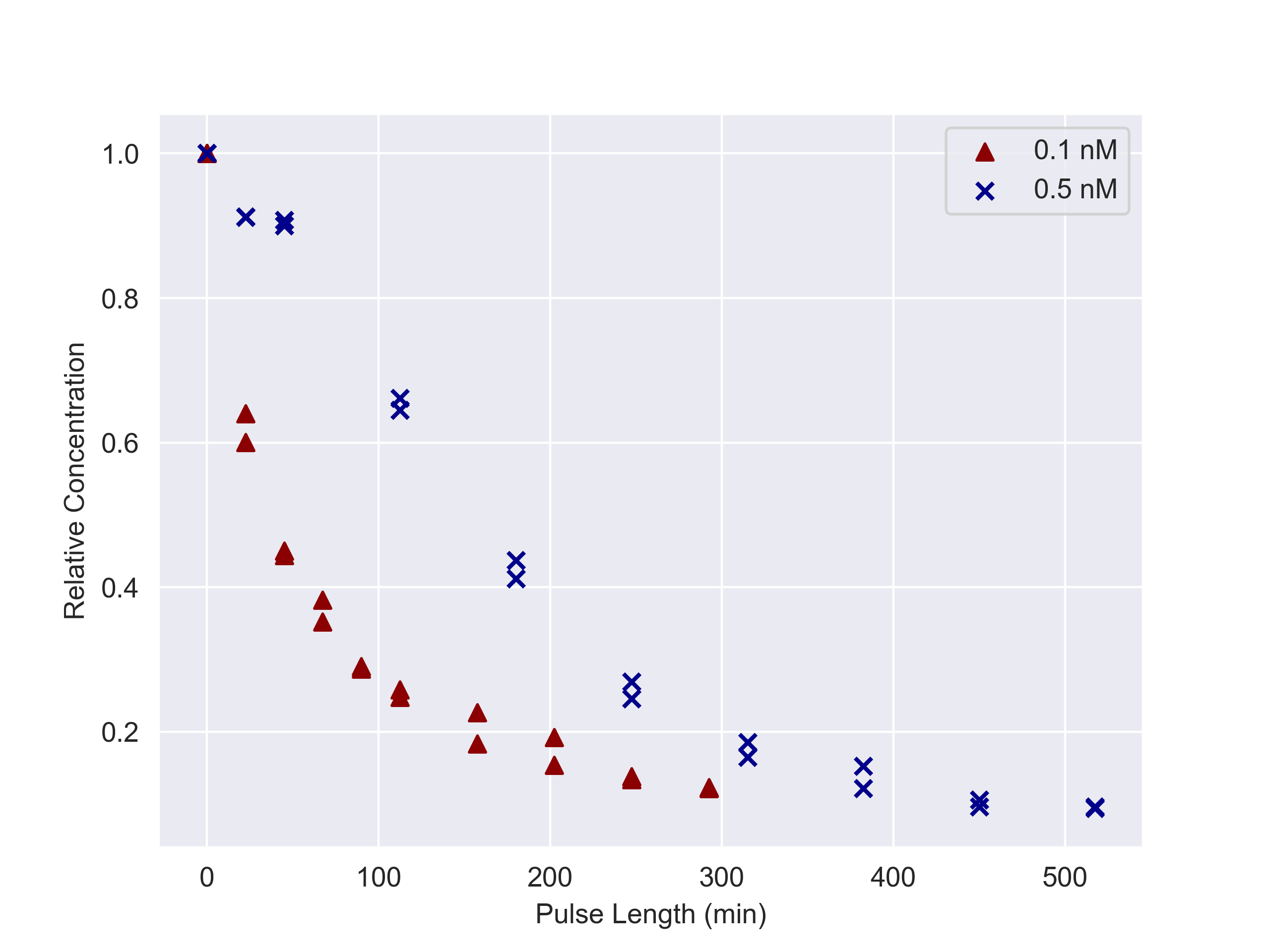

Supplement: Supplementary file 5 — Supplementary Dataset 2 [file 41467_2022_31306_MOESM5_ESM.zip › Individual Simulations Pulse Decoder/44.png]

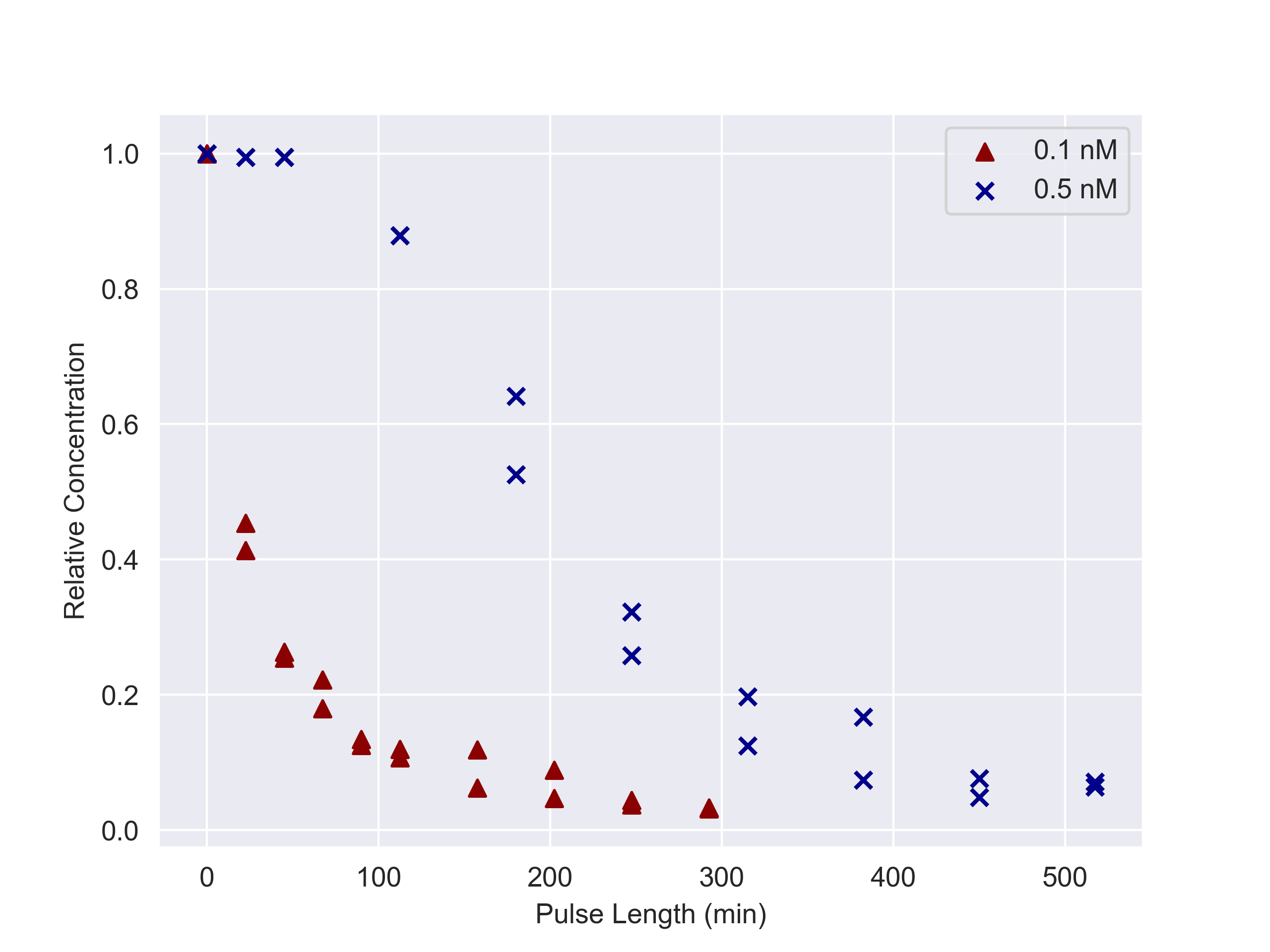

Supplement: Supplementary file 5 — Supplementary Dataset 2 [file 41467_2022_31306_MOESM5_ESM.zip › Individual Simulations Pulse Decoder/45.png]

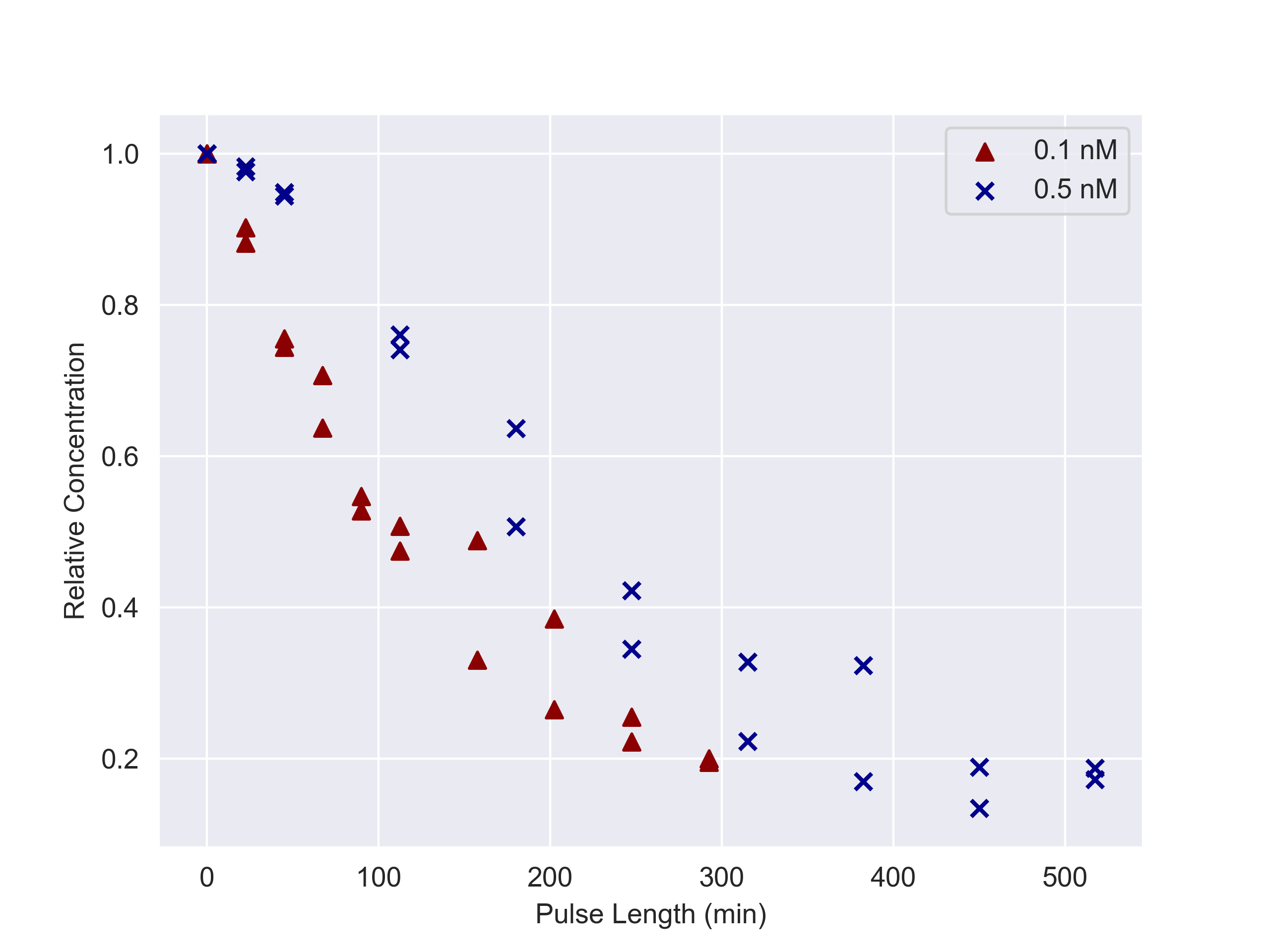

Supplement: Supplementary file 5 — Supplementary Dataset 2 [file 41467_2022_31306_MOESM5_ESM.zip › Individual Simulations Pulse Decoder/46.png]

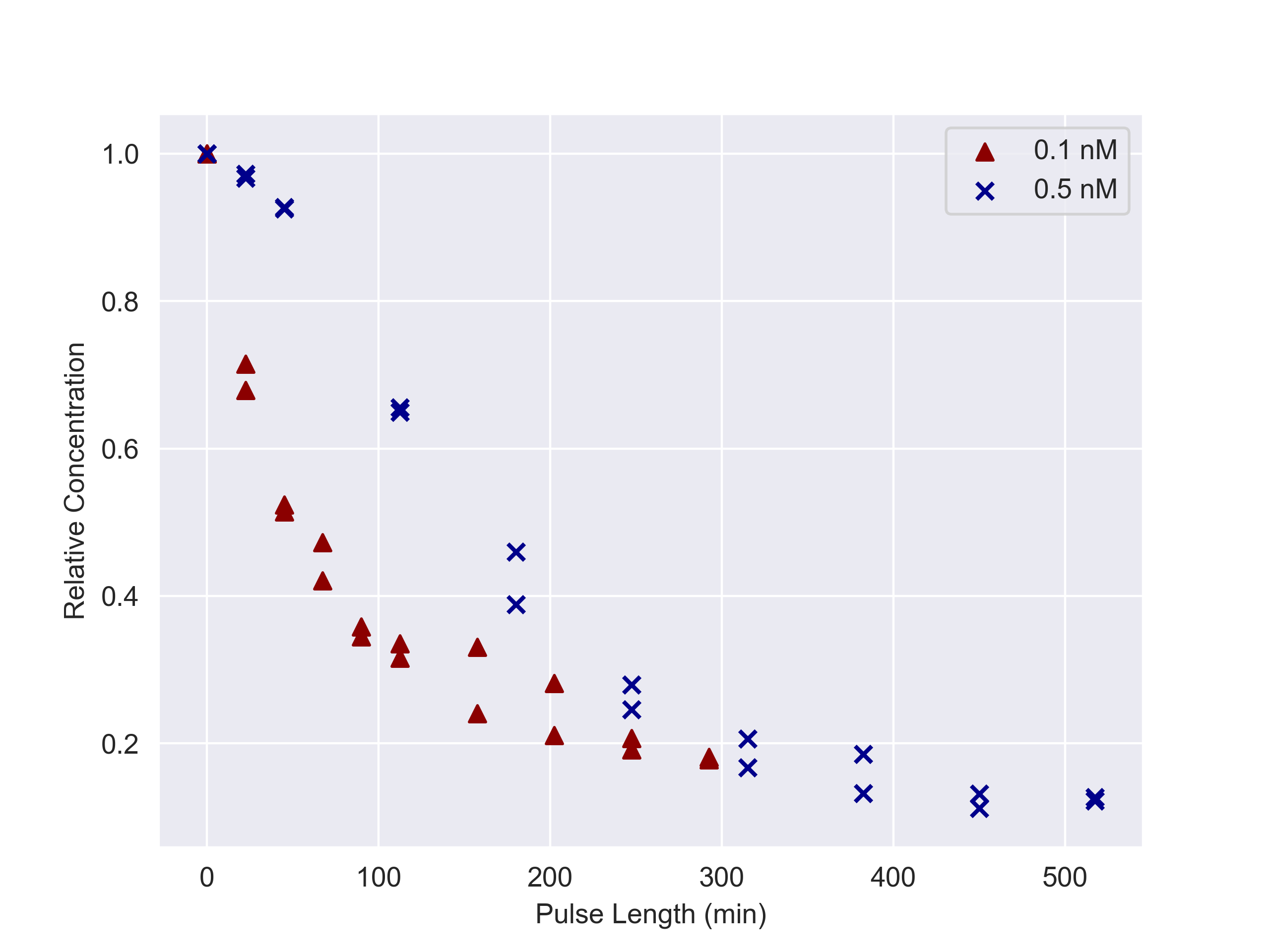

Supplement: Supplementary file 5 — Supplementary Dataset 2 [file 41467_2022_31306_MOESM5_ESM.zip › Individual Simulations Pulse Decoder/47.png]

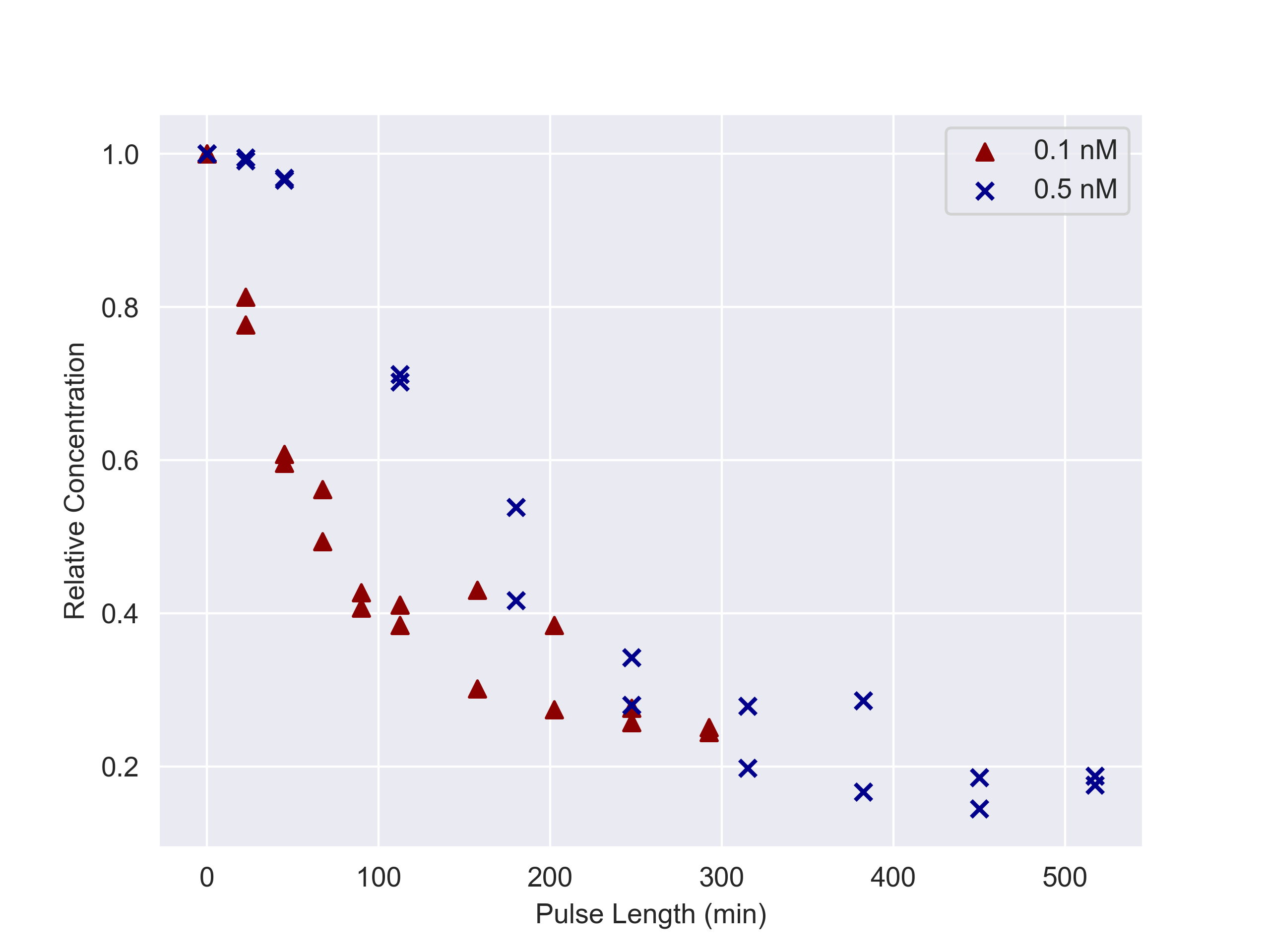

Supplement: Supplementary file 5 — Supplementary Dataset 2 [file 41467_2022_31306_MOESM5_ESM.zip › Individual Simulations Pulse Decoder/48.png]

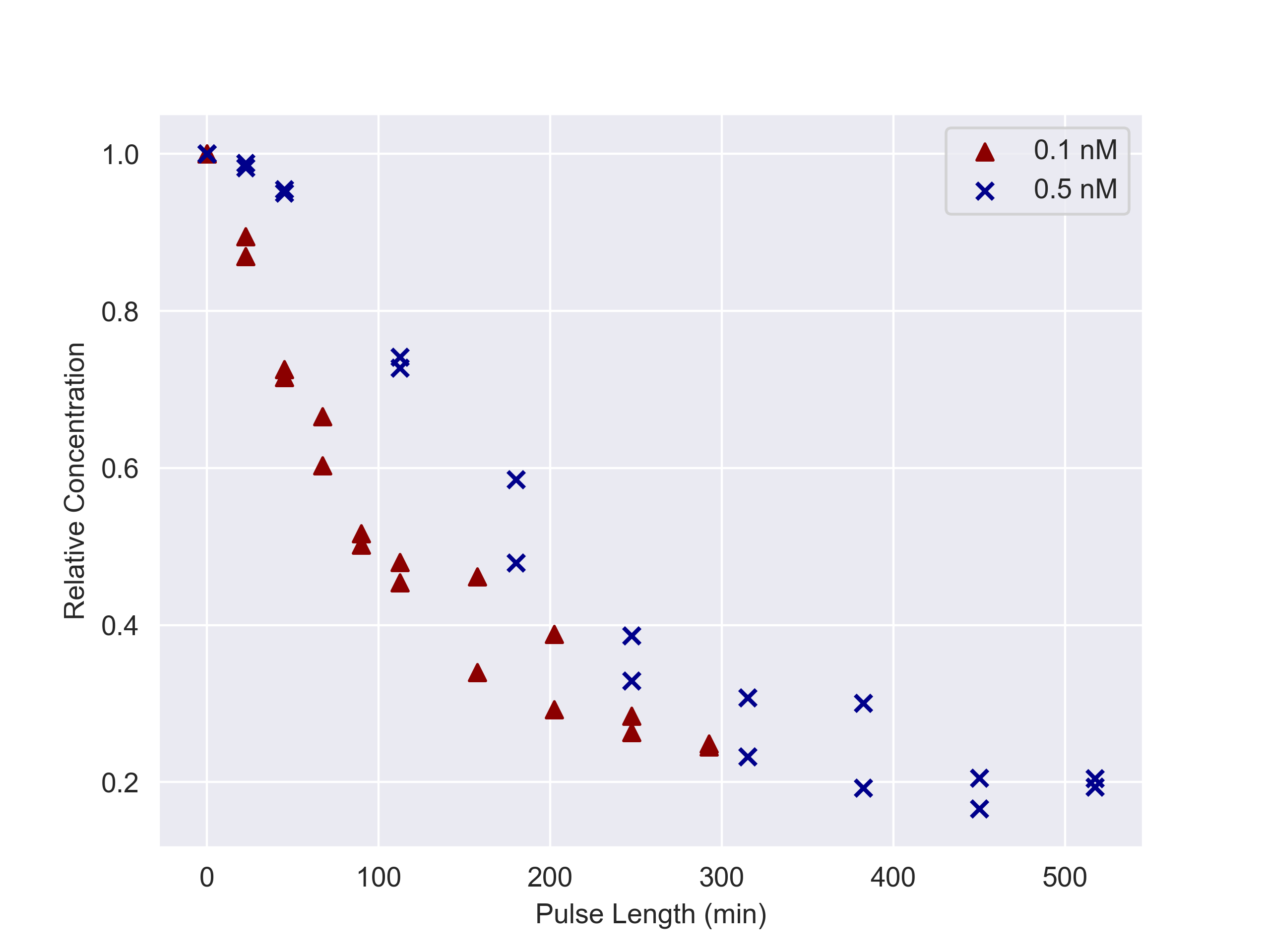

Supplement: Supplementary file 5 — Supplementary Dataset 2 [file 41467_2022_31306_MOESM5_ESM.zip › Individual Simulations Pulse Decoder/49.png]

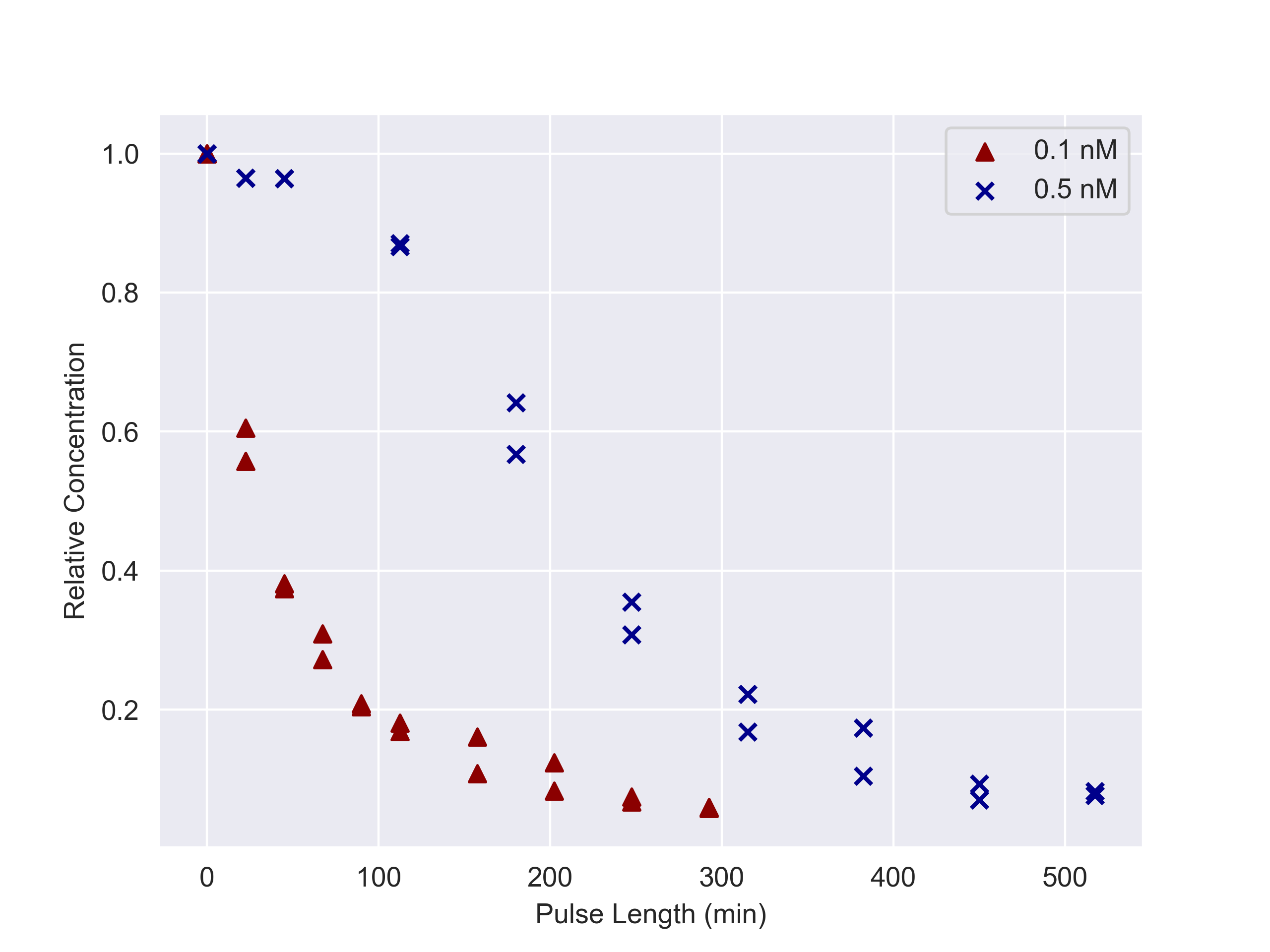

Supplement: Supplementary file 5 — Supplementary Dataset 2 [file 41467_2022_31306_MOESM5_ESM.zip › Individual Simulations Pulse Decoder/5.png]

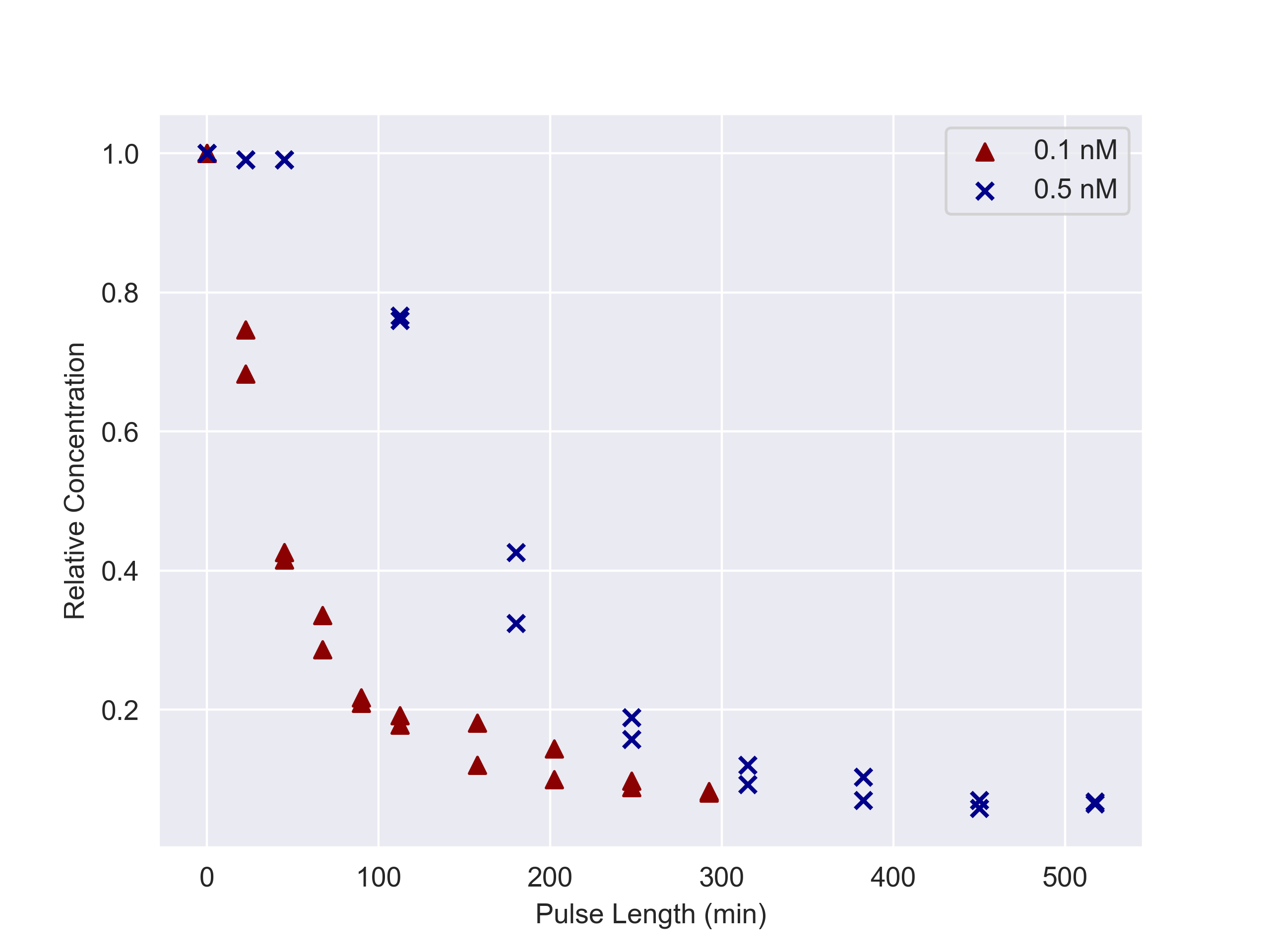

Supplement: Supplementary file 5 — Supplementary Dataset 2 [file 41467_2022_31306_MOESM5_ESM.zip › Individual Simulations Pulse Decoder/50.png]

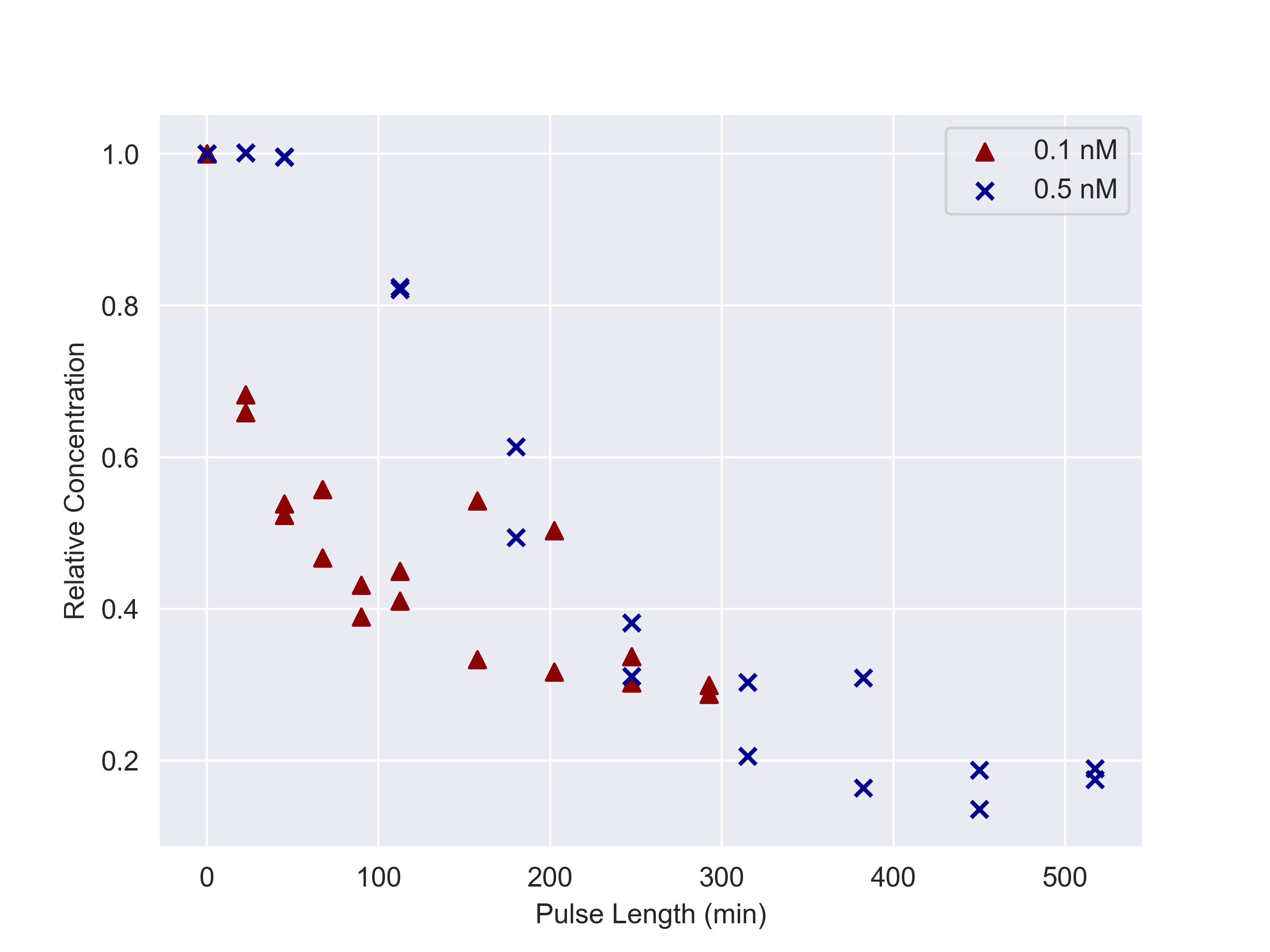

Supplement: Supplementary file 5 — Supplementary Dataset 2 [file 41467_2022_31306_MOESM5_ESM.zip › Individual Simulations Pulse Decoder/51.png]

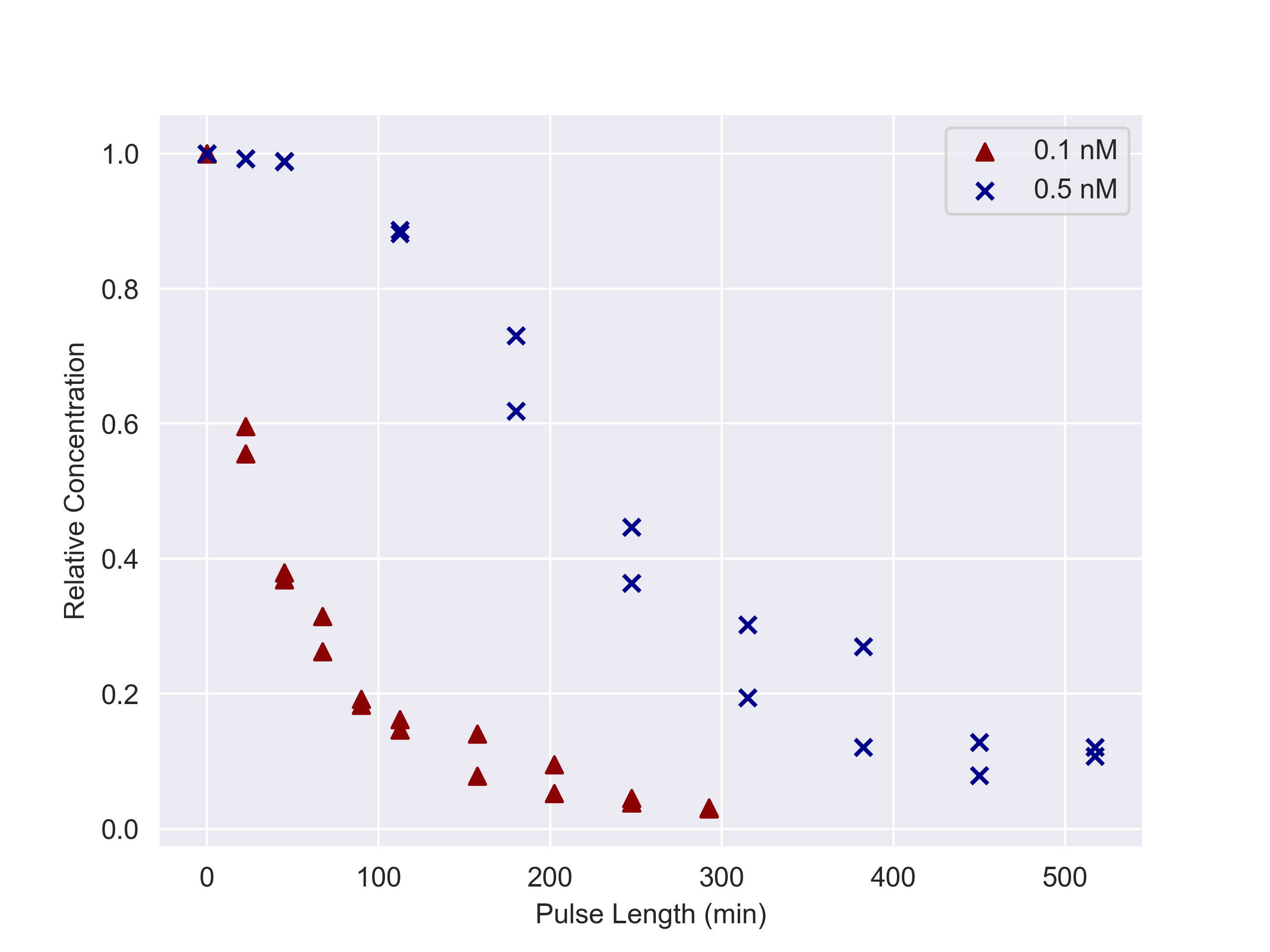

Supplement: Supplementary file 5 — Supplementary Dataset 2 [file 41467_2022_31306_MOESM5_ESM.zip › Individual Simulations Pulse Decoder/52.png]
