# Supplementary figures and images for: A microfluidic optimal experimental design platform for forward design of cell-free genetic networks (part 4 of 4)
Source: Nat Commun. 2022 Jun 24;13:3626. doi: 10.1038/s41467-022-31306-3 (PMC9232554; doi:10.1038/s41467-022-31306-3)

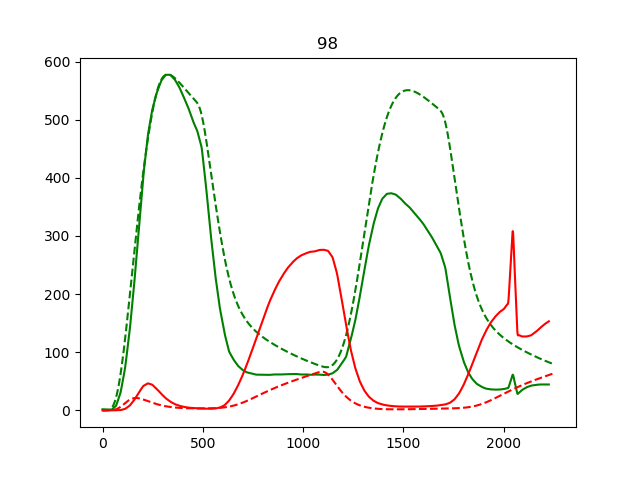

Supplement: Supplementary file 6 — Supplementary Dataset 3 [file 41467_2022_31306_MOESM6_ESM.zip › Individual Simulations Bistable Switch/98.png]

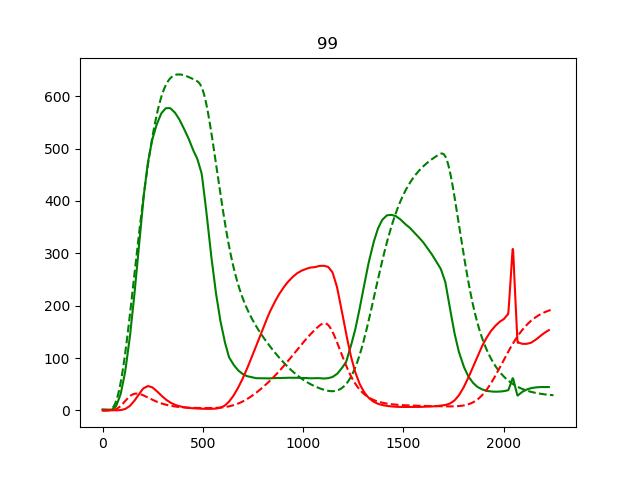

Supplement: Supplementary file 6 — Supplementary Dataset 3 [file 41467_2022_31306_MOESM6_ESM.zip › Individual Simulations Bistable Switch/99.png]
