# Supplementary figures and images for: A microfluidic optimal experimental design platform for forward design of cell-free genetic networks (part 3 of 4)
Source: Nat Commun. 2022 Jun 24;13:3626. doi: 10.1038/s41467-022-31306-3 (PMC9232554; doi:10.1038/s41467-022-31306-3)

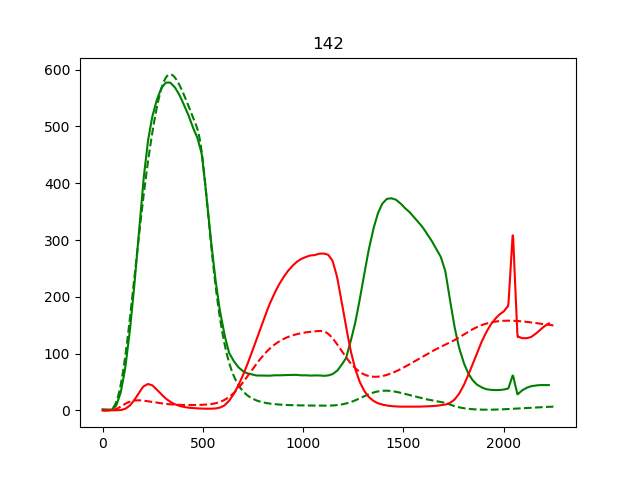

Supplement: Supplementary file 6 — Supplementary Dataset 3 [file 41467_2022_31306_MOESM6_ESM.zip › Individual Simulations Bistable Switch/142.png]

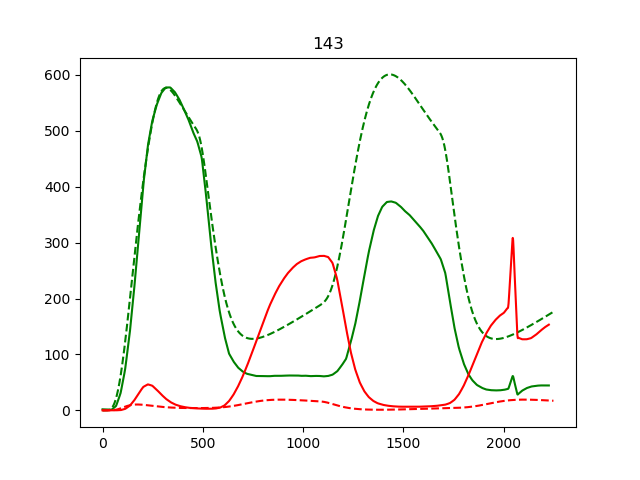

Supplement: Supplementary file 6 — Supplementary Dataset 3 [file 41467_2022_31306_MOESM6_ESM.zip › Individual Simulations Bistable Switch/143.png]

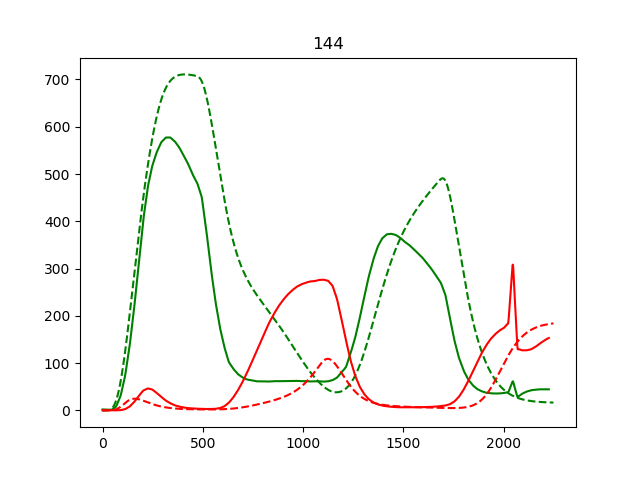

Supplement: Supplementary file 6 — Supplementary Dataset 3 [file 41467_2022_31306_MOESM6_ESM.zip › Individual Simulations Bistable Switch/144.png]

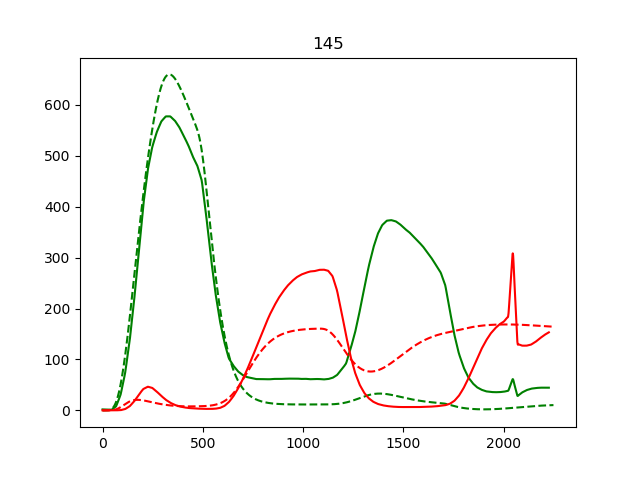

Supplement: Supplementary file 6 — Supplementary Dataset 3 [file 41467_2022_31306_MOESM6_ESM.zip › Individual Simulations Bistable Switch/145.png]

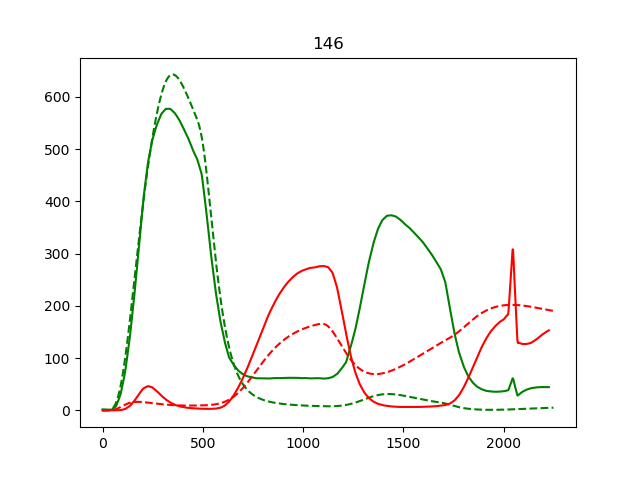

Supplement: Supplementary file 6 — Supplementary Dataset 3 [file 41467_2022_31306_MOESM6_ESM.zip › Individual Simulations Bistable Switch/146.png]

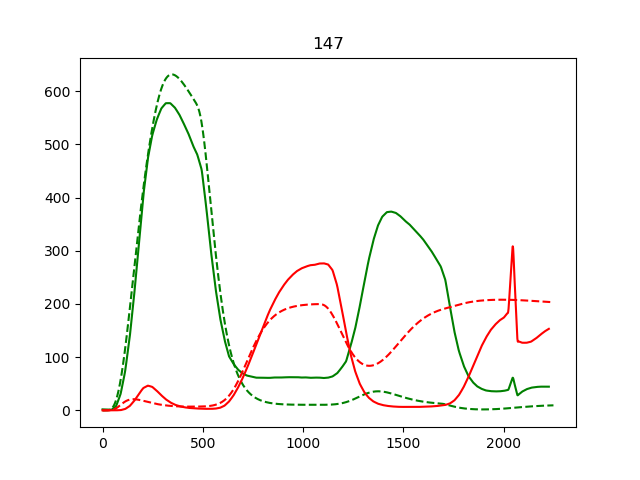

Supplement: Supplementary file 6 — Supplementary Dataset 3 [file 41467_2022_31306_MOESM6_ESM.zip › Individual Simulations Bistable Switch/147.png]

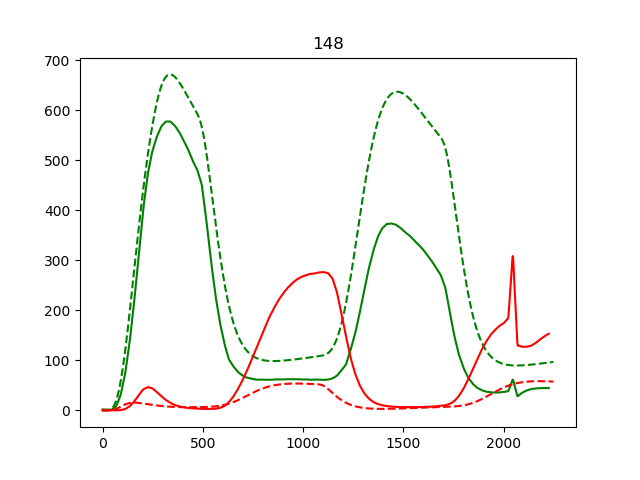

Supplement: Supplementary file 6 — Supplementary Dataset 3 [file 41467_2022_31306_MOESM6_ESM.zip › Individual Simulations Bistable Switch/148.png]

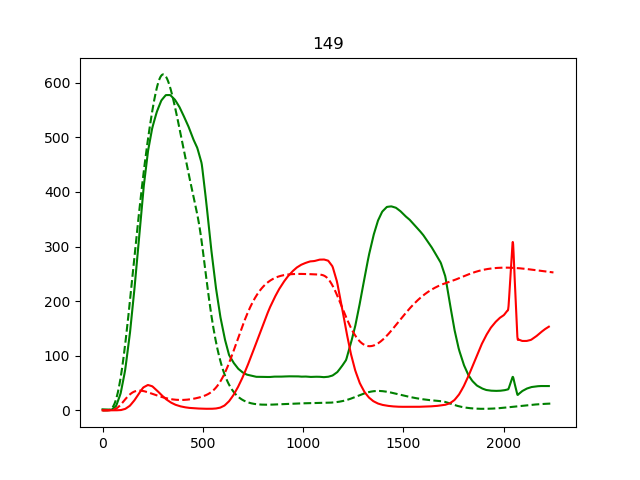

Supplement: Supplementary file 6 — Supplementary Dataset 3 [file 41467_2022_31306_MOESM6_ESM.zip › Individual Simulations Bistable Switch/149.png]

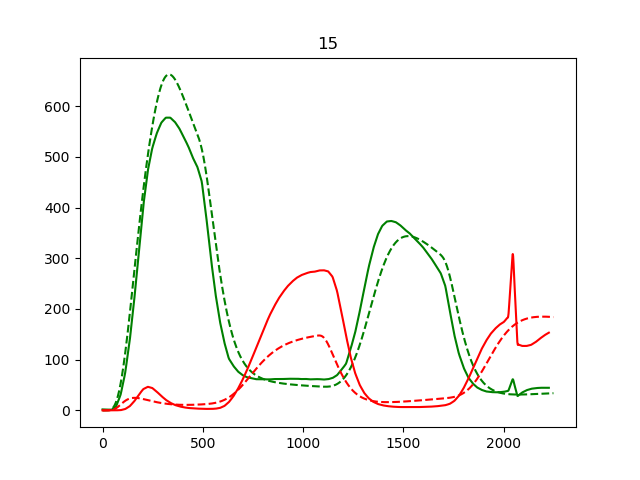

Supplement: Supplementary file 6 — Supplementary Dataset 3 [file 41467_2022_31306_MOESM6_ESM.zip › Individual Simulations Bistable Switch/15.png]

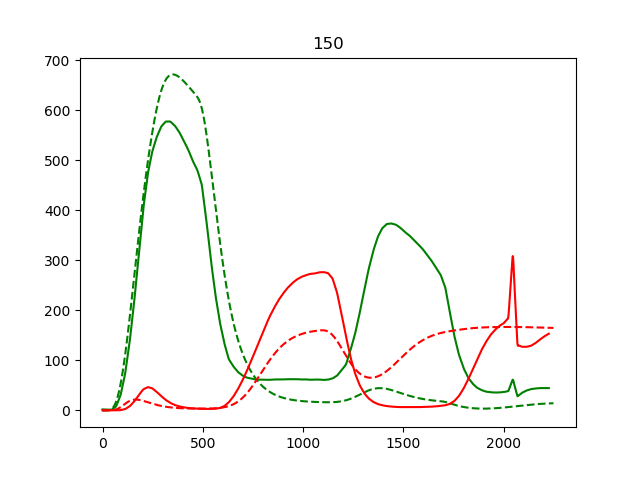

Supplement: Supplementary file 6 — Supplementary Dataset 3 [file 41467_2022_31306_MOESM6_ESM.zip › Individual Simulations Bistable Switch/150.png]

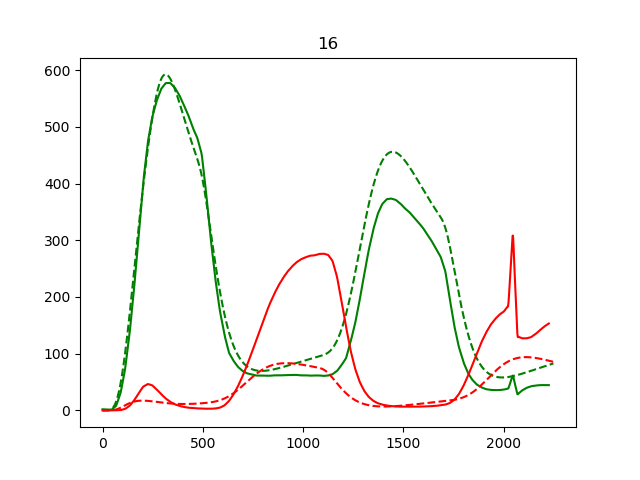

Supplement: Supplementary file 6 — Supplementary Dataset 3 [file 41467_2022_31306_MOESM6_ESM.zip › Individual Simulations Bistable Switch/16.png]

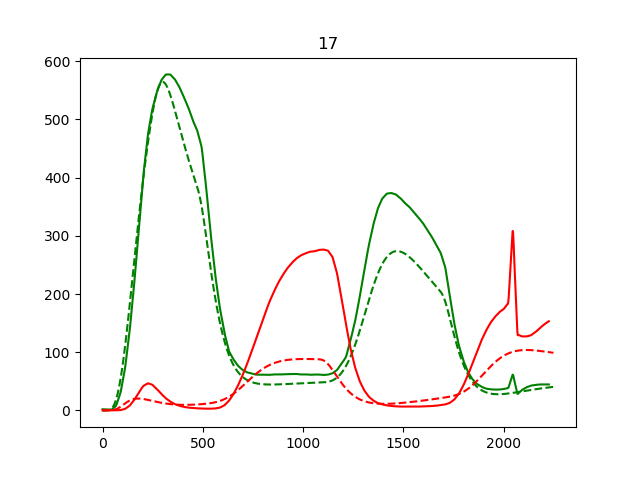

Supplement: Supplementary file 6 — Supplementary Dataset 3 [file 41467_2022_31306_MOESM6_ESM.zip › Individual Simulations Bistable Switch/17.png]

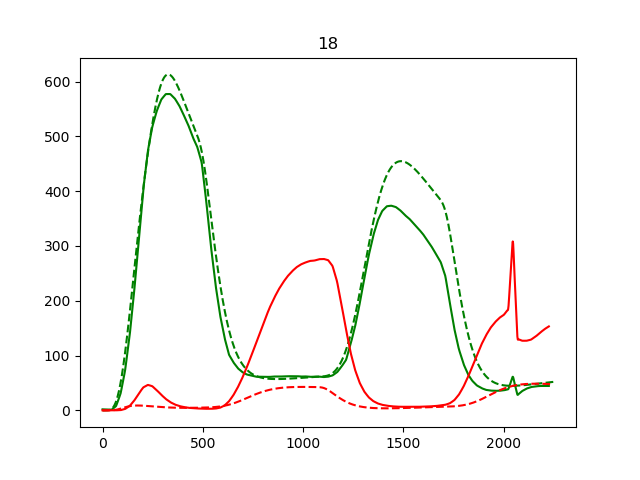

Supplement: Supplementary file 6 — Supplementary Dataset 3 [file 41467_2022_31306_MOESM6_ESM.zip › Individual Simulations Bistable Switch/18.png]

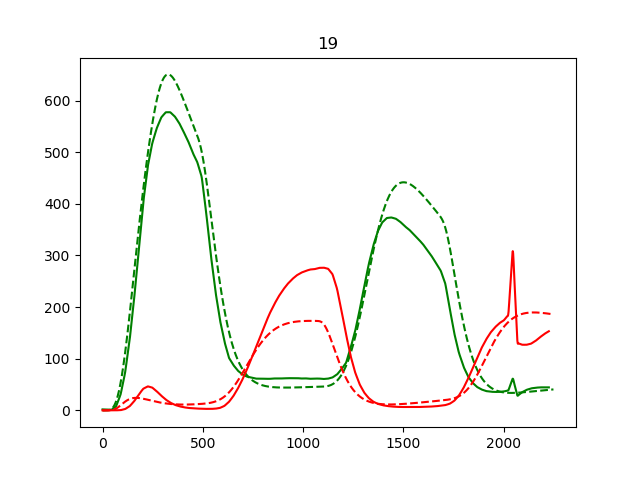

Supplement: Supplementary file 6 — Supplementary Dataset 3 [file 41467_2022_31306_MOESM6_ESM.zip › Individual Simulations Bistable Switch/19.png]

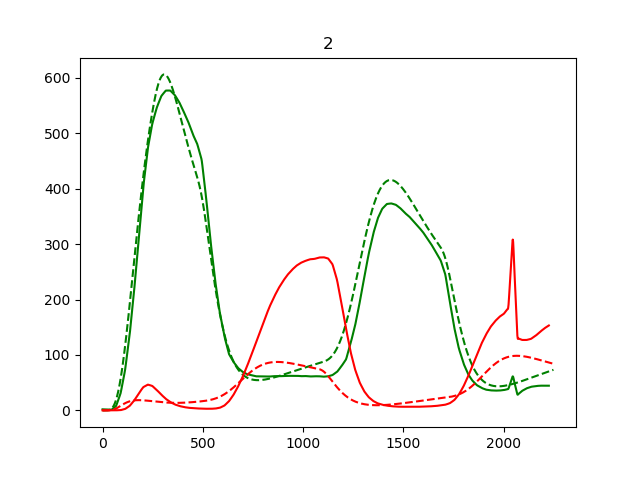

Supplement: Supplementary file 6 — Supplementary Dataset 3 [file 41467_2022_31306_MOESM6_ESM.zip › Individual Simulations Bistable Switch/2.png]

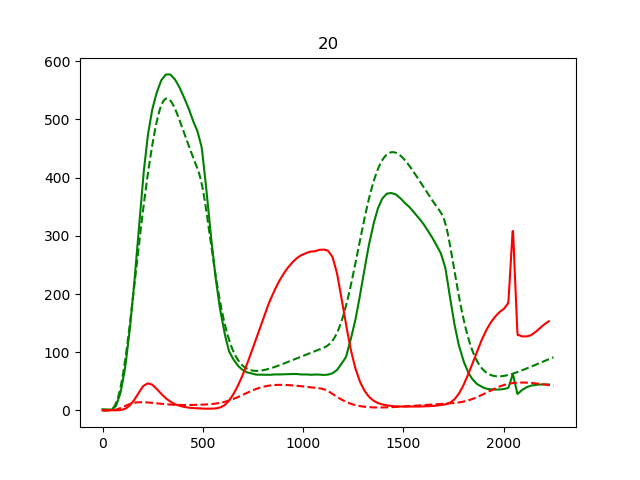

Supplement: Supplementary file 6 — Supplementary Dataset 3 [file 41467_2022_31306_MOESM6_ESM.zip › Individual Simulations Bistable Switch/20.png]

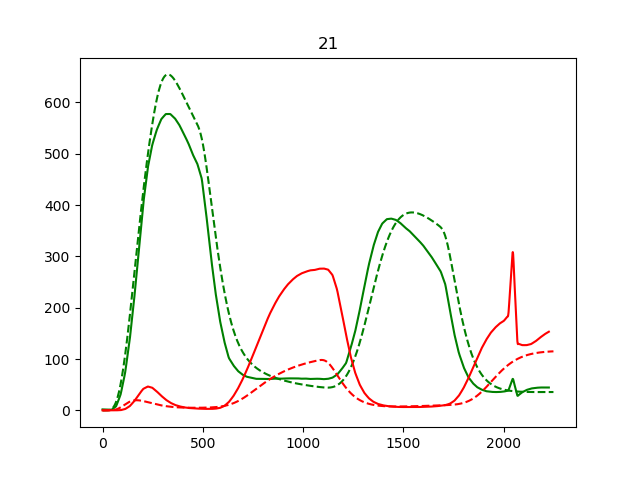

Supplement: Supplementary file 6 — Supplementary Dataset 3 [file 41467_2022_31306_MOESM6_ESM.zip › Individual Simulations Bistable Switch/21.png]

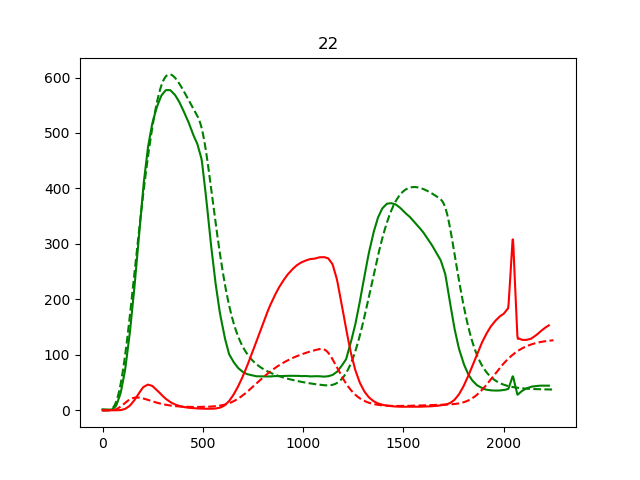

Supplement: Supplementary file 6 — Supplementary Dataset 3 [file 41467_2022_31306_MOESM6_ESM.zip › Individual Simulations Bistable Switch/22.png]

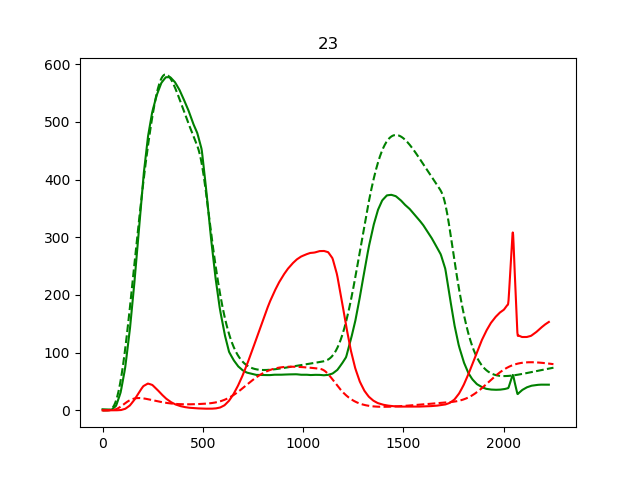

Supplement: Supplementary file 6 — Supplementary Dataset 3 [file 41467_2022_31306_MOESM6_ESM.zip › Individual Simulations Bistable Switch/23.png]

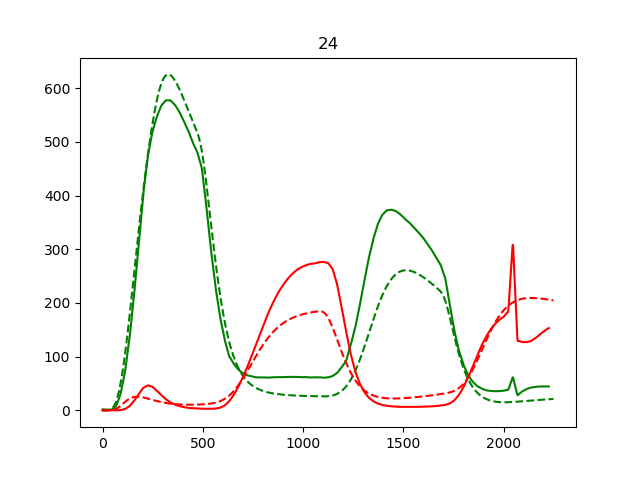

Supplement: Supplementary file 6 — Supplementary Dataset 3 [file 41467_2022_31306_MOESM6_ESM.zip › Individual Simulations Bistable Switch/24.png]

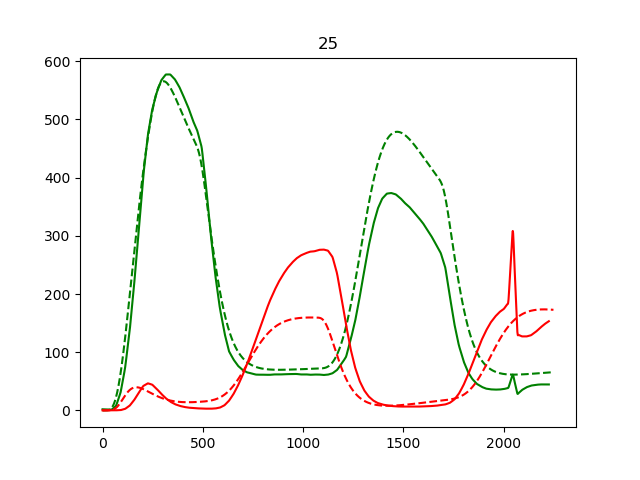

Supplement: Supplementary file 6 — Supplementary Dataset 3 [file 41467_2022_31306_MOESM6_ESM.zip › Individual Simulations Bistable Switch/25.png]

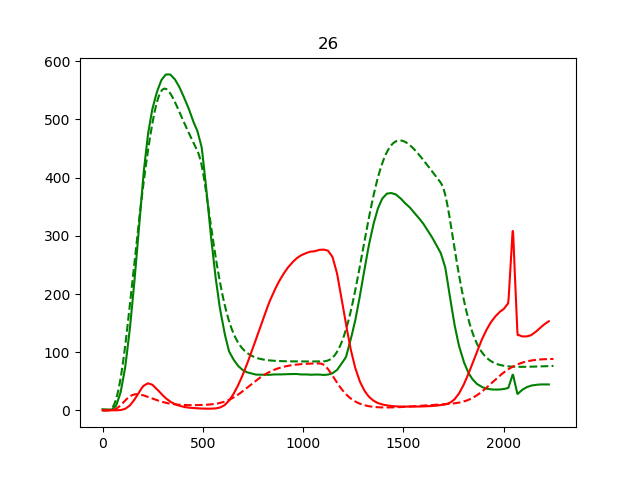

Supplement: Supplementary file 6 — Supplementary Dataset 3 [file 41467_2022_31306_MOESM6_ESM.zip › Individual Simulations Bistable Switch/26.png]

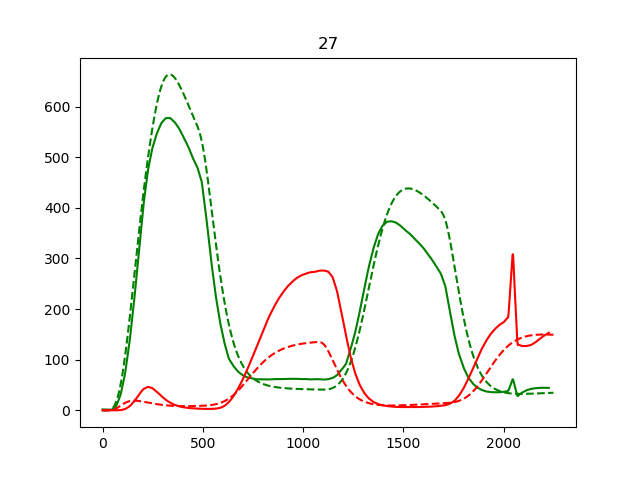

Supplement: Supplementary file 6 — Supplementary Dataset 3 [file 41467_2022_31306_MOESM6_ESM.zip › Individual Simulations Bistable Switch/27.png]

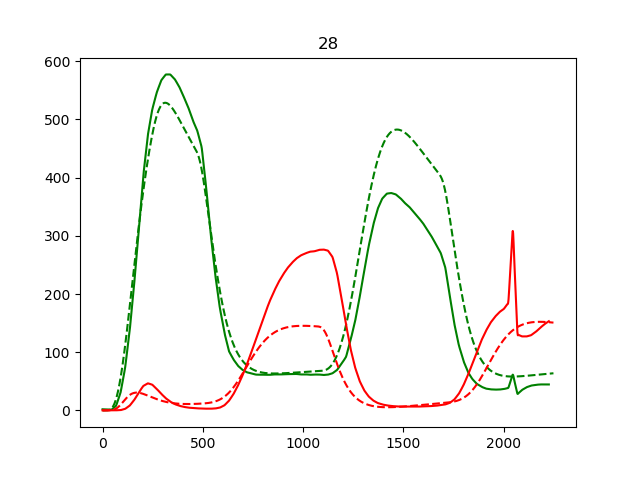

Supplement: Supplementary file 6 — Supplementary Dataset 3 [file 41467_2022_31306_MOESM6_ESM.zip › Individual Simulations Bistable Switch/28.png]

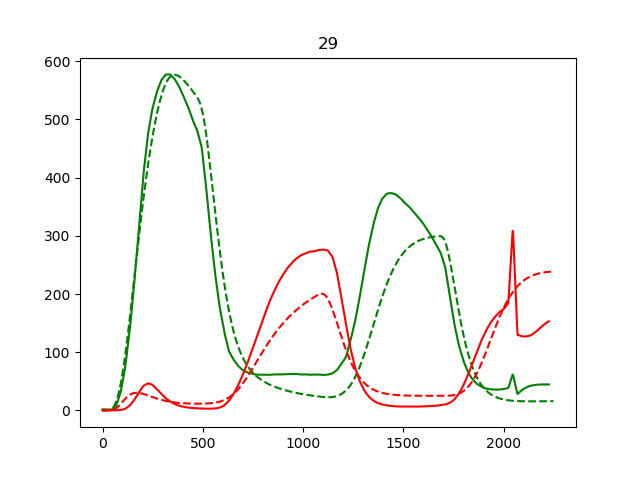

Supplement: Supplementary file 6 — Supplementary Dataset 3 [file 41467_2022_31306_MOESM6_ESM.zip › Individual Simulations Bistable Switch/29.png]

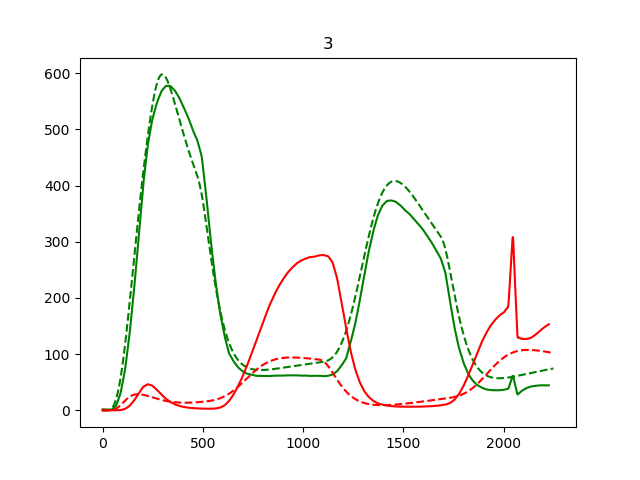

Supplement: Supplementary file 6 — Supplementary Dataset 3 [file 41467_2022_31306_MOESM6_ESM.zip › Individual Simulations Bistable Switch/3.png]

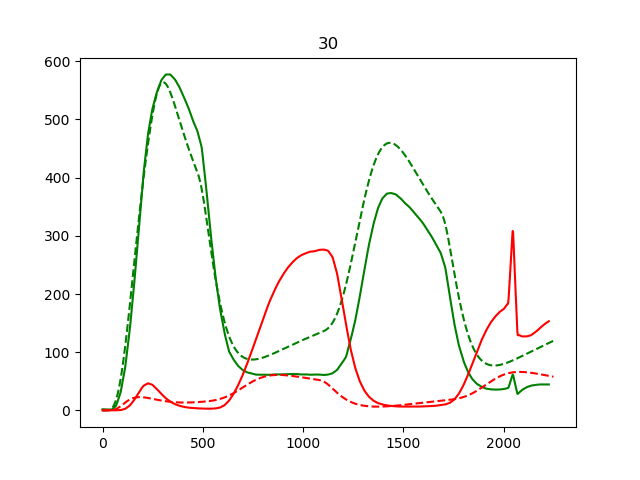

Supplement: Supplementary file 6 — Supplementary Dataset 3 [file 41467_2022_31306_MOESM6_ESM.zip › Individual Simulations Bistable Switch/30.png]

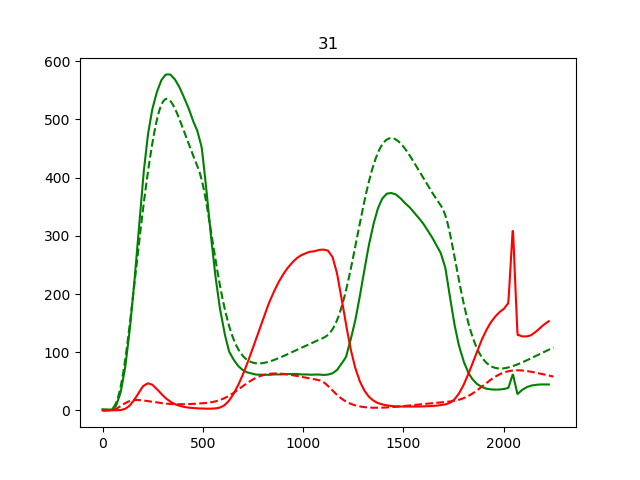

Supplement: Supplementary file 6 — Supplementary Dataset 3 [file 41467_2022_31306_MOESM6_ESM.zip › Individual Simulations Bistable Switch/31.png]

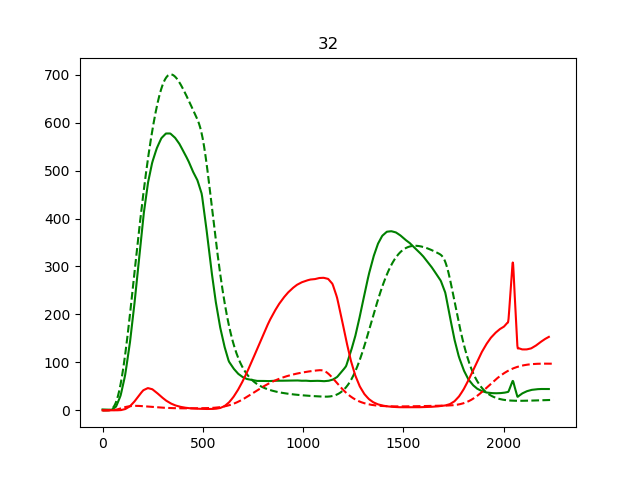

Supplement: Supplementary file 6 — Supplementary Dataset 3 [file 41467_2022_31306_MOESM6_ESM.zip › Individual Simulations Bistable Switch/32.png]

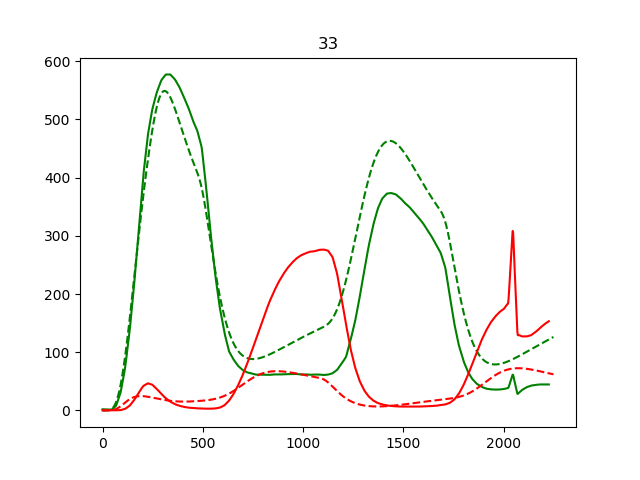

Supplement: Supplementary file 6 — Supplementary Dataset 3 [file 41467_2022_31306_MOESM6_ESM.zip › Individual Simulations Bistable Switch/33.png]

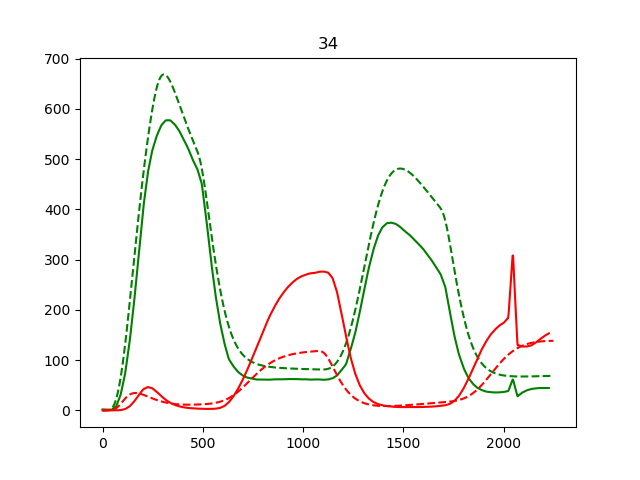

Supplement: Supplementary file 6 — Supplementary Dataset 3 [file 41467_2022_31306_MOESM6_ESM.zip › Individual Simulations Bistable Switch/34.png]

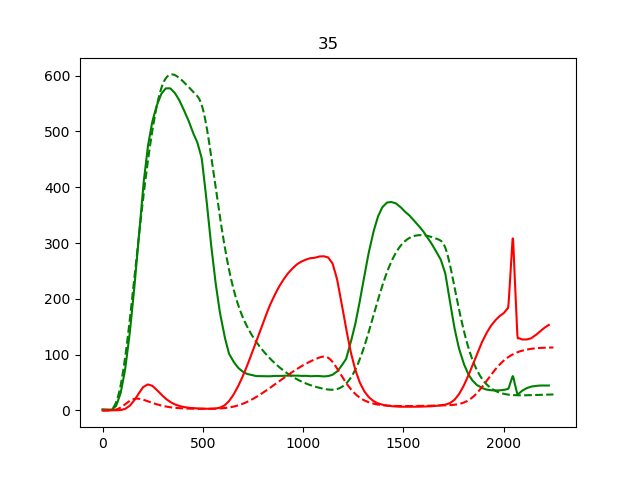

Supplement: Supplementary file 6 — Supplementary Dataset 3 [file 41467_2022_31306_MOESM6_ESM.zip › Individual Simulations Bistable Switch/35.png]

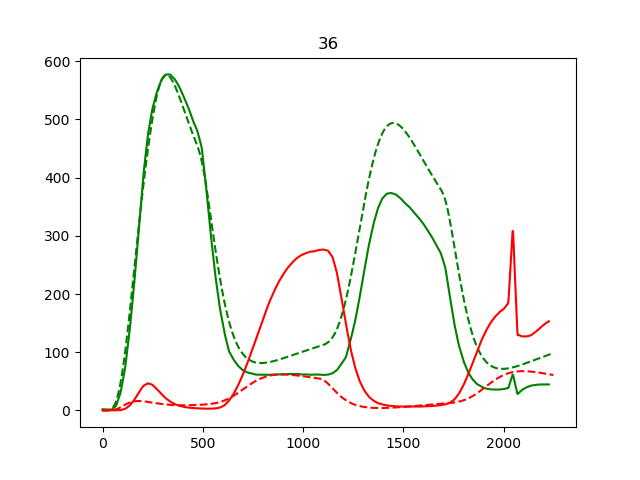

Supplement: Supplementary file 6 — Supplementary Dataset 3 [file 41467_2022_31306_MOESM6_ESM.zip › Individual Simulations Bistable Switch/36.png]

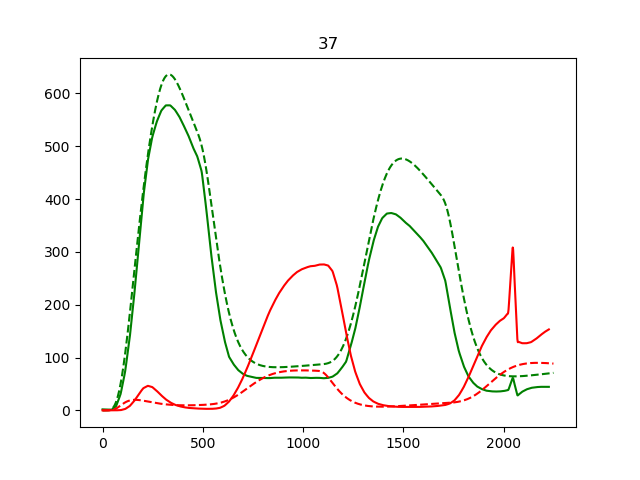

Supplement: Supplementary file 6 — Supplementary Dataset 3 [file 41467_2022_31306_MOESM6_ESM.zip › Individual Simulations Bistable Switch/37.png]

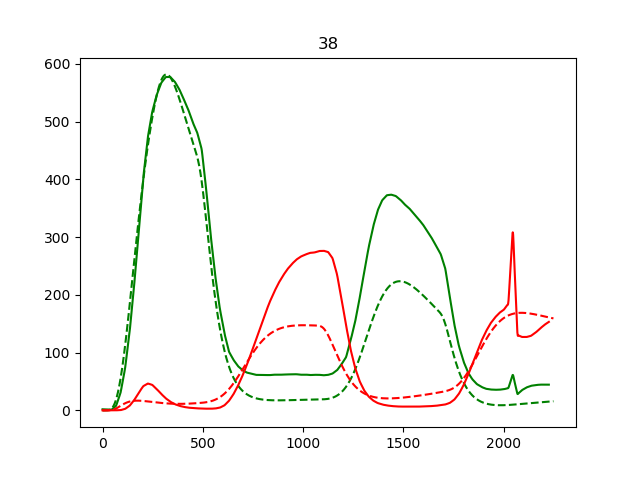

Supplement: Supplementary file 6 — Supplementary Dataset 3 [file 41467_2022_31306_MOESM6_ESM.zip › Individual Simulations Bistable Switch/38.png]

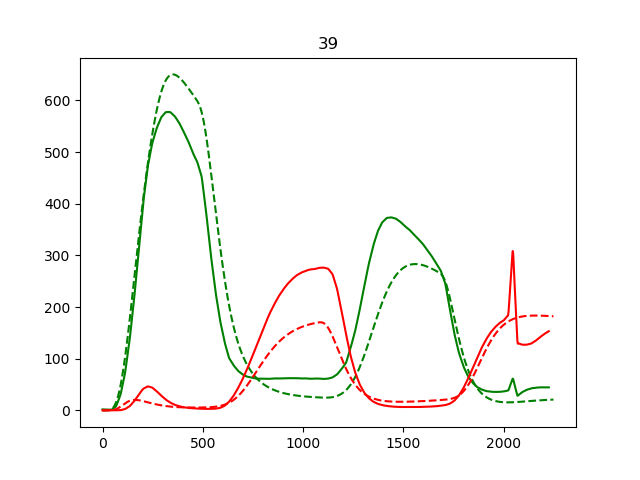

Supplement: Supplementary file 6 — Supplementary Dataset 3 [file 41467_2022_31306_MOESM6_ESM.zip › Individual Simulations Bistable Switch/39.png]

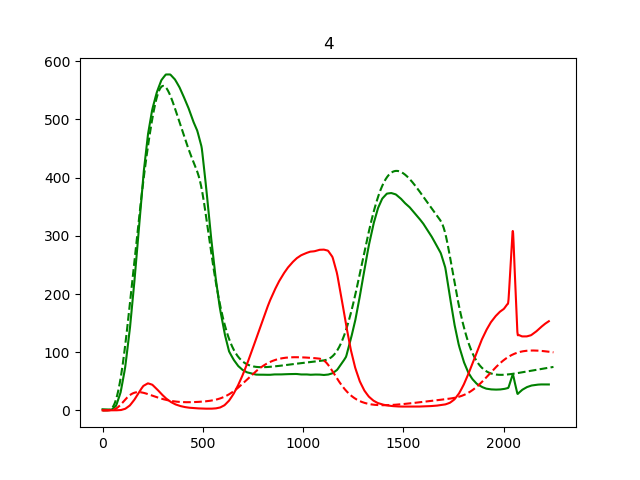

Supplement: Supplementary file 6 — Supplementary Dataset 3 [file 41467_2022_31306_MOESM6_ESM.zip › Individual Simulations Bistable Switch/4.png]

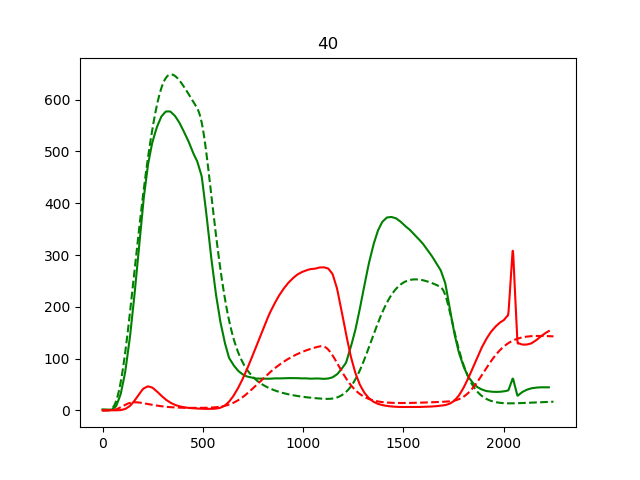

Supplement: Supplementary file 6 — Supplementary Dataset 3 [file 41467_2022_31306_MOESM6_ESM.zip › Individual Simulations Bistable Switch/40.png]

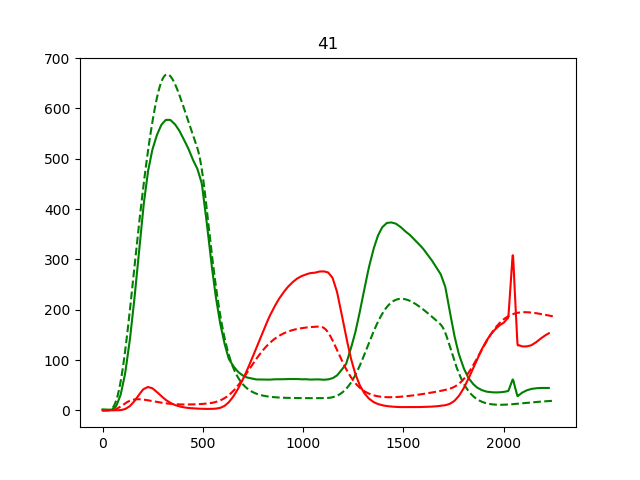

Supplement: Supplementary file 6 — Supplementary Dataset 3 [file 41467_2022_31306_MOESM6_ESM.zip › Individual Simulations Bistable Switch/41.png]

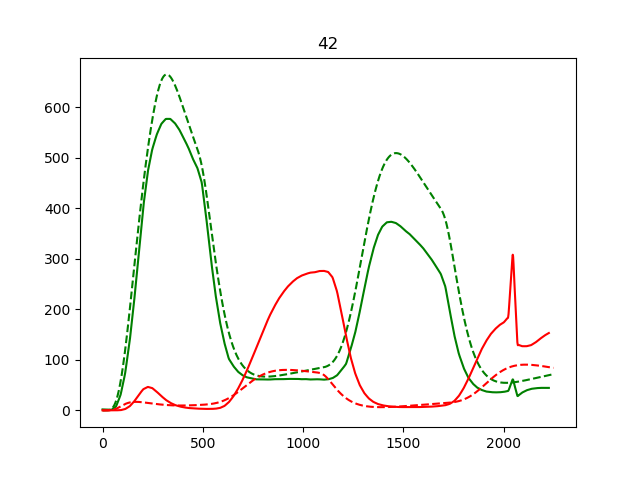

Supplement: Supplementary file 6 — Supplementary Dataset 3 [file 41467_2022_31306_MOESM6_ESM.zip › Individual Simulations Bistable Switch/42.png]

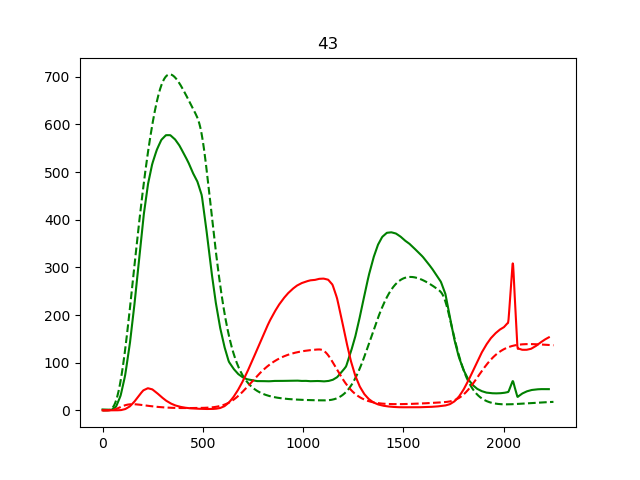

Supplement: Supplementary file 6 — Supplementary Dataset 3 [file 41467_2022_31306_MOESM6_ESM.zip › Individual Simulations Bistable Switch/43.png]

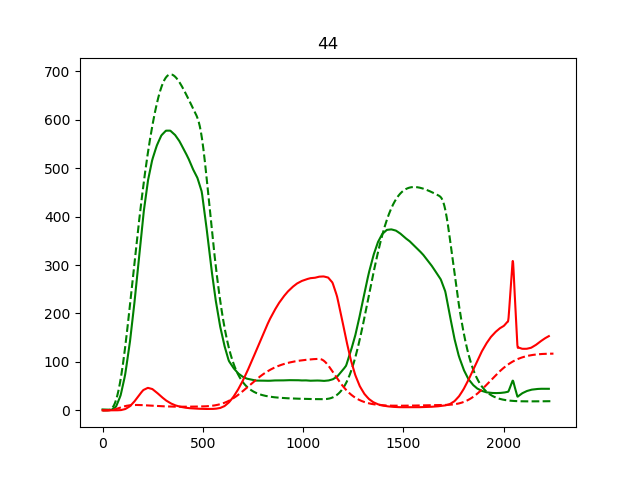

Supplement: Supplementary file 6 — Supplementary Dataset 3 [file 41467_2022_31306_MOESM6_ESM.zip › Individual Simulations Bistable Switch/44.png]

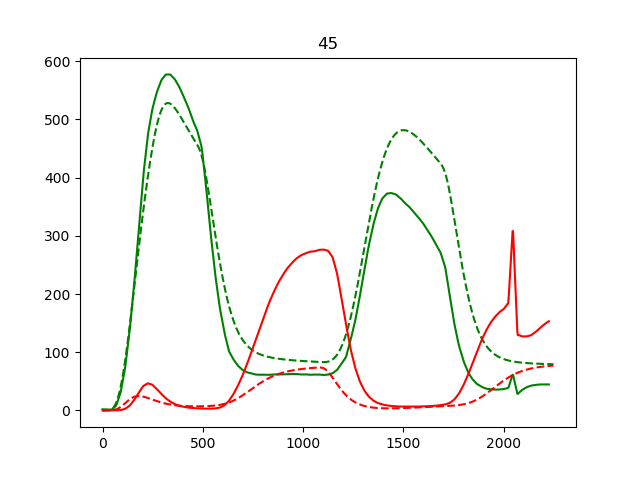

Supplement: Supplementary file 6 — Supplementary Dataset 3 [file 41467_2022_31306_MOESM6_ESM.zip › Individual Simulations Bistable Switch/45.png]

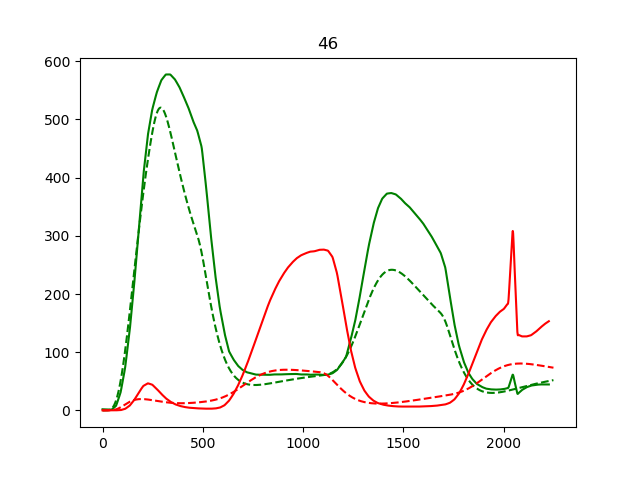

Supplement: Supplementary file 6 — Supplementary Dataset 3 [file 41467_2022_31306_MOESM6_ESM.zip › Individual Simulations Bistable Switch/46.png]

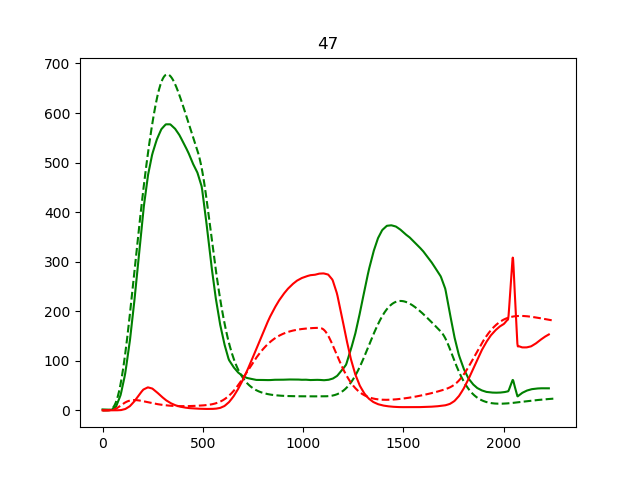

Supplement: Supplementary file 6 — Supplementary Dataset 3 [file 41467_2022_31306_MOESM6_ESM.zip › Individual Simulations Bistable Switch/47.png]

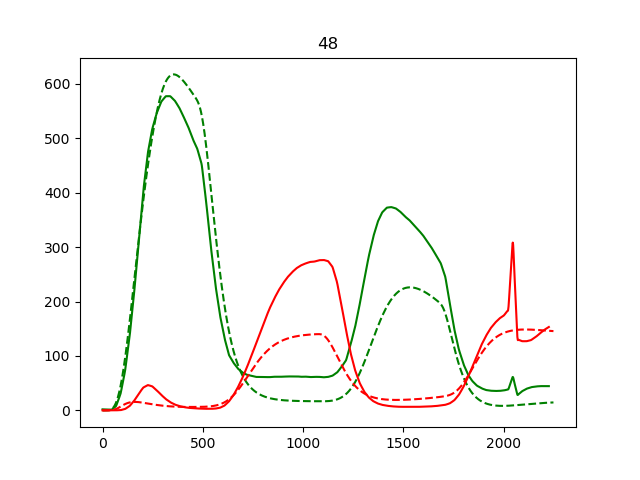

Supplement: Supplementary file 6 — Supplementary Dataset 3 [file 41467_2022_31306_MOESM6_ESM.zip › Individual Simulations Bistable Switch/48.png]

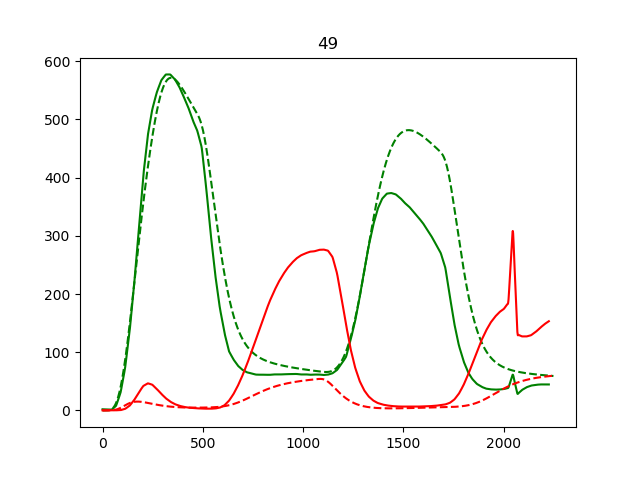

Supplement: Supplementary file 6 — Supplementary Dataset 3 [file 41467_2022_31306_MOESM6_ESM.zip › Individual Simulations Bistable Switch/49.png]

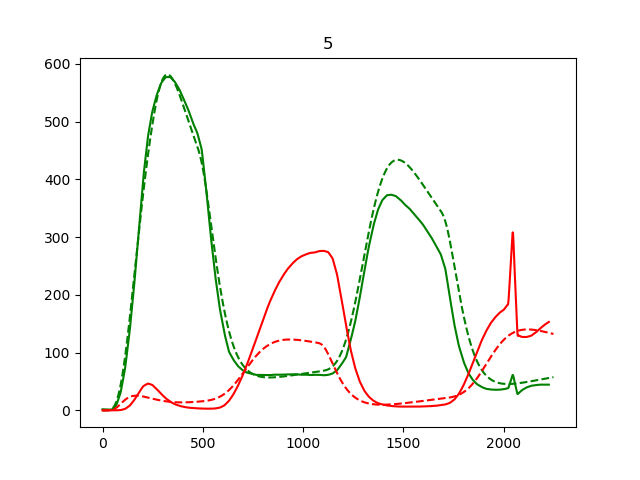

Supplement: Supplementary file 6 — Supplementary Dataset 3 [file 41467_2022_31306_MOESM6_ESM.zip › Individual Simulations Bistable Switch/5.png]

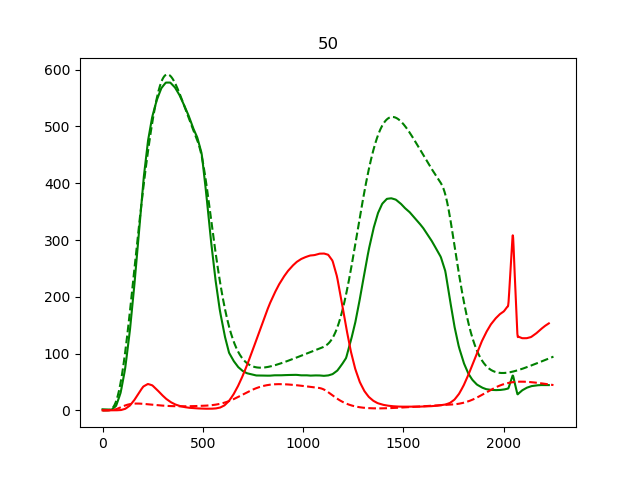

Supplement: Supplementary file 6 — Supplementary Dataset 3 [file 41467_2022_31306_MOESM6_ESM.zip › Individual Simulations Bistable Switch/50.png]

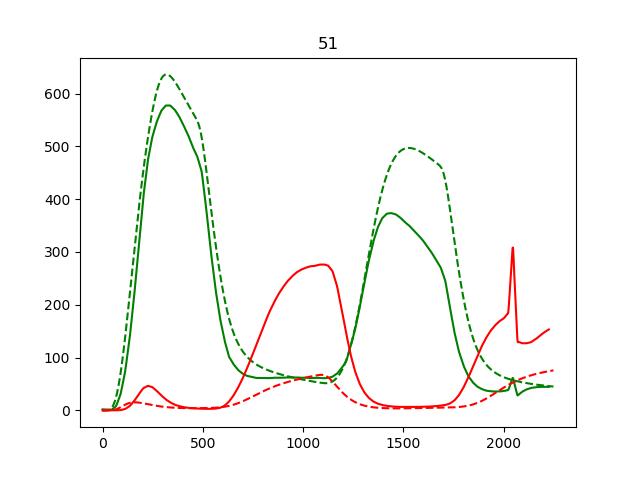

Supplement: Supplementary file 6 — Supplementary Dataset 3 [file 41467_2022_31306_MOESM6_ESM.zip › Individual Simulations Bistable Switch/51.png]

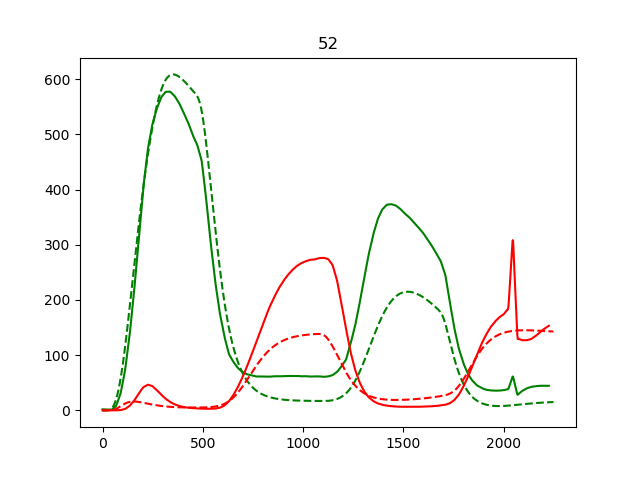

Supplement: Supplementary file 6 — Supplementary Dataset 3 [file 41467_2022_31306_MOESM6_ESM.zip › Individual Simulations Bistable Switch/52.png]

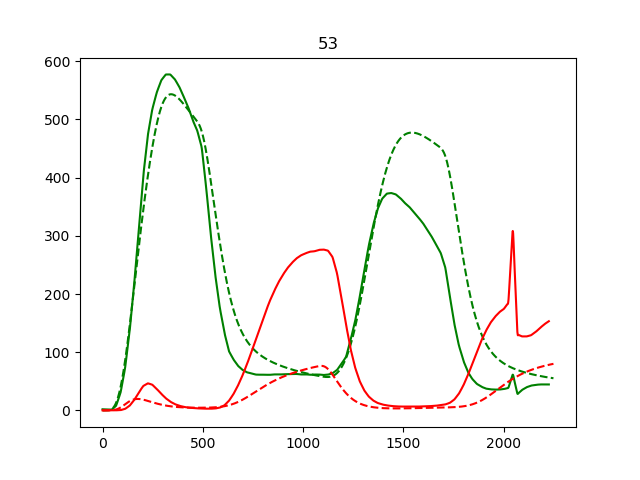

Supplement: Supplementary file 6 — Supplementary Dataset 3 [file 41467_2022_31306_MOESM6_ESM.zip › Individual Simulations Bistable Switch/53.png]

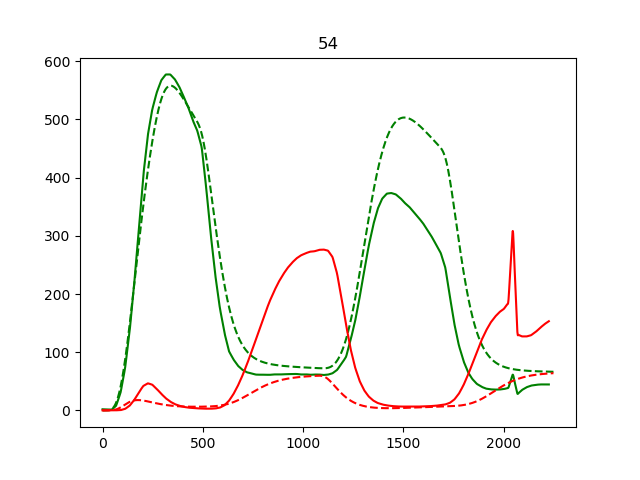

Supplement: Supplementary file 6 — Supplementary Dataset 3 [file 41467_2022_31306_MOESM6_ESM.zip › Individual Simulations Bistable Switch/54.png]

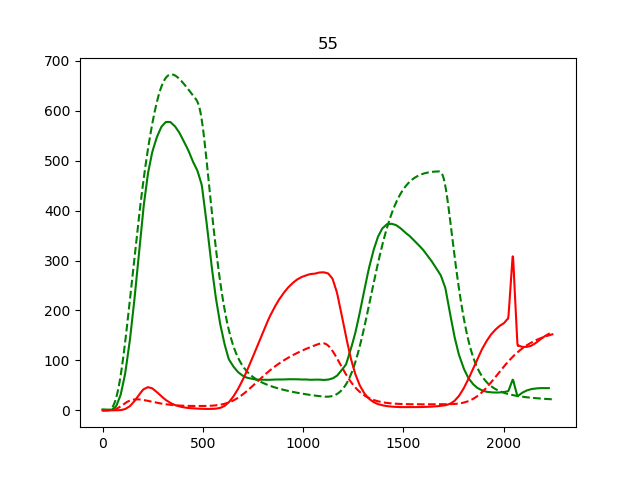

Supplement: Supplementary file 6 — Supplementary Dataset 3 [file 41467_2022_31306_MOESM6_ESM.zip › Individual Simulations Bistable Switch/55.png]

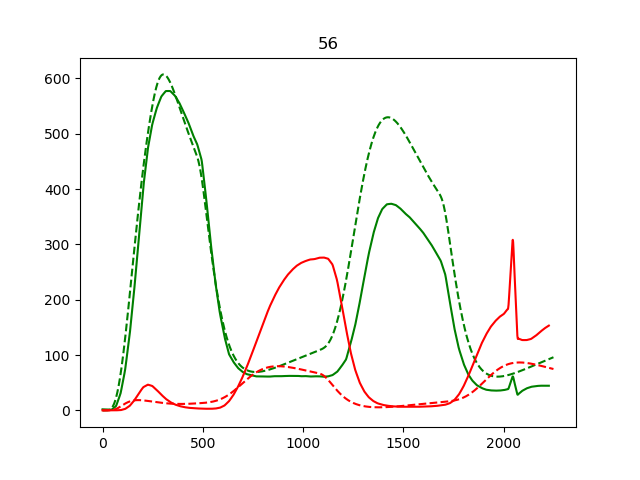

Supplement: Supplementary file 6 — Supplementary Dataset 3 [file 41467_2022_31306_MOESM6_ESM.zip › Individual Simulations Bistable Switch/56.png]

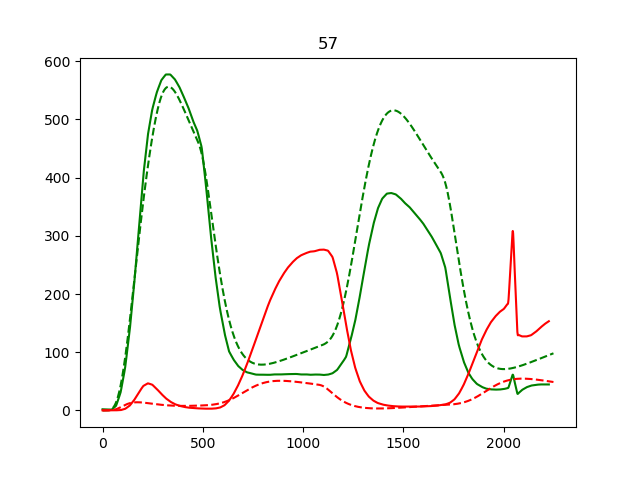

Supplement: Supplementary file 6 — Supplementary Dataset 3 [file 41467_2022_31306_MOESM6_ESM.zip › Individual Simulations Bistable Switch/57.png]

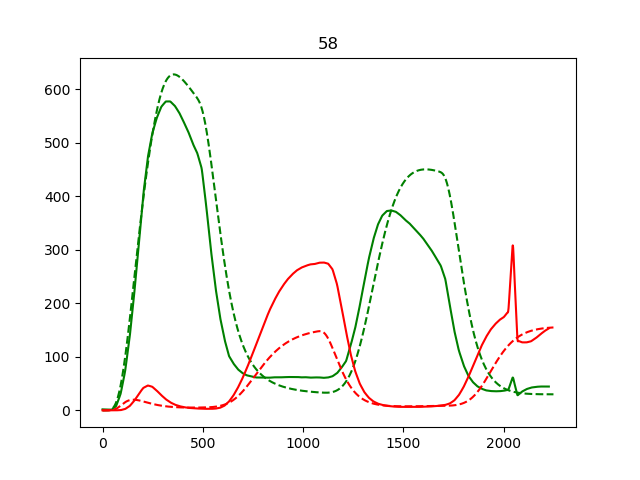

Supplement: Supplementary file 6 — Supplementary Dataset 3 [file 41467_2022_31306_MOESM6_ESM.zip › Individual Simulations Bistable Switch/58.png]

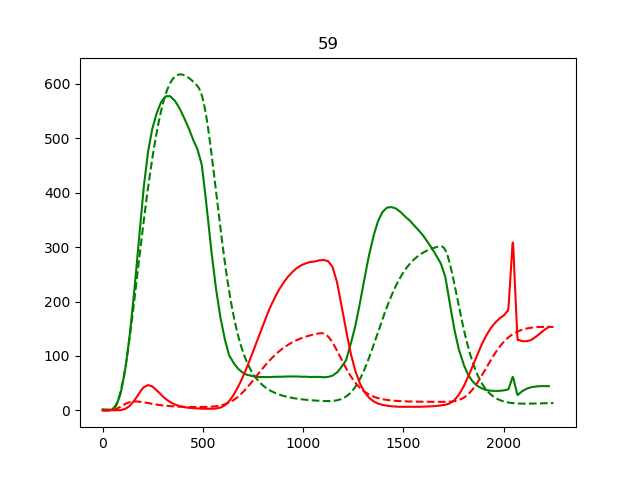

Supplement: Supplementary file 6 — Supplementary Dataset 3 [file 41467_2022_31306_MOESM6_ESM.zip › Individual Simulations Bistable Switch/59.png]

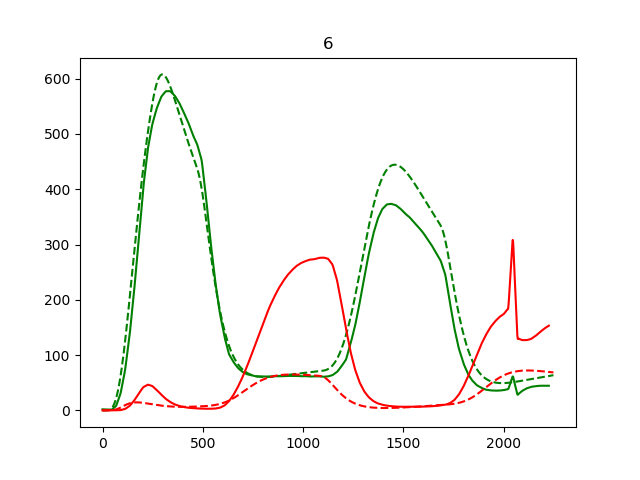

Supplement: Supplementary file 6 — Supplementary Dataset 3 [file 41467_2022_31306_MOESM6_ESM.zip › Individual Simulations Bistable Switch/6.png]

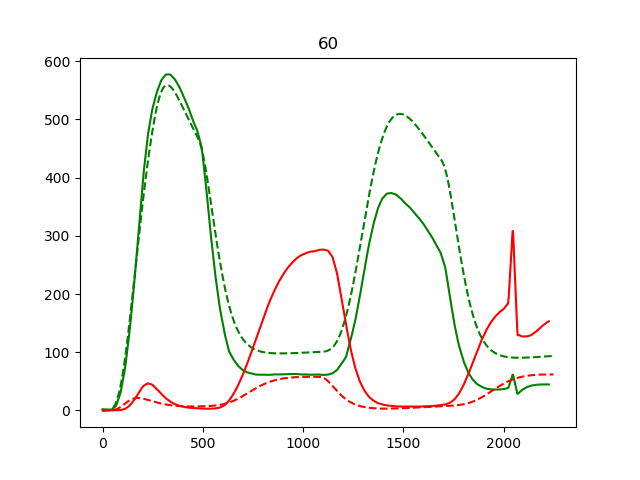

Supplement: Supplementary file 6 — Supplementary Dataset 3 [file 41467_2022_31306_MOESM6_ESM.zip › Individual Simulations Bistable Switch/60.png]

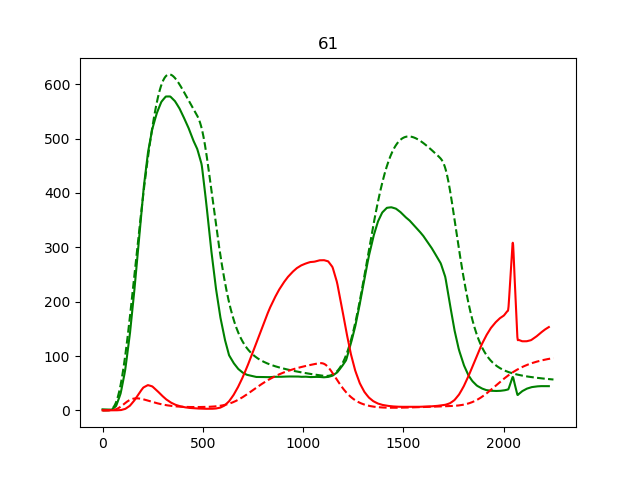

Supplement: Supplementary file 6 — Supplementary Dataset 3 [file 41467_2022_31306_MOESM6_ESM.zip › Individual Simulations Bistable Switch/61.png]

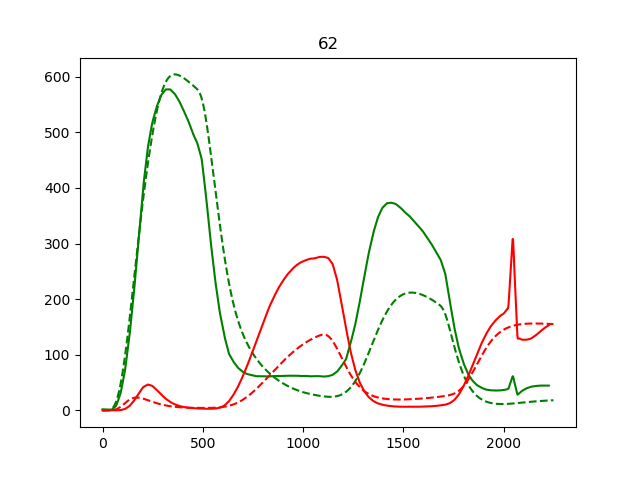

Supplement: Supplementary file 6 — Supplementary Dataset 3 [file 41467_2022_31306_MOESM6_ESM.zip › Individual Simulations Bistable Switch/62.png]

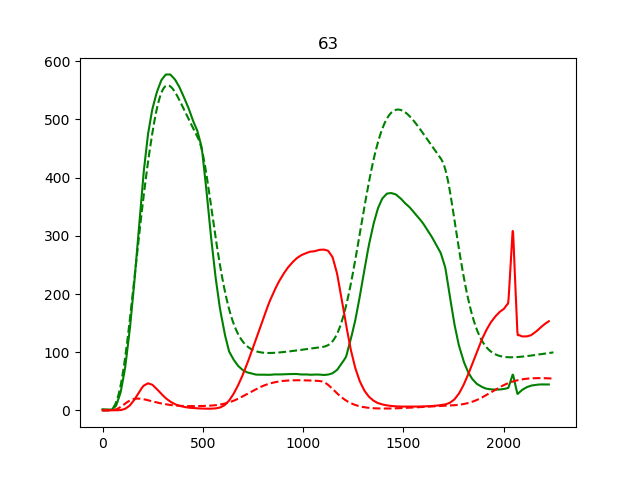

Supplement: Supplementary file 6 — Supplementary Dataset 3 [file 41467_2022_31306_MOESM6_ESM.zip › Individual Simulations Bistable Switch/63.png]

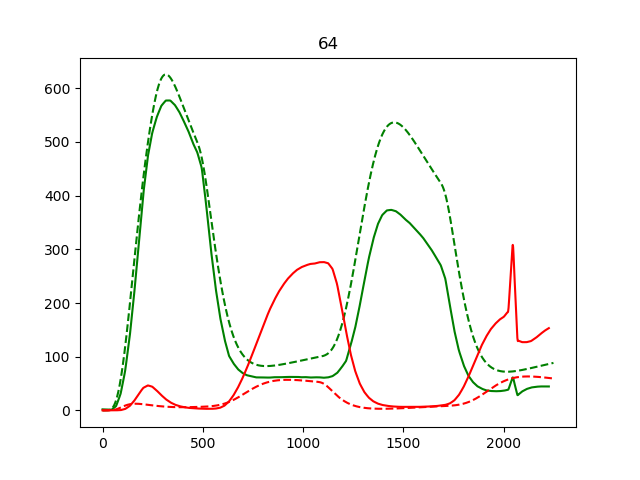

Supplement: Supplementary file 6 — Supplementary Dataset 3 [file 41467_2022_31306_MOESM6_ESM.zip › Individual Simulations Bistable Switch/64.png]

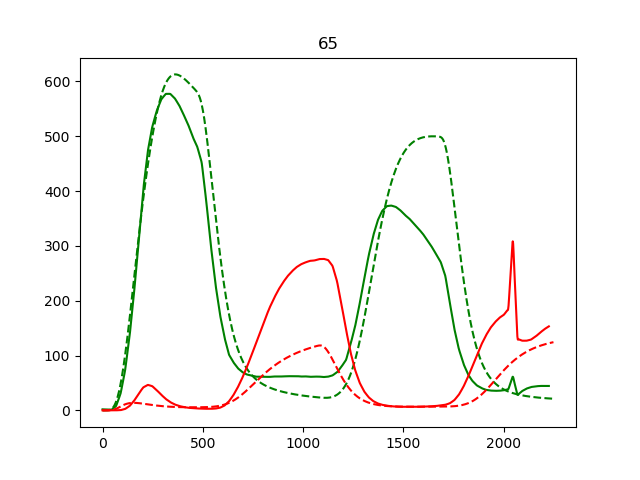

Supplement: Supplementary file 6 — Supplementary Dataset 3 [file 41467_2022_31306_MOESM6_ESM.zip › Individual Simulations Bistable Switch/65.png]

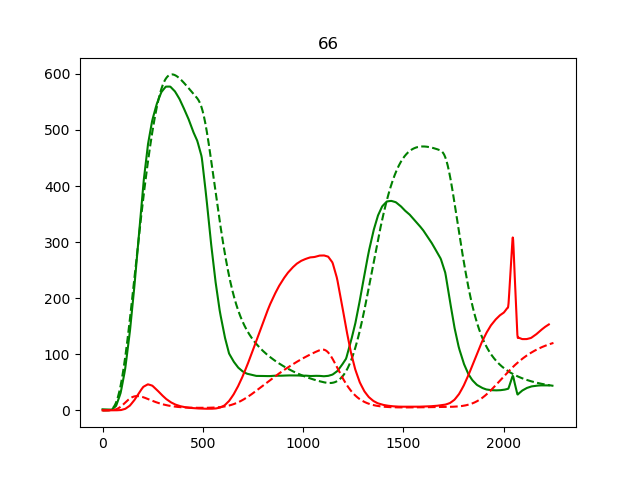

Supplement: Supplementary file 6 — Supplementary Dataset 3 [file 41467_2022_31306_MOESM6_ESM.zip › Individual Simulations Bistable Switch/66.png]

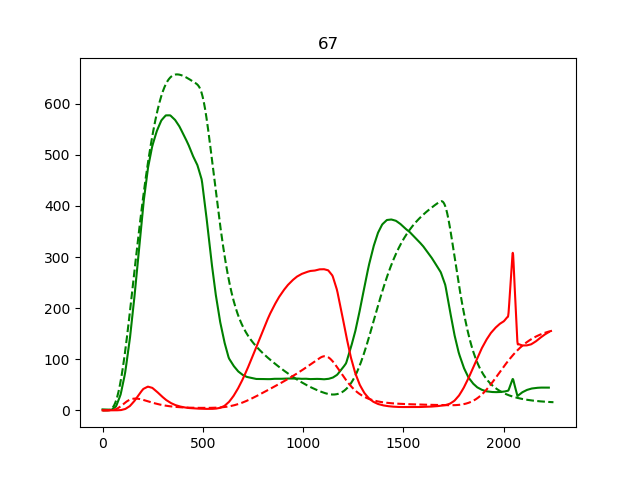

Supplement: Supplementary file 6 — Supplementary Dataset 3 [file 41467_2022_31306_MOESM6_ESM.zip › Individual Simulations Bistable Switch/67.png]

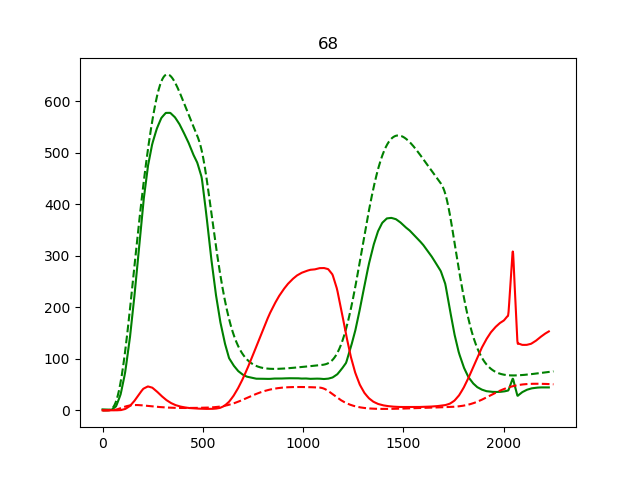

Supplement: Supplementary file 6 — Supplementary Dataset 3 [file 41467_2022_31306_MOESM6_ESM.zip › Individual Simulations Bistable Switch/68.png]

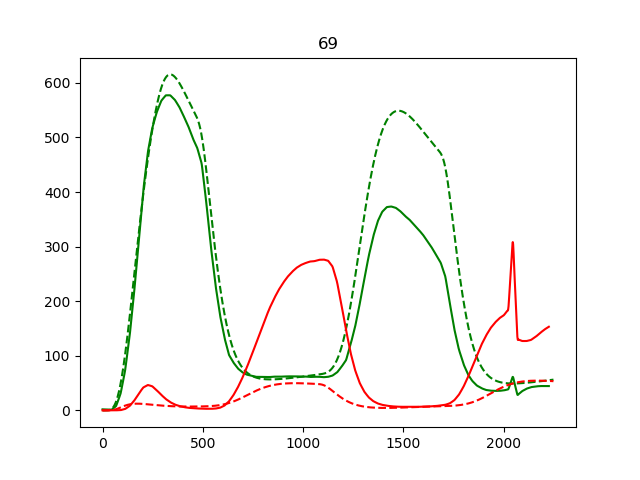

Supplement: Supplementary file 6 — Supplementary Dataset 3 [file 41467_2022_31306_MOESM6_ESM.zip › Individual Simulations Bistable Switch/69.png]

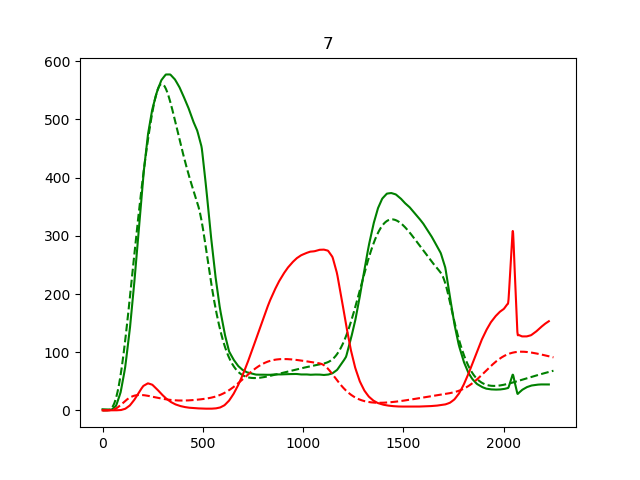

Supplement: Supplementary file 6 — Supplementary Dataset 3 [file 41467_2022_31306_MOESM6_ESM.zip › Individual Simulations Bistable Switch/7.png]

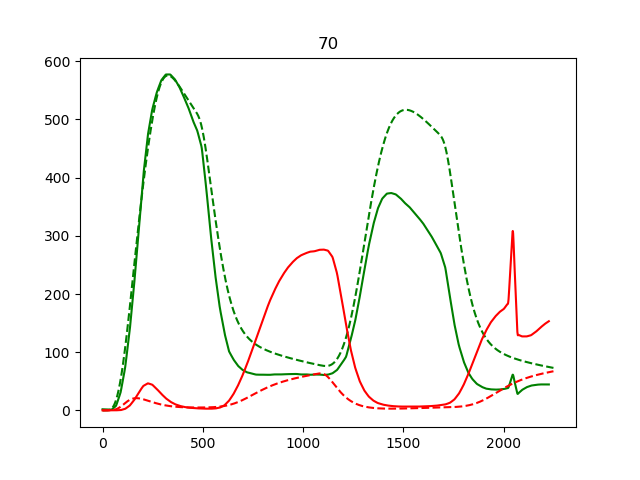

Supplement: Supplementary file 6 — Supplementary Dataset 3 [file 41467_2022_31306_MOESM6_ESM.zip › Individual Simulations Bistable Switch/70.png]

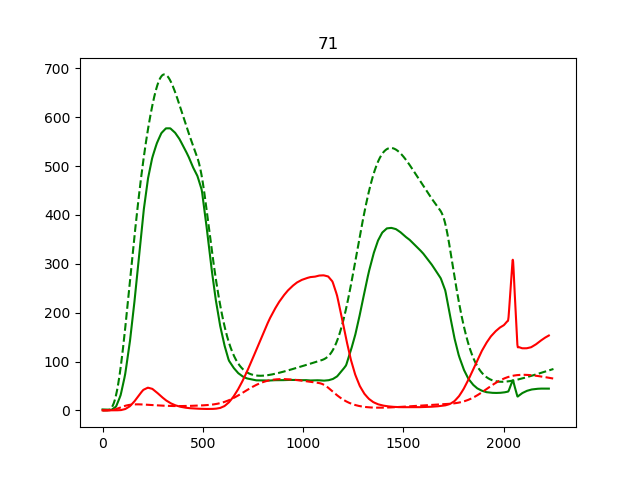

Supplement: Supplementary file 6 — Supplementary Dataset 3 [file 41467_2022_31306_MOESM6_ESM.zip › Individual Simulations Bistable Switch/71.png]

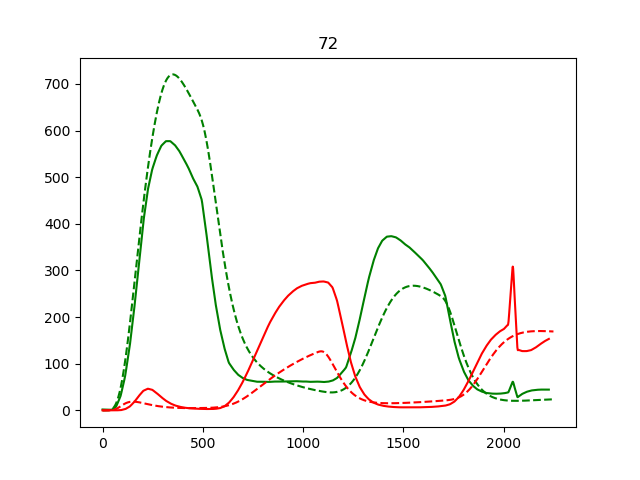

Supplement: Supplementary file 6 — Supplementary Dataset 3 [file 41467_2022_31306_MOESM6_ESM.zip › Individual Simulations Bistable Switch/72.png]

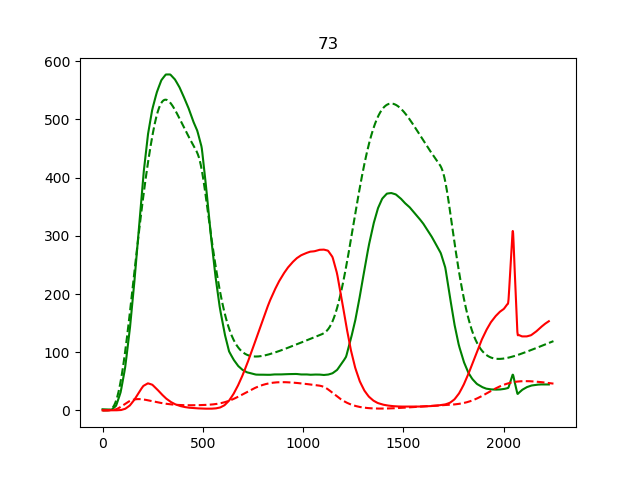

Supplement: Supplementary file 6 — Supplementary Dataset 3 [file 41467_2022_31306_MOESM6_ESM.zip › Individual Simulations Bistable Switch/73.png]

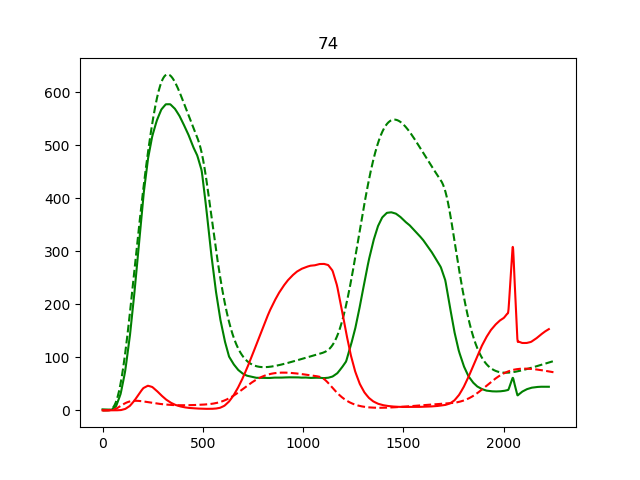

Supplement: Supplementary file 6 — Supplementary Dataset 3 [file 41467_2022_31306_MOESM6_ESM.zip › Individual Simulations Bistable Switch/74.png]

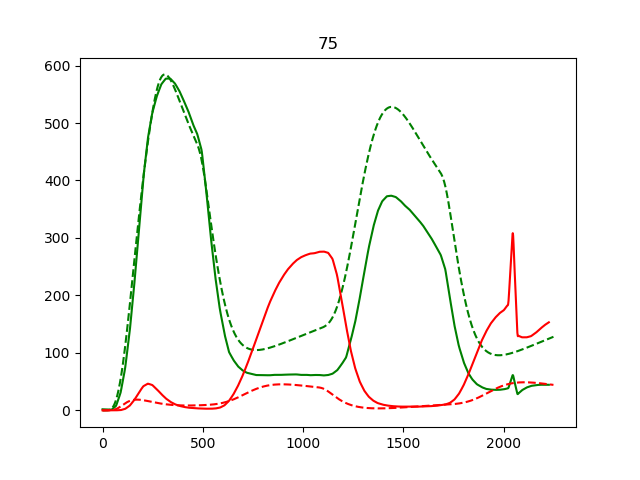

Supplement: Supplementary file 6 — Supplementary Dataset 3 [file 41467_2022_31306_MOESM6_ESM.zip › Individual Simulations Bistable Switch/75.png]

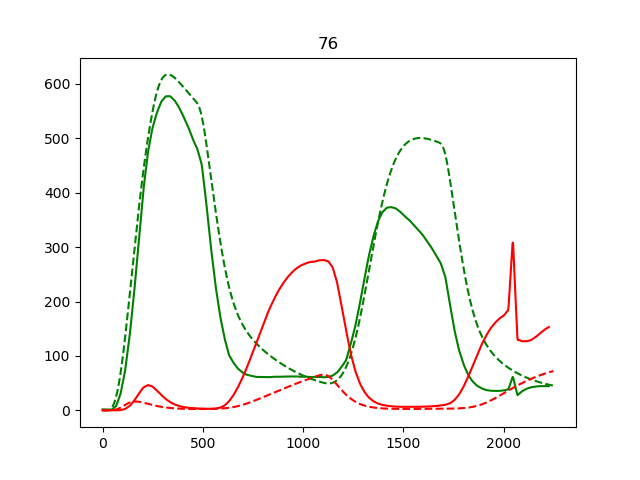

Supplement: Supplementary file 6 — Supplementary Dataset 3 [file 41467_2022_31306_MOESM6_ESM.zip › Individual Simulations Bistable Switch/76.png]

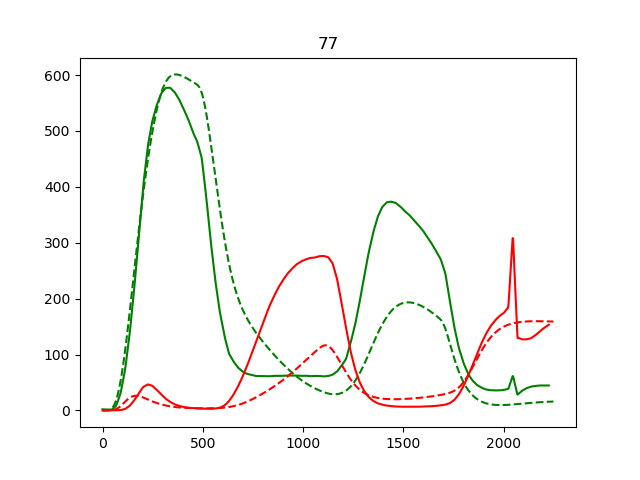

Supplement: Supplementary file 6 — Supplementary Dataset 3 [file 41467_2022_31306_MOESM6_ESM.zip › Individual Simulations Bistable Switch/77.png]

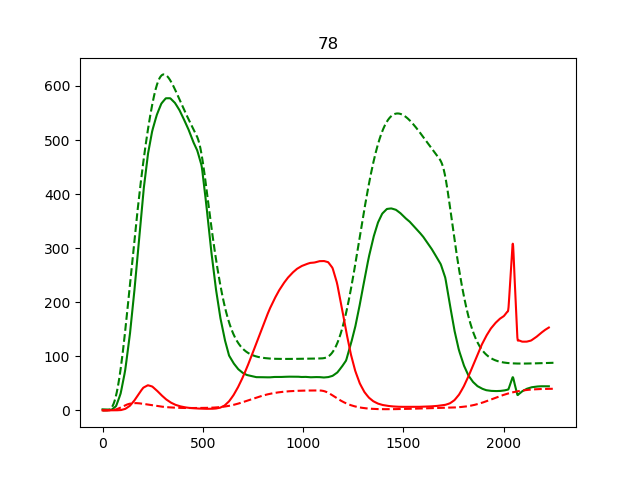

Supplement: Supplementary file 6 — Supplementary Dataset 3 [file 41467_2022_31306_MOESM6_ESM.zip › Individual Simulations Bistable Switch/78.png]

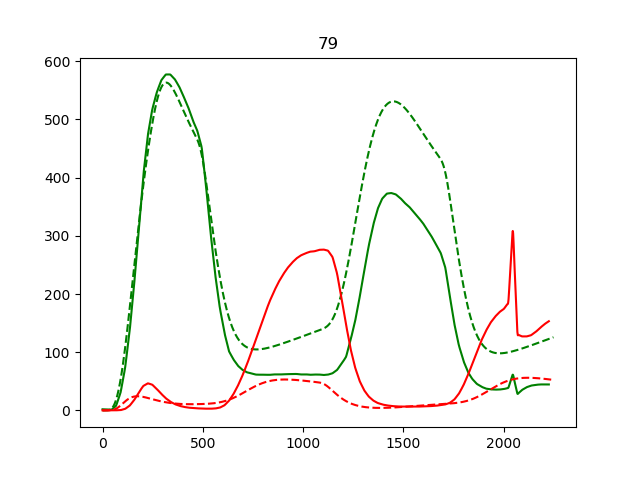

Supplement: Supplementary file 6 — Supplementary Dataset 3 [file 41467_2022_31306_MOESM6_ESM.zip › Individual Simulations Bistable Switch/79.png]

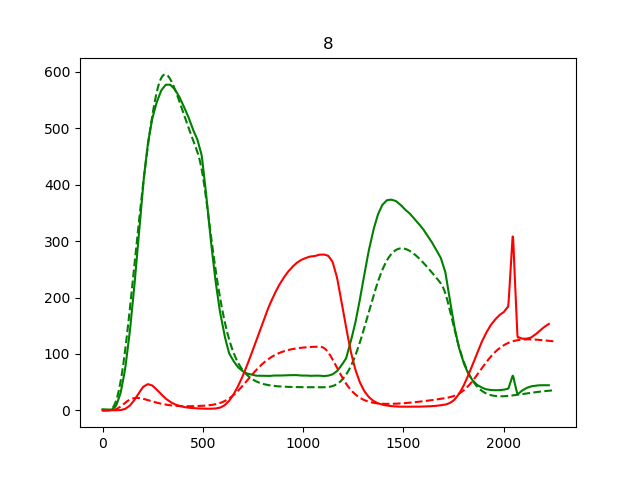

Supplement: Supplementary file 6 — Supplementary Dataset 3 [file 41467_2022_31306_MOESM6_ESM.zip › Individual Simulations Bistable Switch/8.png]

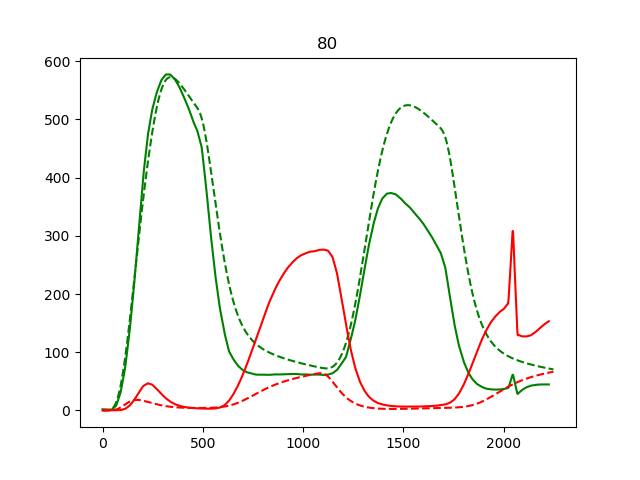

Supplement: Supplementary file 6 — Supplementary Dataset 3 [file 41467_2022_31306_MOESM6_ESM.zip › Individual Simulations Bistable Switch/80.png]

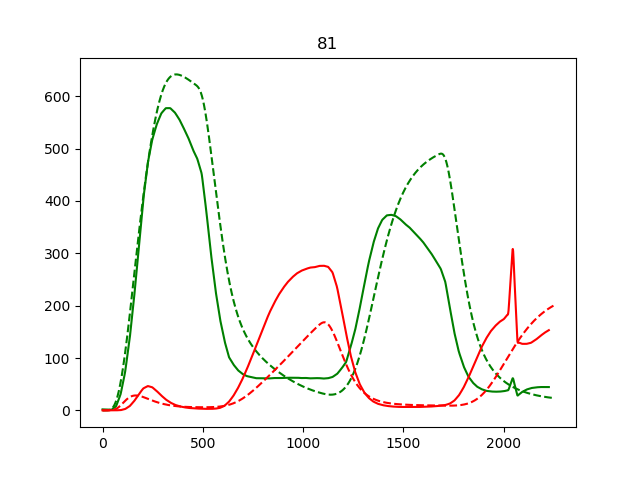

Supplement: Supplementary file 6 — Supplementary Dataset 3 [file 41467_2022_31306_MOESM6_ESM.zip › Individual Simulations Bistable Switch/81.png]

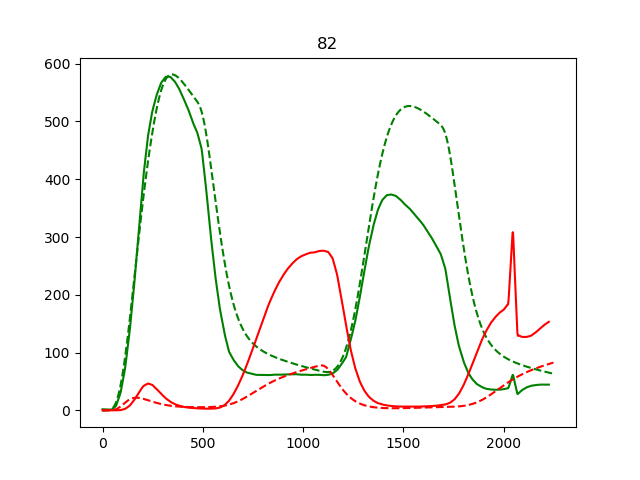

Supplement: Supplementary file 6 — Supplementary Dataset 3 [file 41467_2022_31306_MOESM6_ESM.zip › Individual Simulations Bistable Switch/82.png]

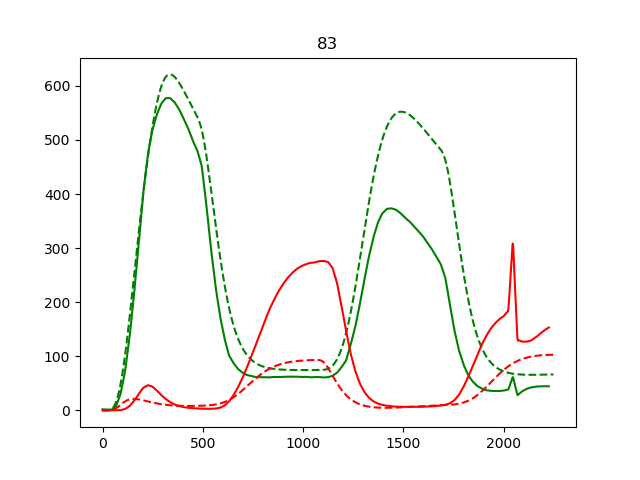

Supplement: Supplementary file 6 — Supplementary Dataset 3 [file 41467_2022_31306_MOESM6_ESM.zip › Individual Simulations Bistable Switch/83.png]

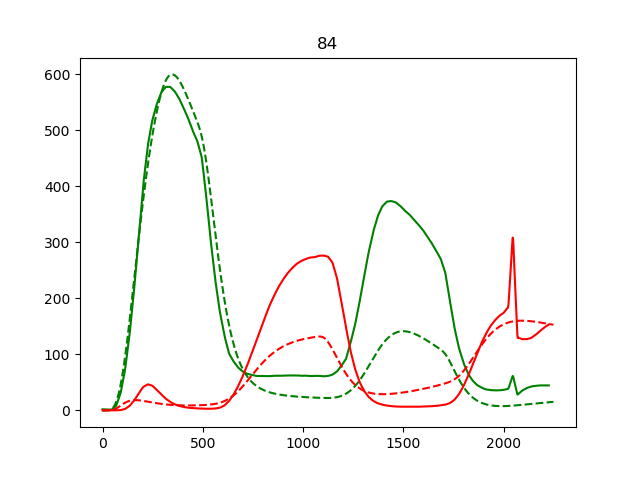

Supplement: Supplementary file 6 — Supplementary Dataset 3 [file 41467_2022_31306_MOESM6_ESM.zip › Individual Simulations Bistable Switch/84.png]

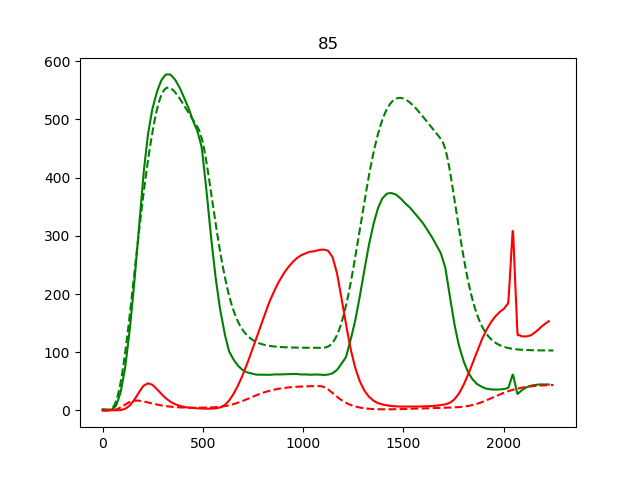

Supplement: Supplementary file 6 — Supplementary Dataset 3 [file 41467_2022_31306_MOESM6_ESM.zip › Individual Simulations Bistable Switch/85.png]

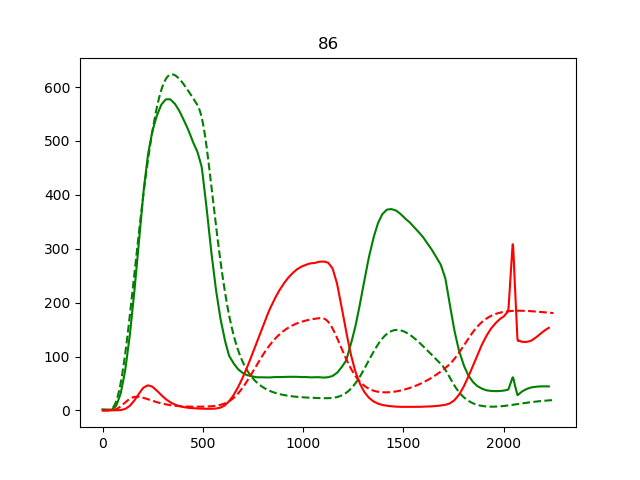

Supplement: Supplementary file 6 — Supplementary Dataset 3 [file 41467_2022_31306_MOESM6_ESM.zip › Individual Simulations Bistable Switch/86.png]

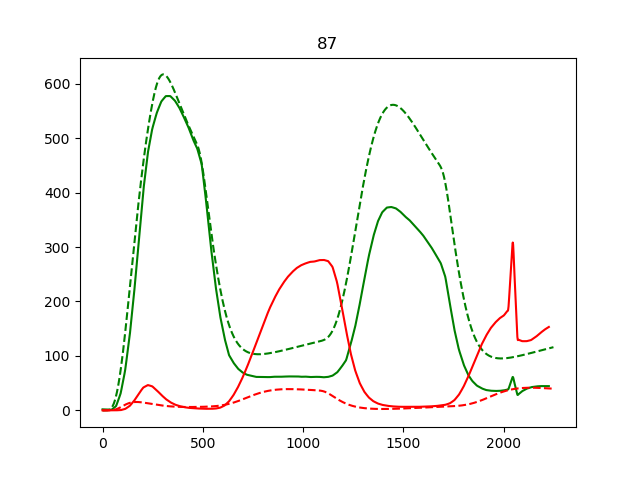

Supplement: Supplementary file 6 — Supplementary Dataset 3 [file 41467_2022_31306_MOESM6_ESM.zip › Individual Simulations Bistable Switch/87.png]

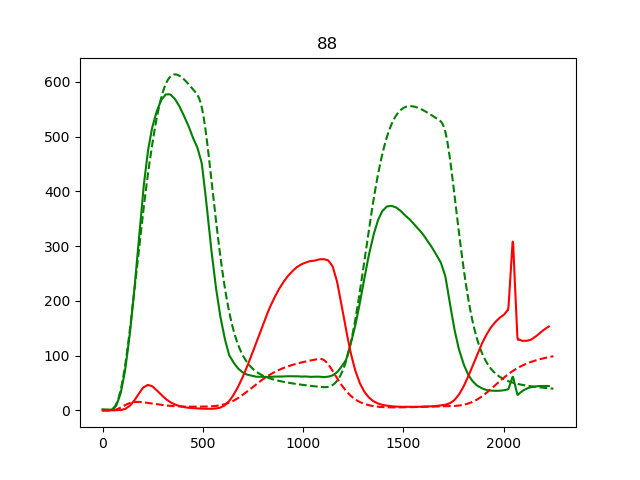

Supplement: Supplementary file 6 — Supplementary Dataset 3 [file 41467_2022_31306_MOESM6_ESM.zip › Individual Simulations Bistable Switch/88.png]

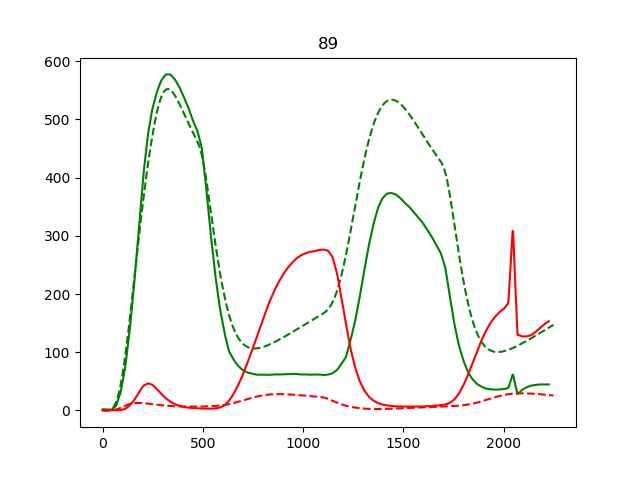

Supplement: Supplementary file 6 — Supplementary Dataset 3 [file 41467_2022_31306_MOESM6_ESM.zip › Individual Simulations Bistable Switch/89.png]

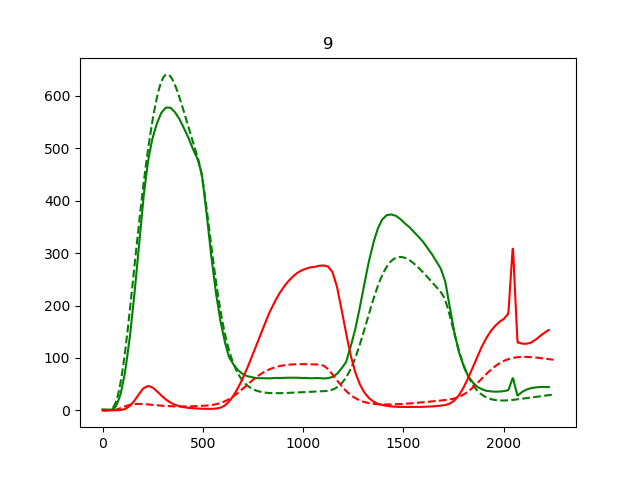

Supplement: Supplementary file 6 — Supplementary Dataset 3 [file 41467_2022_31306_MOESM6_ESM.zip › Individual Simulations Bistable Switch/9.png]

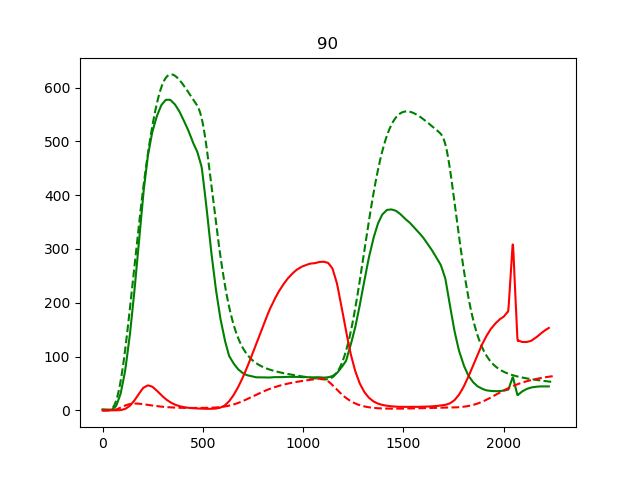

Supplement: Supplementary file 6 — Supplementary Dataset 3 [file 41467_2022_31306_MOESM6_ESM.zip › Individual Simulations Bistable Switch/90.png]

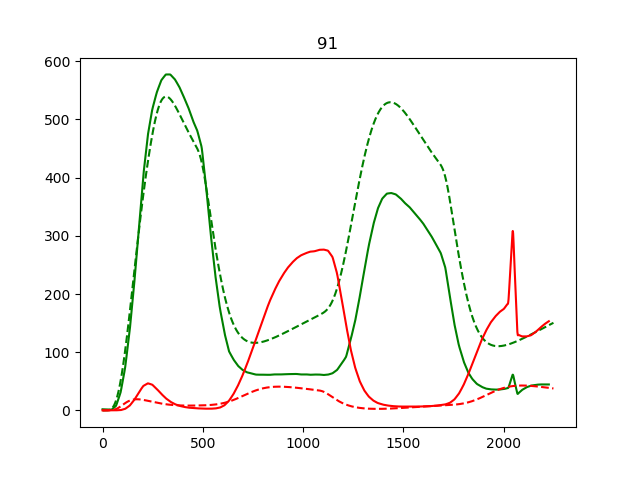

Supplement: Supplementary file 6 — Supplementary Dataset 3 [file 41467_2022_31306_MOESM6_ESM.zip › Individual Simulations Bistable Switch/91.png]

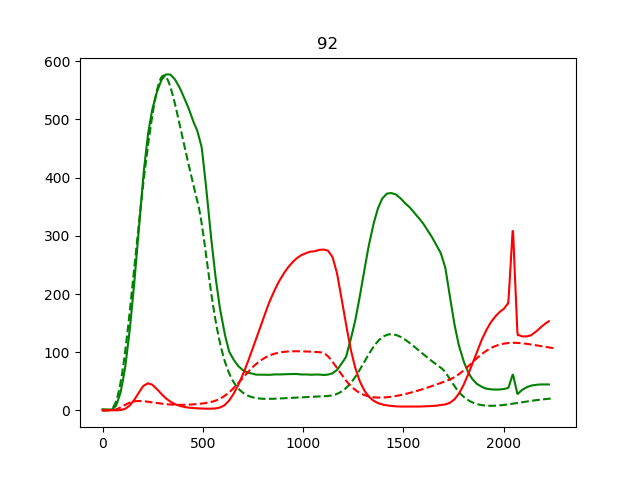

Supplement: Supplementary file 6 — Supplementary Dataset 3 [file 41467_2022_31306_MOESM6_ESM.zip › Individual Simulations Bistable Switch/92.png]

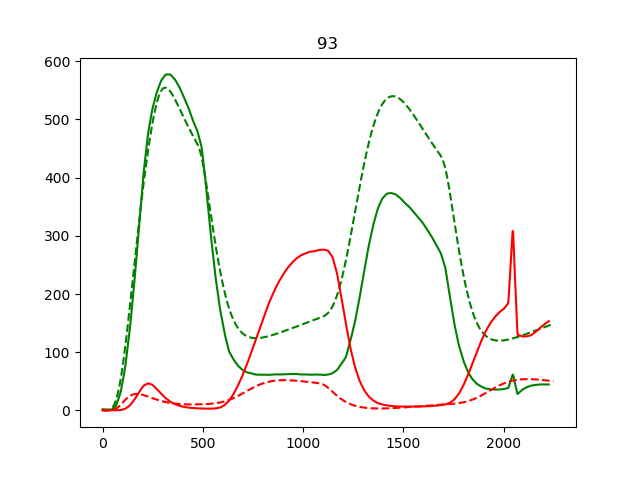

Supplement: Supplementary file 6 — Supplementary Dataset 3 [file 41467_2022_31306_MOESM6_ESM.zip › Individual Simulations Bistable Switch/93.png]

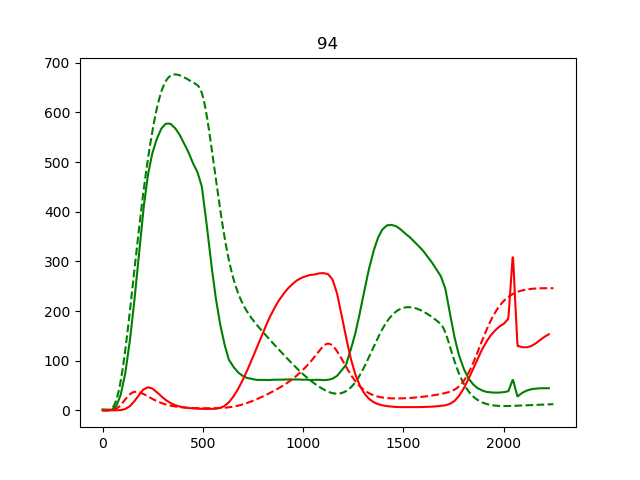

Supplement: Supplementary file 6 — Supplementary Dataset 3 [file 41467_2022_31306_MOESM6_ESM.zip › Individual Simulations Bistable Switch/94.png]

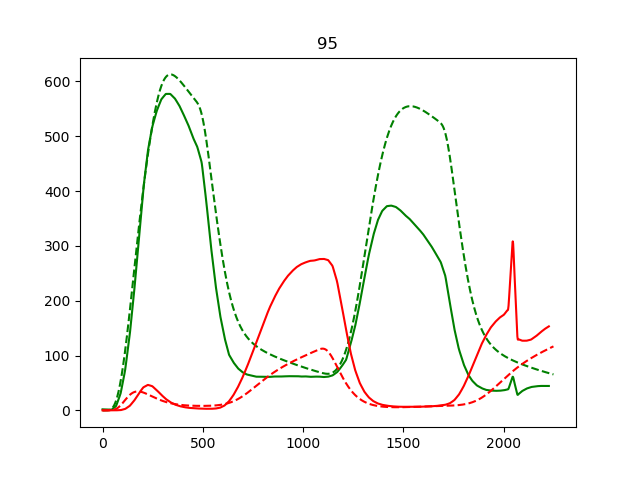

Supplement: Supplementary file 6 — Supplementary Dataset 3 [file 41467_2022_31306_MOESM6_ESM.zip › Individual Simulations Bistable Switch/95.png]

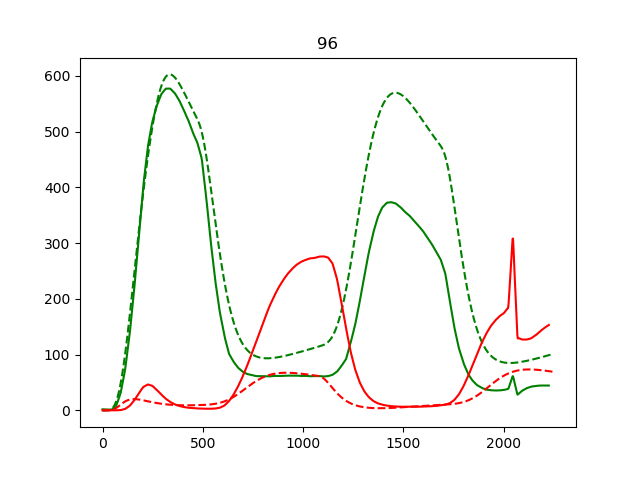

Supplement: Supplementary file 6 — Supplementary Dataset 3 [file 41467_2022_31306_MOESM6_ESM.zip › Individual Simulations Bistable Switch/96.png]

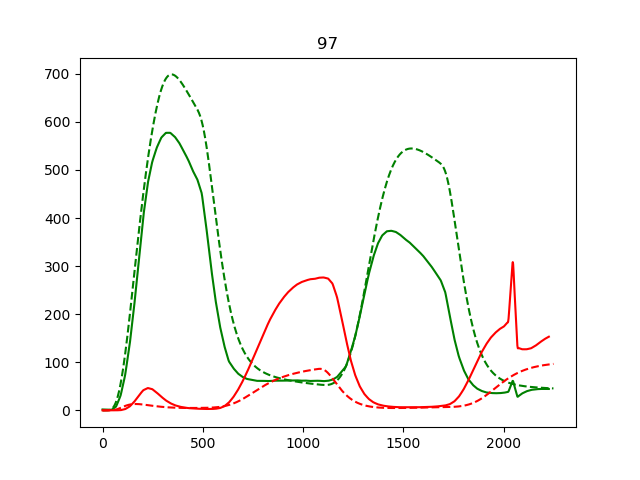

Supplement: Supplementary file 6 — Supplementary Dataset 3 [file 41467_2022_31306_MOESM6_ESM.zip › Individual Simulations Bistable Switch/97.png]
